# Unraveling cross-reactivity of anti-glycan IgG responses in filarial nematode infections

**DOI:** 10.3389/fimmu.2023.1102344

**Published:** 2023-03-06

**Authors:** Laudine M. C. Petralia, Angela van Diepen, Dieu-Linh Nguyen, Lena A. Lokker, Erliyani Sartono, Sasisekhar Bennuru, Thomas B. Nutman, Kenneth Pfarr, Achim Hoerauf, Samuel Wanji, Jeremy M. Foster, Cornelis H. Hokke

**Affiliations:** ^1^ Department of Parasitology, Leiden University – Center of Infectious Diseases, Leiden University Medical Center, Leiden, Netherlands; ^2^ Division of Protein Expression & Modification, New England Biolabs, Ipswich, MA, United States; ^3^ Laboratory of Parasitic Diseases, National Institutes of Health, Bethesda, MD, United States; ^4^ Institute for Medical Microbiology, Immunology and Parasitology, University Hospital Bonn, Bonn, Germany; ^5^ German Center for Infection Research (DZIF), Partner Site Bonn-Cologne, Bonn, Germany; ^6^ Epidemiology and Control of Infectious Diseases, Department of Microbiology and Parasitology, University of Buea, Buea, Cameroon

**Keywords:** filariasis, glycan microarray, MALDI-TOF-MS and MS/MS, glycomics, helminth antigen, glycans, antibody response, infection markers

## Abstract

Parasitic nematodes responsible for filarial diseases cause chronic disablement in humans worldwide. Elimination programs have substantially reduced the rate of infection in certain areas, but limitations of current diagnostics for population surveillance have been pointed out and improved assays are needed to reach the elimination targets. While serological tests detecting antibodies to parasite antigens are convenient tools, those currently available are compromised by the occurrence of antibodies cross-reactive between nematodes, as well as by the presence of residual antibodies in sera years after treatment and clearance of the infection. We recently characterized the N-linked and glycosphingolipid derived glycans of the parasitic nematode *Brugia malayi* and revealed the presence of various antigenic structures that triggered immunoglobulin G (IgG) responses in infected individuals. To address the specificity of IgG binding to these glycan antigens, we screened microarrays containing *Brugia malayi* glycans with plasma from uninfected individuals and from individuals infected with *Loa loa*, *Onchocerca volvulus*, *Mansonella perstans* and *Wuchereria bancrofti*, four closely related filarial nematodes. IgG to a restricted subset of cross-reactive glycans was observed in infection plasmas from all four species. In plasma from *Onchocerca volvulus* and *Mansonella perstans* infected individuals, IgG binding to many more glycans was additionally detected, resulting in total IgG responses similar to the ones of *Brugia malayi* infected individuals. For these infection groups, *Brugia malayi*, *Onchocerca volvulus* and *Mansonella perstans*, we further studied the different IgG subclasses to *Brugia malayi* glycans. In all three infections, IgG1 and IgG2 appeared to be the major subclasses involved in response to glycan antigens. Interestingly, in *Brugia malayi* infected individuals, we observed a marked reduction in particular in IgG2 to parasite glycans post-treatment with anthelminthic, suggesting a promising potential for diagnostic applications. Thus, we compared the IgG response to a broad repertoire of *Brugia malayi* glycans in individuals infected with various filarial nematodes. We identified broadly cross-reactive and more specific glycan targets, extending the currently scarce knowledge of filarial nematode glycosylation and host anti-glycan antibody response. We believe that our initial findings could be further exploited to develop disease-specific diagnostics as part of an integrated approach for filarial disease control.

## Introduction

Parasitic filarial nematodes have been known to infect humans for many centuries (1), causing various types of diseases called filariasis (2). The lymphatic form, resulting from an infection with either *Brugia malayi*, *Brugia timori* or *Wuchereria bancrofti*, is the most widespread, with over 50 million people infected worldwide in 2018 (3). *Loa loa*, *Mansonella streptocerca* and *Onchocerca volvulus* cause subcutaneous forms of filariasis while *Mansonella ozzardi* and *Mansonella perstans* are responsible for serous cavity filariasis (2). Altogether, filarial nematodes represent a great burden in endemic areas, and both onchocerciasis and lymphatic filariasis (LF) are targeted for elimination as part of the World Health Organization (WHO) roadmap for 2030 (4). To achieve this goal, strategic intervention programs ranging from preventive chemotherapy to vector control (mosquitoes for LF and black flies for onchocerciasis) have to be implemented or strengthened. Stopping infection spread in populations in endemic areas relies on mass drug administration (MDA) of ivermectin for onchocerciasis (5) and of a cocktail of ivermectin, diethylcarbamazine (DEC) and albendazole (IDA triple-therapy) for LF (6), except in Africa, where a combination of albendazole and ivermectin is used, due to contraindications of DEC in onchocerchiasis (7). Essential to the success of these programs are diagnostic tools for confirmation of disease, mapping, screening, decision of treatment administration, monitoring and surveillance. For both LF and onchocerciasis, the current diagnostic options are deemed inadequate to complete part or all of these tasks, particularly when it comes to post-treatment surveillance which is crucial to avoid disease resurgence. Diagnosis often still relies on identification by microscopy of microfilariae in blood thick-smear for LF (8) and in skin snip biopsies for onchocerciasis (9). Alternative and more convenient serological methods face several issues such as non-availability in field settings, the difficulty to distinguish past from current infections due to the presence of residual antibodies after parasite clearance, and cross-reactivity between filarial infections (10–12).

Detection of host antibodies to parasite antigens does however have many advantages as a diagnostic strategy since antibodies have been shown to appear earlier than detectable levels of circulating filarial antigen (CFA) (13) and greater sensitivities have been indicated (13, 14). Infected individuals are exposed to a variety of parasite molecules of diverse nature (15–18) to which antibodies are raised, including antibodies to carbohydrates (glycans) (19, 20). We recently reported a comprehensive characterization of *B. malayi* N-linked and glycosphingolipid (GSL) derived glycans. Using a glycan microarray workflow, we demonstrated that many glycan motifs expressed by the parasite were antigenic and that *B. malayi*-infected individuals showed a preferential immunoglobulin (Ig) G binding to a broad subset of glycans (21). Importantly, IgG to these glycans decreased substantially after treatment with DEC anthelminthic, suggesting that IgG to glycan antigens could be exploited as a marker of current or recent infection in the context of LF. Certain features of nematode glycans, such as the common presence of antigenic phosphorylcholine (PC) substituents are already known to be widespread throughout the filarial nematode phylum (22). However, due to the overall lack of knowledge on filarial nematode glycosylation, for PC and many other glycan epitopes identified in our previous study, their potential cross-reactivity with host antibodies induced by other filarial infections has remained poorly understood. Studies into the role of IgG to parasite crude antigens have been well documented in the past both for LF (15, 23) and onchocerciasis (24). Notably, it is known that nematode parasites are typically characterized by T helper 2 (Th2) immunity inducing unusually high levels of IgG4 and IgE antibodies to the parasite in infected humans (25). Nonetheless, with the exception of *B. malayi (* 21), the humoral immune response to filarial carbohydrate antigens has remained largely unexplored.

Thus, in addition to the previously described anti-glycan IgG response from *B. malayi*-infected individuals, we studied IgG responses in plasma of individuals infected with *L. loa*, *M. perstans*, *O. volvulus* or *W. bancrofti* to address cross-reactivity with *B. malayi* glycans. Interestingly, substantial IgG binding to a broad range of glycan structures of *B. malayi* was also observed in plasmas from *M. perstans-* and *O. volvulus*-infected individuals. In comparison, plasma from *L. loa-* and *W. bancrofti*-infected individuals exhibited an overall weaker IgG response, that was restricted to a subset of GSL glycans. Further, evaluation of the antibody subclasses indicated that the reactivity to the glycan antigens across the most reactive infection groups (*B. malayi*, *M. perstans* and *O. volvulus*) was predominantly comprised of IgG1 and IgG2. Reduction of IgG1, IgG2 and IgG4 levels was observed after treatment with DEC. In particular, IgG2 decreased significantly for all individuals tested and to a majority of glycan structures, which may be exploited further for the development of a serology-based method for diagnosis of LF.

## Materials and methods

### Infection plasma and ethics statement

#### B. malayi infection plasma

Plasma from individuals infected with *B. malayi* were described in our previous glycan array study (21). Plasma originated from two sets of five participants obtained from two distinct studies conducted in Indonesia in accordance with the Declaration of Helsinki and the guidelines of the Indonesian Department of Health and Human services. The purposes of the studies and procedures involved were explained to all participants, and only those granting informed consent were enrolled as study participants. The first set of individuals was involved in previously published work (26–28), while the second set was from another study in which infected individuals were treated with DEC anthelminthic after initial sampling in 1990 (15, 29). Thus, for each individual, we had access to plasma sampled pre-treatment and almost two-years post-infection. Details on parasitological parameters regarding the infection status of the subjects (microfilaraemia) and filarial antigen specific IgG titers were obtained from the original studies where the experimental procedures have been described (15, 29). Briefly, blood microfilaremia was estimated by parasitological examination of 1mL of filtered (Nuclepore) venous blood. Blood collection took place at nighttime between 8PM and 11PM, in accordance with the periodicity of the microfilariae in the area. Microfilaraemia data are shown in **Table S1.A** for each individual. Note that for the second set, the microfilaraemia pre-treatment with DEC is indicated, since all individuals were amicrofilaraemic post-treatment. Filarial antigen specific IgG1-4 titers to *B. malayi* adult somatic extract antigen (BmA) were estimated by enzyme-linked immunosorbent assay (ELISA). ELISA protocols were performed as detailed earlier (15, 29) and used monoclonal anti-isotype antibodies to detect plasma IgG1-4 (**Table S1.A**).

#### Other filarial nematode infection plasma

Plasma from individuals infected with either *L. loa*, *M. perstans*, *O. volvulus* or *W. bancrofti* were all obtained from the same study (n = 6 for each infection). Plasma samples from Cameroonian patients were collected and archived as part of the Bill & Melinda Gates Foundation consortium project “Rapid and high throughput diagnosis of *Onchocerca volvulus* infections” (RADIO; OPP1083888; **Table S1.B**). Ethical clearance for the collection of blood samples for biomarker research was obtained from the National Ethics Committee for Human Health Research (Ref: N°2015/09/641/CE/CNERSH/S). In addition, administrative authorization was received from the Ministry of Public Health (Ref. No48/L/MINSANTE/SG/DLMEP/PNLO). All work was conducted in compliance with the Helsinki Declaration on the use of humans in biomedical research. Volunteers received detailed information about the study and how the samples would be biobanked and used for biomarker research to discover new biomarkers for their infections. All volunteers provided written informed consent before donating blood. Infected individuals were identified by palpation of onchocercomata (*O. volvulus*) and identification of microfilariae by microscopy of skin biopsies (*O. volvulus*) or blood (*L. loa*, *M. perstans*, *W. bancrofti*) as described (11, 30, 31).

#### Control plasma

Plasma from six non-infected African donors from Ghana collected in parallel to the Cameroon study were used as controls, in addition to plasma from five non-endemic European donors, that were obtained from Sanquin (Dutch blood bank).

### Glycan microarray

Unless specified otherwise, reagents mentioned in the experimental section were obtained from Sigma-Aldrich.

#### Construction of a B. malayi N-linked and GSL glycan microarray

A glycan microarray of native parasite N-linked and GSL glycans was previously constructed and validated (21). Briefly, glycans were isolated from adult female worm proteins and lipids using enzymatic treatment with N-Glycosidase (PNGase) F (#P0709, New England Biolabs (NEB)) and endoglycoceramidase I (EGCase I, #P0773, NEB) respectively. Released glycans were labelled with 2-aminobenzoic acid (2-AA) and purified using a two-dimensional ultra-high performance liquid chromatography (UHPLC) procedure consisting of an initial separation using hydrophilic interaction chromatography followed by a reverse-phase purification on an octadecylsilane (C18) column. Glycan structures in UHPLC fractions were determined using matrix-assisted laser desorption/ionization-time of flight-mass spectrometry (MALDI-TOF-MS) analysis as detailed previously (21) and summarized below. Fractions containing isolated individual - or small pools of mixed - glycan structures with a minimum glycan content of 20 pmol were selected for array printing. This amounted to a total of 35 N-glycan-containing fractions and 17 GSL glycan-containing fractions, largely covering the defined N-linked and GSL derived glycan repertoires of the parasite in terms of structural diversity. In addition, to evaluate the effect of PC substituents and fucose residues in antibody binding, aliquots of 4 GSL glycan fractions were treated with hydrofluoric acid (HF), a reagent removing α1-2,3,4 linked fucoses and PC, leaving only unsubstituted GSL glycan backbones. HF treatment was performed using the conditions detailed below (see Glycan sequencing section) and fractions were cleaned-up by reverse-phase UHPLC before being added to the array selection, amounting to 21 GSL glycan fractions on the array. A detailed overview of the printed glycan fractions has been published as supplementary data (Table S5) in our previous study (21).

Printing was performed as described previously (32, 33). Briefly, fractions were aliquoted to a 384-well V-bottom polypropylene plate (#784201, Greiner Bio-One) to create a glycan library plate containing 1, 3, 10 or 30 µM printing solutions in 20 μL of 1x spotting buffer (Nexterion Spot, Nexterion #1066029, Schott AG) with 10% DMSO. Additionally, 11 wells filled solely with spotting buffer were included for control of non-specific binding to the array. Samples and controls were printed in triplicate to epoxysilane-coated glass slides (Slide E, Nexterion #1066643, Schott AG), using the Microgrid 600 microarrayer (Genomic Solutions) equipped with SMP3 pins that deposit 0.7 nL upon each contact. Each array was printed eight times per glass slide with a 0.245 mm spacing between spots and 4.60 mm spacing between the printing areas of each array.

#### Glycan array screening

##### Binding assay

The panel of control and infected human plasma samples described above was used to screen the glycan microarrays for anti-glycan antibody binding, following the procedures described previously for slide incubation, scanning and data handling (32–35) with slight modifications where applicable. First, array blocking was performed with a solution of 2% BSA and 50 mM ethanolamine in PBS to cap residual epoxides on the slides. Next, plasma samples were diluted 1:100 in PBS, 0.01% Tween20 with 1% BSA and incubated on the array. Anti-glycan IgG from plasma was detected using goat anti-human IgG (Fc-specific) Cy3-conjugate (Sigma-Aldrich #C-2571) as secondary antibody. Specific binding of the different IgG subclasses was studied using mouse anti-human isotype antibodies purchased from SouthernBiotech. Alexa Fluor^®^ 555-conjugates were used for detection of IgG1 and IgG2 (#9052-32 and #9070-32) and Alexa Fluor^®^ 647-conjugates for IgG3 and IgG4 (#9210-31 and #9190-31). Secondary antibody used for the detection of total plasma IgG was diluted in PBS, 0.01% Tween20 with 1% BSA in a 1:1000 ratio while a 1:400 dilution was used for the isotype specific antibodies, with the exception of anti-human IgG4 where a 1:250 dilution was applied. Successive rinses were performed between each incubation step with PBS containing 0.05% Tween20 and with PBS. Finally, slides were washed with MilliQ water (MQ) prior to drying and scanning.

##### Scanning and data analysis

Fluorescence was detected using a G2565BA scanner (Agilent Technologies) at 10 µm resolution using two laser channels at 532 nm and 633 nm. Image analysis was processed with GenePix Pro 7.0 software (Molecular Devices). Spots were aligned and resized according to published methods (36). Next, data were exported to Excel, corrected for background using average fluorescence intensity of the control blank spots as a baseline, and intensities were averaged for each triplicate.

GLycan Array Dashboard (GLAD) (37), from the glycotoolkit website (glycotoolkit.com/Tools/GLAD/) hosted by the National Center for Functional Glycomics (NCFG), was used for data visualization, data mining, generation of heatmaps and boxplot graphs.

##### Statistical analysis

Statistical data analysis was performed under the publicly available statistical programming language R (http://CRAN.R-project.org/ , version 3.5.0) using Bayesian statistics. Significant differences between log_2_ normalized fluorescence intensities of the various groups were assessed using the R/Bioconductor software package *limma (* 38, 39) as described previously (21).

### Structural characterization of *O. volvulus* glycans

#### Enzymatic release of N-glycans and GSL glycans from O. volvulus

*O. volvulus* adult female worms that were pulverized in liquid N_2_ were used for the extraction of N-linked and GSL glycans, following the procedure mentioned above for *B. malayi*. 2-AA-labeled N-linked and GSL glycan profiles were measured using MALDI-TOF-MS. Next, glycans were similarly purified using the two-dimensional UHPLC workflow. For structural elucidation, selected glycan samples – total N-glycans, N-glycan and GSL glycan-containing UHPLC-fractions – were subjected to glycan sequencing and analyzed by MALDI-TOF-MS analysis.

#### Glycan sequencing

A selection of glycan samples was subjected to selective degradation of glycans using treatment with HF or exoglycosidases to elucidate the main structures expressed by *O. volvulus*. The following enzymes were all obtained from NEB: α1-2,3,6 Mannosidase (P0768), β-N-Acetylglucosaminidase S (P0744), β-N-Acetylhexosaminidase_f_ (P0721), α1-3,6 Galactosidase (P0731) and α1-3,4,6 Galactosidase (P0747). Exoglycosidases were used alone or in combination to digest selected glycan fractions. Small aliquots of 2-AA-labeled glycans (typically 1-2 µL) were mixed with 1-2 µL of the exoglycosidase(s) in recommended buffer (Glycobuffer 1 or 4, NEB). According to the manufacturer’s instructions, the reaction was supplemented with BSA (0.1 µg/mL final concentration) when α-Galactosidases were used, and zinc (2 mM Zn^2+^ final concentration) was added to reactions with α1-2,3,6 Mannosidase. Final reactions were adjusted with MQ to 10 µL total volume and incubated overnight at 37°C. An undigested control consisting of all reagents except enzyme(s) was performed in parallel to each exoglycosidase digestion.

HF was used by mixing small aliquots of 2-AA-labeled glycans suspended in MQ with cold 48% HF in a 1:100 ratio. Samples were incubated at 4°C for 48h and HF was then removed by evaporation under nitrogen (N_2_) flow. Multiple washes were performed by addition and subsequent N_2_ evaporation of MeOH. Finally, the HF treated 2-AA-labeled glycan samples were re-dissolved in MQ.

After treatment with exoglycosidase(s) and HF, enzyme removal and clean-up was performed using C18 Millipore^®^ Zip-Tips (#Z720046-960EA) as described previously (40).

#### MALDI-TOF-MS and MALDI-TOF-MS/MS analysis

2-AA-labeled glycans from *O. volvulus* were analyzed using MALDI-TOF-MS as described previously (21) using a Bruker rapifleX^®^ instrument. Briefly, glycans were spotted onto a 384 well steel polished target plate. 2-AA-labeled glycans solubilized in MQ were mixed on the plate with 20 mg/ml 2,5-dihydroxybenzoic acid (DHB) matrix (#8201346, Bruker Daltonics) in 30% ACN, while products of glycan sequencing (HF treatments, exoglycosidase digestions and corresponding undigested controls) were directly eluted onto the plate in 50% ACN, 0.1% TFA mixed with DHB (10 mg/ml) at the end of the enzyme removal with C18 Millipore^®^ Zip-Tips. All spectra were obtained in negative-ion reflectron mode after external calibration with Bruker^®^ peptide calibration mix (#8206195, Bruker Daltonics). Spectra were obtained over a mass window of *m/z* 700 – 3500 with ion suppression below *m/z* 700. A minimum of 20,000 shots (2000 Hz) were obtained by manual selection of “sweet spots”. The software FlexAnalysis (Version 3.4, Build 50, Bruker Daltonics) was used for data processing including smoothing of the spectra (Savitzky Golay algorithm, peak width: m/z 0.06, 1 cycle), baseline subtraction (Tophat algorithm) and manual peak picking. Peaks with a signal-to-noise ratio below 5 were excluded as well as known non-glycan peaks such as glucose polymers. Glycan compositions correlating with the deprotonated masses of the selected peaks were assigned using the GlycoPeakfinder^®^ (GlycoWorkBench, Version 3, 29 June 2007 (41)). A deviation of 300 ppm was allowed and the 2-AA label was taken into account as a fixed reducing-end modification. Tandem MS (MS/MS) was performed for structural elucidation *via* fragmentation ion analysis by MALDI-TOF/TOF on selected ions using the rapifleX^®^ mass spectrometer in negative-ion mode.

Most probable glycan compositions were assigned based on knowledge from literature, analogy with *B. malayi* and results of MS/MS and glycan sequencing procedures.

## Results

### Cross-reactivity of anti-glycan IgG responses in human filarial infections

#### Comparison of total IgG responses to B. malayi glycans

Given that several glycan features found in *B. malayi* are shared with other filarial nematodes, *e.g.* presence of PC (42) or terminal α-linked galactose (α-Gal) in GSLs (43), we evaluated the potential IgG cross-reactivity in plasma derived from other filarial nematode infections – *L. loa*, *M. perstans*, *O. volvulus* and *W. bancrofti* (n = 6 for each infection) – to *B. malayi* N-linked and GSL-derived glycans printed on the array. Results were compared to those obtained for *B. malayi*-infected individuals (n = 10), Ghanaian individuals not infected with filarial nematodes (n = 6) and uninfected European donors (n = 5).

**Figure 1:**
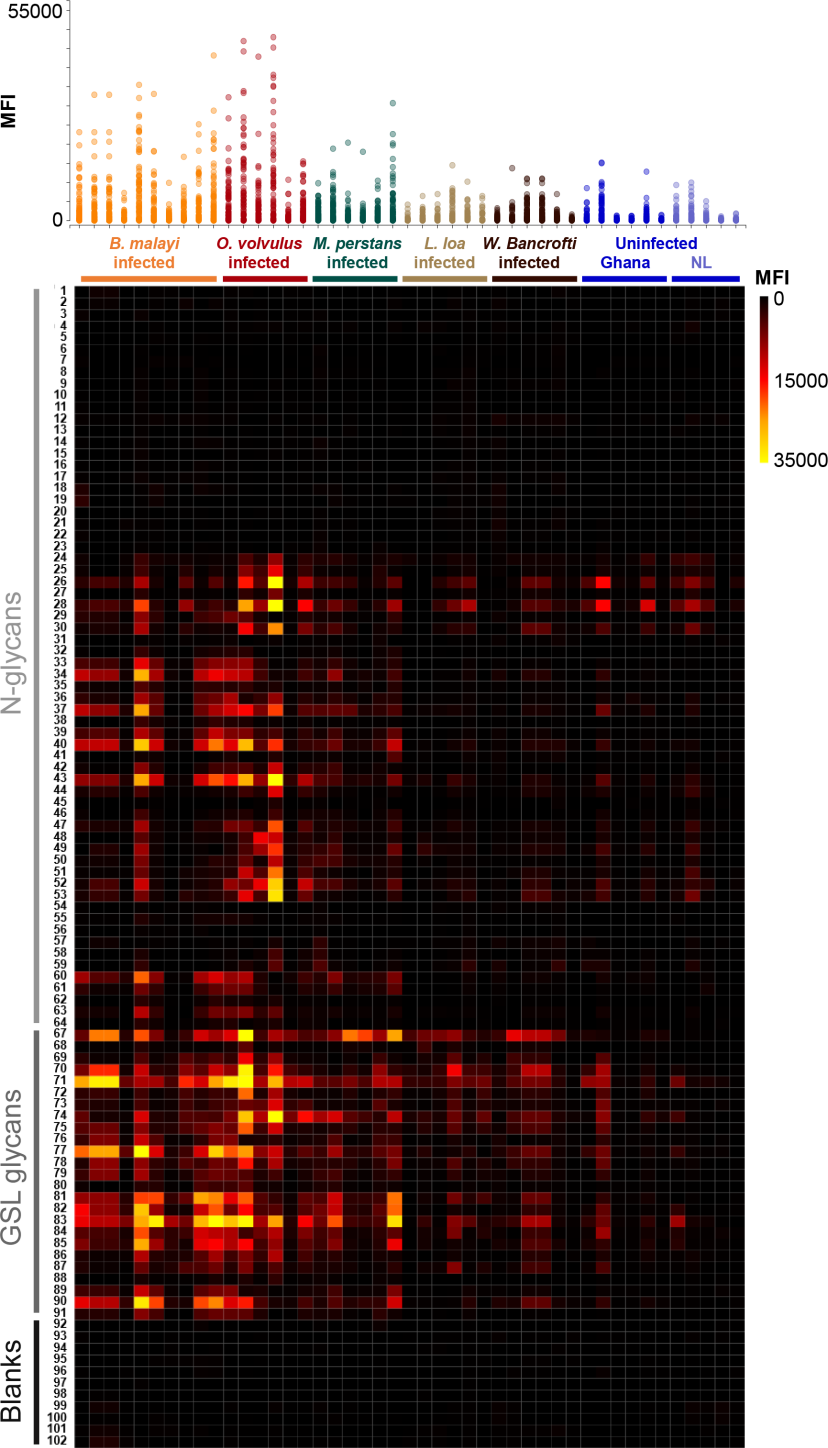
IgG responses to *B. malayi* N-linked and GSL glycans in plasma from various filarial-infected and uninfected individuals. *B. malayi* glycan microarrays were screened with plasma from individuals infected with *B. malayi* (n = 10), *O. volvulus* (n = 6), *M. perstans* (n = 6), *L. loa* (n = 6), *W. bancrofti* (n = 6) and with plasma from uninfected individuals from Africa (Ghana, n = 6) and Europe (The Netherlands, NL, n = 5). Background corrected median fluorescence intensities (MFIs) resulting from IgG binding are shown on the upper graph for each donor (X-axis). Each spot on the Y-axis corresponds to a different glycan fraction or blank spot. The heatmap generated from these data allows the visualization of IgG from each donor (X-axis) to each array fraction (Y-axis) indicated by their ID numbers (21). The type of glycan in the fractions is indicated on the left: N-glycans, GSL glycans or Blanks (negative controls, no glycan). Raw data can be found in **Table S2**.

Substantial IgG binding to *B. malayi* glycans was detected in plasma from individuals infected with any of the other filarial nematodes when compared to plasma from uninfected individuals. In particular, for *O. volvulus*-infected individuals very high fluorescence levels were observed as glycan microarray read-out, and to a lesser extent for *M. perstans*-infected individuals (**Figure 1**). Comparatively, *L. loa* and *W. bancrofti* infection plasma showed lower IgG binding, with clear differences with non-endemic control plasma only apparent for a limited number of GSL containing fractions.

Statistical analysis using limma *F-*test for multiple-groups comparison confirmed these visual observations (**Table S2**). IgG binding was significantly higher to 37 glycan fractions for *B. malayi*-infected plasma, to 35 fractions for *O. volvulus* and to 24 fractions for *M. perstans* when compared to uninfected individuals. For the *L. loa*- and *W. bancrofti*-infected individuals, median fluorescence intensity (MFI) values significantly higher than both uninfected groups were only observed for 5 glycan fractions. Between the *B. malayi*, *M. perstans* and *O. volvulus* groups, significant differences in IgG binding were observed for only a limited number of fractions (5 glycan fractions for *B. malayi* vs *M. perstans*, 7 fractions for *B. malayi vs O. volvulus* and 4 fractions for *M. perstans vs O. volvulus*). This indicated highly similar anti-glycan IgG responses in these infections with a broad (cross-)reactivity to many glycan fractions and a similar absence of recognition of a restricted subset of glycans, clearly visible on the heatmap in **Figure 1**. Many differences were apparent when cross-comparing *B. malayi*-*, M. perstans*- or *O. volvulus*- to *L. loa*- or *W. bancrofti*-infected individuals, confirming a weaker IgG response from the two latter groups to the *B. malayi* glycans. Little IgG binding was observed for the two populations of uninfected individuals, with IgG binding in the Ghanaian control group only slightly but significantly higher than the European group for 8 fractions. The presence of IgG against *B. malayi* glycans in these uninfected Ghanaian individuals might be explained by possible previous infection with cross-reactive helminths or other pathogens, not endemic in Europe.

#### Identification of cross-reactive glycan motifs

The highest MFI values were observed for GSL glycan containing fractions. IgG binding was significantly higher (*p*<0.05) for 16 out of the 21 printed GSL glycan fractions in the *B. malayi*-infected group than for both uninfected groups. Similarly, MFI values were significantly higher (*p*<0.05) for 20 fractions in the *O. volvulus*-infected group and for 14 fractions in the *M. perstans* group compared to uninfected ones. This indicated that both *O. volvulus-* and *M. perstans*-infected individuals elicit IgG towards various glycan motifs present in *B. malayi* GSL glycans. Only a subset of the N-glycan structures printed on the array appeared to be antigenic and MFI levels were generally lower than observed for GSL glycans (**Figure 1**, **Figure S1**). To further identify the specific antigenic motifs targeted by IgG in the different filarial infection groups, we analyzed IgG binding in relation to the glycan structures printed on the array. Our previous work (21) revealed that N-glycans of *B. malayi* contained terminal N-acetylglucosamine (GlcNAc), terminal glucuronic acid (GlcA) residues and mannosidic glycans (Man_2-9_) as well as PC substituents (either GlcNAc or mannose-linked). GSL glycans contained terminal α-Gal, terminal N-acetylgalactosamine (GalNAc), terminal GlcNAc, terminal GlcA, fucosylated terminal α-Gal and fucosylated terminal N-acetylhexosamine (HexNAc, either GalNAc or GlcNAc). Fractions containing the listed glycan motifs were grouped into corresponding categories and MFI values of all fractions in a particular category were averaged for each individual.

**Figure 2:**
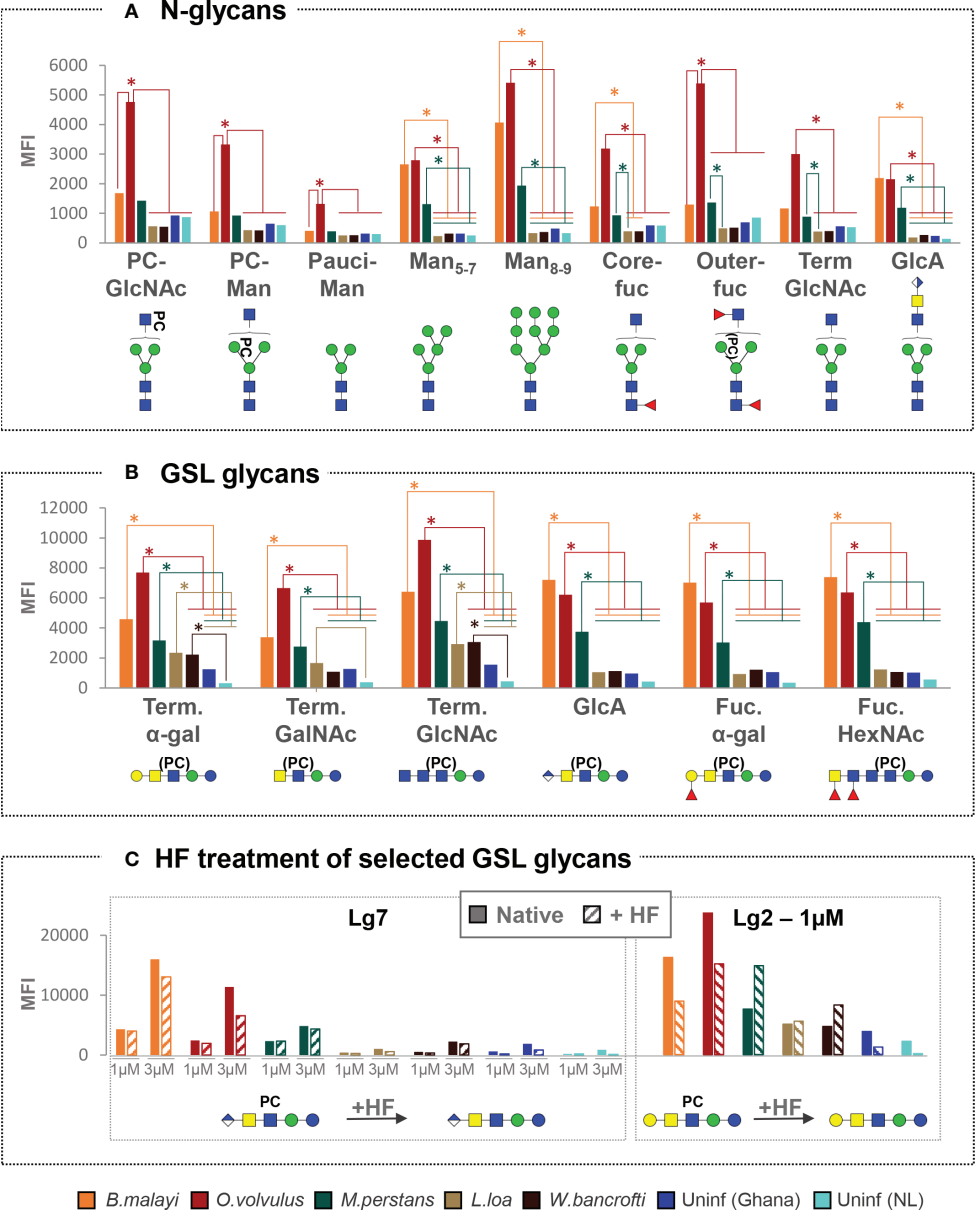
Cross-reactivity of IgG in various filarial infection and control plasmas to *B. malayi* N-linked and GSL glycan motifs. IgG binding to specific glycan motifs was determined for each individual by averaging MFI values of fractions belonging to the same category based on their glycan contents. Fraction groups are shown on the X-axis and named after the corresponding glycan motif present in the fractions: PC-GlcNAc = PC-substituted GlcNAc, PC-Man = PC-substituted mannose, Pauci-Man = Pauci-mannosidic N-glycans, Man_x-y_ = high-mannosidic N-glycans carrying 5 to 7 or 8 to 9 mannose residues, Core-fuc = α1-6 core fucosylated N-glycans, Out-fuc = α1-3 fucose attached to terminal GlcNAc, Term. GlcNAc = Terminal N-acetylglucosamine, GlcA = terminal GlcA, Term. α-Gal = terminal α1-4 Gal, Term. GalNAc = Terminal N-acetylgalactosamine, Fuc. α-Gal = Fucosylated (terminal) α-1-4 Gal, Fuc-HexNAc = Fucosylated HexNAc(s). A representative glycan structure for each category is shown below the X-axis using the Consortium for Functional Glycomics (CFG) nomenclature (see symbol key inset in **Figure 3**). MFI values obtained for each category were averaged for individuals from the same infection or control group: *B. malayi* (n = 10), *O. volvulus* (n = 6), *M. perstans* (n = 6), *L. loa* (n = 6), *W. bancrofti* (n = 6) infected individuals, uninfected (uninf.) Africans (Ghana, n = 6) and uninfected Europeans (The Netherlands, NL, n = 5). The upper graph shows results for N-glycan categories **(A)** and the middle graph for GSL glycan categories **(B)**. Statistical differences between groups were assessed using Bayesian statistics and obtained *p*-values can be found in **Table S3**. Significant differences (with threshold of significance α = 0.05) are represented on the graphs using asterisks and connecting lines. The lower graphs **(C)** compare the averaged MFI values for selected native or HF treated GSL fractions, *i.e.* with or without PC substituents as illustrated below the x-axis, where structures contained in the corresponding fractions are represented.

As shown in **Figure 2A** and **Table S3**, *B. malayi*-infected individuals showed significantly higher MFI values (*p*<0.05) to a limited subset of N-glycans, including high-mannosidic- (both Man5-7 and Man8-9) and GlcA-containing structures, than *L. loa*, *W. bancrofti* and both uninfected groups, for all of which no IgG binding to *B. malayi* derived N-glycans was apparent. Interestingly, while *M. perstans*-infected plasmas mimicked the reactivity seen in the *B. malayi*-infected ones, IgG binding in plasma from *O. volvulus* individuals yielded strikingly higher MFI values to all N-glycans assayed compared to the other infection groups, including the *B. malayi* group.

Notwithstanding these N-glycan associated observations, cross-reactivity of the IgG responses of *B. malayi*-, *O. volvulus*- and *M. perstans*-infected individuals was particularly apparent for the GSL fractions, with no significant differences between these three groups for all GSL glycan categories (**Figure 2B**, **Table S3**). For GlcA, terminal fucosylated α-Gal and fucosylated HexNAc-containing GSL glycans, all 3 infections gave significantly higher MFI values than *L. loa* and *W. bancrofti* groups which showed MFI levels comparable to the controls. This indicated that *B. malayi*, *O. volvulus* and *M. perstans* express these antigenic glycan motifs in the human host but that they may be absent in *L. loa* and *W. bancrofti* glycans. To the terminal α-Gal and terminal GlcNAc-containing GSL glycans however, significantly higher IgG reactivity was measured for all 5 infection groups, including *L. loa*- and *W. bancrofti*-infected individuals compared to the controls, indicating the widespread occurrence of these antigenic glycan motifs among the filarial nematodes (**Figure 2B**).

Since most of the *B. malayi* GSL glycans were substituted with PC, we treated certain fractions with HF to remove the PC substituent and evaluate its influence on IgG binding. As shown in **Figure 2C**, presence or absence of PC did not appear to strongly impact IgG binding to fraction Lg7 that contains a glycan of composition GlcA(β-)GalNAc(β1-4)[PC-6]GlcNAc(β1-3)Man(β1-4)Glc1-R across all the groups. This suggests that the IgG reactivity is directed to a motif generated by the terminal GlcA of the GSL backbone structure. For the HF treated version of fraction Lg2 (putative structure Gal(α1-4)GalNAc(β1-4)[PC-6]GlcNAc(β1-3)Man(β1-4)Glc1-R) a slight decrease in IgG binding was observed for the *B. malayi* and *O. volvulus* infection plasma, and an increase for the *M. perstans* and *W. bancrofti* groups. However, none of these differential reactivities were statistically significant (**Figure 2C**), confirming that the PC substituent was of only minor relevance for the specific IgG binding to this structure.

#### Structural study of O. volvulus N-linked and GSL glycans

**Figure 3:**
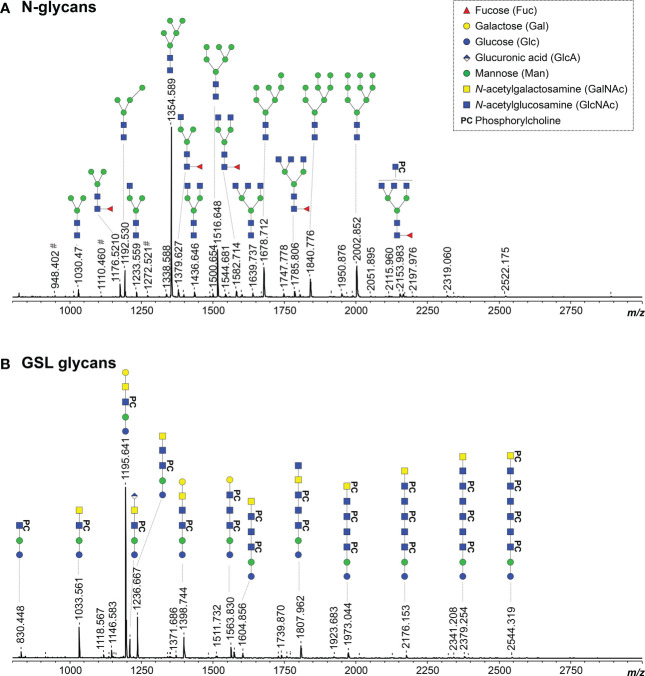
MALDI-TOF-MS of *O. volvulus* adult female worm N-linked and GSL glycans. *O. volvulus* glycoproteins were treated with PNGase F to release N-glycans **(A)** while EGCase I was used on glycolipids **(B)**. Enzymatically released glycans were then labelled with 2-AA and analyzed using MALDI-TOF-MS in negative-ion reflectron mode. Monoisotopic masses of measured signals are indicated and proposed glycan structures for ions with signal-to-noise ratios superior to 5 are depicted using the CFG nomenclature (see symbol key insert). # symbols signal m/z of glucose polymers from unknown origin. Compositions and structures were deduced using a panel of glycan sequencing techniques in combination with MALDI-TOF-MS/MS fragmentation, analogy with *B. malayi* and information from literature. See **Table S4** for MALDI-TOF-MS spectra raw data and complete structural assignment.

Since *O. volvulus* infection plasmas showed IgG binding to a large number of *B. malayi* glycans, we characterized the N-linked and GSL derived glycans from *O. volvulus* adult female worms using the glycomic workflow previously applied to *B. malayi* to determine whether the *O. volvulus* glycan repertoire would explain the observed cross-reactivity. Glycans were enzymatically released, fluorescently labeled with 2-AA and measured using MALDI-TOF-MS (**Figure 3**). MALDI-TOF-MS profiles of *O. volvulus* adult worms were strikingly similar to those obtained for *B. malayi* for both classes of glycoconjugates (21).

As for *B. malayi*, mannosidic N-glycans were present in abundance in *O. volvulus*, as confirmed using digestion with α (1–3, 6) Mannosidase (**Figure S2.A**). Comparative analysis by digestion with β-N-Acetylglucosaminidase S and β-N-Acetylhexosaminidase_f_ highlighted many structures extended with GlcNAc antennae and indicated the presence of β (1, 2)-linked GlcNAc residues (**Figures S2B, E**). The infrequent presence of LacdiNAc (GalNAcβ1-4GlcNAc) terminal units, that are resistant to the β-N-Acetylglucosaminidase but sensitive to the β-N-Acetylhexosaminidase_f_, was also observed (**Figure S2.B**). In addition, HF treatment revealed PC-substitutions in abundance and outer-arm fucoses, most likely α1-3 linked to GlcNAc, that were sensitive to the treatment, while many HF-resistant α1-6 core-fucoses were also observed (**Figures S2C, D**). Digestion with β-N-Acetylglucosaminidase S showed the presence of PC both on the antennae - substituting GlcNAc residues - as well as on the N-glycan core. The enzyme could digest a subset of structures up to a tri-mannosylated core of composition H3N2F1PC1 (*m/z* 1341.665 [M – H]^-^) containing both a core-fucose and a PC substituent (**Figure S2E**). Most likely, this substituent would be linked to one of the terminal mannose residues, as seen in *B. malayi*. Finally, a feature identified in *B. malayi* was the presence of GlcA-containing N-glycans. However, we did not detect any in *O. volvulus* adult parasites.

**Figure 4:**
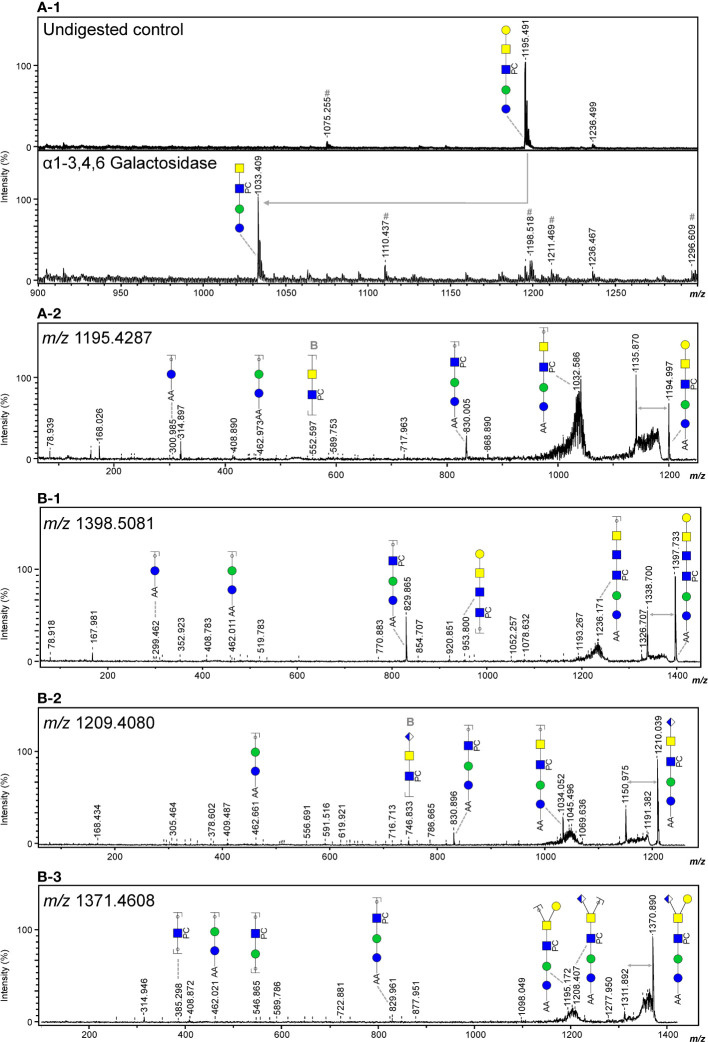
Evidence of terminal α-Gal and GlcA-containing epitopes in the GSL glycans of *O. volvulus*. MALDI-TOF-MS analysis of 2-AA-labeled *O. volvulus* major GSL glycan species subjected to digestion with α1-3,4,6 galactosidase **(A-1)** Digestion product showing the loss of a terminal α-Gal (Δ*m/z* = 162) is highlighted using a grey arrow. Negative ion reflectron mode MALDI-TOF-MS/MS of ions with theoretical *m/z* 1195.4287 **(A-2)**, m/z 1398.5081 (B-1), m/z 1209.4080 (B-2) and m/z 1371.4608 (B-3). Resulting spectra are labeled with graphic representation of Y-type ions, unless indicated otherwise (B = B-type). Loss of mass 59 Da from the parent ion is indicative of a PC loss (44, 45) and is highlighted by grey double arrows. For both MALDI-TOF-MS/MS and MALDI-TOF-MS spectra monoisotopic masses of 2-AA-labeled glycans are indicated and glycans are represented using the CFG nomenclature (see symbol key insert in **Figure 3**).

In line with literature (43), the major GSL glycan structure in *O. volvulus* was the zwitterionic glycan of composition H3N2PC1 (*m/z* 1195.641 [M – H]^-^). Using α1-3,6 Galactosidase and α1-3,4,6 Galactosidase, we determined the terminal hexose to be an α1-4 linked galactose (**Figures 4A–1**, **Figure S3A**), as for *B. malayi*. HF treatment proved the presence of PC-substitution in the structure backbone (**Figure S3B**) that can also be observed by MS/MS analysis of the ion with *m/z* 1195.4287 (**Figure 4A–2**). The HF-sensitive fucosylated version of this glycan (*m/z* 1341.569 [M – H]^-^) was also detected in *O. volvulus* (**Figure S3C**). Interestingly, we noted several structures of composition H3N3PC1-3, with an additional HexNAc and one to three PC substituent(s) (*m/z* 1398.744; 1563.830 and 1728.619 [M – H]^-^). MS/MS analysis of ion with *m/z* 1398.744 (**Figure 4B–1**) and resistance to digestion with both β-N-Acetylhexosaminidase_f_ and β-N-Acetylglucosaminidase S suggested the presence of the terminal α-Gal epitope in those structures as well (**Figures S3D, G**). The presence of hexuronic acid-containing GSL glycans was detected in *O. volvulus*, as shown by MS/MS analysis of the ion with *m/z* 1209.408 [M – H]^-^ (**Figure 4B–2**). As for *B. malayi*, this residue was found to occupy the terminal position on the glycan chain and by analogy, is expected to be β-linked GlcA. Interestingly, structures of compositions H3N2-3A1PC1-2 containing both α-Gal and GlcA residues, that were not detected in *B. malayi*, were found in *O. volvulus* (*m/z* 1371.686; 1574.798; 1739.596 [M – H]^-^). As shown by MS/MS analysis and sensitivity to HF treatment, these structures contained PC substituent(s) (**Figure 4B**, **Figures S3D, F**). Masses corresponding to structures of similar backbone compositions but with a fucose residue (H3N2-4A1F1) were also observed (*m/z* 1352.463 and 1758.622 [M – H]^-^) (**Table S4**). When analyzing the *m/z* 1371.461 [M – H]^-^ ion species using MS/MS, both a loss of a hexose and of a hexuronic acid were observed from the parent ion suggesting a possible branching of those residues at the terminal position (**Figure 4B–3**). Other terminal residues included GlcNAc and GalNAc which were discriminated by comparing sensitivity to digestions with β-N-Acetylglucosaminidase S and β-N-Acetylhexosaminidase_f_ (**Figures S3D, E, G, H**).

Despite some notable differences between species, overall, the structural analysis of the *O. volvulus* N-linked and GSL glycans confirmed the presence of many shared epitopes with *B. malayi*. A detailed list of the N-glycan and GSL glycan structures identified in *O. volvulus* is provided in **Table S4**. Thus, the observed cross-reactivity of IgG from *O. volvulus*-infected plasma to *B. malayi* glycans could be attributed to the overlapping similarity of glycan structures present in *O. volvulus*, and supports the conclusion that (partial) glycome overlaps with the other species also occur.

### IgG subclass responses to *B. malayi* glycan antigens in filarial nematode infections

In view of the particularly strong cross-reactivity observed between *B. malayi, O. volvulus* and *M. perstans* for total IgG and the general lack of data with respect to IgG subclasses and reactivity to helminth glycans, we next investigated the specific IgG subclass responses in these infection groups.

#### Study of plasma IgG subclasses in B. malayi-infected individuals

**Figure 5:**
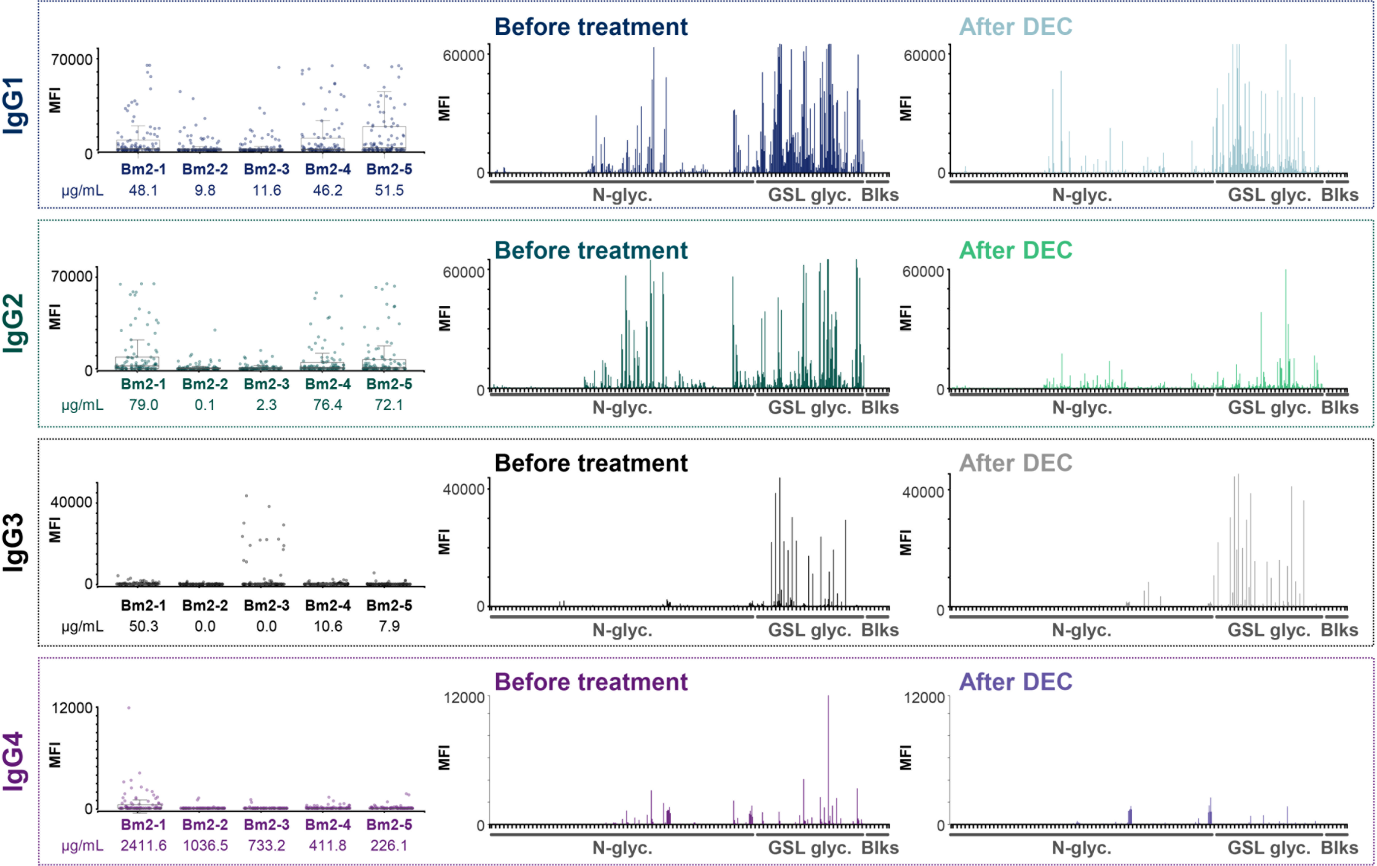
IgG subclass responses to *B. malayi* glycans in plasmas from infected individuals before and after DEC anthelmintic treatment. *B. malayi* glycan microarrays were screened for binding of IgG subclasses in plasma of *B. malayi*-infected individuals during infection and after treatment with DEC anthelminthic when individuals were all amicrofilaraemic. Subclass specific fluorescent secondary antibodies were used for detection of IgG subclass binding. Obtained background corrected MFI values are shown on the Y-axis of boxplots and graphs. Boxplots on the left show IgG subclass binding for all 5 individuals (ID numbers displayed on the X-axis, see **Table S1.A**) to each glycan fraction on the array (n = 100 with various printing concentrations and including buffer spots). BmA-specific IgG1-4 titers from the original study (14, 29) are depicted in µg/ml for each individual below their corresponding ID number underneath the boxplots. Graphs show overall IgG binding to the various glycan array fractions (X-axis, n = 100) for plasma from all 5 individuals either during infection (Before treatment, left) or after DEC treatment (After DEC, right).

Screening of the glycan microarrays with the *B. malayi* infection plasma yielded strong fluorescence signals for IgG1 and IgG2 subclasses (**Figure 5**). A similar pattern of glycan recognition to that seen for total IgG was observed, with a major reactivity toward the GSL glycans. Comparatively, lower MFI values were obtained for IgG3 and very low signals were observed for IgG4 (**Figure 5**, **Table S5**, **Figure S4**) indicating that IgG to *B. malayi* antigenic N-linked and GSL glycans is mainly of the IgG1 and IgG2 subclasses.

During previous studies (15, 29), IgG subclasses to *B. malayi* crude antigen (BmA) were measured by ELISA. Those titers of IgG1-4 to BmA (in µg/mL) are indicated in **Figure 5** below the boxplots showing IgG subclass binding to the *B. malayi* glycan fractions for each individual. When comparing the ELISA data to the glycan microarray results, we noticed a clear correlation between the array MFI values and the BmA-specific IgG titers, with individuals having higher titers of IgG1-4 to BmA (in µg/mL) also showing higher binding to the glycan microarray.

We previously reported that total IgG response to *B. malayi* glycan antigens dropped markedly after DEC treatment (21). Focusing on IgG subclass responses in the same sample set of plasma (n = 5, second set, see Materials and Methods section), a decrease in MFI levels on the glycan microarray after DEC treatment was clearly visible for IgG2 as well as for IgG1 and IgG4 – although less pronounced – but no changes were apparent for IgG3 (**Figure 5**, **Figure S4**). Using Bayesian statistics for paired samples (a moderated paired *t*-test allowing for sib-pair effects in the linear model), we determined that differences pre- and post-treatment were only significant for 4 fractions for IgG1 binding while IgG2 binding to 19 fractions was significantly decreased post-DEC treatment. Differences in binding to specific glycan fractions of IgG3 and IgG4 between the two time-points were not significant (**Table S5**). MFI values for IgG1 towards parasite glycans decreased for 4 of the 5 individuals tested, whereas IgG2 MFI values were clearly lower for all of them (**Figure S4**). In an earlier study, a decrease of IgG1 after DEC treatment was observed also to crude BmA, however with a substantial individual-to-individual variability (29). The consistent and clear reduction of IgG2 to *B. malayi* glycans post-treatment was however not reflected by IgG2 to BmA (29), indicating that specific *B. malayi* glycans form an antigen subset with differential characteristics.

#### Cross-comparison of plasma IgG subclass responses from filaria-infected individuals and uninfected controls

**Figure 6:**
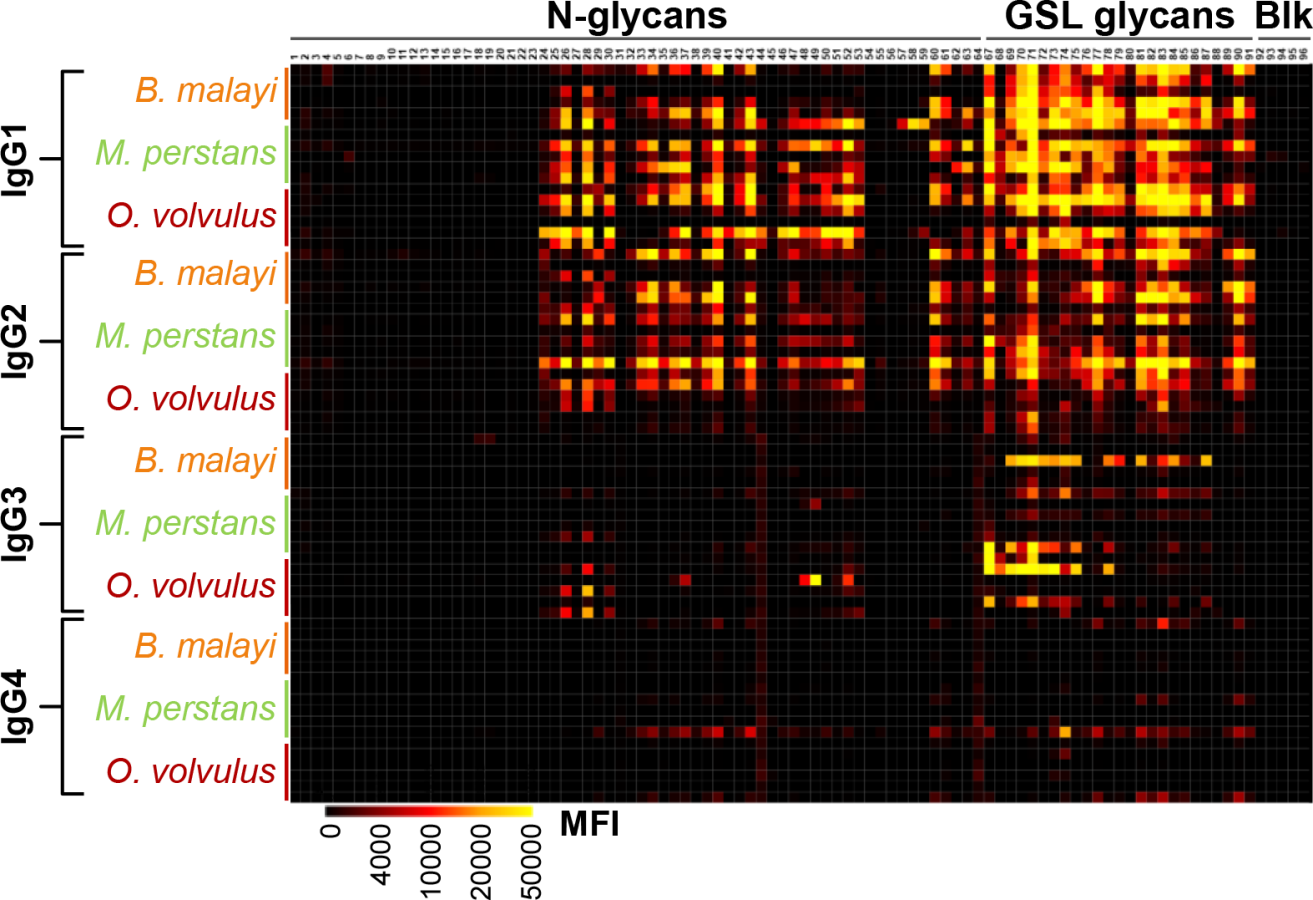
IgG subclass responses in plasma from *B. malayi-*, *O. volvulus-* and *M. perstans-* infected individuals to *B. malayi* glycans. (*B*) *malayi* glycan microarrays were screened for binding of IgG subclasses with plasma of *B. malayi*- (n = 5), *O. volvulus-* (n = 6) and *M. perstans-* (n = 6) infected individuals. Subclass specific fluorescent secondary antibodies were used for detection of IgG subclass binding. Heatmap was generated from obtained background corrected MFIs (see intensity key at the bottom) and shows binding of all IgG subclasses for each infected individual as indicated on the Y-axis. X-axis displays the various glycan fractions printed on the array as detailed in (21). Type of fraction content (N-glycan and GSL glycan) or control (Blk) is indicated above the fraction IDs.

Next, we examined *B. malayi* glycan-reactive IgG subclasses in the highly cross-reactive *O. volvulus* and *M. perstans* infection plasmas. As for *B. malayi*, highest MFI signals were obtained for IgG1 and IgG2 while IgG3 and IgG4 binding to the array yielded lower MFI values (**Figure 6**, **Figures S5-6**). Statistical analysis confirmed the similarity of the IgG subclass responses to the glycans between the three infections (**Table S5**). In each case, MFI values were significantly higher than in uninfected individuals to a large proportion of the glycans on the array. This was particularly clear for IgG1 that was detected to virtually all GSL glycans and to many N-glycans in *O. volvulus* and *M. perstans* infection plasmas. Similar observations were made for IgG2, however with the *O. volvulus*-infected group showing lower MFI values for IgG2 than for IgG1, yielding less significant differences with the control plasmas than the *B. malayi*- and *M. perstans*-infected groups (**Table S5**).

**Figure 7:**
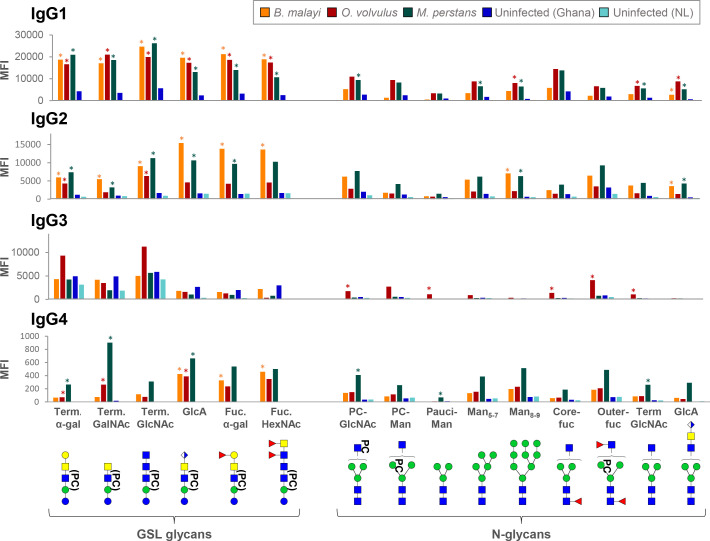
Binding of IgG subclasses from *B. malayi*, *O. volvulus* and *M. perstans* infection and control plasmas to *B. malayi* N-linked and GSL glycan motifs. IgG subclass binding to specific glycan motifs was calculated for each individual by averaging MFIs of fractions belonging to the same category based on their glycan content. Fraction groups are shown on the X-axis and named after the corresponding glycan motif present in the fractions: PC-GlcNAc = PC-substituted GlcNAc, PC-Man = PC-substituted mannose, Pauci-Man = Pauci-mannosidic N-glycans, Man x-y = High-mannosidic N-glycans carrying 5 to 7 or 8 to 9 mannose residues, Core-fuc = α1-6 core fucosylated N-glycans, Out-fuc = α1-3 fucose attached to terminal GlcNAc, Term. GlcNAc = Terminal N-acetylglucosamine, GlcA = terminal GlcA, Term. α-Gal = terminal α1-4 Gal, Term. GalNAc = Terminal N-acetylgalactosamine, Fuc. α-Gal = Fucosylated (terminal) α-1-4 Gal, Fuc-HexNAc = Fucosylated HexNAc(s). A representative glycan structure for each category is shown below the X-axis using the CFG nomenclature (see symbol key inset in **Figure 3**). Category MFIs thus obtained were averaged for individuals from the same infection or control group: *B. malayi*- (n = 5) infected individuals (before DEC treatment), *O. volvulus-* (n = 6) infected individuals, *M. perstans-* (n = 6) infected individuals, uninfected (uninf.) Africans (Ghana, n = 6) and uninfected Europeans (The Netherlands, NL, n = 5). Significant differences between groups were assessed using Bayesian statistics. MFIs significantly higher for infection plasma IgG than for both uninfected groups are indicated with asterisks. Obtained *p*-values can be found in **Table S6**.

MFI values resulting from IgG3 binding to the microarray were lower than the ones observed for IgG1 and IgG2 for all three infection groups but were still significantly higher than the uninfected groups for several GSL glycan fractions. IgG3 to N-glycans however, was very limited in *B. malayi* and *M. perstans* infection plasmas but was detected in *O. volvulus* infection plasmas. Study of IgG subclasses to specific glycan motifs (**Figure 7**) reflected this observation, with *O. volvulus* infection plasma showing significantly higher MFI values than uninfected individuals and *B. malayi*-infected individuals for several N-glycan categories (**Table S6**). Interestingly, 2 individuals in each uninfected group showed relatively high levels of plasma IgG3 to GSL glycans (**Figure S7**). This resulted in the absence of significant differences between infected and uninfected individuals when averaging MFI for the various GSL glycan categories, although some statistical differences were observed for IgG3 binding to individual fractions (**Table S5**).

Although markedly weaker, IgG4 was detected to a few fractions. When compared with uninfected groups, IgG4 to 8 and 11 glycan fractions was significantly higher for the *B. malayi* and *M. perstans* groups, respectively, but only 2 fractions gave significantly higher MFI values for the *O. volvulus* group (**Table S5**). Thus, mirroring observations made for *B. malayi* infections, glycans appear not to be the primary targets of IgG4 in *M. perstans* and *O. volvulus* infections either, despite elevated plasma IgG4 titers (46). Nonetheless, MFI values averaged to assess IgG4 to specific glycan motifs were still significantly higher for the infected groups than the ones measured for the uninfected groups for certain glycan categories (**Figure 7**; **Table S6**).

Overall, the analysis focusing on IgG subclasses to specific glycan motifs (**Figure 7**) confirmed and summarized the similarities observed between the three infections, with few statistical differences between the three groups (**Table S6**). IgG1 was comparable for all, with significantly higher MFI values obtained for each GSL glycan category when compared to uninfected controls. Similarly, the IgG2 response in *B. malayi*- and *M. perstans*-infected individuals was significantly higher to all GSL glycans than in the uninfected groups. However, as mentioned above, IgG2 binding from the *O. volvulus*-infected individuals yielded lower MFI values that were only significantly higher than the uninfected controls for the α-Gal and terminal GlcNAc-containing GSL subsets (**Figure 7**).

## Discussion

We have shown previously that the filarial nematode *B. malayi* expresses a variety of N-glycans and highly antigenic GSL glycans, to which individuals infected with the parasite develop IgG (21). Here, we showed that individuals infected with *O. volvulus* and *M. perstans* also produce IgG to antigenic motifs contained in a broad range of *B. malayi* glycans, while *W. bancrofti*- and *L. loa*-infected individuals appeared to produce IgG reactive with only a restricted subset of the *B. malayi* GSL-derived glycans. This suggests that *B. malayi*, *O. volvulus* and *M. perstans* express highly similar N-linked and GSL glycans that are not present in *L. loa* and *W. bancrofti*.

Structural characterization of the N-linked and GSL glycans of *O. volvulus* adult worms supported this hypothesis. Many (antigenic) glycan elements reported for *B. malayi* were identified in *O. volvulus*, including terminal α-Gal and GlcA in the GSL glycans (**Figure 4**). We also noted the presence of an epitope containing both α-Gal and GlcA in several GSL glycans of *O. volvulus* that was not detected in *B. malayi* (**Figure 4B**). The glycan microarray used in this study contained exclusively glycans isolated from *B. malayi*, but it would be of interest to study antibody binding to this combined α-Gal and GlcA motif, which may perhaps represent an *O. volvulus* specific glycan epitope. N-glycans from both species were very similar, with the presence of mannosidic structures and structures extended with GlcNAc antennae that were fucosylated and/or substituted with PC. These findings were overall in line with previous work conducted on the N-glycans of *O. volvulus (* 47). There too, structures of composition H3N[3-6]F[0-1]PC1 were detected, as well as the presence of both GlcNAc and, to a lesser extent, GalNAc residues within the antennae. We did not detect GlcA as part of *O. volvulus* N-glycans, while we clearly observed IgG to the *B. malayi*-derived GlcA containing N-glycans on the microarray in *O. volvulus* infection plasmas. This suggests that the GlcA-containing epitope present in the *O. volvulus* GSL glycans - and possibly in other classes of glyconjugates not studied here - induce the IgG that also binds to an identical or similar GlcA-containing epitope in the context of N-glycans. Specifically, we have found the GlcA residue to be present as a terminal residue on the LacdiNAc (*i.e.* GalNAcβ1-4GlcNAc) motif in the *B. malayi* N-linked and GSL glycans (21).

In that regard, glycan microarray screening is highly informative in indicating exposure to, and therefore presence of, a specific glycan motif during parasitic or bacterial infections. This is particularly useful when structural glycomic studies of a specific pathogen have not been conducted yet or are not possible due to unavailability of the source materials. Here, performing a comprehensive glycomic study and native glycan release from two species, *B. malayi* and *O. volvulus*, indirectly allowed us to gather information on the glycomes of three other related nematodes. We indeed detected the presence of IgG to GSL glycans containing terminal α-Gal and stretches of GlcNAc residues in the plasma of all infection groups (**Figure 2**) allowing us to infer the expression of these types of structures by all species of nematodes, although they might be present in diverse glycoconjugate classes. The presence of IgG to terminal α-Gal, in particular, is consistent with indications of the zwitterionic glycolipid of composition Gal(α1-4)GalNAc(β1-4)[PC-6]GlcNAc(β1-3)Man(β1-4)Glc (1–1)ceramide being highly conserved in the nematode phylum (43). On the other hand, of the species examined, it is likely that only *B. malayi*, *O. volvulus* and *M. perstans* express other motifs, based on terminal GlcA or fucosylated α-Gal, since IgG to these types of glycans were only measured in plasma from these infections. We cannot completely exclude that *L. loa* and *W. bancrofti* also harbor these glycan epitopes but, if so, they appear to either express them in a much lower amount or in a less antigenic context or in non-mammalian life stages. In that regard, it is also interesting to note the high IgG MFI levels observed with *O. volvulus* infection plasma to virtually all *B. malayi* N-glycans while *B. malayi*-infected individuals only showed high MFI levels to a restricted subset of N-glycans (**Figure 2**). Perhaps higher titers of IgG to N-glycans are elicited in onchocerciasis either as a consequence of more abundant excretion/secretion of antigenic N-glycosylated proteins or of a more efficient and immunogenic presentation on different protein carriers.

Comparative glycogenomic studies are useful alternatives when structural glycomic studies are not possible or required. It would be complementary to our work to study whether certain glycosyltransferase genes and activities are absent from *L. loa* and *W. bancrofti* compared to the other filarial species. However, despite recent improvements for several species of filarial nematodes (48), the genome quality of the five species of filarial nematodes included in this study is uneven. In addition, the insufficiency of functional genomic annotations and the absence of well-characterized glycosylation pathways for non-mammalian organisms, particularly where they diverge from human or mouse pathways, make this type of study challenging.

A well-known shared feature of filarial nematodes is the presence of PC substituents, most of the time present in a phosphodiester linkage with C6 of the GlcNAc residues of N-linked and GSL glycans (42). Immunomodulatory properties of PC-containing epitopes have been extensively studied over the years (49, 50), particularly in relation to the ES-62 glycoprotein of *Acanthocheilonema viteae (* 51, 52). Notably, PC-containing glycoproteins are thought to contribute to low antibody levels and poor lymphocyte responses observed in some filariasis patients (53). In our study, HF treatment of selected GSL containing fractions showed that highly antigenic elements were still present after removal of PC and that these are major IgG targets. Thus, the contribution of PC substituents to IgG binding to glycans was not dominant. This is consistent with previous observations in both brugian (21) and bancroftian filariasis (19) and it appeared that PC substituents do not elicit specific IgG in the five filarial infections studied here, although they might have influenced the overall antibody levels. The presence of PC is known to generate “background” cross-reactivity of antibodies to parasite antigens, both in filarial nematode infections but also with non-endemic serum since PC is present in many bacteria and fungi (46, 54). In our study, averaged MFI values obtained for the control groups to PC-containing N-glycans (both to PC-substituted GlcNAc and PC-substituted mannose categories) indeed tend to be higher than for the other N-glycan categories (**Figure 2**). IgG binding to PC-substituted glycans from individuals not infected with filarial nematodes, is likely to be a consequence of exposure to PC from various other pathogens (55). In the past, cross-reactivity attributed to PC and other determinants has been deemed responsible for hampering the development of serological diagnosis for filariasis (46). However, in our work, the background possibly caused by these shared epitopes was negligible when compared to the strong signals generated by specific IgG recognizing other, potentially more specific and antigenic glycans.

Another consequence of glycan cross-reactivity issues, has been the increased interest towards the IgG4 subclass for serological diagnostic applications since IgG4 was known to recognize restricted subsets of antigens (46) of non-glycan nature (56) compared to the other IgG subclasses. Despite the unusually high IgG4 titers to filarial antigens typical of the immune response in LF (15, 25), we observed very little or no IgG4 binding to glycans in infected plasmas (**Figure 5**), indicating that IgG4 response in LF would mainly target non-carbohydrate epitopes in BmA, presumably protein components. This is consistent with previous work where humans were found to be “genetically restricted” from making IgG4 to PC, polysaccharides and streptococcal carbohydrate antigens (56, 57), although N-glycans and GSL-glycans may have different molecular properties with respect to generating an immune response in the host. The low-intensity IgG4 signals measured in our study were highly variable between individuals (**Figure S4**) but were still significantly higher in *B. malayi*-infected individuals than in uninfected controls to a few glycans such as GlcA-containing fractions (**Table S5**). In *Schistosoma mansoni*-infected individuals (35) IgG4 binding has also been found to be very restricted to specific glycans, in that case, those containing α1-3 core-fucoses and core-xylose. There too, variability between individuals was substantial, which, in our study, is reflected in the low significance levels observed between the different groups (**Figure 7**; **Table S6**).

In contrast to IgG4, a strong IgG1 and IgG2 binding to our glycan microarrays was measured in the filarial infection plasmas when compared to uninfected controls (**Figure 6**). IgG2 is known to play a role in responses to carbohydrate antigens in the context of bacterial and parasitic infections (19, 58), although it is now clear that anti-glycan responses are not restricted to this subclass (35, 59). Plasma IgG subclasses differ highly from each other in terms of antigen specificity and functional properties (60). Their distribution also varies as a function of age with the IgG1 and IgG3 isotype levels reaching adult levels in sera sooner than IgG2 and IgG4 subclasses (61, 62), which is an important consideration to take into account for diagnosis of children. Nonetheless, in other respects, IgG2 to glycan antigens in plasmas from *B. malayi*-infected individuals, and particularly to GSL glycans, showed promising features for potential diagnostic applications. High MFI values were observed in infected plasma, that were significantly decreased in the plasma sampled post-DEC treatment, less than 2 years later. Moreover, IgG2 to the *B. malayi* glycans was also detected in the plasma of *M. perstans*-infected individuals but to a lesser extent in the plasma of *O. volvulus*-infected individuals (**Figure 7**), suggesting that IgG2 might allow a gain in species-specificity compared to the other subclasses. Additionally, cross-reactivity between these filarial nematodes might not be a major concern since *B. malayi* is not co-endemic with *O. volvulus (* 4) or *M. perstans (* 63).

An important consideration in LF is the association of specific IgG subclasses with the various subpopulations in endemic areas where asymptomatic microfilariaemic carriers have higher ratios of IgG4 to IgE while individuals with chronic disease have elevated IgG1-3 (15). Further research should aim to address how IgG subclasses to filarial glycan antigens are distributed within the endemic subpopulations, since it is a crucial point to evaluate for considering a further diagnostic potential of our findings. Moreover, it would be of great interest to determine if symptomatic or putatively protected individuals exhibit differences in anti-glycan antibodies, which could relate to protection or be involved in pathology. Another aspect to consider, is whether the presence of circulating microfilariae is necessary for development of anti-glycan IgG. In our previous study, we demonstrated comparable profiles for the N-glycans and GSL glycans from *B. malayi* human stages - infective third-stage larvae, adult worms (males and females) and microfilariae (21). Thus, it is not known from which parasite life-stage(s) the host immune system gets exposed to glycan antigens. In *B. malayi*-infected rhesus macaques, IgG to glycan epitopes is apparent five weeks post-infection, prior to microfilarial production (21). Whether the same happens in humans and whether the mere presence of adult worms, as occurs in amicrofilaraemic infections, is enough to drive and maintain the anti-glycan antibody response was not addressed in this study, since all individuals were microfilaraemic (**Table S1**).

In conclusion, research on anti-glycan antibodies in the context of parasitic infections is still limited, particularly at the subclass level. With this study, we investigated the plasma IgG response to *B. malayi* glycans from individuals infected with filarial nematodes. We addressed cross-reactivity and subclass specificity of the anti-glycan IgG responses for five major filarial nematode infections. Many crucial aspects should be further investigated on the basis of this work, such as possible differences in IgG responses between different subpopulations in endemic areas, *i.e.* chronic and asymptomatic infections. Also, it will be important to validate our observations on a larger cohort, including individuals from diverse geographic areas. Nonetheless, our data indicates that IgG responses to glycan antigens in filariasis might offer unexplored and valuable alternatives for use in diagnostic applications.

## Data availability statement

The datasets presented in this study can be found in online repositories. The names of the repository/repositories and accession number(s) can be found below: GPST000299 (https://glycopost.glycosmos.org/), and in Github (https://github.com/lmcpetralia/Front-Immunol-doi-10.3389-fimmu.2023.1102344.git/).

## Ethics statement

The studies involving human participants were reviewed and approved by the Indonesian Department of Health and Human Services and the National Ethics Committee for Human Health Research. The patients/participants provided their written informed consent to participate in this study.

## Author contributions

LP: Conceptualization, Investigation, Formal analysis, Writing – Original draft, Reviewing & Editing, Visualization; AvD: Conceptualization, Writing – Reviewing & Editing, Supervision; DLN and LL: Investigation; ES; SB; TN; KP; AH; and SW: Resources; JF and CH: Conceptualization, Writing – Reviewing & Editing, Supervision, Funding acquisition. All authors contributed to the article and approved the submitted version.

## References

[1] Otsuji Y. History, epidemiology and control of filariasis. Trop Med Health (2011) 39(1):3–13. 10.2149/tmh.39-1-suppl_2-3 PMC3153148 22028595

[2] Pearson RD. MSD manual - FILARIAL WORM INFECTIONS OVERVIEW . Available at: https://www.msdmanuals.com/home/infections/parasitic-infections-nematodes-roundworms/filarial-worm-infections-overview.

[3] Deshpande A Miller-Petrie MK Johnson KB Abdoli A Abrigo MRM Adekanmbi V. The global distribution of lymphatic filariasis, 2000–18: a geospatial analysis. Lancet Glob Heal (2020) 8(9):e1186–94. 10.1016/S2214-109X(20)30286-2 PMC7443698 32827480

[4] TEAM WHO. Ending the neglect to attain the sustainable development goals: a road map for neglected tropical diseases 2021–2030. Diseases DMMN/ N tropical, editor. Word Health Organization (2020). 196 p. Available at: https://www.who.int/publications/i/item/9789240010352.

[5] Gyasi ME Okonkwo ON Tripathy K. Onchocerciasis. StatPearls, editor. StatPearls Publishing (2022). Available at: https://pubmed.ncbi.nlm.nih.gov/32644453/. Available at: https://www.ncbi.nlm.nih.gov/books/NBK559027/. 32644453

[6] King CL Weil GJ Kazura JW. Single-dose triple-drug therapy for wuchereria bancrofti — 5-year follow-up. N Engl J Med (2020) 382(20):1956–7. 10.1056/NEJMc1914262 PMC7175637 32402169

[7] Taylor MJ Hoerauf A Bockarie M. Lymphatic filariasis and onchocerciasis. Lancet. (2010) 376(9747):1175–85. 10.1016/S0140-6736(10)60586-7 20739055

[8] Centers for Disease Control and Prevention. Lymphatic filariasis - diagnosis (2018). Available at: https://www.cdc.gov/parasites/lymphaticfilariasis/diagnosis.html.

[9] Centers for Disease Control and Prevention. Onchocerciasis - diagnosis (2019). Available at: https://www.cdc.gov/parasites/onchocerciasis/diagnosis.html.

[10] Lourens GB Ferrell DK. Lymphatic filariasis. Nurs Clin North Am (2019) 54(2):181–92. 10.1016/j.cnur.2019.02.007 31027660

[11] Wanji S Esum ME Njouendou AJ Mbeng A Ndongmo PWC Abong RA. Mapping of lymphatic filariasis in loiasis areas : A new strategy shows no evidence for wuchereria bancrofti endemicity in Cameroon. PloS Negl Trop Dis (2019) 13(3):1–15. 10.1371/journal.pntd.0007192 PMC6436748 30849120

[12] Lakwo T Oguttu D Ukety T Post R. Onchocerciasis Elimination : Progress and challenges. Res Rep Trop Med (2020) 11:81–95. 33117052 10.2147/RRTM.S224364 PMC7548320

[13] Damgaard J Meyrowitsch DW Rwegoshora RT Magesa SM Mukoko DA Simonsen PE. Assessing drivers of the IgG4 antibody reactivity to recombinant antigen Bm14 in wuchereria bancrofti endemic populations in East Africa. Acta Trop (2016) 161:26–32. doi: 10.1016/j.actatropica.2016.05.003 27172877

[14] Weill GJ Kastens W Susapu M Laney SJ Williams SA King CL. The impact of repeated rounds of mass drug-administration with diethylcarbamazine plus albendazole on bancroftian filariasis in Papua new Guinea. PloS Negl Trop Dis (2008) 2(12):1–7. 10.1371/journal.pntd.0000344 PMC2586652 19065257

[15] Kurniawan A Yazdanbakhsh M van Ree R Aalberse R Selkirk ME Partono F. Differential expression of IgE and IgG4 specific antibody responses in asymptomatic and chronic human filariasis. J Immunol (1993) 150(9):3941–50. 8473742

[16] Hotterbeekx A Perneel J Vieri MK Colebunders R Kumar-Singh S. The secretome of filarial nematodes and its role in host-parasite interactions and pathogenicity in onchocerciasis-associated epilepsy. Front Cell Infect Microbiol (2021) 11(April):1–10. 10.3389/fcimb.2021.662766 PMC8113626 33996633

[17] Buck AH Coakley G Simbari F McSorley HJ Quintana JF Le Bihan T. Exosomes secreted by nematode parasites transfer small RNAs to mammalian cells and modulate innate immunity. Nat Commun (2014) 5:1–11. 10.1038/ncomms6488 PMC4263141 25421927

[18] Satapathy AK Bal MS Das MK. Differential antibody response to parasite lipid antigens in lymphatic filariasis. Curr Sci Assoc [Internet]. (2000) 78(11):1371–5.

[19] Mohanty MC Satapathy AK Sahoo PK Ravindran B. Human bancroftian filariasis - a role for antibodies to parasite carbohydrates. Clin Exp Immunol (2001) 124(1):54–61. 11359442 10.1046/j.1365-2249.2001.01484.x PMC1906036

[20] Hertz MI Rush A Nutman TB Weil GJ Bennuru S Budge PJ. Characterization of glycan determinants that mediate recognition of the major wuchereria bancrofti circulating antigen by diagnostic antibodies. Mol Biochem Parasitol (2020) 240(20):111317. doi: 10.1016/j.molbiopara.2020.111317 32961208 PMC11006022

[21] Petralia LMC van Diepen A Lokker LA Nguyen DL Sartono E Khatri V. Mass spectrometric and glycan microarray-based characterization of the filarial nematode brugia malayi glycome reveals anionic and zwitterionic glycan antigens. Mol Cell Proteomics (2022) 21(5):1–22. doi: 10.1016/j.mcpro.2022.100201 PMC9046957 35065273

[22] Hokke CH van Diepen A. Helminth glycomics – glycan repertoires and host-parasite interactions. Mol Biochem Parasitol (2017) 215:47–57. doi: 10.1016/j.molbiopara.2016.12.001 27939587

[23] Ottesen EA Skvaril F Tripathy SP Poindexter RW Hussain R. Prominence of IgG4 in the IgG antibody response to human filariasis. J Immunol (1985) 134(4):2707–12. 2579154

[24] Dafa’alla TH Ghalib HW Abdelmageed A Williams JF. The profile of IgG and IgG subclasses of onchocerciasis patients. Clin Exp Immunol (1992) 88(2):258–63. 10.1111/j.1365-2249.1992.tb03070.x PMC1554306 1572089

[25] Babu S Nutman TB. Immunology of lymphatic filariasis. Parasite Immunol (2014) 36(1):338–46. 10.1111/pim.12081 PMC3990654 24134686

[26] Sartono E Kruize Y Kurniawan A Maizels R Yazdanbakhsh M. Depression of antigen-specific interleukin-5 and interferon-gamma responses in human lymphatic filariasis as a function of clinical status and age. J Infect Dis (1996) 175(5):1276–80. 10.1086/593701 9129104

[27] Sartono E Kruize YCM Kurniawan A Maizels RM Yazdanbakhsh M. In Th2-biased lymphatic filarial patients, responses to purified protein derivative of mycobacterium tuberculosis remain Th1. Eur J Immunol (1996) 26(2):501–4. 10.1002/eji.1830260233 8617323

[28] Sartono E Lopriore C Kruize YCM Kurniawan-Atmadja A Maizels RM Yazdanbakhsh M. Reversal in microfilarial density and T cell responses in human lymphatic filariasis. Parasite Immunol (1999) 21(11):565–71. 10.1046/j.1365-3024.1999.00253.x 10583857

[29] Atmadja AK Atkinson R Sartono E Partono F Yazdanbakhsh M Maizels RM. Differential decline in filaria-specific IgG1 , IgG4 , and IgE antibodies in brugia malayi-infected patients after diethylcarbamazine chemotherapy. J Infect Dis (1995) 172(6):1567–72. 10.1093/infdis/172.6.1567 7594718

[30] Ritter M Ndongmo WPC Njouendou AJ Nghochuzie NN Nchang LC Tayong DB. Mansonella perstans microfilaremic individuals are characterized by enhanced type 2 helper T and regulatory T and b cell subsets and dampened systemic innate and adaptive immune responses. PloS Negl Trop Dis (2018) 12(1):1–21. 10.1371/journal.pntd.0006184 PMC5783424 29324739

[31] Arndts K Specht S Debrah AY Tamarozzi F Klarmann Schulz U Mand S. Immunoepidemiological profiling of onchocerciasis patients reveals associations with microfilaria loads and ivermectin intake on both individual and community levels. PloS Negl Trop Dis (2014) 8(2):1–14. 10.1371/journal.pntd.0002679 PMC3930501 24587458

[32] van Diepen A van der Plas AJ Kozak RP Royle L Dunne DW Hokke CH. Development of a schistosoma mansoni shotgun O-glycan microarray and application to the discovery of new antigenic schistosome glycan motifs. Int J Parasitol [Internet]. (2015) 45(7):465–75. doi: 10.1016/j.ijpara.2015.02.008 25819714

[33] van Diepen A Smit CH van Egmond L Kabatereine NB Pinot de Moira A Dunne DW. Differential anti-glycan antibody responses in schistosoma mansoni-infected children and adults studied by shotgun glycan microarray. PloS Negl Trop Dis (2012) 6(11):1–10. 10.1371/journal.pntd.0001922 PMC3510071 23209862

[34] Yang YYM Li XH Brzezicka K Reichardt NC Wilson RA van Diepen A. Specific anti-glycan antibodies are sustained during and after parasite clearance in schistosoma japonicum-infected rhesus macaques. PloS Negl Trop Dis (2017) 11(2):1–21. 10.1371/journal.pntd.0005339 PMC5308859 28151933

[35] Yang YYM Van DA Brzezicka K Reichardt N Hokke CH. Glycan microarray-assisted identification of IgG subclass targets in schistosomiasis. Front Immunol (2018) 9(October):1–8. 30356796 10.3389/fimmu.2018.02331 PMC6190862

[36] Oyindasola O McShaneb LM Doddb L Gildersleeve JC. Profiling human serum antibodies with a carbohydrate antigen microarray. J Proteome Res (2009) 8(9):4301–10. 10.1021/pr900515y PMC2738755 19624168

[37] Mehta AY Cummings RD. Data and text mining GLAD : GLycan array dashboard, a visual analytics tool for glycan microarrays. Bioinformatics. (2019) 35(18):3536–7. 10.1093/bioinformatics/btz075 PMC6748710 30715201

[38] Ritchie ME Phipson B Wu D Hu Y Law CW Shi W. Limma powers differential expression analyses for RNA-sequencing and microarray studies. Nucleic Acids Res (2015) 43(7):e47. 25605792 10.1093/nar/gkv007 PMC4402510

[39] Phipson B Lee S Majewski IJ Alexander WS Smyth G. Robust hyperparameter estimation protects. Ann Appl Stat (2016) 10(2):946–63. 10.1214/16-AOAS920 PMC5373812 28367255

[40] Vainauskas S Kirk CH Petralia L Ellen PG Mcleod E Bielik A. A novel broad specificity fucosidase capable of core α 1-6 fucose release from n- glycans labeled with urea- linked fluorescent dyes. Sci Rep (2018) 8(9504):1–8. 29934601 10.1038/s41598-018-27797-0 PMC6015026

[41] Ceroni A Maass K Geyer H Geyer R Dell A Haslam SM. GlycoWorkbench: A tool for the computer-assisted annotation of mass spectra of glycans. J Proteome Res (2008) 7(4):1650–9. 10.1021/pr7008252 18311910

[42] Buitrago G Duncombe-Moore J Harnett MM Harnett W. Mini review: Structure and function of nematode phosphorylcholine-containing glycoconjugates. Front Trop Dis (2021) 2(December):1–11.

[43] Wuhrer M Rickhoff S Dennis RD Soboslay PT Baumeister S Geyer R. Phosphocholine-containing, zwitterionic glycosphingolipids of adult onchocerca volvulus as highly conserved antigenic strucutres of parastic nematodes. Biohchem J (2000) 348:417–23. PMC1221081 10816437

[44] Ho Y Huang P. A novel structural analysis of glycerophosphocholines as TFA/K+ adducts by electrospray ionization ion trap tandem mass spectrometry. Rapid Commun Mass Spectrom [Internet]. (2002) 16(16):1582–9. doi: 10.1002/rcm.751 12203251

[45] Murphy RC Axelsen PH. Mass spectrometric analysis of long-chain lipids. Mass Spectrom Rev (2011) 30(4):579–99. 10.1002/mas.20284 PMC3117083 21656842

[46] Weil GJ Ogunrinade AF Chandrashekar R Kale OO. IgG4 subclass antibody serology for onchocerciasis. J Infect Dis (1990) 161(3):549–54. 10.1093/infdis/161.3.549 2179425

[47] Haslam SM Houston KM Harnett W Reason AJ Morris HR Dell A. Structural studies of n -glycans of filarial parasites conservation of phosphorylcholine-substituted glycans among species and discovery of novel chito-oligomers. J Biol Chem (1999) 274(30):20953–60. 10.1074/jbc.274.30.20953 10409642

[48] Bennuru S O’Connell EM Drame PM Nutman TB. Mining filarial genomes for diagnostic and therapeutic targets. Trends Parasitol (2018) 34(1):80–90. 29031509 10.1016/j.pt.2017.09.003 PMC5748246

[49] Grabitzki J Lochnit G. Immunomodulation by phosphocholine-biosynthesis, structures and immunological implications of parasitic PC-epitopes. Mol Immunol (2009) 47(2–3):149–63. 10.1016/j.molimm.2009.09.035 19864025

[50] Harnett W Harnett MM. Modulation of the host immune system by phosphorylcholine-containing glycoproteins secreted by parasitic filarial nematodes. Biochim Biophys Acta - Mol Cell Res (2001) 1539(1–2):7–15. 10.1016/s0167-4889(01)00101-x 11389964

[51] North SJ Botchway K Doonan J Lumb FE Dell A Harnett W. Site-specific glycoproteomic characterization of ES-62: The major secreted product of the parasitic worm acanthocheilonema viteae. Glycobiology. (2019) 29(8):562–71. 10.1093/glycob/cwz035 PMC6639541 31094418

[52] Ahmed UK Maller NC Iqbal AJ Al-Riyami L Harnett W Raynes JG. The carbohydrate-linked phosphorylcholine of the parasitic nematode product ES-62 modulates complement activation. J Biol Chem (2016) 291(22):11939–53. 10.1074/jbc.M115.702746 PMC4882459 27044740

[53] Dell A Haslam SM Morris HR Khoo KH. Immunogenic glycoconjugates implicated in parasitic nematode diseases. Biochim Biophys Acta - Mol Basis Dis (1999) 1455(2–3):353–62. 10.1016/s0925-4439(99)00064-2 10571024

[54] Lal RB Paranjape RS Briles DE Nutman TB Ottesen EA. Circulating parasite antigen(s) in lymphatic filariasis: use of monoclonal antibodies to phosphocholine for immunodiagnosis. J Immunol (1987) 138(10):3454–60. 2437195

[55] Paschinger K Wilson IBH. Comparisons of n-glycans across invertebrate phyla. Parasitology. (2019) 146(14):1733–42. 10.1017/S0031182019000398 PMC6935376 31046847

[56] Lal RB Dhawan RR Tarrand JJ Ayoub EM Ottesen E a. Lack of IgG4 antibody response to carbohydrate antigens in patients with lymphatic filariasis. Immunology (1991) 74(2):333–7. PMC1384614 1748481

[57] Lai RB Ottesen EA. Enhanced diagnostic specificity in human filariasis by IgG4 antibody assessment. J Infect Dis (1988) 158(5):1034–7. 10.1093/infdis/158.5.1034 2460565

[58] Scott MG Schackelford PG Briles DE Nahm MH. Human IgG subclasses and their relation to carbohydrate antigen immunocompetence. Diagn Clin Immunol (1988) 5(5):241–8. 3282712

[59] Schneider C Smith DF Cummings RD Boligan KF Hamilton RG Bochner BS. The human IgG anti-carbohydrate repertoire exhibits a universal architecture and contains specificity for microbial attachment sites. Sci Transl Med (2016) 7(269):1–23. 10.1126/scitranslmed.3010524 PMC4864610 25568069

[60] Vidarsson G Dekkers G Rispens T. IgG subclasses and allotypes: From structure to effector functions. Front Immunol (2014) 5(OCT):1–17. 25368619 10.3389/fimmu.2014.00520 PMC4202688

[61] Schur PH Rosen F Norman ME. Immunoglobulin subclasses in normal children. Pediatr Res (1979) 13(3):181–2. 10.1203/00006450-197903000-00010 471573

[62] Nieuwenhuys EJ Out TA. Comparison of normal values of IgG subclasses. CollProtides Biol fluids. (1989) 36:71–9.

[63] Mediannikov O Ranque S. Mansonellosis, the most neglected human filariasis. New Microbes New Infect (2018) 26:S19–22. 10.1016/j.nmni.2018.08.016 PMC6205574 30402239

