# Supplementary material for: Unraveling cross-reactivity of anti-glycan IgG responses in filarial nematode infections
Source: Front Immunol. 2023 Mar 6;14:1102344. doi: 10.3389/fimmu.2023.1102344 (PMC10026598; doi:10.3389/fimmu.2023.1102344)
Supplement: Supplementary file 1 [file Presentation_1.pptx]

## Slide 1
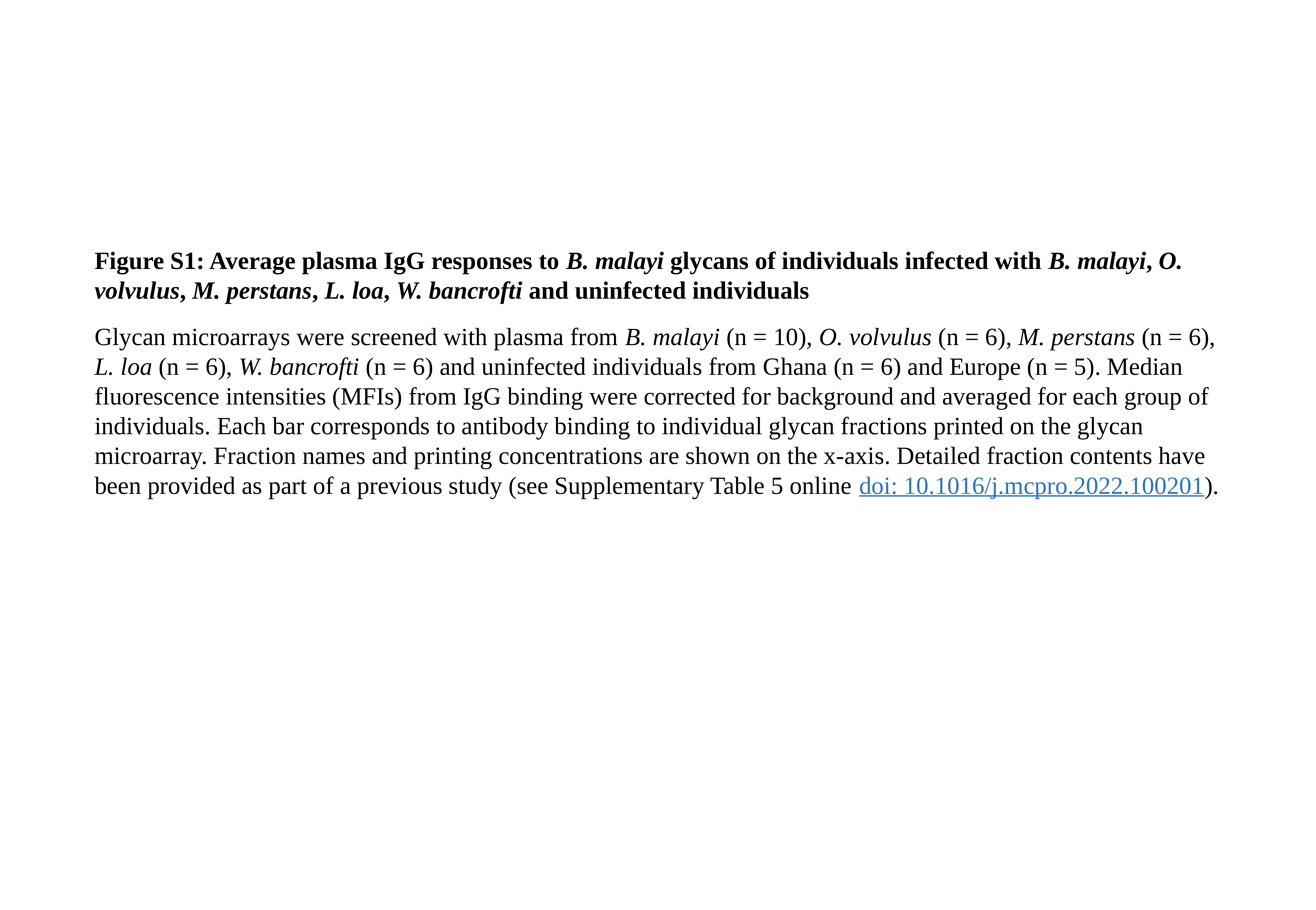

Figure S1: Average plasma IgG responses to B. malayi glycans of individuals infected with B. malayi, O. volvulus, M. perstans, L. loa, W. bancrofti and uninfected individuals
Glycan microarrays were screened with plasma from B. malayi (n = 10), O. volvulus (n = 6), M. perstans (n = 6), L. loa (n = 6), W. bancrofti (n = 6) and uninfected individuals from Ghana (n = 6) and Europe (n = 5). Median fluorescence intensities (MFIs) from IgG binding were corrected for background and averaged for each group of individuals. Each bar corresponds to antibody binding to individual glycan fractions printed on the glycan microarray. Fraction names and printing concentrations are shown on the x-axis. Detailed fraction contents have been provided as part of a previous study (see Supplementary Table 5 online doi: 10.1016/j.mcpro.2022.100201).

## Slide 2
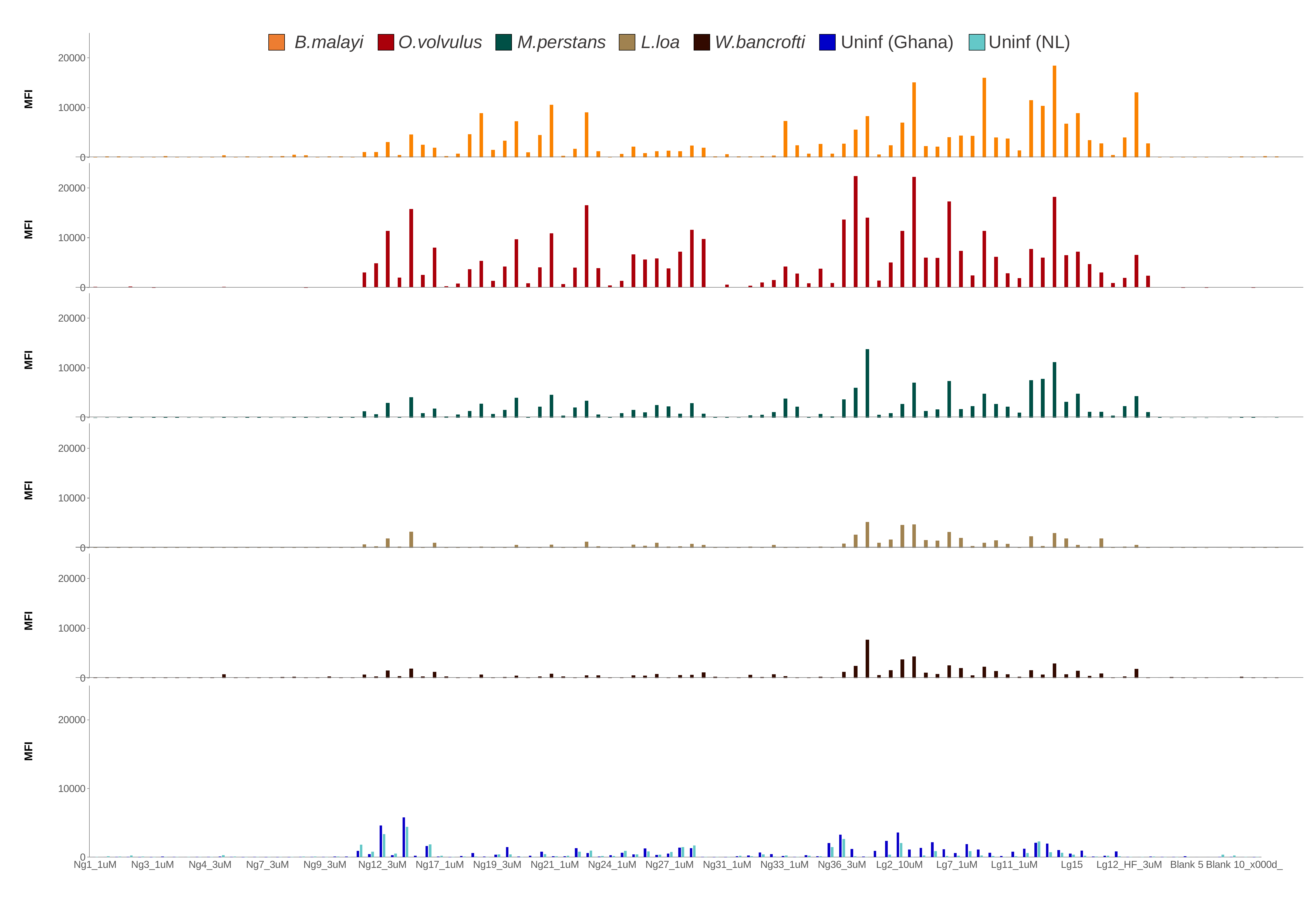

B.malayi O.volvulus M.perstans L.loa W.bancrofti Uninf (Ghana) Uninf (NL)
### Chart
| Category | Bm average |
|---|---|
| Ng 7-13 1µM_x000d_ | 138.66666666666666 |
| Ng 7-13 3µM_x000d_ | 198.4 |
| Ng 9-9 1 µM_x000d_ | 230.7 |
| Ng 9-9 3µM_x000d_ | 23.900000000000006 |
| Ng 9-9 10µM_x000d_ | 72.46666666666667 |
| Ng 10-13 1 µM_x000d_ | 59.1 |
| Ng 10-13 3 µM_x000d_ | 261.43333333333334 |
| Ng 10-13 10 µM_x000d_ | 112.43333333333332 |
| Ng 10-13 30 µM_x000d_ | 174.2666666666667 |
| Ng 10-10 1 µM_x000d_ | 155.03333333333333 |
| Ng 10-10 3 µM_x000d_ | 168.83333333333334 |
| Ng 10-11 1 µM_x000d_ | 425.69999999999993 |
| Ng 10-11 3 µM_x000d_ | 101.4 |
| Ng 10-12 1 µM_x000d_ | 224.13333333333335 |
| Ng 11-10 1 µM_x000d_ | 117.48333333333335 |
| Ng 11-10 3 µM_x000d_ | 197.18333333333334 |
| Ng 11-11 1 µM_x000d_ | 277.73333333333335 |
| Ng 11-11 3µM_x000d_ | 554.8333333333333 |
| Ng 11-11 10µM_x000d_ | 415.0666666666667 |
| Ng 12-11 1 µM_x000d_ | 69.56666666666666 |
| Ng 12-11 3 µM_x000d_ | 191.2666666666667 |
| Ng 13-15 1 µM_x000d_ | 208.60000000000005 |
| Ng 13-15 3 µM_x000d_ | 144.9666666666667 |
| Ng 13-14_x000d_ | 1092.9666666666667 |
| Ng 14-10 1 µM_x000d_ | 1063.7 |
| Ng 14-10 3 µM_x000d_ | 3108.7 |
| Ng 14-11 1 µM_x000d_ | 461.5000000000001 |
| Ng 14-12 1 µM_x000d_ | 4592.266666666666 |
| Ng 14-7 1 µM_x000d_ | 2572.3666666666663 |
| Ng 15-14/15 1 µM_x000d_ | 1922.6666666666667 |
| Ng 15-12 1µM_x000d_ | 247.71666666666664 |
| Ng 17-7 1 µM_x000d_ | 750.4333333333334 |
| Ng 17-7 3 µM_x000d_ | 4677.499999999999 |
| Ng 17-7 10 µM_x000d_ | 8895.766666666666 |
| Ng 19-9 1 µM_x000d_ | 1533.0333333333333 |
| Ng 19-9 3 µM_x000d_ | 3333.3 |
| Ng 19-9 10 µM_x000d_ | 7278.366666666667 |
| Ng 20-7 1 µM_x000d_ | 1005.7166666666668 |
| Ng 20-7 3 µM_x000d_ | 4476.933333333333 |
| Ng 20-7 10 µM_x000d_ | 10599.5 |
| Ng 21-8 1 µM_x000d_ | 336.4666666666667 |
| Ng 21-8 3 µM_x000d_ | 1707.55 |
| Ng 21-8 10 µM_x000d_ | 9051.8 |
| Ng 15-13 3µM_x000d_ | 1214.8333333333335 |
| Ng 16-12 1 µM_x000d_ | 84.63333333333335 |
| Ng 17-9 1 µM_x000d_ | 683.1 |
| Ng 17-9 3 µM_x000d_ | 2169.933333333333 |
| Ng 18-11 1 µM_x000d_ | 867.5666666666666 |
| Ng 18-11 3 µM_x000d_ | 1266.4333333333334 |
| Ng 20-8 1 µM_x000d_ | 1338.8333333333333 |
| Ng 20-9 1 µM_x000d_ | 1233.9333333333332 |
| Ng 20-9 3 µM_x000d_ | 2355.9333333333334 |
| Ng 21-9 1 µM_x000d_ | 1925.4 |
| Ng 13-13 3 µM_x000d_ | 206.20000000000005 |
| Ng 14-8 1 µM_x000d_ | 630.7666666666668 |
| Ng 14-9 1 µM_x000d_ | 207.63333333333335 |
| Ng 14-9 3 µM_x000d_ | 230.49999999999994 |
| Ng 14-9 10 µM_x000d_ | 289.4 |
| Ng 14-9 30 µM_x000d_ | 346.0333333333333 |
| Ng 15-10 1µM_x000d_ | 7331.466666666665 |
| Ng 16-9 1 µM_x000d_ | 2441.6 |
| Ng 17-8 1 µM_x000d_ | 751.5666666666668 |
| Ng 17-8 3 µM_x000d_ | 2729.5 |
| Ng 18-9 1µM_x000d_ | 733.05 |
| Ng 19-11 1 µM_x000d_ | 2782.033333333333 |
| Ng 19-11 3 µM_x000d_ | 5570.7333333333345 |
| Lg 17-15 HF 10 µM_x000d_ | 8305.5 |
| Lg 15-17 1 µM_x000d_ | 583.5666666666667 |
| Lg 17-15 1 µM_x000d_ | 2426.5333333333338 |
| Lg 17-15 3 µM_x000d_ | 6972.3 |
| Lg 17-15 10 µM_x000d_ | 15060.06666666667 |
| Lg 17-17 1 µM_x000d_ | 2284.666666666667 |
| Lg 17-16 1 µM_x000d_ | 2139.9333333333334 |
| Lg 16-17 3 µM_x000d_ | 4090.6 |
| Lg 18-8 1 µM_x000d_ | 4379.866666666667 |
| Lg 19-8 1 µM_x000d_ | 4317.5 |
| Lg 19-8 3 µM_x000d_ | 15975.533333333335 |
| Lg 19-9 1 µM_x000d_ | 4019.4 |
| Lg 20-10_x000d_ | 3771.8666666666672 |
| Lg 22-12 1 µM_x000d_ | 1401.1666666666665 |
| Lg 20-9 1 µM_x000d_ | 11522.366666666665 |
| Lg 23-11 1 µM_x000d_ | 10336.7 |
| Lg 23-11 3 µM_x000d_ | 18454.333333333336 |
| Lg 23-12_x000d_ | 6775.75 |
| Lg 26-8_x000d_ | 8895.133333333333 |
| Lg 26-9_x000d_ | 3446.5666666666666 |
| Lg 22-13_x000d_ | 2824.7333333333336 |
| Lg 26-10_x000d_ | 488.8666666666667 |
| Lg 18-7-F3 HF 1uM | 3999.4333333333334 |
| Lg 19-8-F5 HF 3uM | 13056.166666666668 |
| Lg 23-11-F2 HF 3uM | 2838.233333333333 |
| Blank 1 | 48.11666666666665 |
| Blank 2 | 62.23333333333331 |
| Blank 3 | 35.73333333333334 |
| Blank 4 | 156.4 |
| Blank 5 | 87.30000000000001 |
| Blank 6_x000d_ | 0.0 |
| Blank 7_x000d_ | 17.033333333333303 |
| Blank 8_x000d_ | 199.36666666666667 |
| Blank 9_x000d_ | 59.233333333333334 |
| Blank 10_x000d_ | 244.76666666666665 |
| Blank 11_x000d_ | 225.13333333333335 |
MFI
### Chart
| Category | Ov average |
|---|---|
| Ng 7-13 1µM_x000d_ | 136.7222222222222 |
| Ng 7-13 3µM_x000d_ | 59.55555555555557 |
| Ng 9-9 1 µM_x000d_ | 95.83333333333333 |
| Ng 9-9 3µM_x000d_ | 233.3333333333333 |
| Ng 9-9 10µM_x000d_ | 91.83333333333336 |
| Ng 10-13 1 µM_x000d_ | 18.833333333333346 |
| Ng 10-13 3 µM_x000d_ | 23.333333333333353 |
| Ng 10-13 10 µM_x000d_ | 70.44444444444444 |
| Ng 10-13 30 µM_x000d_ | 59.11111111111112 |
| Ng 10-10 1 µM_x000d_ | 91.22222222222223 |
| Ng 10-10 3 µM_x000d_ | 92.27777777777779 |
| Ng 10-11 1 µM_x000d_ | 175.41666666666669 |
| Ng 10-11 3 µM_x000d_ | 66.77777777777779 |
| Ng 10-12 1 µM_x000d_ | 108.72222222222221 |
| Ng 11-10 1 µM_x000d_ | 125.38888888888891 |
| Ng 11-10 3 µM_x000d_ | 53.55555555555554 |
| Ng 11-11 1 µM_x000d_ | 52.27777777777779 |
| Ng 11-11 3µM_x000d_ | 93.38888888888887 |
| Ng 11-11 10µM_x000d_ | 7.444444444444443 |
| Ng 12-11 1 µM_x000d_ | 55.444444444444436 |
| Ng 12-11 3 µM_x000d_ | 38.88888888888888 |
| Ng 13-15 1 µM_x000d_ | 44.11111111111111 |
| Ng 13-15 3 µM_x000d_ | 68.1111111111111 |
| Ng 13-14_x000d_ | 3018.111111111111 |
| Ng 14-10 1 µM_x000d_ | 4866.055555555555 |
| Ng 14-10 3 µM_x000d_ | 11403.055555555555 |
| Ng 14-11 1 µM_x000d_ | 2018.388888888889 |
| Ng 14-12 1 µM_x000d_ | 15790.944444444443 |
| Ng 14-7 1 µM_x000d_ | 2545.3333333333335 |
| Ng 15-14/15 1 µM_x000d_ | 8033.333333333333 |
| Ng 15-12 1µM_x000d_ | 286.6666666666667 |
| Ng 17-7 1 µM_x000d_ | 828.5555555555557 |
| Ng 17-7 3 µM_x000d_ | 3665.2222222222226 |
| Ng 17-7 10 µM_x000d_ | 5336.5 |
| Ng 19-9 1 µM_x000d_ | 1341.7777777777776 |
| Ng 19-9 3 µM_x000d_ | 4204.055555555556 |
| Ng 19-9 10 µM_x000d_ | 9698.944444444445 |
| Ng 20-7 1 µM_x000d_ | 865.5 |
| Ng 20-7 3 µM_x000d_ | 4068.7777777777774 |
| Ng 20-7 10 µM_x000d_ | 10923.666666666666 |
| Ng 21-8 1 µM_x000d_ | 680.7222222222222 |
| Ng 21-8 3 µM_x000d_ | 3997.8333333333335 |
| Ng 21-8 10 µM_x000d_ | 16518.0 |
| Ng 15-13 3µM_x000d_ | 3883.222222222222 |
| Ng 16-12 1 µM_x000d_ | 429.6666666666667 |
| Ng 17-9 1 µM_x000d_ | 1374.0 |
| Ng 17-9 3 µM_x000d_ | 6690.777777777777 |
| Ng 18-11 1 µM_x000d_ | 5608.833333333333 |
| Ng 18-11 3 µM_x000d_ | 5825.222222222223 |
| Ng 20-8 1 µM_x000d_ | 3848.8888888888887 |
| Ng 20-9 1 µM_x000d_ | 7213.583333333335 |
| Ng 20-9 3 µM_x000d_ | 11605.13888888889 |
| Ng 21-9 1 µM_x000d_ | 9774.888888888889 |
| Ng 13-13 3 µM_x000d_ | 54.583333333333314 |
| Ng 14-8 1 µM_x000d_ | 575.7777777777778 |
| Ng 14-9 1 µM_x000d_ | 26.222222222222225 |
| Ng 14-9 3 µM_x000d_ | 346.16666666666674 |
| Ng 14-9 10 µM_x000d_ | 1032.7222222222222 |
| Ng 14-9 30 µM_x000d_ | 1490.8333333333333 |
| Ng 15-10 1µM_x000d_ | 4211.166666666667 |
| Ng 16-9 1 µM_x000d_ | 2841.8333333333335 |
| Ng 17-8 1 µM_x000d_ | 862.7777777777777 |
| Ng 17-8 3 µM_x000d_ | 3808.2777777777774 |
| Ng 18-9 1µM_x000d_ | 891.4166666666666 |
| Ng 19-11 1 µM_x000d_ | 13648.0 |
| Ng 19-11 3 µM_x000d_ | 22391.5 |
| Lg 17-15 HF 10 µM_x000d_ | 14067.611111111111 |
| Lg 15-17 1 µM_x000d_ | 1414.0 |
| Lg 17-15 1 µM_x000d_ | 5059.611111111112 |
| Lg 17-15 3 µM_x000d_ | 11375.77777777778 |
| Lg 17-15 10 µM_x000d_ | 22217.77777777778 |
| Lg 17-17 1 µM_x000d_ | 6027.722222222222 |
| Lg 17-16 1 µM_x000d_ | 5973.222222222223 |
| Lg 16-17 3 µM_x000d_ | 17319.638888888887 |
| Lg 18-8 1 µM_x000d_ | 7362.333333333333 |
| Lg 19-8 1 µM_x000d_ | 2430.611111111111 |
| Lg 19-8 3 µM_x000d_ | 11366.444444444443 |
| Lg 19-9 1 µM_x000d_ | 6170.5 |
| Lg 20-10_x000d_ | 2852.277777777778 |
| Lg 22-12 1 µM_x000d_ | 1916.6666666666663 |
| Lg 20-9 1 µM_x000d_ | 7739.055555555556 |
| Lg 23-11 1 µM_x000d_ | 6035.888888888888 |
| Lg 23-11 3 µM_x000d_ | 18238.611111111113 |
| Lg 23-12_x000d_ | 6509.333333333333 |
| Lg 26-8_x000d_ | 7226.38888888889 |
| Lg 26-9_x000d_ | 4716.611111111111 |
| Lg 22-13_x000d_ | 3023.5 |
| Lg 26-10_x000d_ | 925.1666666666666 |
| Lg 18-7-F3 HF 1uM | 1948.3333333333333 |
| Lg 19-8-F5 HF 3uM | 6572.38888888889 |
| Lg 23-11-F2 HF 3uM | 2371.972222222222 |
| Blank 1 | 22.22222222222223 |
| Blank 2 | 20.888888888888896 |
| Blank 3 | 14.222222222222223 |
| Blank 4 | 0.0 |
| Blank 5 | 8.111111111111109 |
| Blank 6_x000d_ | 0.0 |
| Blank 7_x000d_ | 0.0 |
| Blank 8_x000d_ | 106.33333333333333 |
| Blank 9_x000d_ | 6.388888888888886 |
| Blank 10_x000d_ | 90.55555555555556 |
| Blank 11_x000d_ | 78.22222222222221 |MFI
### Chart
| Category | Mp average |
|---|---|
| Ng 7-13 1µM_x000d_ | 9.333333333333323 |
| Ng 7-13 3µM_x000d_ | 46.22222222222223 |
| Ng 9-9 1 µM_x000d_ | 46.66666666666666 |
| Ng 9-9 3µM_x000d_ | 141.83333333333334 |
| Ng 9-9 10µM_x000d_ | 22.33333333333333 |
| Ng 10-13 1 µM_x000d_ | 75.66666666666664 |
| Ng 10-13 3 µM_x000d_ | 77.94444444444444 |
| Ng 10-13 10 µM_x000d_ | 145.16666666666669 |
| Ng 10-13 30 µM_x000d_ | 54.888888888888886 |
| Ng 10-10 1 µM_x000d_ | 53.11111111111112 |
| Ng 10-10 3 µM_x000d_ | 14.944444444444443 |
| Ng 10-11 1 µM_x000d_ | 96.0 |
| Ng 10-11 3 µM_x000d_ | 65.61111111111111 |
| Ng 10-12 1 µM_x000d_ | 132.0 |
| Ng 11-10 1 µM_x000d_ | 83.27777777777776 |
| Ng 11-10 3 µM_x000d_ | 51.11111111111111 |
| Ng 11-11 1 µM_x000d_ | 10.388888888888886 |
| Ng 11-11 3µM_x000d_ | 219.05555555555554 |
| Ng 11-11 10µM_x000d_ | 149.2222222222222 |
| Ng 12-11 1 µM_x000d_ | 46.333333333333336 |
| Ng 12-11 3 µM_x000d_ | 80.72222222222223 |
| Ng 13-15 1 µM_x000d_ | 100.44444444444444 |
| Ng 13-15 3 µM_x000d_ | 196.44444444444446 |
| Ng 13-14_x000d_ | 1321.6666666666665 |
| Ng 14-10 1 µM_x000d_ | 678.3333333333334 |
| Ng 14-10 3 µM_x000d_ | 2997.444444444445 |
| Ng 14-11 1 µM_x000d_ | 186.88888888888889 |
| Ng 14-12 1 µM_x000d_ | 4117.277777777778 |
| Ng 14-7 1 µM_x000d_ | 909.2777777777778 |
| Ng 15-14/15 1 µM_x000d_ | 1816.9444444444443 |
| Ng 15-12 1µM_x000d_ | 282.0 |
| Ng 17-7 1 µM_x000d_ | 628.0555555555555 |
| Ng 17-7 3 µM_x000d_ | 1367.777777777778 |
| Ng 17-7 10 µM_x000d_ | 2808.222222222222 |
| Ng 19-9 1 µM_x000d_ | 759.611111111111 |
| Ng 19-9 3 µM_x000d_ | 1541.5 |
| Ng 19-9 10 µM_x000d_ | 4003.5 |
| Ng 20-7 1 µM_x000d_ | 226.16666666666666 |
| Ng 20-7 3 µM_x000d_ | 2197.8333333333335 |
| Ng 20-7 10 µM_x000d_ | 4598.777777777778 |
| Ng 21-8 1 µM_x000d_ | 415.0555555555556 |
| Ng 21-8 3 µM_x000d_ | 2033.6666666666667 |
| Ng 21-8 10 µM_x000d_ | 3397.444444444444 |
| Ng 15-13 3µM_x000d_ | 637.8333333333334 |
| Ng 16-12 1 µM_x000d_ | 173.2222222222222 |
| Ng 17-9 1 µM_x000d_ | 914.2777777777777 |
| Ng 17-9 3 µM_x000d_ | 1589.6111111111113 |
| Ng 18-11 1 µM_x000d_ | 1060.7222222222222 |
| Ng 18-11 3 µM_x000d_ | 2531.5 |
| Ng 20-8 1 µM_x000d_ | 2261.1666666666665 |
| Ng 20-9 1 µM_x000d_ | 813.0 |
| Ng 20-9 3 µM_x000d_ | 2920.444444444444 |
| Ng 21-9 1 µM_x000d_ | 830.888888888889 |
| Ng 13-13 3 µM_x000d_ | 100.77777777777779 |
| Ng 14-8 1 µM_x000d_ | 207.33333333333334 |
| Ng 14-9 1 µM_x000d_ | 48.22222222222222 |
| Ng 14-9 3 µM_x000d_ | 507.66666666666674 |
| Ng 14-9 10 µM_x000d_ | 570.6111111111112 |
| Ng 14-9 30 µM_x000d_ | 1105.7777777777776 |
| Ng 15-10 1µM_x000d_ | 3859.277777777778 |
| Ng 16-9 1 µM_x000d_ | 2230.8888888888887 |
| Ng 17-8 1 µM_x000d_ | 201.55555555555557 |
| Ng 17-8 3 µM_x000d_ | 724.6111111111112 |
| Ng 18-9 1µM_x000d_ | 255.66666666666666 |
| Ng 19-11 1 µM_x000d_ | 3664.6111111111113 |
| Ng 19-11 3 µM_x000d_ | 6033.166666666667 |
| Lg 17-15 HF 10 µM_x000d_ | 13787.611111111111 |
| Lg 15-17 1 µM_x000d_ | 568.8333333333334 |
| Lg 17-15 1 µM_x000d_ | 909.4999999999999 |
| Lg 17-15 3 µM_x000d_ | 2783.6666666666665 |
| Lg 17-15 10 µM_x000d_ | 7040.0 |
| Lg 17-17 1 µM_x000d_ | 1373.0555555555554 |
| Lg 17-16 1 µM_x000d_ | 1649.333333333333 |
| Lg 16-17 3 µM_x000d_ | 7389.0555555555575 |
| Lg 18-8 1 µM_x000d_ | 1742.3333333333333 |
| Lg 19-8 1 µM_x000d_ | 2321.7222222222226 |
| Lg 19-8 3 µM_x000d_ | 4822.555555555556 |
| Lg 19-9 1 µM_x000d_ | 2770.0 |
| Lg 20-10_x000d_ | 2191.0 |
| Lg 22-12 1 µM_x000d_ | 1024.1666666666667 |
| Lg 20-9 1 µM_x000d_ | 7552.222222222222 |
| Lg 23-11 1 µM_x000d_ | 7799.388888888888 |
| Lg 23-11 3 µM_x000d_ | 11158.5 |
| Lg 23-12_x000d_ | 3180.0 |
| Lg 26-8_x000d_ | 4802.722222222223 |
| Lg 26-9_x000d_ | 1172.611111111111 |
| Lg 22-13_x000d_ | 1181.5 |
| Lg 26-10_x000d_ | 417.7777777777778 |
| Lg 18-7-F3 HF 1uM | 2336.111111111111 |
| Lg 19-8-F5 HF 3uM | 4333.555555555556 |
| Lg 23-11-F2 HF 3uM | 1113.1666666666667 |
| Blank 1 | 166.44444444444446 |
| Blank 2 | 2.777777777777781 |
| Blank 3 | 32.61111111111109 |
| Blank 4 | 8.666666666666666 |
| Blank 5 | 10.611111111111105 |
| Blank 6_x000d_ | 0.0 |
| Blank 7_x000d_ | 17.33333333333333 |
| Blank 8_x000d_ | 89.05555555555553 |
| Blank 9_x000d_ | 99.83333333333331 |
| Blank 10_x000d_ | 0.0 |
| Blank 11_x000d_ | 45.72222222222221 |MFI
### Chart
| Category | Ll average |
|---|---|
| Ng 7-13 1µM_x000d_ | 29.05555555555556 |
| Ng 7-13 3µM_x000d_ | 85.72222222222223 |
| Ng 9-9 1 µM_x000d_ | 104.5 |
| Ng 9-9 3µM_x000d_ | 162.44444444444446 |
| Ng 9-9 10µM_x000d_ | 21.333333333333332 |
| Ng 10-13 1 µM_x000d_ | 76.44444444444443 |
| Ng 10-13 3 µM_x000d_ | 73.27777777777777 |
| Ng 10-13 10 µM_x000d_ | 142.38888888888889 |
| Ng 10-13 30 µM_x000d_ | 145.88888888888889 |
| Ng 10-10 1 µM_x000d_ | 108.44444444444446 |
| Ng 10-10 3 µM_x000d_ | 136.6111111111111 |
| Ng 10-11 1 µM_x000d_ | 213.55555555555554 |
| Ng 10-11 3 µM_x000d_ | 158.7222222222222 |
| Ng 10-12 1 µM_x000d_ | 148.16666666666666 |
| Ng 11-10 1 µM_x000d_ | 50.72222222222223 |
| Ng 11-10 3 µM_x000d_ | 126.11111111111109 |
| Ng 11-11 1 µM_x000d_ | 151.66666666666666 |
| Ng 11-11 3µM_x000d_ | 163.00000000000003 |
| Ng 11-11 10µM_x000d_ | 156.16666666666666 |
| Ng 12-11 1 µM_x000d_ | 143.2777777777778 |
| Ng 12-11 3 µM_x000d_ | 117.66666666666667 |
| Ng 13-15 1 µM_x000d_ | 63.50000000000001 |
| Ng 13-15 3 µM_x000d_ | 69.72222222222221 |
| Ng 13-14_x000d_ | 703.8333333333335 |
| Ng 14-10 1 µM_x000d_ | 322.72222222222223 |
| Ng 14-10 3 µM_x000d_ | 1886.111111111111 |
| Ng 14-11 1 µM_x000d_ | 280.0555555555556 |
| Ng 14-12 1 µM_x000d_ | 3240.222222222222 |
| Ng 14-7 1 µM_x000d_ | 186.44444444444446 |
| Ng 15-14/15 1 µM_x000d_ | 1020.8333333333334 |
| Ng 15-12 1µM_x000d_ | 85.1111111111111 |
| Ng 17-7 1 µM_x000d_ | 35.138888888888886 |
| Ng 17-7 3 µM_x000d_ | 94.72222222222223 |
| Ng 17-7 10 µM_x000d_ | 251.50000000000003 |
| Ng 19-9 1 µM_x000d_ | 100.88888888888887 |
| Ng 19-9 3 µM_x000d_ | 229.2777777777778 |
| Ng 19-9 10 µM_x000d_ | 617.3333333333334 |
| Ng 20-7 1 µM_x000d_ | 45.833333333333336 |
| Ng 20-7 3 µM_x000d_ | 175.2777777777778 |
| Ng 20-7 10 µM_x000d_ | 618.6666666666666 |
| Ng 21-8 1 µM_x000d_ | 213.55555555555554 |
| Ng 21-8 3 µM_x000d_ | 109.05555555555559 |
| Ng 21-8 10 µM_x000d_ | 1233.7222222222222 |
| Ng 15-13 3µM_x000d_ | 345.72222222222223 |
| Ng 16-12 1 µM_x000d_ | 60.27777777777777 |
| Ng 17-9 1 µM_x000d_ | 161.61111111111111 |
| Ng 17-9 3 µM_x000d_ | 655.1666666666667 |
| Ng 18-11 1 µM_x000d_ | 438.2222222222222 |
| Ng 18-11 3 µM_x000d_ | 1048.4444444444443 |
| Ng 20-8 1 µM_x000d_ | 286.55555555555554 |
| Ng 20-9 1 µM_x000d_ | 306.77777777777777 |
| Ng 20-9 3 µM_x000d_ | 798.7222222222222 |
| Ng 21-9 1 µM_x000d_ | 596.8888888888888 |
| Ng 13-13 3 µM_x000d_ | 144.0 |
| Ng 14-8 1 µM_x000d_ | 100.05555555555554 |
| Ng 14-9 1 µM_x000d_ | 84.00000000000001 |
| Ng 14-9 3 µM_x000d_ | 269.00000000000006 |
| Ng 14-9 10 µM_x000d_ | 234.66666666666663 |
| Ng 14-9 30 µM_x000d_ | 577.3888888888888 |
| Ng 15-10 1µM_x000d_ | 137.33333333333334 |
| Ng 16-9 1 µM_x000d_ | 101.55555555555556 |
| Ng 17-8 1 µM_x000d_ | 56.83333333333332 |
| Ng 17-8 3 µM_x000d_ | 242.22222222222226 |
| Ng 18-9 1µM_x000d_ | 63.61111111111112 |
| Ng 19-11 1 µM_x000d_ | 866.1666666666665 |
| Ng 19-11 3 µM_x000d_ | 2664.5000000000005 |
| Lg 17-15 HF 10 µM_x000d_ | 5177.11111111111 |
| Lg 15-17 1 µM_x000d_ | 1026.111111111111 |
| Lg 17-15 1 µM_x000d_ | 1697.3888888888887 |
| Lg 17-15 3 µM_x000d_ | 4631.222222222222 |
| Lg 17-15 10 µM_x000d_ | 4699.333333333333 |
| Lg 17-17 1 µM_x000d_ | 1564.388888888889 |
| Lg 17-16 1 µM_x000d_ | 1456.388888888889 |
| Lg 16-17 3 µM_x000d_ | 3213.444444444445 |
| Lg 18-8 1 µM_x000d_ | 2005.277777777778 |
| Lg 19-8 1 µM_x000d_ | 397.66666666666674 |
| Lg 19-8 3 µM_x000d_ | 1019.7222222222222 |
| Lg 19-9 1 µM_x000d_ | 1489.4444444444443 |
| Lg 20-10_x000d_ | 808.2222222222222 |
| Lg 22-12 1 µM_x000d_ | 208.88888888888889 |
| Lg 20-9 1 µM_x000d_ | 2333.1666666666665 |
| Lg 23-11 1 µM_x000d_ | 351.0555555555556 |
| Lg 23-11 3 µM_x000d_ | 3001.6388888888887 |
| Lg 23-12_x000d_ | 1879.7222222222224 |
| Lg 26-8_x000d_ | 566.2777777777778 |
| Lg 26-9_x000d_ | 278.4444444444445 |
| Lg 22-13_x000d_ | 1901.5555555555554 |
| Lg 26-10_x000d_ | 22.833333333333332 |
| Lg 18-7-F3 HF 1uM | 262.5555555555556 |
| Lg 19-8-F5 HF 3uM | 576.3333333333334 |
| Lg 23-11-F2 HF 3uM | 122.55555555555554 |
| Blank 1 | 0.0 |
| Blank 2 | 108.88888888888891 |
| Blank 3 | 54.33333333333332 |
| Blank 4 | 112.77777777777779 |
| Blank 5 | 7.388888888888886 |
| Blank 6_x000d_ | 0.0 |
| Blank 7_x000d_ | 16.777777777777782 |
| Blank 8_x000d_ | 46.00000000000001 |
| Blank 9_x000d_ | 59.33333333333332 |
| Blank 10_x000d_ | 94.22222222222223 |
| Blank 11_x000d_ | 62.833333333333314 |MFI
### Chart
| Category | Wb average |
|---|---|
| Ng 7-13 1µM_x000d_ | 111.44444444444444 |
| Ng 7-13 3µM_x000d_ | 118.72222222222221 |
| Ng 9-9 1 µM_x000d_ | 20.99999999999999 |
| Ng 9-9 3µM_x000d_ | 166.61111111111114 |
| Ng 9-9 10µM_x000d_ | 116.6111111111111 |
| Ng 10-13 1 µM_x000d_ | 122.22222222222224 |
| Ng 10-13 3 µM_x000d_ | 74.11111111111111 |
| Ng 10-13 10 µM_x000d_ | 80.44444444444444 |
| Ng 10-13 30 µM_x000d_ | 158.6111111111111 |
| Ng 10-10 1 µM_x000d_ | 20.44444444444444 |
| Ng 10-10 3 µM_x000d_ | 41.83333333333332 |
| Ng 10-11 1 µM_x000d_ | 741.1666666666666 |
| Ng 10-11 3 µM_x000d_ | 101.88888888888891 |
| Ng 10-12 1 µM_x000d_ | 124.22222222222224 |
| Ng 11-10 1 µM_x000d_ | 110.77777777777777 |
| Ng 11-10 3 µM_x000d_ | 71.11111111111113 |
| Ng 11-11 1 µM_x000d_ | 197.7777777777778 |
| Ng 11-11 3µM_x000d_ | 286.0555555555555 |
| Ng 11-11 10µM_x000d_ | 164.94444444444443 |
| Ng 12-11 1 µM_x000d_ | 181.47222222222217 |
| Ng 12-11 3 µM_x000d_ | 329.5555555555556 |
| Ng 13-15 1 µM_x000d_ | 153.44444444444443 |
| Ng 13-15 3 µM_x000d_ | 104.72222222222221 |
| Ng 13-14_x000d_ | 679.1666666666667 |
| Ng 14-10 1 µM_x000d_ | 335.72222222222223 |
| Ng 14-10 3 µM_x000d_ | 1491.2777777777776 |
| Ng 14-11 1 µM_x000d_ | 346.9444444444445 |
| Ng 14-12 1 µM_x000d_ | 1891.3888888888894 |
| Ng 14-7 1 µM_x000d_ | 330.05555555555554 |
| Ng 15-14/15 1 µM_x000d_ | 1267.9444444444446 |
| Ng 15-12 1µM_x000d_ | 317.3888888888889 |
| Ng 17-7 1 µM_x000d_ | 20.666666666666668 |
| Ng 17-7 3 µM_x000d_ | 121.88888888888887 |
| Ng 17-7 10 µM_x000d_ | 689.6111111111112 |
| Ng 19-9 1 µM_x000d_ | 140.5 |
| Ng 19-9 3 µM_x000d_ | 221.0 |
| Ng 19-9 10 µM_x000d_ | 457.0555555555555 |
| Ng 20-7 1 µM_x000d_ | 66.55555555555556 |
| Ng 20-7 3 µM_x000d_ | 295.0 |
| Ng 20-7 10 µM_x000d_ | 875.4444444444445 |
| Ng 21-8 1 µM_x000d_ | 325.27777777777777 |
| Ng 21-8 3 µM_x000d_ | 116.38888888888886 |
| Ng 21-8 10 µM_x000d_ | 525.9999999999999 |
| Ng 15-13 3µM_x000d_ | 522.0555555555555 |
| Ng 16-12 1 µM_x000d_ | 92.72222222222221 |
| Ng 17-9 1 µM_x000d_ | 41.833333333333336 |
| Ng 17-9 3 µM_x000d_ | 528.8333333333333 |
| Ng 18-11 1 µM_x000d_ | 461.9444444444445 |
| Ng 18-11 3 µM_x000d_ | 814.4999999999999 |
| Ng 20-8 1 µM_x000d_ | 134.72222222222223 |
| Ng 20-9 1 µM_x000d_ | 572.2777777777777 |
| Ng 20-9 3 µM_x000d_ | 642.9444444444445 |
| Ng 21-9 1 µM_x000d_ | 1158.7777777777778 |
| Ng 13-13 3 µM_x000d_ | 240.16666666666666 |
| Ng 14-8 1 µM_x000d_ | 73.77777777777776 |
| Ng 14-9 1 µM_x000d_ | 60.611111111111114 |
| Ng 14-9 3 µM_x000d_ | 629.0 |
| Ng 14-9 10 µM_x000d_ | 200.83333333333334 |
| Ng 14-9 30 µM_x000d_ | 750.6666666666666 |
| Ng 15-10 1µM_x000d_ | 355.4444444444444 |
| Ng 16-9 1 µM_x000d_ | 145.44444444444443 |
| Ng 17-8 1 µM_x000d_ | 103.88888888888891 |
| Ng 17-8 3 µM_x000d_ | 273.47222222222223 |
| Ng 18-9 1µM_x000d_ | 101.38888888888887 |
| Ng 19-11 1 µM_x000d_ | 1262.7222222222222 |
| Ng 19-11 3 µM_x000d_ | 2418.3333333333335 |
| Lg 17-15 HF 10 µM_x000d_ | 7690.666666666667 |
| Lg 15-17 1 µM_x000d_ | 591.5000000000001 |
| Lg 17-15 1 µM_x000d_ | 1575.4444444444443 |
| Lg 17-15 3 µM_x000d_ | 3714.722222222222 |
| Lg 17-15 10 µM_x000d_ | 4351.111111111111 |
| Lg 17-17 1 µM_x000d_ | 1073.1666666666665 |
| Lg 17-16 1 µM_x000d_ | 826.1666666666666 |
| Lg 16-17 3 µM_x000d_ | 2545.222222222222 |
| Lg 18-8 1 µM_x000d_ | 1995.0555555555557 |
| Lg 19-8 1 µM_x000d_ | 509.66666666666674 |
| Lg 19-8 3 µM_x000d_ | 2253.1666666666665 |
| Lg 19-9 1 µM_x000d_ | 1397.388888888889 |
| Lg 20-10_x000d_ | 742.1666666666666 |
| Lg 22-12 1 µM_x000d_ | 272.0555555555556 |
| Lg 20-9 1 µM_x000d_ | 1546.611111111111 |
| Lg 23-11 1 µM_x000d_ | 724.3333333333331 |
| Lg 23-11 3 µM_x000d_ | 2932.1666666666665 |
| Lg 23-12_x000d_ | 761.2222222222222 |
| Lg 26-8_x000d_ | 1463.0555555555557 |
| Lg 26-9_x000d_ | 427.38888888888874 |
| Lg 22-13_x000d_ | 939.4444444444443 |
| Lg 26-10_x000d_ | 25.888888888888896 |
| Lg 18-7-F3 HF 1uM | 323.5555555555556 |
| Lg 19-8-F5 HF 3uM | 1846.888888888889 |
| Lg 23-11-F2 HF 3uM | 47.77777777777778 |
| Blank 1 | 0.0 |
| Blank 2 | 209.16666666666666 |
| Blank 3 | 27.50000000000001 |
| Blank 4 | 3.388888888888895 |
| Blank 5 | 40.833333333333336 |
| Blank 6_x000d_ | 0.0 |
| Blank 7_x000d_ | 0.0 |
| Blank 8_x000d_ | 268.4444444444444 |
| Blank 9_x000d_ | 148.44444444444446 |
| Blank 10_x000d_ | 148.05555555555554 |
| Blank 11_x000d_ | 112.5 |MFI
### Chart
| Category | UG average | UE average |
|---|---|---|
| Ng1_1uM
 | 0.0 | 23.133333333333336 |
| Ng1_3uM
 | 0.0 | 116.33333333333334 |
| Ng2_1uM | 26.944444444444443 | 84.60000000000002 |
| Ng2_3uM | 33.333333333333336 | 243.26666666666665 |
| Ng2_10uM | 1.0555555555555571 | 92.13333333333334 |
| Ng3_1uM | 4.666666666666667 | 7.3333333333333375 |
| Ng3_3uM | 86.16666666666667 | 40.36666666666667 |
| Ng3_10uM | 48.444444444444436 | 47.6 |
| Ng3_30uM | 0.0 | 34.733333333333334 |
| Ng4_1uM | 28.111111111111114 | 31.066666666666663 |
| Ng4_3uM | 22.833333333333332 | 19.333333333333336 |
| Ng5_1uM | 81.77777777777777 | 280.1 |
| Ng5_3uM | 44.666666666666664 | 83.66666666666666 |
| Ng6_1uM | 3.666666666666662 | 7.199999999999994 |
| Ng7_1uM | 23.16666666666667 | 31.133333333333347 |
| Ng7_3uM | 3.7777777777777763 | 32.46666666666666 |
| Ng8_1uM | 4.5 | 36.66666666666667 |
| Ng8_3uM | 4.555555555555557 | 25.733333333333338 |
| Ng8_10uM | 44.77777777777778 | 80.86666666666665 |
| Ng9_1uM | 36.94444444444444 | 69.0 |
| Ng9_3uM | 43.11111111111111 | 11.666666666666668 |
| Ng10_3uM | 86.27777777777779 | 91.13333333333335 |
| Ng10_1uM | 70.33333333333333 | 45.33333333333333 |
| Ng11 | 894.6666666666666 | 1807.6666666666665 |
| Ng12_1uM | 429.8333333333333 | 788.2666666666667 |
| Ng12_3uM | 4606.944444444444 | 3350.1333333333337 |
| Ng13_1uM | 252.94444444444443 | 492.0666666666667 |
| Ng14_1uM | 5769.666666666667 | 4400.133333333334 |
| Ng15_1uM | 172.61111111111111 | 9.866666666666651 |
| Ng16_1uM | 1615.8888888888885 | 1859.3333333333333 |
| Ng17_1uM | 62.72222222222222 | 173.93333333333334 |
| Ng18_1uM | 14.444444444444443 | 24.133333333333333 |
| Ng18_3uM | 137.22222222222223 | 90.63333333333335 |
| Ng18_10uM | 604.5555555555555 | 91.13333333333333 |
| Ng19_1uM | 89.55555555555556 | 7.733333333333332 |
| Ng19_3uM | 357.8333333333333 | 376.93333333333334 |
| Ng19_10uM | 1445.1666666666667 | 408.2 |
| Ng20_1uM | 59.38888888888889 | 46.6 |
| Ng20_3uM | 204.55555555555554 | 43.466666666666676 |
| Ng20_10uM | 775.0555555555557 | 431.0 |
| Ng21_1uM | 97.33333333333336 | 122.26666666666665 |
| Ng21 _3uM | 110.55555555555559 | 174.2 |
| Ng21_10uM | 1288.2777777777778 | 767.2666666666667 |
| Ng22_1uM | 581.2222222222223 | 949.8 |
| Ng23_1uM | 78.0 | 134.79999999999998 |
| Ng24_1uM | 262.3333333333333 | 168.4 |
| Ng24_3uM | 620.7222222222223 | 919.3333333333334 |
| Ng25_1uM | 407.6111111111111 | 392.9 |
| Ng25_3uM | 1262.2777777777778 | 829.8666666666666 |
| Ng26_1uM | 317.0555555555556 | 335.93333333333334 |
| Ng27_1uM | 515.0555555555555 | 742.8666666666666 |
| Ng27_3uM | 1356.0555555555554 | 1469.2 |
| Ng28_1uM | 1307.2777777777778 | 1690.8666666666663 |
| Ng29_1uM | 20.833333333333332 | 19.333333333333336 |
| Ng30_1uM | 15.611111111111114 | 42.13333333333334 |
| Ng31_1uM | 1.6666666666666667 | 37.533333333333324 |
| Ng31_3uM | 125.16666666666667 | 188.66666666666663 |
| Ng31_10uM | 229.8333333333333 | 124.53333333333335 |
| Ng31_30uM | 677.5 | 392.5333333333333 |
| Ng32_1uM | 446.83333333333326 | 91.06666666666666 |
| Ng33_1uM | 146.16666666666666 | 214.8666666666667 |
| Ng34_1uM | 52.27777777777777 | 35.933333333333344 |
| Ng34_3uM | 272.94444444444446 | 175.8 |
| Ng35_1uM | 121.33333333333333 | 117.00000000000003 |
| Ng36_1uM | 2032.2777777777776 | 1467.9333333333334 |
| Ng36_3uM | 3250.611111111111 | 2621.666666666667 |
| Lg2_HF_10uM | 1162.6666666666667 | 132.26666666666665 |
| Lg1_1uM | 77.3888888888889 | 38.60000000000001 |
| Lg2_1uM | 896.9444444444445 | 30.866666666666664 |
| Lg2_3uM | 2373.8333333333335 | 368.6 |
| Lg2_10uM | 3575.3888888888887 | 2047.2666666666664 |
| Lg3_1uM | 1096.7777777777778 | 53.533333333333324 |
| Lg4_1uM | 1334.4999999999998 | 244.7 |
| Lg5_3uM | 2179.0 | 853.6666666666666 |
| Lg6_1uM | 1121.1111111111113 | 156.7 |
| Lg7_1uM | 584.2222222222223 | 196.40000000000003 |
| Lg7_3uM | 1874.5555555555557 | 865.1333333333334 |
| Lg8_1uM | 1090.2777777777776 | 219.8 |
| Lg9 | 643.2777777777777 | 146.0666666666667 |
| Lg10_1uM | 165.05555555555554 | 25.266666666666662 |
| Lg11_1uM | 774.6666666666666 | 97.93333333333335 |
| Lg12_1uM | 1219.8333333333335 | 602.0 |
| Lg12_3uM | 2082.611111111111 | 2279.7333333333336 |
| Lg13 | 1980.3888888888894 | 686.9333333333333 |
| Lg14 | 1007.9444444444445 | 617.3333333333333 |
| Lg15 | 519.1111111111111 | 340.7333333333333 |
| Lg16 | 931.8333333333334 | 180.13333333333333 |
| Lg17 | 57.94444444444446 | 75.4666666666667 |
| Lg18_HF_1uM | 196.55555555555554 | 177.53333333333336 |
| Lg7_HF_3uM | 837.0 | 120.8 |
| Lg12_HF_3uM | 36.72222222222222 | 26.8 |
| Blank 1 | 0.0 | 38.06666666666665 |
| Blank 2 | 57.72222222222223 | 91.80000000000003 |
| Blank 3 | 19.500000000000004 | 12.0 |
| Blank 4 | 26.83333333333333 | 9.6 |
| Blank 5 | 114.83333333333333 | 49.33333333333333 |
| Blank 6_x000d_ | 0.0 | 0.0 |
| Blank 7_x000d_ | 0.0 | 0.0 |
| Blank 8_x000d_ | 28.222222222222218 | 340.9333333333333 |
| Blank 9_x000d_ | 21.944444444444443 | 234.4666666666667 |
| Blank 10_x000d_ | 0.0 | 48.066666666666684 |
| Blank 11_x000d_ | 13.444444444444443 | 25.46666666666666 |MFI

## Slide 3
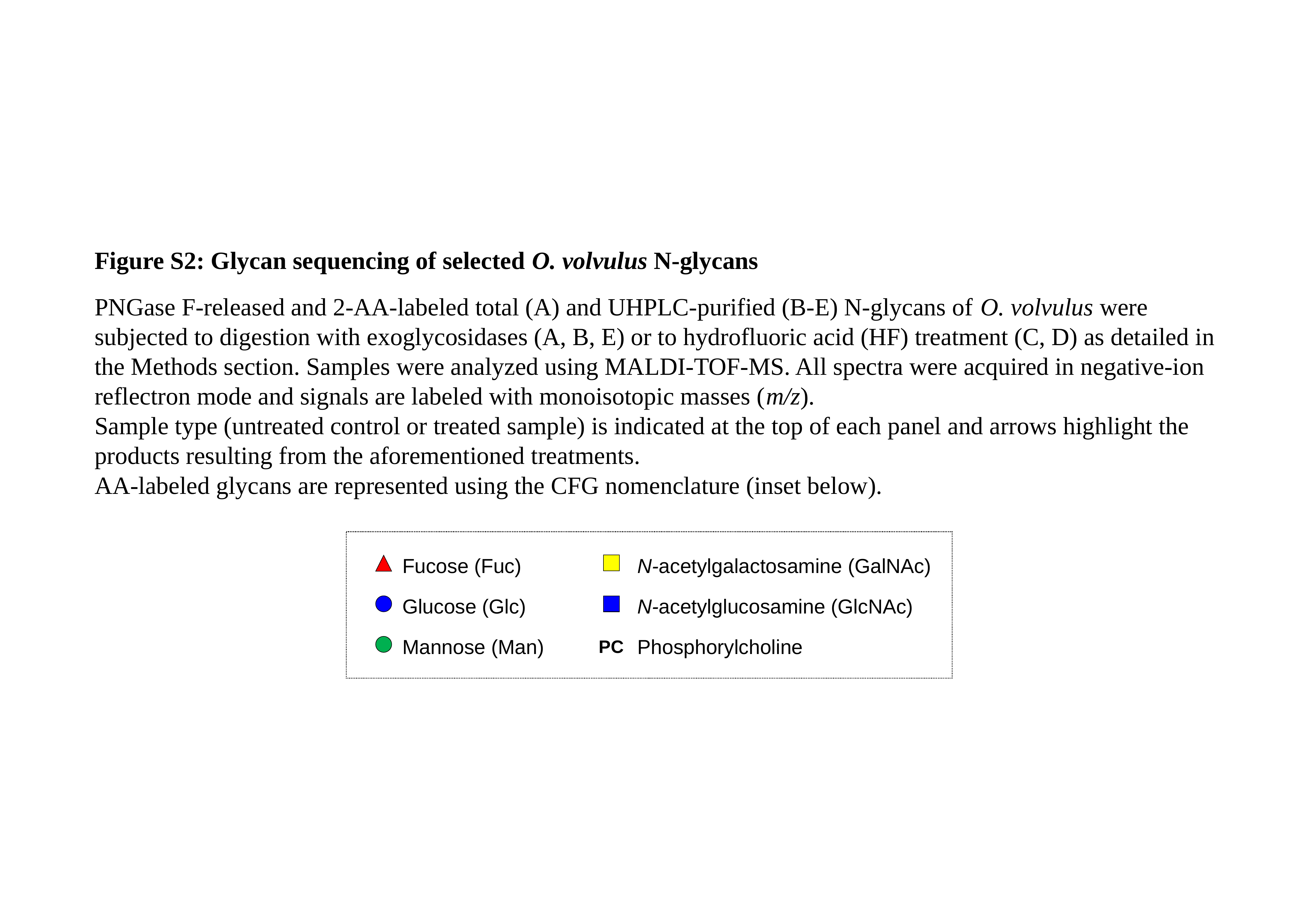

Figure S2: Glycan sequencing of selected O. volvulus N-glycans
PNGase F-released and 2-AA-labeled total (A) and UHPLC-purified (B-E) N-glycans of O. volvulus were subjected to digestion with exoglycosidases (A, B, E) or to hydrofluoric acid (HF) treatment (C, D) as detailed in the Methods section. Samples were analyzed using MALDI-TOF-MS. All spectra were acquired in negative-ion reflectron mode and signals are labeled with monoisotopic masses (m/z).
Sample type (untreated control or treated sample) is indicated at the top of each panel and arrows highlight the products resulting from the aforementioned treatments.
AA-labeled glycans are represented using the CFG nomenclature (inset below).
Fucose (Fuc)
N-acetylgalactosamine (GalNAc)
Glucose (Glc)
N-acetylglucosamine (GlcNAc)
Mannose (Man)
Phosphorylcholine
PC

## Slide 4
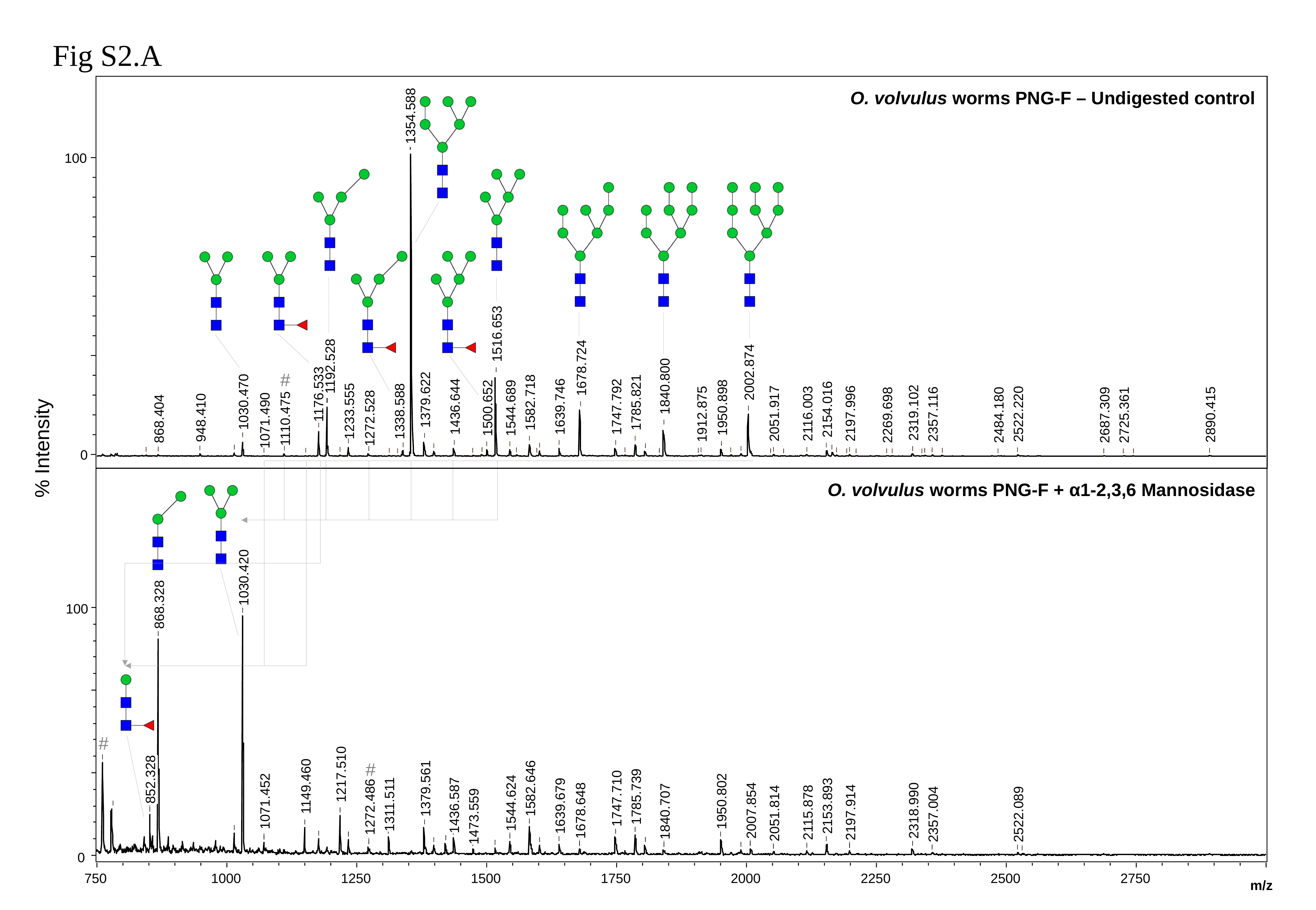

Fig S2.A
O. volvulus worms PNG-F – Undigested control
1354.588
100
1516.653
1192.528
1678.724
2002.874
#
1840.800
1176.533
1379.622
1030.470
1582.718
1785.821
1436.644
1639.746
1747.792
1950.898
1500.652
1544.689
2154.016
1233.555
1338.588
2319.102
2051.917
2116.003
2197.996
2357.116
2522.220
1912.875
2890.415
2269.698
2484.180
2687.309
2725.361
948.410
1272.528
1110.475
868.404
1071.490
% Intensity
0
O. volvulus worms PNG-F + α1-2,3,6 Mannosidase
1030.420
868.328
100
#
#
1217.510
852.328
1149.460
1582.646
1379.561
1785.739
1747.710
1071.452
1950.802
1544.624
1311.511
1436.587
1639.679
2153.893
1272.486
1678.648
2007.854
2318.990
1840.707
2115.878
2197.914
2051.814
2357.004
2522.089
1473.559
0
750
1000
1250
1500
1750
2000
2250
2500
2750
m/z

## Slide 5
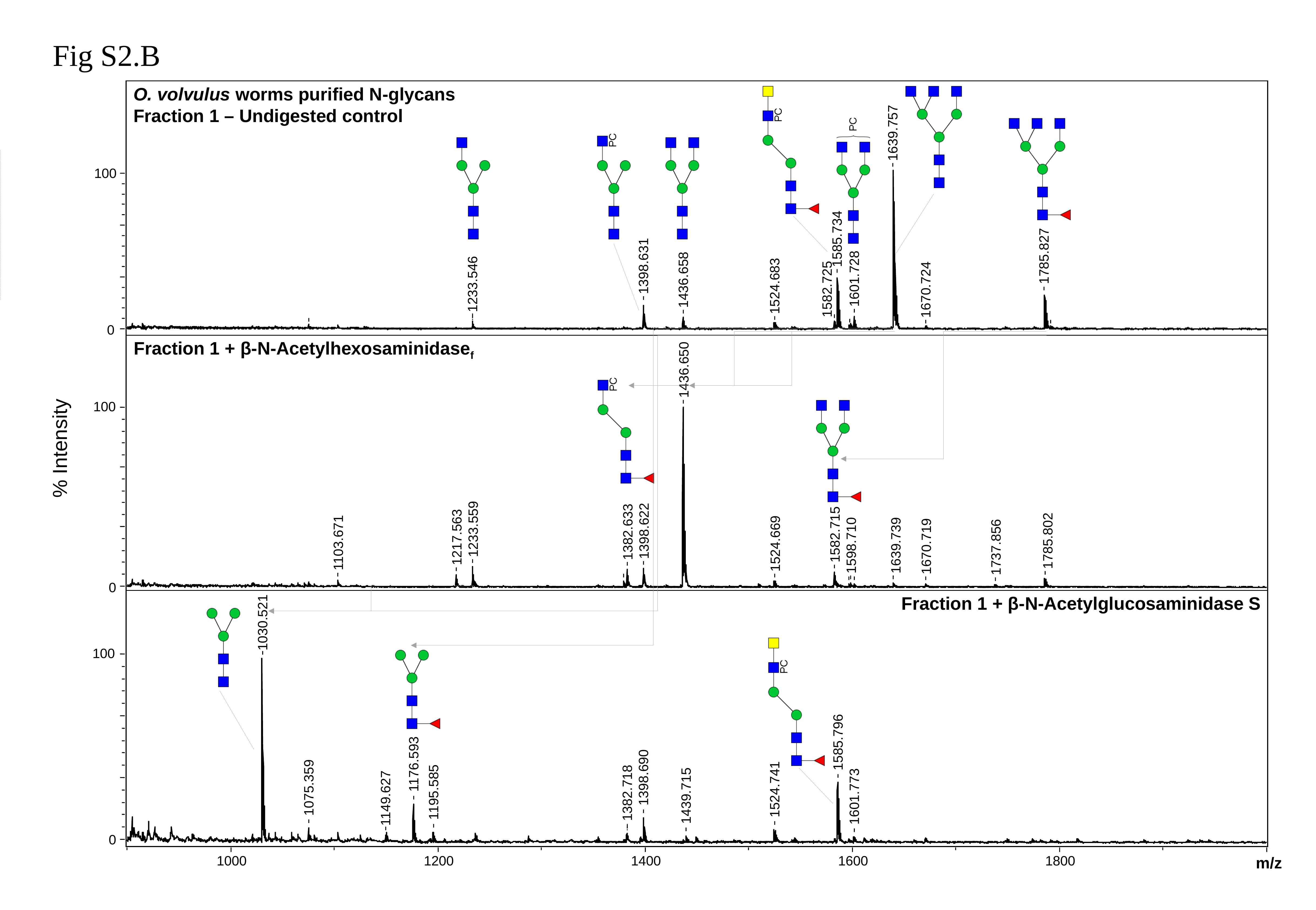

Fig S2.B
O. volvulus worms purified N-glycans
Fraction 1 – Undigested control
1639.757
100
0
1585.734
1785.827
1398.631
1601.728
1436.658
1233.546
1524.683
1582.725
1670.724
Fraction 1 + β-N-Acetylhexosaminidasef
1436.650
100
0
% Intensity
1233.559
1398.622
1382.633
1582.715
1217.563
1785.802
1103.671
1524.669
1598.710
1639.739
1670.719
1737.856
Fraction 1 + β-N-Acetylglucosaminidase S
1030.521
100
0
1585.796
1176.593
1398.690
1075.359
1524.741
1195.585
1382.718
1439.715
1601.773
1149.627
1000
1200
1400
1600
1800
m/z

## Slide 6
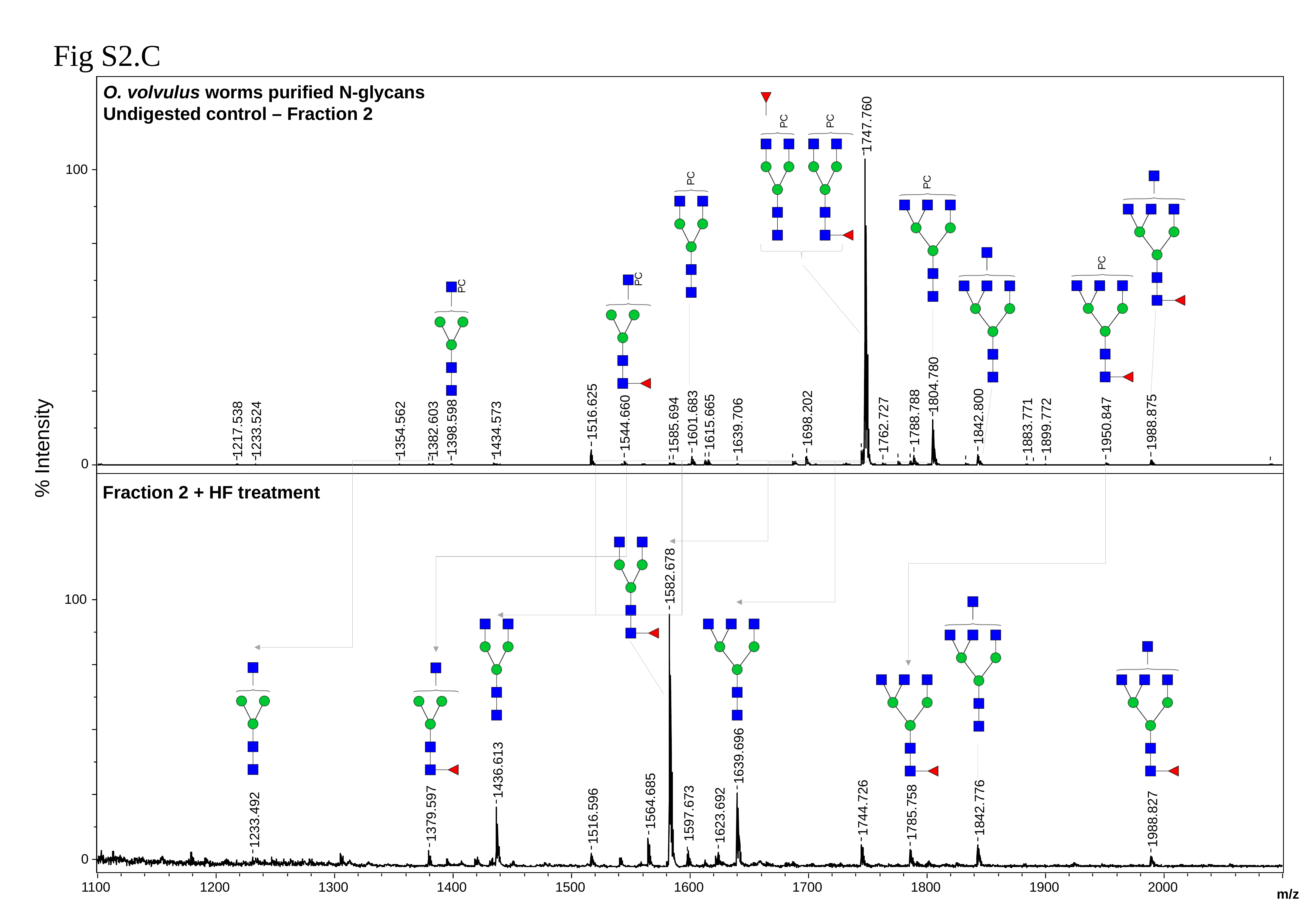

Fig S2.C
O. volvulus worms purified N-glycans
Undigested control – Fraction 2
1747.760
100
1804.780
1516.625
1842.800
1788.788
1698.202
1601.683
1988.875
1615.665
1544.660
1762.727
1950.847
1585.694
1883.771
1899.772
1639.706
1398.598
1434.573
1217.538
1233.524
1354.562
1382.603
% Intensity
0
Fraction 2 + HF treatment
1582.678
100
1639.696
1436.613
1564.685
1744.726
1842.776
1785.758
1597.673
1379.597
1623.692
1516.596
1988.827
1233.492
0
1100
1200
1300
1400
1500
1600
1700
1800
1900
2000
m/z

## Slide 7
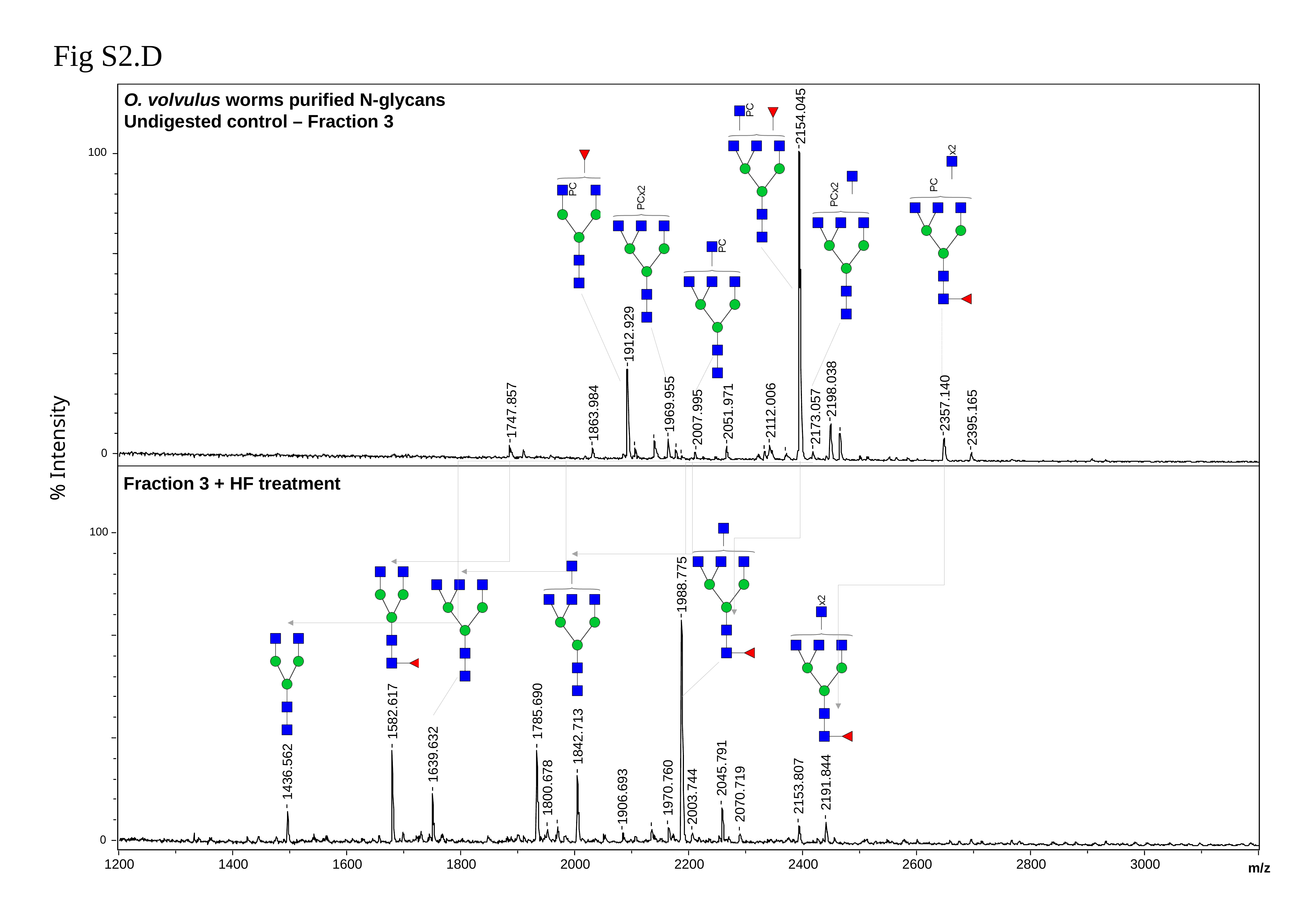

Fig S2.D
O. volvulus worms purified N-glycans
Undigested control – Fraction 3
2154.045
100
0
1912.929
2198.038
2357.140
1969.955
1747.857
2112.006
2051.971
1863.984
2173.057
2007.995
2395.165
% Intensity
Fraction 3 + HF treatment
100
0
1988.775
1785.690
1582.617
1842.713
1639.632
2045.791
1436.562
2191.844
2153.807
1800.678
1970.760
2070.719
2003.744
1906.693
1200
1400
1600
1800
2000
2200
2400
2600
2800
3000
m/z

## Slide 8
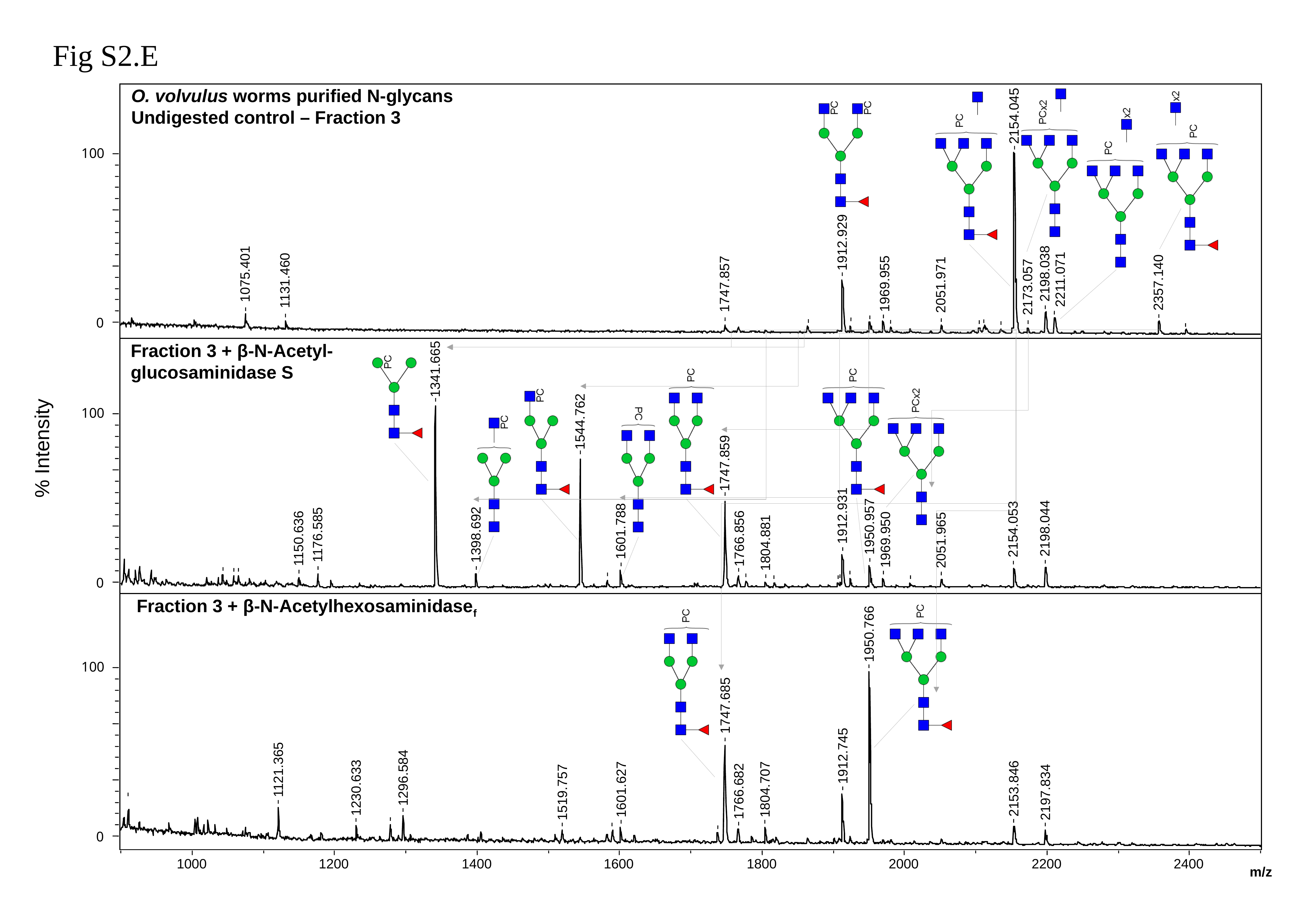

Fig S2.E
O. volvulus worms purified N-glycans
Undigested control – Fraction 3
2154.045
100
0
1912.929
2198.038
1075.401
2211.071
1131.460
2357.140
1969.955
1747.857
2051.971
2173.057
Fraction 3 + β-N-Acetyl-
glucosaminidase S
1341.665
100
0
1544.762
% Intensity
1747.859
1912.931
1950.957
2198.044
2154.053
1601.788
1176.585
1398.692
1150.636
1766.856
1969.950
2051.965
1804.881
Fraction 3 + β-N-Acetylhexosaminidasef
1950.766
100
0
1747.685
1912.745
1121.365
1296.584
1230.633
2153.846
1601.627
1804.707
1766.682
1519.757
2197.834
1000
1200
1400
1600
1800
2000
2200
2400
m/z

## Slide 9
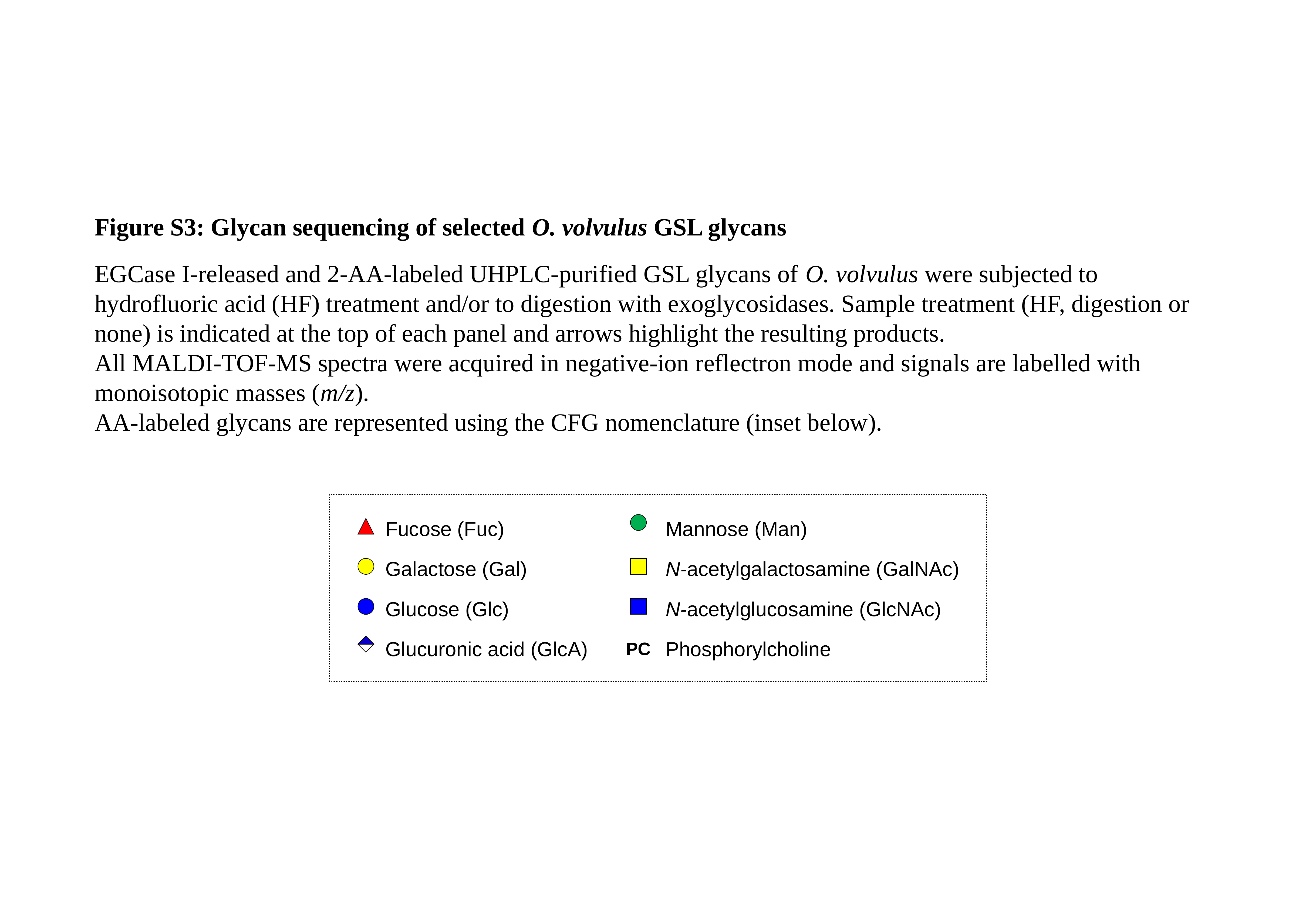

Figure S3: Glycan sequencing of selected O. volvulus GSL glycans
EGCase I-released and 2-AA-labeled UHPLC-purified GSL glycans of O. volvulus were subjected to hydrofluoric acid (HF) treatment and/or to digestion with exoglycosidases. Sample treatment (HF, digestion or none) is indicated at the top of each panel and arrows highlight the resulting products.
All MALDI-TOF-MS spectra were acquired in negative-ion reflectron mode and signals are labelled with monoisotopic masses (m/z).
AA-labeled glycans are represented using the CFG nomenclature (inset below).
Fucose (Fuc)
Mannose (Man)
Galactose (Gal)
N-acetylgalactosamine (GalNAc)
Glucose (Glc)
N-acetylglucosamine (GlcNAc)
Glucuronic acid (GlcA)
Phosphorylcholine
PC

## Slide 10
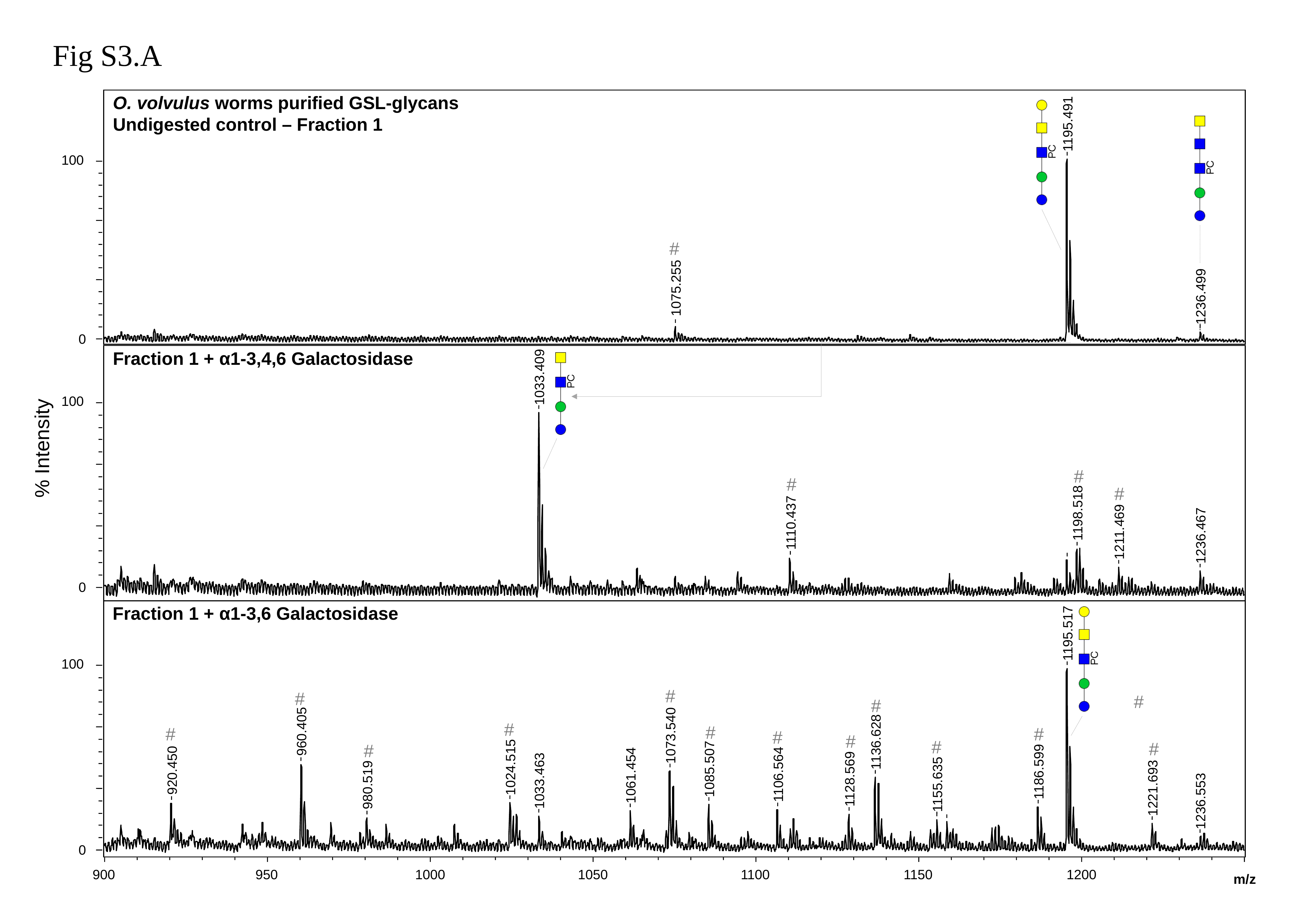

Fig S3.A
O. volvulus worms purified GSL-glycans
Undigested control – Fraction 1
1195.491
100
0
#
1075.255
1236.499
Fraction 1 + α1-3,4,6 Galactosidase
1033.409
100
0
% Intensity
#
#
#
1198.518
1110.437
1211.469
1236.467
Fraction 1 + α1-3,6 Galactosidase
1195.517
100
0
#
#
#
#
#
#
#
#
960.405
#
1073.540
#
#
1136.628
#
#
1024.515
1085.507
920.450
1186.599
1106.564
1061.454
1128.569
1033.463
1155.635
980.519
1221.693
1236.553
900
950
1000
1050
1100
1150
1200
m/z

## Slide 11
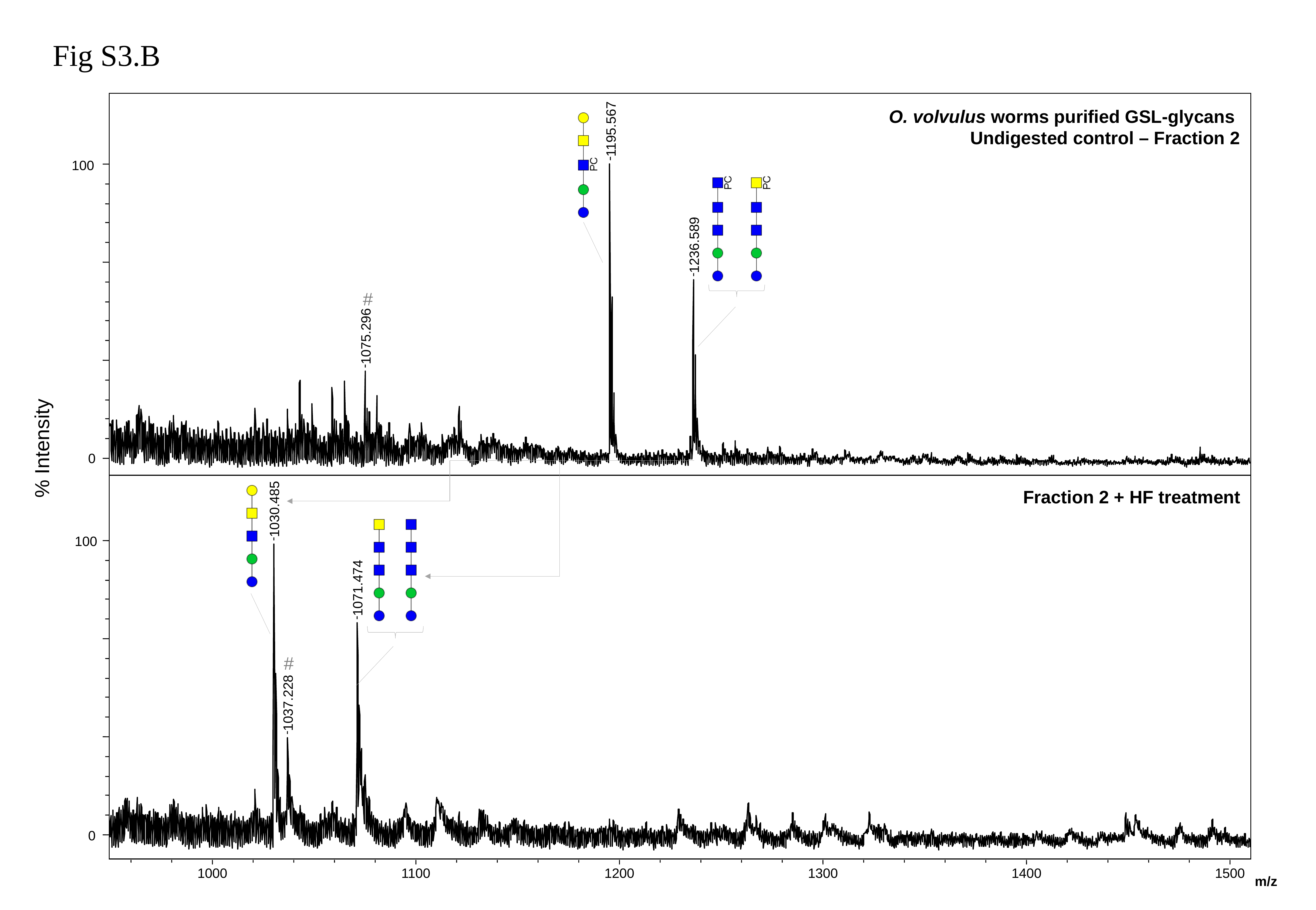

Fig S3.B
O. volvulus worms purified GSL-glycans
Undigested control – Fraction 2
1195.567
100
0
1236.589
#
1075.296
% Intensity
Fraction 2 + HF treatment
1030.485
100
0
1071.474
#
1037.228
1000
1100
1200
1300
1400
1500
m/z

## Slide 12
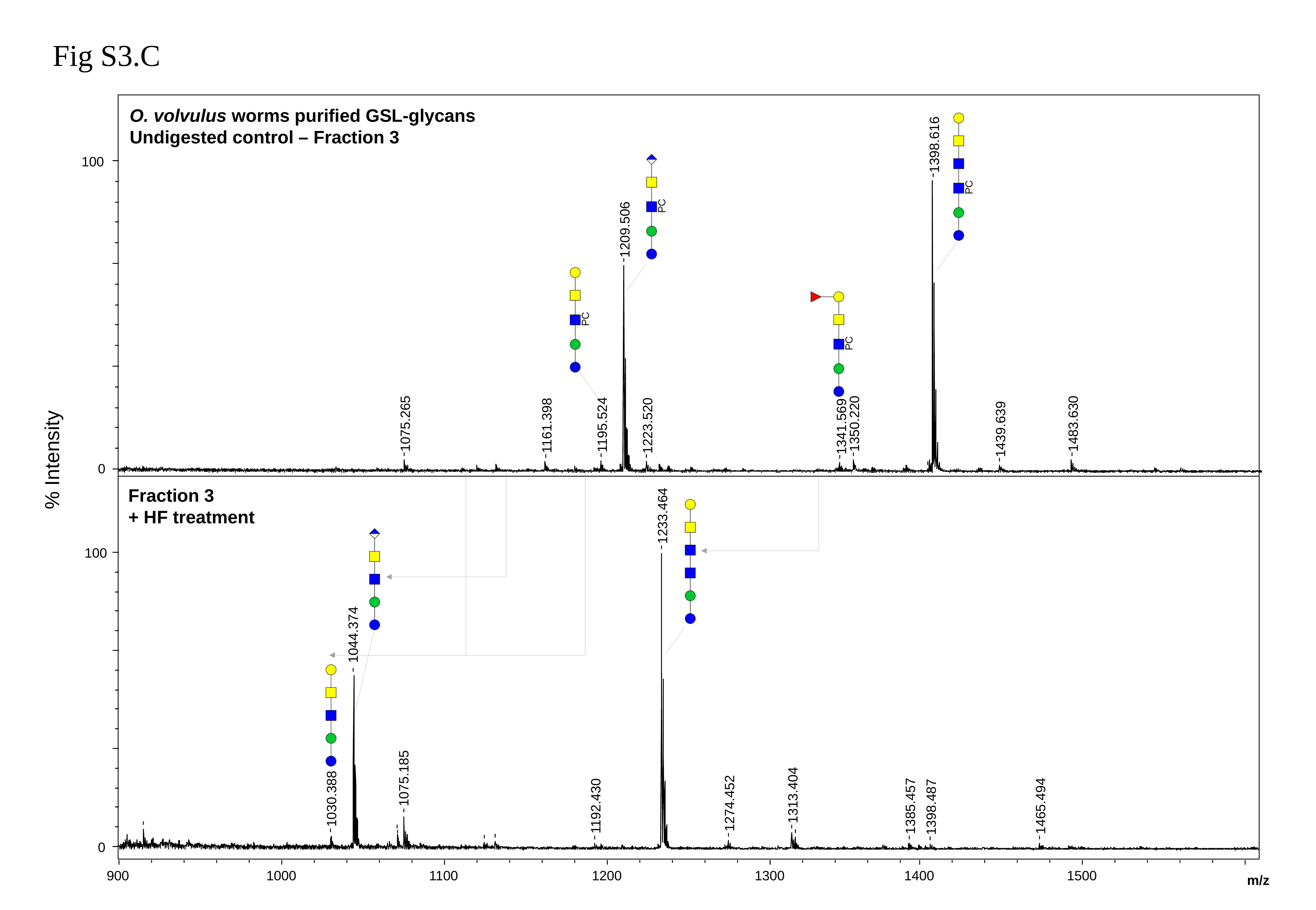

Fig S3.C
O. volvulus worms purified GSL-glycans
Undigested control – Fraction 3
1398.616
100
0
1209.506
1075.265
1350.220
1483.630
1195.524
1161.398
1223.520
1341.569
1439.639
% Intensity
Fraction 3
+ HF treatment
1233.464
100
0
1044.374
1075.185
1313.404
1030.388
1274.452
1192.430
1385.457
1465.494
1398.487
900
1000
1100
1200
1300
1400
1500
m/z

## Slide 13
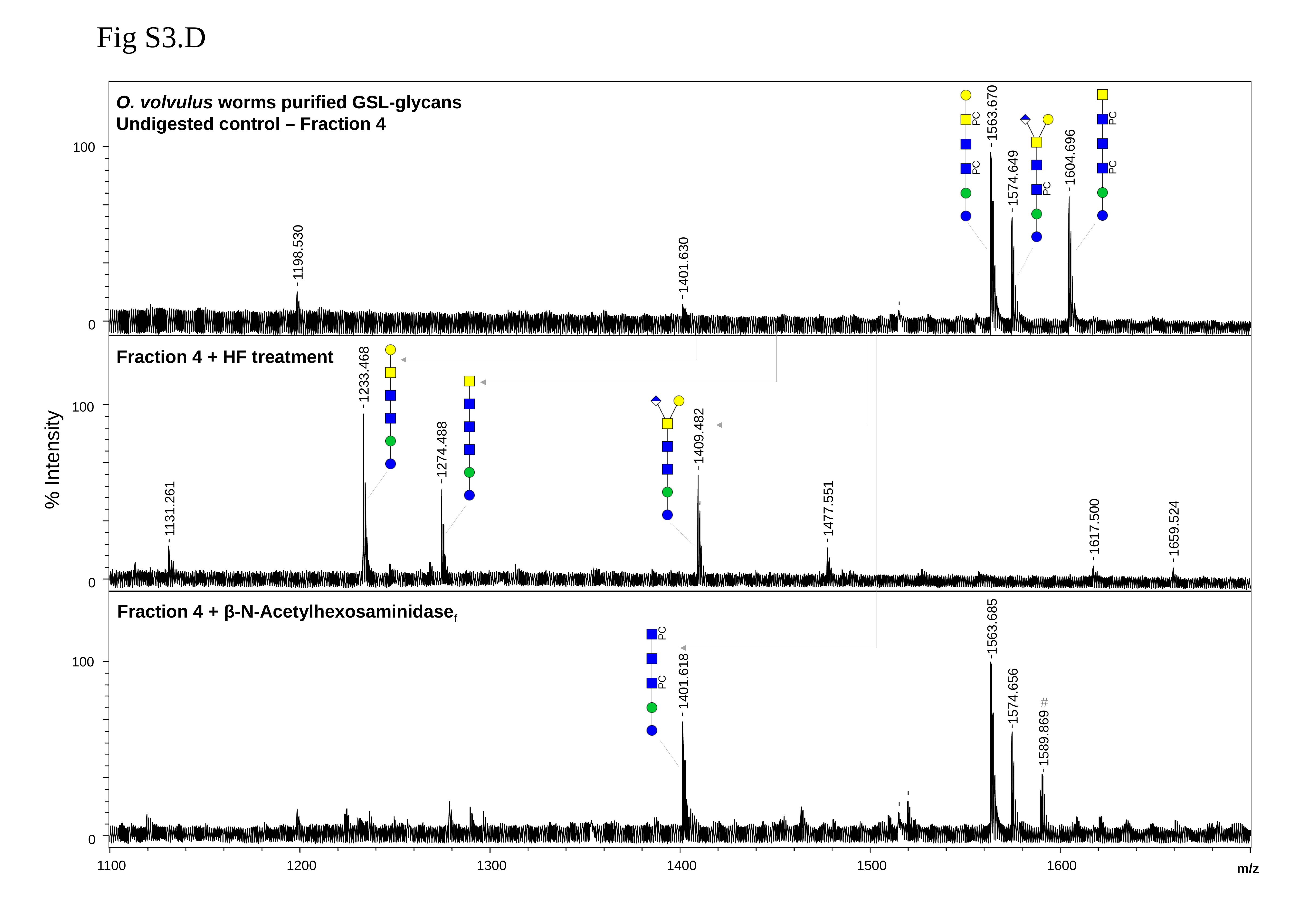

Fig S3.D
O. volvulus worms purified GSL-glycans
Undigested control – Fraction 4
1563.670
100
0
1604.696
1574.649
1198.530
1401.630
Fraction 4 + HF treatment
1233.468
100
0
1409.482
1274.488
% Intensity
1477.551
1131.261
1617.500
1659.524
Fraction 4 + β-N-Acetylhexosaminidasef
1563.685
100
0
1401.618
1574.656
#
1589.869
1100
1200
1300
1400
1500
1600
m/z

## Slide 14
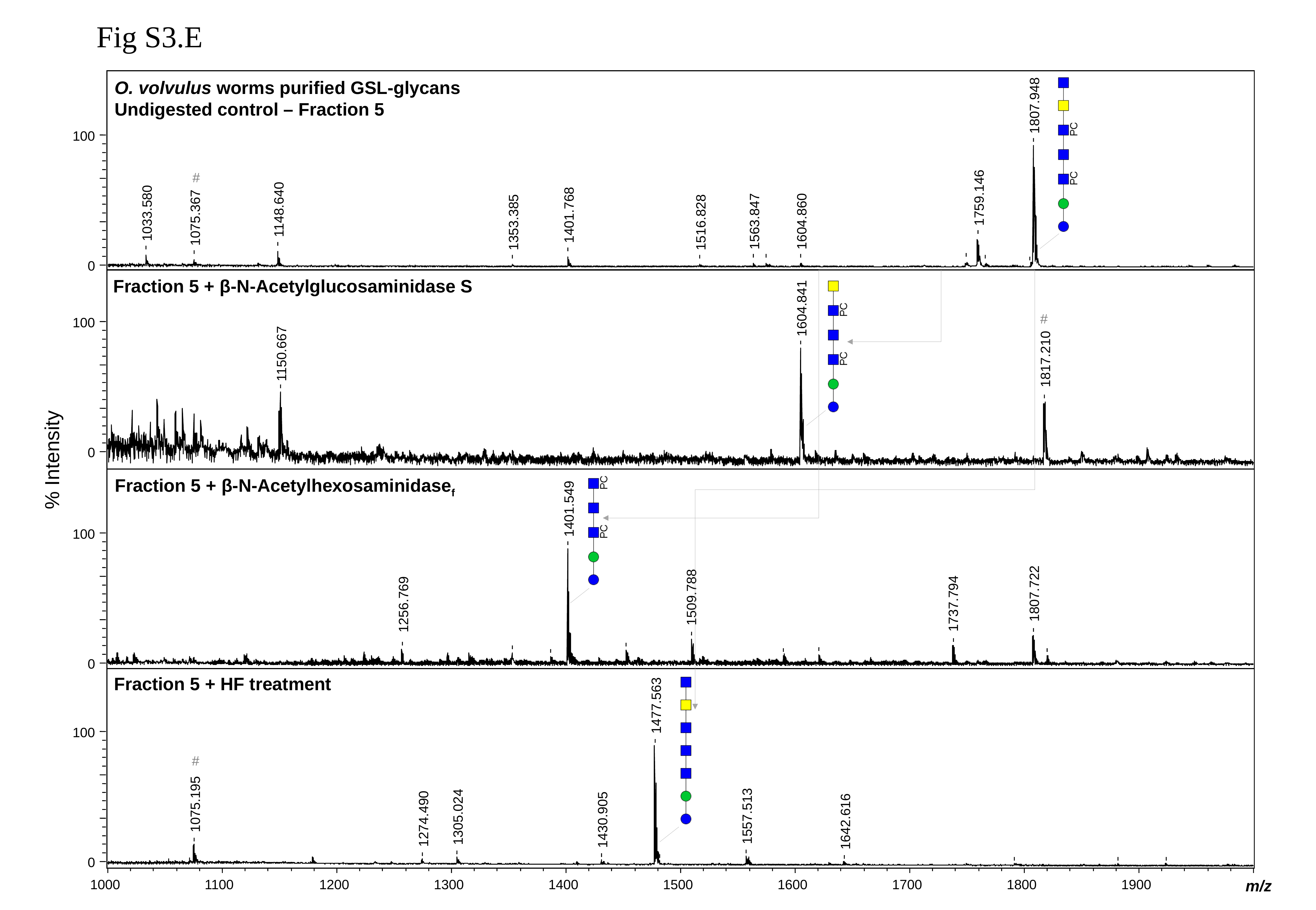

Fig S3.E
O. volvulus worms purified GSL-glycans
Undigested control – Fraction 5
1807.948
100
0
#
1759.146
1148.640
1033.580
1401.768
1075.367
1563.847
1604.860
1353.385
1516.828
Fraction 5 + β-N-Acetylglucosaminidase S
1604.841
#
100
0
1150.667
1817.210
% Intensity
Fraction 5 + β-N-Acetylhexosaminidasef
1401.549
100
0
1807.722
1509.788
1737.794
1256.769
Fraction 5 + HF treatment
1477.563
100
0
#
1075.195
1557.513
1305.024
1274.490
1430.905
1642.616
m/z
1000
1100
1200
1300
1400
1500
1600
1700
1800
1900

## Slide 15
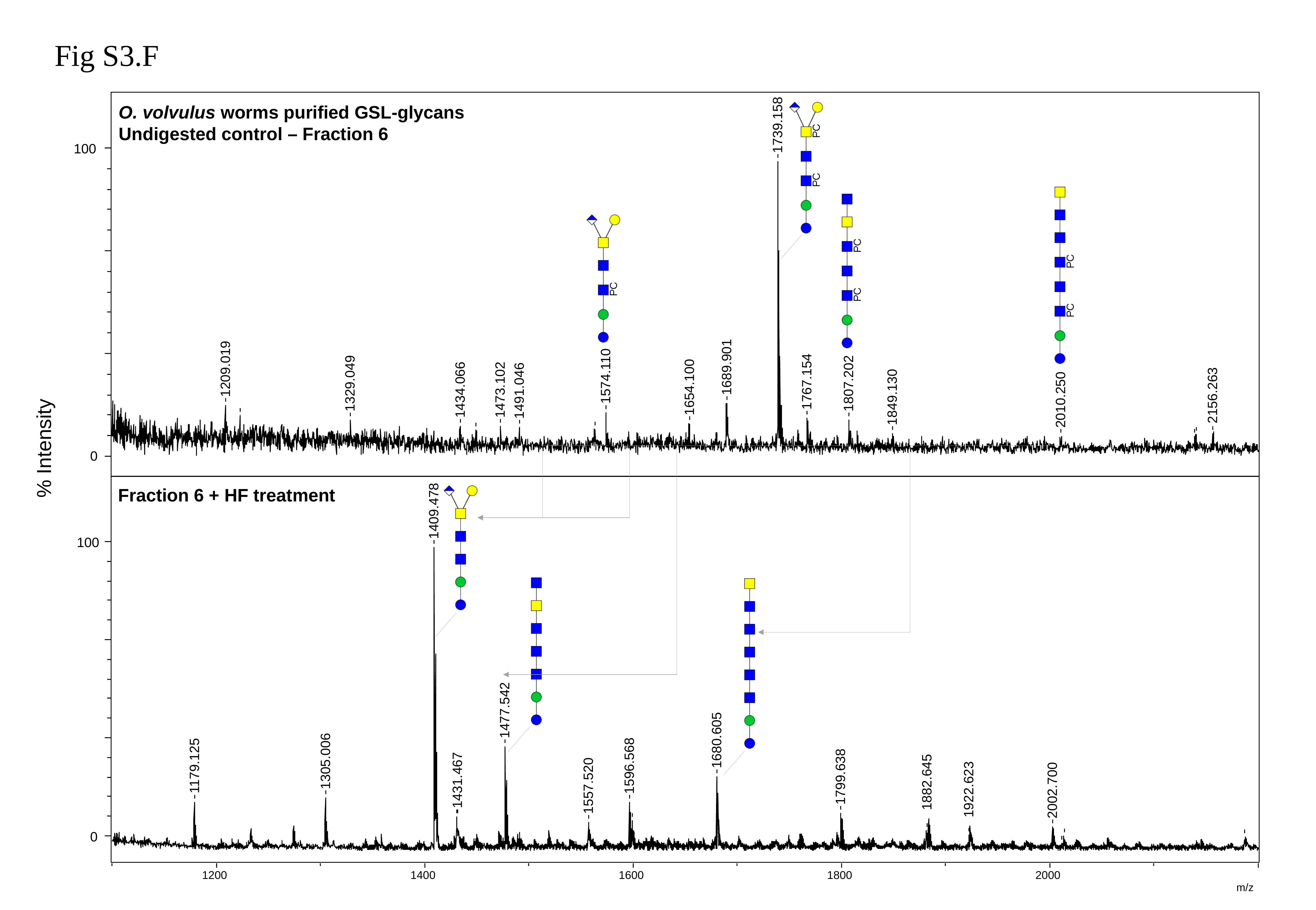

Fig S3.F
O. volvulus worms purified GSL-glycans
Undigested control – Fraction 6
1739.158
100
0
1689.901
1209.019
1574.110
1767.154
1329.049
1807.202
1654.100
1434.066
1473.102
1491.046
2156.263
1849.130
2010.250
% Intensity
Fraction 6 + HF treatment
1409.478
100
0
1477.542
1680.605
1305.006
1596.568
1179.125
1799.638
1431.467
1882.645
1557.520
1922.623
2002.700
1200
1400
1600
1800
2000
m/z

## Slide 16
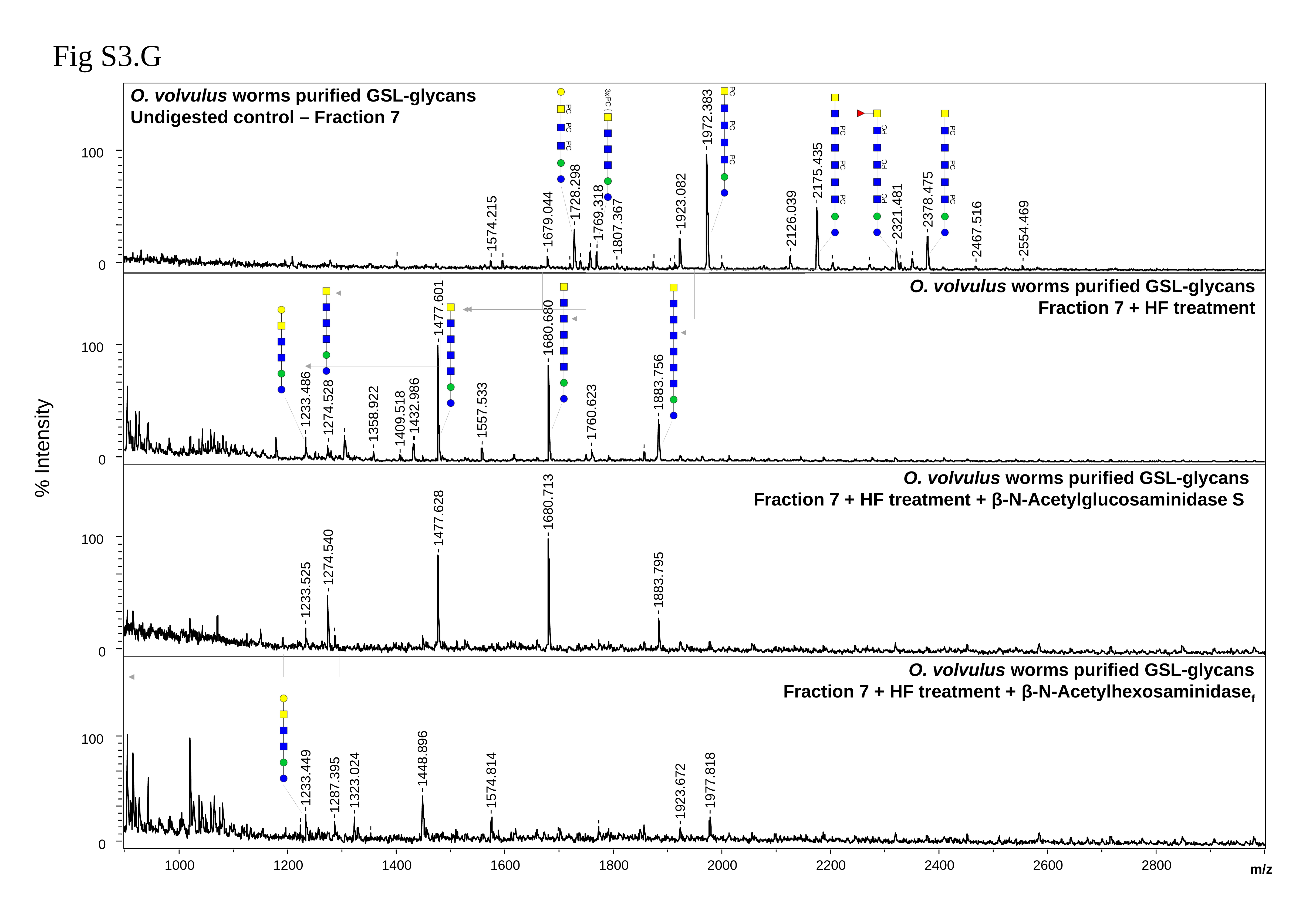

Fig S3.G
O. volvulus worms purified GSL-glycans
Undigested control – Fraction 7
1972.383
100
0
2175.435
1728.298
2378.475
1923.082
2321.481
1769.318
2126.039
1679.044
1574.215
1807.367
2554.469
2467.516
O. volvulus worms purified GSL-glycans
Fraction 7 + HF treatment
1477.601
1680.680
100
0
1883.756
1233.486
1432.986
1274.528
1557.533
1760.623
1358.922
1409.518
% Intensity
O. volvulus worms purified GSL-glycans
Fraction 7 + HF treatment + β-N-Acetylglucosaminidase S
1680.713
1477.628
100
0
1274.540
1883.795
1233.525
O. volvulus worms purified GSL-glycans
Fraction 7 + HF treatment + β-N-Acetylhexosaminidasef
100
0
1448.896
1233.449
1574.814
1977.818
1323.024
1287.395
1923.672
1000
1200
1400
1600
1800
2000
2200
2400
2600
2800
m/z

## Slide 17
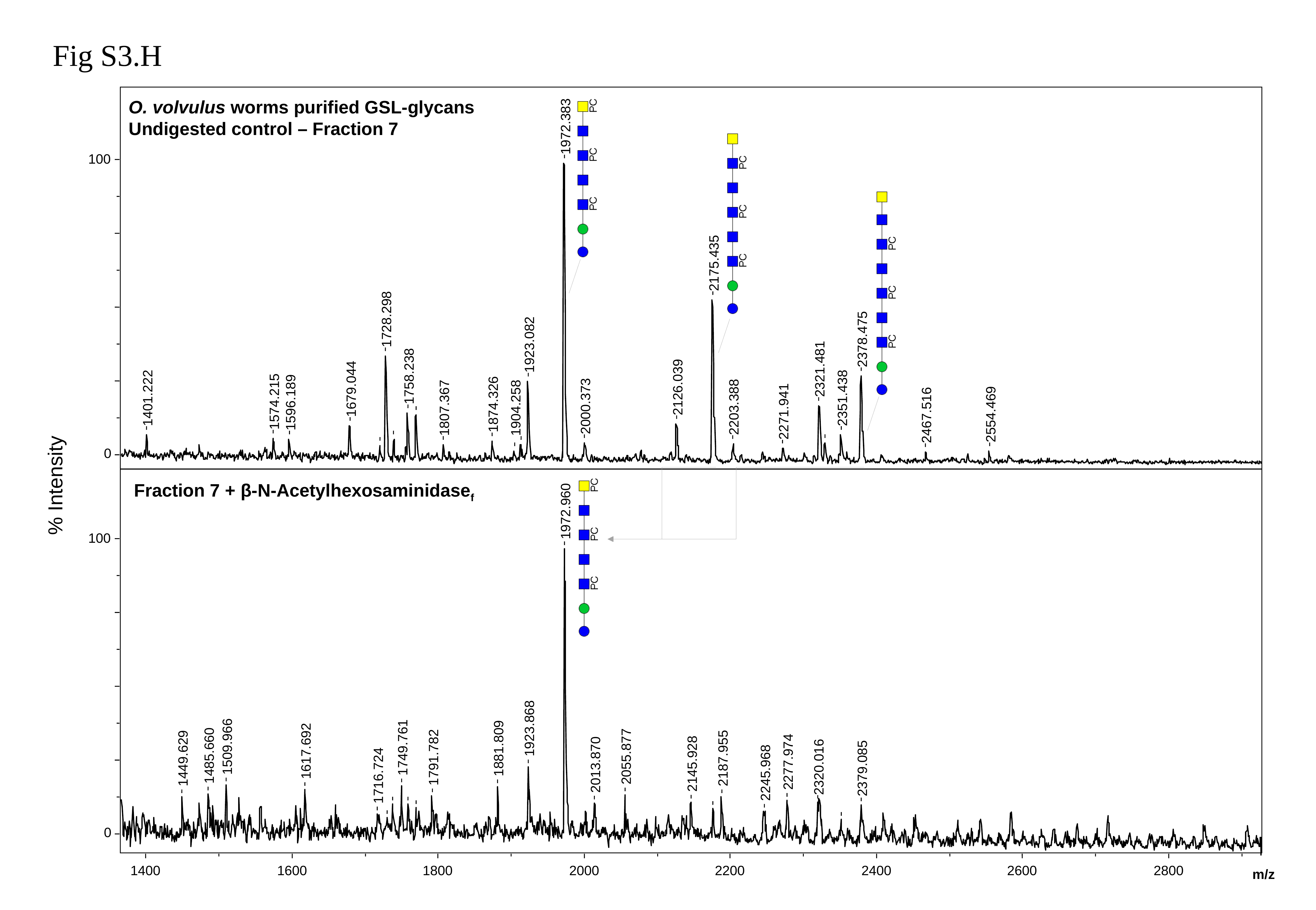

Fig S3.H
O. volvulus worms purified GSL-glycans
Undigested control – Fraction 7
1972.383
100
0
2175.435
1728.298
2378.475
1923.082
2321.481
1758.238
2126.039
1679.044
2351.438
1401.222
1574.215
1596.189
1874.326
2000.373
2203.388
1807.367
1904.258
2271.941
2554.469
2467.516
% Intensity
Fraction 7 + β-N-Acetylhexosaminidasef
1972.960
100
0
1923.868
1509.966
1749.761
1881.809
1617.692
1485.660
2055.877
1791.782
2187.955
1449.629
2277.974
2145.928
2013.870
2320.016
2379.085
2245.968
1716.724
1400
1600
1800
2000
2200
2400
2600
2800
m/z

## Slide 18
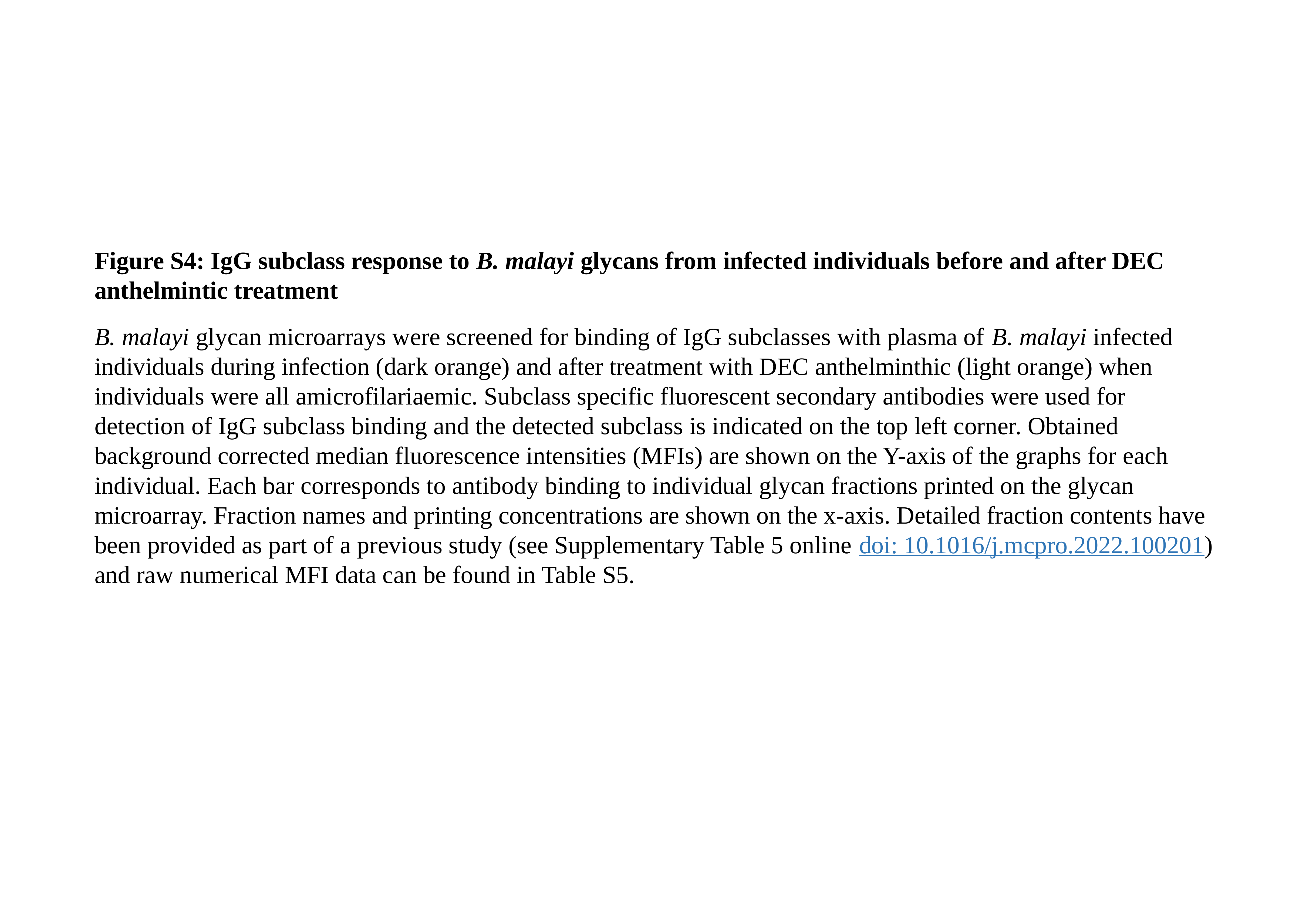

Figure S4: IgG subclass response to B. malayi glycans from infected individuals before and after DEC anthelmintic treatment
B. malayi glycan microarrays were screened for binding of IgG subclasses with plasma of B. malayi infected individuals during infection (dark orange) and after treatment with DEC anthelminthic (light orange) when individuals were all amicrofilariaemic. Subclass specific fluorescent secondary antibodies were used for detection of IgG subclass binding and the detected subclass is indicated on the top left corner. Obtained background corrected median fluorescence intensities (MFIs) are shown on the Y-axis of the graphs for each individual. Each bar corresponds to antibody binding to individual glycan fractions printed on the glycan microarray. Fraction names and printing concentrations are shown on the x-axis. Detailed fraction contents have been provided as part of a previous study (see Supplementary Table 5 online doi: 10.1016/j.mcpro.2022.100201) and raw numerical MFI data can be found in Table S5.

## Slide 19
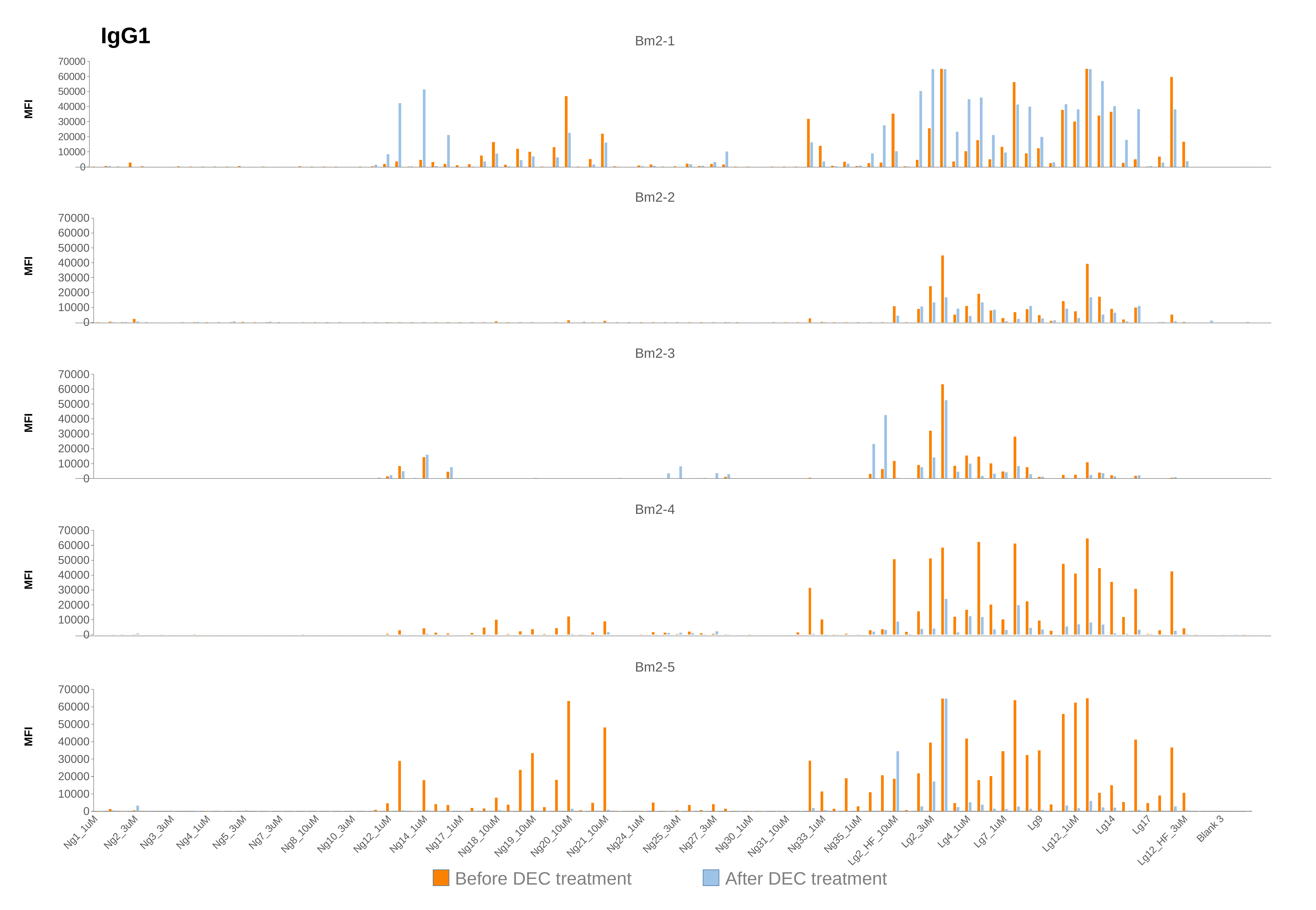

IgG1
### Chart: Bm2-1
| Category | AM 103 (90) IgG1 | AM 103 (92) IgG1 |
|---|---|---|
| Ng1_1uM
 | 197.33333333333331 | 0.0 |
| Ng1_3uM
 | 502.0 | 455.00000000000006 |
| Ng2_1uM | 107.0 | 0.0 |
| Ng2_3uM | 2731.3333333333335 | 6.333333333333371 |
| Ng2_10uM | 300.0 | 0.0 |
| Ng3_1uM | 0.0 | 0.0 |
| Ng3_3uM | 2.0 | 0.0 |
| Ng3_10uM | 325.3333333333333 | 0.0 |
| Ng3_30uM | 80.0 | 0.0 |
| Ng4_1uM | 104.0 | 0.0 |
| Ng4_3uM | 126.0 | 0.0 |
| Ng5_1uM | 179.66666666666669 | 0.0 |
| Ng5_3uM | 466.66666666666663 | 5.0 |
| Ng6_1uM | 22.333333333333343 | 0.0 |
| Ng7_1uM | 48.0 | 0.0 |
| Ng7_3uM | 19.666666666666657 | 0.0 |
| Ng8_1uM | 1.0 | 0.0 |
| Ng8_3uM | 236.33333333333331 | 0.0 |
| Ng8_10uM | 144.0 | 0.0 |
| Ng9_1uM | 49.33333333333334 | 0.0 |
| Ng9_3uM | 158.33333333333331 | 0.0 |
| Ng10_3uM | 5.666666666666657 | 0.0 |
| Ng10_1uM | 152.0 | 0.0 |
| Ng11 | 390.0 | 1391.0 |
| Ng12_1uM | 1870.3333333333333 | 8375.0 |
| Ng12_3uM | 3594.3333333333335 | 42229.33333333333 |
| Ng13_1uM | 190.33333333333331 | 338.00000000000006 |
| Ng14_1uM | 4683.0 | 51406.33333333333 |
| Ng15_1uM | 3094.3333333333335 | 545.0 |
| Ng16_1uM | 2009.6666666666665 | 21008.666666666668 |
| Ng17_1uM | 1039.0 | 0.0 |
| Ng18_1uM | 1747.6666666666667 | 161.00000000000006 |
| Ng18_3uM | 7453.0 | 3563.333333333333 |
| Ng18_10uM | 16400.0 | 8697.666666666666 |
| Ng19_1uM | 1307.0 | 256.00000000000006 |
| Ng19_3uM | 11957.0 | 4513.333333333334 |
| Ng19_10uM | 9963.333333333334 | 6921.333333333334 |
| Ng20_1uM | 162.33333333333331 | 0.0 |
| Ng20_3uM | 13102.0 | 6150.0 |
| Ng20_10uM | 46947.0 | 22610.333333333336 |
| Ng21_1uM | 206.0 | 0.0 |
| Ng21 _3uM | 5067.666666666667 | 1520.0 |
| Ng21_10uM | 21971.666666666668 | 15990.0 |
| Ng22_1uM | 375.66666666666663 | 189.33333333333331 |
| Ng23_1uM | 0.0 | 0.0 |
| Ng24_1uM | 884.3333333333333 | 225.33333333333331 |
| Ng24_3uM | 1554.6666666666667 | 737.3333333333335 |
| Ng25_1uM | 193.0 | 0.0 |
| Ng25_3uM | 339.6666666666667 | 27.666666666666686 |
| Ng26_1uM | 2154.0 | 1740.0000000000002 |
| Ng27_1uM | 403.66666666666663 | 678.3333333333335 |
| Ng27_3uM | 1902.6666666666665 | 3256.333333333333 |
| Ng28_1uM | 1651.6666666666667 | 10162.0 |
| Ng29_1uM | 42.33333333333334 | 0.0 |
| Ng30_1uM | 187.33333333333331 | 0.0 |
| Ng31_1uM | 0.0 | 0.0 |
| Ng31_3uM | 68.0 | 0.0 |
| Ng31_10uM | 180.0 | 0.0 |
| Ng31_30uM | 203.0 | 0.0 |
| Ng32_1uM | 31817.333333333332 | 16205.666666666666 |
| Ng33_1uM | 13847.333333333334 | 3521.0 |
| Ng34_1uM | 610.3333333333334 | 290.00000000000006 |
| Ng34_3uM | 3427.0 | 2129.0 |
| Ng35_1uM | 516.6666666666666 | 907.3333333333335 |
| Ng36_1uM | 2467.3333333333335 | 8977.666666666666 |
| Ng36_3uM | 2876.6666666666665 | 27611.333333333336 |
| Lg2_HF_10uM | 35202.666666666664 | 10265.666666666666 |
| Lg1_1uM | 243.66666666666669 | 67.33333333333337 |
| Lg2_1uM | 4651.666666666667 | 50220.33333333333 |
| Lg2_3uM | 25519.333333333332 | 64785.666666666664 |
| Lg2_10uM | 64979.0 | 64772.666666666664 |
| Lg3_1uM | 3494.6666666666665 | 23175.333333333336 |
| Lg4_1uM | 10351.0 | 44976.0 |
| Lg5_3uM | 17727.666666666668 | 45921.33333333333 |
| Lg6_1uM | 5014.0 | 21059.333333333336 |
| Lg7_1uM | 13233.0 | 9474.0 |
| Lg7_3uM | 56296.0 | 41288.0 |
| Lg8_1uM | 8949.333333333334 | 39876.0 |
| Lg9 | 12298.666666666666 | 19773.666666666668 |
| Lg10_1uM | 2501.6666666666665 | 3027.6666666666665 |
| Lg11_1uM | 37693.333333333336 | 41439.666666666664 |
| Lg12_1uM | 30116.333333333332 | 38145.666666666664 |
| Lg12_3uM | 64990.333333333336 | 64781.666666666664 |
| Lg13 | 34074.666666666664 | 56954.0 |
| Lg14 | 36568.0 | 40199.666666666664 |
| Lg15 | 2551.6666666666665 | 17832.333333333336 |
| Lg16 | 4981.0 | 38264.33333333333 |
| Lg17 | 75.66666666666666 | 581.0 |
| Lg18_HF_1uM | 6683.0 | 2894.333333333333 |
| Lg7_HF_3uM | 59583.666666666664 | 38149.0 |
| Lg12_HF_3uM | 16617.333333333332 | 3783.3333333333335 |
| Blank 1 | 11.333333333333343 | 0.0 |
| Blank 2 | 0.0 | 0.0 |
| Blank 3 | 0.0 | 0.0 |
| Blank 4 | 0.0 | 0.0 |
| Blank 5 | 18.666666666666657 | 0.0 |MFI
### Chart: Bm2-2
| Category | AM 113 (90) IgG1 | AM113 (92) IgG1 |
|---|---|---|
| Ng1_1uM
 | 74.33333333333334 | 0.0 |
| Ng1_3uM
 | 442.33333333333337 | 81.0 |
| Ng2_1uM | 24.333333333333343 | 379.66666666666663 |
| Ng2_3uM | 2273.0 | 753.0000000000001 |
| Ng2_10uM | 63.0 | 0.0 |
| Ng3_1uM | 0.0 | 0.0 |
| Ng3_3uM | 0.0 | 0.0 |
| Ng3_10uM | 58.0 | 0.0 |
| Ng3_30uM | 16.333333333333343 | 391.0000000000001 |
| Ng4_1uM | 30.333333333333343 | 0.0 |
| Ng4_3uM | 116.33333333333331 | 0.0 |
| Ng5_1uM | 48.33333333333334 | 578.3333333333334 |
| Ng5_3uM | 241.0 | 0.0 |
| Ng6_1uM | 52.0 | 0.0 |
| Ng7_1uM | 17.0 | 538.3333333333334 |
| Ng7_3uM | 9.0 | 0.0 |
| Ng8_1uM | 0.0 | 0.0 |
| Ng8_3uM | 78.33333333333334 | 0.0 |
| Ng8_10uM | 50.0 | 0.0 |
| Ng9_1uM | 15.666666666666657 | 0.0 |
| Ng9_3uM | 80.0 | 0.0 |
| Ng10_3uM | 0.0 | 0.0 |
| Ng10_1uM | 74.33333333333334 | 0.0 |
| Ng11 | 74.0 | 0.0 |
| Ng12_1uM | 2.666666666666657 | 107.66666666666674 |
| Ng12_3uM | 97.0 | 0.0 |
| Ng13_1uM | 7.333333333333343 | 0.0 |
| Ng14_1uM | 157.0 | 0.0 |
| Ng15_1uM | 106.0 | 0.0 |
| Ng16_1uM | 35.0 | 0.0 |
| Ng17_1uM | 28.333333333333343 | 0.0 |
| Ng18_1uM | 28.666666666666657 | 0.0 |
| Ng18_3uM | 159.0 | 0.0 |
| Ng18_10uM | 584.0 | 0.0 |
| Ng19_1uM | 0.6666666666666572 | 0.0 |
| Ng19_3uM | 215.33333333333331 | 0.0 |
| Ng19_10uM | 86.0 | 0.0 |
| Ng20_1uM | 0.0 | 0.0 |
| Ng20_3uM | 221.33333333333331 | 0.0 |
| Ng20_10uM | 1424.0 | 0.0 |
| Ng21_1uM | 0.0 | 408.66666666666663 |
| Ng21 _3uM | 44.33333333333334 | 0.0 |
| Ng21_10uM | 1022.6666666666667 | 0.0 |
| Ng22_1uM | 105.0 | 0.0 |
| Ng23_1uM | 6.666666666666657 | 0.0 |
| Ng24_1uM | 25.333333333333343 | 0.0 |
| Ng24_3uM | 93.0 | 0.0 |
| Ng25_1uM | 87.33333333333334 | 0.0 |
| Ng25_3uM | 53.33333333333334 | 0.0 |
| Ng26_1uM | 134.0 | 0.0 |
| Ng27_1uM | 27.666666666666657 | 0.0 |
| Ng27_3uM | 181.0 | 0.0 |
| Ng28_1uM | 9.333333333333343 | 132.0 |
| Ng29_1uM | 15.666666666666657 | 0.0 |
| Ng30_1uM | 0.0 | 0.0 |
| Ng31_1uM | 0.0 | 0.0 |
| Ng31_3uM | 77.66666666666666 | 0.0 |
| Ng31_10uM | 95.0 | 0.0 |
| Ng31_30uM | 107.66666666666669 | 0.0 |
| Ng32_1uM | 2590.0 | 0.0 |
| Ng33_1uM | 261.0 | 0.6666666666667425 |
| Ng34_1uM | 11.0 | 0.0 |
| Ng34_3uM | 98.33333333333334 | 0.0 |
| Ng35_1uM | 28.333333333333343 | 0.0 |
| Ng36_1uM | 156.33333333333331 | 0.0 |
| Ng36_3uM | 209.0 | 0.0 |
| Lg2_HF_10uM | 10833.333333333334 | 4401.333333333333 |
| Lg1_1uM | 67.66666666666666 | 0.0 |
| Lg2_1uM | 9038.666666666666 | 10678.666666666668 |
| Lg2_3uM | 24177.0 | 13355.333333333334 |
| Lg2_10uM | 44832.666666666664 | 16746.0 |
| Lg3_1uM | 5265.333333333333 | 9281.333333333334 |
| Lg4_1uM | 11084.333333333334 | 4304.0 |
| Lg5_3uM | 19092.0 | 13302.666666666668 |
| Lg6_1uM | 7917.333333333333 | 8400.666666666668 |
| Lg7_1uM | 2870.3333333333335 | 784.0000000000001 |
| Lg7_3uM | 6760.0 | 2397.666666666667 |
| Lg8_1uM | 8737.0 | 11008.0 |
| Lg9 | 4904.0 | 2712.0 |
| Lg10_1uM | 1109.3333333333333 | 1361.3333333333335 |
| Lg11_1uM | 14195.0 | 9220.666666666668 |
| Lg12_1uM | 7445.333333333333 | 2860.8333333333335 |
| Lg12_3uM | 39324.666666666664 | 16743.0 |
| Lg13 | 17187.666666666668 | 5160.0 |
| Lg14 | 8955.333333333334 | 6438.666666666666 |
| Lg15 | 1927.6666666666665 | 675.3333333333334 |
| Lg16 | 9998.666666666666 | 10956.333333333334 |
| Lg17 | 0.0 | 0.0 |
| Lg18_HF_1uM | 169.33333333333331 | 347.66666666666663 |
| Lg7_HF_3uM | 5274.0 | 881.6666666666666 |
| Lg12_HF_3uM | 301.3333333333333 | 0.0 |
| Blank 1 | 0.0 | 0.0 |
| Blank 2 | 0.0 | 1273.3333333333335 |
| Blank 3 | 0.0 | 0.0 |
| Blank 4 | 0.0 | 0.0 |
| Blank 5 | 0.0 | 285.33333333333337 |MFI
### Chart: Bm2-3
| Category | AM134 (90) IgG1 | AM 134 (92) IgG1 |
|---|---|---|
| Ng1_1uM
 | 0.0 | 0.0 |
| Ng1_3uM
 | 0.0 | 109.66666666666669 |
| Ng2_1uM | 0.0 | 0.0 |
| Ng2_3uM | 0.0 | 12.0 |
| Ng2_10uM | 0.0 | 36.0 |
| Ng3_1uM | 0.0 | 0.0 |
| Ng3_3uM | 0.0 | 0.0 |
| Ng3_10uM | 0.0 | 29.666666666666657 |
| Ng3_30uM | 0.0 | 51.0 |
| Ng4_1uM | 0.0 | 3.333333333333343 |
| Ng4_3uM | 0.0 | 32.33333333333334 |
| Ng5_1uM | 0.0 | 0.0 |
| Ng5_3uM | 0.0 | 37.33333333333334 |
| Ng6_1uM | 0.0 | 51.33333333333334 |
| Ng7_1uM | 0.0 | 0.0 |
| Ng7_3uM | 0.0 | 56.66666666666666 |
| Ng8_1uM | 0.0 | 0.0 |
| Ng8_3uM | 0.0 | 51.0 |
| Ng8_10uM | 0.0 | 37.33333333333334 |
| Ng9_1uM | 0.0 | 3.0 |
| Ng9_3uM | 0.0 | 62.66666666666666 |
| Ng10_3uM | 0.0 | 0.0 |
| Ng10_1uM | 0.0 | 32.0 |
| Ng11 | 0.0 | 559.3333333333334 |
| Ng12_1uM | 1427.6666666666665 | 2231.3333333333335 |
| Ng12_3uM | 8324.666666666666 | 4918.0 |
| Ng13_1uM | 0.0 | 419.33333333333337 |
| Ng14_1uM | 14389.333333333334 | 16014.0 |
| Ng15_1uM | 0.0 | 0.0 |
| Ng16_1uM | 4546.666666666667 | 7694.333333333333 |
| Ng17_1uM | 0.0 | 0.0 |
| Ng18_1uM | 0.0 | 1.3333333333333428 |
| Ng18_3uM | 0.0 | 97.33333333333334 |
| Ng18_10uM | 0.0 | 84.0 |
| Ng19_1uM | 0.0 | 74.33333333333334 |
| Ng19_3uM | 0.0 | 149.33333333333331 |
| Ng19_10uM | 0.0 | 319.3333333333333 |
| Ng20_1uM | 0.0 | 0.0 |
| Ng20_3uM | 0.0 | 38.0 |
| Ng20_10uM | 0.0 | 142.0 |
| Ng21_1uM | 0.0 | 0.0 |
| Ng21 _3uM | 0.0 | 1.3333333333333428 |
| Ng21_10uM | 0.0 | 127.0 |
| Ng22_1uM | 0.0 | 361.0 |
| Ng23_1uM | 0.0 | 0.0 |
| Ng24_1uM | 0.0 | 53.66666666666666 |
| Ng24_3uM | 0.0 | 218.33333333333331 |
| Ng25_1uM | 0.0 | 3539.0 |
| Ng25_3uM | 0.0 | 8075.666666666666 |
| Ng26_1uM | 0.0 | 47.0 |
| Ng27_1uM | 0.0 | 423.33333333333337 |
| Ng27_3uM | 0.0 | 3640.3333333333335 |
| Ng28_1uM | 1103.6666666666665 | 2821.3333333333335 |
| Ng29_1uM | 0.0 | 94.0 |
| Ng30_1uM | 0.0 | 0.0 |
| Ng31_1uM | 0.0 | 0.0 |
| Ng31_3uM | 0.0 | 5.333333333333343 |
| Ng31_10uM | 0.0 | 108.33333333333331 |
| Ng31_30uM | 0.0 | 65.33333333333334 |
| Ng32_1uM | 479.0 | 21.0 |
| Ng33_1uM | 0.0 | 44.0 |
| Ng34_1uM | 0.0 | 1.0 |
| Ng34_3uM | 0.0 | 42.33333333333334 |
| Ng35_1uM | 0.0 | 0.0 |
| Ng36_1uM | 3098.666666666667 | 23271.666666666668 |
| Ng36_3uM | 6284.0 | 42611.0 |
| Lg2_HF_10uM | 11793.0 | 394.33333333333337 |
| Lg1_1uM | 199.33333333333326 | 50.0 |
| Lg2_1uM | 9072.666666666666 | 7605.333333333333 |
| Lg2_3uM | 32174.666666666664 | 14137.333333333334 |
| Lg2_10uM | 63306.0 | 52668.333333333336 |
| Lg3_1uM | 8573.333333333334 | 4495.666666666667 |
| Lg4_1uM | 15383.0 | 10004.333333333334 |
| Lg5_3uM | 14729.333333333334 | 1767.3333333333333 |
| Lg6_1uM | 10220.666666666666 | 3325.3333333333335 |
| Lg7_1uM | 4697.0 | 4223.666666666667 |
| Lg7_3uM | 28168.0 | 8371.666666666666 |
| Lg8_1uM | 7552.0 | 2989.6666666666665 |
| Lg9 | 1156.3333333333335 | 1351.3333333333333 |
| Lg10_1uM | 0.0 | 7.0 |
| Lg11_1uM | 2282.6666666666665 | 361.6666666666667 |
| Lg12_1uM | 2515.6666666666665 | 299.3333333333333 |
| Lg12_3uM | 10824.0 | 2348.3333333333335 |
| Lg13 | 3986.666666666667 | 3679.3333333333335 |
| Lg14 | 2218.3333333333335 | 1516.0 |
| Lg15 | 0.0 | 226.33333333333331 |
| Lg16 | 1794.6666666666665 | 2113.3333333333335 |
| Lg17 | 0.0 | 10.666666666666657 |
| Lg18_HF_1uM | 0.0 | 24.666666666666657 |
| Lg7_HF_3uM | 590.3333333333333 | 986.6666666666667 |
| Lg12_HF_3uM | 0.0 | 7.333333333333343 |
| Blank 1 | 70.0 | 0.0 |
| Blank 2 | 150.66666666666674 | 0.0 |
| Blank 3 | 0.0 | 0.0 |
| Blank 4 | 0.0 | 0.0 |
| Blank 5 | 0.0 | 0.0 |MFI
### Chart: Bm2-4
| Category | AM151 (90) IgG1 | AM 151 (92) IgG1 |
|---|---|---|
| Ng1_1uM
 | 0.0 | 0.0 |
| Ng1_3uM
 | 0.0 | 22.666666666666686 |
| Ng2_1uM | 62.5 | 0.0 |
| Ng2_3uM | 20.0 | 821.3333333333335 |
| Ng2_10uM | 0.0 | 0.0 |
| Ng3_1uM | 0.0 | 40.33333333333337 |
| Ng3_3uM | 0.0 | 0.0 |
| Ng3_10uM | 0.0 | 0.0 |
| Ng3_30uM | 239.33333333333337 | 0.0 |
| Ng4_1uM | 0.0 | 0.0 |
| Ng4_3uM | 0.0 | 0.0 |
| Ng5_1uM | 0.0 | 0.0 |
| Ng5_3uM | 0.0 | 0.0 |
| Ng6_1uM | 0.0 | 0.0 |
| Ng7_1uM | 0.0 | 0.0 |
| Ng7_3uM | 0.0 | 0.0 |
| Ng8_1uM | 0.0 | 0.0 |
| Ng8_3uM | 25.333333333333314 | 0.0 |
| Ng8_10uM | 0.0 | 0.0 |
| Ng9_1uM | 0.0 | 0.0 |
| Ng9_3uM | 0.0 | 0.0 |
| Ng10_3uM | 0.0 | 0.0 |
| Ng10_1uM | 0.0 | 0.0 |
| Ng11 | 0.0 | 0.0 |
| Ng12_1uM | 528.6666666666666 | 0.0 |
| Ng12_3uM | 2991.0 | 185.00000000000006 |
| Ng13_1uM | 0.0 | 0.0 |
| Ng14_1uM | 4325.333333333333 | 686.6666666666667 |
| Ng15_1uM | 1410.0 | 0.0 |
| Ng16_1uM | 781.6666666666667 | 0.0 |
| Ng17_1uM | 0.0 | 0.0 |
| Ng18_1uM | 1108.3333333333333 | 0.0 |
| Ng18_3uM | 4795.333333333333 | 100.0 |
| Ng18_10uM | 10118.0 | 0.0 |
| Ng19_1uM | 387.0 | 0.0 |
| Ng19_3uM | 2299.0 | 7.666666666666686 |
| Ng19_10uM | 3740.0 | 0.0 |
| Ng20_1uM | 406.0 | 0.0 |
| Ng20_3uM | 4423.333333333333 | 0.0 |
| Ng20_10uM | 12137.333333333334 | 392.00000000000006 |
| Ng21_1uM | 69.0 | 55.666666666666686 |
| Ng21 _3uM | 1425.0 | 22.0 |
| Ng21_10uM | 9013.333333333334 | 1706.0 |
| Ng22_1uM | 0.0 | 0.0 |
| Ng23_1uM | 0.0 | 0.0 |
| Ng24_1uM | 299.0 | 0.0 |
| Ng24_3uM | 1670.6666666666667 | 154.33333333333337 |
| Ng25_1uM | 1332.6666666666667 | 1214.0 |
| Ng25_3uM | 432.66666666666663 | 1318.3333333333335 |
| Ng26_1uM | 2074.6666666666665 | 1122.3333333333335 |
| Ng27_1uM | 986.3333333333333 | 214.00000000000006 |
| Ng27_3uM | 614.6666666666666 | 2326.0 |
| Ng28_1uM | 325.0 | 174.0 |
| Ng29_1uM | 0.0 | 0.0 |
| Ng30_1uM | 57.333333333333314 | 0.0 |
| Ng31_1uM | 0.0 | 0.0 |
| Ng31_3uM | 0.0 | 0.0 |
| Ng31_10uM | 0.0 | 0.0 |
| Ng31_30uM | 1433.5 | 0.0 |
| Ng32_1uM | 31421.333333333332 | 588.0 |
| Ng33_1uM | 10243.333333333334 | 47.0 |
| Ng34_1uM | 85.33333333333331 | 272.3333333333333 |
| Ng34_3uM | 557.3333333333334 | 0.0 |
| Ng35_1uM | 185.0 | 0.0 |
| Ng36_1uM | 2882.3333333333335 | 1978.3333333333333 |
| Ng36_3uM | 3696.6666666666665 | 3081.0 |
| Lg2_HF_10uM | 50619.666666666664 | 8787.0 |
| Lg1_1uM | 1884.0 | 472.6666666666667 |
| Lg2_1uM | 15710.666666666666 | 3902.3333333333335 |
| Lg2_3uM | 51118.333333333336 | 4114.0 |
| Lg2_10uM | 58374.666666666664 | 23932.333333333336 |
| Lg3_1uM | 11978.333333333334 | 1494.0 |
| Lg4_1uM | 16738.666666666668 | 12385.666666666666 |
| Lg5_3uM | 62197.666666666664 | 11793.333333333332 |
| Lg6_1uM | 20124.333333333332 | 3576.0 |
| Lg7_1uM | 10300.0 | 3170.6666666666665 |
| Lg7_3uM | 61091.333333333336 | 19763.666666666668 |
| Lg8_1uM | 22413.0 | 4518.666666666667 |
| Lg9 | 9500.333333333334 | 3504.6666666666665 |
| Lg10_1uM | 2621.6666666666665 | 0.0 |
| Lg11_1uM | 47642.0 | 5593.666666666667 |
| Lg12_1uM | 41089.666666666664 | 6908.0 |
| Lg12_3uM | 64657.0 | 8252.333333333332 |
| Lg13 | 44707.333333333336 | 6747.666666666667 |
| Lg14 | 35332.0 | 916.0 |
| Lg15 | 11931.333333333334 | 577.6666666666667 |
| Lg16 | 30768.333333333332 | 3370.333333333333 |
| Lg17 | 467.66666666666663 | 431.3333333333333 |
| Lg18_HF_1uM | 2938.5 | 68.0 |
| Lg7_HF_3uM | 42533.666666666664 | 2524.6666666666665 |
| Lg12_HF_3uM | 4238.333333333333 | 371.3333333333333 |
| Blank 1 | 53.333333333333314 | 0.0 |
| Blank 2 | 0.0 | 0.0 |
| Blank 3 | 0.0 | 135.33333333333337 |
| Blank 4 | 0.0 | 112.0 |
| Blank 5 | 26.666666666666686 | 0.0 |MFI
### Chart: Bm2-5
| Category | AM 154 (90) IgG1 | AM 154 (92) IgG1 |
|---|---|---|
| Ng1_1uM
 | 46.666666666666686 | 241.33333333333331 |
| Ng1_3uM
 | 1375.0 | 540.3333333333334 |
| Ng2_1uM | 107.66666666666666 | 99.66666666666666 |
| Ng2_3uM | 560.0 | 3354.0 |
| Ng2_10uM | 296.0 | 329.6666666666667 |
| Ng3_1uM | 0.0 | 0.0 |
| Ng3_3uM | 0.0 | 5.0 |
| Ng3_10uM | 0.0 | 330.3333333333333 |
| Ng3_30uM | 0.0 | 29.0 |
| Ng4_1uM | 144.66666666666666 | 93.66666666666666 |
| Ng4_3uM | 0.0 | 166.0 |
| Ng5_1uM | 0.0 | 199.33333333333331 |
| Ng5_3uM | 0.0 | 472.33333333333337 |
| Ng6_1uM | 0.0 | 116.66666666666669 |
| Ng7_1uM | 109.33333333333334 | 143.33333333333331 |
| Ng7_3uM | 18.0 | 36.33333333333334 |
| Ng8_1uM | 0.0 | 2.333333333333343 |
| Ng8_3uM | 0.0 | 74.0 |
| Ng8_10uM | 0.0 | 43.66666666666666 |
| Ng9_1uM | 0.0 | 62.33333333333334 |
| Ng9_3uM | 0.0 | 202.33333333333331 |
| Ng10_3uM | 0.0 | 43.66666666666666 |
| Ng10_1uM | 0.0 | 212.0 |
| Ng11 | 821.0 | 195.0 |
| Ng12_1uM | 4596.333333333333 | 148.66666666666669 |
| Ng12_3uM | 28879.0 | 512.3333333333334 |
| Ng13_1uM | 15.0 | 38.66666666666666 |
| Ng14_1uM | 17872.666666666664 | 596.0 |
| Ng15_1uM | 4183.333333333333 | 175.0 |
| Ng16_1uM | 3615.3333333333335 | 237.33333333333331 |
| Ng17_1uM | 0.0 | 301.0 |
| Ng18_1uM | 1910.3333333333333 | 109.66666666666669 |
| Ng18_3uM | 1586.0 | 353.6666666666667 |
| Ng18_10uM | 7895.666666666666 | 681.0 |
| Ng19_1uM | 3764.666666666667 | 55.33333333333334 |
| Ng19_3uM | 23812.666666666664 | 436.33333333333337 |
| Ng19_10uM | 33378.66666666667 | 604.3333333333334 |
| Ng20_1uM | 2330.0 | 10.666666666666657 |
| Ng20_3uM | 18052.333333333332 | 440.0 |
| Ng20_10uM | 63325.0 | 1398.3333333333333 |
| Ng21_1uM | 516.6666666666667 | 14.0 |
| Ng21 _3uM | 4938.333333333333 | 304.3333333333333 |
| Ng21_10uM | 48117.333333333336 | 842.6666666666666 |
| Ng22_1uM | 130.66666666666666 | 186.66666666666669 |
| Ng23_1uM | 87.33333333333334 | 0.0 |
| Ng24_1uM | 277.66666666666663 | 181.66666666666669 |
| Ng24_3uM | 5053.666666666666 | 193.0 |
| Ng25_1uM | 89.33333333333334 | 116.33333333333331 |
| Ng25_3uM | 465.33333333333337 | 101.33333333333334 |
| Ng26_1uM | 3602.0 | 211.66666666666669 |
| Ng27_1uM | 686.0 | 64.0 |
| Ng27_3uM | 4145.0 | 152.66666666666669 |
| Ng28_1uM | 1499.3333333333333 | 160.0 |
| Ng29_1uM | 0.0 | 69.33333333333334 |
| Ng30_1uM | 183.33333333333334 | 10.666666666666657 |
| Ng31_1uM | 12.333333333333343 | 9.333333333333343 |
| Ng31_3uM | 0.0 | 54.33333333333334 |
| Ng31_10uM | 27.333333333333343 | 147.0 |
| Ng31_30uM | 0.0 | 160.66666666666669 |
| Ng32_1uM | 29087.333333333332 | 1898.0 |
| Ng33_1uM | 11391.0 | 713.6666666666666 |
| Ng34_1uM | 1425.6666666666665 | 28.666666666666657 |
| Ng34_3uM | 19056.666666666664 | 191.33333333333331 |
| Ng35_1uM | 2849.0 | 88.0 |
| Ng36_1uM | 10950.333333333334 | 309.0 |
| Ng36_3uM | 20719.333333333332 | 308.3333333333333 |
| Lg2_HF_10uM | 18655.333333333332 | 34483.0 |
| Lg1_1uM | 751.6666666666667 | 116.66666666666669 |
| Lg2_1uM | 21756.666666666664 | 2841.0 |
| Lg2_3uM | 39491.333333333336 | 17140.0 |
| Lg2_10uM | 64736.333333333336 | 64750.333333333336 |
| Lg3_1uM | 4703.0 | 2438.0 |
| Lg4_1uM | 41775.333333333336 | 5182.333333333333 |
| Lg5_3uM | 17885.333333333332 | 3833.6666666666665 |
| Lg6_1uM | 20312.333333333332 | 1490.0 |
| Lg7_1uM | 34518.0 | 1244.6666666666667 |
| Lg7_3uM | 63880.0 | 2631.0 |
| Lg8_1uM | 32321.0 | 1442.0 |
| Lg9 | 34998.333333333336 | 881.6666666666667 |
| Lg10_1uM | 3931.0000000000005 | 150.66666666666669 |
| Lg11_1uM | 55862.333333333336 | 3368.0 |
| Lg12_1uM | 62418.66666666667 | 1797.0 |
| Lg12_3uM | 64863.333333333336 | 6022.666666666667 |
| Lg13 | 10688.333333333334 | 2255.6666666666665 |
| Lg14 | 15014.333333333334 | 2095.6666666666665 |
| Lg15 | 5362.333333333333 | 217.66666666666669 |
| Lg16 | 41144.333333333336 | 813.0 |
| Lg17 | 4782.333333333333 | 63.33333333333334 |
| Lg18_HF_1uM | 9015.666666666668 | 316.3333333333333 |
| Lg7_HF_3uM | 36735.333333333336 | 2907.0 |
| Lg12_HF_3uM | 10579.333333333334 | 512.0 |
| Blank 1 | 0.0 | 11.0 |
| Blank 2 | 62.33333333333334 | 0.0 |
| Blank 3 | 0.0 | 0.0 |
| Blank 4 | 89.66666666666666 | 0.0 |
| Blank 5 | 0.0 | 26.666666666666657 |MFI
Before DEC treatment After DEC treatment

## Slide 20
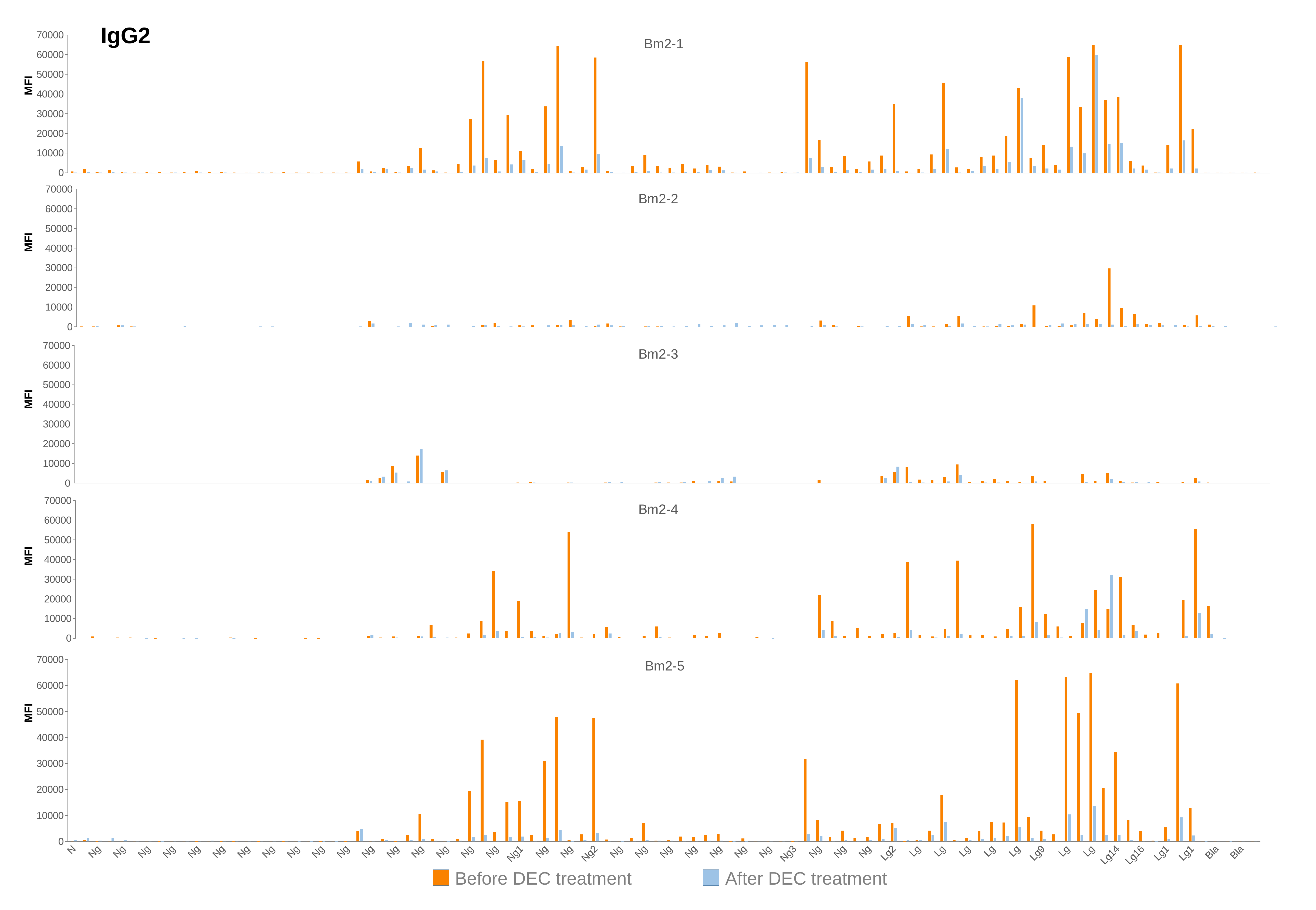

IgG2
### Chart: Bm2-1
| Category | AM 103 (90) IgG2 | AM 103 (92) IgG2 |
|---|---|---|
| Ng1_1uM
 | 631.0 | 31.666666666666657 |
| Ng1_3uM
 | 1875.0 | 313.6666666666667 |
| Ng2_1uM | 433.33333333333337 | 55.0 |
| Ng2_3uM | 1436.0 | 210.33333333333331 |
| Ng2_10uM | 531.0 | 30.666666666666657 |
| Ng3_1uM | 49.33333333333334 | 0.0 |
| Ng3_3uM | 154.33333333333331 | 0.0 |
| Ng3_10uM | 188.0 | 1.3333333333333428 |
| Ng3_30uM | 121.66666666666669 | 24.666666666666657 |
| Ng4_1uM | 507.33333333333337 | 5.0 |
| Ng4_3uM | 1049.6666666666667 | 25.666666666666657 |
| Ng5_1uM | 393.0 | 10.666666666666657 |
| Ng5_3uM | 245.0 | 34.33333333333334 |
| Ng6_1uM | 133.33333333333331 | 1.0 |
| Ng7_1uM | 0.0 | 0.0 |
| Ng7_3uM | 146.33333333333331 | 28.0 |
| Ng8_1uM | 60.0 | 0.0 |
| Ng8_3uM | 223.0 | 1.3333333333333428 |
| Ng8_10uM | 85.0 | 0.0 |
| Ng9_1uM | 42.66666666666666 | 0.0 |
| Ng9_3uM | 20.0 | 2.0 |
| Ng10_3uM | 21.333333333333343 | 0.0 |
| Ng10_1uM | 51.0 | 0.0 |
| Ng11 | 5690.333333333333 | 1756.6666666666667 |
| Ng12_1uM | 656.0 | 282.3333333333333 |
| Ng12_3uM | 2435.0 | 1970.0 |
| Ng13_1uM | 161.66666666666669 | 24.333333333333343 |
| Ng14_1uM | 3403.3333333333335 | 2541.6666666666665 |
| Ng15_1uM | 12702.0 | 1597.0 |
| Ng16_1uM | 1198.6666666666667 | 657.0 |
| Ng17_1uM | 151.66666666666669 | 0.6666666666666572 |
| Ng18_1uM | 4556.333333333333 | 461.66666666666663 |
| Ng18_3uM | 27172.666666666668 | 3717.6666666666665 |
| Ng18_10uM | 56803.0 | 7482.666666666667 |
| Ng19_1uM | 6384.0 | 681.3333333333334 |
| Ng19_3uM | 29257.0 | 4157.0 |
| Ng19_10uM | 11226.0 | 6459.0 |
| Ng20_1uM | 1991.6666666666665 | 160.0 |
| Ng20_3uM | 33710.666666666664 | 4365.333333333333 |
| Ng20_10uM | 64629.0 | 13712.333333333334 |
| Ng21_1uM | 787.3333333333334 | 125.33333333333331 |
| Ng21 _3uM | 2985.3333333333335 | 1533.6666666666667 |
| Ng21_10uM | 58560.0 | 9348.333333333334 |
| Ng22_1uM | 767.0 | 228.0 |
| Ng23_1uM | 13.333333333333343 | 0.0 |
| Ng24_1uM | 3330.6666666666665 | 315.3333333333333 |
| Ng24_3uM | 8894.333333333334 | 1105.6666666666667 |
| Ng25_1uM | 3341.3333333333335 | 87.33333333333334 |
| Ng25_3uM | 2488.0 | 91.33333333333334 |
| Ng26_1uM | 4570.0 | 327.0 |
| Ng27_1uM | 2128.3333333333335 | 319.3333333333333 |
| Ng27_3uM | 4071.666666666667 | 1414.6666666666667 |
| Ng28_1uM | 3079.0 | 1180.6666666666667 |
| Ng29_1uM | 79.0 | 0.0 |
| Ng30_1uM | 621.0 | 9.333333333333343 |
| Ng31_1uM | 8.0 | 0.0 |
| Ng31_3uM | 135.33333333333331 | 12.333333333333343 |
| Ng31_10uM | 171.66666666666669 | 70.66666666666666 |
| Ng31_30uM | 0.0 | 16.666666666666657 |
| Ng32_1uM | 56293.0 | 7485.666666666667 |
| Ng33_1uM | 16704.0 | 2850.6666666666665 |
| Ng34_1uM | 2850.0 | 281.0 |
| Ng34_3uM | 8441.0 | 1452.6666666666667 |
| Ng35_1uM | 1912.3333333333335 | 298.3333333333333 |
| Ng36_1uM | 5725.333333333333 | 1557.0 |
| Ng36_3uM | 8737.333333333334 | 1779.0 |
| Lg2_HF_10uM | 35142.333333333336 | 783.3333333333334 |
| Lg1_1uM | 664.3333333333334 | 0.0 |
| Lg2_1uM | 1842.0 | 77.33333333333334 |
| Lg2_3uM | 9227.333333333334 | 1933.3333333333335 |
| Lg2_10uM | 45774.666666666664 | 12030.666666666666 |
| Lg3_1uM | 2698.0 | 19.0 |
| Lg4_1uM | 1811.0 | 750.6666666666666 |
| Lg5_3uM | 8097.0 | 3536.0 |
| Lg6_1uM | 8665.333333333334 | 2045.3333333333335 |
| Lg7_1uM | 18646.666666666668 | 5621.666666666667 |
| Lg7_3uM | 42935.666666666664 | 38163.0 |
| Lg8_1uM | 7486.0 | 3213.3333333333335 |
| Lg9 | 14116.333333333334 | 2146.3333333333335 |
| Lg10_1uM | 3941.0 | 1608.0 |
| Lg11_1uM | 58876.666666666664 | 13198.666666666666 |
| Lg12_1uM | 33423.0 | 9884.333333333334 |
| Lg12_3uM | 64991.0 | 59662.333333333336 |
| Lg13 | 37201.333333333336 | 14794.0 |
| Lg14 | 38511.0 | 15036.0 |
| Lg15 | 5805.0 | 2123.0 |
| Lg16 | 3707.0 | 1583.3333333333333 |
| Lg17 | 114.33333333333331 | 39.66666666666666 |
| Lg18_HF_1uM | 14239.0 | 2157.6666666666665 |
| Lg7_HF_3uM | 64992.333333333336 | 16443.0 |
| Lg12_HF_3uM | 22094.333333333332 | 2175.6666666666665 |
| Blank 1 | 0.0 | 0.0 |
| Blank 2 | 0.0 | 0.0 |
| Blank 3 | 0.0 | 0.0 |
| Blank 4 | 0.0 | 0.0 |
| Blank 5 | 52.66666666666666 | 0.0 |MFI
### Chart: Bm2-2
| Category | AM 113 (90) IgG2 | AM113 (92) IgG2 |
|---|---|---|
| Ng1_1uM
 | 188.66666666666669 | 0.0 |
| Ng1_3uM
 | 154.66666666666669 | 406.16666666666663 |
| Ng2_1uM | 0.0 | 0.0 |
| Ng2_3uM | 683.3333333333334 | 690.8333333333334 |
| Ng2_10uM | 199.33333333333331 | 198.5 |
| Ng3_1uM | 0.0 | 0.0 |
| Ng3_3uM | 46.33333333333334 | 15.833333333333314 |
| Ng3_10uM | 0.0 | 46.5 |
| Ng3_30uM | 6.333333333333343 | 427.5 |
| Ng4_1uM | 0.0 | 0.0 |
| Ng4_3uM | 44.33333333333334 | 50.5 |
| Ng5_1uM | 43.0 | 60.5 |
| Ng5_3uM | 42.66666666666666 | 97.16666666666669 |
| Ng6_1uM | 1.0 | 0.0 |
| Ng7_1uM | 22.0 | 165.83333333333331 |
| Ng7_3uM | 66.0 | 134.16666666666669 |
| Ng8_1uM | 24.666666666666657 | 0.0 |
| Ng8_3uM | 53.0 | 65.16666666666669 |
| Ng8_10uM | 7.666666666666657 | 0.0 |
| Ng9_1uM | 2.666666666666657 | 84.16666666666669 |
| Ng9_3uM | 2.333333333333343 | 82.16666666666669 |
| Ng10_3uM | 0.0 | 0.0 |
| Ng10_1uM | 13.666666666666657 | 140.5 |
| Ng11 | 2863.3333333333335 | 1704.5 |
| Ng12_1uM | 0.0 | 48.833333333333314 |
| Ng12_3uM | 9.0 | 103.5 |
| Ng13_1uM | 0.0 | 1959.1666666666665 |
| Ng14_1uM | 71.66666666666666 | 1090.8333333333333 |
| Ng15_1uM | 267.6666666666667 | 879.1666666666667 |
| Ng16_1uM | 54.33333333333334 | 1155.8333333333333 |
| Ng17_1uM | 6.0 | 0.0 |
| Ng18_1uM | 55.0 | 444.83333333333337 |
| Ng18_3uM | 841.0 | 672.5 |
| Ng18_10uM | 1825.3333333333333 | 426.83333333333337 |
| Ng19_1uM | 88.33333333333334 | 144.5 |
| Ng19_3uM | 686.6666666666666 | 100.5 |
| Ng19_10uM | 741.3333333333334 | 0.0 |
| Ng20_1uM | 23.666666666666657 | 744.8333333333333 |
| Ng20_3uM | 1048.3333333333333 | 928.1666666666667 |
| Ng20_10uM | 3300.3333333333335 | 771.8333333333333 |
| Ng21_1uM | 14.666666666666657 | 487.83333333333337 |
| Ng21 _3uM | 247.66666666666669 | 1054.1666666666667 |
| Ng21_10uM | 1657.3333333333333 | 670.5 |
| Ng22_1uM | 130.66666666666669 | 588.5 |
| Ng23_1uM | 2.333333333333343 | 43.5 |
| Ng24_1uM | 143.66666666666669 | 318.5 |
| Ng24_3uM | 192.0 | 361.83333333333337 |
| Ng25_1uM | 44.33333333333334 | 143.16666666666669 |
| Ng25_3uM | 0.0 | 498.16666666666663 |
| Ng26_1uM | 49.66666666666666 | 1377.5 |
| Ng27_1uM | 0.0 | 571.5 |
| Ng27_3uM | 54.33333333333334 | 738.1666666666667 |
| Ng28_1uM | 73.0 | 1809.1666666666665 |
| Ng29_1uM | 0.6666666666666572 | 473.83333333333337 |
| Ng30_1uM | 4.666666666666657 | 732.5 |
| Ng31_1uM | 0.0 | 913.1666666666667 |
| Ng31_3uM | 58.66666666666666 | 803.8333333333333 |
| Ng31_10uM | 65.0 | 81.16666666666669 |
| Ng31_30uM | 12.333333333333343 | 271.83333333333337 |
| Ng32_1uM | 3189.3333333333335 | 958.5 |
| Ng33_1uM | 866.0 | 185.16666666666669 |
| Ng34_1uM | 31.333333333333343 | 46.166666666666686 |
| Ng34_3uM | 245.0 | 141.16666666666669 |
| Ng35_1uM | 76.66666666666666 | 0.0 |
| Ng36_1uM | 12.0 | 305.16666666666663 |
| Ng36_3uM | 24.0 | 453.5 |
| Lg2_HF_10uM | 5426.0 | 1487.1666666666667 |
| Lg1_1uM | 117.0 | 1021.1666666666667 |
| Lg2_1uM | 102.66666666666666 | 22.5 |
| Lg2_3uM | 1477.3333333333333 | 316.16666666666663 |
| Lg2_10uM | 5308.333333333333 | 1658.8333333333333 |
| Lg3_1uM | 28.666666666666657 | 454.5 |
| Lg4_1uM | 140.0 | 202.16666666666663 |
| Lg5_3uM | 500.0 | 1496.8333333333333 |
| Lg6_1uM | 301.3333333333333 | 720.5 |
| Lg7_1uM | 1530.6666666666667 | 1130.5 |
| Lg7_3uM | 10923.0 | 63.5 |
| Lg8_1uM | 389.33333333333337 | 867.5 |
| Lg9 | 530.3333333333334 | 1644.8333333333333 |
| Lg10_1uM | 688.6666666666666 | 1571.8333333333333 |
| Lg11_1uM | 6829.333333333333 | 1328.8333333333333 |
| Lg12_1uM | 4136.0 | 1429.1666666666667 |
| Lg12_3uM | 29591.666666666668 | 1152.1666666666667 |
| Lg13 | 9685.333333333334 | 450.83333333333337 |
| Lg14 | 6388.333333333333 | 1297.8333333333333 |
| Lg15 | 1481.0 | 893.1666666666667 |
| Lg16 | 1778.0 | 770.8333333333333 |
| Lg17 | 40.0 | 796.1666666666667 |
| Lg18_HF_1uM | 913.0 | 107.16666666666669 |
| Lg7_HF_3uM | 5780.666666666667 | 633.1666666666666 |
| Lg12_HF_3uM | 1119.6666666666667 | 460.5 |
| Blank 1 | 0.0 | 419.16666666666663 |
| Blank 2 | 0.0 | 0.0 |
| Blank 3 | 0.0 | 0.0 |
| Blank 4 | 0.0 | 0.0 |
| Blank 5 | 0.0 | 186.5 |MFI
### Chart: Bm2-3
| Category | AM134 (90) IgG2 | AM 134 (92) IgG2 |
|---|---|---|
| Ng1_1uM
 | 1.3333333333333428 | 12.0 |
| Ng1_3uM
 | 85.33333333333334 | 119.33333333333331 |
| Ng2_1uM | 2.333333333333343 | 0.0 |
| Ng2_3uM | 155.66666666666669 | 177.66666666666669 |
| Ng2_10uM | 25.333333333333343 | 93.33333333333334 |
| Ng3_1uM | 0.0 | 0.0 |
| Ng3_3uM | 0.0 | 0.0 |
| Ng3_10uM | 0.0 | 0.0 |
| Ng3_30uM | 0.0 | 0.0 |
| Ng4_1uM | 0.0 | 1.0 |
| Ng4_3uM | 0.0 | 3.0 |
| Ng5_1uM | 0.0 | 0.0 |
| Ng5_3uM | 9.333333333333343 | 17.666666666666657 |
| Ng6_1uM | 0.0 | 12.333333333333343 |
| Ng7_1uM | 0.0 | 0.0 |
| Ng7_3uM | 0.0 | 6.666666666666657 |
| Ng8_1uM | 0.0 | 0.0 |
| Ng8_3uM | 0.0 | 0.0 |
| Ng8_10uM | 0.0 | 0.0 |
| Ng9_1uM | 0.0 | 0.0 |
| Ng9_3uM | 0.0 | 0.0 |
| Ng10_3uM | 0.0 | 0.0 |
| Ng10_1uM | 0.0 | 0.0 |
| Ng11 | 1451.3333333333333 | 1276.6666666666667 |
| Ng12_1uM | 2514.3333333333335 | 3343.6666666666665 |
| Ng12_3uM | 8837.0 | 5288.333333333333 |
| Ng13_1uM | 69.66666666666666 | 753.0 |
| Ng14_1uM | 13978.666666666666 | 17414.333333333332 |
| Ng15_1uM | 34.33333333333334 | 0.0 |
| Ng16_1uM | 5687.333333333333 | 6474.0 |
| Ng17_1uM | 0.0 | 0.0 |
| Ng18_1uM | 3.0 | 0.0 |
| Ng18_3uM | 56.0 | 47.33333333333334 |
| Ng18_10uM | 118.33333333333331 | 142.0 |
| Ng19_1uM | 6.0 | 0.0 |
| Ng19_3uM | 237.33333333333331 | 133.66666666666669 |
| Ng19_10uM | 545.3333333333334 | 289.6666666666667 |
| Ng20_1uM | 2.333333333333343 | 0.0 |
| Ng20_3uM | 36.0 | 56.33333333333334 |
| Ng20_10uM | 322.0 | 247.66666666666669 |
| Ng21_1uM | 3.0 | 0.0 |
| Ng21 _3uM | 3.666666666666657 | 8.666666666666657 |
| Ng21_10uM | 329.3333333333333 | 382.66666666666663 |
| Ng22_1uM | 155.66666666666669 | 578.0 |
| Ng23_1uM | 0.0 | 0.0 |
| Ng24_1uM | 41.0 | 81.0 |
| Ng24_3uM | 314.6666666666667 | 401.66666666666663 |
| Ng25_1uM | 280.3333333333333 | 190.66666666666669 |
| Ng25_3uM | 215.33333333333331 | 380.33333333333337 |
| Ng26_1uM | 958.3333333333333 | 53.33333333333334 |
| Ng27_1uM | 156.33333333333331 | 909.6666666666667 |
| Ng27_3uM | 1211.3333333333333 | 2624.3333333333335 |
| Ng28_1uM | 766.0 | 3269.6666666666665 |
| Ng29_1uM | 0.0 | 0.0 |
| Ng30_1uM | 0.0 | 0.0 |
| Ng31_1uM | 12.666666666666657 | 0.0 |
| Ng31_3uM | 42.33333333333334 | 34.0 |
| Ng31_10uM | 61.0 | 109.33333333333331 |
| Ng31_30uM | 77.0 | 119.0 |
| Ng32_1uM | 1533.0 | 145.66666666666669 |
| Ng33_1uM | 101.33333333333334 | 71.0 |
| Ng34_1uM | 0.0 | 0.0 |
| Ng34_3uM | 46.33333333333334 | 16.333333333333343 |
| Ng35_1uM | 59.0 | 9.333333333333343 |
| Ng36_1uM | 3704.3333333333335 | 2775.6666666666665 |
| Ng36_3uM | 5731.666666666667 | 8301.0 |
| Lg2_HF_10uM | 8029.666666666667 | 717.6666666666666 |
| Lg1_1uM | 1742.3333333333333 | 234.33333333333331 |
| Lg2_1uM | 1482.3333333333333 | 82.0 |
| Lg2_3uM | 2968.6666666666665 | 811.3333333333334 |
| Lg2_10uM | 9462.333333333334 | 4163.666666666667 |
| Lg3_1uM | 744.0 | 77.0 |
| Lg4_1uM | 1266.6666666666667 | 226.33333333333331 |
| Lg5_3uM | 2070.0 | 255.66666666666669 |
| Lg6_1uM | 1008.6666666666667 | 169.33333333333331 |
| Lg7_1uM | 555.0 | 64.33333333333334 |
| Lg7_3uM | 3490.3333333333335 | 869.3333333333334 |
| Lg8_1uM | 1161.3333333333333 | 137.0 |
| Lg9 | 61.33333333333334 | 32.0 |
| Lg10_1uM | 20.0 | 2.0 |
| Lg11_1uM | 4499.0 | 357.6666666666667 |
| Lg12_1uM | 1162.3333333333333 | 165.66666666666669 |
| Lg12_3uM | 5086.333333333333 | 2089.0 |
| Lg13 | 1161.6666666666667 | 435.0 |
| Lg14 | 292.0 | 353.6666666666667 |
| Lg15 | 120.33333333333331 | 639.0 |
| Lg16 | 551.6666666666666 | 116.0 |
| Lg17 | 18.333333333333343 | 0.3333333333333428 |
| Lg18_HF_1uM | 390.66666666666663 | 11.666666666666657 |
| Lg7_HF_3uM | 2596.3333333333335 | 829.3333333333334 |
| Lg12_HF_3uM | 299.3333333333333 | 26.333333333333343 |
| Blank 1 | 0.0 | 0.0 |
| Blank 2 | 0.0 | 0.0 |
| Blank 3 | 0.0 | 0.0 |
| Blank 4 | 0.0 | 0.0 |
| Blank 5 | 0.0 | 0.0 |MFI
### Chart: Bm2-4
| Category | AM151 (90) IgG2 | AM 151 (92) IgG2 |
|---|---|---|
| Ng1_1uM
 | 91.33333333333334 | 82.66666666666666 |
| Ng1_3uM
 | 957.6666666666667 | 60.0 |
| Ng2_1uM | 218.66666666666669 | 0.0 |
| Ng2_3uM | 322.0 | 113.66666666666669 |
| Ng2_10uM | 396.33333333333337 | 142.66666666666669 |
| Ng3_1uM | 0.0 | 5.333333333333343 |
| Ng3_3uM | 13.0 | 0.0 |
| Ng3_10uM | 200.33333333333331 | 47.33333333333334 |
| Ng3_30uM | 0.0 | 0.3333333333333428 |
| Ng4_1uM | 160.66666666666669 | 4.333333333333343 |
| Ng4_3uM | 58.66666666666666 | 59.33333333333334 |
| Ng5_1uM | 48.0 | 0.0 |
| Ng5_3uM | 320.0 | 9.0 |
| Ng6_1uM | 47.33333333333334 | 37.66666666666666 |
| Ng7_1uM | 8.666666666666657 | 0.0 |
| Ng7_3uM | 74.33333333333334 | 47.33333333333334 |
| Ng8_1uM | 29.333333333333343 | 34.66666666666666 |
| Ng8_3uM | 62.66666666666666 | 80.66666666666666 |
| Ng8_10uM | 4.0 | 0.0 |
| Ng9_1uM | 2.666666666666657 | 0.0 |
| Ng9_3uM | 82.0 | 31.0 |
| Ng10_3uM | 0.0 | 0.0 |
| Ng10_1uM | 46.33333333333334 | 35.33333333333334 |
| Ng11 | 1167.6666666666667 | 1796.6666666666667 |
| Ng12_1uM | 375.66666666666663 | 19.666666666666657 |
| Ng12_3uM | 836.6666666666666 | 319.0 |
| Ng13_1uM | 22.333333333333343 | 31.0 |
| Ng14_1uM | 1308.3333333333333 | 844.6666666666666 |
| Ng15_1uM | 6642.0 | 829.3333333333334 |
| Ng16_1uM | 237.33333333333331 | 318.0 |
| Ng17_1uM | 345.6666666666667 | 0.0 |
| Ng18_1uM | 2369.0 | 22.666666666666657 |
| Ng18_3uM | 8643.0 | 1403.3333333333333 |
| Ng18_10uM | 34296.333333333336 | 3477.0 |
| Ng19_1uM | 3531.0 | 236.66666666666669 |
| Ng19_3uM | 18730.666666666668 | 683.3333333333334 |
| Ng19_10uM | 3792.6666666666665 | 735.3333333333334 |
| Ng20_1uM | 1000.0 | 286.0 |
| Ng20_3uM | 2279.3333333333335 | 2547.6666666666665 |
| Ng20_10uM | 53857.333333333336 | 3127.0 |
| Ng21_1uM | 322.3333333333333 | 0.0 |
| Ng21 _3uM | 2225.3333333333335 | 61.0 |
| Ng21_10uM | 5828.666666666667 | 2407.6666666666665 |
| Ng22_1uM | 467.0 | 219.0 |
| Ng23_1uM | 0.0 | 0.0 |
| Ng24_1uM | 1287.0 | 42.0 |
| Ng24_3uM | 5934.666666666667 | 530.6666666666666 |
| Ng25_1uM | 306.6666666666667 | 68.33333333333334 |
| Ng25_3uM | 236.66666666666669 | 139.66666666666669 |
| Ng26_1uM | 1708.6666666666667 | 108.66666666666669 |
| Ng27_1uM | 1134.0 | 71.0 |
| Ng27_3uM | 2711.0 | 99.66666666666666 |
| Ng28_1uM | 100.0 | 214.33333333333331 |
| Ng29_1uM | 38.66666666666666 | 58.33333333333334 |
| Ng30_1uM | 606.3333333333334 | 88.0 |
| Ng31_1uM | 0.0 | 0.6666666666666572 |
| Ng31_3uM | 22.666666666666657 | 64.0 |
| Ng31_10uM | 137.33333333333331 | 40.0 |
| Ng31_30uM | 61.0 | 43.0 |
| Ng32_1uM | 21910.0 | 4019.0 |
| Ng33_1uM | 8683.0 | 1299.3333333333333 |
| Ng34_1uM | 1385.0 | 72.33333333333334 |
| Ng34_3uM | 5092.0 | 145.66666666666669 |
| Ng35_1uM | 1314.3333333333333 | 48.33333333333334 |
| Ng36_1uM | 2090.6666666666665 | 130.0 |
| Ng36_3uM | 2880.3333333333335 | 543.3333333333334 |
| Lg2_HF_10uM | 38674.0 | 4028.0 |
| Lg1_1uM | 1609.0 | 138.33333333333331 |
| Lg2_1uM | 918.6666666666667 | 399.66666666666663 |
| Lg2_3uM | 4758.333333333333 | 1311.0 |
| Lg2_10uM | 39451.0 | 2327.3333333333335 |
| Lg3_1uM | 1424.0 | 194.0 |
| Lg4_1uM | 1749.0 | 133.33333333333331 |
| Lg5_3uM | 914.3333333333333 | 185.33333333333331 |
| Lg6_1uM | 4631.666666666667 | 1072.6666666666667 |
| Lg7_1uM | 15787.0 | 1045.0 |
| Lg7_3uM | 58087.333333333336 | 8163.333333333334 |
| Lg8_1uM | 12408.666666666666 | 1449.0 |
| Lg9 | 5968.666666666667 | 412.0 |
| Lg10_1uM | 1138.6666666666667 | 45.33333333333334 |
| Lg11_1uM | 7853.0 | 15004.666666666666 |
| Lg12_1uM | 24384.0 | 4026.666666666667 |
| Lg12_3uM | 14733.0 | 32206.333333333332 |
| Lg13 | 31077.333333333332 | 1582.3333333333333 |
| Lg14 | 6749.666666666667 | 3488.6666666666665 |
| Lg15 | 1876.3333333333333 | 151.66666666666669 |
| Lg16 | 2535.3333333333335 | 281.3333333333333 |
| Lg17 | 0.0 | 0.0 |
| Lg18_HF_1uM | 19499.333333333332 | 1130.0 |
| Lg7_HF_3uM | 55598.0 | 12813.0 |
| Lg12_HF_3uM | 16479.0 | 2326.6666666666665 |
| Blank 1 | 0.0 | 13.0 |
| Blank 2 | 0.0 | 0.0 |
| Blank 3 | 0.0 | 0.0 |
| Blank 4 | 0.0 | 0.0 |
| Blank 5 | 30.333333333333343 | 0.0 |MFI
### Chart: Bm2-5
| Category | AM 154 (90) IgG2 | AM 154 (92) IgG2 |
|---|---|---|
| Ng1_1uM
 | 139.33333333333331 | 570.3333333333334 |
| Ng1_3uM
 | 391.66666666666663 | 1365.3333333333333 |
| Ng2_1uM | 0.3333333333333428 | 336.0 |
| Ng2_3uM | 128.33333333333331 | 1294.0 |
| Ng2_10uM | 76.66666666666666 | 428.33333333333337 |
| Ng3_1uM | 0.0 | 75.0 |
| Ng3_3uM | 0.0 | 70.0 |
| Ng3_10uM | 6.333333333333343 | 29.666666666666657 |
| Ng3_30uM | 0.0 | 177.66666666666669 |
| Ng4_1uM | 0.0 | 213.0 |
| Ng4_3uM | 31.666666666666657 | 206.66666666666669 |
| Ng5_1uM | 0.0 | 296.3333333333333 |
| Ng5_3uM | 4.333333333333343 | 59.0 |
| Ng6_1uM | 4.333333333333343 | 150.0 |
| Ng7_1uM | 0.0 | 127.0 |
| Ng7_3uM | 7.333333333333343 | 145.66666666666669 |
| Ng8_1uM | 0.0 | 55.33333333333334 |
| Ng8_3uM | 11.333333333333343 | 105.0 |
| Ng8_10uM | 0.0 | 23.0 |
| Ng9_1uM | 0.0 | 53.33333333333334 |
| Ng9_3uM | 6.333333333333343 | 45.33333333333334 |
| Ng10_3uM | 0.0 | 23.0 |
| Ng10_1uM | 23.333333333333343 | 0.0 |
| Ng11 | 4021.0 | 4863.0 |
| Ng12_1uM | 120.33333333333331 | 170.0 |
| Ng12_3uM | 843.3333333333334 | 530.3333333333334 |
| Ng13_1uM | 29.666666666666657 | 85.66666666666666 |
| Ng14_1uM | 2436.6666666666665 | 627.6666666666666 |
| Ng15_1uM | 10608.666666666666 | 802.6666666666666 |
| Ng16_1uM | 1041.6666666666667 | 281.0 |
| Ng17_1uM | 0.0 | 45.66666666666666 |
| Ng18_1uM | 1033.0 | 329.3333333333333 |
| Ng18_3uM | 19507.333333333332 | 1646.0 |
| Ng18_10uM | 39206.0 | 2582.3333333333335 |
| Ng19_1uM | 3718.3333333333335 | 316.6666666666667 |
| Ng19_3uM | 15097.0 | 1620.0 |
| Ng19_10uM | 15546.333333333334 | 1918.3333333333335 |
| Ng20_1uM | 2411.0 | 122.33333333333331 |
| Ng20_3uM | 30824.666666666668 | 1498.0 |
| Ng20_10uM | 47829.333333333336 | 4405.0 |
| Ng21_1uM | 568.0 | 28.333333333333343 |
| Ng21 _3uM | 2680.3333333333335 | 521.0 |
| Ng21_10uM | 47378.333333333336 | 3274.3333333333335 |
| Ng22_1uM | 682.0 | 175.66666666666669 |
| Ng23_1uM | 0.0 | 14.333333333333343 |
| Ng24_1uM | 1384.6666666666667 | 67.0 |
| Ng24_3uM | 7182.0 | 589.6666666666666 |
| Ng25_1uM | 364.0 | 347.3333333333333 |
| Ng25_3uM | 399.33333333333337 | 316.3333333333333 |
| Ng26_1uM | 1872.6666666666667 | 138.33333333333331 |
| Ng27_1uM | 1669.3333333333333 | 188.66666666666669 |
| Ng27_3uM | 2521.0 | 322.6666666666667 |
| Ng28_1uM | 2812.0 | 342.6666666666667 |
| Ng29_1uM | 24.666666666666657 | 10.0 |
| Ng30_1uM | 1136.0 | 26.0 |
| Ng31_1uM | 0.0 | 0.0 |
| Ng31_3uM | 64.0 | 113.33333333333331 |
| Ng31_10uM | 77.33333333333334 | 132.0 |
| Ng31_30uM | 28.333333333333343 | 77.66666666666666 |
| Ng32_1uM | 31846.666666666668 | 2880.6666666666665 |
| Ng33_1uM | 8305.666666666666 | 2046.0 |
| Ng34_1uM | 1643.0 | 202.33333333333331 |
| Ng34_3uM | 4119.666666666667 | 611.3333333333334 |
| Ng35_1uM | 1377.3333333333333 | 189.33333333333331 |
| Ng36_1uM | 1546.6666666666667 | 528.6666666666666 |
| Ng36_3uM | 6749.666666666667 | 889.0 |
| Lg2_HF_10uM | 6968.666666666667 | 5226.666666666667 |
| Lg1_1uM | 20.0 | 456.66666666666663 |
| Lg2_1uM | 526.0 | 444.66666666666663 |
| Lg2_3uM | 4125.333333333333 | 2369.3333333333335 |
| Lg2_10uM | 18011.0 | 7367.0 |
| Lg3_1uM | 461.33333333333337 | 319.0 |
| Lg4_1uM | 1317.0 | 466.0 |
| Lg5_3uM | 3993.0 | 966.6666666666667 |
| Lg6_1uM | 7497.0 | 1431.0 |
| Lg7_1uM | 7286.333333333333 | 2133.0 |
| Lg7_3uM | 62174.333333333336 | 5627.0 |
| Lg8_1uM | 9360.0 | 1236.3333333333333 |
| Lg9 | 4144.333333333333 | 1066.0 |
| Lg10_1uM | 2729.3333333333335 | 288.6666666666667 |
| Lg11_1uM | 63214.0 | 10382.333333333334 |
| Lg12_1uM | 49314.333333333336 | 2416.0 |
| Lg12_3uM | 64997.0 | 13486.666666666666 |
| Lg13 | 20440.0 | 2401.3333333333335 |
| Lg14 | 34360.0 | 2480.6666666666665 |
| Lg15 | 8069.666666666666 | 408.33333333333337 |
| Lg16 | 4029.666666666667 | 186.66666666666669 |
| Lg17 | 292.0 | 39.0 |
| Lg18_HF_1uM | 5423.333333333333 | 926.3333333333333 |
| Lg7_HF_3uM | 60773.666666666664 | 9227.0 |
| Lg12_HF_3uM | 12926.333333333334 | 2274.3333333333335 |
| Blank 1 | 0.0 | 1.6666666666666572 |
| Blank 2 | 0.0 | 0.0 |
| Blank 3 | 0.0 | 5.333333333333343 |
| Blank 4 | 0.0 | 0.0 |
| Blank 5 | 0.0 | 0.0 |MFI
Before DEC treatment After DEC treatment

## Slide 21
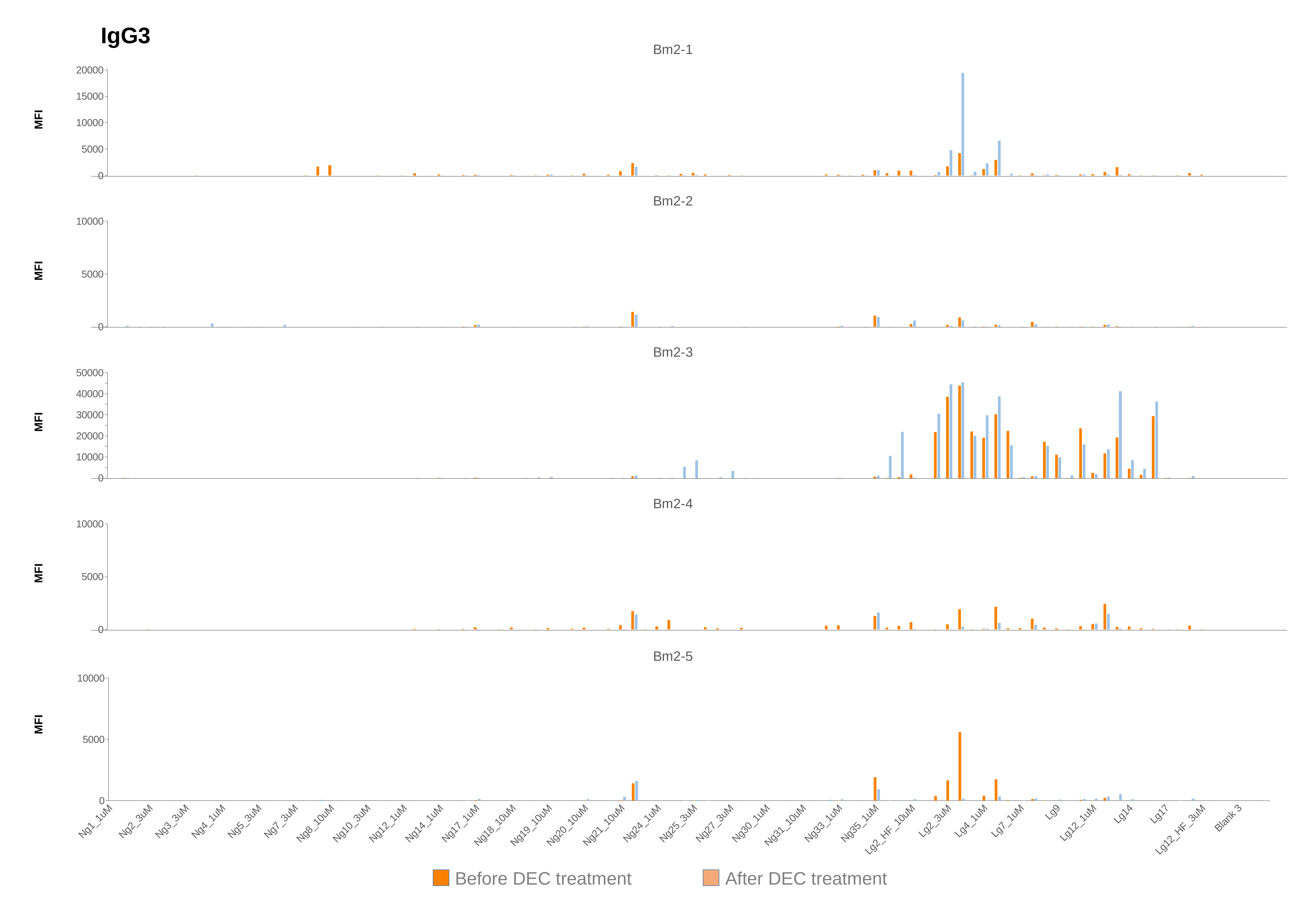

IgG3
### Chart: Bm2-1
| Category | AM 103 (90) IgG3 | AM 103 (92) IgG3 |
|---|---|---|
| Ng1_1uM
 | 0.0 | 0.0 |
| Ng1_3uM
 | 0.0 | 0.0 |
| Ng2_1uM | 0.0 | 0.0 |
| Ng2_3uM | 0.0 | 0.0 |
| Ng2_10uM | 0.0 | 0.0 |
| Ng3_1uM | 0.0 | 0.0 |
| Ng3_3uM | 0.0 | 0.0 |
| Ng3_10uM | 4.0 | 0.0 |
| Ng3_30uM | 0.0 | 0.0 |
| Ng4_1uM | 0.0 | 0.0 |
| Ng4_3uM | 0.0 | 0.0 |
| Ng5_1uM | 0.0 | 0.0 |
| Ng5_3uM | 0.0 | 0.0 |
| Ng6_1uM | 0.0 | 0.0 |
| Ng7_1uM | 0.0 | 0.0 |
| Ng7_3uM | 0.0 | 0.0 |
| Ng8_1uM | 37.33333333333334 | 0.0 |
| Ng8_3uM | 1659.3333333333333 | 0.0 |
| Ng8_10uM | 1908.6666666666665 | 0.0 |
| Ng9_1uM | 0.0 | 0.0 |
| Ng9_3uM | 0.0 | 0.0 |
| Ng10_3uM | 0.0 | 0.0 |
| Ng10_1uM | 37.33333333333334 | 0.0 |
| Ng11 | 0.0 | 0.0 |
| Ng12_1uM | 7.666666666666657 | 0.0 |
| Ng12_3uM | 414.33333333333337 | 0.0 |
| Ng13_1uM | 0.0 | 0.0 |
| Ng14_1uM | 170.33333333333331 | 2.666666666666657 |
| Ng15_1uM | 0.0 | 0.0 |
| Ng16_1uM | 112.33333333333331 | 24.666666666666657 |
| Ng17_1uM | 123.66666666666669 | 101.0 |
| Ng18_1uM | 0.0 | 0.0 |
| Ng18_3uM | 0.0 | 0.0 |
| Ng18_10uM | 111.33333333333331 | 15.0 |
| Ng19_1uM | 0.0 | 0.0 |
| Ng19_3uM | 9.666666666666657 | 0.0 |
| Ng19_10uM | 137.66666666666669 | 181.66666666666669 |
| Ng20_1uM | 0.0 | 0.0 |
| Ng20_3uM | 59.33333333333334 | 0.0 |
| Ng20_10uM | 352.3333333333333 | 11.333333333333343 |
| Ng21_1uM | 0.0 | 0.0 |
| Ng21 _3uM | 148.33333333333331 | 0.0 |
| Ng21_10uM | 789.3333333333334 | 49.33333333333334 |
| Ng22_1uM | 2327.3333333333335 | 1647.6666666666667 |
| Ng23_1uM | 0.0 | 0.0 |
| Ng24_1uM | 27.0 | 0.0 |
| Ng24_3uM | 24.0 | 0.0 |
| Ng25_1uM | 303.6666666666667 | 22.666666666666657 |
| Ng25_3uM | 488.33333333333337 | 114.66666666666669 |
| Ng26_1uM | 200.33333333333331 | 0.0 |
| Ng27_1uM | 0.0 | 0.0 |
| Ng27_3uM | 91.66666666666666 | 0.0 |
| Ng28_1uM | 35.33333333333334 | 0.0 |
| Ng29_1uM | 0.0 | 0.0 |
| Ng30_1uM | 0.0 | 0.0 |
| Ng31_1uM | 0.0 | 0.0 |
| Ng31_3uM | 0.0 | 0.0 |
| Ng31_10uM | 0.0 | 0.0 |
| Ng31_30uM | 0.0 | 0.0 |
| Ng32_1uM | 217.33333333333331 | 0.0 |
| Ng33_1uM | 128.33333333333331 | 29.666666666666657 |
| Ng34_1uM | 10.0 | 0.0 |
| Ng34_3uM | 124.66666666666669 | 4.0 |
| Ng35_1uM | 1034.6666666666667 | 1019.6666666666667 |
| Ng36_1uM | 441.0 | 0.0 |
| Ng36_3uM | 901.6666666666667 | 0.0 |
| Lg2_HF_10uM | 915.0 | 84.0 |
| Lg1_1uM | 0.0 | 0.0 |
| Lg2_1uM | 65.66666666666666 | 656.0 |
| Lg2_3uM | 1709.0 | 4788.666666666667 |
| Lg2_10uM | 4213.333333333333 | 19442.333333333332 |
| Lg3_1uM | 44.66666666666666 | 727.3333333333334 |
| Lg4_1uM | 1207.0 | 2280.6666666666665 |
| Lg5_3uM | 2934.0 | 6574.666666666667 |
| Lg6_1uM | 0.0 | 338.0 |
| Lg7_1uM | 53.0 | 0.0 |
| Lg7_3uM | 411.0 | 4.666666666666657 |
| Lg8_1uM | 62.0 | 178.66666666666669 |
| Lg9 | 89.66666666666666 | 58.0 |
| Lg10_1uM | 0.0 | 0.0 |
| Lg11_1uM | 172.66666666666669 | 190.66666666666669 |
| Lg12_1uM | 231.33333333333331 | 0.0 |
| Lg12_3uM | 644.0 | 207.0 |
| Lg13 | 1574.3333333333333 | 103.33333333333334 |
| Lg14 | 252.66666666666669 | 15.333333333333343 |
| Lg15 | 12.333333333333343 | 0.0 |
| Lg16 | 9.333333333333343 | 12.333333333333343 |
| Lg17 | 0.0 | 0.0 |
| Lg18_HF_1uM | 18.333333333333343 | 0.0 |
| Lg7_HF_3uM | 466.0 | 0.0 |
| Lg12_HF_3uM | 128.0 | 0.0 |
| Blank 1 | 0.0 | 0.0 |
| Blank 2 | 0.0 | 0.0 |
| Blank 3 | 0.0 | 0.0 |
| Blank 4 | 0.0 | 0.0 |
| Blank 5 | 0.0 | 0.0 |MFI
### Chart: Bm2-2
| Category | AM 113 (90) IgG3 | AM113 (92) IgG3 |
|---|---|---|
| Ng1_1uM
 | 0.0 | 0.0 |
| Ng1_3uM
 | 0.0 | 112.0 |
| Ng2_1uM | 0.0 | 5.0 |
| Ng2_3uM | 0.0 | 23.333333333333343 |
| Ng2_10uM | 0.0 | 8.666666666666657 |
| Ng3_1uM | 0.0 | 0.0 |
| Ng3_3uM | 0.0 | 0.0 |
| Ng3_10uM | 0.0 | 0.0 |
| Ng3_30uM | 0.0 | 304.0 |
| Ng4_1uM | 0.0 | 12.333333333333343 |
| Ng4_3uM | 0.0 | 0.0 |
| Ng5_1uM | 0.0 | 13.666666666666657 |
| Ng5_3uM | 0.0 | 37.33333333333334 |
| Ng6_1uM | 0.0 | 0.0 |
| Ng7_1uM | 0.0 | 175.66666666666669 |
| Ng7_3uM | 0.0 | 0.0 |
| Ng8_1uM | 0.0 | 0.0 |
| Ng8_3uM | 0.0 | 0.0 |
| Ng8_10uM | 0.0 | 0.0 |
| Ng9_1uM | 0.0 | 0.0 |
| Ng9_3uM | 0.0 | 19.333333333333343 |
| Ng10_3uM | 0.0 | 0.0 |
| Ng10_1uM | 0.0 | 5.666666666666657 |
| Ng11 | 0.0 | 0.0 |
| Ng12_1uM | 0.0 | 0.0 |
| Ng12_3uM | 0.0 | 11.666666666666657 |
| Ng13_1uM | 0.0 | 0.0 |
| Ng14_1uM | 0.0 | 4.666666666666657 |
| Ng15_1uM | 0.0 | 0.0 |
| Ng16_1uM | 6.333333333333343 | 15.0 |
| Ng17_1uM | 150.33333333333331 | 197.0 |
| Ng18_1uM | 0.0 | 0.0 |
| Ng18_3uM | 0.0 | 0.0 |
| Ng18_10uM | 0.0 | 8.666666666666657 |
| Ng19_1uM | 0.0 | 0.0 |
| Ng19_3uM | 0.0 | 0.0 |
| Ng19_10uM | 0.0 | 0.0 |
| Ng20_1uM | 0.0 | 0.0 |
| Ng20_3uM | 0.0 | 1.6666666666666572 |
| Ng20_10uM | 7.0 | 48.0 |
| Ng21_1uM | 0.0 | 0.0 |
| Ng21 _3uM | 0.0 | 0.0 |
| Ng21_10uM | 13.0 | 32.0 |
| Ng22_1uM | 1414.0 | 1137.0 |
| Ng23_1uM | 0.0 | 0.0 |
| Ng24_1uM | 0.0 | 2.0 |
| Ng24_3uM | 0.0 | 86.66666666666666 |
| Ng25_1uM | 0.0 | 0.0 |
| Ng25_3uM | 0.0 | 0.0 |
| Ng26_1uM | 0.0 | 0.0 |
| Ng27_1uM | 0.0 | 0.0 |
| Ng27_3uM | 0.0 | 0.0 |
| Ng28_1uM | 0.0 | 13.333333333333343 |
| Ng29_1uM | 0.0 | 0.0 |
| Ng30_1uM | 0.0 | 0.0 |
| Ng31_1uM | 0.0 | 0.0 |
| Ng31_3uM | 0.0 | 0.0 |
| Ng31_10uM | 0.0 | 0.0 |
| Ng31_30uM | 0.0 | 0.0 |
| Ng32_1uM | 0.0 | 0.0 |
| Ng33_1uM | 3.666666666666657 | 113.66666666666669 |
| Ng34_1uM | 0.0 | 0.0 |
| Ng34_3uM | 0.0 | 8.333333333333343 |
| Ng35_1uM | 1057.3333333333333 | 918.0 |
| Ng36_1uM | 0.0 | 19.0 |
| Ng36_3uM | 0.0 | 0.0 |
| Lg2_HF_10uM | 255.0 | 605.6666666666666 |
| Lg1_1uM | 0.0 | 0.0 |
| Lg2_1uM | 0.0 | 0.0 |
| Lg2_3uM | 179.66666666666669 | 72.0 |
| Lg2_10uM | 869.3333333333334 | 622.6666666666666 |
| Lg3_1uM | 0.0 | 22.333333333333343 |
| Lg4_1uM | 3.333333333333343 | 43.66666666666666 |
| Lg5_3uM | 171.66666666666669 | 120.0 |
| Lg6_1uM | 0.0 | 0.0 |
| Lg7_1uM | 0.0 | 0.6666666666666572 |
| Lg7_3uM | 475.66666666666663 | 234.33333333333331 |
| Lg8_1uM | 0.0 | 35.66666666666666 |
| Lg9 | 24.0 | 0.0 |
| Lg10_1uM | 0.0 | 8.333333333333343 |
| Lg11_1uM | 15.666666666666657 | 31.0 |
| Lg12_1uM | 22.333333333333343 | 0.0 |
| Lg12_3uM | 190.33333333333331 | 231.66666666666669 |
| Lg13 | 48.66666666666666 | 7.333333333333343 |
| Lg14 | 0.0 | 26.666666666666657 |
| Lg15 | 0.0 | 0.0 |
| Lg16 | 0.0 | 9.333333333333343 |
| Lg17 | 0.0 | 0.0 |
| Lg18_HF_1uM | 0.0 | 7.333333333333343 |
| Lg7_HF_3uM | 32.66666666666666 | 91.0 |
| Lg12_HF_3uM | 0.0 | 9.666666666666657 |
| Blank 1 | 0.0 | 0.0 |
| Blank 2 | 0.0 | 0.0 |
| Blank 3 | 0.0 | 0.0 |
| Blank 4 | 0.0 | 0.0 |
| Blank 5 | 0.0 | 0.0 |MFI
### Chart: Bm2-3
| Category | AM134 (90) IgG3 | AM 134 (92) IgG3 |
|---|---|---|
| Ng1_1uM
 | 0.0 | 0.0 |
| Ng1_3uM
 | 35.33333333333334 | 63.0 |
| Ng2_1uM | 0.0 | 0.0 |
| Ng2_3uM | 0.0 | 0.0 |
| Ng2_10uM | 0.0 | 0.0 |
| Ng3_1uM | 0.0 | 0.0 |
| Ng3_3uM | 0.0 | 0.0 |
| Ng3_10uM | 0.0 | 0.0 |
| Ng3_30uM | 13.666666666666657 | 0.0 |
| Ng4_1uM | 0.0 | 0.0 |
| Ng4_3uM | 0.0 | 0.0 |
| Ng5_1uM | 0.0 | 0.0 |
| Ng5_3uM | 0.0 | 0.0 |
| Ng6_1uM | 0.0 | 5.333333333333343 |
| Ng7_1uM | 0.0 | 0.0 |
| Ng7_3uM | 0.0 | 1.6666666666666572 |
| Ng8_1uM | 0.0 | 0.0 |
| Ng8_3uM | 0.0 | 11.0 |
| Ng8_10uM | 0.0 | 0.0 |
| Ng9_1uM | 0.0 | 0.0 |
| Ng9_3uM | 0.0 | 0.0 |
| Ng10_3uM | 0.0 | 0.0 |
| Ng10_1uM | 0.0 | 0.0 |
| Ng11 | 0.0 | 0.0 |
| Ng12_1uM | 0.0 | 0.0 |
| Ng12_3uM | 0.0 | 20.666666666666657 |
| Ng13_1uM | 0.0 | 0.0 |
| Ng14_1uM | 63.66666666666666 | 74.33333333333334 |
| Ng15_1uM | 0.0 | 0.0 |
| Ng16_1uM | 0.6666666666666572 | 87.0 |
| Ng17_1uM | 166.66666666666669 | 92.66666666666666 |
| Ng18_1uM | 0.0 | 0.0 |
| Ng18_3uM | 0.0 | 0.0 |
| Ng18_10uM | 0.0 | 0.0 |
| Ng19_1uM | 0.0 | 20.666666666666657 |
| Ng19_3uM | 0.0 | 273.3333333333333 |
| Ng19_10uM | 0.0 | 648.0 |
| Ng20_1uM | 0.0 | 0.0 |
| Ng20_3uM | 0.0 | 13.666666666666657 |
| Ng20_10uM | 0.0 | 8.333333333333343 |
| Ng21_1uM | 0.0 | 0.0 |
| Ng21 _3uM | 0.0 | 25.0 |
| Ng21_10uM | 0.0 | 112.66666666666669 |
| Ng22_1uM | 897.6666666666667 | 1224.3333333333333 |
| Ng23_1uM | 0.0 | 0.0 |
| Ng24_1uM | 0.0 | 28.666666666666657 |
| Ng24_3uM | 0.0 | 19.0 |
| Ng25_1uM | 0.0 | 5367.0 |
| Ng25_3uM | 0.0 | 8425.0 |
| Ng26_1uM | 0.0 | 30.666666666666657 |
| Ng27_1uM | 0.0 | 401.66666666666663 |
| Ng27_3uM | 0.0 | 3434.3333333333335 |
| Ng28_1uM | 0.0 | 22.333333333333343 |
| Ng29_1uM | 0.0 | 19.333333333333343 |
| Ng30_1uM | 0.0 | 0.0 |
| Ng31_1uM | 0.0 | 0.0 |
| Ng31_3uM | 0.0 | 0.0 |
| Ng31_10uM | 0.0 | 0.0 |
| Ng31_30uM | 0.0 | 0.0 |
| Ng32_1uM | 0.0 | 3.0 |
| Ng33_1uM | 95.0 | 86.33333333333334 |
| Ng34_1uM | 0.0 | 0.0 |
| Ng34_3uM | 0.0 | 0.0 |
| Ng35_1uM | 762.6666666666666 | 1052.6666666666667 |
| Ng36_1uM | 23.666666666666657 | 10624.0 |
| Ng36_3uM | 405.0 | 21939.666666666668 |
| Lg2_HF_10uM | 1742.0 | 234.33333333333331 |
| Lg1_1uM | 0.0 | 7.666666666666657 |
| Lg2_1uM | 21854.0 | 30511.333333333332 |
| Lg2_3uM | 38532.333333333336 | 44474.666666666664 |
| Lg2_10uM | 43833.0 | 45434.333333333336 |
| Lg3_1uM | 22109.333333333332 | 20031.0 |
| Lg4_1uM | 19110.666666666668 | 29714.333333333332 |
| Lg5_3uM | 30319.0 | 38746.666666666664 |
| Lg6_1uM | 22322.0 | 15522.666666666666 |
| Lg7_1uM | 15.333333333333343 | 526.6666666666666 |
| Lg7_3uM | 820.0 | 958.3333333333333 |
| Lg8_1uM | 17190.666666666668 | 15298.0 |
| Lg9 | 11067.0 | 9787.0 |
| Lg10_1uM | 135.0 | 1322.3333333333333 |
| Lg11_1uM | 23662.666666666668 | 15900.666666666666 |
| Lg12_1uM | 2569.0 | 1911.3333333333335 |
| Lg12_3uM | 11742.0 | 13815.0 |
| Lg13 | 19266.666666666668 | 41099.666666666664 |
| Lg14 | 4405.333333333333 | 8554.0 |
| Lg15 | 1581.6666666666667 | 4386.0 |
| Lg16 | 29414.0 | 36286.666666666664 |
| Lg17 | 20.0 | 152.33333333333331 |
| Lg18_HF_1uM | 0.0 | 13.0 |
| Lg7_HF_3uM | 99.33333333333334 | 931.0 |
| Lg12_HF_3uM | 0.0 | 0.0 |
| Blank 1 | 0.0 | 0.0 |
| Blank 2 | 0.0 | 0.0 |
| Blank 3 | 0.0 | 0.0 |
| Blank 4 | 0.0 | 0.0 |
| Blank 5 | 0.0 | 0.0 |MFI
### Chart: Bm2-4
| Category | AM151 (90) IgG3 | AM 151 (92) IgG3 |
|---|---|---|
| Ng1_1uM
 | 0.0 | 0.0 |
| Ng1_3uM
 | 0.0 | 0.0 |
| Ng2_1uM | 0.0 | 0.0 |
| Ng2_3uM | 11.333333333333343 | 0.0 |
| Ng2_10uM | 0.0 | 0.0 |
| Ng3_1uM | 0.0 | 0.0 |
| Ng3_3uM | 0.0 | 0.0 |
| Ng3_10uM | 0.0 | 0.0 |
| Ng3_30uM | 0.0 | 0.0 |
| Ng4_1uM | 0.0 | 0.0 |
| Ng4_3uM | 0.0 | 0.0 |
| Ng5_1uM | 0.0 | 0.0 |
| Ng5_3uM | 0.0 | 0.0 |
| Ng6_1uM | 0.0 | 0.0 |
| Ng7_1uM | 0.0 | 0.0 |
| Ng7_3uM | 0.0 | 0.0 |
| Ng8_1uM | 0.0 | 0.0 |
| Ng8_3uM | 0.0 | 0.0 |
| Ng8_10uM | 0.0 | 0.0 |
| Ng9_1uM | 0.0 | 0.0 |
| Ng9_3uM | 0.0 | 0.0 |
| Ng10_3uM | 0.0 | 0.0 |
| Ng10_1uM | 0.0 | 0.0 |
| Ng11 | 0.0 | 0.0 |
| Ng12_1uM | 0.0 | 0.0 |
| Ng12_3uM | 45.33333333333334 | 0.0 |
| Ng13_1uM | 0.0 | 0.0 |
| Ng14_1uM | 15.666666666666657 | 0.0 |
| Ng15_1uM | 0.0 | 0.0 |
| Ng16_1uM | 59.0 | 0.0 |
| Ng17_1uM | 205.33333333333331 | 0.6666666666666572 |
| Ng18_1uM | 0.0 | 0.0 |
| Ng18_3uM | 38.66666666666666 | 0.0 |
| Ng18_10uM | 183.33333333333331 | 0.0 |
| Ng19_1uM | 0.0 | 0.0 |
| Ng19_3uM | 29.333333333333343 | 0.0 |
| Ng19_10uM | 137.66666666666669 | 0.0 |
| Ng20_1uM | 0.0 | 0.0 |
| Ng20_3uM | 65.0 | 0.0 |
| Ng20_10uM | 171.33333333333331 | 0.0 |
| Ng21_1uM | 0.0 | 0.0 |
| Ng21 _3uM | 55.0 | 0.0 |
| Ng21_10uM | 406.0 | 1.3333333333333428 |
| Ng22_1uM | 1740.6666666666667 | 1420.3333333333333 |
| Ng23_1uM | 20.333333333333343 | 0.0 |
| Ng24_1uM | 292.5 | 0.0 |
| Ng24_3uM | 899.3333333333333 | 0.0 |
| Ng25_1uM | 0.0 | 0.0 |
| Ng25_3uM | 0.0 | 0.0 |
| Ng26_1uM | 199.33333333333331 | 0.0 |
| Ng27_1uM | 102.0 | 0.0 |
| Ng27_3uM | 0.0 | 0.0 |
| Ng28_1uM | 146.33333333333331 | 0.0 |
| Ng29_1uM | 0.0 | 0.0 |
| Ng30_1uM | 0.0 | 0.0 |
| Ng31_1uM | 0.0 | 0.0 |
| Ng31_3uM | 0.0 | 0.0 |
| Ng31_10uM | 0.0 | 0.0 |
| Ng31_30uM | 0.0 | 0.0 |
| Ng32_1uM | 367.66666666666663 | 0.0 |
| Ng33_1uM | 388.66666666666663 | 0.0 |
| Ng34_1uM | 0.0 | 0.0 |
| Ng34_3uM | 0.0 | 0.0 |
| Ng35_1uM | 1287.6666666666667 | 1600.3333333333333 |
| Ng36_1uM | 180.66666666666669 | 0.0 |
| Ng36_3uM | 339.0 | 0.0 |
| Lg2_HF_10uM | 691.3333333333334 | 40.66666666666666 |
| Lg1_1uM | 0.0 | 0.0 |
| Lg2_1uM | 5.666666666666657 | 0.0 |
| Lg2_3uM | 493.33333333333337 | 34.0 |
| Lg2_10uM | 1917.6666666666665 | 247.0 |
| Lg3_1uM | 21.333333333333343 | 0.0 |
| Lg4_1uM | 58.66666666666666 | 79.33333333333334 |
| Lg5_3uM | 2150.6666666666665 | 620.6666666666666 |
| Lg6_1uM | 108.0 | 0.0 |
| Lg7_1uM | 129.0 | 0.0 |
| Lg7_3uM | 1036.0 | 433.66666666666663 |
| Lg8_1uM | 170.33333333333331 | 0.0 |
| Lg9 | 116.0 | 0.0 |
| Lg10_1uM | 22.0 | 0.0 |
| Lg11_1uM | 312.6666666666667 | 0.0 |
| Lg12_1uM | 510.0 | 558.0 |
| Lg12_3uM | 2428.3333333333335 | 1475.6666666666667 |
| Lg13 | 241.0 | 84.66666666666666 |
| Lg14 | 282.3333333333333 | 0.0 |
| Lg15 | 94.0 | 0.0 |
| Lg16 | 45.66666666666666 | 0.0 |
| Lg17 | 0.0 | 9.0 |
| Lg18_HF_1uM | 31.0 | 0.0 |
| Lg7_HF_3uM | 370.66666666666663 | 0.0 |
| Lg12_HF_3uM | 7.333333333333343 | 0.0 |
| Blank 1 | 0.0 | 0.0 |
| Blank 2 | 0.0 | 0.0 |
| Blank 3 | 0.0 | 0.0 |
| Blank 4 | 0.0 | 0.0 |
| Blank 5 | 0.0 | 0.0 |MFI
### Chart: Bm2-5
| Category | AM 154 (90) IgG3 | AM 154 (92) IgG3 |
|---|---|---|
| Ng1_1uM
 | 0.0 | 0.0 |
| Ng1_3uM
 | 0.0 | 0.0 |
| Ng2_1uM | 0.0 | 0.0 |
| Ng2_3uM | 0.0 | 0.0 |
| Ng2_10uM | 0.0 | 0.0 |
| Ng3_1uM | 0.0 | 0.0 |
| Ng3_3uM | 0.0 | 0.0 |
| Ng3_10uM | 0.0 | 0.0 |
| Ng3_30uM | 0.0 | 0.0 |
| Ng4_1uM | 0.0 | 0.0 |
| Ng4_3uM | 0.0 | 0.0 |
| Ng5_1uM | 0.0 | 0.0 |
| Ng5_3uM | 0.0 | 0.0 |
| Ng6_1uM | 0.0 | 0.0 |
| Ng7_1uM | 0.0 | 0.0 |
| Ng7_3uM | 0.0 | 0.0 |
| Ng8_1uM | 0.0 | 0.0 |
| Ng8_3uM | 0.0 | 66.66666666666666 |
| Ng8_10uM | 0.0 | 0.0 |
| Ng9_1uM | 0.0 | 0.0 |
| Ng9_3uM | 0.0 | 0.0 |
| Ng10_3uM | 0.0 | 0.0 |
| Ng10_1uM | 0.0 | 0.0 |
| Ng11 | 0.0 | 0.0 |
| Ng12_1uM | 0.0 | 0.0 |
| Ng12_3uM | 0.0 | 0.0 |
| Ng13_1uM | 0.0 | 0.0 |
| Ng14_1uM | 0.0 | 0.0 |
| Ng15_1uM | 0.0 | 0.0 |
| Ng16_1uM | 0.0 | 4.0 |
| Ng17_1uM | 45.0 | 131.0 |
| Ng18_1uM | 0.0 | 0.0 |
| Ng18_3uM | 0.0 | 0.0 |
| Ng18_10uM | 0.0 | 0.0 |
| Ng19_1uM | 0.0 | 0.0 |
| Ng19_3uM | 0.0 | 0.0 |
| Ng19_10uM | 0.0 | 0.0 |
| Ng20_1uM | 0.0 | 0.0 |
| Ng20_3uM | 0.0 | 0.0 |
| Ng20_10uM | 12.0 | 105.0 |
| Ng21_1uM | 0.0 | 0.0 |
| Ng21 _3uM | 0.0 | 31.666666666666657 |
| Ng21_10uM | 33.33333333333334 | 298.3333333333333 |
| Ng22_1uM | 1401.3333333333333 | 1613.6666666666667 |
| Ng23_1uM | 0.0 | 0.0 |
| Ng24_1uM | 0.0 | 0.0 |
| Ng24_3uM | 0.0 | 0.0 |
| Ng25_1uM | 0.0 | 25.0 |
| Ng25_3uM | 2.666666666666657 | 31.333333333333343 |
| Ng26_1uM | 0.0 | 1.6666666666666572 |
| Ng27_1uM | 0.0 | 0.0 |
| Ng27_3uM | 0.0 | 0.0 |
| Ng28_1uM | 0.0 | 0.0 |
| Ng29_1uM | 0.0 | 0.0 |
| Ng30_1uM | 0.0 | 0.0 |
| Ng31_1uM | 0.0 | 0.0 |
| Ng31_3uM | 0.0 | 0.0 |
| Ng31_10uM | 0.0 | 0.0 |
| Ng31_30uM | 0.0 | 0.0 |
| Ng32_1uM | 0.0 | 54.66666666666666 |
| Ng33_1uM | 7.333333333333343 | 110.66666666666669 |
| Ng34_1uM | 0.0 | 0.0 |
| Ng34_3uM | 0.0 | 0.0 |
| Ng35_1uM | 1917.0 | 923.0 |
| Ng36_1uM | 0.0 | 24.333333333333343 |
| Ng36_3uM | 0.0 | 30.333333333333343 |
| Lg2_HF_10uM | 0.0 | 113.66666666666669 |
| Lg1_1uM | 0.0 | 0.0 |
| Lg2_1uM | 361.0 | 0.0 |
| Lg2_3uM | 1656.3333333333333 | 0.0 |
| Lg2_10uM | 5606.333333333333 | 147.66666666666669 |
| Lg3_1uM | 0.0 | 0.0 |
| Lg4_1uM | 392.33333333333337 | 5.666666666666657 |
| Lg5_3uM | 1729.6666666666667 | 348.3333333333333 |
| Lg6_1uM | 32.0 | 40.33333333333334 |
| Lg7_1uM | 0.0 | 16.666666666666657 |
| Lg7_3uM | 133.33333333333331 | 163.0 |
| Lg8_1uM | 3.666666666666657 | 0.0 |
| Lg9 | 0.0 | 93.0 |
| Lg10_1uM | 0.0 | 0.0 |
| Lg11_1uM | 62.66666666666666 | 132.0 |
| Lg12_1uM | 10.666666666666657 | 145.66666666666669 |
| Lg12_3uM | 222.0 | 326.6666666666667 |
| Lg13 | 0.0 | 533.6666666666666 |
| Lg14 | 0.0 | 113.33333333333331 |
| Lg15 | 0.0 | 0.0 |
| Lg16 | 0.0 | 0.0 |
| Lg17 | 0.0 | 0.0 |
| Lg18_HF_1uM | 0.0 | 1.3333333333333428 |
| Lg7_HF_3uM | 0.0 | 142.0 |
| Lg12_HF_3uM | 0.0 | 49.33333333333334 |
| Blank 1 | 0.0 | 0.0 |
| Blank 2 | 0.0 | 0.0 |
| Blank 3 | 0.0 | 0.0 |
| Blank 4 | 0.0 | 0.0 |
| Blank 5 | 0.0 | 1.0 |MFI
Before DEC treatment After DEC treatment

## Slide 22
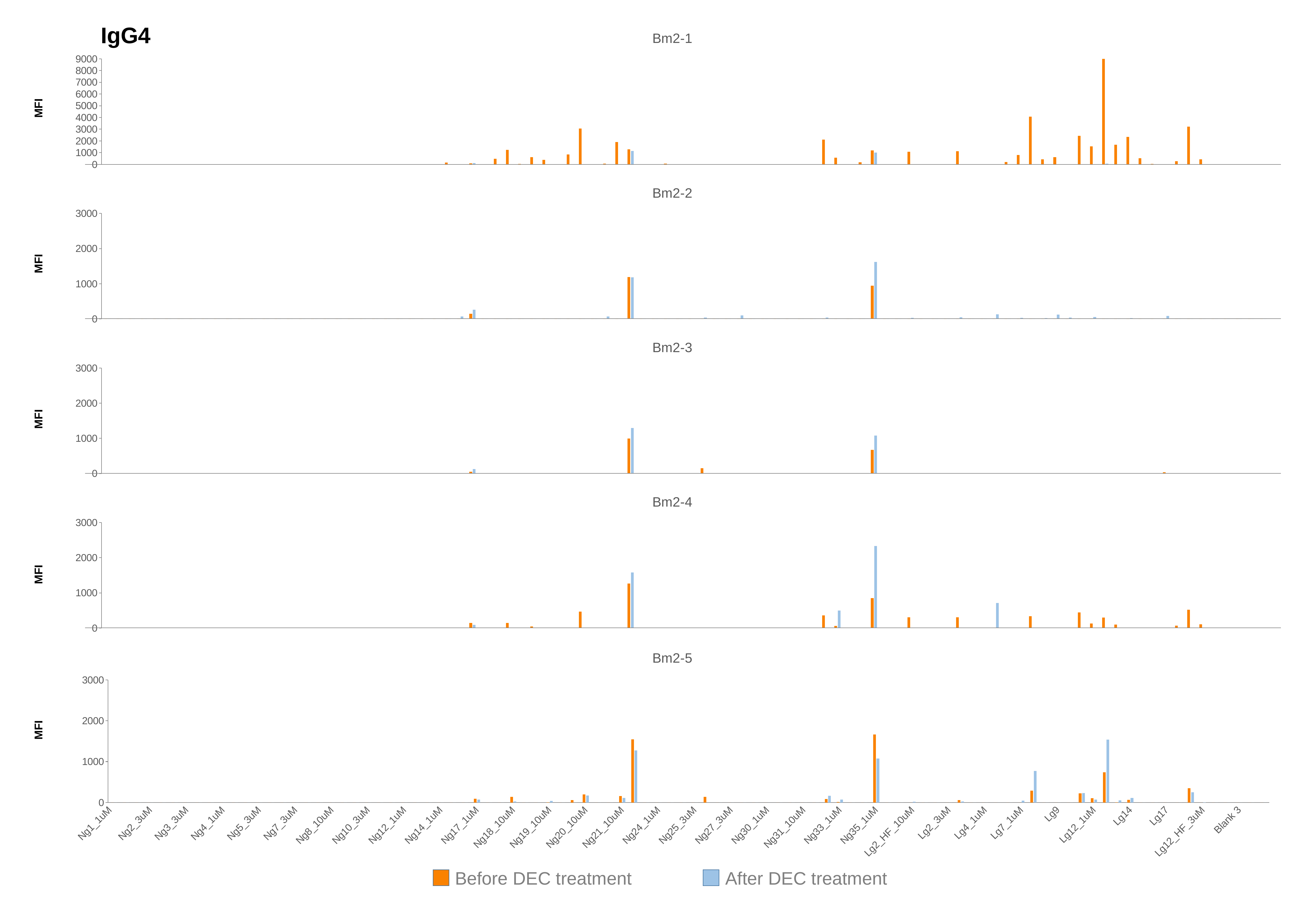

IgG4
### Chart: Bm2-1
| Category | AM 103 (90) IgG4 | AM 103 (92) IgG4 |
|---|---|---|
| Ng1_1uM
 | 0.0 | 0.0 |
| Ng1_3uM
 | 0.0 | 0.0 |
| Ng2_1uM | 0.0 | 0.0 |
| Ng2_3uM | 0.0 | 0.0 |
| Ng2_10uM | 0.0 | 0.0 |
| Ng3_1uM | 0.0 | 0.0 |
| Ng3_3uM | 0.0 | 0.0 |
| Ng3_10uM | 0.0 | 0.0 |
| Ng3_30uM | 0.0 | 0.0 |
| Ng4_1uM | 0.0 | 0.0 |
| Ng4_3uM | 0.0 | 0.0 |
| Ng5_1uM | 0.0 | 0.0 |
| Ng5_3uM | 0.0 | 0.0 |
| Ng6_1uM | 0.0 | 0.0 |
| Ng7_1uM | 0.0 | 0.0 |
| Ng7_3uM | 0.0 | 0.0 |
| Ng8_1uM | 0.0 | 0.0 |
| Ng8_3uM | 0.0 | 0.0 |
| Ng8_10uM | 0.0 | 0.0 |
| Ng9_1uM | 0.0 | 0.0 |
| Ng9_3uM | 0.0 | 0.0 |
| Ng10_3uM | 0.0 | 0.0 |
| Ng10_1uM | 0.0 | 0.0 |
| Ng11 | 0.0 | 0.0 |
| Ng12_1uM | 0.0 | 0.0 |
| Ng12_3uM | 0.0 | 0.0 |
| Ng13_1uM | 0.0 | 0.0 |
| Ng14_1uM | 0.0 | 0.0 |
| Ng15_1uM | 145.0 | 0.0 |
| Ng16_1uM | 0.0 | 0.0 |
| Ng17_1uM | 83.66666666666666 | 113.66666666666669 |
| Ng18_1uM | 1.0 | 0.0 |
| Ng18_3uM | 466.0 | 0.0 |
| Ng18_10uM | 1228.0 | 0.0 |
| Ng19_1uM | 32.0 | 0.0 |
| Ng19_3uM | 603.0 | 0.0 |
| Ng19_10uM | 383.0 | 0.0 |
| Ng20_1uM | 0.0 | 0.0 |
| Ng20_3uM | 842.3333333333334 | 0.0 |
| Ng20_10uM | 3044.3333333333335 | 0.0 |
| Ng21_1uM | 0.0 | 0.0 |
| Ng21 _3uM | 57.0 | 0.0 |
| Ng21_10uM | 1911.6666666666665 | 0.0 |
| Ng22_1uM | 1284.6666666666667 | 1138.6666666666667 |
| Ng23_1uM | 0.0 | 0.0 |
| Ng24_1uM | 0.0 | 0.0 |
| Ng24_3uM | 54.66666666666666 | 0.0 |
| Ng25_1uM | 0.0 | 0.0 |
| Ng25_3uM | 0.0 | 0.0 |
| Ng26_1uM | 17.666666666666657 | 0.0 |
| Ng27_1uM | 0.0 | 0.0 |
| Ng27_3uM | 1.0 | 0.0 |
| Ng28_1uM | 0.0 | 0.0 |
| Ng29_1uM | 0.0 | 0.0 |
| Ng30_1uM | 0.0 | 0.0 |
| Ng31_1uM | 0.0 | 0.0 |
| Ng31_3uM | 0.0 | 0.0 |
| Ng31_10uM | 0.0 | 0.0 |
| Ng31_30uM | 0.0 | 0.0 |
| Ng32_1uM | 2119.0 | 0.0 |
| Ng33_1uM | 566.3333333333334 | 0.0 |
| Ng34_1uM | 0.0 | 0.0 |
| Ng34_3uM | 165.0 | 0.0 |
| Ng35_1uM | 1200.3333333333333 | 1014.6666666666667 |
| Ng36_1uM | 0.0 | 0.0 |
| Ng36_3uM | 27.0 | 0.0 |
| Lg2_HF_10uM | 1074.6666666666667 | 0.0 |
| Lg1_1uM | 0.0 | 0.0 |
| Lg2_1uM | 0.0 | 0.0 |
| Lg2_3uM | 0.3333333333333428 | 0.0 |
| Lg2_10uM | 1118.3333333333333 | 6.333333333333343 |
| Lg3_1uM | 0.0 | 0.0 |
| Lg4_1uM | 0.0 | 0.0 |
| Lg5_3uM | 0.0 | 0.0 |
| Lg6_1uM | 189.66666666666669 | 0.0 |
| Lg7_1uM | 804.3333333333334 | 0.0 |
| Lg7_3uM | 4068.0 | 0.0 |
| Lg8_1uM | 426.33333333333337 | 0.0 |
| Lg9 | 613.0 | 0.0 |
| Lg10_1uM | 16.333333333333343 | 0.0 |
| Lg11_1uM | 2445.0 | 0.0 |
| Lg12_1uM | 1525.3333333333333 | 0.0 |
| Lg12_3uM | 11604.666666666666 | 50.66666666666666 |
| Lg13 | 1665.0 | 0.0 |
| Lg14 | 2350.0 | 0.0 |
| Lg15 | 525.6666666666666 | 0.0 |
| Lg16 | 30.333333333333343 | 0.0 |
| Lg17 | 0.0 | 0.0 |
| Lg18_HF_1uM | 275.6666666666667 | 0.0 |
| Lg7_HF_3uM | 3220.3333333333335 | 0.0 |
| Lg12_HF_3uM | 432.33333333333337 | 0.0 |
| Blank 1 | 0.0 | 0.0 |
| Blank 2 | 0.0 | 0.0 |
| Blank 3 | 0.0 | 0.0 |
| Blank 4 | 0.0 | 0.0 |
| Blank 5 | 0.0 | 0.0 |MFI
### Chart: Bm2-2
| Category | AM 113 (90) IgG4 | AM113 (92) IgG4 |
|---|---|---|
| Ng1_1uM
 | 0.0 | 0.0 |
| Ng1_3uM
 | 0.0 | 0.0 |
| Ng2_1uM | 0.0 | 0.0 |
| Ng2_3uM | 0.0 | 0.0 |
| Ng2_10uM | 0.0 | 0.0 |
| Ng3_1uM | 0.0 | 0.0 |
| Ng3_3uM | 0.0 | 0.0 |
| Ng3_10uM | 0.0 | 0.0 |
| Ng3_30uM | 0.0 | 0.0 |
| Ng4_1uM | 0.0 | 0.0 |
| Ng4_3uM | 0.0 | 0.0 |
| Ng5_1uM | 0.0 | 0.0 |
| Ng5_3uM | 0.0 | 0.0 |
| Ng6_1uM | 0.0 | 0.0 |
| Ng7_1uM | 0.0 | 0.0 |
| Ng7_3uM | 0.0 | 0.0 |
| Ng8_1uM | 0.0 | 0.0 |
| Ng8_3uM | 0.0 | 0.0 |
| Ng8_10uM | 0.0 | 0.0 |
| Ng9_1uM | 0.0 | 0.0 |
| Ng9_3uM | 0.0 | 0.0 |
| Ng10_3uM | 0.0 | 0.0 |
| Ng10_1uM | 0.0 | 0.0 |
| Ng11 | 0.0 | 0.0 |
| Ng12_1uM | 0.0 | 0.0 |
| Ng12_3uM | 0.0 | 0.0 |
| Ng13_1uM | 0.0 | 13.0 |
| Ng14_1uM | 0.0 | 6.333333333333343 |
| Ng15_1uM | 0.0 | 0.0 |
| Ng16_1uM | 0.0 | 66.66666666666666 |
| Ng17_1uM | 148.0 | 258.3333333333333 |
| Ng18_1uM | 0.0 | 0.0 |
| Ng18_3uM | 0.0 | 0.0 |
| Ng18_10uM | 0.0 | 0.0 |
| Ng19_1uM | 0.0 | 0.0 |
| Ng19_3uM | 0.0 | 0.0 |
| Ng19_10uM | 0.0 | 0.0 |
| Ng20_1uM | 0.0 | 0.0 |
| Ng20_3uM | 0.0 | 6.666666666666657 |
| Ng20_10uM | 0.0 | 0.0 |
| Ng21_1uM | 0.0 | 0.0 |
| Ng21 _3uM | 0.0 | 67.33333333333334 |
| Ng21_10uM | 0.0 | 15.0 |
| Ng22_1uM | 1189.3333333333333 | 1184.3333333333333 |
| Ng23_1uM | 0.0 | 0.0 |
| Ng24_1uM | 0.0 | 0.0 |
| Ng24_3uM | 0.0 | 0.0 |
| Ng25_1uM | 0.0 | 0.0 |
| Ng25_3uM | 0.0 | 17.0 |
| Ng26_1uM | 0.0 | 37.0 |
| Ng27_1uM | 0.0 | 0.0 |
| Ng27_3uM | 0.0 | 0.0 |
| Ng28_1uM | 0.0 | 103.33333333333334 |
| Ng29_1uM | 0.0 | 0.0 |
| Ng30_1uM | 0.0 | 0.0 |
| Ng31_1uM | 0.0 | 0.0 |
| Ng31_3uM | 0.0 | 4.666666666666657 |
| Ng31_10uM | 0.0 | 0.0 |
| Ng31_30uM | 0.0 | 0.0 |
| Ng32_1uM | 0.0 | 35.0 |
| Ng33_1uM | 0.0 | 14.333333333333343 |
| Ng34_1uM | 0.0 | 0.0 |
| Ng34_3uM | 0.0 | 0.0 |
| Ng35_1uM | 945.0 | 1624.0 |
| Ng36_1uM | 0.0 | 0.0 |
| Ng36_3uM | 0.0 | 0.0 |
| Lg2_HF_10uM | 0.0 | 32.33333333333334 |
| Lg1_1uM | 0.0 | 15.0 |
| Lg2_1uM | 0.0 | 0.0 |
| Lg2_3uM | 0.0 | 0.0 |
| Lg2_10uM | 0.0 | 48.0 |
| Lg3_1uM | 0.0 | 0.0 |
| Lg4_1uM | 0.0 | 0.0 |
| Lg5_3uM | 0.0 | 129.0 |
| Lg6_1uM | 0.0 | 0.0 |
| Lg7_1uM | 0.0 | 28.0 |
| Lg7_3uM | 0.0 | 0.0 |
| Lg8_1uM | 0.0 | 25.666666666666657 |
| Lg9 | 0.0 | 124.33333333333331 |
| Lg10_1uM | 0.0 | 39.0 |
| Lg11_1uM | 0.0 | 18.666666666666657 |
| Lg12_1uM | 0.0 | 50.66666666666666 |
| Lg12_3uM | 0.0 | 0.0 |
| Lg13 | 0.0 | 0.0 |
| Lg14 | 0.0 | 27.333333333333343 |
| Lg15 | 0.0 | 18.333333333333343 |
| Lg16 | 0.0 | 12.666666666666657 |
| Lg17 | 0.0 | 87.33333333333334 |
| Lg18_HF_1uM | 0.0 | 0.0 |
| Lg7_HF_3uM | 0.0 | 0.0 |
| Lg12_HF_3uM | 0.0 | 0.0 |
| Blank 1 | 0.0 | 0.0 |
| Blank 2 | 0.0 | 0.0 |
| Blank 3 | 0.0 | 0.0 |
| Blank 4 | 0.0 | 0.0 |
| Blank 5 | 0.0 | 0.0 |MFI
### Chart: Bm2-3
| Category | AM134 (90) IgG4 | AM 134 (92) IgG4 |
|---|---|---|
| Ng1_1uM
 | 0.0 | 0.0 |
| Ng1_3uM
 | 0.0 | 0.0 |
| Ng2_1uM | 0.0 | 0.0 |
| Ng2_3uM | 0.0 | 0.0 |
| Ng2_10uM | 0.0 | 0.0 |
| Ng3_1uM | 0.0 | 0.0 |
| Ng3_3uM | 0.0 | 0.0 |
| Ng3_10uM | 0.0 | 0.0 |
| Ng3_30uM | 0.0 | 0.0 |
| Ng4_1uM | 0.0 | 0.0 |
| Ng4_3uM | 0.0 | 0.0 |
| Ng5_1uM | 0.0 | 0.0 |
| Ng5_3uM | 0.0 | 0.0 |
| Ng6_1uM | 0.0 | 0.0 |
| Ng7_1uM | 0.0 | 0.0 |
| Ng7_3uM | 0.0 | 0.0 |
| Ng8_1uM | 0.0 | 0.0 |
| Ng8_3uM | 0.0 | 0.0 |
| Ng8_10uM | 0.0 | 0.0 |
| Ng9_1uM | 0.0 | 0.0 |
| Ng9_3uM | 0.0 | 0.0 |
| Ng10_3uM | 0.0 | 0.0 |
| Ng10_1uM | 0.0 | 0.0 |
| Ng11 | 0.0 | 0.0 |
| Ng12_1uM | 0.0 | 0.0 |
| Ng12_3uM | 0.0 | 0.0 |
| Ng13_1uM | 0.0 | 0.0 |
| Ng14_1uM | 0.0 | 0.0 |
| Ng15_1uM | 0.0 | 0.0 |
| Ng16_1uM | 0.0 | 0.0 |
| Ng17_1uM | 50.33333333333334 | 124.33333333333331 |
| Ng18_1uM | 0.0 | 0.0 |
| Ng18_3uM | 0.0 | 0.0 |
| Ng18_10uM | 0.0 | 0.0 |
| Ng19_1uM | 0.0 | 0.0 |
| Ng19_3uM | 0.0 | 0.0 |
| Ng19_10uM | 0.0 | 0.0 |
| Ng20_1uM | 0.0 | 0.0 |
| Ng20_3uM | 0.0 | 0.0 |
| Ng20_10uM | 0.0 | 0.0 |
| Ng21_1uM | 0.0 | 0.0 |
| Ng21 _3uM | 0.0 | 0.0 |
| Ng21_10uM | 0.0 | 0.0 |
| Ng22_1uM | 996.6666666666667 | 1296.6666666666667 |
| Ng23_1uM | 0.0 | 0.0 |
| Ng24_1uM | 0.0 | 0.0 |
| Ng24_3uM | 0.0 | 0.0 |
| Ng25_1uM | 0.0 | 0.0 |
| Ng25_3uM | 0.0 | 0.0 |
| Ng26_1uM | 150.0 | 0.0 |
| Ng27_1uM | 0.0 | 0.0 |
| Ng27_3uM | 0.0 | 0.0 |
| Ng28_1uM | 0.0 | 0.0 |
| Ng29_1uM | 0.0 | 0.0 |
| Ng30_1uM | 0.0 | 0.0 |
| Ng31_1uM | 0.0 | 0.0 |
| Ng31_3uM | 0.0 | 0.0 |
| Ng31_10uM | 0.0 | 0.0 |
| Ng31_30uM | 0.0 | 0.0 |
| Ng32_1uM | 0.0 | 0.0 |
| Ng33_1uM | 0.0 | 0.0 |
| Ng34_1uM | 0.0 | 0.0 |
| Ng34_3uM | 0.0 | 0.0 |
| Ng35_1uM | 671.3333333333334 | 1081.6666666666667 |
| Ng36_1uM | 0.0 | 0.0 |
| Ng36_3uM | 0.0 | 0.0 |
| Lg2_HF_10uM | 0.0 | 0.0 |
| Lg1_1uM | 0.0 | 0.0 |
| Lg2_1uM | 0.0 | 0.0 |
| Lg2_3uM | 0.0 | 0.0 |
| Lg2_10uM | 0.0 | 11.333333333333343 |
| Lg3_1uM | 0.0 | 0.0 |
| Lg4_1uM | 0.0 | 0.0 |
| Lg5_3uM | 0.0 | 0.0 |
| Lg6_1uM | 0.0 | 0.0 |
| Lg7_1uM | 0.0 | 0.0 |
| Lg7_3uM | 0.0 | 0.0 |
| Lg8_1uM | 0.0 | 0.0 |
| Lg9 | 0.0 | 0.0 |
| Lg10_1uM | 0.0 | 0.0 |
| Lg11_1uM | 0.0 | 0.0 |
| Lg12_1uM | 0.0 | 0.0 |
| Lg12_3uM | 0.0 | 0.0 |
| Lg13 | 0.0 | 0.0 |
| Lg14 | 0.0 | 0.0 |
| Lg15 | 0.0 | 0.0 |
| Lg16 | 0.0 | 0.0 |
| Lg17 | 32.33333333333334 | 0.0 |
| Lg18_HF_1uM | 0.0 | 0.0 |
| Lg7_HF_3uM | 0.0 | 0.0 |
| Lg12_HF_3uM | 0.0 | 0.0 |
| Blank 1 | 0.0 | 0.0 |
| Blank 2 | 0.0 | 0.0 |
| Blank 3 | 0.0 | 0.0 |
| Blank 4 | 0.0 | 0.0 |
| Blank 5 | 0.0 | 0.0 |MFI
### Chart: Bm2-4
| Category | AM151 (90) IgG4 | AM 151 (92) IgG4 |
|---|---|---|
| Ng1_1uM
 | 0.0 | 0.0 |
| Ng1_3uM
 | 0.0 | 0.0 |
| Ng2_1uM | 0.0 | 0.0 |
| Ng2_3uM | 0.0 | 0.0 |
| Ng2_10uM | 0.0 | 0.0 |
| Ng3_1uM | 0.0 | 0.0 |
| Ng3_3uM | 0.0 | 0.0 |
| Ng3_10uM | 0.0 | 0.0 |
| Ng3_30uM | 0.0 | 0.0 |
| Ng4_1uM | 0.0 | 0.0 |
| Ng4_3uM | 0.0 | 0.0 |
| Ng5_1uM | 0.0 | 0.0 |
| Ng5_3uM | 0.0 | 0.0 |
| Ng6_1uM | 0.0 | 0.0 |
| Ng7_1uM | 0.0 | 0.0 |
| Ng7_3uM | 0.0 | 0.0 |
| Ng8_1uM | 0.0 | 0.0 |
| Ng8_3uM | 0.0 | 0.0 |
| Ng8_10uM | 0.0 | 0.0 |
| Ng9_1uM | 0.0 | 0.0 |
| Ng9_3uM | 0.0 | 0.0 |
| Ng10_3uM | 0.0 | 0.0 |
| Ng10_1uM | 0.0 | 0.0 |
| Ng11 | 0.0 | 0.0 |
| Ng12_1uM | 0.0 | 0.0 |
| Ng12_3uM | 0.0 | 0.0 |
| Ng13_1uM | 0.0 | 0.0 |
| Ng14_1uM | 0.0 | 0.0 |
| Ng15_1uM | 0.0 | 0.0 |
| Ng16_1uM | 0.0 | 0.0 |
| Ng17_1uM | 143.0 | 91.0 |
| Ng18_1uM | 0.0 | 0.0 |
| Ng18_3uM | 0.0 | 0.0 |
| Ng18_10uM | 149.0 | 0.0 |
| Ng19_1uM | 0.0 | 0.0 |
| Ng19_3uM | 42.0 | 0.0 |
| Ng19_10uM | 0.0 | 0.0 |
| Ng20_1uM | 0.0 | 0.0 |
| Ng20_3uM | 0.0 | 0.0 |
| Ng20_10uM | 467.66666666666663 | 0.0 |
| Ng21_1uM | 0.0 | 0.0 |
| Ng21 _3uM | 0.0 | 0.0 |
| Ng21_10uM | 0.0 | 0.0 |
| Ng22_1uM | 1263.3333333333333 | 1580.6666666666667 |
| Ng23_1uM | 0.0 | 0.0 |
| Ng24_1uM | 0.0 | 0.0 |
| Ng24_3uM | 0.0 | 0.0 |
| Ng25_1uM | 0.0 | 0.0 |
| Ng25_3uM | 0.0 | 0.0 |
| Ng26_1uM | 0.0 | 0.0 |
| Ng27_1uM | 0.0 | 0.0 |
| Ng27_3uM | 0.0 | 0.0 |
| Ng28_1uM | 0.0 | 0.0 |
| Ng29_1uM | 0.0 | 0.0 |
| Ng30_1uM | 0.0 | 0.0 |
| Ng31_1uM | 0.0 | 0.0 |
| Ng31_3uM | 0.0 | 0.0 |
| Ng31_10uM | 0.0 | 0.0 |
| Ng31_30uM | 0.0 | 0.0 |
| Ng32_1uM | 362.0 | 0.0 |
| Ng33_1uM | 61.66666666666666 | 502.33333333333337 |
| Ng34_1uM | 0.0 | 0.0 |
| Ng34_3uM | 0.0 | 0.0 |
| Ng35_1uM | 852.6666666666666 | 2332.0 |
| Ng36_1uM | 0.0 | 0.0 |
| Ng36_3uM | 0.0 | 0.0 |
| Lg2_HF_10uM | 309.3333333333333 | 0.0 |
| Lg1_1uM | 0.0 | 0.0 |
| Lg2_1uM | 0.0 | 0.0 |
| Lg2_3uM | 0.0 | 0.0 |
| Lg2_10uM | 308.6666666666667 | 0.0 |
| Lg3_1uM | 0.0 | 0.0 |
| Lg4_1uM | 0.0 | 0.0 |
| Lg5_3uM | 0.0 | 713.3333333333334 |
| Lg6_1uM | 0.0 | 0.0 |
| Lg7_1uM | 0.0 | 0.0 |
| Lg7_3uM | 334.0 | 0.0 |
| Lg8_1uM | 0.0 | 0.0 |
| Lg9 | 0.0 | 0.0 |
| Lg10_1uM | 0.0 | 0.0 |
| Lg11_1uM | 442.0 | 0.0 |
| Lg12_1uM | 127.33333333333331 | 0.0 |
| Lg12_3uM | 299.0 | 0.0 |
| Lg13 | 98.66666666666666 | 0.0 |
| Lg14 | 0.0 | 0.0 |
| Lg15 | 0.0 | 0.0 |
| Lg16 | 0.0 | 0.0 |
| Lg17 | 0.0 | 0.0 |
| Lg18_HF_1uM | 71.0 | 0.0 |
| Lg7_HF_3uM | 518.3333333333334 | 0.0 |
| Lg12_HF_3uM | 103.33333333333334 | 0.0 |
| Blank 1 | 0.0 | 0.0 |
| Blank 2 | 0.0 | 0.0 |
| Blank 3 | 0.0 | 0.0 |
| Blank 4 | 0.0 | 0.0 |
| Blank 5 | 0.0 | 0.0 |MFI
### Chart: Bm2-5
| Category | AM 154 (90) IgG4 | AM 154 (92) IgG4 |
|---|---|---|
| Ng1_1uM
 | 0.0 | 0.0 |
| Ng1_3uM
 | 0.0 | 0.0 |
| Ng2_1uM | 0.0 | 0.0 |
| Ng2_3uM | 0.0 | 0.0 |
| Ng2_10uM | 0.0 | 0.0 |
| Ng3_1uM | 0.0 | 0.0 |
| Ng3_3uM | 0.0 | 0.0 |
| Ng3_10uM | 0.0 | 0.0 |
| Ng3_30uM | 0.0 | 0.0 |
| Ng4_1uM | 0.0 | 0.0 |
| Ng4_3uM | 0.0 | 0.0 |
| Ng5_1uM | 0.0 | 0.0 |
| Ng5_3uM | 0.0 | 0.0 |
| Ng6_1uM | 0.0 | 0.0 |
| Ng7_1uM | 0.0 | 0.0 |
| Ng7_3uM | 0.0 | 0.0 |
| Ng8_1uM | 0.0 | 0.0 |
| Ng8_3uM | 0.0 | 0.0 |
| Ng8_10uM | 0.0 | 0.0 |
| Ng9_1uM | 0.0 | 0.0 |
| Ng9_3uM | 0.0 | 0.0 |
| Ng10_3uM | 0.0 | 0.0 |
| Ng10_1uM | 0.0 | 0.0 |
| Ng11 | 0.0 | 0.0 |
| Ng12_1uM | 0.0 | 0.0 |
| Ng12_3uM | 0.0 | 0.0 |
| Ng13_1uM | 0.0 | 0.0 |
| Ng14_1uM | 0.0 | 0.0 |
| Ng15_1uM | 0.0 | 0.0 |
| Ng16_1uM | 0.0 | 0.0 |
| Ng17_1uM | 94.0 | 70.33333333333334 |
| Ng18_1uM | 0.0 | 0.0 |
| Ng18_3uM | 0.0 | 0.0 |
| Ng18_10uM | 135.33333333333331 | 28.0 |
| Ng19_1uM | 0.0 | 0.0 |
| Ng19_3uM | 0.0 | 0.0 |
| Ng19_10uM | 6.0 | 37.66666666666666 |
| Ng20_1uM | 0.0 | 0.0 |
| Ng20_3uM | 58.33333333333334 | 0.0 |
| Ng20_10uM | 199.33333333333331 | 168.0 |
| Ng21_1uM | 0.0 | 0.0 |
| Ng21 _3uM | 0.0 | 0.0 |
| Ng21_10uM | 158.0 | 111.33333333333331 |
| Ng22_1uM | 1548.6666666666667 | 1277.3333333333333 |
| Ng23_1uM | 0.0 | 0.0 |
| Ng24_1uM | 0.0 | 0.0 |
| Ng24_3uM | 0.0 | 0.0 |
| Ng25_1uM | 0.0 | 0.0 |
| Ng25_3uM | 0.0 | 0.0 |
| Ng26_1uM | 140.0 | 0.0 |
| Ng27_1uM | 0.0 | 0.0 |
| Ng27_3uM | 0.0 | 0.0 |
| Ng28_1uM | 0.0 | 0.0 |
| Ng29_1uM | 0.0 | 0.0 |
| Ng30_1uM | 0.0 | 0.0 |
| Ng31_1uM | 0.0 | 0.0 |
| Ng31_3uM | 0.0 | 0.0 |
| Ng31_10uM | 0.0 | 0.0 |
| Ng31_30uM | 0.0 | 0.0 |
| Ng32_1uM | 84.0 | 161.33333333333331 |
| Ng33_1uM | 14.333333333333343 | 70.66666666666666 |
| Ng34_1uM | 0.0 | 0.0 |
| Ng34_3uM | 0.0 | 0.0 |
| Ng35_1uM | 1664.3333333333333 | 1075.6666666666667 |
| Ng36_1uM | 0.0 | 0.0 |
| Ng36_3uM | 0.0 | 0.0 |
| Lg2_HF_10uM | 0.0 | 21.0 |
| Lg1_1uM | 0.0 | 0.0 |
| Lg2_1uM | 0.0 | 0.0 |
| Lg2_3uM | 0.0 | 0.0 |
| Lg2_10uM | 59.33333333333334 | 21.666666666666657 |
| Lg3_1uM | 0.0 | 0.0 |
| Lg4_1uM | 0.0 | 0.0 |
| Lg5_3uM | 0.0 | 0.0 |
| Lg6_1uM | 0.0 | 0.0 |
| Lg7_1uM | 0.0 | 47.33333333333334 |
| Lg7_3uM | 291.3333333333333 | 771.3333333333334 |
| Lg8_1uM | 0.0 | 0.0 |
| Lg9 | 0.0 | 0.0 |
| Lg10_1uM | 0.0 | 0.0 |
| Lg11_1uM | 223.33333333333331 | 231.0 |
| Lg12_1uM | 106.66666666666669 | 71.66666666666666 |
| Lg12_3uM | 738.3333333333334 | 1539.3333333333333 |
| Lg13 | 3.666666666666657 | 48.33333333333334 |
| Lg14 | 64.66666666666666 | 111.66666666666669 |
| Lg15 | 0.0 | 0.0 |
| Lg16 | 0.0 | 0.0 |
| Lg17 | 0.0 | 0.0 |
| Lg18_HF_1uM | 0.0 | 0.0 |
| Lg7_HF_3uM | 351.0 | 250.0 |
| Lg12_HF_3uM | 0.0 | 7.666666666666657 |
| Blank 1 | 0.0 | 0.0 |
| Blank 2 | 0.0 | 0.0 |
| Blank 3 | 0.0 | 0.0 |
| Blank 4 | 0.0 | 0.0 |
| Blank 5 | 0.0 | 0.0 |MFI
Before DEC treatment After DEC treatment

## Slide 23
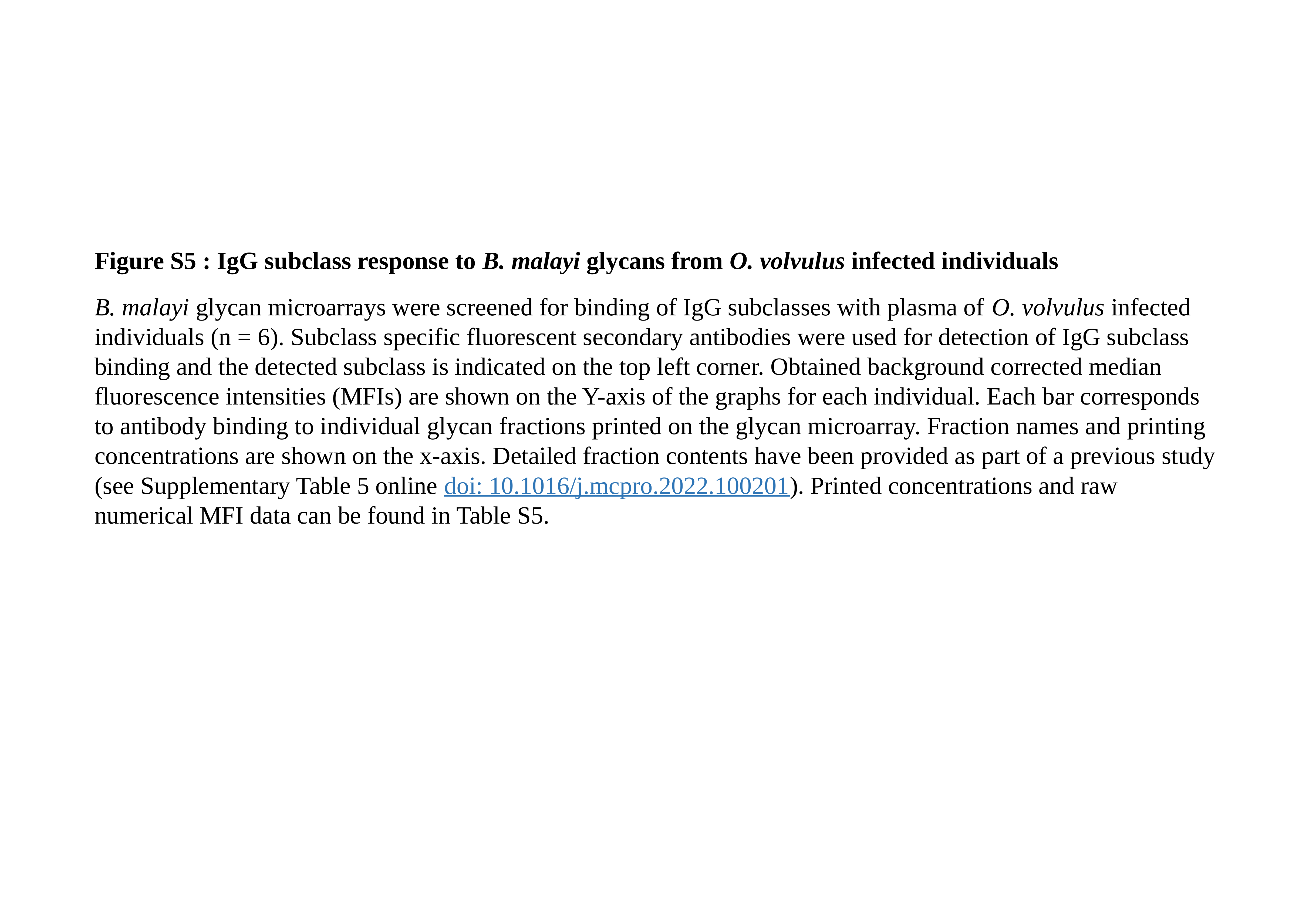

Figure S5 : IgG subclass response to B. malayi glycans from O. volvulus infected individuals
B. malayi glycan microarrays were screened for binding of IgG subclasses with plasma of O. volvulus infected individuals (n = 6). Subclass specific fluorescent secondary antibodies were used for detection of IgG subclass binding and the detected subclass is indicated on the top left corner. Obtained background corrected median fluorescence intensities (MFIs) are shown on the Y-axis of the graphs for each individual. Each bar corresponds to antibody binding to individual glycan fractions printed on the glycan microarray. Fraction names and printing concentrations are shown on the x-axis. Detailed fraction contents have been provided as part of a previous study (see Supplementary Table 5 online doi: 10.1016/j.mcpro.2022.100201). Printed concentrations and raw numerical MFI data can be found in Table S5.

## Slide 24
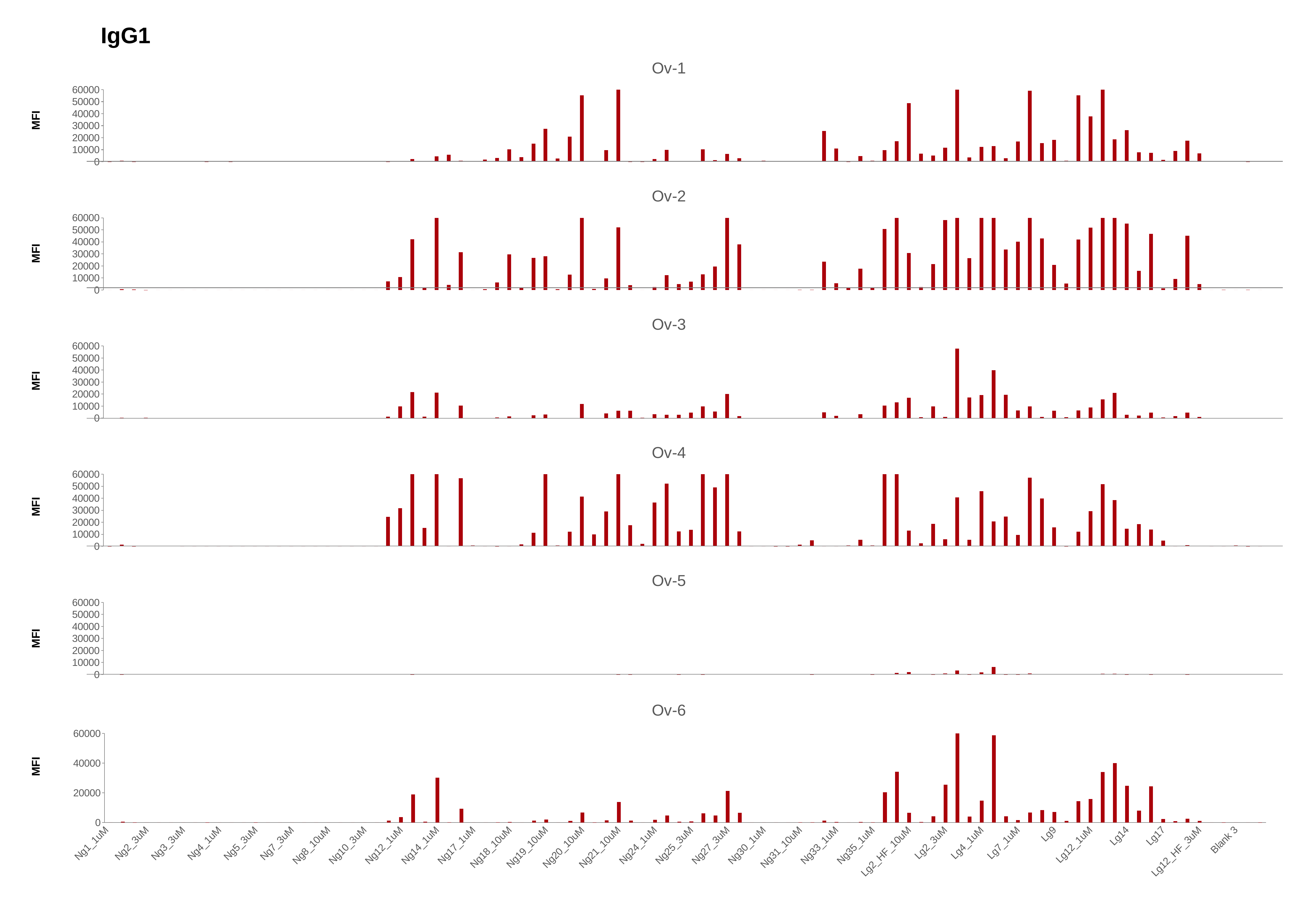

IgG1
### Chart: Ov-1
| Category | Ov 38-207 IgG1 |
|---|---|
| Ng 7-13 1µM_x000d_ | 1.6666666666666288 |
| Ng 7-13 3µM_x000d_ | 924.6666666666666 |
| Ng 9-9 1 µM_x000d_ | 11.333333333333258 |
| Ng 9-9 3µM_x000d_ | 516.6666666666666 |
| Ng 9-9 10µM_x000d_ | 142.33333333333326 |
| Ng 10-13 1 µM_x000d_ | 264.0 |
| Ng 10-13 3 µM_x000d_ | 0.0 |
| Ng 10-13 10 µM_x000d_ | 0.0 |
| Ng 10-13 30 µM_x000d_ | 0.6666666666666288 |
| Ng 10-10 1 µM_x000d_ | 0.0 |
| Ng 10-10 3 µM_x000d_ | 40.33333333333326 |
| Ng 10-11 1 µM_x000d_ | 0.0 |
| Ng 10-11 3 µM_x000d_ | 0.0 |
| Ng 10-12 1 µM_x000d_ | 0.0 |
| Ng 11-10 1 µM_x000d_ | 0.0 |
| Ng 11-10 3 µM_x000d_ | 0.0 |
| Ng 11-11 1 µM_x000d_ | 0.0 |
| Ng 11-11 3µM_x000d_ | 0.0 |
| Ng 11-11 10µM_x000d_ | 0.0 |
| Ng 12-11 1 µM_x000d_ | 0.0 |
| Ng 12-11 3 µM_x000d_ | 0.0 |
| Ng 13-15 1 µM_x000d_ | 0.0 |
| Ng 13-15 3 µM_x000d_ | 0.0 |
| Ng 13-14_x000d_ | 13.666666666666629 |
| Ng 14-10 1 µM_x000d_ | 776.9999999999999 |
| Ng 14-10 3 µM_x000d_ | 2292.6666666666665 |
| Ng 14-11 1 µM_x000d_ | 0.0 |
| Ng 14-12 1 µM_x000d_ | 4472.0 |
| Ng 14-7 1 µM_x000d_ | 5825.333333333334 |
| Ng 15-14/15 1 µM_x000d_ | 958.6666666666666 |
| Ng 15-12 1µM_x000d_ | 0.0 |
| Ng 17-7 1 µM_x000d_ | 1710.0 |
| Ng 17-7 3 µM_x000d_ | 3227.333333333333 |
| Ng 17-7 10 µM_x000d_ | 10328.333333333332 |
| Ng 19-9 1 µM_x000d_ | 3792.3333333333335 |
| Ng 19-9 3 µM_x000d_ | 15035.333333333332 |
| Ng 19-9 10 µM_x000d_ | 27418.666666666668 |
| Ng 20-7 1 µM_x000d_ | 2594.0 |
| Ng 20-7 3 µM_x000d_ | 20980.666666666668 |
| Ng 20-7 10 µM_x000d_ | 55282.0 |
| Ng 21-8 1 µM_x000d_ | 732.3333333333334 |
| Ng 21-8 3 µM_x000d_ | 9613.333333333332 |
| Ng 21-8 10 µM_x000d_ | 62279.0 |
| Ng 15-13 3µM_x000d_ | 74.66666666666663 |
| Ng 16-12 1 µM_x000d_ | 103.0 |
| Ng 17-9 1 µM_x000d_ | 2287.6666666666665 |
| Ng 17-9 3 µM_x000d_ | 9872.666666666666 |
| Ng 18-11 1 µM_x000d_ | 479.66666666666663 |
| Ng 18-11 3 µM_x000d_ | 620.6666666666666 |
| Ng 20-8 1 µM_x000d_ | 10326.666666666666 |
| Ng 20-9 1 µM_x000d_ | 1360.6666666666665 |
| Ng 20-9 3 µM_x000d_ | 6421.0 |
| Ng 21-9 1 µM_x000d_ | 2850.6666666666665 |
| Ng 13-13 3 µM_x000d_ | 0.0 |
| Ng 14-8 1 µM_x000d_ | 826.6666666666666 |
| Ng 14-9 1 µM_x000d_ | 0.0 |
| Ng 14-9 3 µM_x000d_ | 0.0 |
| Ng 14-9 10 µM_x000d_ | 0.0 |
| Ng 14-9 30 µM_x000d_ | 0.0 |
| Ng 15-10 1µM_x000d_ | 25710.0 |
| Ng 16-9 1 µM_x000d_ | 10993.333333333332 |
| Ng 17-8 1 µM_x000d_ | 17.0 |
| Ng 17-8 3 µM_x000d_ | 4756.333333333334 |
| Ng 18-9 1µM_x000d_ | 996.9999999999999 |
| Ng 19-11 1 µM_x000d_ | 9551.333333333332 |
| Ng 19-11 3 µM_x000d_ | 17129.0 |
| Lg 17-15 HF 10 µM_x000d_ | 48779.0 |
| Lg 15-17 1 µM_x000d_ | 6779.0 |
| Lg 17-15 1 µM_x000d_ | 5254.0 |
| Lg 17-15 3 µM_x000d_ | 11716.333333333332 |
| Lg 17-15 10 µM_x000d_ | 63816.666666666664 |
| Lg 17-17 1 µM_x000d_ | 3505.0 |
| Lg 17-16 1 µM_x000d_ | 12329.666666666666 |
| Lg 16-17 3 µM_x000d_ | 13065.0 |
| Lg 18-8 1 µM_x000d_ | 2864.6666666666665 |
| Lg 19-8 1 µM_x000d_ | 16834.0 |
| Lg 19-8 3 µM_x000d_ | 59100.0 |
| Lg 19-9 1 µM_x000d_ | 15577.0 |
| Lg 20-10_x000d_ | 18246.0 |
| Lg 22-12 1 µM_x000d_ | 809.3333333333334 |
| Lg 20-9 1 µM_x000d_ | 55314.0 |
| Lg 23-11 1 µM_x000d_ | 37690.33333333333 |
| Lg 23-11 3 µM_x000d_ | 63088.33333333333 |
| Lg 23-12_x000d_ | 18597.0 |
| Lg 26-8_x000d_ | 26263.666666666668 |
| Lg 26-9_x000d_ | 7771.000000000001 |
| Lg 22-13_x000d_ | 7419.333333333334 |
| Lg 26-10_x000d_ | 1628.333333333333 |
| Lg 18-7-F3 HF 1uM | 9034.0 |
| Lg 19-8-F5 HF 3uM | 17520.0 |
| Lg 23-11-F2 HF 3uM | 7039.0 |
| Blank 1 | 0.0 |
| Blank 2 | 130.0 |
| Blank 3 | 0.0 |
| Blank 4 | 56.66666666666663 |
| Blank 5 | 0.0 |MFI
### Chart: Ov-2
| Category | Ov k44 IgG1 |
|---|---|
| Ng 7-13 1µM_x000d_ | 0.0 |
| Ng 7-13 3µM_x000d_ | 593.3333333333333 |
| Ng 9-9 1 µM_x000d_ | 376.66666666666663 |
| Ng 9-9 3µM_x000d_ | 41.33333333333337 |
| Ng 9-9 10µM_x000d_ | 0.0 |
| Ng 10-13 1 µM_x000d_ | 0.0 |
| Ng 10-13 3 µM_x000d_ | 0.0 |
| Ng 10-13 10 µM_x000d_ | 0.0 |
| Ng 10-13 30 µM_x000d_ | 0.0 |
| Ng 10-10 1 µM_x000d_ | 0.0 |
| Ng 10-10 3 µM_x000d_ | 0.0 |
| Ng 10-11 1 µM_x000d_ | 0.0 |
| Ng 10-11 3 µM_x000d_ | 0.0 |
| Ng 10-12 1 µM_x000d_ | 0.0 |
| Ng 11-10 1 µM_x000d_ | 0.0 |
| Ng 11-10 3 µM_x000d_ | 0.0 |
| Ng 11-11 1 µM_x000d_ | 0.0 |
| Ng 11-11 3µM_x000d_ | 0.0 |
| Ng 11-11 10µM_x000d_ | 0.0 |
| Ng 12-11 1 µM_x000d_ | 0.0 |
| Ng 12-11 3 µM_x000d_ | 0.0 |
| Ng 13-15 1 µM_x000d_ | 0.0 |
| Ng 13-15 3 µM_x000d_ | 0.0 |
| Ng 13-14_x000d_ | 7135.0 |
| Ng 14-10 1 µM_x000d_ | 10816.333333333334 |
| Ng 14-10 3 µM_x000d_ | 42284.333333333336 |
| Ng 14-11 1 µM_x000d_ | 1975.6666666666665 |
| Ng 14-12 1 µM_x000d_ | 64466.0 |
| Ng 14-7 1 µM_x000d_ | 4095.0 |
| Ng 15-14/15 1 µM_x000d_ | 31457.666666666668 |
| Ng 15-12 1µM_x000d_ | 0.0 |
| Ng 17-7 1 µM_x000d_ | 544.6666666666667 |
| Ng 17-7 3 µM_x000d_ | 6226.333333333333 |
| Ng 17-7 10 µM_x000d_ | 29639.333333333332 |
| Ng 19-9 1 µM_x000d_ | 1409.3333333333333 |
| Ng 19-9 3 µM_x000d_ | 26694.333333333332 |
| Ng 19-9 10 µM_x000d_ | 28037.333333333332 |
| Ng 20-7 1 µM_x000d_ | 581.3333333333333 |
| Ng 20-7 3 µM_x000d_ | 12650.0 |
| Ng 20-7 10 µM_x000d_ | 64126.0 |
| Ng 21-8 1 µM_x000d_ | 755.0 |
| Ng 21-8 3 µM_x000d_ | 9536.666666666666 |
| Ng 21-8 10 µM_x000d_ | 52127.333333333336 |
| Ng 15-13 3µM_x000d_ | 4032.666666666667 |
| Ng 16-12 1 µM_x000d_ | 0.0 |
| Ng 17-9 1 µM_x000d_ | 2329.6666666666665 |
| Ng 17-9 3 µM_x000d_ | 12219.0 |
| Ng 18-11 1 µM_x000d_ | 4889.333333333333 |
| Ng 18-11 3 µM_x000d_ | 7018.0 |
| Ng 20-8 1 µM_x000d_ | 12969.333333333334 |
| Ng 20-9 1 µM_x000d_ | 19412.333333333332 |
| Ng 20-9 3 µM_x000d_ | 64549.333333333336 |
| Ng 21-9 1 µM_x000d_ | 37879.666666666664 |
| Ng 13-13 3 µM_x000d_ | 0.0 |
| Ng 14-8 1 µM_x000d_ | 0.0 |
| Ng 14-9 1 µM_x000d_ | 0.0 |
| Ng 14-9 3 µM_x000d_ | 0.0 |
| Ng 14-9 10 µM_x000d_ | 228.33333333333337 |
| Ng 14-9 30 µM_x000d_ | 86.66666666666663 |
| Ng 15-10 1µM_x000d_ | 23462.0 |
| Ng 16-9 1 µM_x000d_ | 5599.666666666667 |
| Ng 17-8 1 µM_x000d_ | 1448.3333333333333 |
| Ng 17-8 3 µM_x000d_ | 17658.0 |
| Ng 18-9 1µM_x000d_ | 1628.3333333333335 |
| Ng 19-11 1 µM_x000d_ | 50746.333333333336 |
| Ng 19-11 3 µM_x000d_ | 63584.0 |
| Lg 17-15 HF 10 µM_x000d_ | 30813.0 |
| Lg 15-17 1 µM_x000d_ | 2407.3333333333335 |
| Lg 17-15 1 µM_x000d_ | 21467.333333333332 |
| Lg 17-15 3 µM_x000d_ | 58134.666666666664 |
| Lg 17-15 10 µM_x000d_ | 64393.666666666664 |
| Lg 17-17 1 µM_x000d_ | 26384.333333333332 |
| Lg 17-16 1 µM_x000d_ | 60876.0 |
| Lg 16-17 3 µM_x000d_ | 64486.333333333336 |
| Lg 18-8 1 µM_x000d_ | 33612.0 |
| Lg 19-8 1 µM_x000d_ | 40183.666666666664 |
| Lg 19-8 3 µM_x000d_ | 63898.0 |
| Lg 19-9 1 µM_x000d_ | 42766.333333333336 |
| Lg 20-10_x000d_ | 20881.666666666668 |
| Lg 22-12 1 µM_x000d_ | 5244.666666666667 |
| Lg 20-9 1 µM_x000d_ | 42054.0 |
| Lg 23-11 1 µM_x000d_ | 51842.0 |
| Lg 23-11 3 µM_x000d_ | 64444.333333333336 |
| Lg 23-12_x000d_ | 64588.0 |
| Lg 26-8_x000d_ | 55325.666666666664 |
| Lg 26-9_x000d_ | 15915.666666666668 |
| Lg 22-13_x000d_ | 46701.666666666664 |
| Lg 26-10_x000d_ | 1306.6666666666667 |
| Lg 18-7-F3 HF 1uM | 9253.0 |
| Lg 19-8-F5 HF 3uM | 45111.0 |
| Lg 23-11-F2 HF 3uM | 4915.333333333333 |
| Blank 1 | 0.0 |
| Blank 2 | 79.33333333333337 |
| Blank 3 | 0.0 |
| Blank 4 | 55.0 |
| Blank 5 | 0.0 |MFI
### Chart: Ov-3
| Category | Ov k84 IgG1 |
|---|---|
| Ng 7-13 1µM_x000d_ | 71.33333333333334 |
| Ng 7-13 3µM_x000d_ | 311.6666666666667 |
| Ng 9-9 1 µM_x000d_ | 3.666666666666657 |
| Ng 9-9 3µM_x000d_ | 364.33333333333337 |
| Ng 9-9 10µM_x000d_ | 88.0 |
| Ng 10-13 1 µM_x000d_ | 0.0 |
| Ng 10-13 3 µM_x000d_ | 0.0 |
| Ng 10-13 10 µM_x000d_ | 28.666666666666657 |
| Ng 10-13 30 µM_x000d_ | 3.666666666666657 |
| Ng 10-10 1 µM_x000d_ | 15.0 |
| Ng 10-10 3 µM_x000d_ | 62.66666666666666 |
| Ng 10-11 1 µM_x000d_ | 46.0 |
| Ng 10-11 3 µM_x000d_ | 41.66666666666666 |
| Ng 10-12 1 µM_x000d_ | 28.333333333333343 |
| Ng 11-10 1 µM_x000d_ | 6.666666666666657 |
| Ng 11-10 3 µM_x000d_ | 54.66666666666666 |
| Ng 11-11 1 µM_x000d_ | 26.0 |
| Ng 11-11 3µM_x000d_ | 39.0 |
| Ng 11-11 10µM_x000d_ | 0.0 |
| Ng 12-11 1 µM_x000d_ | 40.0 |
| Ng 12-11 3 µM_x000d_ | 41.33333333333334 |
| Ng 13-15 1 µM_x000d_ | 3.666666666666657 |
| Ng 13-15 3 µM_x000d_ | 72.33333333333334 |
| Ng 13-14_x000d_ | 1241.0 |
| Ng 14-10 1 µM_x000d_ | 9775.0 |
| Ng 14-10 3 µM_x000d_ | 21617.666666666668 |
| Ng 14-11 1 µM_x000d_ | 1173.0 |
| Ng 14-12 1 µM_x000d_ | 21166.0 |
| Ng 14-7 1 µM_x000d_ | 134.0 |
| Ng 15-14/15 1 µM_x000d_ | 10402.0 |
| Ng 15-12 1µM_x000d_ | 5.666666666666657 |
| Ng 17-7 1 µM_x000d_ | 90.66666666666666 |
| Ng 17-7 3 µM_x000d_ | 603.6666666666666 |
| Ng 17-7 10 µM_x000d_ | 1424.3333333333333 |
| Ng 19-9 1 µM_x000d_ | 22.0 |
| Ng 19-9 3 µM_x000d_ | 2439.0 |
| Ng 19-9 10 µM_x000d_ | 2911.3333333333335 |
| Ng 20-7 1 µM_x000d_ | 4.333333333333343 |
| Ng 20-7 3 µM_x000d_ | 63.0 |
| Ng 20-7 10 µM_x000d_ | 11814.0 |
| Ng 21-8 1 µM_x000d_ | 181.33333333333331 |
| Ng 21-8 3 µM_x000d_ | 3880.6666666666665 |
| Ng 21-8 10 µM_x000d_ | 6082.666666666667 |
| Ng 15-13 3µM_x000d_ | 6178.333333333333 |
| Ng 16-12 1 µM_x000d_ | 245.0 |
| Ng 17-9 1 µM_x000d_ | 3212.6666666666665 |
| Ng 17-9 3 µM_x000d_ | 2749.0 |
| Ng 18-11 1 µM_x000d_ | 2849.3333333333335 |
| Ng 18-11 3 µM_x000d_ | 4607.666666666667 |
| Ng 20-8 1 µM_x000d_ | 9715.333333333334 |
| Ng 20-9 1 µM_x000d_ | 5404.666666666667 |
| Ng 20-9 3 µM_x000d_ | 20158.0 |
| Ng 21-9 1 µM_x000d_ | 1603.0 |
| Ng 13-13 3 µM_x000d_ | 107.0 |
| Ng 14-8 1 µM_x000d_ | 10.0 |
| Ng 14-9 1 µM_x000d_ | 0.0 |
| Ng 14-9 3 µM_x000d_ | 86.33333333333334 |
| Ng 14-9 10 µM_x000d_ | 190.66666666666669 |
| Ng 14-9 30 µM_x000d_ | 147.66666666666669 |
| Ng 15-10 1µM_x000d_ | 4866.333333333333 |
| Ng 16-9 1 µM_x000d_ | 1840.0 |
| Ng 17-8 1 µM_x000d_ | 43.33333333333334 |
| Ng 17-8 3 µM_x000d_ | 3313.6666666666665 |
| Ng 18-9 1µM_x000d_ | 188.0 |
| Ng 19-11 1 µM_x000d_ | 10409.666666666666 |
| Ng 19-11 3 µM_x000d_ | 13046.0 |
| Lg 17-15 HF 10 µM_x000d_ | 16928.0 |
| Lg 15-17 1 µM_x000d_ | 707.3333333333334 |
| Lg 17-15 1 µM_x000d_ | 9743.666666666666 |
| Lg 17-15 3 µM_x000d_ | 883.3333333333333 |
| Lg 17-15 10 µM_x000d_ | 57918.666666666664 |
| Lg 17-17 1 µM_x000d_ | 17230.333333333332 |
| Lg 17-16 1 µM_x000d_ | 19220.666666666668 |
| Lg 16-17 3 µM_x000d_ | 39887.0 |
| Lg 18-8 1 µM_x000d_ | 19417.0 |
| Lg 19-8 1 µM_x000d_ | 6354.0 |
| Lg 19-8 3 µM_x000d_ | 9649.666666666666 |
| Lg 19-9 1 µM_x000d_ | 908.0 |
| Lg 20-10_x000d_ | 6078.0 |
| Lg 22-12 1 µM_x000d_ | 712.0 |
| Lg 20-9 1 µM_x000d_ | 6291.0 |
| Lg 23-11 1 µM_x000d_ | 8744.0 |
| Lg 23-11 3 µM_x000d_ | 15660.333333333334 |
| Lg 23-12_x000d_ | 20939.333333333332 |
| Lg 26-8_x000d_ | 2850.0 |
| Lg 26-9_x000d_ | 2098.0 |
| Lg 22-13_x000d_ | 4661.0 |
| Lg 26-10_x000d_ | 454.0 |
| Lg 18-7-F3 HF 1uM | 1690.6666666666667 |
| Lg 19-8-F5 HF 3uM | 4624.0 |
| Lg 23-11-F2 HF 3uM | 939.0 |
| Blank 1 | 0.0 |
| Blank 2 | 0.0 |
| Blank 3 | 0.0 |
| Blank 4 | 0.0 |
| Blank 5 | 0.0 |MFI
### Chart: Ov-4
| Category | Ov k-96 IgG1 |
|---|---|
| Ng 7-13 1µM_x000d_ | 7.3333333333332575 |
| Ng 7-13 3µM_x000d_ | 1356.1666666666665 |
| Ng 9-9 1 µM_x000d_ | 109.33333333333326 |
| Ng 9-9 3µM_x000d_ | 573.9999999999999 |
| Ng 9-9 10µM_x000d_ | 205.0 |
| Ng 10-13 1 µM_x000d_ | 218.33333333333326 |
| Ng 10-13 3 µM_x000d_ | 0.0 |
| Ng 10-13 10 µM_x000d_ | 0.0 |
| Ng 10-13 30 µM_x000d_ | 0.0 |
| Ng 10-10 1 µM_x000d_ | 0.0 |
| Ng 10-10 3 µM_x000d_ | 0.0 |
| Ng 10-11 1 µM_x000d_ | 0.0 |
| Ng 10-11 3 µM_x000d_ | 0.0 |
| Ng 10-12 1 µM_x000d_ | 0.0 |
| Ng 11-10 1 µM_x000d_ | 0.0 |
| Ng 11-10 3 µM_x000d_ | 0.0 |
| Ng 11-11 1 µM_x000d_ | 0.0 |
| Ng 11-11 3µM_x000d_ | 0.0 |
| Ng 11-11 10µM_x000d_ | 0.0 |
| Ng 12-11 1 µM_x000d_ | 0.0 |
| Ng 12-11 3 µM_x000d_ | 0.0 |
| Ng 13-15 1 µM_x000d_ | 0.0 |
| Ng 13-15 3 µM_x000d_ | 0.0 |
| Ng 13-14_x000d_ | 24565.0 |
| Ng 14-10 1 µM_x000d_ | 31795.333333333336 |
| Ng 14-10 3 µM_x000d_ | 64262.666666666664 |
| Ng 14-11 1 µM_x000d_ | 15235.0 |
| Ng 14-12 1 µM_x000d_ | 63966.33333333333 |
| Ng 14-7 1 µM_x000d_ | 0.0 |
| Ng 15-14/15 1 µM_x000d_ | 56540.666666666664 |
| Ng 15-12 1µM_x000d_ | 807.6666666666666 |
| Ng 17-7 1 µM_x000d_ | 0.0 |
| Ng 17-7 3 µM_x000d_ | 56.66666666666663 |
| Ng 17-7 10 µM_x000d_ | 0.0 |
| Ng 19-9 1 µM_x000d_ | 1554.6666666666665 |
| Ng 19-9 3 µM_x000d_ | 11257.333333333332 |
| Ng 19-9 10 µM_x000d_ | 61898.666666666664 |
| Ng 20-7 1 µM_x000d_ | 698.6666666666666 |
| Ng 20-7 3 µM_x000d_ | 12155.333333333332 |
| Ng 20-7 10 µM_x000d_ | 41319.666666666664 |
| Ng 21-8 1 µM_x000d_ | 9811.666666666666 |
| Ng 21-8 3 µM_x000d_ | 29094.166666666668 |
| Ng 21-8 10 µM_x000d_ | 64276.666666666664 |
| Ng 15-13 3µM_x000d_ | 17472.333333333336 |
| Ng 16-12 1 µM_x000d_ | 2043.6666666666665 |
| Ng 17-9 1 µM_x000d_ | 36486.33333333333 |
| Ng 17-9 3 µM_x000d_ | 52176.33333333333 |
| Ng 18-11 1 µM_x000d_ | 12459.0 |
| Ng 18-11 3 µM_x000d_ | 13792.333333333332 |
| Ng 20-8 1 µM_x000d_ | 64440.0 |
| Ng 20-9 1 µM_x000d_ | 48929.0 |
| Ng 20-9 3 µM_x000d_ | 64182.0 |
| Ng 21-9 1 µM_x000d_ | 12400.0 |
| Ng 13-13 3 µM_x000d_ | 0.0 |
| Ng 14-8 1 µM_x000d_ | 0.0 |
| Ng 14-9 1 µM_x000d_ | 65.0 |
| Ng 14-9 3 µM_x000d_ | 113.66666666666663 |
| Ng 14-9 10 µM_x000d_ | 1349.0 |
| Ng 14-9 30 µM_x000d_ | 4863.666666666667 |
| Ng 15-10 1µM_x000d_ | 0.0 |
| Ng 16-9 1 µM_x000d_ | 0.0 |
| Ng 17-8 1 µM_x000d_ | 776.3333333333334 |
| Ng 17-8 3 µM_x000d_ | 5365.0 |
| Ng 18-9 1µM_x000d_ | 629.9999999999999 |
| Ng 19-11 1 µM_x000d_ | 64418.666666666664 |
| Ng 19-11 3 µM_x000d_ | 63925.0 |
| Lg 17-15 HF 10 µM_x000d_ | 13104.0 |
| Lg 15-17 1 µM_x000d_ | 2474.0 |
| Lg 17-15 1 µM_x000d_ | 18715.0 |
| Lg 17-15 3 µM_x000d_ | 5813.0 |
| Lg 17-15 10 µM_x000d_ | 40723.33333333333 |
| Lg 17-17 1 µM_x000d_ | 5432.666666666667 |
| Lg 17-16 1 µM_x000d_ | 45854.666666666664 |
| Lg 16-17 3 µM_x000d_ | 20683.0 |
| Lg 18-8 1 µM_x000d_ | 24725.0 |
| Lg 19-8 1 µM_x000d_ | 9465.666666666666 |
| Lg 19-8 3 µM_x000d_ | 57088.0 |
| Lg 19-9 1 µM_x000d_ | 39757.0 |
| Lg 20-10_x000d_ | 15799.999999999998 |
| Lg 22-12 1 µM_x000d_ | 44.0 |
| Lg 20-9 1 µM_x000d_ | 12119.333333333332 |
| Lg 23-11 1 µM_x000d_ | 29272.0 |
| Lg 23-11 3 µM_x000d_ | 51742.0 |
| Lg 23-12_x000d_ | 38370.666666666664 |
| Lg 26-8_x000d_ | 14623.0 |
| Lg 26-9_x000d_ | 18513.0 |
| Lg 22-13_x000d_ | 13879.0 |
| Lg 26-10_x000d_ | 4767.333333333334 |
| Lg 18-7-F3 HF 1uM | 0.0 |
| Lg 19-8-F5 HF 3uM | 851.9999999999999 |
| Lg 23-11-F2 HF 3uM | 142.33333333333326 |
| Blank 1 | 0.0 |
| Blank 2 | 0.0 |
| Blank 3 | 643.6666666666666 |
| Blank 4 | 113.66666666666663 |
| Blank 5 | 0.0 |MFI
### Chart: Ov-5
| Category | Ov k-99 IgG1 |
|---|---|
| Ng 7-13 1µM_x000d_ | 0.0 |
| Ng 7-13 3µM_x000d_ | 19.0 |
| Ng 9-9 1 µM_x000d_ | 0.0 |
| Ng 9-9 3µM_x000d_ | 73.0 |
| Ng 9-9 10µM_x000d_ | 0.0 |
| Ng 10-13 1 µM_x000d_ | 0.0 |
| Ng 10-13 3 µM_x000d_ | 0.0 |
| Ng 10-13 10 µM_x000d_ | 0.0 |
| Ng 10-13 30 µM_x000d_ | 0.0 |
| Ng 10-10 1 µM_x000d_ | 0.0 |
| Ng 10-10 3 µM_x000d_ | 0.0 |
| Ng 10-11 1 µM_x000d_ | 0.0 |
| Ng 10-11 3 µM_x000d_ | 0.0 |
| Ng 10-12 1 µM_x000d_ | 0.0 |
| Ng 11-10 1 µM_x000d_ | 0.0 |
| Ng 11-10 3 µM_x000d_ | 0.0 |
| Ng 11-11 1 µM_x000d_ | 0.0 |
| Ng 11-11 3µM_x000d_ | 0.0 |
| Ng 11-11 10µM_x000d_ | 0.0 |
| Ng 12-11 1 µM_x000d_ | 0.0 |
| Ng 12-11 3 µM_x000d_ | 0.0 |
| Ng 13-15 1 µM_x000d_ | 0.0 |
| Ng 13-15 3 µM_x000d_ | 0.0 |
| Ng 13-14_x000d_ | 0.0 |
| Ng 14-10 1 µM_x000d_ | 0.0 |
| Ng 14-10 3 µM_x000d_ | 8.0 |
| Ng 14-11 1 µM_x000d_ | 0.0 |
| Ng 14-12 1 µM_x000d_ | 120.0 |
| Ng 14-7 1 µM_x000d_ | 0.0 |
| Ng 15-14/15 1 µM_x000d_ | 0.0 |
| Ng 15-12 1µM_x000d_ | 0.0 |
| Ng 17-7 1 µM_x000d_ | 0.0 |
| Ng 17-7 3 µM_x000d_ | 0.0 |
| Ng 17-7 10 µM_x000d_ | 0.0 |
| Ng 19-9 1 µM_x000d_ | 0.0 |
| Ng 19-9 3 µM_x000d_ | 0.0 |
| Ng 19-9 10 µM_x000d_ | 0.0 |
| Ng 20-7 1 µM_x000d_ | 0.0 |
| Ng 20-7 3 µM_x000d_ | 0.0 |
| Ng 20-7 10 µM_x000d_ | 127.66666666666669 |
| Ng 21-8 1 µM_x000d_ | 0.0 |
| Ng 21-8 3 µM_x000d_ | 0.0 |
| Ng 21-8 10 µM_x000d_ | 55.0 |
| Ng 15-13 3µM_x000d_ | 9.666666666666657 |
| Ng 16-12 1 µM_x000d_ | 0.0 |
| Ng 17-9 1 µM_x000d_ | 0.0 |
| Ng 17-9 3 µM_x000d_ | 0.0 |
| Ng 18-11 1 µM_x000d_ | 60.66666666666666 |
| Ng 18-11 3 µM_x000d_ | 143.66666666666669 |
| Ng 20-8 1 µM_x000d_ | 4.666666666666657 |
| Ng 20-9 1 µM_x000d_ | 0.0 |
| Ng 20-9 3 µM_x000d_ | 145.66666666666669 |
| Ng 21-9 1 µM_x000d_ | 0.0 |
| Ng 13-13 3 µM_x000d_ | 0.0 |
| Ng 14-8 1 µM_x000d_ | 0.0 |
| Ng 14-9 1 µM_x000d_ | 0.0 |
| Ng 14-9 3 µM_x000d_ | 0.0 |
| Ng 14-9 10 µM_x000d_ | 0.0 |
| Ng 14-9 30 µM_x000d_ | 2.0 |
| Ng 15-10 1µM_x000d_ | 0.0 |
| Ng 16-9 1 µM_x000d_ | 0.0 |
| Ng 17-8 1 µM_x000d_ | 0.0 |
| Ng 17-8 3 µM_x000d_ | 0.0 |
| Ng 18-9 1µM_x000d_ | 52.33333333333334 |
| Ng 19-11 1 µM_x000d_ | 398.66666666666663 |
| Ng 19-11 3 µM_x000d_ | 1338.3333333333333 |
| Lg 17-15 HF 10 µM_x000d_ | 2021.6666666666665 |
| Lg 15-17 1 µM_x000d_ | 0.0 |
| Lg 17-15 1 µM_x000d_ | 46.0 |
| Lg 17-15 3 µM_x000d_ | 924.3333333333333 |
| Lg 17-15 10 µM_x000d_ | 3407.6666666666665 |
| Lg 17-17 1 µM_x000d_ | 7.0 |
| Lg 17-16 1 µM_x000d_ | 1677.0 |
| Lg 16-17 3 µM_x000d_ | 6337.666666666667 |
| Lg 18-8 1 µM_x000d_ | 33.33333333333334 |
| Lg 19-8 1 µM_x000d_ | 30.0 |
| Lg 19-8 3 µM_x000d_ | 899.6666666666667 |
| Lg 19-9 1 µM_x000d_ | 126.0 |
| Lg 20-10_x000d_ | 0.0 |
| Lg 22-12 1 µM_x000d_ | 0.0 |
| Lg 20-9 1 µM_x000d_ | 133.66666666666669 |
| Lg 23-11 1 µM_x000d_ | 82.66666666666666 |
| Lg 23-11 3 µM_x000d_ | 722.0 |
| Lg 23-12_x000d_ | 516.0 |
| Lg 26-8_x000d_ | 8.0 |
| Lg 26-9_x000d_ | 0.0 |
| Lg 22-13_x000d_ | 16.666666666666657 |
| Lg 26-10_x000d_ | 0.0 |
| Lg 18-7-F3 HF 1uM | 0.0 |
| Lg 19-8-F5 HF 3uM | 58.33333333333334 |
| Lg 23-11-F2 HF 3uM | 0.0 |
| Blank 1 | 0.0 |
| Blank 2 | 0.0 |
| Blank 3 | 0.0 |
| Blank 4 | 0.0 |
| Blank 5 | 0.0 |MFI
### Chart: Ov-6
| Category | Ov k-100 IgG1 |
|---|---|
| Ng1_1uM
 | 0.0 |
| Ng1_3uM
 | 561.6666666666667 |
| Ng2_1uM | 1.6666666666666856 |
| Ng2_3uM | 0.0 |
| Ng2_10uM | 0.0 |
| Ng3_1uM | 0.0 |
| Ng3_3uM | 0.0 |
| Ng3_10uM | 0.0 |
| Ng3_30uM | 6.333333333333371 |
| Ng4_1uM | 0.0 |
| Ng4_3uM | 0.0 |
| Ng5_1uM | 0.0 |
| Ng5_3uM | 26.666666666666686 |
| Ng6_1uM | 0.0 |
| Ng7_1uM | 0.0 |
| Ng7_3uM | 0.0 |
| Ng8_1uM | 0.0 |
| Ng8_3uM | 0.0 |
| Ng8_10uM | 0.0 |
| Ng9_1uM | 0.0 |
| Ng9_3uM | 0.0 |
| Ng10_3uM | 0.0 |
| Ng10_1uM | 0.0 |
| Ng11 | 1212.6666666666667 |
| Ng12_1uM | 3569.0 |
| Ng12_3uM | 18887.333333333336 |
| Ng13_1uM | 548.6666666666667 |
| Ng14_1uM | 30092.666666666668 |
| Ng15_1uM | 88.0 |
| Ng16_1uM | 9302.333333333332 |
| Ng17_1uM | 0.0 |
| Ng18_1uM | 0.0 |
| Ng18_3uM | 108.66666666666669 |
| Ng18_10uM | 419.6666666666667 |
| Ng19_1uM | 0.0 |
| Ng19_3uM | 1198.0 |
| Ng19_10uM | 1991.0000000000002 |
| Ng20_1uM | 0.0 |
| Ng20_3uM | 1043.3333333333335 |
| Ng20_10uM | 6693.666666666667 |
| Ng21_1uM | 35.666666666666686 |
| Ng21 _3uM | 1381.6666666666667 |
| Ng21_10uM | 13784.0 |
| Ng22_1uM | 1260.0 |
| Ng23_1uM | 0.0 |
| Ng24_1uM | 1710.3333333333335 |
| Ng24_3uM | 4682.333333333334 |
| Ng25_1uM | 576.0 |
| Ng25_3uM | 790.3333333333335 |
| Ng26_1uM | 6119.0 |
| Ng27_1uM | 4783.666666666667 |
| Ng27_3uM | 21271.0 |
| Ng28_1uM | 6517.333333333334 |
| Ng29_1uM | 0.0 |
| Ng30_1uM | 0.0 |
| Ng31_1uM | 0.0 |
| Ng31_3uM | 0.0 |
| Ng31_10uM | 70.0 |
| Ng31_30uM | 107.33333333333337 |
| Ng32_1uM | 1219.3333333333335 |
| Ng33_1uM | 395.3333333333333 |
| Ng34_1uM | 0.0 |
| Ng34_3uM | 264.00000000000006 |
| Ng35_1uM | 78.66666666666669 |
| Ng36_1uM | 20282.0 |
| Ng36_3uM | 34269.0 |
| Lg2_HF_10uM | 6446.666666666667 |
| Lg1_1uM | 284.3333333333333 |
| Lg2_1uM | 4218.333333333334 |
| Lg2_3uM | 25458.666666666668 |
| Lg2_10uM | 64815.33333333333 |
| Lg3_1uM | 4003.3333333333335 |
| Lg4_1uM | 14767.333333333332 |
| Lg5_3uM | 58817.0 |
| Lg6_1uM | 4094.9999999999995 |
| Lg7_1uM | 1687.6666666666667 |
| Lg7_3uM | 6653.666666666667 |
| Lg8_1uM | 8281.0 |
| Lg9 | 7021.333333333334 |
| Lg10_1uM | 1054.0 |
| Lg11_1uM | 14283.0 |
| Lg12_1uM | 15864.0 |
| Lg12_3uM | 34080.0 |
| Lg13 | 40040.666666666664 |
| Lg14 | 24635.666666666668 |
| Lg15 | 7957.666666666667 |
| Lg16 | 24381.0 |
| Lg17 | 2262.6666666666665 |
| Lg18_HF_1uM | 845.6666666666667 |
| Lg7_HF_3uM | 2568.0 |
| Lg12_HF_3uM | 995.6666666666667 |
| Blank 1 | 0.0 |
| Blank 2 | 43.666666666666686 |
| Blank 3 | 0.0 |
| Blank 4 | 0.0 |
| Blank 5 | 10.666666666666686 |MFI

## Slide 25
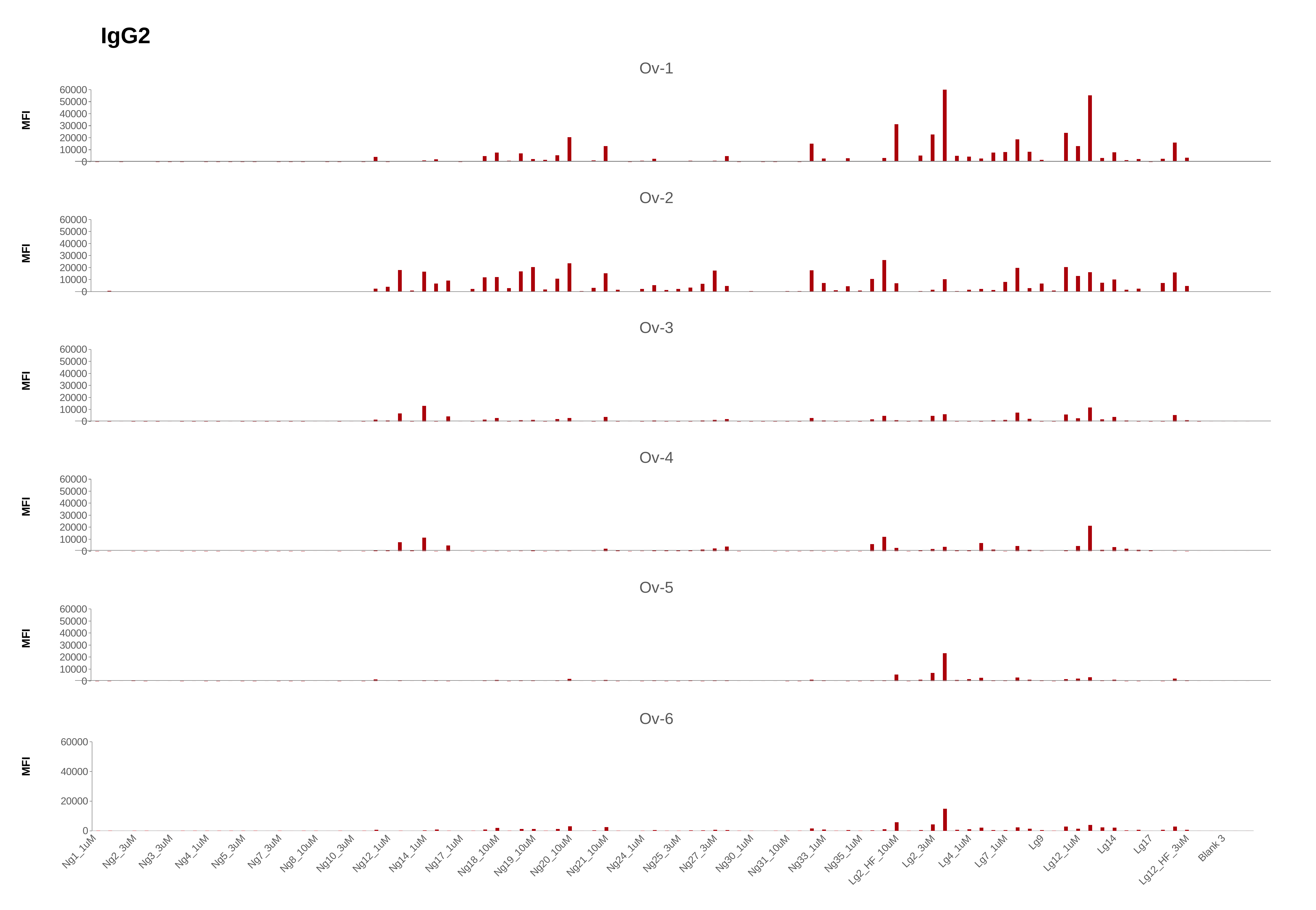

IgG2
### Chart: Ov-1
| Category | Ov 38-207 IgG2 |
|---|---|
| Ng 7-13 1µM_x000d_ | 80.66666666666666 |
| Ng 7-13 3µM_x000d_ | 647.6666666666666 |
| Ng 9-9 1 µM_x000d_ | 104.0 |
| Ng 9-9 3µM_x000d_ | 304.3333333333333 |
| Ng 9-9 10µM_x000d_ | 212.33333333333331 |
| Ng 10-13 1 µM_x000d_ | 13.0 |
| Ng 10-13 3 µM_x000d_ | 22.666666666666657 |
| Ng 10-13 10 µM_x000d_ | 66.66666666666666 |
| Ng 10-13 30 µM_x000d_ | 125.0 |
| Ng 10-10 1 µM_x000d_ | 54.33333333333334 |
| Ng 10-10 3 µM_x000d_ | 40.0 |
| Ng 10-11 1 µM_x000d_ | 79.0 |
| Ng 10-11 3 µM_x000d_ | 20.666666666666657 |
| Ng 10-12 1 µM_x000d_ | 39.33333333333334 |
| Ng 11-10 1 µM_x000d_ | 0.0 |
| Ng 11-10 3 µM_x000d_ | 82.33333333333334 |
| Ng 11-11 1 µM_x000d_ | 48.66666666666666 |
| Ng 11-11 3µM_x000d_ | 57.33333333333334 |
| Ng 11-11 10µM_x000d_ | 0.0 |
| Ng 12-11 1 µM_x000d_ | 37.0 |
| Ng 12-11 3 µM_x000d_ | 46.0 |
| Ng 13-15 1 µM_x000d_ | 0.0 |
| Ng 13-15 3 µM_x000d_ | 52.0 |
| Ng 13-14_x000d_ | 3951.333333333333 |
| Ng 14-10 1 µM_x000d_ | 91.33333333333334 |
| Ng 14-10 3 µM_x000d_ | 246.66666666666669 |
| Ng 14-11 1 µM_x000d_ | 0.0 |
| Ng 14-12 1 µM_x000d_ | 1200.6666666666667 |
| Ng 14-7 1 µM_x000d_ | 2106.3333333333335 |
| Ng 15-14/15 1 µM_x000d_ | 272.3333333333333 |
| Ng 15-12 1µM_x000d_ | 32.33333333333334 |
| Ng 17-7 1 µM_x000d_ | 266.0 |
| Ng 17-7 3 µM_x000d_ | 4666.666666666667 |
| Ng 17-7 10 µM_x000d_ | 7537.666666666667 |
| Ng 19-9 1 µM_x000d_ | 808.0 |
| Ng 19-9 3 µM_x000d_ | 6881.333333333333 |
| Ng 19-9 10 µM_x000d_ | 2338.6666666666665 |
| Ng 20-7 1 µM_x000d_ | 1571.0 |
| Ng 20-7 3 µM_x000d_ | 5462.0 |
| Ng 20-7 10 µM_x000d_ | 20506.0 |
| Ng 21-8 1 µM_x000d_ | 171.66666666666669 |
| Ng 21-8 3 µM_x000d_ | 1028.6666666666667 |
| Ng 21-8 10 µM_x000d_ | 13127.666666666666 |
| Ng 15-13 3µM_x000d_ | 289.6666666666667 |
| Ng 16-12 1 µM_x000d_ | 0.6666666666666572 |
| Ng 17-9 1 µM_x000d_ | 855.0 |
| Ng 17-9 3 µM_x000d_ | 2534.0 |
| Ng 18-11 1 µM_x000d_ | 310.3333333333333 |
| Ng 18-11 3 µM_x000d_ | 307.0 |
| Ng 20-8 1 µM_x000d_ | 783.6666666666666 |
| Ng 20-9 1 µM_x000d_ | 700.6666666666666 |
| Ng 20-9 3 µM_x000d_ | 968.3333333333333 |
| Ng 21-9 1 µM_x000d_ | 4693.666666666667 |
| Ng 13-13 3 µM_x000d_ | 42.33333333333334 |
| Ng 14-8 1 µM_x000d_ | 432.0 |
| Ng 14-9 1 µM_x000d_ | 7.333333333333343 |
| Ng 14-9 3 µM_x000d_ | 53.0 |
| Ng 14-9 10 µM_x000d_ | 211.33333333333331 |
| Ng 14-9 30 µM_x000d_ | 71.0 |
| Ng 15-10 1µM_x000d_ | 15041.0 |
| Ng 16-9 1 µM_x000d_ | 2662.3333333333335 |
| Ng 17-8 1 µM_x000d_ | 300.3333333333333 |
| Ng 17-8 3 µM_x000d_ | 2900.3333333333335 |
| Ng 18-9 1µM_x000d_ | 391.66666666666663 |
| Ng 19-11 1 µM_x000d_ | 675.3333333333334 |
| Ng 19-11 3 µM_x000d_ | 3129.0 |
| Lg 17-15 HF 10 µM_x000d_ | 31243.333333333332 |
| Lg 15-17 1 µM_x000d_ | 272.6666666666667 |
| Lg 17-15 1 µM_x000d_ | 5110.666666666667 |
| Lg 17-15 3 µM_x000d_ | 22606.333333333332 |
| Lg 17-15 10 µM_x000d_ | 64866.333333333336 |
| Lg 17-17 1 µM_x000d_ | 5012.0 |
| Lg 17-16 1 µM_x000d_ | 4366.333333333333 |
| Lg 16-17 3 µM_x000d_ | 2705.3333333333335 |
| Lg 18-8 1 µM_x000d_ | 7569.0 |
| Lg 19-8 1 µM_x000d_ | 8061.666666666666 |
| Lg 19-8 3 µM_x000d_ | 18734.0 |
| Lg 19-9 1 µM_x000d_ | 8384.666666666666 |
| Lg 20-10_x000d_ | 1668.3333333333333 |
| Lg 22-12 1 µM_x000d_ | 367.0 |
| Lg 20-9 1 µM_x000d_ | 24071.666666666668 |
| Lg 23-11 1 µM_x000d_ | 13017.333333333334 |
| Lg 23-11 3 µM_x000d_ | 55318.0 |
| Lg 23-12_x000d_ | 3164.0 |
| Lg 26-8_x000d_ | 7788.0 |
| Lg 26-9_x000d_ | 1332.6666666666667 |
| Lg 22-13_x000d_ | 2335.0 |
| Lg 26-10_x000d_ | 35.66666666666666 |
| Lg 18-7-F3 HF 1uM | 2495.0 |
| Lg 19-8-F5 HF 3uM | 15833.333333333334 |
| Lg 23-11-F2 HF 3uM | 3415.3333333333335 |
| Blank 1 | 0.0 |
| Blank 2 | 0.0 |
| Blank 3 | 0.0 |
| Blank 4 | 0.0 |
| Blank 5 | 0.0 |MFI
### Chart: Ov-2
| Category | Ov k44 IgG2 |
|---|---|
| Ng 7-13 1µM_x000d_ | 78.0 |
| Ng 7-13 3µM_x000d_ | 636.0 |
| Ng 9-9 1 µM_x000d_ | 48.0 |
| Ng 9-9 3µM_x000d_ | 178.00000000000003 |
| Ng 9-9 10µM_x000d_ | 96.66666666666669 |
| Ng 10-13 1 µM_x000d_ | 0.0 |
| Ng 10-13 3 µM_x000d_ | 0.0 |
| Ng 10-13 10 µM_x000d_ | 0.0 |
| Ng 10-13 30 µM_x000d_ | 0.0 |
| Ng 10-10 1 µM_x000d_ | 3.6666666666666856 |
| Ng 10-10 3 µM_x000d_ | 7.0 |
| Ng 10-11 1 µM_x000d_ | 2.6666666666666856 |
| Ng 10-11 3 µM_x000d_ | 33.666666666666686 |
| Ng 10-12 1 µM_x000d_ | 0.0 |
| Ng 11-10 1 µM_x000d_ | 20.333333333333343 |
| Ng 11-10 3 µM_x000d_ | 0.0 |
| Ng 11-11 1 µM_x000d_ | 0.0 |
| Ng 11-11 3µM_x000d_ | 0.0 |
| Ng 11-11 10µM_x000d_ | 0.0 |
| Ng 12-11 1 µM_x000d_ | 0.0 |
| Ng 12-11 3 µM_x000d_ | 0.0 |
| Ng 13-15 1 µM_x000d_ | 0.0 |
| Ng 13-15 3 µM_x000d_ | 2.333333333333343 |
| Ng 13-14_x000d_ | 2314.666666666667 |
| Ng 14-10 1 µM_x000d_ | 4050.6666666666665 |
| Ng 14-10 3 µM_x000d_ | 17970.333333333332 |
| Ng 14-11 1 µM_x000d_ | 880.0000000000001 |
| Ng 14-12 1 µM_x000d_ | 16596.0 |
| Ng 14-7 1 µM_x000d_ | 6781.666666666666 |
| Ng 15-14/15 1 µM_x000d_ | 9095.333333333334 |
| Ng 15-12 1µM_x000d_ | 162.66666666666666 |
| Ng 17-7 1 µM_x000d_ | 2123.0 |
| Ng 17-7 3 µM_x000d_ | 11868.666666666668 |
| Ng 17-7 10 µM_x000d_ | 12050.666666666668 |
| Ng 19-9 1 µM_x000d_ | 2766.0 |
| Ng 19-9 3 µM_x000d_ | 16861.333333333332 |
| Ng 19-9 10 µM_x000d_ | 20398.0 |
| Ng 20-7 1 µM_x000d_ | 1819.0 |
| Ng 20-7 3 µM_x000d_ | 10704.0 |
| Ng 20-7 10 µM_x000d_ | 23440.0 |
| Ng 21-8 1 µM_x000d_ | 328.66666666666663 |
| Ng 21-8 3 µM_x000d_ | 3086.666666666667 |
| Ng 21-8 10 µM_x000d_ | 15138.0 |
| Ng 15-13 3µM_x000d_ | 1470.0 |
| Ng 16-12 1 µM_x000d_ | 97.33333333333334 |
| Ng 17-9 1 µM_x000d_ | 2088.3333333333335 |
| Ng 17-9 3 µM_x000d_ | 5436.333333333333 |
| Ng 18-11 1 µM_x000d_ | 1200.3333333333333 |
| Ng 18-11 3 µM_x000d_ | 2167.0 |
| Ng 20-8 1 µM_x000d_ | 3366.0 |
| Ng 20-9 1 µM_x000d_ | 6356.0 |
| Ng 20-9 3 µM_x000d_ | 17485.666666666664 |
| Ng 21-9 1 µM_x000d_ | 4661.0 |
| Ng 13-13 3 µM_x000d_ | 6.333333333333343 |
| Ng 14-8 1 µM_x000d_ | 306.0 |
| Ng 14-9 1 µM_x000d_ | 36.666666666666686 |
| Ng 14-9 3 µM_x000d_ | 136.66666666666666 |
| Ng 14-9 10 µM_x000d_ | 372.0 |
| Ng 14-9 30 µM_x000d_ | 331.0 |
| Ng 15-10 1µM_x000d_ | 17700.666666666664 |
| Ng 16-9 1 µM_x000d_ | 7183.0 |
| Ng 17-8 1 µM_x000d_ | 971.6666666666666 |
| Ng 17-8 3 µM_x000d_ | 4410.666666666666 |
| Ng 18-9 1µM_x000d_ | 893.6666666666666 |
| Ng 19-11 1 µM_x000d_ | 10530.333333333334 |
| Ng 19-11 3 µM_x000d_ | 26221.0 |
| Lg 17-15 HF 10 µM_x000d_ | 6851.333333333333 |
| Lg 15-17 1 µM_x000d_ | 249.33333333333334 |
| Lg 17-15 1 µM_x000d_ | 405.0 |
| Lg 17-15 3 µM_x000d_ | 1596.6666666666665 |
| Lg 17-15 10 µM_x000d_ | 10395.0 |
| Lg 17-17 1 µM_x000d_ | 305.66666666666663 |
| Lg 17-16 1 µM_x000d_ | 1437.0 |
| Lg 16-17 3 µM_x000d_ | 2181.666666666667 |
| Lg 18-8 1 µM_x000d_ | 1205.6666666666665 |
| Lg 19-8 1 µM_x000d_ | 8148.999999999999 |
| Lg 19-8 3 µM_x000d_ | 19724.666666666664 |
| Lg 19-9 1 µM_x000d_ | 2849.3333333333335 |
| Lg 20-10_x000d_ | 6750.0 |
| Lg 22-12 1 µM_x000d_ | 939.3333333333334 |
| Lg 20-9 1 µM_x000d_ | 20332.333333333332 |
| Lg 23-11 1 µM_x000d_ | 12973.333333333334 |
| Lg 23-11 3 µM_x000d_ | 16132.333333333334 |
| Lg 23-12_x000d_ | 7477.0 |
| Lg 26-8_x000d_ | 9968.333333333334 |
| Lg 26-9_x000d_ | 1632.6666666666665 |
| Lg 22-13_x000d_ | 2362.666666666667 |
| Lg 26-10_x000d_ | 239.00000000000003 |
| Lg 18-7-F3 HF 1uM | 7246.0 |
| Lg 19-8-F5 HF 3uM | 15804.333333333334 |
| Lg 23-11-F2 HF 3uM | 4764.0 |
| Blank 1 | 29.333333333333343 |
| Blank 2 | 0.0 |
| Blank 3 | 0.0 |
| Blank 4 | 28.666666666666686 |
| Blank 5 | 0.0 |MFI
### Chart: Ov-3
| Category | Ov k-84 IgG2 |
|---|---|
| Ng 7-13 1µM_x000d_ | 214.33333333333331 |
| Ng 7-13 3µM_x000d_ | 190.33333333333331 |
| Ng 9-9 1 µM_x000d_ | 0.0 |
| Ng 9-9 3µM_x000d_ | 509.33333333333337 |
| Ng 9-9 10µM_x000d_ | 248.0 |
| Ng 10-13 1 µM_x000d_ | 39.66666666666666 |
| Ng 10-13 3 µM_x000d_ | 0.0 |
| Ng 10-13 10 µM_x000d_ | 156.0 |
| Ng 10-13 30 µM_x000d_ | 33.66666666666666 |
| Ng 10-10 1 µM_x000d_ | 149.33333333333331 |
| Ng 10-10 3 µM_x000d_ | 82.66666666666666 |
| Ng 10-11 1 µM_x000d_ | 0.0 |
| Ng 10-11 3 µM_x000d_ | 93.33333333333334 |
| Ng 10-12 1 µM_x000d_ | 55.66666666666666 |
| Ng 11-10 1 µM_x000d_ | 83.0 |
| Ng 11-10 3 µM_x000d_ | 164.66666666666669 |
| Ng 11-11 1 µM_x000d_ | 80.0 |
| Ng 11-11 3µM_x000d_ | 108.66666666666669 |
| Ng 11-11 10µM_x000d_ | 0.0 |
| Ng 12-11 1 µM_x000d_ | 0.0 |
| Ng 12-11 3 µM_x000d_ | 61.66666666666666 |
| Ng 13-15 1 µM_x000d_ | 0.0 |
| Ng 13-15 3 µM_x000d_ | 154.0 |
| Ng 13-14_x000d_ | 1405.6666666666667 |
| Ng 14-10 1 µM_x000d_ | 752.6666666666666 |
| Ng 14-10 3 µM_x000d_ | 6665.0 |
| Ng 14-11 1 µM_x000d_ | 418.0 |
| Ng 14-12 1 µM_x000d_ | 13005.333333333334 |
| Ng 14-7 1 µM_x000d_ | 658.0 |
| Ng 15-14/15 1 µM_x000d_ | 4088.0 |
| Ng 15-12 1µM_x000d_ | 0.0 |
| Ng 17-7 1 µM_x000d_ | 68.33333333333334 |
| Ng 17-7 3 µM_x000d_ | 1382.0 |
| Ng 17-7 10 µM_x000d_ | 2852.3333333333335 |
| Ng 19-9 1 µM_x000d_ | 312.6666666666667 |
| Ng 19-9 3 µM_x000d_ | 965.5 |
| Ng 19-9 10 µM_x000d_ | 1364.6666666666667 |
| Ng 20-7 1 µM_x000d_ | 219.0 |
| Ng 20-7 3 µM_x000d_ | 2034.0 |
| Ng 20-7 10 µM_x000d_ | 2844.0 |
| Ng 21-8 1 µM_x000d_ | 0.0 |
| Ng 21-8 3 µM_x000d_ | 412.0 |
| Ng 21-8 10 µM_x000d_ | 3805.3333333333335 |
| Ng 15-13 3µM_x000d_ | 642.3333333333334 |
| Ng 16-12 1 µM_x000d_ | 0.0 |
| Ng 17-9 1 µM_x000d_ | 506.0 |
| Ng 17-9 3 µM_x000d_ | 802.6666666666666 |
| Ng 18-11 1 µM_x000d_ | 388.0 |
| Ng 18-11 3 µM_x000d_ | 468.33333333333337 |
| Ng 20-8 1 µM_x000d_ | 475.33333333333337 |
| Ng 20-9 1 µM_x000d_ | 893.6666666666667 |
| Ng 20-9 3 µM_x000d_ | 1251.3333333333333 |
| Ng 21-9 1 µM_x000d_ | 1863.6666666666667 |
| Ng 13-13 3 µM_x000d_ | 25.0 |
| Ng 14-8 1 µM_x000d_ | 175.0 |
| Ng 14-9 1 µM_x000d_ | 45.66666666666666 |
| Ng 14-9 3 µM_x000d_ | 162.0 |
| Ng 14-9 10 µM_x000d_ | 98.0 |
| Ng 14-9 30 µM_x000d_ | 166.33333333333331 |
| Ng 15-10 1µM_x000d_ | 2873.3333333333335 |
| Ng 16-9 1 µM_x000d_ | 917.6666666666667 |
| Ng 17-8 1 µM_x000d_ | 226.0 |
| Ng 17-8 3 µM_x000d_ | 486.66666666666663 |
| Ng 18-9 1µM_x000d_ | 96.33333333333334 |
| Ng 19-11 1 µM_x000d_ | 1611.3333333333333 |
| Ng 19-11 3 µM_x000d_ | 4526.666666666667 |
| Lg 17-15 HF 10 µM_x000d_ | 981.3333333333333 |
| Lg 15-17 1 µM_x000d_ | 60.66666666666666 |
| Lg 17-15 1 µM_x000d_ | 916.0 |
| Lg 17-15 3 µM_x000d_ | 4715.666666666667 |
| Lg 17-15 10 µM_x000d_ | 6061.666666666667 |
| Lg 17-17 1 µM_x000d_ | 331.3333333333333 |
| Lg 17-16 1 µM_x000d_ | 221.33333333333331 |
| Lg 16-17 3 µM_x000d_ | 230.66666666666669 |
| Lg 18-8 1 µM_x000d_ | 949.0 |
| Lg 19-8 1 µM_x000d_ | 1197.6666666666667 |
| Lg 19-8 3 µM_x000d_ | 7352.333333333333 |
| Lg 19-9 1 µM_x000d_ | 2172.0 |
| Lg 20-10_x000d_ | 582.6666666666666 |
| Lg 22-12 1 µM_x000d_ | 116.0 |
| Lg 20-9 1 µM_x000d_ | 5710.333333333333 |
| Lg 23-11 1 µM_x000d_ | 2651.0 |
| Lg 23-11 3 µM_x000d_ | 11506.0 |
| Lg 23-12_x000d_ | 1638.3333333333333 |
| Lg 26-8_x000d_ | 3762.0 |
| Lg 26-9_x000d_ | 743.6666666666666 |
| Lg 22-13_x000d_ | 645.6666666666666 |
| Lg 26-10_x000d_ | 36.0 |
| Lg 18-7-F3 HF 1uM | 450.33333333333337 |
| Lg 19-8-F5 HF 3uM | 5220.333333333333 |
| Lg 23-11-F2 HF 3uM | 1146.0 |
| Blank 1 | 30.666666666666657 |
| Blank 2 | 0.0 |
| Blank 3 | 0.0 |
| Blank 4 | 0.0 |
| Blank 5 | 0.0 |MFI
### Chart: Ov-4
| Category | Ov k-96 IgG2 |
|---|---|
| Ng 7-13 1µM_x000d_ | 93.33333333333334 |
| Ng 7-13 3µM_x000d_ | 70.33333333333334 |
| Ng 9-9 1 µM_x000d_ | 0.0 |
| Ng 9-9 3µM_x000d_ | 123.33333333333331 |
| Ng 9-9 10µM_x000d_ | 171.0 |
| Ng 10-13 1 µM_x000d_ | 10.333333333333343 |
| Ng 10-13 3 µM_x000d_ | 0.0 |
| Ng 10-13 10 µM_x000d_ | 61.33333333333334 |
| Ng 10-13 30 µM_x000d_ | 33.66666666666666 |
| Ng 10-10 1 µM_x000d_ | 9.5 |
| Ng 10-10 3 µM_x000d_ | 74.33333333333334 |
| Ng 10-11 1 µM_x000d_ | 0.0 |
| Ng 10-11 3 µM_x000d_ | 43.66666666666666 |
| Ng 10-12 1 µM_x000d_ | 77.66666666666666 |
| Ng 11-10 1 µM_x000d_ | 56.0 |
| Ng 11-10 3 µM_x000d_ | 12.333333333333343 |
| Ng 11-11 1 µM_x000d_ | 17.333333333333343 |
| Ng 11-11 3µM_x000d_ | 59.33333333333334 |
| Ng 11-11 10µM_x000d_ | 0.0 |
| Ng 12-11 1 µM_x000d_ | 0.0 |
| Ng 12-11 3 µM_x000d_ | 68.33333333333334 |
| Ng 13-15 1 µM_x000d_ | 0.0 |
| Ng 13-15 3 µM_x000d_ | 118.0 |
| Ng 13-14_x000d_ | 743.3333333333334 |
| Ng 14-10 1 µM_x000d_ | 824.6666666666666 |
| Ng 14-10 3 µM_x000d_ | 7524.666666666667 |
| Ng 14-11 1 µM_x000d_ | 754.6666666666666 |
| Ng 14-12 1 µM_x000d_ | 11249.666666666666 |
| Ng 14-7 1 µM_x000d_ | 138.66666666666669 |
| Ng 15-14/15 1 µM_x000d_ | 4861.666666666667 |
| Ng 15-12 1µM_x000d_ | 0.0 |
| Ng 17-7 1 µM_x000d_ | 5.0 |
| Ng 17-7 3 µM_x000d_ | 163.0 |
| Ng 17-7 10 µM_x000d_ | 359.0 |
| Ng 19-9 1 µM_x000d_ | 70.0 |
| Ng 19-9 3 µM_x000d_ | 229.0 |
| Ng 19-9 10 µM_x000d_ | 1000.0 |
| Ng 20-7 1 µM_x000d_ | 16.333333333333343 |
| Ng 20-7 3 µM_x000d_ | 258.3333333333333 |
| Ng 20-7 10 µM_x000d_ | 384.66666666666663 |
| Ng 21-8 1 µM_x000d_ | 0.0 |
| Ng 21-8 3 µM_x000d_ | 245.66666666666669 |
| Ng 21-8 10 µM_x000d_ | 2042.0 |
| Ng 15-13 3µM_x000d_ | 516.3333333333334 |
| Ng 16-12 1 µM_x000d_ | 4.333333333333343 |
| Ng 17-9 1 µM_x000d_ | 256.6666666666667 |
| Ng 17-9 3 µM_x000d_ | 780.3333333333334 |
| Ng 18-11 1 µM_x000d_ | 477.0 |
| Ng 18-11 3 µM_x000d_ | 613.0 |
| Ng 20-8 1 µM_x000d_ | 830.6666666666666 |
| Ng 20-9 1 µM_x000d_ | 1451.3333333333333 |
| Ng 20-9 3 µM_x000d_ | 2411.6666666666665 |
| Ng 21-9 1 µM_x000d_ | 3945.3333333333335 |
| Ng 13-13 3 µM_x000d_ | 62.0 |
| Ng 14-8 1 µM_x000d_ | 0.0 |
| Ng 14-9 1 µM_x000d_ | 0.0 |
| Ng 14-9 3 µM_x000d_ | 63.33333333333334 |
| Ng 14-9 10 µM_x000d_ | 103.0 |
| Ng 14-9 30 µM_x000d_ | 182.33333333333331 |
| Ng 15-10 1µM_x000d_ | 283.0 |
| Ng 16-9 1 µM_x000d_ | 176.66666666666669 |
| Ng 17-8 1 µM_x000d_ | 33.66666666666666 |
| Ng 17-8 3 µM_x000d_ | 89.0 |
| Ng 18-9 1µM_x000d_ | 74.33333333333334 |
| Ng 19-11 1 µM_x000d_ | 6014.333333333333 |
| Ng 19-11 3 µM_x000d_ | 12034.0 |
| Lg 17-15 HF 10 µM_x000d_ | 2887.3333333333335 |
| Lg 15-17 1 µM_x000d_ | 117.33333333333331 |
| Lg 17-15 1 µM_x000d_ | 984.0 |
| Lg 17-15 3 µM_x000d_ | 1874.0 |
| Lg 17-15 10 µM_x000d_ | 3589.0 |
| Lg 17-17 1 µM_x000d_ | 477.66666666666663 |
| Lg 17-16 1 µM_x000d_ | 507.33333333333337 |
| Lg 16-17 3 µM_x000d_ | 6818.666666666667 |
| Lg 18-8 1 µM_x000d_ | 1404.3333333333333 |
| Lg 19-8 1 µM_x000d_ | 86.0 |
| Lg 19-8 3 µM_x000d_ | 4319.333333333333 |
| Lg 19-9 1 µM_x000d_ | 1289.0 |
| Lg 20-10_x000d_ | 302.6666666666667 |
| Lg 22-12 1 µM_x000d_ | 0.0 |
| Lg 20-9 1 µM_x000d_ | 519.3333333333334 |
| Lg 23-11 1 µM_x000d_ | 4310.0 |
| Lg 23-11 3 µM_x000d_ | 21294.666666666668 |
| Lg 23-12_x000d_ | 1237.3333333333333 |
| Lg 26-8_x000d_ | 3391.3333333333335 |
| Lg 26-9_x000d_ | 2064.3333333333335 |
| Lg 22-13_x000d_ | 1194.3333333333333 |
| Lg 26-10_x000d_ | 702.3333333333334 |
| Lg 18-7-F3 HF 1uM | 0.0 |
| Lg 19-8-F5 HF 3uM | 353.6666666666667 |
| Lg 23-11-F2 HF 3uM | 61.66666666666666 |
| Blank 1 | 0.0 |
| Blank 2 | 0.0 |
| Blank 3 | 0.0 |
| Blank 4 | 0.0 |
| Blank 5 | 0.0 |MFI
### Chart: Ov-5
| Category | Ov k-99 IgG2 |
|---|---|
| Ng 7-13 1µM_x000d_ | 73.0 |
| Ng 7-13 3µM_x000d_ | 84.33333333333334 |
| Ng 9-9 1 µM_x000d_ | 0.0 |
| Ng 9-9 3µM_x000d_ | 461.0 |
| Ng 9-9 10µM_x000d_ | 80.33333333333334 |
| Ng 10-13 1 µM_x000d_ | 0.0 |
| Ng 10-13 3 µM_x000d_ | 0.0 |
| Ng 10-13 10 µM_x000d_ | 2.0 |
| Ng 10-13 30 µM_x000d_ | 0.0 |
| Ng 10-10 1 µM_x000d_ | 3.333333333333343 |
| Ng 10-10 3 µM_x000d_ | 25.666666666666657 |
| Ng 10-11 1 µM_x000d_ | 0.0 |
| Ng 10-11 3 µM_x000d_ | 66.66666666666666 |
| Ng 10-12 1 µM_x000d_ | 17.0 |
| Ng 11-10 1 µM_x000d_ | 0.0 |
| Ng 11-10 3 µM_x000d_ | 5.0 |
| Ng 11-11 1 µM_x000d_ | 1.6666666666666572 |
| Ng 11-11 3µM_x000d_ | 82.33333333333334 |
| Ng 11-11 10µM_x000d_ | 0.0 |
| Ng 12-11 1 µM_x000d_ | 0.0 |
| Ng 12-11 3 µM_x000d_ | 24.666666666666657 |
| Ng 13-15 1 µM_x000d_ | 0.0 |
| Ng 13-15 3 µM_x000d_ | 29.333333333333343 |
| Ng 13-14_x000d_ | 1303.0 |
| Ng 14-10 1 µM_x000d_ | 0.0 |
| Ng 14-10 3 µM_x000d_ | 224.0 |
| Ng 14-11 1 µM_x000d_ | 0.0 |
| Ng 14-12 1 µM_x000d_ | 251.0 |
| Ng 14-7 1 µM_x000d_ | 227.0 |
| Ng 15-14/15 1 µM_x000d_ | 58.33333333333334 |
| Ng 15-12 1µM_x000d_ | 0.0 |
| Ng 17-7 1 µM_x000d_ | 0.0 |
| Ng 17-7 3 µM_x000d_ | 224.33333333333331 |
| Ng 17-7 10 µM_x000d_ | 1041.0 |
| Ng 19-9 1 µM_x000d_ | 13.0 |
| Ng 19-9 3 µM_x000d_ | 392.33333333333337 |
| Ng 19-9 10 µM_x000d_ | 721.6666666666666 |
| Ng 20-7 1 µM_x000d_ | 0.0 |
| Ng 20-7 3 µM_x000d_ | 427.0 |
| Ng 20-7 10 µM_x000d_ | 1820.6666666666667 |
| Ng 21-8 1 µM_x000d_ | 0.0 |
| Ng 21-8 3 µM_x000d_ | 67.33333333333334 |
| Ng 21-8 10 µM_x000d_ | 987.0 |
| Ng 15-13 3µM_x000d_ | 91.0 |
| Ng 16-12 1 µM_x000d_ | 0.0 |
| Ng 17-9 1 µM_x000d_ | 67.0 |
| Ng 17-9 3 µM_x000d_ | 233.66666666666669 |
| Ng 18-11 1 µM_x000d_ | 5.333333333333343 |
| Ng 18-11 3 µM_x000d_ | 22.666666666666657 |
| Ng 20-8 1 µM_x000d_ | 250.33333333333331 |
| Ng 20-9 1 µM_x000d_ | 65.0 |
| Ng 20-9 3 µM_x000d_ | 321.0 |
| Ng 21-9 1 µM_x000d_ | 220.0 |
| Ng 13-13 3 µM_x000d_ | 0.0 |
| Ng 14-8 1 µM_x000d_ | 0.0 |
| Ng 14-9 1 µM_x000d_ | 0.0 |
| Ng 14-9 3 µM_x000d_ | 0.0 |
| Ng 14-9 10 µM_x000d_ | 17.333333333333343 |
| Ng 14-9 30 µM_x000d_ | 44.33333333333334 |
| Ng 15-10 1µM_x000d_ | 1284.3333333333333 |
| Ng 16-9 1 µM_x000d_ | 293.0 |
| Ng 17-8 1 µM_x000d_ | 0.0 |
| Ng 17-8 3 µM_x000d_ | 144.0 |
| Ng 18-9 1µM_x000d_ | 33.33333333333334 |
| Ng 19-11 1 µM_x000d_ | 181.33333333333331 |
| Ng 19-11 3 µM_x000d_ | 338.3333333333333 |
| Lg 17-15 HF 10 µM_x000d_ | 5502.666666666667 |
| Lg 15-17 1 µM_x000d_ | 5.0 |
| Lg 17-15 1 µM_x000d_ | 1194.0 |
| Lg 17-15 3 µM_x000d_ | 6872.333333333333 |
| Lg 17-15 10 µM_x000d_ | 23197.0 |
| Lg 17-17 1 µM_x000d_ | 855.3333333333334 |
| Lg 17-16 1 µM_x000d_ | 1564.6666666666667 |
| Lg 16-17 3 µM_x000d_ | 2706.3333333333335 |
| Lg 18-8 1 µM_x000d_ | 443.66666666666663 |
| Lg 19-8 1 µM_x000d_ | 551.0 |
| Lg 19-8 3 µM_x000d_ | 3002.3333333333335 |
| Lg 19-9 1 µM_x000d_ | 1082.0 |
| Lg 20-10_x000d_ | 206.0 |
| Lg 22-12 1 µM_x000d_ | 65.66666666666666 |
| Lg 20-9 1 µM_x000d_ | 1706.6666666666667 |
| Lg 23-11 1 µM_x000d_ | 2009.5 |
| Lg 23-11 3 µM_x000d_ | 3183.0 |
| Lg 23-12_x000d_ | 753.3333333333334 |
| Lg 26-8_x000d_ | 1185.0 |
| Lg 26-9_x000d_ | 93.66666666666666 |
| Lg 22-13_x000d_ | 77.66666666666666 |
| Lg 26-10_x000d_ | 0.0 |
| Lg 18-7-F3 HF 1uM | 160.66666666666669 |
| Lg 19-8-F5 HF 3uM | 2047.0 |
| Lg 23-11-F2 HF 3uM | 241.66666666666669 |
| Blank 1 | 0.0 |
| Blank 2 | 0.0 |
| Blank 3 | 0.0 |
| Blank 4 | 0.0 |
| Blank 5 | 0.0 |MFI
### Chart: Ov-6
| Category | Ov k-100 IgG2 |
|---|---|
| Ng1_1uM
 | 56.33333333333334 |
| Ng1_3uM
 | 139.66666666666669 |
| Ng2_1uM | 0.0 |
| Ng2_3uM | 172.33333333333331 |
| Ng2_10uM | 75.66666666666666 |
| Ng3_1uM | 0.0 |
| Ng3_3uM | 0.0 |
| Ng3_10uM | 18.0 |
| Ng3_30uM | 50.0 |
| Ng4_1uM | 8.666666666666657 |
| Ng4_3uM | 38.33333333333334 |
| Ng5_1uM | 0.3333333333333428 |
| Ng5_3uM | 82.33333333333334 |
| Ng6_1uM | 28.666666666666657 |
| Ng7_1uM | 0.0 |
| Ng7_3uM | 21.0 |
| Ng8_1uM | 0.0 |
| Ng8_3uM | 31.0 |
| Ng8_10uM | 3.666666666666657 |
| Ng9_1uM | 0.0 |
| Ng9_3uM | 62.66666666666666 |
| Ng10_3uM | 0.0 |
| Ng10_1uM | 34.5 |
| Ng11 | 712.6666666666666 |
| Ng12_1uM | 0.0 |
| Ng12_3uM | 156.66666666666669 |
| Ng13_1uM | 0.0 |
| Ng14_1uM | 298.0 |
| Ng15_1uM | 754.3333333333334 |
| Ng16_1uM | 71.0 |
| Ng17_1uM | 0.0 |
| Ng18_1uM | 59.66666666666666 |
| Ng18_3uM | 782.6666666666666 |
| Ng18_10uM | 1828.0 |
| Ng19_1uM | 178.33333333333331 |
| Ng19_3uM | 1128.0 |
| Ng19_10uM | 1226.0 |
| Ng20_1uM | 80.66666666666666 |
| Ng20_3uM | 1102.6666666666667 |
| Ng20_10uM | 3021.0 |
| Ng21_1uM | 0.0 |
| Ng21 _3uM | 183.0 |
| Ng21_10uM | 2368.0 |
| Ng22_1uM | 165.33333333333331 |
| Ng23_1uM | 0.0 |
| Ng24_1uM | 50.66666666666666 |
| Ng24_3uM | 498.33333333333337 |
| Ng25_1uM | 117.66666666666669 |
| Ng25_3uM | 165.66666666666669 |
| Ng26_1uM | 194.0 |
| Ng27_1uM | 301.0 |
| Ng27_3uM | 640.0 |
| Ng28_1uM | 377.66666666666663 |
| Ng29_1uM | 9.333333333333343 |
| Ng30_1uM | 63.33333333333334 |
| Ng31_1uM | 0.0 |
| Ng31_3uM | 1.0 |
| Ng31_10uM | 70.33333333333334 |
| Ng31_30uM | 85.0 |
| Ng32_1uM | 1488.0 |
| Ng33_1uM | 846.0 |
| Ng34_1uM | 79.0 |
| Ng34_3uM | 429.33333333333337 |
| Ng35_1uM | 97.0 |
| Ng36_1uM | 363.0 |
| Ng36_3uM | 1094.3333333333333 |
| Lg2_HF_10uM | 5817.333333333333 |
| Lg1_1uM | 12.0 |
| Lg2_1uM | 524.6666666666666 |
| Lg2_3uM | 4286.0 |
| Lg2_10uM | 14812.333333333334 |
| Lg3_1uM | 701.6666666666666 |
| Lg4_1uM | 1014.3333333333333 |
| Lg5_3uM | 2115.0 |
| Lg6_1uM | 472.33333333333337 |
| Lg7_1uM | 519.3333333333334 |
| Lg7_3uM | 2181.3333333333335 |
| Lg8_1uM | 1357.3333333333333 |
| Lg9 | 462.66666666666663 |
| Lg10_1uM | 147.33333333333331 |
| Lg11_1uM | 2758.3333333333335 |
| Lg12_1uM | 1436.0 |
| Lg12_3uM | 3899.6666666666665 |
| Lg13 | 2304.6666666666665 |
| Lg14 | 2037.0 |
| Lg15 | 251.33333333333331 |
| Lg16 | 563.3333333333334 |
| Lg17 | 0.0 |
| Lg18_HF_1uM | 658.6666666666666 |
| Lg7_HF_3uM | 2908.3333333333335 |
| Lg12_HF_3uM | 638.6666666666666 |
| Blank 1 | 0.0 |
| Blank 2 | 0.0 |
| Blank 3 | 0.0 |
| Blank 4 | 0.0 |
| Blank 5 | 0.0 |MFI

## Slide 26
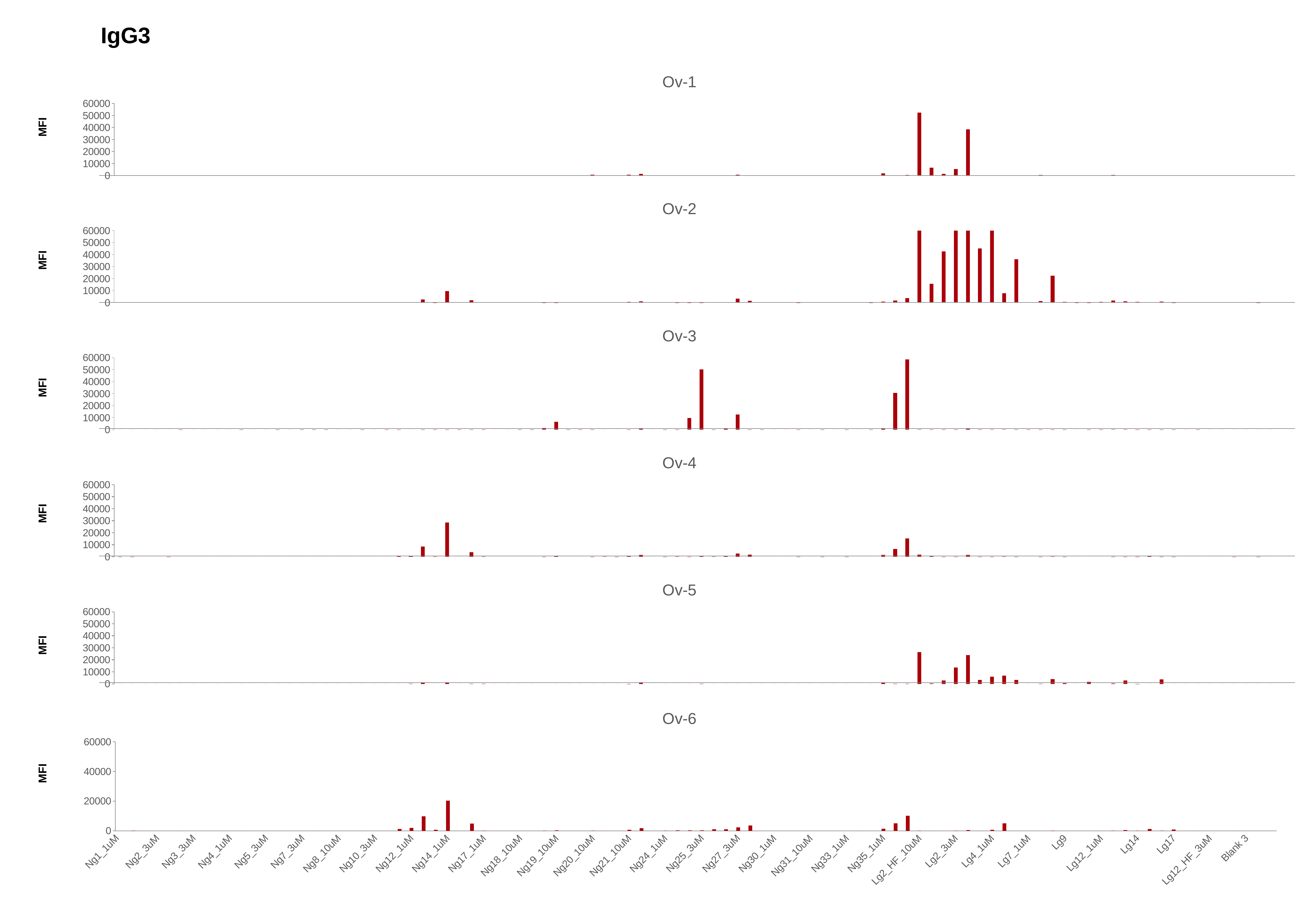

IgG3
### Chart: Ov-1
| Category | Ov 38-207 IgG3 |
|---|---|
| Ng 7-13 1µM_x000d_ | 0.0 |
| Ng 7-13 3µM_x000d_ | 16.333333333333343 |
| Ng 9-9 1 µM_x000d_ | 0.0 |
| Ng 9-9 3µM_x000d_ | 0.0 |
| Ng 9-9 10µM_x000d_ | 0.0 |
| Ng 10-13 1 µM_x000d_ | 0.0 |
| Ng 10-13 3 µM_x000d_ | 0.0 |
| Ng 10-13 10 µM_x000d_ | 0.0 |
| Ng 10-13 30 µM_x000d_ | 0.0 |
| Ng 10-10 1 µM_x000d_ | 0.0 |
| Ng 10-10 3 µM_x000d_ | 0.0 |
| Ng 10-11 1 µM_x000d_ | 0.0 |
| Ng 10-11 3 µM_x000d_ | 0.0 |
| Ng 10-12 1 µM_x000d_ | 0.0 |
| Ng 11-10 1 µM_x000d_ | 0.0 |
| Ng 11-10 3 µM_x000d_ | 0.0 |
| Ng 11-11 1 µM_x000d_ | 0.0 |
| Ng 11-11 3µM_x000d_ | 0.0 |
| Ng 11-11 10µM_x000d_ | 0.0 |
| Ng 12-11 1 µM_x000d_ | 0.0 |
| Ng 12-11 3 µM_x000d_ | 0.0 |
| Ng 13-15 1 µM_x000d_ | 0.0 |
| Ng 13-15 3 µM_x000d_ | 0.0 |
| Ng 13-14_x000d_ | 0.0 |
| Ng 14-10 1 µM_x000d_ | 0.0 |
| Ng 14-10 3 µM_x000d_ | 31.0 |
| Ng 14-11 1 µM_x000d_ | 0.0 |
| Ng 14-12 1 µM_x000d_ | 52.0 |
| Ng 14-7 1 µM_x000d_ | 19.0 |
| Ng 15-14/15 1 µM_x000d_ | 11.0 |
| Ng 15-12 1µM_x000d_ | 112.0 |
| Ng 17-7 1 µM_x000d_ | 0.0 |
| Ng 17-7 3 µM_x000d_ | 0.0 |
| Ng 17-7 10 µM_x000d_ | 43.66666666666666 |
| Ng 19-9 1 µM_x000d_ | 34.66666666666666 |
| Ng 19-9 3 µM_x000d_ | 63.33333333333334 |
| Ng 19-9 10 µM_x000d_ | 236.0 |
| Ng 20-7 1 µM_x000d_ | 0.0 |
| Ng 20-7 3 µM_x000d_ | 186.66666666666669 |
| Ng 20-7 10 µM_x000d_ | 646.0 |
| Ng 21-8 1 µM_x000d_ | 0.0 |
| Ng 21-8 3 µM_x000d_ | 104.0 |
| Ng 21-8 10 µM_x000d_ | 754.3333333333334 |
| Ng 15-13 3µM_x000d_ | 1512.6666666666667 |
| Ng 16-12 1 µM_x000d_ | 0.0 |
| Ng 17-9 1 µM_x000d_ | 23.333333333333343 |
| Ng 17-9 3 µM_x000d_ | 323.0 |
| Ng 18-11 1 µM_x000d_ | 50.0 |
| Ng 18-11 3 µM_x000d_ | 37.66666666666666 |
| Ng 20-8 1 µM_x000d_ | 188.66666666666669 |
| Ng 20-9 1 µM_x000d_ | 27.333333333333343 |
| Ng 20-9 3 µM_x000d_ | 787.3333333333334 |
| Ng 21-9 1 µM_x000d_ | 67.0 |
| Ng 13-13 3 µM_x000d_ | 0.0 |
| Ng 14-8 1 µM_x000d_ | 0.0 |
| Ng 14-9 1 µM_x000d_ | 0.0 |
| Ng 14-9 3 µM_x000d_ | 0.0 |
| Ng 14-9 10 µM_x000d_ | 0.0 |
| Ng 14-9 30 µM_x000d_ | 0.0 |
| Ng 15-10 1µM_x000d_ | 121.33333333333331 |
| Ng 16-9 1 µM_x000d_ | 120.66666666666669 |
| Ng 17-8 1 µM_x000d_ | 0.0 |
| Ng 17-8 3 µM_x000d_ | 0.6666666666666572 |
| Ng 18-9 1µM_x000d_ | 1828.6666666666667 |
| Ng 19-11 1 µM_x000d_ | 248.33333333333331 |
| Ng 19-11 3 µM_x000d_ | 604.6666666666666 |
| Lg 17-15 HF 10 µM_x000d_ | 52349.666666666664 |
| Lg 15-17 1 µM_x000d_ | 6507.666666666667 |
| Lg 17-15 1 µM_x000d_ | 1334.3333333333333 |
| Lg 17-15 3 µM_x000d_ | 5486.0 |
| Lg 17-15 10 µM_x000d_ | 38584.333333333336 |
| Lg 17-17 1 µM_x000d_ | 355.0 |
| Lg 17-16 1 µM_x000d_ | 141.0 |
| Lg 16-17 3 µM_x000d_ | 38.33333333333334 |
| Lg 18-8 1 µM_x000d_ | 0.0 |
| Lg 19-8 1 µM_x000d_ | 80.0 |
| Lg 19-8 3 µM_x000d_ | 531.6666666666666 |
| Lg 19-9 1 µM_x000d_ | 125.66666666666669 |
| Lg 20-10_x000d_ | 179.33333333333331 |
| Lg 22-12 1 µM_x000d_ | 0.0 |
| Lg 20-9 1 µM_x000d_ | 384.33333333333337 |
| Lg 23-11 1 µM_x000d_ | 217.66666666666669 |
| Lg 23-11 3 µM_x000d_ | 553.6666666666666 |
| Lg 23-12_x000d_ | 87.0 |
| Lg 26-8_x000d_ | 319.3333333333333 |
| Lg 26-9_x000d_ | 86.0 |
| Lg 22-13_x000d_ | 90.33333333333334 |
| Lg 26-10_x000d_ | 2.666666666666657 |
| Lg 18-7-F3 HF 1uM | 52.0 |
| Lg 19-8-F5 HF 3uM | 197.66666666666669 |
| Lg 23-11-F2 HF 3uM | 0.6666666666666572 |
| Blank 1 | 0.0 |
| Blank 2 | 0.0 |
| Blank 3 | 0.0 |
| Blank 4 | 0.0 |
| Blank 5 | 0.0 |MFI
### Chart: Ov-2
| Category | Ov k44 IgG3 |
|---|---|
| Ng 7-13 1µM_x000d_ | 0.0 |
| Ng 7-13 3µM_x000d_ | 0.0 |
| Ng 9-9 1 µM_x000d_ | 0.0 |
| Ng 9-9 3µM_x000d_ | 0.0 |
| Ng 9-9 10µM_x000d_ | 0.0 |
| Ng 10-13 1 µM_x000d_ | 0.0 |
| Ng 10-13 3 µM_x000d_ | 0.0 |
| Ng 10-13 10 µM_x000d_ | 0.0 |
| Ng 10-13 30 µM_x000d_ | 0.0 |
| Ng 10-10 1 µM_x000d_ | 0.0 |
| Ng 10-10 3 µM_x000d_ | 0.0 |
| Ng 10-11 1 µM_x000d_ | 0.0 |
| Ng 10-11 3 µM_x000d_ | 0.0 |
| Ng 10-12 1 µM_x000d_ | 0.0 |
| Ng 11-10 1 µM_x000d_ | 0.0 |
| Ng 11-10 3 µM_x000d_ | 0.0 |
| Ng 11-11 1 µM_x000d_ | 0.0 |
| Ng 11-11 3µM_x000d_ | 0.0 |
| Ng 11-11 10µM_x000d_ | 0.0 |
| Ng 12-11 1 µM_x000d_ | 0.0 |
| Ng 12-11 3 µM_x000d_ | 0.0 |
| Ng 13-15 1 µM_x000d_ | 0.0 |
| Ng 13-15 3 µM_x000d_ | 0.0 |
| Ng 13-14_x000d_ | 321.6666666666667 |
| Ng 14-10 1 µM_x000d_ | 378.66666666666663 |
| Ng 14-10 3 µM_x000d_ | 2746.0 |
| Ng 14-11 1 µM_x000d_ | 26.0 |
| Ng 14-12 1 µM_x000d_ | 9645.0 |
| Ng 14-7 1 µM_x000d_ | 0.0 |
| Ng 15-14/15 1 µM_x000d_ | 1906.0 |
| Ng 15-12 1µM_x000d_ | 102.0 |
| Ng 17-7 1 µM_x000d_ | 0.0 |
| Ng 17-7 3 µM_x000d_ | 0.0 |
| Ng 17-7 10 µM_x000d_ | 0.0 |
| Ng 19-9 1 µM_x000d_ | 0.0 |
| Ng 19-9 3 µM_x000d_ | 38.0 |
| Ng 19-9 10 µM_x000d_ | 68.0 |
| Ng 20-7 1 µM_x000d_ | 0.0 |
| Ng 20-7 3 µM_x000d_ | 0.0 |
| Ng 20-7 10 µM_x000d_ | 324.0 |
| Ng 21-8 1 µM_x000d_ | 0.0 |
| Ng 21-8 3 µM_x000d_ | 96.66666666666666 |
| Ng 21-8 10 µM_x000d_ | 548.3333333333334 |
| Ng 15-13 3µM_x000d_ | 1099.6666666666667 |
| Ng 16-12 1 µM_x000d_ | 0.0 |
| Ng 17-9 1 µM_x000d_ | 0.0 |
| Ng 17-9 3 µM_x000d_ | 47.33333333333334 |
| Ng 18-11 1 µM_x000d_ | 25.666666666666657 |
| Ng 18-11 3 µM_x000d_ | 87.66666666666666 |
| Ng 20-8 1 µM_x000d_ | 191.66666666666669 |
| Ng 20-9 1 µM_x000d_ | 472.66666666666663 |
| Ng 20-9 3 µM_x000d_ | 3389.0 |
| Ng 21-9 1 µM_x000d_ | 1457.6666666666667 |
| Ng 13-13 3 µM_x000d_ | 0.0 |
| Ng 14-8 1 µM_x000d_ | 0.0 |
| Ng 14-9 1 µM_x000d_ | 0.0 |
| Ng 14-9 3 µM_x000d_ | 1.0 |
| Ng 14-9 10 µM_x000d_ | 0.0 |
| Ng 14-9 30 µM_x000d_ | 0.0 |
| Ng 15-10 1µM_x000d_ | 0.0 |
| Ng 16-9 1 µM_x000d_ | 0.0 |
| Ng 17-8 1 µM_x000d_ | 0.0 |
| Ng 17-8 3 µM_x000d_ | 18.666666666666657 |
| Ng 18-9 1µM_x000d_ | 809.6666666666666 |
| Ng 19-11 1 µM_x000d_ | 1750.0 |
| Ng 19-11 3 µM_x000d_ | 3760.3333333333335 |
| Lg 17-15 HF 10 µM_x000d_ | 64955.0 |
| Lg 15-17 1 µM_x000d_ | 15788.333333333334 |
| Lg 17-15 1 µM_x000d_ | 42694.0 |
| Lg 17-15 3 µM_x000d_ | 64859.333333333336 |
| Lg 17-15 10 µM_x000d_ | 61897.333333333336 |
| Lg 17-17 1 µM_x000d_ | 45095.333333333336 |
| Lg 17-16 1 µM_x000d_ | 61253.0 |
| Lg 16-17 3 µM_x000d_ | 7807.666666666667 |
| Lg 18-8 1 µM_x000d_ | 36207.333333333336 |
| Lg 19-8 1 µM_x000d_ | 303.0 |
| Lg 19-8 3 µM_x000d_ | 1262.3333333333333 |
| Lg 19-9 1 µM_x000d_ | 22547.666666666668 |
| Lg 20-10_x000d_ | 701.0 |
| Lg 22-12 1 µM_x000d_ | 49.33333333333334 |
| Lg 20-9 1 µM_x000d_ | 36.33333333333334 |
| Lg 23-11 1 µM_x000d_ | 552.3333333333334 |
| Lg 23-11 3 µM_x000d_ | 1782.3333333333333 |
| Lg 23-12_x000d_ | 1100.0 |
| Lg 26-8_x000d_ | 626.3333333333334 |
| Lg 26-9_x000d_ | 277.3333333333333 |
| Lg 22-13_x000d_ | 770.3333333333334 |
| Lg 26-10_x000d_ | 61.0 |
| Lg 18-7-F3 HF 1uM | 0.0 |
| Lg 19-8-F5 HF 3uM | 451.0 |
| Lg 23-11-F2 HF 3uM | 0.0 |
| Blank 1 | 0.0 |
| Blank 2 | 0.0 |
| Blank 3 | 0.0 |
| Blank 4 | 21.333333333333343 |
| Blank 5 | 0.0 |MFI
### Chart: Ov-3
| Category | Ov k84 IgG3 |
|---|---|
| Ng 7-13 1µM_x000d_ | 0.0 |
| Ng 7-13 3µM_x000d_ | 0.0 |
| Ng 9-9 1 µM_x000d_ | 0.0 |
| Ng 9-9 3µM_x000d_ | 0.0 |
| Ng 9-9 10µM_x000d_ | 0.0 |
| Ng 10-13 1 µM_x000d_ | 65.0 |
| Ng 10-13 3 µM_x000d_ | 0.0 |
| Ng 10-13 10 µM_x000d_ | 0.0 |
| Ng 10-13 30 µM_x000d_ | 0.0 |
| Ng 10-10 1 µM_x000d_ | 0.0 |
| Ng 10-10 3 µM_x000d_ | 61.33333333333334 |
| Ng 10-11 1 µM_x000d_ | 0.0 |
| Ng 10-11 3 µM_x000d_ | 0.0 |
| Ng 10-12 1 µM_x000d_ | 7.0 |
| Ng 11-10 1 µM_x000d_ | 0.0 |
| Ng 11-10 3 µM_x000d_ | 24.666666666666657 |
| Ng 11-11 1 µM_x000d_ | 6.666666666666657 |
| Ng 11-11 3µM_x000d_ | 116.66666666666669 |
| Ng 11-11 10µM_x000d_ | 0.0 |
| Ng 12-11 1 µM_x000d_ | 0.0 |
| Ng 12-11 3 µM_x000d_ | 40.0 |
| Ng 13-15 1 µM_x000d_ | 0.0 |
| Ng 13-15 3 µM_x000d_ | 24.0 |
| Ng 13-14_x000d_ | 18.666666666666657 |
| Ng 14-10 1 µM_x000d_ | 0.0 |
| Ng 14-10 3 µM_x000d_ | 84.0 |
| Ng 14-11 1 µM_x000d_ | 23.666666666666657 |
| Ng 14-12 1 µM_x000d_ | 56.0 |
| Ng 14-7 1 µM_x000d_ | 80.0 |
| Ng 15-14/15 1 µM_x000d_ | 72.0 |
| Ng 15-12 1µM_x000d_ | 151.33333333333331 |
| Ng 17-7 1 µM_x000d_ | 0.0 |
| Ng 17-7 3 µM_x000d_ | 0.0 |
| Ng 17-7 10 µM_x000d_ | 32.0 |
| Ng 19-9 1 µM_x000d_ | 36.66666666666666 |
| Ng 19-9 3 µM_x000d_ | 1481.0 |
| Ng 19-9 10 µM_x000d_ | 6595.333333333333 |
| Ng 20-7 1 µM_x000d_ | 2.666666666666657 |
| Ng 20-7 3 µM_x000d_ | 27.333333333333343 |
| Ng 20-7 10 µM_x000d_ | 3.0 |
| Ng 21-8 1 µM_x000d_ | 0.0 |
| Ng 21-8 3 µM_x000d_ | 0.0 |
| Ng 21-8 10 µM_x000d_ | 80.0 |
| Ng 15-13 3µM_x000d_ | 1309.0 |
| Ng 16-12 1 µM_x000d_ | 0.0 |
| Ng 17-9 1 µM_x000d_ | 4.0 |
| Ng 17-9 3 µM_x000d_ | 137.66666666666669 |
| Ng 18-11 1 µM_x000d_ | 9806.0 |
| Ng 18-11 3 µM_x000d_ | 50174.0 |
| Ng 20-8 1 µM_x000d_ | 18.333333333333343 |
| Ng 20-9 1 µM_x000d_ | 1331.0 |
| Ng 20-9 3 µM_x000d_ | 12591.666666666666 |
| Ng 21-9 1 µM_x000d_ | 46.66666666666666 |
| Ng 13-13 3 µM_x000d_ | 67.66666666666666 |
| Ng 14-8 1 µM_x000d_ | 0.0 |
| Ng 14-9 1 µM_x000d_ | 0.0 |
| Ng 14-9 3 µM_x000d_ | 26.666666666666657 |
| Ng 14-9 10 µM_x000d_ | 0.0 |
| Ng 14-9 30 µM_x000d_ | 29.666666666666657 |
| Ng 15-10 1µM_x000d_ | 0.0 |
| Ng 16-9 1 µM_x000d_ | 74.0 |
| Ng 17-8 1 µM_x000d_ | 0.0 |
| Ng 17-8 3 µM_x000d_ | 5.333333333333343 |
| Ng 18-9 1µM_x000d_ | 881.3333333333333 |
| Ng 19-11 1 µM_x000d_ | 30653.666666666668 |
| Ng 19-11 3 µM_x000d_ | 58490.666666666664 |
| Lg 17-15 HF 10 µM_x000d_ | 290.0 |
| Lg 15-17 1 µM_x000d_ | 14.333333333333343 |
| Lg 17-15 1 µM_x000d_ | 7.0 |
| Lg 17-15 3 µM_x000d_ | 15.333333333333343 |
| Lg 17-15 10 µM_x000d_ | 990.3333333333333 |
| Lg 17-17 1 µM_x000d_ | 57.66666666666666 |
| Lg 17-16 1 µM_x000d_ | 117.0 |
| Lg 16-17 3 µM_x000d_ | 237.0 |
| Lg 18-8 1 µM_x000d_ | 22.333333333333343 |
| Lg 19-8 1 µM_x000d_ | 14.0 |
| Lg 19-8 3 µM_x000d_ | 84.33333333333334 |
| Lg 19-9 1 µM_x000d_ | 11.666666666666657 |
| Lg 20-10_x000d_ | 75.33333333333334 |
| Lg 22-12 1 µM_x000d_ | 0.0 |
| Lg 20-9 1 µM_x000d_ | 3.0 |
| Lg 23-11 1 µM_x000d_ | 178.33333333333331 |
| Lg 23-11 3 µM_x000d_ | 325.0 |
| Lg 23-12_x000d_ | 259.0 |
| Lg 26-8_x000d_ | 66.66666666666666 |
| Lg 26-9_x000d_ | 53.66666666666666 |
| Lg 22-13_x000d_ | 165.33333333333331 |
| Lg 26-10_x000d_ | 46.0 |
| Lg 18-7-F3 HF 1uM | 0.0 |
| Lg 19-8-F5 HF 3uM | 1.3333333333333428 |
| Lg 23-11-F2 HF 3uM | 0.0 |
| Blank 1 | 0.0 |
| Blank 2 | 0.0 |
| Blank 3 | 0.0 |
| Blank 4 | 0.0 |
| Blank 5 | 0.0 |MFI
### Chart: Ov-4
| Category | Ov k-96 IgG3 |
|---|---|
| Ng 7-13 1µM_x000d_ | 15.333333333333343 |
| Ng 7-13 3µM_x000d_ | 57.0 |
| Ng 9-9 1 µM_x000d_ | 0.0 |
| Ng 9-9 3µM_x000d_ | 0.0 |
| Ng 9-9 10µM_x000d_ | 13.0 |
| Ng 10-13 1 µM_x000d_ | 0.0 |
| Ng 10-13 3 µM_x000d_ | 0.0 |
| Ng 10-13 10 µM_x000d_ | 0.0 |
| Ng 10-13 30 µM_x000d_ | 0.0 |
| Ng 10-10 1 µM_x000d_ | 0.0 |
| Ng 10-10 3 µM_x000d_ | 0.0 |
| Ng 10-11 1 µM_x000d_ | 0.0 |
| Ng 10-11 3 µM_x000d_ | 0.0 |
| Ng 10-12 1 µM_x000d_ | 0.0 |
| Ng 11-10 1 µM_x000d_ | 0.0 |
| Ng 11-10 3 µM_x000d_ | 0.0 |
| Ng 11-11 1 µM_x000d_ | 0.0 |
| Ng 11-11 3µM_x000d_ | 0.0 |
| Ng 11-11 10µM_x000d_ | 0.0 |
| Ng 12-11 1 µM_x000d_ | 0.0 |
| Ng 12-11 3 µM_x000d_ | 0.0 |
| Ng 13-15 1 µM_x000d_ | 0.0 |
| Ng 13-15 3 µM_x000d_ | 0.0 |
| Ng 13-14_x000d_ | 545.3333333333334 |
| Ng 14-10 1 µM_x000d_ | 912.0 |
| Ng 14-10 3 µM_x000d_ | 8528.0 |
| Ng 14-11 1 µM_x000d_ | 261.3333333333333 |
| Ng 14-12 1 µM_x000d_ | 28490.333333333332 |
| Ng 14-7 1 µM_x000d_ | 0.0 |
| Ng 15-14/15 1 µM_x000d_ | 3726.0 |
| Ng 15-12 1µM_x000d_ | 124.66666666666669 |
| Ng 17-7 1 µM_x000d_ | 0.0 |
| Ng 17-7 3 µM_x000d_ | 0.0 |
| Ng 17-7 10 µM_x000d_ | 0.0 |
| Ng 19-9 1 µM_x000d_ | 0.0 |
| Ng 19-9 3 µM_x000d_ | 10.333333333333343 |
| Ng 19-9 10 µM_x000d_ | 535.3333333333334 |
| Ng 20-7 1 µM_x000d_ | 0.0 |
| Ng 20-7 3 µM_x000d_ | 0.0 |
| Ng 20-7 10 µM_x000d_ | 70.66666666666666 |
| Ng 21-8 1 µM_x000d_ | 183.5 |
| Ng 21-8 3 µM_x000d_ | 34.0 |
| Ng 21-8 10 µM_x000d_ | 681.6666666666666 |
| Ng 15-13 3µM_x000d_ | 1630.3333333333333 |
| Ng 16-12 1 µM_x000d_ | 0.0 |
| Ng 17-9 1 µM_x000d_ | 0.3333333333333428 |
| Ng 17-9 3 µM_x000d_ | 216.66666666666669 |
| Ng 18-11 1 µM_x000d_ | 81.33333333333334 |
| Ng 18-11 3 µM_x000d_ | 349.3333333333333 |
| Ng 20-8 1 µM_x000d_ | 309.0 |
| Ng 20-9 1 µM_x000d_ | 813.3333333333334 |
| Ng 20-9 3 µM_x000d_ | 2800.0 |
| Ng 21-9 1 µM_x000d_ | 1784.0 |
| Ng 13-13 3 µM_x000d_ | 0.0 |
| Ng 14-8 1 µM_x000d_ | 0.0 |
| Ng 14-9 1 µM_x000d_ | 0.0 |
| Ng 14-9 3 µM_x000d_ | 14.666666666666657 |
| Ng 14-9 10 µM_x000d_ | 0.0 |
| Ng 14-9 30 µM_x000d_ | 41.66666666666666 |
| Ng 15-10 1µM_x000d_ | 0.0 |
| Ng 16-9 1 µM_x000d_ | 4.333333333333343 |
| Ng 17-8 1 µM_x000d_ | 0.0 |
| Ng 17-8 3 µM_x000d_ | 0.0 |
| Ng 18-9 1µM_x000d_ | 1480.3333333333333 |
| Ng 19-11 1 µM_x000d_ | 6492.0 |
| Ng 19-11 3 µM_x000d_ | 15312.333333333334 |
| Lg 17-15 HF 10 µM_x000d_ | 1809.0 |
| Lg 15-17 1 µM_x000d_ | 720.0 |
| Lg 17-15 1 µM_x000d_ | 59.66666666666666 |
| Lg 17-15 3 µM_x000d_ | 4.333333333333343 |
| Lg 17-15 10 µM_x000d_ | 1526.3333333333333 |
| Lg 17-17 1 µM_x000d_ | 6.666666666666657 |
| Lg 17-16 1 µM_x000d_ | 63.0 |
| Lg 16-17 3 µM_x000d_ | 111.66666666666669 |
| Lg 18-8 1 µM_x000d_ | 83.0 |
| Lg 19-8 1 µM_x000d_ | 0.0 |
| Lg 19-8 3 µM_x000d_ | 73.33333333333334 |
| Lg 19-9 1 µM_x000d_ | 134.66666666666669 |
| Lg 20-10_x000d_ | 54.0 |
| Lg 22-12 1 µM_x000d_ | 0.0 |
| Lg 20-9 1 µM_x000d_ | 0.0 |
| Lg 23-11 1 µM_x000d_ | 0.0 |
| Lg 23-11 3 µM_x000d_ | 36.0 |
| Lg 23-12_x000d_ | 90.33333333333334 |
| Lg 26-8_x000d_ | 22.666666666666657 |
| Lg 26-9_x000d_ | 493.66666666666663 |
| Lg 22-13_x000d_ | 5.666666666666657 |
| Lg 26-10_x000d_ | 54.66666666666666 |
| Lg 18-7-F3 HF 1uM | 0.0 |
| Lg 19-8-F5 HF 3uM | 0.0 |
| Lg 23-11-F2 HF 3uM | 0.0 |
| Blank 1 | 0.0 |
| Blank 2 | 25.0 |
| Blank 3 | 0.0 |
| Blank 4 | 6.666666666666657 |
| Blank 5 | 0.0 |MFI
### Chart: Ov-5
| Category | Ov k-99 IgG3 |
|---|---|
| Ng 7-13 1µM_x000d_ | 0.0 |
| Ng 7-13 3µM_x000d_ | 0.0 |
| Ng 9-9 1 µM_x000d_ | 0.0 |
| Ng 9-9 3µM_x000d_ | 0.0 |
| Ng 9-9 10µM_x000d_ | 0.0 |
| Ng 10-13 1 µM_x000d_ | 0.0 |
| Ng 10-13 3 µM_x000d_ | 0.0 |
| Ng 10-13 10 µM_x000d_ | 0.0 |
| Ng 10-13 30 µM_x000d_ | 0.0 |
| Ng 10-10 1 µM_x000d_ | 0.0 |
| Ng 10-10 3 µM_x000d_ | 0.0 |
| Ng 10-11 1 µM_x000d_ | 0.0 |
| Ng 10-11 3 µM_x000d_ | 0.0 |
| Ng 10-12 1 µM_x000d_ | 0.0 |
| Ng 11-10 1 µM_x000d_ | 0.0 |
| Ng 11-10 3 µM_x000d_ | 0.0 |
| Ng 11-11 1 µM_x000d_ | 0.0 |
| Ng 11-11 3µM_x000d_ | 0.0 |
| Ng 11-11 10µM_x000d_ | 0.0 |
| Ng 12-11 1 µM_x000d_ | 0.0 |
| Ng 12-11 3 µM_x000d_ | 0.0 |
| Ng 13-15 1 µM_x000d_ | 0.0 |
| Ng 13-15 3 µM_x000d_ | 0.0 |
| Ng 13-14_x000d_ | 0.0 |
| Ng 14-10 1 µM_x000d_ | 25.333333333333343 |
| Ng 14-10 3 µM_x000d_ | 820.0 |
| Ng 14-11 1 µM_x000d_ | 0.0 |
| Ng 14-12 1 µM_x000d_ | 1345.3333333333333 |
| Ng 14-7 1 µM_x000d_ | 0.0 |
| Ng 15-14/15 1 µM_x000d_ | 111.66666666666669 |
| Ng 15-12 1µM_x000d_ | 38.33333333333334 |
| Ng 17-7 1 µM_x000d_ | 0.0 |
| Ng 17-7 3 µM_x000d_ | 0.0 |
| Ng 17-7 10 µM_x000d_ | 0.0 |
| Ng 19-9 1 µM_x000d_ | 0.0 |
| Ng 19-9 3 µM_x000d_ | 0.0 |
| Ng 19-9 10 µM_x000d_ | 0.0 |
| Ng 20-7 1 µM_x000d_ | 0.0 |
| Ng 20-7 3 µM_x000d_ | 0.0 |
| Ng 20-7 10 µM_x000d_ | 0.0 |
| Ng 21-8 1 µM_x000d_ | 0.0 |
| Ng 21-8 3 µM_x000d_ | 0.0 |
| Ng 21-8 10 µM_x000d_ | 3.333333333333343 |
| Ng 15-13 3µM_x000d_ | 1066.6666666666667 |
| Ng 16-12 1 µM_x000d_ | 0.0 |
| Ng 17-9 1 µM_x000d_ | 0.0 |
| Ng 17-9 3 µM_x000d_ | 0.0 |
| Ng 18-11 1 µM_x000d_ | 0.0 |
| Ng 18-11 3 µM_x000d_ | 23.666666666666657 |
| Ng 20-8 1 µM_x000d_ | 0.0 |
| Ng 20-9 1 µM_x000d_ | 0.0 |
| Ng 20-9 3 µM_x000d_ | 0.0 |
| Ng 21-9 1 µM_x000d_ | 0.0 |
| Ng 13-13 3 µM_x000d_ | 0.0 |
| Ng 14-8 1 µM_x000d_ | 0.0 |
| Ng 14-9 1 µM_x000d_ | 0.0 |
| Ng 14-9 3 µM_x000d_ | 0.0 |
| Ng 14-9 10 µM_x000d_ | 0.0 |
| Ng 14-9 30 µM_x000d_ | 0.0 |
| Ng 15-10 1µM_x000d_ | 0.0 |
| Ng 16-9 1 µM_x000d_ | 0.0 |
| Ng 17-8 1 µM_x000d_ | 0.0 |
| Ng 17-8 3 µM_x000d_ | 0.0 |
| Ng 18-9 1µM_x000d_ | 827.0 |
| Ng 19-11 1 µM_x000d_ | 49.0 |
| Ng 19-11 3 µM_x000d_ | 152.33333333333331 |
| Lg 17-15 HF 10 µM_x000d_ | 26444.333333333332 |
| Lg 15-17 1 µM_x000d_ | 435.33333333333337 |
| Lg 17-15 1 µM_x000d_ | 2785.3333333333335 |
| Lg 17-15 3 µM_x000d_ | 13637.333333333334 |
| Lg 17-15 10 µM_x000d_ | 23947.666666666668 |
| Lg 17-17 1 µM_x000d_ | 3194.0 |
| Lg 17-16 1 µM_x000d_ | 5874.666666666667 |
| Lg 16-17 3 µM_x000d_ | 6883.666666666667 |
| Lg 18-8 1 µM_x000d_ | 3345.6666666666665 |
| Lg 19-8 1 µM_x000d_ | 0.0 |
| Lg 19-8 3 µM_x000d_ | 206.66666666666669 |
| Lg 19-9 1 µM_x000d_ | 4053.666666666667 |
| Lg 20-10_x000d_ | 598.0 |
| Lg 22-12 1 µM_x000d_ | 0.0 |
| Lg 20-9 1 µM_x000d_ | 1789.0 |
| Lg 23-11 1 µM_x000d_ | 0.0 |
| Lg 23-11 3 µM_x000d_ | 256.6666666666667 |
| Lg 23-12_x000d_ | 2924.6666666666665 |
| Lg 26-8_x000d_ | 2.333333333333343 |
| Lg 26-9_x000d_ | 0.0 |
| Lg 22-13_x000d_ | 3760.0 |
| Lg 26-10_x000d_ | 0.0 |
| Lg 18-7-F3 HF 1uM | 0.0 |
| Lg 19-8-F5 HF 3uM | 0.0 |
| Lg 23-11-F2 HF 3uM | 0.0 |
| Blank 1 | 0.0 |
| Blank 2 | 0.0 |
| Blank 3 | 0.0 |
| Blank 4 | 0.0 |
| Blank 5 | 0.0 |MFI
### Chart: Ov-6
| Category | Ov k-100 IgG3 |
|---|---|
| Ng1_1uM
 | 0.0 |
| Ng1_3uM
 | 24.666666666666657 |
| Ng2_1uM | 0.0 |
| Ng2_3uM | 0.0 |
| Ng2_10uM | 0.0 |
| Ng3_1uM | 0.0 |
| Ng3_3uM | 0.0 |
| Ng3_10uM | 0.0 |
| Ng3_30uM | 0.0 |
| Ng4_1uM | 0.0 |
| Ng4_3uM | 0.0 |
| Ng5_1uM | 0.0 |
| Ng5_3uM | 0.0 |
| Ng6_1uM | 0.0 |
| Ng7_1uM | 0.0 |
| Ng7_3uM | 0.0 |
| Ng8_1uM | 0.0 |
| Ng8_3uM | 0.0 |
| Ng8_10uM | 0.0 |
| Ng9_1uM | 0.0 |
| Ng9_3uM | 0.0 |
| Ng10_3uM | 0.0 |
| Ng10_1uM | 0.0 |
| Ng11 | 1263.6666666666667 |
| Ng12_1uM | 1985.3333333333335 |
| Ng12_3uM | 9699.666666666666 |
| Ng13_1uM | 554.3333333333334 |
| Ng14_1uM | 20212.666666666668 |
| Ng15_1uM | 0.0 |
| Ng16_1uM | 4853.666666666667 |
| Ng17_1uM | 100.66666666666666 |
| Ng18_1uM | 0.0 |
| Ng18_3uM | 0.0 |
| Ng18_10uM | 0.0 |
| Ng19_1uM | 0.0 |
| Ng19_3uM | 8.0 |
| Ng19_10uM | 241.66666666666669 |
| Ng20_1uM | 0.0 |
| Ng20_3uM | 0.0 |
| Ng20_10uM | 53.33333333333334 |
| Ng21_1uM | 0.0 |
| Ng21 _3uM | 0.0 |
| Ng21_10uM | 589.3333333333334 |
| Ng22_1uM | 1752.6666666666667 |
| Ng23_1uM | 0.0 |
| Ng24_1uM | 26.0 |
| Ng24_3uM | 328.3333333333333 |
| Ng25_1uM | 233.66666666666669 |
| Ng25_3uM | 212.0 |
| Ng26_1uM | 949.0 |
| Ng27_1uM | 948.0 |
| Ng27_3uM | 2300.6666666666665 |
| Ng28_1uM | 3584.6666666666665 |
| Ng29_1uM | 0.0 |
| Ng30_1uM | 0.0 |
| Ng31_1uM | 0.0 |
| Ng31_3uM | 0.0 |
| Ng31_10uM | 0.0 |
| Ng31_30uM | 0.0 |
| Ng32_1uM | 0.0 |
| Ng33_1uM | 0.0 |
| Ng34_1uM | 0.0 |
| Ng34_3uM | 0.0 |
| Ng35_1uM | 1312.3333333333333 |
| Ng36_1uM | 5034.666666666667 |
| Ng36_3uM | 10099.333333333334 |
| Lg2_HF_10uM | 14.666666666666657 |
| Lg1_1uM | 0.0 |
| Lg2_1uM | 0.0 |
| Lg2_3uM | 15.666666666666657 |
| Lg2_10uM | 479.0 |
| Lg3_1uM | 0.0 |
| Lg4_1uM | 598.6666666666666 |
| Lg5_3uM | 5033.666666666667 |
| Lg6_1uM | 0.0 |
| Lg7_1uM | 0.0 |
| Lg7_3uM | 0.0 |
| Lg8_1uM | 16.333333333333343 |
| Lg9 | 0.0 |
| Lg10_1uM | 0.0 |
| Lg11_1uM | 0.0 |
| Lg12_1uM | 0.0 |
| Lg12_3uM | 18.0 |
| Lg13 | 367.0 |
| Lg14 | 11.333333333333343 |
| Lg15 | 1150.6666666666667 |
| Lg16 | 170.0 |
| Lg17 | 891.0 |
| Lg18_HF_1uM | 0.0 |
| Lg7_HF_3uM | 0.0 |
| Lg12_HF_3uM | 0.0 |
| Blank 1 | 0.0 |
| Blank 2 | 0.0 |
| Blank 3 | 0.0 |
| Blank 4 | 0.0 |
| Blank 5 | 0.0 |MFI

## Slide 27
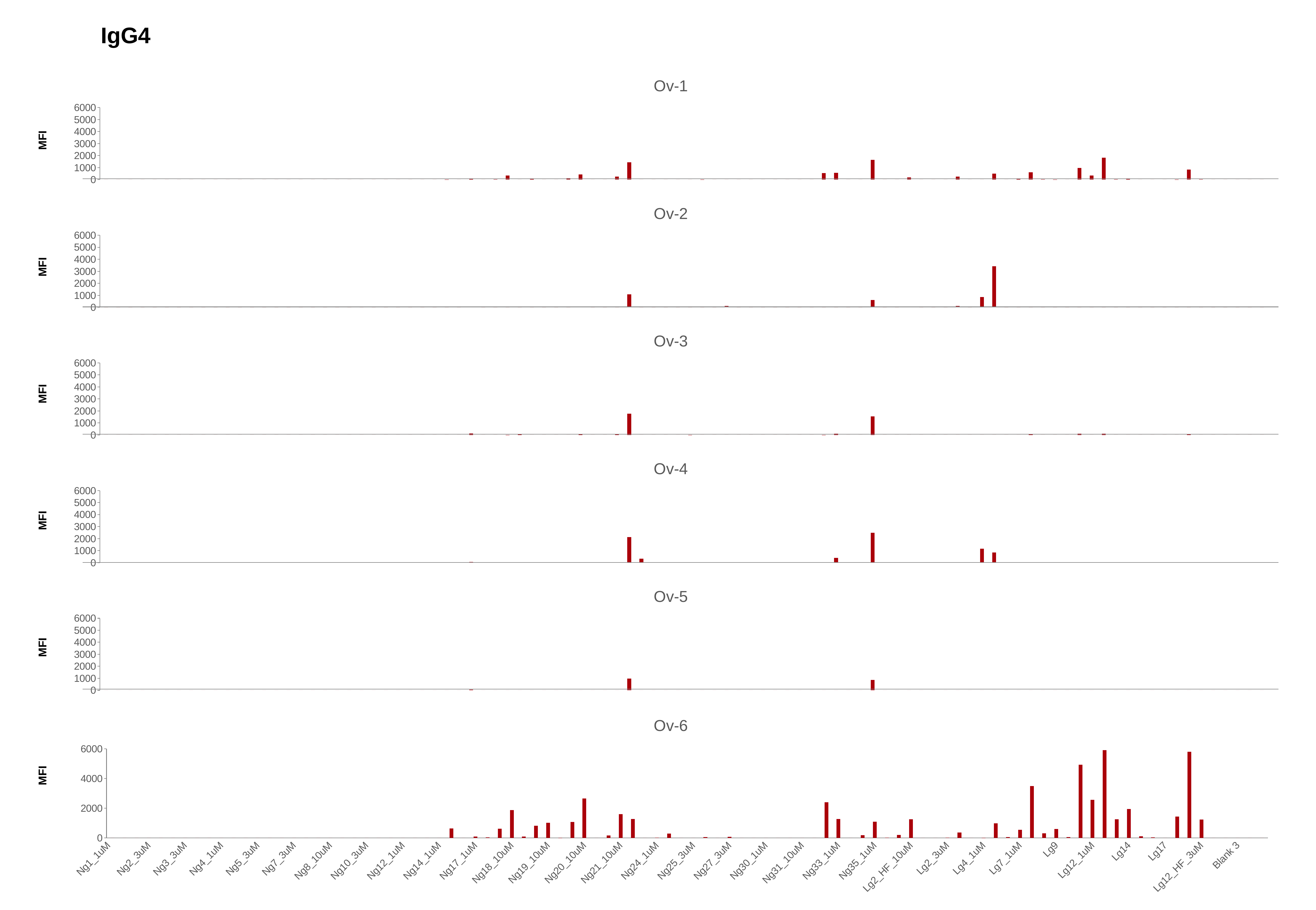

IgG4
### Chart: Ov-1
| Category | Ov 38-207 IgG4 |
|---|---|
| Ng 7-13 1µM_x000d_ | 0.0 |
| Ng 7-13 3µM_x000d_ | 0.0 |
| Ng 9-9 1 µM_x000d_ | 0.0 |
| Ng 9-9 3µM_x000d_ | 0.0 |
| Ng 9-9 10µM_x000d_ | 0.0 |
| Ng 10-13 1 µM_x000d_ | 0.0 |
| Ng 10-13 3 µM_x000d_ | 0.0 |
| Ng 10-13 10 µM_x000d_ | 0.0 |
| Ng 10-13 30 µM_x000d_ | 0.0 |
| Ng 10-10 1 µM_x000d_ | 0.0 |
| Ng 10-10 3 µM_x000d_ | 0.0 |
| Ng 10-11 1 µM_x000d_ | 0.0 |
| Ng 10-11 3 µM_x000d_ | 0.0 |
| Ng 10-12 1 µM_x000d_ | 0.0 |
| Ng 11-10 1 µM_x000d_ | 0.0 |
| Ng 11-10 3 µM_x000d_ | 0.0 |
| Ng 11-11 1 µM_x000d_ | 0.0 |
| Ng 11-11 3µM_x000d_ | 0.0 |
| Ng 11-11 10µM_x000d_ | 0.0 |
| Ng 12-11 1 µM_x000d_ | 0.0 |
| Ng 12-11 3 µM_x000d_ | 0.0 |
| Ng 13-15 1 µM_x000d_ | 0.0 |
| Ng 13-15 3 µM_x000d_ | 0.0 |
| Ng 13-14_x000d_ | 0.0 |
| Ng 14-10 1 µM_x000d_ | 0.0 |
| Ng 14-10 3 µM_x000d_ | 0.0 |
| Ng 14-11 1 µM_x000d_ | 0.0 |
| Ng 14-12 1 µM_x000d_ | 0.0 |
| Ng 14-7 1 µM_x000d_ | 4.333333333333343 |
| Ng 15-14/15 1 µM_x000d_ | 0.0 |
| Ng 15-12 1µM_x000d_ | 98.66666666666666 |
| Ng 17-7 1 µM_x000d_ | 0.0 |
| Ng 17-7 3 µM_x000d_ | 27.0 |
| Ng 17-7 10 µM_x000d_ | 342.3333333333333 |
| Ng 19-9 1 µM_x000d_ | 0.0 |
| Ng 19-9 3 µM_x000d_ | 88.0 |
| Ng 19-9 10 µM_x000d_ | 0.0 |
| Ng 20-7 1 µM_x000d_ | 0.0 |
| Ng 20-7 3 µM_x000d_ | 128.66666666666669 |
| Ng 20-7 10 µM_x000d_ | 440.66666666666663 |
| Ng 21-8 1 µM_x000d_ | 0.0 |
| Ng 21-8 3 µM_x000d_ | 0.0 |
| Ng 21-8 10 µM_x000d_ | 252.33333333333331 |
| Ng 15-13 3µM_x000d_ | 1442.6666666666667 |
| Ng 16-12 1 µM_x000d_ | 0.0 |
| Ng 17-9 1 µM_x000d_ | 0.0 |
| Ng 17-9 3 µM_x000d_ | 0.0 |
| Ng 18-11 1 µM_x000d_ | 0.0 |
| Ng 18-11 3 µM_x000d_ | 0.0 |
| Ng 20-8 1 µM_x000d_ | 7.333333333333343 |
| Ng 20-9 1 µM_x000d_ | 0.0 |
| Ng 20-9 3 µM_x000d_ | 0.0 |
| Ng 21-9 1 µM_x000d_ | 0.0 |
| Ng 13-13 3 µM_x000d_ | 0.0 |
| Ng 14-8 1 µM_x000d_ | 0.0 |
| Ng 14-9 1 µM_x000d_ | 0.0 |
| Ng 14-9 3 µM_x000d_ | 0.0 |
| Ng 14-9 10 µM_x000d_ | 0.0 |
| Ng 14-9 30 µM_x000d_ | 0.0 |
| Ng 15-10 1µM_x000d_ | 556.6666666666666 |
| Ng 16-9 1 µM_x000d_ | 580.3333333333334 |
| Ng 17-8 1 µM_x000d_ | 0.0 |
| Ng 17-8 3 µM_x000d_ | 0.0 |
| Ng 18-9 1µM_x000d_ | 1650.3333333333333 |
| Ng 19-11 1 µM_x000d_ | 0.0 |
| Ng 19-11 3 µM_x000d_ | 0.0 |
| Lg 17-15 HF 10 µM_x000d_ | 195.33333333333331 |
| Lg 15-17 1 µM_x000d_ | 0.0 |
| Lg 17-15 1 µM_x000d_ | 0.0 |
| Lg 17-15 3 µM_x000d_ | 0.0 |
| Lg 17-15 10 µM_x000d_ | 252.0 |
| Lg 17-17 1 µM_x000d_ | 0.0 |
| Lg 17-16 1 µM_x000d_ | 0.0 |
| Lg 16-17 3 µM_x000d_ | 508.0 |
| Lg 18-8 1 µM_x000d_ | 0.0 |
| Lg 19-8 1 µM_x000d_ | 46.0 |
| Lg 19-8 3 µM_x000d_ | 615.0 |
| Lg 19-9 1 µM_x000d_ | 24.333333333333343 |
| Lg 20-10_x000d_ | 11.666666666666657 |
| Lg 22-12 1 µM_x000d_ | 0.0 |
| Lg 20-9 1 µM_x000d_ | 965.0 |
| Lg 23-11 1 µM_x000d_ | 342.3333333333333 |
| Lg 23-11 3 µM_x000d_ | 1837.6666666666667 |
| Lg 23-12_x000d_ | 37.33333333333334 |
| Lg 26-8_x000d_ | 65.33333333333334 |
| Lg 26-9_x000d_ | 0.0 |
| Lg 22-13_x000d_ | 0.0 |
| Lg 26-10_x000d_ | 0.0 |
| Lg 18-7-F3 HF 1uM | 1.6666666666666572 |
| Lg 19-8-F5 HF 3uM | 845.0 |
| Lg 23-11-F2 HF 3uM | 28.333333333333343 |
| Blank 1 | 0.0 |
| Blank 2 | 0.0 |
| Blank 3 | 0.0 |
| Blank 4 | 0.0 |
| Blank 5 | 0.0 |MFI
### Chart: Ov-2
| Category | Ov k44 IgG4 |
|---|---|
| Ng 7-13 1µM_x000d_ | 0.0 |
| Ng 7-13 3µM_x000d_ | 0.0 |
| Ng 9-9 1 µM_x000d_ | 0.0 |
| Ng 9-9 3µM_x000d_ | 0.0 |
| Ng 9-9 10µM_x000d_ | 0.0 |
| Ng 10-13 1 µM_x000d_ | 0.0 |
| Ng 10-13 3 µM_x000d_ | 0.0 |
| Ng 10-13 10 µM_x000d_ | 0.0 |
| Ng 10-13 30 µM_x000d_ | 0.0 |
| Ng 10-10 1 µM_x000d_ | 0.0 |
| Ng 10-10 3 µM_x000d_ | 0.0 |
| Ng 10-11 1 µM_x000d_ | 0.0 |
| Ng 10-11 3 µM_x000d_ | 0.0 |
| Ng 10-12 1 µM_x000d_ | 0.0 |
| Ng 11-10 1 µM_x000d_ | 0.0 |
| Ng 11-10 3 µM_x000d_ | 0.0 |
| Ng 11-11 1 µM_x000d_ | 0.0 |
| Ng 11-11 3µM_x000d_ | 0.0 |
| Ng 11-11 10µM_x000d_ | 0.0 |
| Ng 12-11 1 µM_x000d_ | 0.0 |
| Ng 12-11 3 µM_x000d_ | 0.0 |
| Ng 13-15 1 µM_x000d_ | 0.0 |
| Ng 13-15 3 µM_x000d_ | 0.0 |
| Ng 13-14_x000d_ | 0.0 |
| Ng 14-10 1 µM_x000d_ | 0.0 |
| Ng 14-10 3 µM_x000d_ | 0.0 |
| Ng 14-11 1 µM_x000d_ | 0.0 |
| Ng 14-12 1 µM_x000d_ | 0.0 |
| Ng 14-7 1 µM_x000d_ | 0.0 |
| Ng 15-14/15 1 µM_x000d_ | 0.0 |
| Ng 15-12 1µM_x000d_ | 26.666666666666657 |
| Ng 17-7 1 µM_x000d_ | 0.0 |
| Ng 17-7 3 µM_x000d_ | 0.0 |
| Ng 17-7 10 µM_x000d_ | 0.0 |
| Ng 19-9 1 µM_x000d_ | 0.0 |
| Ng 19-9 3 µM_x000d_ | 0.0 |
| Ng 19-9 10 µM_x000d_ | 0.0 |
| Ng 20-7 1 µM_x000d_ | 0.0 |
| Ng 20-7 3 µM_x000d_ | 0.0 |
| Ng 20-7 10 µM_x000d_ | 27.666666666666657 |
| Ng 21-8 1 µM_x000d_ | 0.0 |
| Ng 21-8 3 µM_x000d_ | 0.0 |
| Ng 21-8 10 µM_x000d_ | 0.0 |
| Ng 15-13 3µM_x000d_ | 1085.6666666666667 |
| Ng 16-12 1 µM_x000d_ | 0.0 |
| Ng 17-9 1 µM_x000d_ | 0.0 |
| Ng 17-9 3 µM_x000d_ | 0.0 |
| Ng 18-11 1 µM_x000d_ | 0.0 |
| Ng 18-11 3 µM_x000d_ | 0.0 |
| Ng 20-8 1 µM_x000d_ | 0.0 |
| Ng 20-9 1 µM_x000d_ | 0.0 |
| Ng 20-9 3 µM_x000d_ | 123.0 |
| Ng 21-9 1 µM_x000d_ | 0.0 |
| Ng 13-13 3 µM_x000d_ | 0.0 |
| Ng 14-8 1 µM_x000d_ | 0.0 |
| Ng 14-9 1 µM_x000d_ | 0.0 |
| Ng 14-9 3 µM_x000d_ | 0.0 |
| Ng 14-9 10 µM_x000d_ | 0.0 |
| Ng 14-9 30 µM_x000d_ | 0.0 |
| Ng 15-10 1µM_x000d_ | 0.0 |
| Ng 16-9 1 µM_x000d_ | 0.0 |
| Ng 17-8 1 µM_x000d_ | 0.0 |
| Ng 17-8 3 µM_x000d_ | 0.0 |
| Ng 18-9 1µM_x000d_ | 616.6666666666666 |
| Ng 19-11 1 µM_x000d_ | 0.0 |
| Ng 19-11 3 µM_x000d_ | 0.0 |
| Lg 17-15 HF 10 µM_x000d_ | 23.0 |
| Lg 15-17 1 µM_x000d_ | 0.0 |
| Lg 17-15 1 µM_x000d_ | 0.0 |
| Lg 17-15 3 µM_x000d_ | 0.0 |
| Lg 17-15 10 µM_x000d_ | 122.66666666666669 |
| Lg 17-17 1 µM_x000d_ | 0.0 |
| Lg 17-16 1 µM_x000d_ | 853.6666666666666 |
| Lg 16-17 3 µM_x000d_ | 3423.0 |
| Lg 18-8 1 µM_x000d_ | 0.0 |
| Lg 19-8 1 µM_x000d_ | 0.0 |
| Lg 19-8 3 µM_x000d_ | 0.0 |
| Lg 19-9 1 µM_x000d_ | 0.0 |
| Lg 20-10_x000d_ | 0.0 |
| Lg 22-12 1 µM_x000d_ | 0.0 |
| Lg 20-9 1 µM_x000d_ | 0.0 |
| Lg 23-11 1 µM_x000d_ | 0.0 |
| Lg 23-11 3 µM_x000d_ | 0.0 |
| Lg 23-12_x000d_ | 0.0 |
| Lg 26-8_x000d_ | 0.0 |
| Lg 26-9_x000d_ | 0.0 |
| Lg 22-13_x000d_ | 0.0 |
| Lg 26-10_x000d_ | 0.0 |
| Lg 18-7-F3 HF 1uM | 0.0 |
| Lg 19-8-F5 HF 3uM | 0.0 |
| Lg 23-11-F2 HF 3uM | 0.0 |
| Blank 1 | 0.0 |
| Blank 2 | 0.0 |
| Blank 3 | 0.0 |
| Blank 4 | 0.0 |
| Blank 5 | 0.0 |MFI
### Chart: Ov-3
| Category | Ov k-84 IgG4 |
|---|---|
| Ng 7-13 1µM_x000d_ | 0.0 |
| Ng 7-13 3µM_x000d_ | 0.0 |
| Ng 9-9 1 µM_x000d_ | 0.0 |
| Ng 9-9 3µM_x000d_ | 0.0 |
| Ng 9-9 10µM_x000d_ | 0.0 |
| Ng 10-13 1 µM_x000d_ | 0.0 |
| Ng 10-13 3 µM_x000d_ | 0.0 |
| Ng 10-13 10 µM_x000d_ | 0.0 |
| Ng 10-13 30 µM_x000d_ | 0.0 |
| Ng 10-10 1 µM_x000d_ | 0.0 |
| Ng 10-10 3 µM_x000d_ | 0.0 |
| Ng 10-11 1 µM_x000d_ | 0.0 |
| Ng 10-11 3 µM_x000d_ | 0.0 |
| Ng 10-12 1 µM_x000d_ | 0.0 |
| Ng 11-10 1 µM_x000d_ | 0.0 |
| Ng 11-10 3 µM_x000d_ | 0.0 |
| Ng 11-11 1 µM_x000d_ | 0.0 |
| Ng 11-11 3µM_x000d_ | 0.0 |
| Ng 11-11 10µM_x000d_ | 0.0 |
| Ng 12-11 1 µM_x000d_ | 0.0 |
| Ng 12-11 3 µM_x000d_ | 0.0 |
| Ng 13-15 1 µM_x000d_ | 0.0 |
| Ng 13-15 3 µM_x000d_ | 0.0 |
| Ng 13-14_x000d_ | 0.0 |
| Ng 14-10 1 µM_x000d_ | 0.0 |
| Ng 14-10 3 µM_x000d_ | 0.0 |
| Ng 14-11 1 µM_x000d_ | 0.0 |
| Ng 14-12 1 µM_x000d_ | 0.0 |
| Ng 14-7 1 µM_x000d_ | 0.0 |
| Ng 15-14/15 1 µM_x000d_ | 0.0 |
| Ng 15-12 1µM_x000d_ | 123.0 |
| Ng 17-7 1 µM_x000d_ | 0.0 |
| Ng 17-7 3 µM_x000d_ | 0.0 |
| Ng 17-7 10 µM_x000d_ | 4.0 |
| Ng 19-9 1 µM_x000d_ | 63.0 |
| Ng 19-9 3 µM_x000d_ | 0.0 |
| Ng 19-9 10 µM_x000d_ | 0.0 |
| Ng 20-7 1 µM_x000d_ | 0.0 |
| Ng 20-7 3 µM_x000d_ | 0.0 |
| Ng 20-7 10 µM_x000d_ | 38.0 |
| Ng 21-8 1 µM_x000d_ | 0.0 |
| Ng 21-8 3 µM_x000d_ | 0.0 |
| Ng 21-8 10 µM_x000d_ | 58.66666666666666 |
| Ng 15-13 3µM_x000d_ | 1767.0 |
| Ng 16-12 1 µM_x000d_ | 0.0 |
| Ng 17-9 1 µM_x000d_ | 0.0 |
| Ng 17-9 3 µM_x000d_ | 0.0 |
| Ng 18-11 1 µM_x000d_ | 0.0 |
| Ng 18-11 3 µM_x000d_ | 2.333333333333343 |
| Ng 20-8 1 µM_x000d_ | 0.0 |
| Ng 20-9 1 µM_x000d_ | 0.0 |
| Ng 20-9 3 µM_x000d_ | 0.0 |
| Ng 21-9 1 µM_x000d_ | 0.0 |
| Ng 13-13 3 µM_x000d_ | 0.0 |
| Ng 14-8 1 µM_x000d_ | 0.0 |
| Ng 14-9 1 µM_x000d_ | 0.0 |
| Ng 14-9 3 µM_x000d_ | 0.0 |
| Ng 14-9 10 µM_x000d_ | 0.0 |
| Ng 14-9 30 µM_x000d_ | 0.0 |
| Ng 15-10 1µM_x000d_ | 1.0 |
| Ng 16-9 1 µM_x000d_ | 114.66666666666669 |
| Ng 17-8 1 µM_x000d_ | 0.0 |
| Ng 17-8 3 µM_x000d_ | 0.0 |
| Ng 18-9 1µM_x000d_ | 1557.6666666666667 |
| Ng 19-11 1 µM_x000d_ | 0.0 |
| Ng 19-11 3 µM_x000d_ | 0.0 |
| Lg 17-15 HF 10 µM_x000d_ | 0.0 |
| Lg 15-17 1 µM_x000d_ | 0.0 |
| Lg 17-15 1 µM_x000d_ | 0.0 |
| Lg 17-15 3 µM_x000d_ | 0.0 |
| Lg 17-15 10 µM_x000d_ | 0.0 |
| Lg 17-17 1 µM_x000d_ | 0.0 |
| Lg 17-16 1 µM_x000d_ | 0.0 |
| Lg 16-17 3 µM_x000d_ | 0.0 |
| Lg 18-8 1 µM_x000d_ | 0.0 |
| Lg 19-8 1 µM_x000d_ | 0.0 |
| Lg 19-8 3 µM_x000d_ | 35.66666666666666 |
| Lg 19-9 1 µM_x000d_ | 0.0 |
| Lg 20-10_x000d_ | 0.0 |
| Lg 22-12 1 µM_x000d_ | 0.0 |
| Lg 20-9 1 µM_x000d_ | 108.0 |
| Lg 23-11 1 µM_x000d_ | 0.0 |
| Lg 23-11 3 µM_x000d_ | 120.66666666666669 |
| Lg 23-12_x000d_ | 0.0 |
| Lg 26-8_x000d_ | 0.0 |
| Lg 26-9_x000d_ | 0.0 |
| Lg 22-13_x000d_ | 0.0 |
| Lg 26-10_x000d_ | 0.0 |
| Lg 18-7-F3 HF 1uM | 0.0 |
| Lg 19-8-F5 HF 3uM | 74.33333333333334 |
| Lg 23-11-F2 HF 3uM | 0.0 |
| Blank 1 | 0.0 |
| Blank 2 | 0.0 |
| Blank 3 | 0.0 |
| Blank 4 | 0.0 |
| Blank 5 | 0.0 |MFI
### Chart: Ov-4
| Category | Ov k-96 IgG4 |
|---|---|
| Ng 7-13 1µM_x000d_ | 0.0 |
| Ng 7-13 3µM_x000d_ | 0.0 |
| Ng 9-9 1 µM_x000d_ | 0.0 |
| Ng 9-9 3µM_x000d_ | 0.0 |
| Ng 9-9 10µM_x000d_ | 0.0 |
| Ng 10-13 1 µM_x000d_ | 0.0 |
| Ng 10-13 3 µM_x000d_ | 0.0 |
| Ng 10-13 10 µM_x000d_ | 0.0 |
| Ng 10-13 30 µM_x000d_ | 0.0 |
| Ng 10-10 1 µM_x000d_ | 0.0 |
| Ng 10-10 3 µM_x000d_ | 0.0 |
| Ng 10-11 1 µM_x000d_ | 0.0 |
| Ng 10-11 3 µM_x000d_ | 0.0 |
| Ng 10-12 1 µM_x000d_ | 0.0 |
| Ng 11-10 1 µM_x000d_ | 0.0 |
| Ng 11-10 3 µM_x000d_ | 0.0 |
| Ng 11-11 1 µM_x000d_ | 0.0 |
| Ng 11-11 3µM_x000d_ | 0.0 |
| Ng 11-11 10µM_x000d_ | 0.0 |
| Ng 12-11 1 µM_x000d_ | 0.0 |
| Ng 12-11 3 µM_x000d_ | 0.0 |
| Ng 13-15 1 µM_x000d_ | 0.0 |
| Ng 13-15 3 µM_x000d_ | 0.0 |
| Ng 13-14_x000d_ | 0.0 |
| Ng 14-10 1 µM_x000d_ | 0.0 |
| Ng 14-10 3 µM_x000d_ | 0.0 |
| Ng 14-11 1 µM_x000d_ | 0.0 |
| Ng 14-12 1 µM_x000d_ | 0.0 |
| Ng 14-7 1 µM_x000d_ | 0.0 |
| Ng 15-14/15 1 µM_x000d_ | 0.0 |
| Ng 15-12 1µM_x000d_ | 69.0 |
| Ng 17-7 1 µM_x000d_ | 0.0 |
| Ng 17-7 3 µM_x000d_ | 0.0 |
| Ng 17-7 10 µM_x000d_ | 0.0 |
| Ng 19-9 1 µM_x000d_ | 0.0 |
| Ng 19-9 3 µM_x000d_ | 0.0 |
| Ng 19-9 10 µM_x000d_ | 0.0 |
| Ng 20-7 1 µM_x000d_ | 0.0 |
| Ng 20-7 3 µM_x000d_ | 0.0 |
| Ng 20-7 10 µM_x000d_ | 0.0 |
| Ng 21-8 1 µM_x000d_ | 0.0 |
| Ng 21-8 3 µM_x000d_ | 0.0 |
| Ng 21-8 10 µM_x000d_ | 0.0 |
| Ng 15-13 3µM_x000d_ | 2123.6666666666665 |
| Ng 16-12 1 µM_x000d_ | 340.6666666666667 |
| Ng 17-9 1 µM_x000d_ | 0.0 |
| Ng 17-9 3 µM_x000d_ | 0.0 |
| Ng 18-11 1 µM_x000d_ | 0.0 |
| Ng 18-11 3 µM_x000d_ | 0.0 |
| Ng 20-8 1 µM_x000d_ | 0.0 |
| Ng 20-9 1 µM_x000d_ | 0.0 |
| Ng 20-9 3 µM_x000d_ | 0.0 |
| Ng 21-9 1 µM_x000d_ | 0.0 |
| Ng 13-13 3 µM_x000d_ | 0.0 |
| Ng 14-8 1 µM_x000d_ | 0.0 |
| Ng 14-9 1 µM_x000d_ | 0.0 |
| Ng 14-9 3 µM_x000d_ | 0.0 |
| Ng 14-9 10 µM_x000d_ | 0.0 |
| Ng 14-9 30 µM_x000d_ | 0.0 |
| Ng 15-10 1µM_x000d_ | 0.0 |
| Ng 16-9 1 µM_x000d_ | 408.66666666666663 |
| Ng 17-8 1 µM_x000d_ | 0.0 |
| Ng 17-8 3 µM_x000d_ | 0.0 |
| Ng 18-9 1µM_x000d_ | 2492.0 |
| Ng 19-11 1 µM_x000d_ | 0.0 |
| Ng 19-11 3 µM_x000d_ | 0.0 |
| Lg 17-15 HF 10 µM_x000d_ | 0.0 |
| Lg 15-17 1 µM_x000d_ | 0.0 |
| Lg 17-15 1 µM_x000d_ | 0.0 |
| Lg 17-15 3 µM_x000d_ | 0.0 |
| Lg 17-15 10 µM_x000d_ | 0.0 |
| Lg 17-17 1 µM_x000d_ | 0.0 |
| Lg 17-16 1 µM_x000d_ | 1171.0 |
| Lg 16-17 3 µM_x000d_ | 848.0 |
| Lg 18-8 1 µM_x000d_ | 0.0 |
| Lg 19-8 1 µM_x000d_ | 0.0 |
| Lg 19-8 3 µM_x000d_ | 0.0 |
| Lg 19-9 1 µM_x000d_ | 0.0 |
| Lg 20-10_x000d_ | 0.0 |
| Lg 22-12 1 µM_x000d_ | 0.0 |
| Lg 20-9 1 µM_x000d_ | 0.0 |
| Lg 23-11 1 µM_x000d_ | 0.0 |
| Lg 23-11 3 µM_x000d_ | 0.0 |
| Lg 23-12_x000d_ | 0.0 |
| Lg 26-8_x000d_ | 0.0 |
| Lg 26-9_x000d_ | 0.0 |
| Lg 22-13_x000d_ | 0.0 |
| Lg 26-10_x000d_ | 0.0 |
| Lg 18-7-F3 HF 1uM | 0.0 |
| Lg 19-8-F5 HF 3uM | 0.0 |
| Lg 23-11-F2 HF 3uM | 0.0 |
| Blank 1 | 0.0 |
| Blank 2 | 0.0 |
| Blank 3 | 0.0 |
| Blank 4 | 0.0 |
| Blank 5 | 0.0 |MFI
### Chart: Ov-5
| Category | Ov k-99 IgG4 |
|---|---|
| Ng 7-13 1µM_x000d_ | 0.0 |
| Ng 7-13 3µM_x000d_ | 0.0 |
| Ng 9-9 1 µM_x000d_ | 0.0 |
| Ng 9-9 3µM_x000d_ | 0.0 |
| Ng 9-9 10µM_x000d_ | 0.0 |
| Ng 10-13 1 µM_x000d_ | 0.0 |
| Ng 10-13 3 µM_x000d_ | 0.0 |
| Ng 10-13 10 µM_x000d_ | 0.0 |
| Ng 10-13 30 µM_x000d_ | 0.0 |
| Ng 10-10 1 µM_x000d_ | 0.0 |
| Ng 10-10 3 µM_x000d_ | 0.0 |
| Ng 10-11 1 µM_x000d_ | 0.0 |
| Ng 10-11 3 µM_x000d_ | 0.0 |
| Ng 10-12 1 µM_x000d_ | 0.0 |
| Ng 11-10 1 µM_x000d_ | 0.0 |
| Ng 11-10 3 µM_x000d_ | 0.0 |
| Ng 11-11 1 µM_x000d_ | 0.0 |
| Ng 11-11 3µM_x000d_ | 0.0 |
| Ng 11-11 10µM_x000d_ | 0.0 |
| Ng 12-11 1 µM_x000d_ | 0.0 |
| Ng 12-11 3 µM_x000d_ | 0.0 |
| Ng 13-15 1 µM_x000d_ | 0.0 |
| Ng 13-15 3 µM_x000d_ | 0.0 |
| Ng 13-14_x000d_ | 0.0 |
| Ng 14-10 1 µM_x000d_ | 0.0 |
| Ng 14-10 3 µM_x000d_ | 0.0 |
| Ng 14-11 1 µM_x000d_ | 0.0 |
| Ng 14-12 1 µM_x000d_ | 0.0 |
| Ng 14-7 1 µM_x000d_ | 0.0 |
| Ng 15-14/15 1 µM_x000d_ | 0.0 |
| Ng 15-12 1µM_x000d_ | 56.66666666666666 |
| Ng 17-7 1 µM_x000d_ | 0.0 |
| Ng 17-7 3 µM_x000d_ | 0.0 |
| Ng 17-7 10 µM_x000d_ | 0.0 |
| Ng 19-9 1 µM_x000d_ | 0.0 |
| Ng 19-9 3 µM_x000d_ | 0.0 |
| Ng 19-9 10 µM_x000d_ | 0.0 |
| Ng 20-7 1 µM_x000d_ | 0.0 |
| Ng 20-7 3 µM_x000d_ | 0.0 |
| Ng 20-7 10 µM_x000d_ | 0.0 |
| Ng 21-8 1 µM_x000d_ | 0.0 |
| Ng 21-8 3 µM_x000d_ | 0.0 |
| Ng 21-8 10 µM_x000d_ | 0.0 |
| Ng 15-13 3µM_x000d_ | 983.6666666666667 |
| Ng 16-12 1 µM_x000d_ | 0.0 |
| Ng 17-9 1 µM_x000d_ | 0.0 |
| Ng 17-9 3 µM_x000d_ | 0.0 |
| Ng 18-11 1 µM_x000d_ | 0.0 |
| Ng 18-11 3 µM_x000d_ | 0.0 |
| Ng 20-8 1 µM_x000d_ | 0.0 |
| Ng 20-9 1 µM_x000d_ | 0.0 |
| Ng 20-9 3 µM_x000d_ | 0.0 |
| Ng 21-9 1 µM_x000d_ | 0.0 |
| Ng 13-13 3 µM_x000d_ | 0.0 |
| Ng 14-8 1 µM_x000d_ | 0.0 |
| Ng 14-9 1 µM_x000d_ | 0.0 |
| Ng 14-9 3 µM_x000d_ | 0.0 |
| Ng 14-9 10 µM_x000d_ | 0.0 |
| Ng 14-9 30 µM_x000d_ | 0.0 |
| Ng 15-10 1µM_x000d_ | 0.0 |
| Ng 16-9 1 µM_x000d_ | 0.0 |
| Ng 17-8 1 µM_x000d_ | 0.0 |
| Ng 17-8 3 µM_x000d_ | 0.0 |
| Ng 18-9 1µM_x000d_ | 871.3333333333334 |
| Ng 19-11 1 µM_x000d_ | 0.0 |
| Ng 19-11 3 µM_x000d_ | 0.0 |
| Lg 17-15 HF 10 µM_x000d_ | 0.0 |
| Lg 15-17 1 µM_x000d_ | 0.0 |
| Lg 17-15 1 µM_x000d_ | 0.0 |
| Lg 17-15 3 µM_x000d_ | 0.0 |
| Lg 17-15 10 µM_x000d_ | 0.0 |
| Lg 17-17 1 µM_x000d_ | 0.0 |
| Lg 17-16 1 µM_x000d_ | 0.0 |
| Lg 16-17 3 µM_x000d_ | 0.0 |
| Lg 18-8 1 µM_x000d_ | 0.0 |
| Lg 19-8 1 µM_x000d_ | 0.0 |
| Lg 19-8 3 µM_x000d_ | 0.0 |
| Lg 19-9 1 µM_x000d_ | 0.0 |
| Lg 20-10_x000d_ | 0.0 |
| Lg 22-12 1 µM_x000d_ | 0.0 |
| Lg 20-9 1 µM_x000d_ | 0.0 |
| Lg 23-11 1 µM_x000d_ | 0.0 |
| Lg 23-11 3 µM_x000d_ | 0.0 |
| Lg 23-12_x000d_ | 0.0 |
| Lg 26-8_x000d_ | 0.0 |
| Lg 26-9_x000d_ | 0.0 |
| Lg 22-13_x000d_ | 0.0 |
| Lg 26-10_x000d_ | 0.0 |
| Lg 18-7-F3 HF 1uM | 0.0 |
| Lg 19-8-F5 HF 3uM | 0.0 |
| Lg 23-11-F2 HF 3uM | 0.0 |
| Blank 1 | 0.0 |
| Blank 2 | 0.0 |
| Blank 3 | 0.0 |
| Blank 4 | 0.0 |
| Blank 5 | 0.0 |MFI
### Chart: Ov-6
| Category | Ov k-100 IgG4 |
|---|---|
| Ng1_1uM
 | 0.0 |
| Ng1_3uM
 | 0.0 |
| Ng2_1uM | 0.0 |
| Ng2_3uM | 0.0 |
| Ng2_10uM | 0.0 |
| Ng3_1uM | 0.0 |
| Ng3_3uM | 0.0 |
| Ng3_10uM | 0.0 |
| Ng3_30uM | 0.0 |
| Ng4_1uM | 0.0 |
| Ng4_3uM | 0.0 |
| Ng5_1uM | 0.0 |
| Ng5_3uM | 0.0 |
| Ng6_1uM | 0.0 |
| Ng7_1uM | 0.0 |
| Ng7_3uM | 0.0 |
| Ng8_1uM | 0.0 |
| Ng8_3uM | 0.0 |
| Ng8_10uM | 0.0 |
| Ng9_1uM | 0.0 |
| Ng9_3uM | 0.0 |
| Ng10_3uM | 0.0 |
| Ng10_1uM | 0.0 |
| Ng11 | 0.0 |
| Ng12_1uM | 0.0 |
| Ng12_3uM | 0.0 |
| Ng13_1uM | 0.0 |
| Ng14_1uM | 0.0 |
| Ng15_1uM | 631.0 |
| Ng16_1uM | 0.0 |
| Ng17_1uM | 85.33333333333334 |
| Ng18_1uM | 37.66666666666666 |
| Ng18_3uM | 614.0 |
| Ng18_10uM | 1872.0 |
| Ng19_1uM | 79.0 |
| Ng19_3uM | 816.3333333333334 |
| Ng19_10uM | 1020.0 |
| Ng20_1uM | 2.666666666666657 |
| Ng20_3uM | 1066.6666666666667 |
| Ng20_10uM | 2655.0 |
| Ng21_1uM | 0.0 |
| Ng21 _3uM | 150.66666666666669 |
| Ng21_10uM | 1586.3333333333333 |
| Ng22_1uM | 1270.3333333333333 |
| Ng23_1uM | 0.0 |
| Ng24_1uM | 7.0 |
| Ng24_3uM | 277.0 |
| Ng25_1uM | 0.0 |
| Ng25_3uM | 0.0 |
| Ng26_1uM | 52.66666666666666 |
| Ng27_1uM | 0.0 |
| Ng27_3uM | 65.66666666666666 |
| Ng28_1uM | 0.0 |
| Ng29_1uM | 0.0 |
| Ng30_1uM | 0.0 |
| Ng31_1uM | 0.0 |
| Ng31_3uM | 0.0 |
| Ng31_10uM | 0.0 |
| Ng31_30uM | 0.0 |
| Ng32_1uM | 2400.0 |
| Ng33_1uM | 1267.0 |
| Ng34_1uM | 0.0 |
| Ng34_3uM | 180.0 |
| Ng35_1uM | 1094.3333333333333 |
| Ng36_1uM | 15.666666666666657 |
| Ng36_3uM | 199.33333333333331 |
| Lg2_HF_10uM | 1255.0 |
| Lg1_1uM | 0.0 |
| Lg2_1uM | 0.0 |
| Lg2_3uM | 7.666666666666657 |
| Lg2_10uM | 358.6666666666667 |
| Lg3_1uM | 0.0 |
| Lg4_1uM | 1.6666666666666572 |
| Lg5_3uM | 977.3333333333333 |
| Lg6_1uM | 48.33333333333334 |
| Lg7_1uM | 545.6666666666666 |
| Lg7_3uM | 3488.0 |
| Lg8_1uM | 304.6666666666667 |
| Lg9 | 598.6666666666666 |
| Lg10_1uM | 49.0 |
| Lg11_1uM | 4923.666666666667 |
| Lg12_1uM | 2565.0 |
| Lg12_3uM | 5911.0 |
| Lg13 | 1256.3333333333333 |
| Lg14 | 1938.6666666666665 |
| Lg15 | 98.0 |
| Lg16 | 27.666666666666657 |
| Lg17 | 0.0 |
| Lg18_HF_1uM | 1428.0 |
| Lg7_HF_3uM | 5798.0 |
| Lg12_HF_3uM | 1235.3333333333333 |
| Blank 1 | 0.0 |
| Blank 2 | 0.0 |
| Blank 3 | 0.0 |
| Blank 4 | 0.0 |
| Blank 5 | 0.0 |MFI

## Slide 28
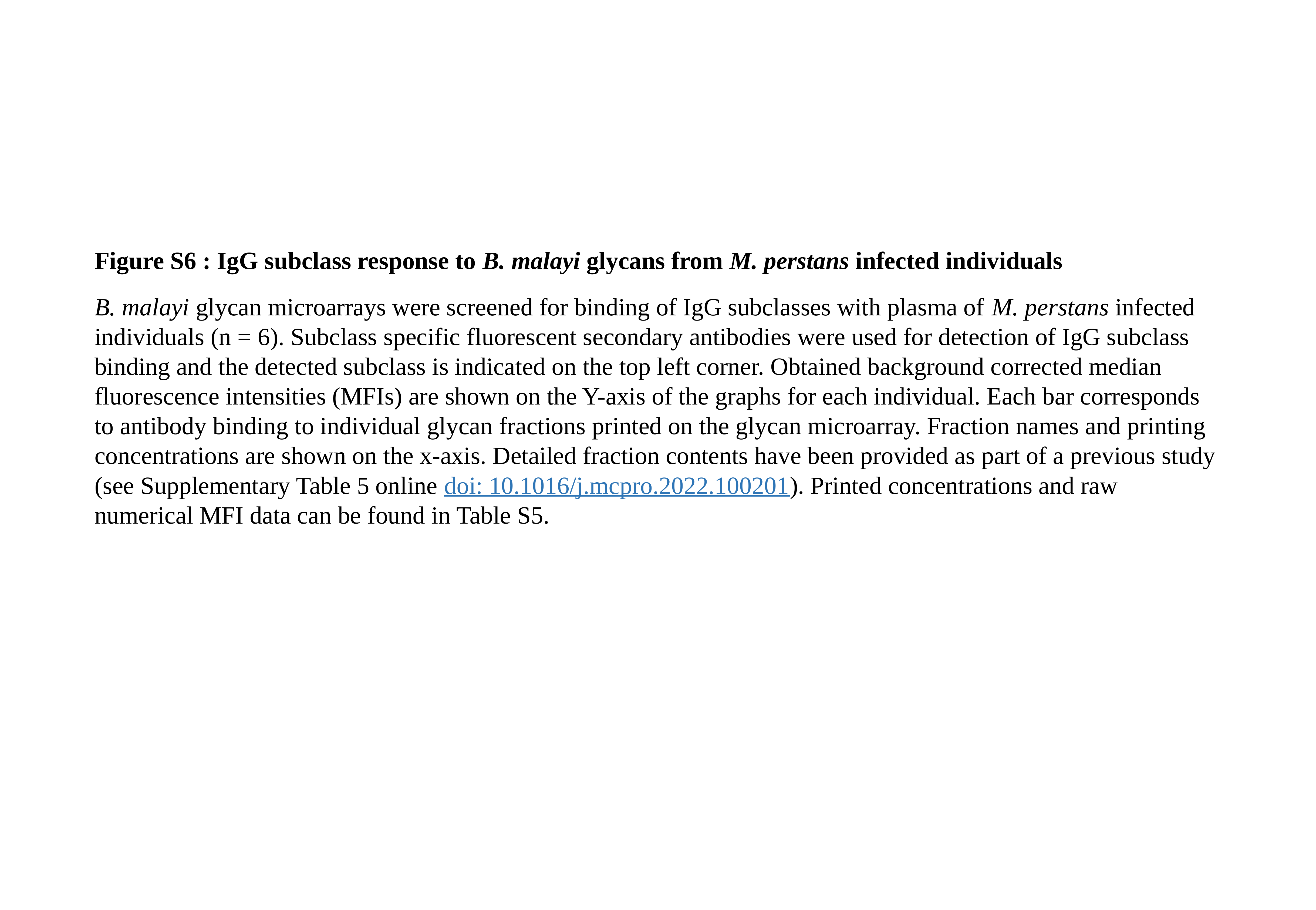

Figure S6 : IgG subclass response to B. malayi glycans from M. perstans infected individuals
B. malayi glycan microarrays were screened for binding of IgG subclasses with plasma of M. perstans infected individuals (n = 6). Subclass specific fluorescent secondary antibodies were used for detection of IgG subclass binding and the detected subclass is indicated on the top left corner. Obtained background corrected median fluorescence intensities (MFIs) are shown on the Y-axis of the graphs for each individual. Each bar corresponds to antibody binding to individual glycan fractions printed on the glycan microarray. Fraction names and printing concentrations are shown on the x-axis. Detailed fraction contents have been provided as part of a previous study (see Supplementary Table 5 online doi: 10.1016/j.mcpro.2022.100201). Printed concentrations and raw numerical MFI data can be found in Table S5.

## Slide 29
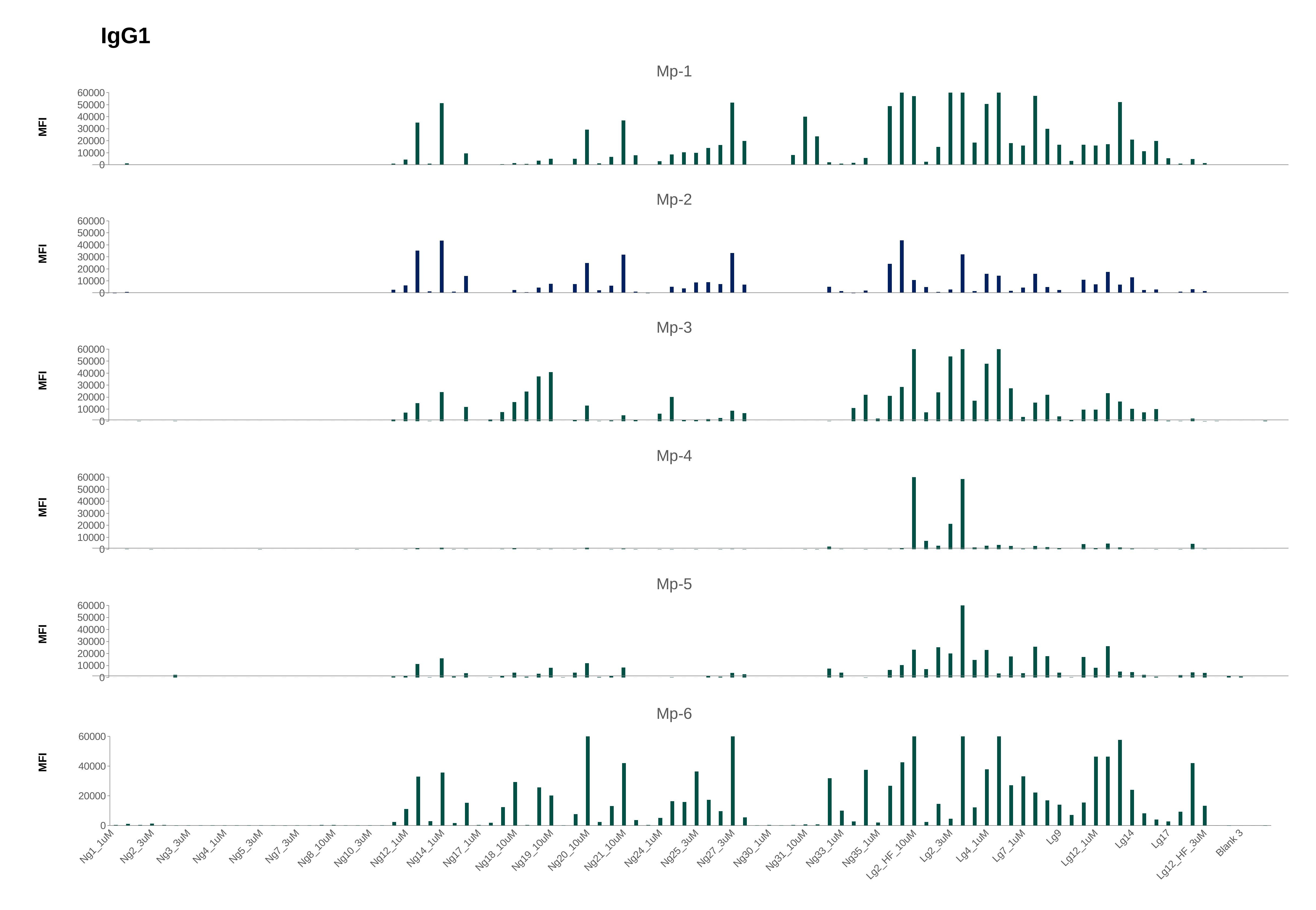

IgG1
### Chart: Mp-1
| Category | Mp 36-51 IgG1 |
|---|---|
| Ng 7-13 1µM_x000d_ | 68.33333333333334 |
| Ng 7-13 3µM_x000d_ | 1074.3333333333333 |
| Ng 9-9 1 µM_x000d_ | 166.33333333333331 |
| Ng 9-9 3µM_x000d_ | 120.33333333333331 |
| Ng 9-9 10µM_x000d_ | 163.33333333333331 |
| Ng 10-13 1 µM_x000d_ | 0.0 |
| Ng 10-13 3 µM_x000d_ | 45.66666666666666 |
| Ng 10-13 10 µM_x000d_ | 6.333333333333343 |
| Ng 10-13 30 µM_x000d_ | 149.0 |
| Ng 10-10 1 µM_x000d_ | 14.666666666666657 |
| Ng 10-10 3 µM_x000d_ | 37.66666666666666 |
| Ng 10-11 1 µM_x000d_ | 72.33333333333334 |
| Ng 10-11 3 µM_x000d_ | 42.66666666666666 |
| Ng 10-12 1 µM_x000d_ | 2.333333333333343 |
| Ng 11-10 1 µM_x000d_ | 78.0 |
| Ng 11-10 3 µM_x000d_ | 7.0 |
| Ng 11-11 1 µM_x000d_ | 0.0 |
| Ng 11-11 3µM_x000d_ | 0.0 |
| Ng 11-11 10µM_x000d_ | 49.0 |
| Ng 12-11 1 µM_x000d_ | 35.33333333333334 |
| Ng 12-11 3 µM_x000d_ | 67.66666666666666 |
| Ng 13-15 1 µM_x000d_ | 33.66666666666666 |
| Ng 13-15 3 µM_x000d_ | 16.666666666666657 |
| Ng 13-14_x000d_ | 1058.3333333333333 |
| Ng 14-10 1 µM_x000d_ | 4374.333333333333 |
| Ng 14-10 3 µM_x000d_ | 35050.666666666664 |
| Ng 14-11 1 µM_x000d_ | 925.0 |
| Ng 14-12 1 µM_x000d_ | 51190.333333333336 |
| Ng 14-7 1 µM_x000d_ | 360.3333333333333 |
| Ng 15-14/15 1 µM_x000d_ | 9484.333333333334 |
| Ng 15-12 1µM_x000d_ | 136.66666666666669 |
| Ng 17-7 1 µM_x000d_ | 89.66666666666666 |
| Ng 17-7 3 µM_x000d_ | 511.66666666666663 |
| Ng 17-7 10 µM_x000d_ | 1381.0 |
| Ng 19-9 1 µM_x000d_ | 765.6666666666666 |
| Ng 19-9 3 µM_x000d_ | 3542.6666666666665 |
| Ng 19-9 10 µM_x000d_ | 4969.333333333333 |
| Ng 20-7 1 µM_x000d_ | 280.6666666666667 |
| Ng 20-7 3 µM_x000d_ | 4993.333333333333 |
| Ng 20-7 10 µM_x000d_ | 29250.666666666668 |
| Ng 21-8 1 µM_x000d_ | 1261.0 |
| Ng 21-8 3 µM_x000d_ | 6623.666666666667 |
| Ng 21-8 10 µM_x000d_ | 36829.666666666664 |
| Ng 15-13 3µM_x000d_ | 7978.0 |
| Ng 16-12 1 µM_x000d_ | 230.66666666666669 |
| Ng 17-9 1 µM_x000d_ | 2954.0 |
| Ng 17-9 3 µM_x000d_ | 8546.333333333334 |
| Ng 18-11 1 µM_x000d_ | 10406.0 |
| Ng 18-11 3 µM_x000d_ | 9862.666666666666 |
| Ng 20-8 1 µM_x000d_ | 14093.333333333334 |
| Ng 20-9 1 µM_x000d_ | 16481.0 |
| Ng 20-9 3 µM_x000d_ | 51653.0 |
| Ng 21-9 1 µM_x000d_ | 19876.666666666668 |
| Ng 13-13 3 µM_x000d_ | 0.0 |
| Ng 14-8 1 µM_x000d_ | 24.0 |
| Ng 14-9 1 µM_x000d_ | 301.6666666666667 |
| Ng 14-9 3 µM_x000d_ | 8177.666666666666 |
| Ng 14-9 10 µM_x000d_ | 40047.666666666664 |
| Ng 14-9 30 µM_x000d_ | 23672.333333333332 |
| Ng 15-10 1µM_x000d_ | 2042.0 |
| Ng 16-9 1 µM_x000d_ | 840.3333333333334 |
| Ng 17-8 1 µM_x000d_ | 1531.0 |
| Ng 17-8 3 µM_x000d_ | 5671.0 |
| Ng 18-9 1µM_x000d_ | 378.33333333333337 |
| Ng 19-11 1 µM_x000d_ | 48876.333333333336 |
| Ng 19-11 3 µM_x000d_ | 63794.666666666664 |
| Lg 17-15 HF 10 µM_x000d_ | 57052.333333333336 |
| Lg 15-17 1 µM_x000d_ | 2634.3333333333335 |
| Lg 17-15 1 µM_x000d_ | 14900.666666666666 |
| Lg 17-15 3 µM_x000d_ | 60564.333333333336 |
| Lg 17-15 10 µM_x000d_ | 64939.0 |
| Lg 17-17 1 µM_x000d_ | 18472.666666666668 |
| Lg 17-16 1 µM_x000d_ | 50619.0 |
| Lg 16-17 3 µM_x000d_ | 64880.666666666664 |
| Lg 18-8 1 µM_x000d_ | 18008.0 |
| Lg 19-8 1 µM_x000d_ | 15968.0 |
| Lg 19-8 3 µM_x000d_ | 57265.666666666664 |
| Lg 19-9 1 µM_x000d_ | 29871.666666666668 |
| Lg 20-10_x000d_ | 16609.333333333332 |
| Lg 22-12 1 µM_x000d_ | 3189.3333333333335 |
| Lg 20-9 1 µM_x000d_ | 16688.666666666668 |
| Lg 23-11 1 µM_x000d_ | 16102.666666666666 |
| Lg 23-11 3 µM_x000d_ | 17175.666666666668 |
| Lg 23-12_x000d_ | 52232.0 |
| Lg 26-8_x000d_ | 20976.666666666668 |
| Lg 26-9_x000d_ | 11207.333333333334 |
| Lg 22-13_x000d_ | 19723.0 |
| Lg 26-10_x000d_ | 5539.333333333333 |
| Lg 18-7-F3 HF 1uM | 958.6666666666667 |
| Lg 19-8-F5 HF 3uM | 4704.0 |
| Lg 23-11-F2 HF 3uM | 1314.0 |
| Blank 1 | 0.0 |
| Blank 2 | 35.33333333333334 |
| Blank 3 | 0.0 |
| Blank 4 | 0.0 |
| Blank 5 | 0.0 |MFI
### Chart: Mp-2
| Category | Mp 38-202 IgG1 |
|---|---|
| Ng 7-13 1µM_x000d_ | 29.333333333333314 |
| Ng 7-13 3µM_x000d_ | 927.3333333333335 |
| Ng 9-9 1 µM_x000d_ | 0.0 |
| Ng 9-9 3µM_x000d_ | 0.0 |
| Ng 9-9 10µM_x000d_ | 0.0 |
| Ng 10-13 1 µM_x000d_ | 0.0 |
| Ng 10-13 3 µM_x000d_ | 0.0 |
| Ng 10-13 10 µM_x000d_ | 0.0 |
| Ng 10-13 30 µM_x000d_ | 0.0 |
| Ng 10-10 1 µM_x000d_ | 0.0 |
| Ng 10-10 3 µM_x000d_ | 0.0 |
| Ng 10-11 1 µM_x000d_ | 0.0 |
| Ng 10-11 3 µM_x000d_ | 0.0 |
| Ng 10-12 1 µM_x000d_ | 0.0 |
| Ng 11-10 1 µM_x000d_ | 0.0 |
| Ng 11-10 3 µM_x000d_ | 0.0 |
| Ng 11-11 1 µM_x000d_ | 0.0 |
| Ng 11-11 3µM_x000d_ | 451.00000000000006 |
| Ng 11-11 10µM_x000d_ | 256.00000000000006 |
| Ng 12-11 1 µM_x000d_ | 0.0 |
| Ng 12-11 3 µM_x000d_ | 0.0 |
| Ng 13-15 1 µM_x000d_ | 0.0 |
| Ng 13-15 3 µM_x000d_ | 0.0 |
| Ng 13-14_x000d_ | 2593.333333333333 |
| Ng 14-10 1 µM_x000d_ | 6250.0 |
| Ng 14-10 3 µM_x000d_ | 35302.0 |
| Ng 14-11 1 µM_x000d_ | 1441.3333333333335 |
| Ng 14-12 1 µM_x000d_ | 43690.33333333333 |
| Ng 14-7 1 µM_x000d_ | 1004.6666666666667 |
| Ng 15-14/15 1 µM_x000d_ | 14207.333333333332 |
| Ng 15-12 1µM_x000d_ | 0.0 |
| Ng 17-7 1 µM_x000d_ | 0.0 |
| Ng 17-7 3 µM_x000d_ | 0.0 |
| Ng 17-7 10 µM_x000d_ | 2451.0 |
| Ng 19-9 1 µM_x000d_ | 693.0 |
| Ng 19-9 3 µM_x000d_ | 4522.666666666667 |
| Ng 19-9 10 µM_x000d_ | 7538.666666666667 |
| Ng 20-7 1 µM_x000d_ | 354.00000000000006 |
| Ng 20-7 3 µM_x000d_ | 7292.0 |
| Ng 20-7 10 µM_x000d_ | 24906.333333333336 |
| Ng 21-8 1 µM_x000d_ | 2340.333333333333 |
| Ng 21-8 3 µM_x000d_ | 5976.0 |
| Ng 21-8 10 µM_x000d_ | 31899.333333333336 |
| Ng 15-13 3µM_x000d_ | 1042.0 |
| Ng 16-12 1 µM_x000d_ | 43.333333333333314 |
| Ng 17-9 1 µM_x000d_ | 0.0 |
| Ng 17-9 3 µM_x000d_ | 5128.333333333334 |
| Ng 18-11 1 µM_x000d_ | 3872.3333333333335 |
| Ng 18-11 3 µM_x000d_ | 8830.0 |
| Ng 20-8 1 µM_x000d_ | 9043.333333333332 |
| Ng 20-9 1 µM_x000d_ | 7499.666666666667 |
| Ng 20-9 3 µM_x000d_ | 33279.0 |
| Ng 21-9 1 µM_x000d_ | 6881.333333333334 |
| Ng 13-13 3 µM_x000d_ | 0.0 |
| Ng 14-8 1 µM_x000d_ | 0.0 |
| Ng 14-9 1 µM_x000d_ | 0.0 |
| Ng 14-9 3 µM_x000d_ | 0.0 |
| Ng 14-9 10 µM_x000d_ | 0.0 |
| Ng 14-9 30 µM_x000d_ | 0.0 |
| Ng 15-10 1µM_x000d_ | 5202.666666666667 |
| Ng 16-9 1 µM_x000d_ | 1504.3333333333335 |
| Ng 17-8 1 µM_x000d_ | 71.33333333333331 |
| Ng 17-8 3 µM_x000d_ | 2050.333333333333 |
| Ng 18-9 1µM_x000d_ | 159.66666666666669 |
| Ng 19-11 1 µM_x000d_ | 24298.333333333336 |
| Ng 19-11 3 µM_x000d_ | 43757.0 |
| Lg 17-15 HF 10 µM_x000d_ | 10726.666666666666 |
| Lg 15-17 1 µM_x000d_ | 4836.666666666667 |
| Lg 17-15 1 µM_x000d_ | 940.3333333333335 |
| Lg 17-15 3 µM_x000d_ | 2943.6666666666665 |
| Lg 17-15 10 µM_x000d_ | 32168.666666666668 |
| Lg 17-17 1 µM_x000d_ | 1504.0 |
| Lg 17-16 1 µM_x000d_ | 15957.999999999998 |
| Lg 16-17 3 µM_x000d_ | 14397.666666666666 |
| Lg 18-8 1 µM_x000d_ | 1722.3333333333333 |
| Lg 19-8 1 µM_x000d_ | 4514.0 |
| Lg 19-8 3 µM_x000d_ | 15836.666666666666 |
| Lg 19-9 1 µM_x000d_ | 5025.333333333334 |
| Lg 20-10_x000d_ | 2412.0 |
| Lg 22-12 1 µM_x000d_ | 94.00000000000006 |
| Lg 20-9 1 µM_x000d_ | 11077.666666666666 |
| Lg 23-11 1 µM_x000d_ | 7113.333333333334 |
| Lg 23-11 3 µM_x000d_ | 17401.666666666668 |
| Lg 23-12_x000d_ | 7036.0 |
| Lg 26-8_x000d_ | 13059.0 |
| Lg 26-9_x000d_ | 2539.333333333333 |
| Lg 22-13_x000d_ | 2804.333333333333 |
| Lg 26-10_x000d_ | 0.0 |
| Lg 18-7-F3 HF 1uM | 1204.6666666666667 |
| Lg 19-8-F5 HF 3uM | 3158.333333333333 |
| Lg 23-11-F2 HF 3uM | 1534.6666666666667 |
| Blank 1 | 0.0 |
| Blank 2 | 0.0 |
| Blank 3 | 0.0 |
| Blank 4 | 0.0 |
| Blank 5 | 0.0 |MFI
### Chart: Mp-3
| Category | Mp39-64 IgG1 |
|---|---|
| Ng 7-13 1µM_x000d_ | 0.0 |
| Ng 7-13 3µM_x000d_ | 0.0 |
| Ng 9-9 1 µM_x000d_ | 76.99999999999994 |
| Ng 9-9 3µM_x000d_ | 0.0 |
| Ng 9-9 10µM_x000d_ | 0.0 |
| Ng 10-13 1 µM_x000d_ | 179.99999999999994 |
| Ng 10-13 3 µM_x000d_ | 0.0 |
| Ng 10-13 10 µM_x000d_ | 0.0 |
| Ng 10-13 30 µM_x000d_ | 0.0 |
| Ng 10-10 1 µM_x000d_ | 0.0 |
| Ng 10-10 3 µM_x000d_ | 0.0 |
| Ng 10-11 1 µM_x000d_ | 0.0 |
| Ng 10-11 3 µM_x000d_ | 0.0 |
| Ng 10-12 1 µM_x000d_ | 0.0 |
| Ng 11-10 1 µM_x000d_ | 0.0 |
| Ng 11-10 3 µM_x000d_ | 0.0 |
| Ng 11-11 1 µM_x000d_ | 0.0 |
| Ng 11-11 3µM_x000d_ | 0.0 |
| Ng 11-11 10µM_x000d_ | 0.0 |
| Ng 12-11 1 µM_x000d_ | 0.0 |
| Ng 12-11 3 µM_x000d_ | 0.0 |
| Ng 13-15 1 µM_x000d_ | 0.0 |
| Ng 13-15 3 µM_x000d_ | 0.0 |
| Ng 13-14_x000d_ | 1558.3333333333333 |
| Ng 14-10 1 µM_x000d_ | 7216.333333333333 |
| Ng 14-10 3 µM_x000d_ | 15041.666666666668 |
| Ng 14-11 1 µM_x000d_ | 232.33333333333331 |
| Ng 14-12 1 µM_x000d_ | 24279.0 |
| Ng 14-7 1 µM_x000d_ | 0.0 |
| Ng 15-14/15 1 µM_x000d_ | 11904.666666666668 |
| Ng 15-12 1µM_x000d_ | 0.0 |
| Ng 17-7 1 µM_x000d_ | 1468.6666666666665 |
| Ng 17-7 3 µM_x000d_ | 7490.666666666666 |
| Ng 17-7 10 µM_x000d_ | 15781.0 |
| Ng 19-9 1 µM_x000d_ | 24693.666666666664 |
| Ng 19-9 3 µM_x000d_ | 37181.333333333336 |
| Ng 19-9 10 µM_x000d_ | 40745.333333333336 |
| Ng 20-7 1 µM_x000d_ | 0.0 |
| Ng 20-7 3 µM_x000d_ | 811.3333333333333 |
| Ng 20-7 10 µM_x000d_ | 12861.666666666668 |
| Ng 21-8 1 µM_x000d_ | 103.66666666666669 |
| Ng 21-8 3 µM_x000d_ | 686.0 |
| Ng 21-8 10 µM_x000d_ | 4872.0 |
| Ng 15-13 3µM_x000d_ | 1332.6666666666665 |
| Ng 16-12 1 µM_x000d_ | 0.0 |
| Ng 17-9 1 µM_x000d_ | 6210.666666666666 |
| Ng 17-9 3 µM_x000d_ | 20190.666666666664 |
| Ng 18-11 1 µM_x000d_ | 1045.0 |
| Ng 18-11 3 µM_x000d_ | 964.3333333333333 |
| Ng 20-8 1 µM_x000d_ | 1666.6666666666667 |
| Ng 20-9 1 µM_x000d_ | 2607.0 |
| Ng 20-9 3 µM_x000d_ | 8779.666666666668 |
| Ng 21-9 1 µM_x000d_ | 6779.0 |
| Ng 13-13 3 µM_x000d_ | 0.0 |
| Ng 14-8 1 µM_x000d_ | 0.0 |
| Ng 14-9 1 µM_x000d_ | 0.0 |
| Ng 14-9 3 µM_x000d_ | 0.0 |
| Ng 14-9 10 µM_x000d_ | 0.0 |
| Ng 14-9 30 µM_x000d_ | 0.0 |
| Ng 15-10 1µM_x000d_ | 39.99999999999994 |
| Ng 16-9 1 µM_x000d_ | 0.0 |
| Ng 17-8 1 µM_x000d_ | 10941.666666666668 |
| Ng 17-8 3 µM_x000d_ | 21943.0 |
| Ng 18-9 1µM_x000d_ | 2247.3333333333335 |
| Ng 19-11 1 µM_x000d_ | 21011.0 |
| Ng 19-11 3 µM_x000d_ | 28523.666666666664 |
| Lg 17-15 HF 10 µM_x000d_ | 62633.333333333336 |
| Lg 15-17 1 µM_x000d_ | 7357.666666666666 |
| Lg 17-15 1 µM_x000d_ | 23982.666666666664 |
| Lg 17-15 3 µM_x000d_ | 53791.0 |
| Lg 17-15 10 µM_x000d_ | 64296.333333333336 |
| Lg 17-17 1 µM_x000d_ | 17045.0 |
| Lg 17-16 1 µM_x000d_ | 47891.0 |
| Lg 16-17 3 µM_x000d_ | 63701.333333333336 |
| Lg 18-8 1 µM_x000d_ | 27403.333333333332 |
| Lg 19-8 1 µM_x000d_ | 3585.0 |
| Lg 19-8 3 µM_x000d_ | 15344.333333333334 |
| Lg 19-9 1 µM_x000d_ | 21967.666666666664 |
| Lg 20-10_x000d_ | 3944.0000000000005 |
| Lg 22-12 1 µM_x000d_ | 1241.6666666666665 |
| Lg 20-9 1 µM_x000d_ | 9671.333333333334 |
| Lg 23-11 1 µM_x000d_ | 9669.0 |
| Lg 23-11 3 µM_x000d_ | 23397.333333333332 |
| Lg 23-12_x000d_ | 16267.666666666666 |
| Lg 26-8_x000d_ | 10166.0 |
| Lg 26-9_x000d_ | 7240.666666666666 |
| Lg 22-13_x000d_ | 10057.0 |
| Lg 26-10_x000d_ | 421.3333333333333 |
| Lg 18-7-F3 HF 1uM | 123.66666666666669 |
| Lg 19-8-F5 HF 3uM | 2247.0 |
| Lg 23-11-F2 HF 3uM | 30.999999999999943 |
| Blank 1 | 247.66666666666669 |
| Blank 2 | 0.0 |
| Blank 3 | 0.0 |
| Blank 4 | 0.0 |
| Blank 5 | 396.3333333333333 |MFI
### Chart: Mp-4
| Category | Mp 39-115 IgG1 |
|---|---|
| Ng 7-13 1µM_x000d_ | 0.0 |
| Ng 7-13 3µM_x000d_ | 250.5 |
| Ng 9-9 1 µM_x000d_ | 0.0 |
| Ng 9-9 3µM_x000d_ | 33.0 |
| Ng 9-9 10µM_x000d_ | 0.0 |
| Ng 10-13 1 µM_x000d_ | 0.0 |
| Ng 10-13 3 µM_x000d_ | 0.0 |
| Ng 10-13 10 µM_x000d_ | 0.0 |
| Ng 10-13 30 µM_x000d_ | 0.0 |
| Ng 10-10 1 µM_x000d_ | 0.0 |
| Ng 10-10 3 µM_x000d_ | 0.0 |
| Ng 10-11 1 µM_x000d_ | 0.0 |
| Ng 10-11 3 µM_x000d_ | 15.333333333333314 |
| Ng 10-12 1 µM_x000d_ | 0.0 |
| Ng 11-10 1 µM_x000d_ | 0.0 |
| Ng 11-10 3 µM_x000d_ | 0.0 |
| Ng 11-11 1 µM_x000d_ | 0.0 |
| Ng 11-11 3µM_x000d_ | 0.0 |
| Ng 11-11 10µM_x000d_ | 0.0 |
| Ng 12-11 1 µM_x000d_ | 0.0 |
| Ng 12-11 3 µM_x000d_ | 134.33333333333331 |
| Ng 13-15 1 µM_x000d_ | 0.0 |
| Ng 13-15 3 µM_x000d_ | 0.0 |
| Ng 13-14_x000d_ | 0.0 |
| Ng 14-10 1 µM_x000d_ | 42.333333333333314 |
| Ng 14-10 3 µM_x000d_ | 1018.6666666666667 |
| Ng 14-11 1 µM_x000d_ | 0.0 |
| Ng 14-12 1 µM_x000d_ | 1351.6666666666667 |
| Ng 14-7 1 µM_x000d_ | 14.0 |
| Ng 15-14/15 1 µM_x000d_ | 315.0 |
| Ng 15-12 1µM_x000d_ | 0.0 |
| Ng 17-7 1 µM_x000d_ | 0.0 |
| Ng 17-7 3 µM_x000d_ | 206.0 |
| Ng 17-7 10 µM_x000d_ | 800.6666666666667 |
| Ng 19-9 1 µM_x000d_ | 0.0 |
| Ng 19-9 3 µM_x000d_ | 196.33333333333331 |
| Ng 19-9 10 µM_x000d_ | 389.66666666666663 |
| Ng 20-7 1 µM_x000d_ | 0.0 |
| Ng 20-7 3 µM_x000d_ | 163.33333333333331 |
| Ng 20-7 10 µM_x000d_ | 1402.3333333333333 |
| Ng 21-8 1 µM_x000d_ | 0.0 |
| Ng 21-8 3 µM_x000d_ | 12.666666666666686 |
| Ng 21-8 10 µM_x000d_ | 632.3333333333334 |
| Ng 15-13 3µM_x000d_ | 162.33333333333331 |
| Ng 16-12 1 µM_x000d_ | 0.0 |
| Ng 17-9 1 µM_x000d_ | 26.333333333333314 |
| Ng 17-9 3 µM_x000d_ | 106.0 |
| Ng 18-11 1 µM_x000d_ | 0.0 |
| Ng 18-11 3 µM_x000d_ | 84.33333333333331 |
| Ng 20-8 1 µM_x000d_ | 0.0 |
| Ng 20-9 1 µM_x000d_ | 1.6666666666666856 |
| Ng 20-9 3 µM_x000d_ | 342.66666666666663 |
| Ng 21-9 1 µM_x000d_ | 156.66666666666669 |
| Ng 13-13 3 µM_x000d_ | 0.0 |
| Ng 14-8 1 µM_x000d_ | 0.0 |
| Ng 14-9 1 µM_x000d_ | 0.0 |
| Ng 14-9 3 µM_x000d_ | 0.0 |
| Ng 14-9 10 µM_x000d_ | 19.333333333333314 |
| Ng 14-9 30 µM_x000d_ | 88.66666666666669 |
| Ng 15-10 1µM_x000d_ | 2381.6666666666665 |
| Ng 16-9 1 µM_x000d_ | 365.33333333333337 |
| Ng 17-8 1 µM_x000d_ | 0.0 |
| Ng 17-8 3 µM_x000d_ | 15.666666666666686 |
| Ng 18-9 1µM_x000d_ | 0.0 |
| Ng 19-11 1 µM_x000d_ | 380.0 |
| Ng 19-11 3 µM_x000d_ | 850.3333333333333 |
| Lg 17-15 HF 10 µM_x000d_ | 63033.0 |
| Lg 15-17 1 µM_x000d_ | 7122.666666666667 |
| Lg 17-15 1 µM_x000d_ | 2898.3333333333335 |
| Lg 17-15 3 µM_x000d_ | 21247.333333333332 |
| Lg 17-15 10 µM_x000d_ | 58476.333333333336 |
| Lg 17-17 1 µM_x000d_ | 1710.6666666666667 |
| Lg 17-16 1 µM_x000d_ | 3009.0 |
| Lg 16-17 3 µM_x000d_ | 3658.0 |
| Lg 18-8 1 µM_x000d_ | 2730.3333333333335 |
| Lg 19-8 1 µM_x000d_ | 428.0 |
| Lg 19-8 3 µM_x000d_ | 2846.3333333333335 |
| Lg 19-9 1 µM_x000d_ | 1771.6666666666665 |
| Lg 20-10_x000d_ | 666.3333333333334 |
| Lg 22-12 1 µM_x000d_ | 0.0 |
| Lg 20-9 1 µM_x000d_ | 4400.666666666667 |
| Lg 23-11 1 µM_x000d_ | 681.0 |
| Lg 23-11 3 µM_x000d_ | 4827.0 |
| Lg 23-12_x000d_ | 1702.3333333333333 |
| Lg 26-8_x000d_ | 502.66666666666663 |
| Lg 26-9_x000d_ | 0.0 |
| Lg 22-13_x000d_ | 95.0 |
| Lg 26-10_x000d_ | 0.0 |
| Lg 18-7-F3 HF 1uM | 135.66666666666669 |
| Lg 19-8-F5 HF 3uM | 4613.333333333333 |
| Lg 23-11-F2 HF 3uM | 235.33333333333337 |
| Blank 1 | 0.0 |
| Blank 2 | 0.0 |
| Blank 3 | 0.0 |
| Blank 4 | 0.0 |
| Blank 5 | 0.0 |MFI
### Chart: Mp-5
| Category | Mp k-26 IgG1 |
|---|---|
| Ng 7-13 1µM_x000d_ | 0.0 |
| Ng 7-13 3µM_x000d_ | 0.0 |
| Ng 9-9 1 µM_x000d_ | 0.0 |
| Ng 9-9 3µM_x000d_ | 0.0 |
| Ng 9-9 10µM_x000d_ | 0.0 |
| Ng 10-13 1 µM_x000d_ | 2205.0 |
| Ng 10-13 3 µM_x000d_ | 0.0 |
| Ng 10-13 10 µM_x000d_ | 0.0 |
| Ng 10-13 30 µM_x000d_ | 0.0 |
| Ng 10-10 1 µM_x000d_ | 0.0 |
| Ng 10-10 3 µM_x000d_ | 0.0 |
| Ng 10-11 1 µM_x000d_ | 0.0 |
| Ng 10-11 3 µM_x000d_ | 0.0 |
| Ng 10-12 1 µM_x000d_ | 0.0 |
| Ng 11-10 1 µM_x000d_ | 0.0 |
| Ng 11-10 3 µM_x000d_ | 0.0 |
| Ng 11-11 1 µM_x000d_ | 0.0 |
| Ng 11-11 3µM_x000d_ | 0.0 |
| Ng 11-11 10µM_x000d_ | 0.0 |
| Ng 12-11 1 µM_x000d_ | 0.0 |
| Ng 12-11 3 µM_x000d_ | 0.0 |
| Ng 13-15 1 µM_x000d_ | 0.0 |
| Ng 13-15 3 µM_x000d_ | 0.0 |
| Ng 13-14_x000d_ | 836.0 |
| Ng 14-10 1 µM_x000d_ | 1638.6666666666665 |
| Ng 14-10 3 µM_x000d_ | 11318.666666666666 |
| Ng 14-11 1 µM_x000d_ | 224.33333333333337 |
| Ng 14-12 1 µM_x000d_ | 15999.333333333332 |
| Ng 14-7 1 µM_x000d_ | 845.6666666666667 |
| Ng 15-14/15 1 µM_x000d_ | 3623.666666666667 |
| Ng 15-12 1µM_x000d_ | 0.0 |
| Ng 17-7 1 µM_x000d_ | 273.0 |
| Ng 17-7 3 µM_x000d_ | 1102.6666666666667 |
| Ng 17-7 10 µM_x000d_ | 4152.333333333333 |
| Ng 19-9 1 µM_x000d_ | 579.6666666666667 |
| Ng 19-9 3 µM_x000d_ | 3256.0 |
| Ng 19-9 10 µM_x000d_ | 8024.333333333334 |
| Ng 20-7 1 µM_x000d_ | 148.0 |
| Ng 20-7 3 µM_x000d_ | 3982.0 |
| Ng 20-7 10 µM_x000d_ | 11817.666666666666 |
| Ng 21-8 1 µM_x000d_ | 451.66666666666674 |
| Ng 21-8 3 µM_x000d_ | 1097.6666666666667 |
| Ng 21-8 10 µM_x000d_ | 8271.666666666666 |
| Ng 15-13 3µM_x000d_ | 0.0 |
| Ng 16-12 1 µM_x000d_ | 0.0 |
| Ng 17-9 1 µM_x000d_ | 0.0 |
| Ng 17-9 3 µM_x000d_ | 204.66666666666663 |
| Ng 18-11 1 µM_x000d_ | 0.0 |
| Ng 18-11 3 µM_x000d_ | 0.0 |
| Ng 20-8 1 µM_x000d_ | 1421.6666666666665 |
| Ng 20-9 1 µM_x000d_ | 722.0 |
| Ng 20-9 3 µM_x000d_ | 3734.0 |
| Ng 21-9 1 µM_x000d_ | 2604.6666666666665 |
| Ng 13-13 3 µM_x000d_ | 0.0 |
| Ng 14-8 1 µM_x000d_ | 0.0 |
| Ng 14-9 1 µM_x000d_ | 0.0 |
| Ng 14-9 3 µM_x000d_ | 0.0 |
| Ng 14-9 10 µM_x000d_ | 0.0 |
| Ng 14-9 30 µM_x000d_ | 0.0 |
| Ng 15-10 1µM_x000d_ | 7500.666666666667 |
| Ng 16-9 1 µM_x000d_ | 4154.333333333333 |
| Ng 17-8 1 µM_x000d_ | 0.0 |
| Ng 17-8 3 µM_x000d_ | 110.33333333333337 |
| Ng 18-9 1µM_x000d_ | 0.0 |
| Ng 19-11 1 µM_x000d_ | 6384.333333333333 |
| Ng 19-11 3 µM_x000d_ | 10308.333333333334 |
| Lg 17-15 HF 10 µM_x000d_ | 23249.0 |
| Lg 15-17 1 µM_x000d_ | 7064.0 |
| Lg 17-15 1 µM_x000d_ | 25241.0 |
| Lg 17-15 3 µM_x000d_ | 20020.666666666668 |
| Lg 17-15 10 µM_x000d_ | 60913.0 |
| Lg 17-17 1 µM_x000d_ | 14719.0 |
| Lg 17-16 1 µM_x000d_ | 22956.0 |
| Lg 16-17 3 µM_x000d_ | 3268.6666666666665 |
| Lg 18-8 1 µM_x000d_ | 17530.0 |
| Lg 19-8 1 µM_x000d_ | 3644.333333333333 |
| Lg 19-8 3 µM_x000d_ | 25556.333333333332 |
| Lg 19-9 1 µM_x000d_ | 17648.666666666668 |
| Lg 20-10_x000d_ | 4138.666666666667 |
| Lg 22-12 1 µM_x000d_ | 334.66666666666663 |
| Lg 20-9 1 µM_x000d_ | 16994.666666666668 |
| Lg 23-11 1 µM_x000d_ | 8065.666666666666 |
| Lg 23-11 3 µM_x000d_ | 26054.0 |
| Lg 23-12_x000d_ | 5027.333333333333 |
| Lg 26-8_x000d_ | 4411.0 |
| Lg 26-9_x000d_ | 2166.0 |
| Lg 22-13_x000d_ | 632.0 |
| Lg 26-10_x000d_ | 0.0 |
| Lg 18-7-F3 HF 1uM | 1969.0 |
| Lg 19-8-F5 HF 3uM | 4179.666666666667 |
| Lg 23-11-F2 HF 3uM | 3792.333333333333 |
| Blank 1 | 0.0 |
| Blank 2 | 1219.3333333333333 |
| Blank 3 | 823.3333333333333 |
| Blank 4 | 0.0 |
| Blank 5 | 0.0 |MFI
### Chart: Mp-6
| Category | Mp L23 IgG1 |
|---|---|
| Ng1_1uM
 | 353.33333333333337 |
| Ng1_3uM
 | 1129.0 |
| Ng2_1uM | 423.66666666666674 |
| Ng2_3uM | 1219.6666666666665 |
| Ng2_10uM | 364.0 |
| Ng3_1uM | 75.66666666666669 |
| Ng3_3uM | 225.00000000000003 |
| Ng3_10uM | 136.33333333333334 |
| Ng3_30uM | 142.66666666666666 |
| Ng4_1uM | 222.66666666666666 |
| Ng4_3uM | 156.66666666666666 |
| Ng5_1uM | 228.00000000000003 |
| Ng5_3uM | 178.66666666666666 |
| Ng6_1uM | 131.00000000000003 |
| Ng7_1uM | 111.33333333333334 |
| Ng7_3uM | 162.00000000000003 |
| Ng8_1uM | 153.66666666666666 |
| Ng8_3uM | 357.66666666666674 |
| Ng8_10uM | 332.0 |
| Ng9_1uM | 227.66666666666666 |
| Ng9_3uM | 195.00000000000003 |
| Ng10_3uM | 178.33333333333334 |
| Ng10_1uM | 195.00000000000003 |
| Ng11 | 2475.0 |
| Ng12_1uM | 11204.666666666668 |
| Ng12_3uM | 33000.66666666667 |
| Ng13_1uM | 2928.3333333333335 |
| Ng14_1uM | 35765.0 |
| Ng15_1uM | 1613.6666666666665 |
| Ng16_1uM | 15375.0 |
| Ng17_1uM | 315.0 |
| Ng18_1uM | 1794.3333333333333 |
| Ng18_3uM | 12454.0 |
| Ng18_10uM | 29340.0 |
| Ng19_1uM | 455.0 |
| Ng19_3uM | 25769.0 |
| Ng19_10uM | 20125.666666666664 |
| Ng20_1uM | 237.00000000000003 |
| Ng20_3uM | 7626.333333333333 |
| Ng20_10uM | 64928.0 |
| Ng21_1uM | 2428.666666666667 |
| Ng21 _3uM | 13092.0 |
| Ng21_10uM | 42072.66666666667 |
| Ng22_1uM | 3602.666666666667 |
| Ng23_1uM | 371.66666666666674 |
| Ng24_1uM | 5062.666666666666 |
| Ng24_3uM | 16403.666666666664 |
| Ng25_1uM | 15770.666666666668 |
| Ng25_3uM | 36437.333333333336 |
| Ng26_1uM | 17301.666666666664 |
| Ng27_1uM | 9736.666666666668 |
| Ng27_3uM | 62328.0 |
| Ng28_1uM | 5425.333333333333 |
| Ng29_1uM | 226.00000000000003 |
| Ng30_1uM | 303.33333333333337 |
| Ng31_1uM | 142.66666666666666 |
| Ng31_3uM | 307.33333333333337 |
| Ng31_10uM | 815.0 |
| Ng31_30uM | 790.0 |
| Ng32_1uM | 31863.666666666664 |
| Ng33_1uM | 10034.0 |
| Ng34_1uM | 2808.3333333333335 |
| Ng34_3uM | 37533.333333333336 |
| Ng35_1uM | 1983.3333333333333 |
| Ng36_1uM | 26715.333333333332 |
| Ng36_3uM | 42649.333333333336 |
| Lg2_HF_10uM | 63664.0 |
| Lg1_1uM | 2409.3333333333335 |
| Lg2_1uM | 14495.333333333334 |
| Lg2_3uM | 4602.0 |
| Lg2_10uM | 64926.66666666667 |
| Lg3_1uM | 12165.666666666668 |
| Lg4_1uM | 37885.333333333336 |
| Lg5_3uM | 61384.0 |
| Lg6_1uM | 27063.333333333332 |
| Lg7_1uM | 33159.0 |
| Lg7_3uM | 22284.666666666664 |
| Lg8_1uM | 16983.333333333332 |
| Lg9 | 14051.333333333334 |
| Lg10_1uM | 7205.333333333333 |
| Lg11_1uM | 15428.333333333334 |
| Lg12_1uM | 46451.333333333336 |
| Lg12_3uM | 46470.333333333336 |
| Lg13 | 57656.0 |
| Lg14 | 23956.333333333332 |
| Lg15 | 8217.333333333334 |
| Lg16 | 4111.333333333333 |
| Lg17 | 2763.0 |
| Lg18_HF_1uM | 9300.0 |
| Lg7_HF_3uM | 41965.0 |
| Lg12_HF_3uM | 13388.333333333334 |
| Blank 1 | 0.0 |
| Blank 2 | 24.666666666666686 |
| Blank 3 | 0.0 |
| Blank 4 | 0.0 |
| Blank 5 | 26.0 |MFI

## Slide 30
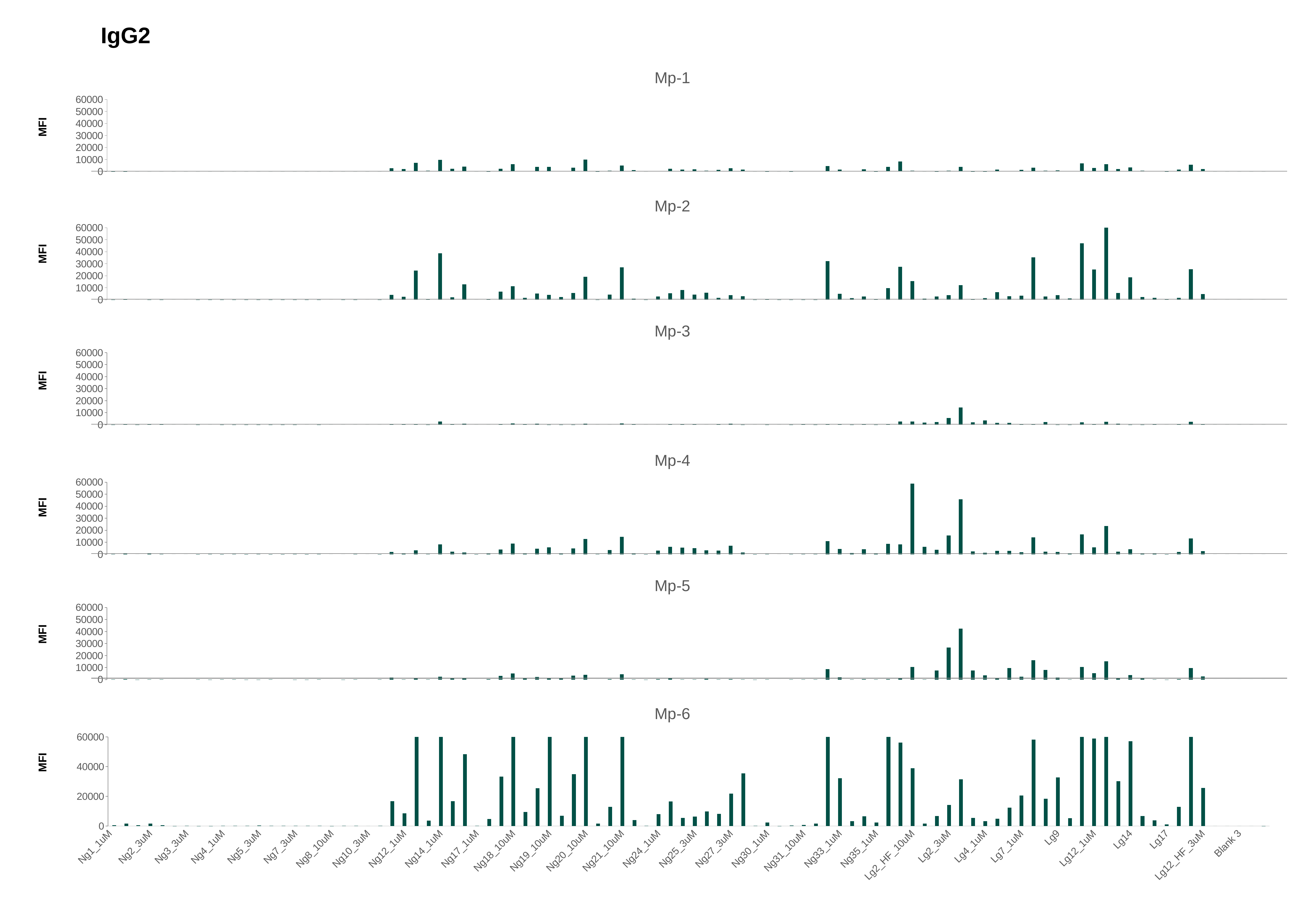

IgG2
### Chart: Mp-1
| Category | Mp 36-51 IgG2 |
|---|---|
| Ng 7-13 1µM_x000d_ | 0.6666666666666572 |
| Ng 7-13 3µM_x000d_ | 151.66666666666669 |
| Ng 9-9 1 µM_x000d_ | 0.0 |
| Ng 9-9 3µM_x000d_ | 0.0 |
| Ng 9-9 10µM_x000d_ | 0.0 |
| Ng 10-13 1 µM_x000d_ | 0.0 |
| Ng 10-13 3 µM_x000d_ | 0.0 |
| Ng 10-13 10 µM_x000d_ | 0.0 |
| Ng 10-13 30 µM_x000d_ | 0.0 |
| Ng 10-10 1 µM_x000d_ | 0.0 |
| Ng 10-10 3 µM_x000d_ | 0.0 |
| Ng 10-11 1 µM_x000d_ | 0.0 |
| Ng 10-11 3 µM_x000d_ | 0.0 |
| Ng 10-12 1 µM_x000d_ | 0.0 |
| Ng 11-10 1 µM_x000d_ | 0.0 |
| Ng 11-10 3 µM_x000d_ | 0.0 |
| Ng 11-11 1 µM_x000d_ | 0.0 |
| Ng 11-11 3µM_x000d_ | 0.0 |
| Ng 11-11 10µM_x000d_ | 0.0 |
| Ng 12-11 1 µM_x000d_ | 0.0 |
| Ng 12-11 3 µM_x000d_ | 0.0 |
| Ng 13-15 1 µM_x000d_ | 0.0 |
| Ng 13-15 3 µM_x000d_ | 0.0 |
| Ng 13-14_x000d_ | 2738.6666666666665 |
| Ng 14-10 1 µM_x000d_ | 2081.3333333333335 |
| Ng 14-10 3 µM_x000d_ | 7204.333333333333 |
| Ng 14-11 1 µM_x000d_ | 693.6666666666666 |
| Ng 14-12 1 µM_x000d_ | 9671.333333333334 |
| Ng 14-7 1 µM_x000d_ | 2251.0 |
| Ng 15-14/15 1 µM_x000d_ | 4046.666666666667 |
| Ng 15-12 1µM_x000d_ | 0.0 |
| Ng 17-7 1 µM_x000d_ | 126.0 |
| Ng 17-7 3 µM_x000d_ | 2335.6666666666665 |
| Ng 17-7 10 µM_x000d_ | 6262.333333333333 |
| Ng 19-9 1 µM_x000d_ | 478.0 |
| Ng 19-9 3 µM_x000d_ | 3857.3333333333335 |
| Ng 19-9 10 µM_x000d_ | 3930.3333333333335 |
| Ng 20-7 1 µM_x000d_ | 235.33333333333331 |
| Ng 20-7 3 µM_x000d_ | 3324.6666666666665 |
| Ng 20-7 10 µM_x000d_ | 10013.0 |
| Ng 21-8 1 µM_x000d_ | 28.666666666666657 |
| Ng 21-8 3 µM_x000d_ | 668.3333333333334 |
| Ng 21-8 10 µM_x000d_ | 5037.0 |
| Ng 15-13 3µM_x000d_ | 1205.0 |
| Ng 16-12 1 µM_x000d_ | 0.0 |
| Ng 17-9 1 µM_x000d_ | 341.0 |
| Ng 17-9 3 µM_x000d_ | 2308.0 |
| Ng 18-11 1 µM_x000d_ | 1631.3333333333333 |
| Ng 18-11 3 µM_x000d_ | 1964.0 |
| Ng 20-8 1 µM_x000d_ | 683.0 |
| Ng 20-9 1 µM_x000d_ | 1506.6666666666667 |
| Ng 20-9 3 µM_x000d_ | 2883.6666666666665 |
| Ng 21-9 1 µM_x000d_ | 1661.6666666666667 |
| Ng 13-13 3 µM_x000d_ | 0.0 |
| Ng 14-8 1 µM_x000d_ | 51.0 |
| Ng 14-9 1 µM_x000d_ | 0.0 |
| Ng 14-9 3 µM_x000d_ | 0.6666666666666572 |
| Ng 14-9 10 µM_x000d_ | 346.0 |
| Ng 14-9 30 µM_x000d_ | 452.33333333333337 |
| Ng 15-10 1µM_x000d_ | 4537.0 |
| Ng 16-9 1 µM_x000d_ | 1715.6666666666667 |
| Ng 17-8 1 µM_x000d_ | 382.0 |
| Ng 17-8 3 µM_x000d_ | 1995.6666666666665 |
| Ng 18-9 1µM_x000d_ | 127.33333333333331 |
| Ng 19-11 1 µM_x000d_ | 3839.0 |
| Ng 19-11 3 µM_x000d_ | 8411.0 |
| Lg 17-15 HF 10 µM_x000d_ | 866.6666666666666 |
| Lg 15-17 1 µM_x000d_ | 0.0 |
| Lg 17-15 1 µM_x000d_ | 62.66666666666666 |
| Lg 17-15 3 µM_x000d_ | 749.3333333333334 |
| Lg 17-15 10 µM_x000d_ | 3996.0 |
| Lg 17-17 1 µM_x000d_ | 105.0 |
| Lg 17-16 1 µM_x000d_ | 176.66666666666669 |
| Lg 16-17 3 µM_x000d_ | 1562.6666666666667 |
| Lg 18-8 1 µM_x000d_ | 502.33333333333337 |
| Lg 19-8 1 µM_x000d_ | 1451.3333333333333 |
| Lg 19-8 3 µM_x000d_ | 3340.6666666666665 |
| Lg 19-9 1 µM_x000d_ | 814.0 |
| Lg 20-10_x000d_ | 916.3333333333333 |
| Lg 22-12 1 µM_x000d_ | 558.3333333333334 |
| Lg 20-9 1 µM_x000d_ | 6809.0 |
| Lg 23-11 1 µM_x000d_ | 2929.0 |
| Lg 23-11 3 µM_x000d_ | 6234.333333333333 |
| Lg 23-12_x000d_ | 2018.6666666666665 |
| Lg 26-8_x000d_ | 3554.0 |
| Lg 26-9_x000d_ | 726.6666666666666 |
| Lg 22-13_x000d_ | 461.33333333333337 |
| Lg 26-10_x000d_ | 7.666666666666657 |
| Lg 18-7-F3 HF 1uM | 1709.6666666666667 |
| Lg 19-8-F5 HF 3uM | 5644.666666666667 |
| Lg 23-11-F2 HF 3uM | 2148.0 |
| Blank 1 | 0.0 |
| Blank 2 | 0.0 |
| Blank 3 | 0.0 |
| Blank 4 | 0.0 |
| Blank 5 | 0.0 |MFI
### Chart: Mp-2
| Category | Mp 38-202 IgG2 |
|---|---|
| Ng 7-13 1µM_x000d_ | 105.33333333333334 |
| Ng 7-13 3µM_x000d_ | 278.6666666666667 |
| Ng 9-9 1 µM_x000d_ | 0.0 |
| Ng 9-9 3µM_x000d_ | 120.66666666666669 |
| Ng 9-9 10µM_x000d_ | 192.0 |
| Ng 10-13 1 µM_x000d_ | 0.0 |
| Ng 10-13 3 µM_x000d_ | 0.0 |
| Ng 10-13 10 µM_x000d_ | 71.0 |
| Ng 10-13 30 µM_x000d_ | 54.66666666666666 |
| Ng 10-10 1 µM_x000d_ | 106.33333333333331 |
| Ng 10-10 3 µM_x000d_ | 36.66666666666666 |
| Ng 10-11 1 µM_x000d_ | 1.6666666666666572 |
| Ng 10-11 3 µM_x000d_ | 67.66666666666666 |
| Ng 10-12 1 µM_x000d_ | 40.33333333333334 |
| Ng 11-10 1 µM_x000d_ | 21.0 |
| Ng 11-10 3 µM_x000d_ | 27.0 |
| Ng 11-11 1 µM_x000d_ | 47.66666666666666 |
| Ng 11-11 3µM_x000d_ | 15.333333333333343 |
| Ng 11-11 10µM_x000d_ | 0.0 |
| Ng 12-11 1 µM_x000d_ | 1.0 |
| Ng 12-11 3 µM_x000d_ | 41.0 |
| Ng 13-15 1 µM_x000d_ | 0.0 |
| Ng 13-15 3 µM_x000d_ | 52.66666666666666 |
| Ng 13-14_x000d_ | 4143.666666666667 |
| Ng 14-10 1 µM_x000d_ | 2524.0 |
| Ng 14-10 3 µM_x000d_ | 24429.0 |
| Ng 14-11 1 µM_x000d_ | 731.6666666666666 |
| Ng 14-12 1 µM_x000d_ | 38755.333333333336 |
| Ng 14-7 1 µM_x000d_ | 2203.0 |
| Ng 15-14/15 1 µM_x000d_ | 12852.333333333334 |
| Ng 15-12 1µM_x000d_ | 0.0 |
| Ng 17-7 1 µM_x000d_ | 473.66666666666663 |
| Ng 17-7 3 µM_x000d_ | 6781.666666666667 |
| Ng 17-7 10 µM_x000d_ | 11389.666666666666 |
| Ng 19-9 1 µM_x000d_ | 1579.0 |
| Ng 19-9 3 µM_x000d_ | 5336.666666666667 |
| Ng 19-9 10 µM_x000d_ | 4108.666666666667 |
| Ng 20-7 1 µM_x000d_ | 2290.6666666666665 |
| Ng 20-7 3 µM_x000d_ | 5701.666666666667 |
| Ng 20-7 10 µM_x000d_ | 19141.333333333332 |
| Ng 21-8 1 µM_x000d_ | 55.0 |
| Ng 21-8 3 µM_x000d_ | 4361.333333333333 |
| Ng 21-8 10 µM_x000d_ | 27006.333333333332 |
| Ng 15-13 3µM_x000d_ | 999.3333333333333 |
| Ng 16-12 1 µM_x000d_ | 0.6666666666666572 |
| Ng 17-9 1 µM_x000d_ | 2733.0 |
| Ng 17-9 3 µM_x000d_ | 5381.333333333333 |
| Ng 18-11 1 µM_x000d_ | 8256.0 |
| Ng 18-11 3 µM_x000d_ | 4416.0 |
| Ng 20-8 1 µM_x000d_ | 5822.666666666667 |
| Ng 20-9 1 µM_x000d_ | 1592.3333333333333 |
| Ng 20-9 3 µM_x000d_ | 3970.666666666667 |
| Ng 21-9 1 µM_x000d_ | 2894.0 |
| Ng 13-13 3 µM_x000d_ | 33.0 |
| Ng 14-8 1 µM_x000d_ | 207.33333333333331 |
| Ng 14-9 1 µM_x000d_ | 2.0 |
| Ng 14-9 3 µM_x000d_ | 95.33333333333334 |
| Ng 14-9 10 µM_x000d_ | 160.33333333333331 |
| Ng 14-9 30 µM_x000d_ | 168.0 |
| Ng 15-10 1µM_x000d_ | 32185.0 |
| Ng 16-9 1 µM_x000d_ | 4956.666666666667 |
| Ng 17-8 1 µM_x000d_ | 1373.0 |
| Ng 17-8 3 µM_x000d_ | 2668.6666666666665 |
| Ng 18-9 1µM_x000d_ | 416.0 |
| Ng 19-11 1 µM_x000d_ | 9639.0 |
| Ng 19-11 3 µM_x000d_ | 27415.0 |
| Lg 17-15 HF 10 µM_x000d_ | 15679.666666666666 |
| Lg 15-17 1 µM_x000d_ | 908.6666666666667 |
| Lg 17-15 1 µM_x000d_ | 2747.0 |
| Lg 17-15 3 µM_x000d_ | 3950.666666666667 |
| Lg 17-15 10 µM_x000d_ | 12232.666666666666 |
| Lg 17-17 1 µM_x000d_ | 868.0 |
| Lg 17-16 1 µM_x000d_ | 1379.6666666666667 |
| Lg 16-17 3 µM_x000d_ | 6294.666666666667 |
| Lg 18-8 1 µM_x000d_ | 2897.3333333333335 |
| Lg 19-8 1 µM_x000d_ | 3492.0 |
| Lg 19-8 3 µM_x000d_ | 35304.666666666664 |
| Lg 19-9 1 µM_x000d_ | 2754.0 |
| Lg 20-10_x000d_ | 3913.3333333333335 |
| Lg 22-12 1 µM_x000d_ | 1112.0 |
| Lg 20-9 1 µM_x000d_ | 47158.0 |
| Lg 23-11 1 µM_x000d_ | 25178.666666666668 |
| Lg 23-11 3 µM_x000d_ | 63026.0 |
| Lg 23-12_x000d_ | 5714.0 |
| Lg 26-8_x000d_ | 18789.0 |
| Lg 26-9_x000d_ | 2220.0 |
| Lg 22-13_x000d_ | 1633.6666666666667 |
| Lg 26-10_x000d_ | 482.0 |
| Lg 18-7-F3 HF 1uM | 1619.0 |
| Lg 19-8-F5 HF 3uM | 25567.0 |
| Lg 23-11-F2 HF 3uM | 4798.333333333333 |
| Blank 1 | 0.0 |
| Blank 2 | 0.0 |
| Blank 3 | 0.0 |
| Blank 4 | 0.0 |
| Blank 5 | 0.0 |MFI
### Chart: Mp-3
| Category | Mp39-64 IgG2 |
|---|---|
| Ng 7-13 1µM_x000d_ | 86.33333333333334 |
| Ng 7-13 3µM_x000d_ | 405.0 |
| Ng 9-9 1 µM_x000d_ | 5.333333333333343 |
| Ng 9-9 3µM_x000d_ | 571.6666666666666 |
| Ng 9-9 10µM_x000d_ | 238.66666666666669 |
| Ng 10-13 1 µM_x000d_ | 0.0 |
| Ng 10-13 3 µM_x000d_ | 0.0 |
| Ng 10-13 10 µM_x000d_ | 4.333333333333343 |
| Ng 10-13 30 µM_x000d_ | 0.0 |
| Ng 10-10 1 µM_x000d_ | 14.333333333333343 |
| Ng 10-10 3 µM_x000d_ | 38.66666666666666 |
| Ng 10-11 1 µM_x000d_ | 13.333333333333343 |
| Ng 10-11 3 µM_x000d_ | 41.0 |
| Ng 10-12 1 µM_x000d_ | 3.0 |
| Ng 11-10 1 µM_x000d_ | 10.0 |
| Ng 11-10 3 µM_x000d_ | 15.0 |
| Ng 11-11 1 µM_x000d_ | 0.0 |
| Ng 11-11 3µM_x000d_ | 0.3333333333333428 |
| Ng 11-11 10µM_x000d_ | 0.0 |
| Ng 12-11 1 µM_x000d_ | 0.0 |
| Ng 12-11 3 µM_x000d_ | 0.0 |
| Ng 13-15 1 µM_x000d_ | 0.0 |
| Ng 13-15 3 µM_x000d_ | 0.0 |
| Ng 13-14_x000d_ | 635.3333333333334 |
| Ng 14-10 1 µM_x000d_ | 621.3333333333334 |
| Ng 14-10 3 µM_x000d_ | 691.3333333333334 |
| Ng 14-11 1 µM_x000d_ | 45.0 |
| Ng 14-12 1 µM_x000d_ | 2624.3333333333335 |
| Ng 14-7 1 µM_x000d_ | 290.3333333333333 |
| Ng 15-14/15 1 µM_x000d_ | 779.0 |
| Ng 15-12 1µM_x000d_ | 0.0 |
| Ng 17-7 1 µM_x000d_ | 0.0 |
| Ng 17-7 3 µM_x000d_ | 189.0 |
| Ng 17-7 10 µM_x000d_ | 1074.0 |
| Ng 19-9 1 µM_x000d_ | 209.33333333333331 |
| Ng 19-9 3 µM_x000d_ | 939.3333333333333 |
| Ng 19-9 10 µM_x000d_ | 0.3333333333333428 |
| Ng 20-7 1 µM_x000d_ | 12.666666666666657 |
| Ng 20-7 3 µM_x000d_ | 83.0 |
| Ng 20-7 10 µM_x000d_ | 875.6666666666667 |
| Ng 21-8 1 µM_x000d_ | 0.0 |
| Ng 21-8 3 µM_x000d_ | 0.0 |
| Ng 21-8 10 µM_x000d_ | 1120.3333333333333 |
| Ng 15-13 3µM_x000d_ | 171.0 |
| Ng 16-12 1 µM_x000d_ | 0.0 |
| Ng 17-9 1 µM_x000d_ | 0.0 |
| Ng 17-9 3 µM_x000d_ | 489.66666666666663 |
| Ng 18-11 1 µM_x000d_ | 157.0 |
| Ng 18-11 3 µM_x000d_ | 123.66666666666669 |
| Ng 20-8 1 µM_x000d_ | 0.0 |
| Ng 20-9 1 µM_x000d_ | 93.33333333333334 |
| Ng 20-9 3 µM_x000d_ | 945.0 |
| Ng 21-9 1 µM_x000d_ | 76.66666666666666 |
| Ng 13-13 3 µM_x000d_ | 0.0 |
| Ng 14-8 1 µM_x000d_ | 3.666666666666657 |
| Ng 14-9 1 µM_x000d_ | 0.0 |
| Ng 14-9 3 µM_x000d_ | 14.0 |
| Ng 14-9 10 µM_x000d_ | 105.33333333333334 |
| Ng 14-9 30 µM_x000d_ | 60.66666666666666 |
| Ng 15-10 1µM_x000d_ | 99.0 |
| Ng 16-9 1 µM_x000d_ | 147.0 |
| Ng 17-8 1 µM_x000d_ | 67.66666666666666 |
| Ng 17-8 3 µM_x000d_ | 364.0 |
| Ng 18-9 1µM_x000d_ | 41.33333333333334 |
| Ng 19-11 1 µM_x000d_ | 245.66666666666669 |
| Ng 19-11 3 µM_x000d_ | 2706.6666666666665 |
| Lg 17-15 HF 10 µM_x000d_ | 2722.3333333333335 |
| Lg 15-17 1 µM_x000d_ | 1870.0 |
| Lg 17-15 1 µM_x000d_ | 2313.0 |
| Lg 17-15 3 µM_x000d_ | 5591.0 |
| Lg 17-15 10 µM_x000d_ | 14397.666666666666 |
| Lg 17-17 1 µM_x000d_ | 1944.0 |
| Lg 17-16 1 µM_x000d_ | 3552.0 |
| Lg 16-17 3 µM_x000d_ | 1477.0 |
| Lg 18-8 1 µM_x000d_ | 1589.6666666666667 |
| Lg 19-8 1 µM_x000d_ | 217.33333333333331 |
| Lg 19-8 3 µM_x000d_ | 498.0 |
| Lg 19-9 1 µM_x000d_ | 2139.3333333333335 |
| Lg 20-10_x000d_ | 54.33333333333334 |
| Lg 22-12 1 µM_x000d_ | 56.33333333333334 |
| Lg 20-9 1 µM_x000d_ | 1935.6666666666665 |
| Lg 23-11 1 µM_x000d_ | 151.0 |
| Lg 23-11 3 µM_x000d_ | 2551.6666666666665 |
| Lg 23-12_x000d_ | 964.6666666666667 |
| Lg 26-8_x000d_ | 59.66666666666666 |
| Lg 26-9_x000d_ | 42.33333333333334 |
| Lg 22-13_x000d_ | 171.66666666666669 |
| Lg 26-10_x000d_ | 0.0 |
| Lg 18-7-F3 HF 1uM | 499.66666666666663 |
| Lg 19-8-F5 HF 3uM | 2376.6666666666665 |
| Lg 23-11-F2 HF 3uM | 490.33333333333337 |
| Blank 1 | 0.0 |
| Blank 2 | 0.0 |
| Blank 3 | 0.0 |
| Blank 4 | 0.0 |
| Blank 5 | 0.0 |MFI
### Chart: Mp-4
| Category | Mp 39-115 IgG2 |
|---|---|
| Ng 7-13 1µM_x000d_ | 186.33333333333331 |
| Ng 7-13 3µM_x000d_ | 372.0 |
| Ng 9-9 1 µM_x000d_ | 0.0 |
| Ng 9-9 3µM_x000d_ | 265.0 |
| Ng 9-9 10µM_x000d_ | 185.33333333333331 |
| Ng 10-13 1 µM_x000d_ | 0.0 |
| Ng 10-13 3 µM_x000d_ | 0.0 |
| Ng 10-13 10 µM_x000d_ | 19.0 |
| Ng 10-13 30 µM_x000d_ | 105.66666666666666 |
| Ng 10-10 1 µM_x000d_ | 1.3333333333333428 |
| Ng 10-10 3 µM_x000d_ | 63.66666666666666 |
| Ng 10-11 1 µM_x000d_ | 16.0 |
| Ng 10-11 3 µM_x000d_ | 65.33333333333334 |
| Ng 10-12 1 µM_x000d_ | 20.0 |
| Ng 11-10 1 µM_x000d_ | 3.0 |
| Ng 11-10 3 µM_x000d_ | 154.0 |
| Ng 11-11 1 µM_x000d_ | 0.6666666666666572 |
| Ng 11-11 3µM_x000d_ | 28.0 |
| Ng 11-11 10µM_x000d_ | 0.0 |
| Ng 12-11 1 µM_x000d_ | 0.0 |
| Ng 12-11 3 µM_x000d_ | 39.33333333333334 |
| Ng 13-15 1 µM_x000d_ | 0.0 |
| Ng 13-15 3 µM_x000d_ | 17.0 |
| Ng 13-14_x000d_ | 1894.0 |
| Ng 14-10 1 µM_x000d_ | 266.0 |
| Ng 14-10 3 µM_x000d_ | 3289.3333333333335 |
| Ng 14-11 1 µM_x000d_ | 141.33333333333331 |
| Ng 14-12 1 µM_x000d_ | 8171.0 |
| Ng 14-7 1 µM_x000d_ | 2263.3333333333335 |
| Ng 15-14/15 1 µM_x000d_ | 1388.3333333333333 |
| Ng 15-12 1µM_x000d_ | 22.333333333333343 |
| Ng 17-7 1 µM_x000d_ | 679.0 |
| Ng 17-7 3 µM_x000d_ | 4002.0 |
| Ng 17-7 10 µM_x000d_ | 8797.333333333334 |
| Ng 19-9 1 µM_x000d_ | 723.3333333333334 |
| Ng 19-9 3 µM_x000d_ | 4662.666666666667 |
| Ng 19-9 10 µM_x000d_ | 5740.0 |
| Ng 20-7 1 µM_x000d_ | 295.6666666666667 |
| Ng 20-7 3 µM_x000d_ | 4910.333333333333 |
| Ng 20-7 10 µM_x000d_ | 12782.0 |
| Ng 21-8 1 µM_x000d_ | 79.0 |
| Ng 21-8 3 µM_x000d_ | 3590.6666666666665 |
| Ng 21-8 10 µM_x000d_ | 14540.0 |
| Ng 15-13 3µM_x000d_ | 333.0 |
| Ng 16-12 1 µM_x000d_ | 9.0 |
| Ng 17-9 1 µM_x000d_ | 3164.3333333333335 |
| Ng 17-9 3 µM_x000d_ | 6272.0 |
| Ng 18-11 1 µM_x000d_ | 5629.0 |
| Ng 18-11 3 µM_x000d_ | 5011.666666666667 |
| Ng 20-8 1 µM_x000d_ | 3304.0 |
| Ng 20-9 1 µM_x000d_ | 2952.3333333333335 |
| Ng 20-9 3 µM_x000d_ | 7184.0 |
| Ng 21-9 1 µM_x000d_ | 1458.0 |
| Ng 13-13 3 µM_x000d_ | 18.333333333333343 |
| Ng 14-8 1 µM_x000d_ | 98.33333333333334 |
| Ng 14-9 1 µM_x000d_ | 0.0 |
| Ng 14-9 3 µM_x000d_ | 44.33333333333334 |
| Ng 14-9 10 µM_x000d_ | 162.66666666666669 |
| Ng 14-9 30 µM_x000d_ | 174.33333333333331 |
| Ng 15-10 1µM_x000d_ | 10952.0 |
| Ng 16-9 1 µM_x000d_ | 4491.666666666667 |
| Ng 17-8 1 µM_x000d_ | 932.0 |
| Ng 17-8 3 µM_x000d_ | 4266.0 |
| Ng 18-9 1µM_x000d_ | 291.0 |
| Ng 19-11 1 µM_x000d_ | 8755.0 |
| Ng 19-11 3 µM_x000d_ | 8223.333333333334 |
| Lg 17-15 HF 10 µM_x000d_ | 58799.666666666664 |
| Lg 15-17 1 µM_x000d_ | 6108.333333333333 |
| Lg 17-15 1 µM_x000d_ | 3762.3333333333335 |
| Lg 17-15 3 µM_x000d_ | 15706.666666666666 |
| Lg 17-15 10 µM_x000d_ | 45837.666666666664 |
| Lg 17-17 1 µM_x000d_ | 2283.3333333333335 |
| Lg 17-16 1 µM_x000d_ | 1235.6666666666667 |
| Lg 16-17 3 µM_x000d_ | 2791.0 |
| Lg 18-8 1 µM_x000d_ | 2828.0 |
| Lg 19-8 1 µM_x000d_ | 1780.6666666666667 |
| Lg 19-8 3 µM_x000d_ | 13978.333333333334 |
| Lg 19-9 1 µM_x000d_ | 2216.6666666666665 |
| Lg 20-10_x000d_ | 1963.6666666666665 |
| Lg 22-12 1 µM_x000d_ | 265.3333333333333 |
| Lg 20-9 1 µM_x000d_ | 16554.333333333332 |
| Lg 23-11 1 µM_x000d_ | 5741.333333333333 |
| Lg 23-11 3 µM_x000d_ | 23420.666666666668 |
| Lg 23-12_x000d_ | 2261.0 |
| Lg 26-8_x000d_ | 4208.333333333333 |
| Lg 26-9_x000d_ | 262.0 |
| Lg 22-13_x000d_ | 327.3333333333333 |
| Lg 26-10_x000d_ | 14.0 |
| Lg 18-7-F3 HF 1uM | 1944.6666666666665 |
| Lg 19-8-F5 HF 3uM | 13136.0 |
| Lg 23-11-F2 HF 3uM | 2672.0 |
| Blank 1 | 0.0 |
| Blank 2 | 0.0 |
| Blank 3 | 0.0 |
| Blank 4 | 0.0 |
| Blank 5 | 0.0 |MFI
### Chart: Mp-5
| Category | Mp k-26 IgG2 |
|---|---|
| Ng 7-13 1µM_x000d_ | 66.66666666666666 |
| Ng 7-13 3µM_x000d_ | 377.0 |
| Ng 9-9 1 µM_x000d_ | 14.666666666666657 |
| Ng 9-9 3µM_x000d_ | 57.33333333333334 |
| Ng 9-9 10µM_x000d_ | 66.0 |
| Ng 10-13 1 µM_x000d_ | 0.0 |
| Ng 10-13 3 µM_x000d_ | 0.0 |
| Ng 10-13 10 µM_x000d_ | 51.66666666666666 |
| Ng 10-13 30 µM_x000d_ | 2.0 |
| Ng 10-10 1 µM_x000d_ | 41.0 |
| Ng 10-10 3 µM_x000d_ | 85.66666666666666 |
| Ng 10-11 1 µM_x000d_ | 13.5 |
| Ng 10-11 3 µM_x000d_ | 22.5 |
| Ng 10-12 1 µM_x000d_ | 87.66666666666666 |
| Ng 11-10 1 µM_x000d_ | 0.0 |
| Ng 11-10 3 µM_x000d_ | 34.33333333333334 |
| Ng 11-11 1 µM_x000d_ | 21.666666666666657 |
| Ng 11-11 3µM_x000d_ | 69.0 |
| Ng 11-11 10µM_x000d_ | 0.0 |
| Ng 12-11 1 µM_x000d_ | 27.0 |
| Ng 12-11 3 µM_x000d_ | 55.0 |
| Ng 13-15 1 µM_x000d_ | 0.0 |
| Ng 13-15 3 µM_x000d_ | 83.0 |
| Ng 13-14_x000d_ | 1805.6666666666667 |
| Ng 14-10 1 µM_x000d_ | 84.66666666666666 |
| Ng 14-10 3 µM_x000d_ | 1230.6666666666667 |
| Ng 14-11 1 µM_x000d_ | 79.66666666666666 |
| Ng 14-12 1 µM_x000d_ | 2392.0 |
| Ng 14-7 1 µM_x000d_ | 1418.0 |
| Ng 15-14/15 1 µM_x000d_ | 763.6666666666666 |
| Ng 15-12 1µM_x000d_ | 0.0 |
| Ng 17-7 1 µM_x000d_ | 407.66666666666663 |
| Ng 17-7 3 µM_x000d_ | 2977.0 |
| Ng 17-7 10 µM_x000d_ | 5141.333333333333 |
| Ng 19-9 1 µM_x000d_ | 1026.0 |
| Ng 19-9 3 µM_x000d_ | 2134.6666666666665 |
| Ng 19-9 10 µM_x000d_ | 1460.3333333333333 |
| Ng 20-7 1 µM_x000d_ | 848.6666666666666 |
| Ng 20-7 3 µM_x000d_ | 3347.3333333333335 |
| Ng 20-7 10 µM_x000d_ | 3985.0 |
| Ng 21-8 1 µM_x000d_ | 0.0 |
| Ng 21-8 3 µM_x000d_ | 417.0 |
| Ng 21-8 10 µM_x000d_ | 4404.333333333333 |
| Ng 15-13 3µM_x000d_ | 260.3333333333333 |
| Ng 16-12 1 µM_x000d_ | 0.6666666666666572 |
| Ng 17-9 1 µM_x000d_ | 363.66666666666663 |
| Ng 17-9 3 µM_x000d_ | 1261.3333333333333 |
| Ng 18-11 1 µM_x000d_ | 92.0 |
| Ng 18-11 3 µM_x000d_ | 101.66666666666666 |
| Ng 20-8 1 µM_x000d_ | 521.3333333333334 |
| Ng 20-9 1 µM_x000d_ | 209.0 |
| Ng 20-9 3 µM_x000d_ | 388.0 |
| Ng 21-9 1 µM_x000d_ | 212.0 |
| Ng 13-13 3 µM_x000d_ | 29.666666666666657 |
| Ng 14-8 1 µM_x000d_ | 156.33333333333331 |
| Ng 14-9 1 µM_x000d_ | 0.0 |
| Ng 14-9 3 µM_x000d_ | 46.66666666666666 |
| Ng 14-9 10 µM_x000d_ | 85.66666666666666 |
| Ng 14-9 30 µM_x000d_ | 54.33333333333334 |
| Ng 15-10 1µM_x000d_ | 8728.666666666666 |
| Ng 16-9 1 µM_x000d_ | 1993.6666666666665 |
| Ng 17-8 1 µM_x000d_ | 204.0 |
| Ng 17-8 3 µM_x000d_ | 447.66666666666663 |
| Ng 18-9 1µM_x000d_ | 201.0 |
| Ng 19-11 1 µM_x000d_ | 396.33333333333337 |
| Ng 19-11 3 µM_x000d_ | 971.6666666666667 |
| Lg 17-15 HF 10 µM_x000d_ | 10600.0 |
| Lg 15-17 1 µM_x000d_ | 153.33333333333331 |
| Lg 17-15 1 µM_x000d_ | 7563.666666666667 |
| Lg 17-15 3 µM_x000d_ | 26759.666666666668 |
| Lg 17-15 10 µM_x000d_ | 42344.0 |
| Lg 17-17 1 µM_x000d_ | 7486.333333333333 |
| Lg 17-16 1 µM_x000d_ | 3551.6666666666665 |
| Lg 16-17 3 µM_x000d_ | 1548.6666666666667 |
| Lg 18-8 1 µM_x000d_ | 9693.666666666666 |
| Lg 19-8 1 µM_x000d_ | 2288.6666666666665 |
| Lg 19-8 3 µM_x000d_ | 16014.333333333334 |
| Lg 19-9 1 µM_x000d_ | 7920.333333333333 |
| Lg 20-10_x000d_ | 1760.3333333333333 |
| Lg 22-12 1 µM_x000d_ | 78.0 |
| Lg 20-9 1 µM_x000d_ | 10450.0 |
| Lg 23-11 1 µM_x000d_ | 5426.666666666667 |
| Lg 23-11 3 µM_x000d_ | 15305.0 |
| Lg 23-12_x000d_ | 1275.0 |
| Lg 26-8_x000d_ | 3857.3333333333335 |
| Lg 26-9_x000d_ | 984.6666666666667 |
| Lg 22-13_x000d_ | 248.0 |
| Lg 26-10_x000d_ | 2.0 |
| Lg 18-7-F3 HF 1uM | 424.33333333333337 |
| Lg 19-8-F5 HF 3uM | 9518.666666666666 |
| Lg 23-11-F2 HF 3uM | 2665.0 |
| Blank 1 | 0.0 |
| Blank 2 | 0.0 |
| Blank 3 | 0.0 |
| Blank 4 | 0.0 |
| Blank 5 | 0.0 |MFI
### Chart: Mp-6
| Category | Mp L23 IgG2 |
|---|---|
| Ng1_1uM
 | 463.0 |
| Ng1_3uM
 | 1578.3333333333333 |
| Ng2_1uM | 447.33333333333337 |
| Ng2_3uM | 1623.0 |
| Ng2_10uM | 453.33333333333337 |
| Ng3_1uM | 28.0 |
| Ng3_3uM | 80.0 |
| Ng3_10uM | 21.0 |
| Ng3_30uM | 3.333333333333343 |
| Ng4_1uM | 184.0 |
| Ng4_3uM | 120.66666666666669 |
| Ng5_1uM | 230.33333333333331 |
| Ng5_3uM | 293.6666666666667 |
| Ng6_1uM | 81.0 |
| Ng7_1uM | 108.33333333333331 |
| Ng7_3uM | 110.33333333333331 |
| Ng8_1uM | 79.0 |
| Ng8_3uM | 109.0 |
| Ng8_10uM | 17.0 |
| Ng9_1uM | 60.33333333333334 |
| Ng9_3uM | 61.66666666666666 |
| Ng10_3uM | 0.0 |
| Ng10_1uM | 115.0 |
| Ng11 | 16739.0 |
| Ng12_1uM | 8571.0 |
| Ng12_3uM | 64974.666666666664 |
| Ng13_1uM | 3656.3333333333335 |
| Ng14_1uM | 63392.0 |
| Ng15_1uM | 16779.333333333332 |
| Ng16_1uM | 48279.0 |
| Ng17_1uM | 137.0 |
| Ng18_1uM | 4767.333333333333 |
| Ng18_3uM | 33200.666666666664 |
| Ng18_10uM | 64034.0 |
| Ng19_1uM | 9457.0 |
| Ng19_3uM | 25392.666666666668 |
| Ng19_10uM | 63810.666666666664 |
| Ng20_1uM | 6840.666666666667 |
| Ng20_3uM | 34890.0 |
| Ng20_10uM | 61352.333333333336 |
| Ng21_1uM | 1646.0 |
| Ng21 _3uM | 12821.0 |
| Ng21_10uM | 64989.666666666664 |
| Ng22_1uM | 4033.666666666667 |
| Ng23_1uM | 127.0 |
| Ng24_1uM | 7920.666666666667 |
| Ng24_3uM | 16546.666666666668 |
| Ng25_1uM | 5459.666666666667 |
| Ng25_3uM | 6257.666666666667 |
| Ng26_1uM | 9782.0 |
| Ng27_1uM | 8173.0 |
| Ng27_3uM | 21773.333333333332 |
| Ng28_1uM | 35403.0 |
| Ng29_1uM | 86.0 |
| Ng30_1uM | 2356.0 |
| Ng31_1uM | 50.0 |
| Ng31_3uM | 387.0 |
| Ng31_10uM | 618.0 |
| Ng31_30uM | 1678.3333333333333 |
| Ng32_1uM | 61588.0 |
| Ng33_1uM | 32260.333333333332 |
| Ng34_1uM | 3242.3333333333335 |
| Ng34_3uM | 6552.0 |
| Ng35_1uM | 2295.6666666666665 |
| Ng36_1uM | 64969.0 |
| Ng36_3uM | 56183.666666666664 |
| Lg2_HF_10uM | 38971.0 |
| Lg1_1uM | 1594.0 |
| Lg2_1uM | 6745.333333333333 |
| Lg2_3uM | 14167.666666666666 |
| Lg2_10uM | 31417.0 |
| Lg3_1uM | 5393.0 |
| Lg4_1uM | 3229.3333333333335 |
| Lg5_3uM | 4914.0 |
| Lg6_1uM | 12358.0 |
| Lg7_1uM | 20448.0 |
| Lg7_3uM | 58276.0 |
| Lg8_1uM | 18349.666666666668 |
| Lg9 | 32761.333333333336 |
| Lg10_1uM | 5265.0 |
| Lg11_1uM | 61788.0 |
| Lg12_1uM | 58865.333333333336 |
| Lg12_3uM | 64988.0 |
| Lg13 | 30126.666666666668 |
| Lg14 | 57108.333333333336 |
| Lg15 | 6732.0 |
| Lg16 | 3801.0 |
| Lg17 | 1085.3333333333333 |
| Lg18_HF_1uM | 12928.666666666666 |
| Lg7_HF_3uM | 64619.666666666664 |
| Lg12_HF_3uM | 25608.333333333332 |
| Blank 1 | 0.0 |
| Blank 2 | 0.0 |
| Blank 3 | 0.0 |
| Blank 4 | 0.0 |
| Blank 5 | 8.666666666666657 |MFI

## Slide 31
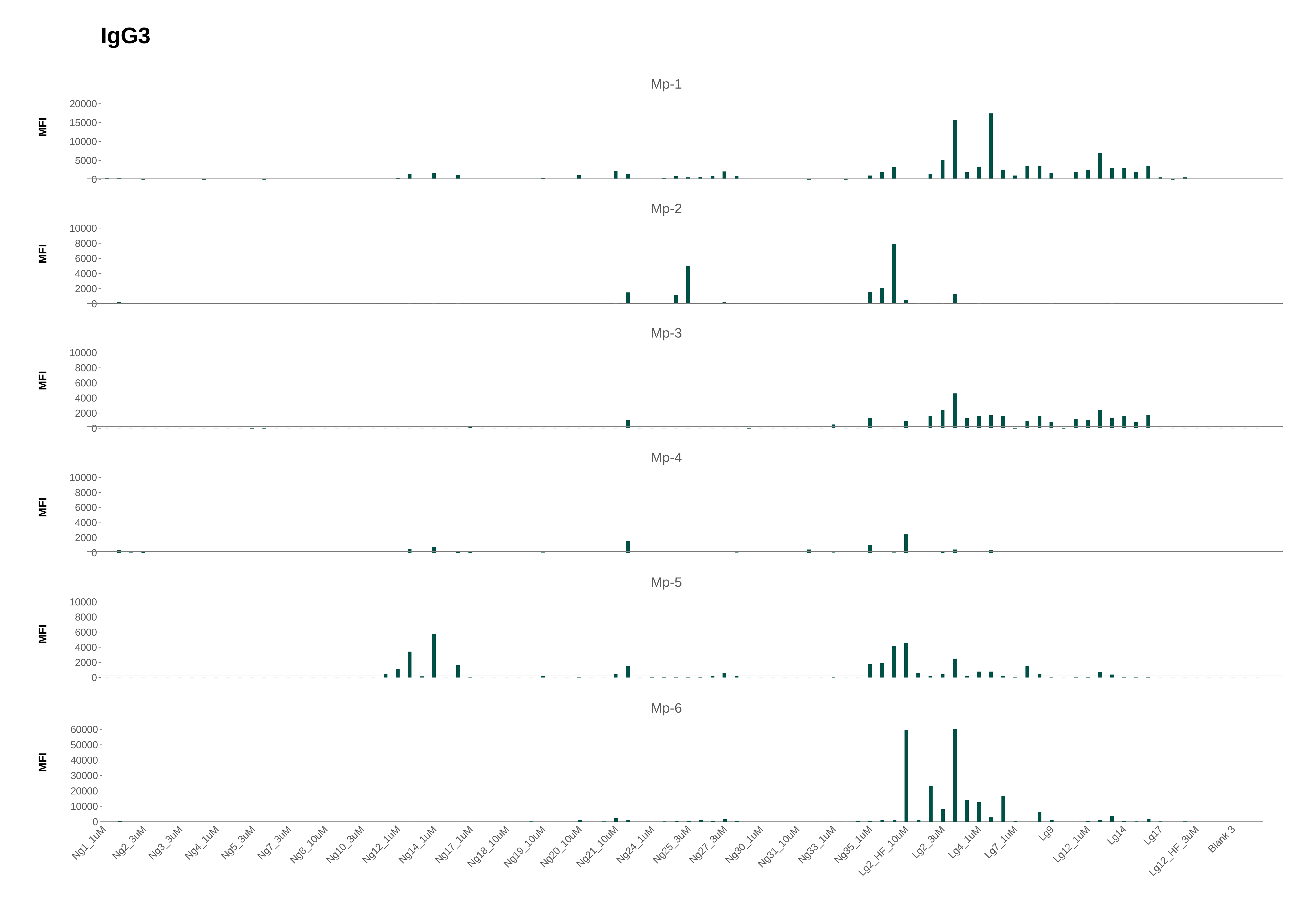

IgG3
### Chart: Mp-1
| Category | Mp 36-51 IgG3 |
|---|---|
| Ng 7-13 1µM_x000d_ | 370.0 |
| Ng 7-13 3µM_x000d_ | 338.6666666666667 |
| Ng 9-9 1 µM_x000d_ | 0.0 |
| Ng 9-9 3µM_x000d_ | 9.0 |
| Ng 9-9 10µM_x000d_ | 39.66666666666666 |
| Ng 10-13 1 µM_x000d_ | 0.0 |
| Ng 10-13 3 µM_x000d_ | 0.0 |
| Ng 10-13 10 µM_x000d_ | 0.0 |
| Ng 10-13 30 µM_x000d_ | 11.333333333333343 |
| Ng 10-10 1 µM_x000d_ | 0.0 |
| Ng 10-10 3 µM_x000d_ | 0.0 |
| Ng 10-11 1 µM_x000d_ | 0.0 |
| Ng 10-11 3 µM_x000d_ | 0.0 |
| Ng 10-12 1 µM_x000d_ | 2.0 |
| Ng 11-10 1 µM_x000d_ | 0.0 |
| Ng 11-10 3 µM_x000d_ | 0.0 |
| Ng 11-11 1 µM_x000d_ | 0.0 |
| Ng 11-11 3µM_x000d_ | 0.0 |
| Ng 11-11 10µM_x000d_ | 0.0 |
| Ng 12-11 1 µM_x000d_ | 0.0 |
| Ng 12-11 3 µM_x000d_ | 0.0 |
| Ng 13-15 1 µM_x000d_ | 0.0 |
| Ng 13-15 3 µM_x000d_ | 0.0 |
| Ng 13-14_x000d_ | 220.66666666666669 |
| Ng 14-10 1 µM_x000d_ | 252.66666666666669 |
| Ng 14-10 3 µM_x000d_ | 1515.6666666666667 |
| Ng 14-11 1 µM_x000d_ | 70.66666666666666 |
| Ng 14-12 1 µM_x000d_ | 1527.0 |
| Ng 14-7 1 µM_x000d_ | 0.0 |
| Ng 15-14/15 1 µM_x000d_ | 1093.3333333333333 |
| Ng 15-12 1µM_x000d_ | 116.66666666666669 |
| Ng 17-7 1 µM_x000d_ | 0.0 |
| Ng 17-7 3 µM_x000d_ | 0.0 |
| Ng 17-7 10 µM_x000d_ | 135.33333333333331 |
| Ng 19-9 1 µM_x000d_ | 0.0 |
| Ng 19-9 3 µM_x000d_ | 68.0 |
| Ng 19-9 10 µM_x000d_ | 274.3333333333333 |
| Ng 20-7 1 µM_x000d_ | 0.0 |
| Ng 20-7 3 µM_x000d_ | 133.33333333333331 |
| Ng 20-7 10 µM_x000d_ | 1056.6666666666667 |
| Ng 21-8 1 µM_x000d_ | 0.0 |
| Ng 21-8 3 µM_x000d_ | 203.33333333333331 |
| Ng 21-8 10 µM_x000d_ | 2277.6666666666665 |
| Ng 15-13 3µM_x000d_ | 1313.6666666666667 |
| Ng 16-12 1 µM_x000d_ | 0.0 |
| Ng 17-9 1 µM_x000d_ | 0.0 |
| Ng 17-9 3 µM_x000d_ | 347.3333333333333 |
| Ng 18-11 1 µM_x000d_ | 779.6666666666666 |
| Ng 18-11 3 µM_x000d_ | 453.33333333333337 |
| Ng 20-8 1 µM_x000d_ | 651.0 |
| Ng 20-9 1 µM_x000d_ | 870.6666666666666 |
| Ng 20-9 3 µM_x000d_ | 2054.6666666666665 |
| Ng 21-9 1 µM_x000d_ | 864.6666666666666 |
| Ng 13-13 3 µM_x000d_ | 0.0 |
| Ng 14-8 1 µM_x000d_ | 0.0 |
| Ng 14-9 1 µM_x000d_ | 0.0 |
| Ng 14-9 3 µM_x000d_ | 0.0 |
| Ng 14-9 10 µM_x000d_ | 0.0 |
| Ng 14-9 30 µM_x000d_ | 6.666666666666657 |
| Ng 15-10 1µM_x000d_ | 90.33333333333334 |
| Ng 16-9 1 µM_x000d_ | 25.666666666666657 |
| Ng 17-8 1 µM_x000d_ | 17.666666666666657 |
| Ng 17-8 3 µM_x000d_ | 169.0 |
| Ng 18-9 1µM_x000d_ | 991.3333333333333 |
| Ng 19-11 1 µM_x000d_ | 1843.0 |
| Ng 19-11 3 µM_x000d_ | 3208.6666666666665 |
| Lg 17-15 HF 10 µM_x000d_ | 174.66666666666669 |
| Lg 15-17 1 µM_x000d_ | 0.0 |
| Lg 17-15 1 µM_x000d_ | 1471.3333333333333 |
| Lg 17-15 3 µM_x000d_ | 5037.666666666667 |
| Lg 17-15 10 µM_x000d_ | 15641.0 |
| Lg 17-17 1 µM_x000d_ | 1859.3333333333333 |
| Lg 17-16 1 µM_x000d_ | 3347.0 |
| Lg 16-17 3 µM_x000d_ | 17402.0 |
| Lg 18-8 1 µM_x000d_ | 2406.6666666666665 |
| Lg 19-8 1 µM_x000d_ | 951.6666666666667 |
| Lg 19-8 3 µM_x000d_ | 3564.0 |
| Lg 19-9 1 µM_x000d_ | 3411.0 |
| Lg 20-10_x000d_ | 1556.3333333333333 |
| Lg 22-12 1 µM_x000d_ | 136.0 |
| Lg 20-9 1 µM_x000d_ | 1986.3333333333335 |
| Lg 23-11 1 µM_x000d_ | 2393.6666666666665 |
| Lg 23-11 3 µM_x000d_ | 7015.0 |
| Lg 23-12_x000d_ | 3083.6666666666665 |
| Lg 26-8_x000d_ | 2951.6666666666665 |
| Lg 26-9_x000d_ | 1901.6666666666665 |
| Lg 22-13_x000d_ | 3522.6666666666665 |
| Lg 26-10_x000d_ | 494.33333333333337 |
| Lg 18-7-F3 HF 1uM | 20.0 |
| Lg 19-8-F5 HF 3uM | 476.0 |
| Lg 23-11-F2 HF 3uM | 61.33333333333334 |
| Blank 1 | 0.0 |
| Blank 2 | 0.0 |
| Blank 3 | 0.0 |
| Blank 4 | 0.0 |
| Blank 5 | 0.0 |MFI
### Chart: Mp-2
| Category | Mp 38-202 IgG3 |
|---|---|
| Ng 7-13 1µM_x000d_ | 27.666666666666657 |
| Ng 7-13 3µM_x000d_ | 239.0 |
| Ng 9-9 1 µM_x000d_ | 0.0 |
| Ng 9-9 3µM_x000d_ | 0.0 |
| Ng 9-9 10µM_x000d_ | 0.0 |
| Ng 10-13 1 µM_x000d_ | 0.0 |
| Ng 10-13 3 µM_x000d_ | 0.0 |
| Ng 10-13 10 µM_x000d_ | 0.0 |
| Ng 10-13 30 µM_x000d_ | 0.0 |
| Ng 10-10 1 µM_x000d_ | 0.0 |
| Ng 10-10 3 µM_x000d_ | 0.0 |
| Ng 10-11 1 µM_x000d_ | 0.0 |
| Ng 10-11 3 µM_x000d_ | 0.0 |
| Ng 10-12 1 µM_x000d_ | 0.0 |
| Ng 11-10 1 µM_x000d_ | 0.0 |
| Ng 11-10 3 µM_x000d_ | 0.0 |
| Ng 11-11 1 µM_x000d_ | 0.0 |
| Ng 11-11 3µM_x000d_ | 0.0 |
| Ng 11-11 10µM_x000d_ | 0.0 |
| Ng 12-11 1 µM_x000d_ | 0.0 |
| Ng 12-11 3 µM_x000d_ | 0.0 |
| Ng 13-15 1 µM_x000d_ | 0.0 |
| Ng 13-15 3 µM_x000d_ | 0.0 |
| Ng 13-14_x000d_ | 0.0 |
| Ng 14-10 1 µM_x000d_ | 0.0 |
| Ng 14-10 3 µM_x000d_ | 1.6666666666666572 |
| Ng 14-11 1 µM_x000d_ | 0.0 |
| Ng 14-12 1 µM_x000d_ | 110.66666666666669 |
| Ng 14-7 1 µM_x000d_ | 0.0 |
| Ng 15-14/15 1 µM_x000d_ | 145.0 |
| Ng 15-12 1µM_x000d_ | 70.33333333333334 |
| Ng 17-7 1 µM_x000d_ | 0.0 |
| Ng 17-7 3 µM_x000d_ | 0.0 |
| Ng 17-7 10 µM_x000d_ | 0.0 |
| Ng 19-9 1 µM_x000d_ | 0.0 |
| Ng 19-9 3 µM_x000d_ | 0.0 |
| Ng 19-9 10 µM_x000d_ | 40.0 |
| Ng 20-7 1 µM_x000d_ | 0.0 |
| Ng 20-7 3 µM_x000d_ | 0.0 |
| Ng 20-7 10 µM_x000d_ | 0.0 |
| Ng 21-8 1 µM_x000d_ | 0.0 |
| Ng 21-8 3 µM_x000d_ | 0.0 |
| Ng 21-8 10 µM_x000d_ | 97.33333333333334 |
| Ng 15-13 3µM_x000d_ | 1490.0 |
| Ng 16-12 1 µM_x000d_ | 0.0 |
| Ng 17-9 1 µM_x000d_ | 0.0 |
| Ng 17-9 3 µM_x000d_ | 0.0 |
| Ng 18-11 1 µM_x000d_ | 1156.6666666666667 |
| Ng 18-11 3 µM_x000d_ | 5027.666666666667 |
| Ng 20-8 1 µM_x000d_ | 0.0 |
| Ng 20-9 1 µM_x000d_ | 0.0 |
| Ng 20-9 3 µM_x000d_ | 289.6666666666667 |
| Ng 21-9 1 µM_x000d_ | 0.0 |
| Ng 13-13 3 µM_x000d_ | 0.0 |
| Ng 14-8 1 µM_x000d_ | 0.0 |
| Ng 14-9 1 µM_x000d_ | 0.0 |
| Ng 14-9 3 µM_x000d_ | 0.0 |
| Ng 14-9 10 µM_x000d_ | 0.0 |
| Ng 14-9 30 µM_x000d_ | 0.0 |
| Ng 15-10 1µM_x000d_ | 0.0 |
| Ng 16-9 1 µM_x000d_ | 0.0 |
| Ng 17-8 1 µM_x000d_ | 0.0 |
| Ng 17-8 3 µM_x000d_ | 0.0 |
| Ng 18-9 1µM_x000d_ | 1557.0 |
| Ng 19-11 1 µM_x000d_ | 2086.6666666666665 |
| Ng 19-11 3 µM_x000d_ | 7895.0 |
| Lg 17-15 HF 10 µM_x000d_ | 532.6666666666666 |
| Lg 15-17 1 µM_x000d_ | 4.0 |
| Lg 17-15 1 µM_x000d_ | 0.0 |
| Lg 17-15 3 µM_x000d_ | 5.333333333333343 |
| Lg 17-15 10 µM_x000d_ | 1335.6666666666667 |
| Lg 17-17 1 µM_x000d_ | 0.0 |
| Lg 17-16 1 µM_x000d_ | 94.0 |
| Lg 16-17 3 µM_x000d_ | 0.0 |
| Lg 18-8 1 µM_x000d_ | 0.0 |
| Lg 19-8 1 µM_x000d_ | 0.0 |
| Lg 19-8 3 µM_x000d_ | 0.0 |
| Lg 19-9 1 µM_x000d_ | 45.0 |
| Lg 20-10_x000d_ | 2.666666666666657 |
| Lg 22-12 1 µM_x000d_ | 0.0 |
| Lg 20-9 1 µM_x000d_ | 0.0 |
| Lg 23-11 1 µM_x000d_ | 0.0 |
| Lg 23-11 3 µM_x000d_ | 0.0 |
| Lg 23-12_x000d_ | 3.333333333333343 |
| Lg 26-8_x000d_ | 48.66666666666666 |
| Lg 26-9_x000d_ | 0.0 |
| Lg 22-13_x000d_ | 31.666666666666657 |
| Lg 26-10_x000d_ | 0.0 |
| Lg 18-7-F3 HF 1uM | 0.0 |
| Lg 19-8-F5 HF 3uM | 0.0 |
| Lg 23-11-F2 HF 3uM | 0.0 |
| Blank 1 | 0.0 |
| Blank 2 | 0.0 |
| Blank 3 | 0.0 |
| Blank 4 | 0.0 |
| Blank 5 | 0.0 |MFI
### Chart: Mp-3
| Category | Mp39-64 IgG3 |
|---|---|
| Ng 7-13 1µM_x000d_ | 0.0 |
| Ng 7-13 3µM_x000d_ | 0.0 |
| Ng 9-9 1 µM_x000d_ | 0.0 |
| Ng 9-9 3µM_x000d_ | 0.0 |
| Ng 9-9 10µM_x000d_ | 0.0 |
| Ng 10-13 1 µM_x000d_ | 0.0 |
| Ng 10-13 3 µM_x000d_ | 0.0 |
| Ng 10-13 10 µM_x000d_ | 0.0 |
| Ng 10-13 30 µM_x000d_ | 0.0 |
| Ng 10-10 1 µM_x000d_ | 0.0 |
| Ng 10-10 3 µM_x000d_ | 0.0 |
| Ng 10-11 1 µM_x000d_ | 0.0 |
| Ng 10-11 3 µM_x000d_ | 25.333333333333343 |
| Ng 10-12 1 µM_x000d_ | 1.0 |
| Ng 11-10 1 µM_x000d_ | 0.0 |
| Ng 11-10 3 µM_x000d_ | 0.0 |
| Ng 11-11 1 µM_x000d_ | 0.0 |
| Ng 11-11 3µM_x000d_ | 0.0 |
| Ng 11-11 10µM_x000d_ | 0.0 |
| Ng 12-11 1 µM_x000d_ | 0.0 |
| Ng 12-11 3 µM_x000d_ | 0.0 |
| Ng 13-15 1 µM_x000d_ | 0.0 |
| Ng 13-15 3 µM_x000d_ | 0.0 |
| Ng 13-14_x000d_ | 0.0 |
| Ng 14-10 1 µM_x000d_ | 0.0 |
| Ng 14-10 3 µM_x000d_ | 0.0 |
| Ng 14-11 1 µM_x000d_ | 0.0 |
| Ng 14-12 1 µM_x000d_ | 0.0 |
| Ng 14-7 1 µM_x000d_ | 0.0 |
| Ng 15-14/15 1 µM_x000d_ | 0.0 |
| Ng 15-12 1µM_x000d_ | 141.66666666666669 |
| Ng 17-7 1 µM_x000d_ | 0.0 |
| Ng 17-7 3 µM_x000d_ | 0.0 |
| Ng 17-7 10 µM_x000d_ | 0.0 |
| Ng 19-9 1 µM_x000d_ | 0.0 |
| Ng 19-9 3 µM_x000d_ | 0.0 |
| Ng 19-9 10 µM_x000d_ | 0.0 |
| Ng 20-7 1 µM_x000d_ | 0.0 |
| Ng 20-7 3 µM_x000d_ | 0.0 |
| Ng 20-7 10 µM_x000d_ | 0.0 |
| Ng 21-8 1 µM_x000d_ | 0.0 |
| Ng 21-8 3 µM_x000d_ | 0.0 |
| Ng 21-8 10 µM_x000d_ | 0.0 |
| Ng 15-13 3µM_x000d_ | 1141.3333333333333 |
| Ng 16-12 1 µM_x000d_ | 0.0 |
| Ng 17-9 1 µM_x000d_ | 0.0 |
| Ng 17-9 3 µM_x000d_ | 0.0 |
| Ng 18-11 1 µM_x000d_ | 0.0 |
| Ng 18-11 3 µM_x000d_ | 0.0 |
| Ng 20-8 1 µM_x000d_ | 0.0 |
| Ng 20-9 1 µM_x000d_ | 0.0 |
| Ng 20-9 3 µM_x000d_ | 0.0 |
| Ng 21-9 1 µM_x000d_ | 0.0 |
| Ng 13-13 3 µM_x000d_ | 2.666666666666657 |
| Ng 14-8 1 µM_x000d_ | 0.0 |
| Ng 14-9 1 µM_x000d_ | 0.0 |
| Ng 14-9 3 µM_x000d_ | 0.0 |
| Ng 14-9 10 µM_x000d_ | 0.0 |
| Ng 14-9 30 µM_x000d_ | 0.0 |
| Ng 15-10 1µM_x000d_ | 0.0 |
| Ng 16-9 1 µM_x000d_ | 508.66666666666663 |
| Ng 17-8 1 µM_x000d_ | 0.0 |
| Ng 17-8 3 µM_x000d_ | 0.0 |
| Ng 18-9 1µM_x000d_ | 1362.0 |
| Ng 19-11 1 µM_x000d_ | 0.0 |
| Ng 19-11 3 µM_x000d_ | 0.0 |
| Lg 17-15 HF 10 µM_x000d_ | 961.0 |
| Lg 15-17 1 µM_x000d_ | 97.33333333333334 |
| Lg 17-15 1 µM_x000d_ | 1607.3333333333333 |
| Lg 17-15 3 µM_x000d_ | 2484.3333333333335 |
| Lg 17-15 10 µM_x000d_ | 4623.333333333333 |
| Lg 17-17 1 µM_x000d_ | 1329.6666666666667 |
| Lg 17-16 1 µM_x000d_ | 1612.3333333333333 |
| Lg 16-17 3 µM_x000d_ | 1736.6666666666667 |
| Lg 18-8 1 µM_x000d_ | 1654.6666666666667 |
| Lg 19-8 1 µM_x000d_ | 25.0 |
| Lg 19-8 3 µM_x000d_ | 964.3333333333333 |
| Lg 19-9 1 µM_x000d_ | 1661.3333333333333 |
| Lg 20-10_x000d_ | 829.3333333333334 |
| Lg 22-12 1 µM_x000d_ | 27.333333333333343 |
| Lg 20-9 1 µM_x000d_ | 1274.6666666666667 |
| Lg 23-11 1 µM_x000d_ | 1154.6666666666667 |
| Lg 23-11 3 µM_x000d_ | 2482.0 |
| Lg 23-12_x000d_ | 1324.6666666666667 |
| Lg 26-8_x000d_ | 1653.0 |
| Lg 26-9_x000d_ | 789.6666666666666 |
| Lg 22-13_x000d_ | 1771.6666666666667 |
| Lg 26-10_x000d_ | 0.0 |
| Lg 18-7-F3 HF 1uM | 0.0 |
| Lg 19-8-F5 HF 3uM | 0.0 |
| Lg 23-11-F2 HF 3uM | 0.0 |
| Blank 1 | 0.0 |
| Blank 2 | 0.0 |
| Blank 3 | 0.0 |
| Blank 4 | 0.0 |
| Blank 5 | 0.0 |MFI
### Chart: Mp-4
| Category | Mp 39-115 IgG3 |
|---|---|
| Ng 7-13 1µM_x000d_ | 21.666666666666657 |
| Ng 7-13 3µM_x000d_ | 383.5 |
| Ng 9-9 1 µM_x000d_ | 47.66666666666666 |
| Ng 9-9 3µM_x000d_ | 119.0 |
| Ng 9-9 10µM_x000d_ | 4.0 |
| Ng 10-13 1 µM_x000d_ | 3.0 |
| Ng 10-13 3 µM_x000d_ | 0.0 |
| Ng 10-13 10 µM_x000d_ | 6.666666666666657 |
| Ng 10-13 30 µM_x000d_ | 11.333333333333343 |
| Ng 10-10 1 µM_x000d_ | 0.0 |
| Ng 10-10 3 µM_x000d_ | 5.666666666666657 |
| Ng 10-11 1 µM_x000d_ | 0.0 |
| Ng 10-11 3 µM_x000d_ | 0.0 |
| Ng 10-12 1 µM_x000d_ | 0.0 |
| Ng 11-10 1 µM_x000d_ | 33.33333333333334 |
| Ng 11-10 3 µM_x000d_ | 0.0 |
| Ng 11-11 1 µM_x000d_ | 0.0 |
| Ng 11-11 3µM_x000d_ | 14.666666666666657 |
| Ng 11-11 10µM_x000d_ | 0.0 |
| Ng 12-11 1 µM_x000d_ | 0.0 |
| Ng 12-11 3 µM_x000d_ | 1.0 |
| Ng 13-15 1 µM_x000d_ | 0.0 |
| Ng 13-15 3 µM_x000d_ | 0.0 |
| Ng 13-14_x000d_ | 0.0 |
| Ng 14-10 1 µM_x000d_ | 0.0 |
| Ng 14-10 3 µM_x000d_ | 513.6666666666666 |
| Ng 14-11 1 µM_x000d_ | 0.0 |
| Ng 14-12 1 µM_x000d_ | 811.3333333333334 |
| Ng 14-7 1 µM_x000d_ | 0.0 |
| Ng 15-14/15 1 µM_x000d_ | 143.33333333333331 |
| Ng 15-12 1µM_x000d_ | 234.0 |
| Ng 17-7 1 µM_x000d_ | 0.0 |
| Ng 17-7 3 µM_x000d_ | 0.0 |
| Ng 17-7 10 µM_x000d_ | 0.0 |
| Ng 19-9 1 µM_x000d_ | 0.0 |
| Ng 19-9 3 µM_x000d_ | 0.0 |
| Ng 19-9 10 µM_x000d_ | 51.0 |
| Ng 20-7 1 µM_x000d_ | 0.0 |
| Ng 20-7 3 µM_x000d_ | 0.0 |
| Ng 20-7 10 µM_x000d_ | 0.0 |
| Ng 21-8 1 µM_x000d_ | 3.333333333333343 |
| Ng 21-8 3 µM_x000d_ | 0.0 |
| Ng 21-8 10 µM_x000d_ | 17.0 |
| Ng 15-13 3µM_x000d_ | 1573.3333333333333 |
| Ng 16-12 1 µM_x000d_ | 0.0 |
| Ng 17-9 1 µM_x000d_ | 0.0 |
| Ng 17-9 3 µM_x000d_ | 2.666666666666657 |
| Ng 18-11 1 µM_x000d_ | 0.0 |
| Ng 18-11 3 µM_x000d_ | 9.333333333333343 |
| Ng 20-8 1 µM_x000d_ | 0.0 |
| Ng 20-9 1 µM_x000d_ | 0.0 |
| Ng 20-9 3 µM_x000d_ | 5.0 |
| Ng 21-9 1 µM_x000d_ | 52.33333333333334 |
| Ng 13-13 3 µM_x000d_ | 0.0 |
| Ng 14-8 1 µM_x000d_ | 0.0 |
| Ng 14-9 1 µM_x000d_ | 0.0 |
| Ng 14-9 3 µM_x000d_ | 14.666666666666657 |
| Ng 14-9 10 µM_x000d_ | 27.0 |
| Ng 14-9 30 µM_x000d_ | 434.33333333333337 |
| Ng 15-10 1µM_x000d_ | 0.0 |
| Ng 16-9 1 µM_x000d_ | 36.66666666666666 |
| Ng 17-8 1 µM_x000d_ | 0.0 |
| Ng 17-8 3 µM_x000d_ | 0.0 |
| Ng 18-9 1µM_x000d_ | 1075.3333333333333 |
| Ng 19-11 1 µM_x000d_ | 3.333333333333343 |
| Ng 19-11 3 µM_x000d_ | 63.66666666666666 |
| Lg 17-15 HF 10 µM_x000d_ | 2453.6666666666665 |
| Lg 15-17 1 µM_x000d_ | 21.666666666666657 |
| Lg 17-15 1 µM_x000d_ | 6.666666666666657 |
| Lg 17-15 3 µM_x000d_ | 124.33333333333331 |
| Lg 17-15 10 µM_x000d_ | 465.0 |
| Lg 17-17 1 µM_x000d_ | 16.666666666666657 |
| Lg 17-16 1 µM_x000d_ | 27.0 |
| Lg 16-17 3 µM_x000d_ | 389.33333333333337 |
| Lg 18-8 1 µM_x000d_ | 0.0 |
| Lg 19-8 1 µM_x000d_ | 0.0 |
| Lg 19-8 3 µM_x000d_ | 0.0 |
| Lg 19-9 1 µM_x000d_ | 0.0 |
| Lg 20-10_x000d_ | 0.0 |
| Lg 22-12 1 µM_x000d_ | 0.0 |
| Lg 20-9 1 µM_x000d_ | 0.0 |
| Lg 23-11 1 µM_x000d_ | 0.0 |
| Lg 23-11 3 µM_x000d_ | 25.333333333333343 |
| Lg 23-12_x000d_ | 4.0 |
| Lg 26-8_x000d_ | 0.0 |
| Lg 26-9_x000d_ | 0.0 |
| Lg 22-13_x000d_ | 0.0 |
| Lg 26-10_x000d_ | 10.0 |
| Lg 18-7-F3 HF 1uM | 0.0 |
| Lg 19-8-F5 HF 3uM | 0.0 |
| Lg 23-11-F2 HF 3uM | 0.0 |
| Blank 1 | 0.0 |
| Blank 2 | 0.0 |
| Blank 3 | 0.0 |
| Blank 4 | 0.0 |
| Blank 5 | 0.0 |MFI
### Chart: Mp-5
| Category | Mp k-26 IgG3 |
|---|---|
| Ng 7-13 1µM_x000d_ | 0.0 |
| Ng 7-13 3µM_x000d_ | 0.0 |
| Ng 9-9 1 µM_x000d_ | 0.0 |
| Ng 9-9 3µM_x000d_ | 0.0 |
| Ng 9-9 10µM_x000d_ | 0.0 |
| Ng 10-13 1 µM_x000d_ | 0.0 |
| Ng 10-13 3 µM_x000d_ | 0.0 |
| Ng 10-13 10 µM_x000d_ | 0.0 |
| Ng 10-13 30 µM_x000d_ | 0.0 |
| Ng 10-10 1 µM_x000d_ | 0.0 |
| Ng 10-10 3 µM_x000d_ | 0.0 |
| Ng 10-11 1 µM_x000d_ | 0.0 |
| Ng 10-11 3 µM_x000d_ | 0.0 |
| Ng 10-12 1 µM_x000d_ | 0.0 |
| Ng 11-10 1 µM_x000d_ | 0.0 |
| Ng 11-10 3 µM_x000d_ | 0.0 |
| Ng 11-11 1 µM_x000d_ | 0.0 |
| Ng 11-11 3µM_x000d_ | 0.0 |
| Ng 11-11 10µM_x000d_ | 0.0 |
| Ng 12-11 1 µM_x000d_ | 0.0 |
| Ng 12-11 3 µM_x000d_ | 0.0 |
| Ng 13-15 1 µM_x000d_ | 0.0 |
| Ng 13-15 3 µM_x000d_ | 0.0 |
| Ng 13-14_x000d_ | 487.66666666666663 |
| Ng 14-10 1 µM_x000d_ | 1116.6666666666667 |
| Ng 14-10 3 µM_x000d_ | 3422.6666666666665 |
| Ng 14-11 1 µM_x000d_ | 134.66666666666669 |
| Ng 14-12 1 µM_x000d_ | 5794.0 |
| Ng 14-7 1 µM_x000d_ | 0.0 |
| Ng 15-14/15 1 µM_x000d_ | 1611.0 |
| Ng 15-12 1µM_x000d_ | 54.0 |
| Ng 17-7 1 µM_x000d_ | 0.0 |
| Ng 17-7 3 µM_x000d_ | 0.0 |
| Ng 17-7 10 µM_x000d_ | 0.0 |
| Ng 19-9 1 µM_x000d_ | 0.0 |
| Ng 19-9 3 µM_x000d_ | 0.0 |
| Ng 19-9 10 µM_x000d_ | 190.66666666666669 |
| Ng 20-7 1 µM_x000d_ | 0.0 |
| Ng 20-7 3 µM_x000d_ | 0.0 |
| Ng 20-7 10 µM_x000d_ | 67.33333333333334 |
| Ng 21-8 1 µM_x000d_ | 0.0 |
| Ng 21-8 3 µM_x000d_ | 0.0 |
| Ng 21-8 10 µM_x000d_ | 425.0 |
| Ng 15-13 3µM_x000d_ | 1483.6666666666667 |
| Ng 16-12 1 µM_x000d_ | 0.0 |
| Ng 17-9 1 µM_x000d_ | 9.0 |
| Ng 17-9 3 µM_x000d_ | 19.0 |
| Ng 18-11 1 µM_x000d_ | 67.66666666666666 |
| Ng 18-11 3 µM_x000d_ | 88.0 |
| Ng 20-8 1 µM_x000d_ | 46.33333333333334 |
| Ng 20-9 1 µM_x000d_ | 160.66666666666669 |
| Ng 20-9 3 µM_x000d_ | 614.6666666666666 |
| Ng 21-9 1 µM_x000d_ | 240.0 |
| Ng 13-13 3 µM_x000d_ | 0.0 |
| Ng 14-8 1 µM_x000d_ | 0.0 |
| Ng 14-9 1 µM_x000d_ | 0.0 |
| Ng 14-9 3 µM_x000d_ | 0.0 |
| Ng 14-9 10 µM_x000d_ | 0.0 |
| Ng 14-9 30 µM_x000d_ | 0.0 |
| Ng 15-10 1µM_x000d_ | 0.0 |
| Ng 16-9 1 µM_x000d_ | 21.666666666666657 |
| Ng 17-8 1 µM_x000d_ | 0.0 |
| Ng 17-8 3 µM_x000d_ | 0.0 |
| Ng 18-9 1µM_x000d_ | 1737.0 |
| Ng 19-11 1 µM_x000d_ | 1891.6666666666667 |
| Ng 19-11 3 µM_x000d_ | 4128.333333333333 |
| Lg 17-15 HF 10 µM_x000d_ | 4564.666666666667 |
| Lg 15-17 1 µM_x000d_ | 589.6666666666666 |
| Lg 17-15 1 µM_x000d_ | 236.33333333333331 |
| Lg 17-15 3 µM_x000d_ | 424.0 |
| Lg 17-15 10 µM_x000d_ | 2503.3333333333335 |
| Lg 17-17 1 µM_x000d_ | 235.66666666666669 |
| Lg 17-16 1 µM_x000d_ | 781.0 |
| Lg 16-17 3 µM_x000d_ | 774.6666666666666 |
| Lg 18-8 1 µM_x000d_ | 196.0 |
| Lg 19-8 1 µM_x000d_ | 1.6666666666666572 |
| Lg 19-8 3 µM_x000d_ | 1503.0 |
| Lg 19-9 1 µM_x000d_ | 450.66666666666663 |
| Lg 20-10_x000d_ | 80.66666666666666 |
| Lg 22-12 1 µM_x000d_ | 0.0 |
| Lg 20-9 1 µM_x000d_ | 21.333333333333343 |
| Lg 23-11 1 µM_x000d_ | 13.333333333333343 |
| Lg 23-11 3 µM_x000d_ | 744.0 |
| Lg 23-12_x000d_ | 377.0 |
| Lg 26-8_x000d_ | 24.0 |
| Lg 26-9_x000d_ | 108.33333333333331 |
| Lg 22-13_x000d_ | 18.666666666666657 |
| Lg 26-10_x000d_ | 0.0 |
| Lg 18-7-F3 HF 1uM | 0.0 |
| Lg 19-8-F5 HF 3uM | 0.0 |
| Lg 23-11-F2 HF 3uM | 0.0 |
| Blank 1 | 0.0 |
| Blank 2 | 0.0 |
| Blank 3 | 0.0 |
| Blank 4 | 0.0 |
| Blank 5 | 0.0 |MFI
### Chart: Mp-6
| Category | Mp L23 IgG3 |
|---|---|
| Ng1_1uM
 | 4.0 |
| Ng1_3uM
 | 428.33333333333337 |
| Ng2_1uM | 0.0 |
| Ng2_3uM | 0.0 |
| Ng2_10uM | 0.0 |
| Ng3_1uM | 0.0 |
| Ng3_3uM | 0.0 |
| Ng3_10uM | 0.0 |
| Ng3_30uM | 0.0 |
| Ng4_1uM | 0.0 |
| Ng4_3uM | 0.0 |
| Ng5_1uM | 0.0 |
| Ng5_3uM | 0.0 |
| Ng6_1uM | 0.0 |
| Ng7_1uM | 0.0 |
| Ng7_3uM | 0.0 |
| Ng8_1uM | 0.0 |
| Ng8_3uM | 0.0 |
| Ng8_10uM | 0.0 |
| Ng9_1uM | 0.0 |
| Ng9_3uM | 0.0 |
| Ng10_3uM | 0.0 |
| Ng10_1uM | 0.0 |
| Ng11 | 0.0 |
| Ng12_1uM | 0.0 |
| Ng12_3uM | 5.0 |
| Ng13_1uM | 0.0 |
| Ng14_1uM | 96.0 |
| Ng15_1uM | 0.0 |
| Ng16_1uM | 3.666666666666657 |
| Ng17_1uM | 135.66666666666669 |
| Ng18_1uM | 0.0 |
| Ng18_3uM | 0.0 |
| Ng18_10uM | 59.66666666666666 |
| Ng19_1uM | 0.0 |
| Ng19_3uM | 172.66666666666669 |
| Ng19_10uM | 83.0 |
| Ng20_1uM | 0.0 |
| Ng20_3uM | 79.33333333333334 |
| Ng20_10uM | 1310.0 |
| Ng21_1uM | 44.66666666666666 |
| Ng21 _3uM | 254.0 |
| Ng21_10uM | 2375.3333333333335 |
| Ng22_1uM | 1254.3333333333333 |
| Ng23_1uM | 0.0 |
| Ng24_1uM | 60.0 |
| Ng24_3uM | 315.3333333333333 |
| Ng25_1uM | 667.6666666666666 |
| Ng25_3uM | 843.3333333333334 |
| Ng26_1uM | 1005.6666666666667 |
| Ng27_1uM | 428.33333333333337 |
| Ng27_3uM | 1591.3333333333333 |
| Ng28_1uM | 561.0 |
| Ng29_1uM | 0.0 |
| Ng30_1uM | 0.0 |
| Ng31_1uM | 0.0 |
| Ng31_3uM | 0.0 |
| Ng31_10uM | 0.0 |
| Ng31_30uM | 0.0 |
| Ng32_1uM | 31.0 |
| Ng33_1uM | 130.0 |
| Ng34_1uM | 7.0 |
| Ng34_3uM | 772.3333333333334 |
| Ng35_1uM | 764.3333333333334 |
| Ng36_1uM | 1067.0 |
| Ng36_3uM | 1152.0 |
| Lg2_HF_10uM | 59696.333333333336 |
| Lg1_1uM | 1311.3333333333333 |
| Lg2_1uM | 23403.333333333332 |
| Lg2_3uM | 8215.0 |
| Lg2_10uM | 64938.0 |
| Lg3_1uM | 14279.666666666666 |
| Lg4_1uM | 12725.0 |
| Lg5_3uM | 2930.6666666666665 |
| Lg6_1uM | 16918.333333333332 |
| Lg7_1uM | 862.3333333333334 |
| Lg7_3uM | 7.0 |
| Lg8_1uM | 6591.333333333333 |
| Lg9 | 1002.3333333333333 |
| Lg10_1uM | 124.66666666666669 |
| Lg11_1uM | 15.666666666666657 |
| Lg12_1uM | 631.3333333333334 |
| Lg12_3uM | 1088.3333333333333 |
| Lg13 | 3829.3333333333335 |
| Lg14 | 601.6666666666666 |
| Lg15 | 234.33333333333331 |
| Lg16 | 1928.0 |
| Lg17 | 189.0 |
| Lg18_HF_1uM | 5.333333333333343 |
| Lg7_HF_3uM | 261.3333333333333 |
| Lg12_HF_3uM | 0.0 |
| Blank 1 | 0.0 |
| Blank 2 | 0.0 |
| Blank 3 | 0.0 |
| Blank 4 | 0.0 |
| Blank 5 | 0.0 |MFI

## Slide 32
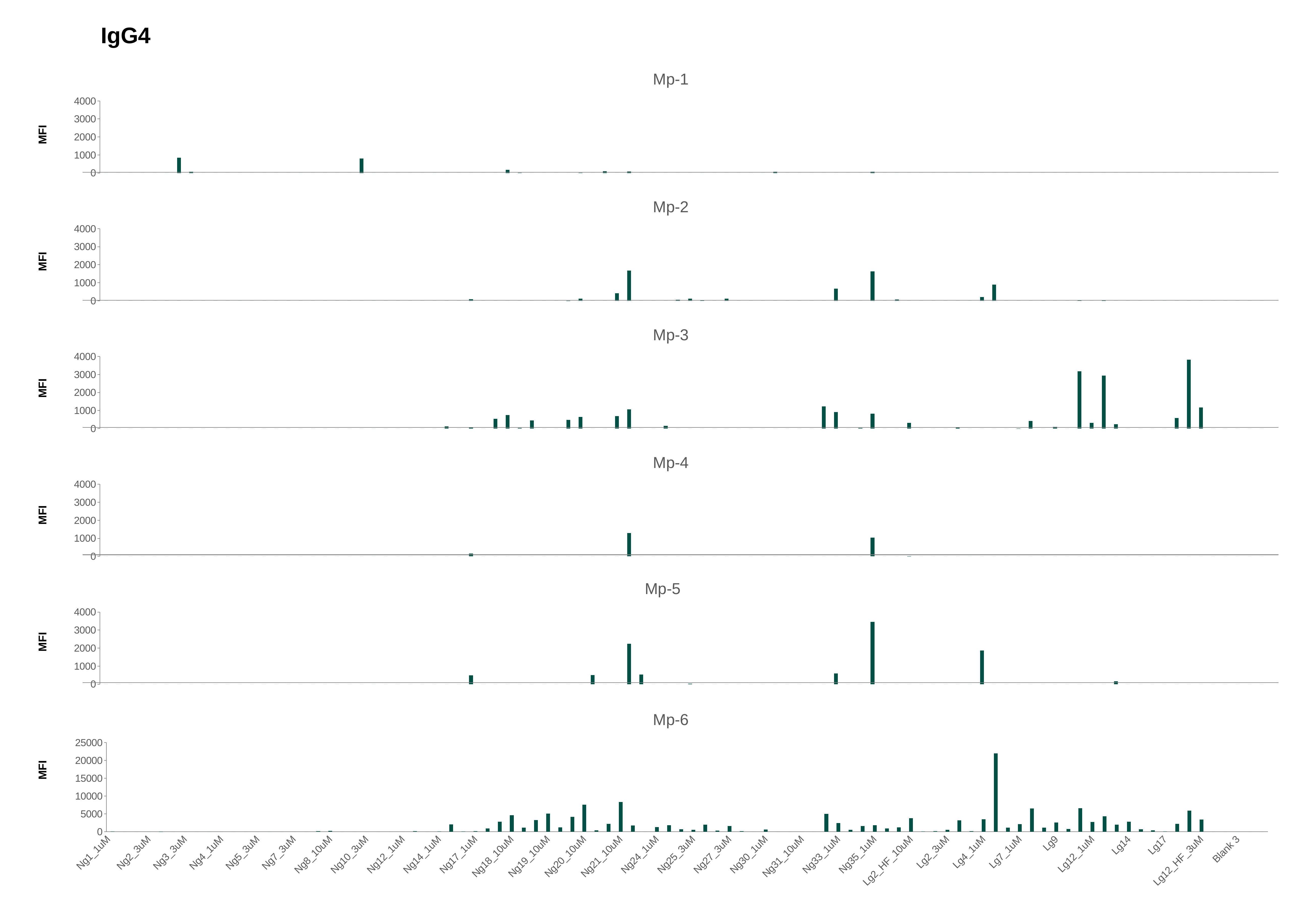

IgG4
### Chart: Mp-1
| Category | Mp 36-51 IgG4 |
|---|---|
| Ng 7-13 1µM_x000d_ | 0.0 |
| Ng 7-13 3µM_x000d_ | 0.0 |
| Ng 9-9 1 µM_x000d_ | 0.0 |
| Ng 9-9 3µM_x000d_ | 0.0 |
| Ng 9-9 10µM_x000d_ | 0.0 |
| Ng 10-13 1 µM_x000d_ | 0.0 |
| Ng 10-13 3 µM_x000d_ | 856.0 |
| Ng 10-13 10 µM_x000d_ | 69.0 |
| Ng 10-13 30 µM_x000d_ | 0.0 |
| Ng 10-10 1 µM_x000d_ | 0.0 |
| Ng 10-10 3 µM_x000d_ | 0.0 |
| Ng 10-11 1 µM_x000d_ | 0.0 |
| Ng 10-11 3 µM_x000d_ | 0.0 |
| Ng 10-12 1 µM_x000d_ | 0.0 |
| Ng 11-10 1 µM_x000d_ | 0.0 |
| Ng 11-10 3 µM_x000d_ | 0.0 |
| Ng 11-11 1 µM_x000d_ | 0.0 |
| Ng 11-11 3µM_x000d_ | 0.0 |
| Ng 11-11 10µM_x000d_ | 0.0 |
| Ng 12-11 1 µM_x000d_ | 0.0 |
| Ng 12-11 3 µM_x000d_ | 0.0 |
| Ng 13-15 1 µM_x000d_ | 806.6666666666666 |
| Ng 13-15 3 µM_x000d_ | 0.0 |
| Ng 13-14_x000d_ | 0.0 |
| Ng 14-10 1 µM_x000d_ | 0.0 |
| Ng 14-10 3 µM_x000d_ | 0.0 |
| Ng 14-11 1 µM_x000d_ | 0.0 |
| Ng 14-12 1 µM_x000d_ | 0.0 |
| Ng 14-7 1 µM_x000d_ | 0.0 |
| Ng 15-14/15 1 µM_x000d_ | 0.0 |
| Ng 15-12 1µM_x000d_ | 0.0 |
| Ng 17-7 1 µM_x000d_ | 0.0 |
| Ng 17-7 3 µM_x000d_ | 0.0 |
| Ng 17-7 10 µM_x000d_ | 168.66666666666669 |
| Ng 19-9 1 µM_x000d_ | 10.0 |
| Ng 19-9 3 µM_x000d_ | 0.0 |
| Ng 19-9 10 µM_x000d_ | 0.0 |
| Ng 20-7 1 µM_x000d_ | 0.0 |
| Ng 20-7 3 µM_x000d_ | 0.0 |
| Ng 20-7 10 µM_x000d_ | 5.666666666666657 |
| Ng 21-8 1 µM_x000d_ | 0.0 |
| Ng 21-8 3 µM_x000d_ | 102.0 |
| Ng 21-8 10 µM_x000d_ | 0.0 |
| Ng 15-13 3µM_x000d_ | 84.33333333333334 |
| Ng 16-12 1 µM_x000d_ | 0.0 |
| Ng 17-9 1 µM_x000d_ | 0.0 |
| Ng 17-9 3 µM_x000d_ | 0.0 |
| Ng 18-11 1 µM_x000d_ | 0.0 |
| Ng 18-11 3 µM_x000d_ | 0.0 |
| Ng 20-8 1 µM_x000d_ | 0.0 |
| Ng 20-9 1 µM_x000d_ | 0.0 |
| Ng 20-9 3 µM_x000d_ | 0.0 |
| Ng 21-9 1 µM_x000d_ | 0.0 |
| Ng 13-13 3 µM_x000d_ | 0.0 |
| Ng 14-8 1 µM_x000d_ | 0.0 |
| Ng 14-9 1 µM_x000d_ | 63.0 |
| Ng 14-9 3 µM_x000d_ | 0.0 |
| Ng 14-9 10 µM_x000d_ | 0.0 |
| Ng 14-9 30 µM_x000d_ | 0.0 |
| Ng 15-10 1µM_x000d_ | 0.0 |
| Ng 16-9 1 µM_x000d_ | 0.0 |
| Ng 17-8 1 µM_x000d_ | 0.0 |
| Ng 17-8 3 µM_x000d_ | 0.0 |
| Ng 18-9 1µM_x000d_ | 74.66666666666666 |
| Ng 19-11 1 µM_x000d_ | 0.0 |
| Ng 19-11 3 µM_x000d_ | 0.0 |
| Lg 17-15 HF 10 µM_x000d_ | 0.0 |
| Lg 15-17 1 µM_x000d_ | 0.0 |
| Lg 17-15 1 µM_x000d_ | 0.0 |
| Lg 17-15 3 µM_x000d_ | 0.0 |
| Lg 17-15 10 µM_x000d_ | 0.0 |
| Lg 17-17 1 µM_x000d_ | 0.0 |
| Lg 17-16 1 µM_x000d_ | 0.0 |
| Lg 16-17 3 µM_x000d_ | 0.0 |
| Lg 18-8 1 µM_x000d_ | 0.0 |
| Lg 19-8 1 µM_x000d_ | 0.0 |
| Lg 19-8 3 µM_x000d_ | 0.0 |
| Lg 19-9 1 µM_x000d_ | 0.0 |
| Lg 20-10_x000d_ | 0.0 |
| Lg 22-12 1 µM_x000d_ | 0.0 |
| Lg 20-9 1 µM_x000d_ | 0.0 |
| Lg 23-11 1 µM_x000d_ | 0.0 |
| Lg 23-11 3 µM_x000d_ | 0.0 |
| Lg 23-12_x000d_ | 0.0 |
| Lg 26-8_x000d_ | 0.0 |
| Lg 26-9_x000d_ | 0.0 |
| Lg 22-13_x000d_ | 0.0 |
| Lg 26-10_x000d_ | 0.0 |
| Lg 18-7-F3 HF 1uM | 0.0 |
| Lg 19-8-F5 HF 3uM | 0.0 |
| Lg 23-11-F2 HF 3uM | 0.0 |
| Blank 1 | 0.0 |
| Blank 2 | 0.0 |
| Blank 3 | 0.0 |
| Blank 4 | 0.0 |
| Blank 5 | 0.0 |MFI
### Chart: Mp-2
| Category | Mp 38-202 IgG4 |
|---|---|
| Ng 7-13 1µM_x000d_ | 0.0 |
| Ng 7-13 3µM_x000d_ | 0.0 |
| Ng 9-9 1 µM_x000d_ | 0.0 |
| Ng 9-9 3µM_x000d_ | 0.0 |
| Ng 9-9 10µM_x000d_ | 0.0 |
| Ng 10-13 1 µM_x000d_ | 0.0 |
| Ng 10-13 3 µM_x000d_ | 0.0 |
| Ng 10-13 10 µM_x000d_ | 0.0 |
| Ng 10-13 30 µM_x000d_ | 0.0 |
| Ng 10-10 1 µM_x000d_ | 0.0 |
| Ng 10-10 3 µM_x000d_ | 0.0 |
| Ng 10-11 1 µM_x000d_ | 0.0 |
| Ng 10-11 3 µM_x000d_ | 0.0 |
| Ng 10-12 1 µM_x000d_ | 0.0 |
| Ng 11-10 1 µM_x000d_ | 0.0 |
| Ng 11-10 3 µM_x000d_ | 0.0 |
| Ng 11-11 1 µM_x000d_ | 0.0 |
| Ng 11-11 3µM_x000d_ | 0.0 |
| Ng 11-11 10µM_x000d_ | 0.0 |
| Ng 12-11 1 µM_x000d_ | 0.0 |
| Ng 12-11 3 µM_x000d_ | 0.0 |
| Ng 13-15 1 µM_x000d_ | 0.0 |
| Ng 13-15 3 µM_x000d_ | 0.0 |
| Ng 13-14_x000d_ | 0.0 |
| Ng 14-10 1 µM_x000d_ | 0.0 |
| Ng 14-10 3 µM_x000d_ | 0.0 |
| Ng 14-11 1 µM_x000d_ | 0.0 |
| Ng 14-12 1 µM_x000d_ | 0.0 |
| Ng 14-7 1 µM_x000d_ | 0.0 |
| Ng 15-14/15 1 µM_x000d_ | 0.0 |
| Ng 15-12 1µM_x000d_ | 91.33333333333334 |
| Ng 17-7 1 µM_x000d_ | 0.0 |
| Ng 17-7 3 µM_x000d_ | 0.0 |
| Ng 17-7 10 µM_x000d_ | 0.0 |
| Ng 19-9 1 µM_x000d_ | 0.0 |
| Ng 19-9 3 µM_x000d_ | 0.0 |
| Ng 19-9 10 µM_x000d_ | 0.0 |
| Ng 20-7 1 µM_x000d_ | 0.0 |
| Ng 20-7 3 µM_x000d_ | 2.0 |
| Ng 20-7 10 µM_x000d_ | 124.66666666666669 |
| Ng 21-8 1 µM_x000d_ | 0.0 |
| Ng 21-8 3 µM_x000d_ | 0.0 |
| Ng 21-8 10 µM_x000d_ | 413.66666666666663 |
| Ng 15-13 3µM_x000d_ | 1681.0 |
| Ng 16-12 1 µM_x000d_ | 0.0 |
| Ng 17-9 1 µM_x000d_ | 0.0 |
| Ng 17-9 3 µM_x000d_ | 0.0 |
| Ng 18-11 1 µM_x000d_ | 64.0 |
| Ng 18-11 3 µM_x000d_ | 124.66666666666669 |
| Ng 20-8 1 µM_x000d_ | 27.666666666666657 |
| Ng 20-9 1 µM_x000d_ | 0.0 |
| Ng 20-9 3 µM_x000d_ | 118.66666666666669 |
| Ng 21-9 1 µM_x000d_ | 0.0 |
| Ng 13-13 3 µM_x000d_ | 0.0 |
| Ng 14-8 1 µM_x000d_ | 0.0 |
| Ng 14-9 1 µM_x000d_ | 0.0 |
| Ng 14-9 3 µM_x000d_ | 0.0 |
| Ng 14-9 10 µM_x000d_ | 0.0 |
| Ng 14-9 30 µM_x000d_ | 0.0 |
| Ng 15-10 1µM_x000d_ | 0.0 |
| Ng 16-9 1 µM_x000d_ | 671.3333333333334 |
| Ng 17-8 1 µM_x000d_ | 0.0 |
| Ng 17-8 3 µM_x000d_ | 0.0 |
| Ng 18-9 1µM_x000d_ | 1629.0 |
| Ng 19-11 1 µM_x000d_ | 0.0 |
| Ng 19-11 3 µM_x000d_ | 66.33333333333334 |
| Lg 17-15 HF 10 µM_x000d_ | 0.0 |
| Lg 15-17 1 µM_x000d_ | 0.0 |
| Lg 17-15 1 µM_x000d_ | 0.0 |
| Lg 17-15 3 µM_x000d_ | 0.0 |
| Lg 17-15 10 µM_x000d_ | 0.0 |
| Lg 17-17 1 µM_x000d_ | 0.0 |
| Lg 17-16 1 µM_x000d_ | 209.0 |
| Lg 16-17 3 µM_x000d_ | 898.6666666666667 |
| Lg 18-8 1 µM_x000d_ | 0.0 |
| Lg 19-8 1 µM_x000d_ | 0.0 |
| Lg 19-8 3 µM_x000d_ | 0.0 |
| Lg 19-9 1 µM_x000d_ | 0.0 |
| Lg 20-10_x000d_ | 0.0 |
| Lg 22-12 1 µM_x000d_ | 0.0 |
| Lg 20-9 1 µM_x000d_ | 18.333333333333343 |
| Lg 23-11 1 µM_x000d_ | 0.0 |
| Lg 23-11 3 µM_x000d_ | 31.333333333333343 |
| Lg 23-12_x000d_ | 0.0 |
| Lg 26-8_x000d_ | 0.0 |
| Lg 26-9_x000d_ | 0.0 |
| Lg 22-13_x000d_ | 0.0 |
| Lg 26-10_x000d_ | 0.0 |
| Lg 18-7-F3 HF 1uM | 0.0 |
| Lg 19-8-F5 HF 3uM | 0.0 |
| Lg 23-11-F2 HF 3uM | 0.0 |
| Blank 1 | 0.0 |
| Blank 2 | 0.0 |
| Blank 3 | 0.0 |
| Blank 4 | 0.0 |
| Blank 5 | 0.0 |MFI
### Chart: Mp-3
| Category | Mp39-64 IgG4 |
|---|---|
| Ng 7-13 1µM_x000d_ | 0.0 |
| Ng 7-13 3µM_x000d_ | 0.0 |
| Ng 9-9 1 µM_x000d_ | 0.0 |
| Ng 9-9 3µM_x000d_ | 0.0 |
| Ng 9-9 10µM_x000d_ | 0.0 |
| Ng 10-13 1 µM_x000d_ | 0.0 |
| Ng 10-13 3 µM_x000d_ | 0.0 |
| Ng 10-13 10 µM_x000d_ | 0.0 |
| Ng 10-13 30 µM_x000d_ | 0.0 |
| Ng 10-10 1 µM_x000d_ | 0.0 |
| Ng 10-10 3 µM_x000d_ | 0.0 |
| Ng 10-11 1 µM_x000d_ | 0.0 |
| Ng 10-11 3 µM_x000d_ | 0.0 |
| Ng 10-12 1 µM_x000d_ | 0.0 |
| Ng 11-10 1 µM_x000d_ | 0.0 |
| Ng 11-10 3 µM_x000d_ | 0.0 |
| Ng 11-11 1 µM_x000d_ | 0.0 |
| Ng 11-11 3µM_x000d_ | 0.0 |
| Ng 11-11 10µM_x000d_ | 0.0 |
| Ng 12-11 1 µM_x000d_ | 0.0 |
| Ng 12-11 3 µM_x000d_ | 0.0 |
| Ng 13-15 1 µM_x000d_ | 0.0 |
| Ng 13-15 3 µM_x000d_ | 0.0 |
| Ng 13-14_x000d_ | 0.0 |
| Ng 14-10 1 µM_x000d_ | 0.0 |
| Ng 14-10 3 µM_x000d_ | 0.0 |
| Ng 14-11 1 µM_x000d_ | 0.0 |
| Ng 14-12 1 µM_x000d_ | 0.0 |
| Ng 14-7 1 µM_x000d_ | 126.66666666666669 |
| Ng 15-14/15 1 µM_x000d_ | 0.0 |
| Ng 15-12 1µM_x000d_ | 69.33333333333334 |
| Ng 17-7 1 µM_x000d_ | 0.0 |
| Ng 17-7 3 µM_x000d_ | 541.0 |
| Ng 17-7 10 µM_x000d_ | 749.0 |
| Ng 19-9 1 µM_x000d_ | 29.0 |
| Ng 19-9 3 µM_x000d_ | 458.0 |
| Ng 19-9 10 µM_x000d_ | 0.0 |
| Ng 20-7 1 µM_x000d_ | 0.0 |
| Ng 20-7 3 µM_x000d_ | 474.33333333333337 |
| Ng 20-7 10 µM_x000d_ | 640.3333333333334 |
| Ng 21-8 1 µM_x000d_ | 0.0 |
| Ng 21-8 3 µM_x000d_ | 0.0 |
| Ng 21-8 10 µM_x000d_ | 693.6666666666666 |
| Ng 15-13 3µM_x000d_ | 1061.6666666666667 |
| Ng 16-12 1 µM_x000d_ | 0.0 |
| Ng 17-9 1 µM_x000d_ | 0.0 |
| Ng 17-9 3 µM_x000d_ | 144.33333333333331 |
| Ng 18-11 1 µM_x000d_ | 0.0 |
| Ng 18-11 3 µM_x000d_ | 0.0 |
| Ng 20-8 1 µM_x000d_ | 0.0 |
| Ng 20-9 1 µM_x000d_ | 0.0 |
| Ng 20-9 3 µM_x000d_ | 0.0 |
| Ng 21-9 1 µM_x000d_ | 0.0 |
| Ng 13-13 3 µM_x000d_ | 0.0 |
| Ng 14-8 1 µM_x000d_ | 0.0 |
| Ng 14-9 1 µM_x000d_ | 0.0 |
| Ng 14-9 3 µM_x000d_ | 0.0 |
| Ng 14-9 10 µM_x000d_ | 0.0 |
| Ng 14-9 30 µM_x000d_ | 0.0 |
| Ng 15-10 1µM_x000d_ | 1235.6666666666667 |
| Ng 16-9 1 µM_x000d_ | 918.0 |
| Ng 17-8 1 µM_x000d_ | 0.0 |
| Ng 17-8 3 µM_x000d_ | 24.0 |
| Ng 18-9 1µM_x000d_ | 827.3333333333334 |
| Ng 19-11 1 µM_x000d_ | 0.0 |
| Ng 19-11 3 µM_x000d_ | 0.0 |
| Lg 17-15 HF 10 µM_x000d_ | 308.0 |
| Lg 15-17 1 µM_x000d_ | 0.0 |
| Lg 17-15 1 µM_x000d_ | 0.0 |
| Lg 17-15 3 µM_x000d_ | 0.0 |
| Lg 17-15 10 µM_x000d_ | 77.66666666666666 |
| Lg 17-17 1 µM_x000d_ | 0.0 |
| Lg 17-16 1 µM_x000d_ | 0.0 |
| Lg 16-17 3 µM_x000d_ | 0.0 |
| Lg 18-8 1 µM_x000d_ | 0.0 |
| Lg 19-8 1 µM_x000d_ | 5.666666666666657 |
| Lg 19-8 3 µM_x000d_ | 421.0 |
| Lg 19-9 1 µM_x000d_ | 0.0 |
| Lg 20-10_x000d_ | 93.0 |
| Lg 22-12 1 µM_x000d_ | 0.0 |
| Lg 20-9 1 µM_x000d_ | 3182.0 |
| Lg 23-11 1 µM_x000d_ | 321.6666666666667 |
| Lg 23-11 3 µM_x000d_ | 2937.3333333333335 |
| Lg 23-12_x000d_ | 239.66666666666669 |
| Lg 26-8_x000d_ | 0.0 |
| Lg 26-9_x000d_ | 0.0 |
| Lg 22-13_x000d_ | 0.0 |
| Lg 26-10_x000d_ | 0.0 |
| Lg 18-7-F3 HF 1uM | 580.6666666666666 |
| Lg 19-8-F5 HF 3uM | 3816.3333333333335 |
| Lg 23-11-F2 HF 3uM | 1174.0 |
| Blank 1 | 0.0 |
| Blank 2 | 0.0 |
| Blank 3 | 0.0 |
| Blank 4 | 0.0 |
| Blank 5 | 0.0 |MFI
### Chart: Mp-4
| Category | Mp 39-115 IgG4 |
|---|---|
| Ng 7-13 1µM_x000d_ | 0.0 |
| Ng 7-13 3µM_x000d_ | 0.0 |
| Ng 9-9 1 µM_x000d_ | 0.0 |
| Ng 9-9 3µM_x000d_ | 0.0 |
| Ng 9-9 10µM_x000d_ | 0.0 |
| Ng 10-13 1 µM_x000d_ | 0.0 |
| Ng 10-13 3 µM_x000d_ | 0.0 |
| Ng 10-13 10 µM_x000d_ | 0.0 |
| Ng 10-13 30 µM_x000d_ | 0.0 |
| Ng 10-10 1 µM_x000d_ | 0.0 |
| Ng 10-10 3 µM_x000d_ | 0.0 |
| Ng 10-11 1 µM_x000d_ | 0.0 |
| Ng 10-11 3 µM_x000d_ | 0.0 |
| Ng 10-12 1 µM_x000d_ | 0.0 |
| Ng 11-10 1 µM_x000d_ | 0.0 |
| Ng 11-10 3 µM_x000d_ | 0.0 |
| Ng 11-11 1 µM_x000d_ | 0.0 |
| Ng 11-11 3µM_x000d_ | 0.0 |
| Ng 11-11 10µM_x000d_ | 0.0 |
| Ng 12-11 1 µM_x000d_ | 0.0 |
| Ng 12-11 3 µM_x000d_ | 0.0 |
| Ng 13-15 1 µM_x000d_ | 0.0 |
| Ng 13-15 3 µM_x000d_ | 0.0 |
| Ng 13-14_x000d_ | 0.0 |
| Ng 14-10 1 µM_x000d_ | 0.0 |
| Ng 14-10 3 µM_x000d_ | 0.0 |
| Ng 14-11 1 µM_x000d_ | 0.0 |
| Ng 14-12 1 µM_x000d_ | 0.0 |
| Ng 14-7 1 µM_x000d_ | 0.0 |
| Ng 15-14/15 1 µM_x000d_ | 0.0 |
| Ng 15-12 1µM_x000d_ | 160.33333333333331 |
| Ng 17-7 1 µM_x000d_ | 0.0 |
| Ng 17-7 3 µM_x000d_ | 0.0 |
| Ng 17-7 10 µM_x000d_ | 0.0 |
| Ng 19-9 1 µM_x000d_ | 0.0 |
| Ng 19-9 3 µM_x000d_ | 0.0 |
| Ng 19-9 10 µM_x000d_ | 0.0 |
| Ng 20-7 1 µM_x000d_ | 0.0 |
| Ng 20-7 3 µM_x000d_ | 0.0 |
| Ng 20-7 10 µM_x000d_ | 0.0 |
| Ng 21-8 1 µM_x000d_ | 0.0 |
| Ng 21-8 3 µM_x000d_ | 0.0 |
| Ng 21-8 10 µM_x000d_ | 0.0 |
| Ng 15-13 3µM_x000d_ | 1296.6666666666667 |
| Ng 16-12 1 µM_x000d_ | 0.0 |
| Ng 17-9 1 µM_x000d_ | 0.0 |
| Ng 17-9 3 µM_x000d_ | 0.0 |
| Ng 18-11 1 µM_x000d_ | 0.0 |
| Ng 18-11 3 µM_x000d_ | 0.0 |
| Ng 20-8 1 µM_x000d_ | 0.0 |
| Ng 20-9 1 µM_x000d_ | 0.0 |
| Ng 20-9 3 µM_x000d_ | 0.0 |
| Ng 21-9 1 µM_x000d_ | 0.0 |
| Ng 13-13 3 µM_x000d_ | 0.0 |
| Ng 14-8 1 µM_x000d_ | 0.0 |
| Ng 14-9 1 µM_x000d_ | 0.0 |
| Ng 14-9 3 µM_x000d_ | 0.0 |
| Ng 14-9 10 µM_x000d_ | 0.0 |
| Ng 14-9 30 µM_x000d_ | 0.0 |
| Ng 15-10 1µM_x000d_ | 0.0 |
| Ng 16-9 1 µM_x000d_ | 0.0 |
| Ng 17-8 1 µM_x000d_ | 0.0 |
| Ng 17-8 3 µM_x000d_ | 0.0 |
| Ng 18-9 1µM_x000d_ | 1042.6666666666667 |
| Ng 19-11 1 µM_x000d_ | 0.0 |
| Ng 19-11 3 µM_x000d_ | 0.0 |
| Lg 17-15 HF 10 µM_x000d_ | 7.666666666666657 |
| Lg 15-17 1 µM_x000d_ | 0.0 |
| Lg 17-15 1 µM_x000d_ | 0.0 |
| Lg 17-15 3 µM_x000d_ | 0.0 |
| Lg 17-15 10 µM_x000d_ | 0.0 |
| Lg 17-17 1 µM_x000d_ | 0.0 |
| Lg 17-16 1 µM_x000d_ | 0.0 |
| Lg 16-17 3 µM_x000d_ | 0.0 |
| Lg 18-8 1 µM_x000d_ | 0.0 |
| Lg 19-8 1 µM_x000d_ | 0.0 |
| Lg 19-8 3 µM_x000d_ | 0.0 |
| Lg 19-9 1 µM_x000d_ | 0.0 |
| Lg 20-10_x000d_ | 0.0 |
| Lg 22-12 1 µM_x000d_ | 0.0 |
| Lg 20-9 1 µM_x000d_ | 0.0 |
| Lg 23-11 1 µM_x000d_ | 0.0 |
| Lg 23-11 3 µM_x000d_ | 0.0 |
| Lg 23-12_x000d_ | 0.0 |
| Lg 26-8_x000d_ | 0.0 |
| Lg 26-9_x000d_ | 0.0 |
| Lg 22-13_x000d_ | 0.0 |
| Lg 26-10_x000d_ | 0.0 |
| Lg 18-7-F3 HF 1uM | 0.0 |
| Lg 19-8-F5 HF 3uM | 0.0 |
| Lg 23-11-F2 HF 3uM | 0.0 |
| Blank 1 | 0.0 |
| Blank 2 | 0.0 |
| Blank 3 | 0.0 |
| Blank 4 | 0.0 |
| Blank 5 | 0.0 |MFI
### Chart: Mp-5
| Category | Mp k-26 IgG4 |
|---|---|
| Ng 7-13 1µM_x000d_ | 0.0 |
| Ng 7-13 3µM_x000d_ | 0.0 |
| Ng 9-9 1 µM_x000d_ | 0.0 |
| Ng 9-9 3µM_x000d_ | 0.0 |
| Ng 9-9 10µM_x000d_ | 0.0 |
| Ng 10-13 1 µM_x000d_ | 0.0 |
| Ng 10-13 3 µM_x000d_ | 0.0 |
| Ng 10-13 10 µM_x000d_ | 0.0 |
| Ng 10-13 30 µM_x000d_ | 0.0 |
| Ng 10-10 1 µM_x000d_ | 0.0 |
| Ng 10-10 3 µM_x000d_ | 0.0 |
| Ng 10-11 1 µM_x000d_ | 0.0 |
| Ng 10-11 3 µM_x000d_ | 0.0 |
| Ng 10-12 1 µM_x000d_ | 0.0 |
| Ng 11-10 1 µM_x000d_ | 0.0 |
| Ng 11-10 3 µM_x000d_ | 0.0 |
| Ng 11-11 1 µM_x000d_ | 0.0 |
| Ng 11-11 3µM_x000d_ | 0.0 |
| Ng 11-11 10µM_x000d_ | 0.0 |
| Ng 12-11 1 µM_x000d_ | 0.0 |
| Ng 12-11 3 µM_x000d_ | 0.0 |
| Ng 13-15 1 µM_x000d_ | 0.0 |
| Ng 13-15 3 µM_x000d_ | 0.0 |
| Ng 13-14_x000d_ | 0.0 |
| Ng 14-10 1 µM_x000d_ | 0.0 |
| Ng 14-10 3 µM_x000d_ | 0.0 |
| Ng 14-11 1 µM_x000d_ | 0.0 |
| Ng 14-12 1 µM_x000d_ | 0.0 |
| Ng 14-7 1 µM_x000d_ | 0.0 |
| Ng 15-14/15 1 µM_x000d_ | 0.0 |
| Ng 15-12 1µM_x000d_ | 490.33333333333337 |
| Ng 17-7 1 µM_x000d_ | 0.0 |
| Ng 17-7 3 µM_x000d_ | 0.0 |
| Ng 17-7 10 µM_x000d_ | 0.0 |
| Ng 19-9 1 µM_x000d_ | 0.0 |
| Ng 19-9 3 µM_x000d_ | 0.0 |
| Ng 19-9 10 µM_x000d_ | 0.0 |
| Ng 20-7 1 µM_x000d_ | 0.0 |
| Ng 20-7 3 µM_x000d_ | 0.0 |
| Ng 20-7 10 µM_x000d_ | 0.0 |
| Ng 21-8 1 µM_x000d_ | 505.0 |
| Ng 21-8 3 µM_x000d_ | 0.0 |
| Ng 21-8 10 µM_x000d_ | 0.0 |
| Ng 15-13 3µM_x000d_ | 2245.6666666666665 |
| Ng 16-12 1 µM_x000d_ | 533.6666666666666 |
| Ng 17-9 1 µM_x000d_ | 0.0 |
| Ng 17-9 3 µM_x000d_ | 0.0 |
| Ng 18-11 1 µM_x000d_ | 0.0 |
| Ng 18-11 3 µM_x000d_ | 24.666666666666657 |
| Ng 20-8 1 µM_x000d_ | 0.0 |
| Ng 20-9 1 µM_x000d_ | 0.0 |
| Ng 20-9 3 µM_x000d_ | 0.0 |
| Ng 21-9 1 µM_x000d_ | 0.0 |
| Ng 13-13 3 µM_x000d_ | 0.0 |
| Ng 14-8 1 µM_x000d_ | 0.0 |
| Ng 14-9 1 µM_x000d_ | 0.0 |
| Ng 14-9 3 µM_x000d_ | 0.0 |
| Ng 14-9 10 µM_x000d_ | 0.0 |
| Ng 14-9 30 µM_x000d_ | 0.0 |
| Ng 15-10 1µM_x000d_ | 0.0 |
| Ng 16-9 1 µM_x000d_ | 589.6666666666666 |
| Ng 17-8 1 µM_x000d_ | 0.0 |
| Ng 17-8 3 µM_x000d_ | 0.0 |
| Ng 18-9 1µM_x000d_ | 3448.0 |
| Ng 19-11 1 µM_x000d_ | 0.0 |
| Ng 19-11 3 µM_x000d_ | 0.0 |
| Lg 17-15 HF 10 µM_x000d_ | 0.0 |
| Lg 15-17 1 µM_x000d_ | 0.0 |
| Lg 17-15 1 µM_x000d_ | 0.0 |
| Lg 17-15 3 µM_x000d_ | 0.0 |
| Lg 17-15 10 µM_x000d_ | 0.0 |
| Lg 17-17 1 µM_x000d_ | 0.0 |
| Lg 17-16 1 µM_x000d_ | 1873.0 |
| Lg 16-17 3 µM_x000d_ | 0.0 |
| Lg 18-8 1 µM_x000d_ | 0.0 |
| Lg 19-8 1 µM_x000d_ | 0.0 |
| Lg 19-8 3 µM_x000d_ | 0.0 |
| Lg 19-9 1 µM_x000d_ | 0.0 |
| Lg 20-10_x000d_ | 0.0 |
| Lg 22-12 1 µM_x000d_ | 0.0 |
| Lg 20-9 1 µM_x000d_ | 0.0 |
| Lg 23-11 1 µM_x000d_ | 0.0 |
| Lg 23-11 3 µM_x000d_ | 0.0 |
| Lg 23-12_x000d_ | 154.0 |
| Lg 26-8_x000d_ | 0.0 |
| Lg 26-9_x000d_ | 0.0 |
| Lg 22-13_x000d_ | 0.0 |
| Lg 26-10_x000d_ | 0.0 |
| Lg 18-7-F3 HF 1uM | 0.0 |
| Lg 19-8-F5 HF 3uM | 0.0 |
| Lg 23-11-F2 HF 3uM | 0.0 |
| Blank 1 | 0.0 |
| Blank 2 | 0.0 |
| Blank 3 | 0.0 |
| Blank 4 | 0.0 |
| Blank 5 | 0.0 |MFI
### Chart: Mp-6
| Category | Mp L23 IgG4 |
|---|---|
| Ng1_1uM
 | 96.33333333333334 |
| Ng1_3uM
 | 0.0 |
| Ng2_1uM | 0.0 |
| Ng2_3uM | 0.0 |
| Ng2_10uM | 1.0 |
| Ng3_1uM | 0.0 |
| Ng3_3uM | 0.0 |
| Ng3_10uM | 0.0 |
| Ng3_30uM | 0.0 |
| Ng4_1uM | 0.0 |
| Ng4_3uM | 0.0 |
| Ng5_1uM | 0.0 |
| Ng5_3uM | 0.0 |
| Ng6_1uM | 0.0 |
| Ng7_1uM | 0.0 |
| Ng7_3uM | 0.0 |
| Ng8_1uM | 0.0 |
| Ng8_3uM | 148.33333333333331 |
| Ng8_10uM | 209.66666666666669 |
| Ng9_1uM | 0.0 |
| Ng9_3uM | 0.0 |
| Ng10_3uM | 0.0 |
| Ng10_1uM | 0.0 |
| Ng11 | 0.0 |
| Ng12_1uM | 0.0 |
| Ng12_3uM | 137.33333333333331 |
| Ng13_1uM | 0.0 |
| Ng14_1uM | 83.66666666666666 |
| Ng15_1uM | 2072.0 |
| Ng16_1uM | 114.66666666666669 |
| Ng17_1uM | 140.66666666666669 |
| Ng18_1uM | 945.6666666666667 |
| Ng18_3uM | 2839.0 |
| Ng18_10uM | 4608.666666666667 |
| Ng19_1uM | 1133.3333333333333 |
| Ng19_3uM | 3257.6666666666665 |
| Ng19_10uM | 5093.0 |
| Ng20_1uM | 1213.0 |
| Ng20_3uM | 4177.666666666667 |
| Ng20_10uM | 7575.0 |
| Ng21_1uM | 395.66666666666663 |
| Ng21 _3uM | 2185.6666666666665 |
| Ng21_10uM | 8320.666666666666 |
| Ng22_1uM | 1780.6666666666667 |
| Ng23_1uM | 0.0 |
| Ng24_1uM | 1324.6666666666667 |
| Ng24_3uM | 1838.3333333333333 |
| Ng25_1uM | 688.0 |
| Ng25_3uM | 542.0 |
| Ng26_1uM | 1999.3333333333335 |
| Ng27_1uM | 323.3333333333333 |
| Ng27_3uM | 1573.0 |
| Ng28_1uM | 189.0 |
| Ng29_1uM | 0.0 |
| Ng30_1uM | 627.6666666666666 |
| Ng31_1uM | 0.0 |
| Ng31_3uM | 0.0 |
| Ng31_10uM | 0.0 |
| Ng31_30uM | 0.0 |
| Ng32_1uM | 5024.666666666667 |
| Ng33_1uM | 2424.0 |
| Ng34_1uM | 559.3333333333334 |
| Ng34_3uM | 1576.3333333333333 |
| Ng35_1uM | 1812.3333333333333 |
| Ng36_1uM | 942.3333333333333 |
| Ng36_3uM | 1214.3333333333333 |
| Lg2_HF_10uM | 3792.6666666666665 |
| Lg1_1uM | 110.33333333333331 |
| Lg2_1uM | 182.66666666666669 |
| Lg2_3uM | 547.6666666666666 |
| Lg2_10uM | 3175.3333333333335 |
| Lg3_1uM | 184.0 |
| Lg4_1uM | 3507.3333333333335 |
| Lg5_3uM | 22028.666666666668 |
| Lg6_1uM | 1138.6666666666667 |
| Lg7_1uM | 2140.0 |
| Lg7_3uM | 6535.666666666667 |
| Lg8_1uM | 1154.3333333333333 |
| Lg9 | 2561.6666666666665 |
| Lg10_1uM | 775.3333333333334 |
| Lg11_1uM | 6636.333333333333 |
| Lg12_1uM | 2768.0 |
| Lg12_3uM | 4349.666666666667 |
| Lg13 | 1958.0 |
| Lg14 | 2815.0 |
| Lg15 | 717.0 |
| Lg16 | 371.0 |
| Lg17 | 67.66666666666666 |
| Lg18_HF_1uM | 2212.0 |
| Lg7_HF_3uM | 5909.333333333333 |
| Lg12_HF_3uM | 3419.0 |
| Blank 1 | 0.0 |
| Blank 2 | 0.0 |
| Blank 3 | 0.0 |
| Blank 4 | 0.0 |
| Blank 5 | 0.0 |MFI

## Slide 33
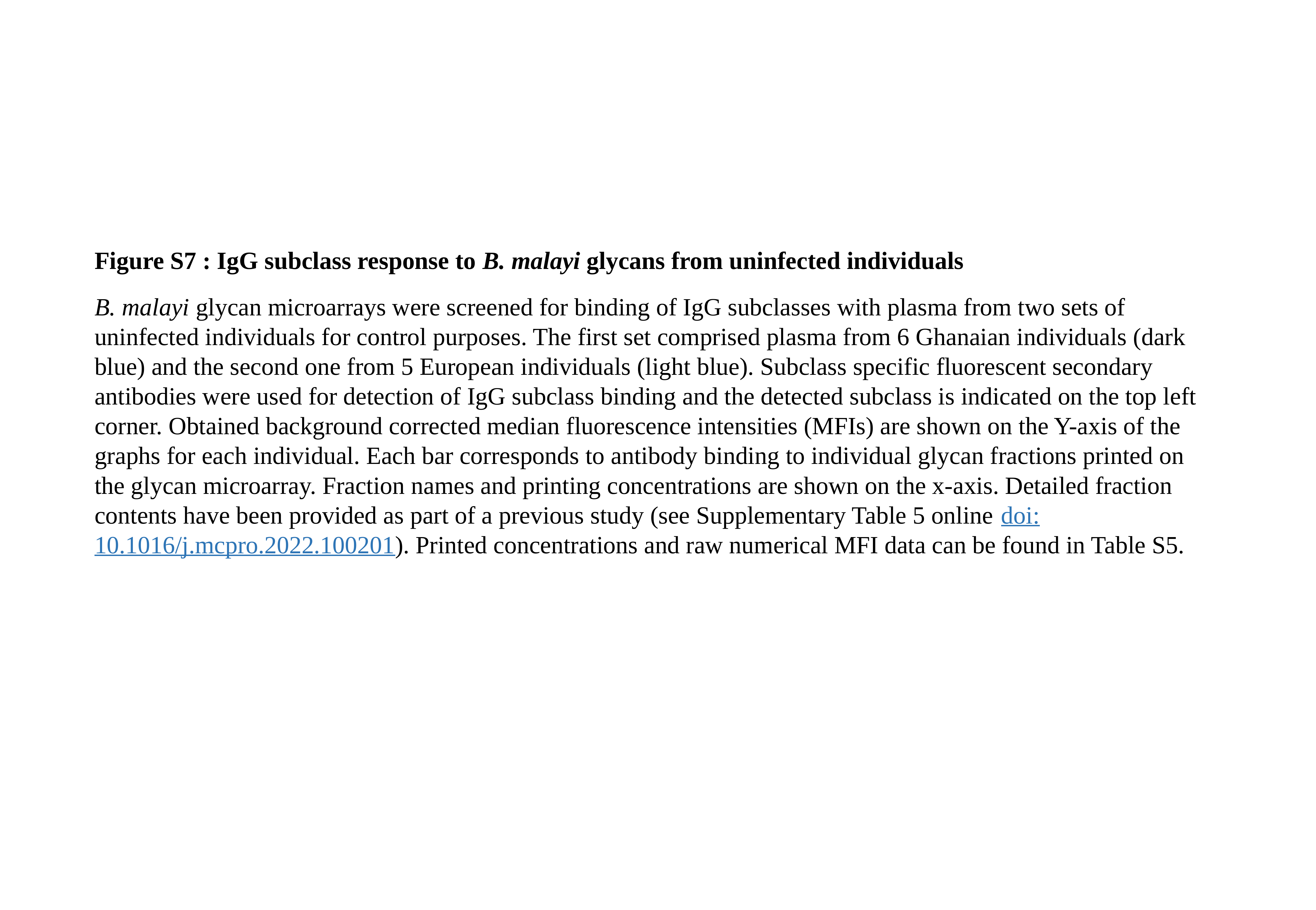

Figure S7 : IgG subclass response to B. malayi glycans from uninfected individuals
B. malayi glycan microarrays were screened for binding of IgG subclasses with plasma from two sets of uninfected individuals for control purposes. The first set comprised plasma from 6 Ghanaian individuals (dark blue) and the second one from 5 European individuals (light blue). Subclass specific fluorescent secondary antibodies were used for detection of IgG subclass binding and the detected subclass is indicated on the top left corner. Obtained background corrected median fluorescence intensities (MFIs) are shown on the Y-axis of the graphs for each individual. Each bar corresponds to antibody binding to individual glycan fractions printed on the glycan microarray. Fraction names and printing concentrations are shown on the x-axis. Detailed fraction contents have been provided as part of a previous study (see Supplementary Table 5 online doi: 10.1016/j.mcpro.2022.100201). Printed concentrations and raw numerical MFI data can be found in Table S5.

## Slide 34
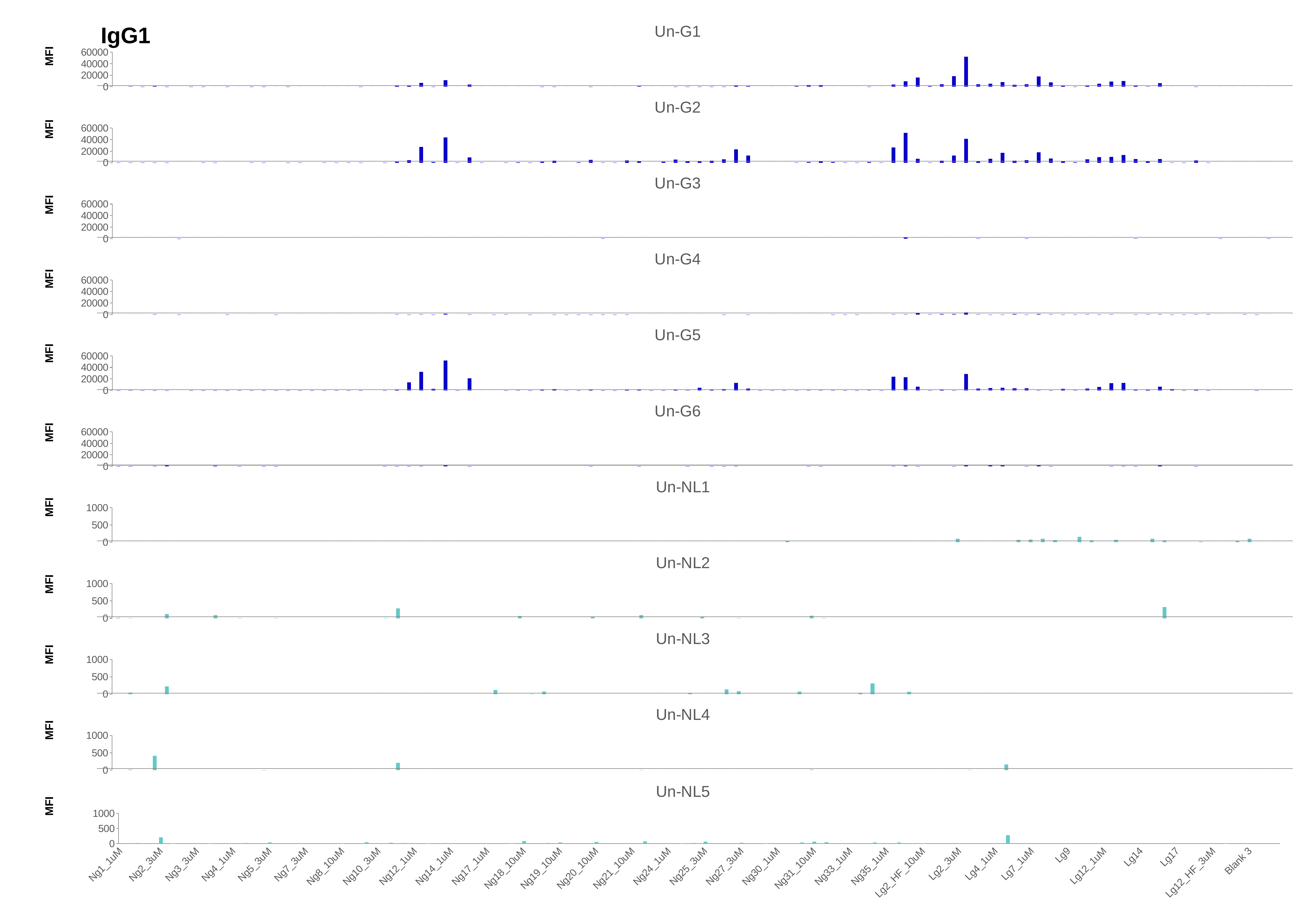

### Chart: Un-G1
| Category | UG 000-214 IgG1 |
|---|---|
| Ng 7-13 1µM_x000d_ | 0.0 |
| Ng 7-13 3µM_x000d_ | 454.0 |
| Ng 9-9 1 µM_x000d_ | 120.33333333333331 |
| Ng 9-9 3µM_x000d_ | 1109.6666666666667 |
| Ng 9-9 10µM_x000d_ | 217.66666666666669 |
| Ng 10-13 1 µM_x000d_ | 0.0 |
| Ng 10-13 3 µM_x000d_ | 14.333333333333343 |
| Ng 10-13 10 µM_x000d_ | 37.66666666666666 |
| Ng 10-13 30 µM_x000d_ | 0.0 |
| Ng 10-10 1 µM_x000d_ | 3.333333333333343 |
| Ng 10-10 3 µM_x000d_ | 0.0 |
| Ng 10-11 1 µM_x000d_ | 4.666666666666657 |
| Ng 10-11 3 µM_x000d_ | 112.66666666666669 |
| Ng 10-12 1 µM_x000d_ | 0.0 |
| Ng 11-10 1 µM_x000d_ | 29.333333333333343 |
| Ng 11-10 3 µM_x000d_ | 0.0 |
| Ng 11-11 1 µM_x000d_ | 0.0 |
| Ng 11-11 3µM_x000d_ | 0.0 |
| Ng 11-11 10µM_x000d_ | 0.0 |
| Ng 12-11 1 µM_x000d_ | 0.0 |
| Ng 12-11 3 µM_x000d_ | 30.0 |
| Ng 13-15 1 µM_x000d_ | 0.0 |
| Ng 13-15 3 µM_x000d_ | 0.0 |
| Ng 13-14_x000d_ | 1589.0 |
| Ng 14-10 1 µM_x000d_ | 2177.0 |
| Ng 14-10 3 µM_x000d_ | 6569.333333333333 |
| Ng 14-11 1 µM_x000d_ | 96.66666666666666 |
| Ng 14-12 1 µM_x000d_ | 11323.0 |
| Ng 14-7 1 µM_x000d_ | 0.0 |
| Ng 15-14/15 1 µM_x000d_ | 3914.3333333333335 |
| Ng 15-12 1µM_x000d_ | 0.0 |
| Ng 17-7 1 µM_x000d_ | 0.0 |
| Ng 17-7 3 µM_x000d_ | 0.0 |
| Ng 17-7 10 µM_x000d_ | 0.0 |
| Ng 19-9 1 µM_x000d_ | 0.0 |
| Ng 19-9 3 µM_x000d_ | 16.0 |
| Ng 19-9 10 µM_x000d_ | 183.33333333333331 |
| Ng 20-7 1 µM_x000d_ | 0.0 |
| Ng 20-7 3 µM_x000d_ | 0.0 |
| Ng 20-7 10 µM_x000d_ | 64.33333333333334 |
| Ng 21-8 1 µM_x000d_ | 0.0 |
| Ng 21-8 3 µM_x000d_ | 0.0 |
| Ng 21-8 10 µM_x000d_ | 0.0 |
| Ng 15-13 3µM_x000d_ | 938.0 |
| Ng 16-12 1 µM_x000d_ | 0.0 |
| Ng 17-9 1 µM_x000d_ | 0.0 |
| Ng 17-9 3 µM_x000d_ | 365.66666666666663 |
| Ng 18-11 1 µM_x000d_ | 190.66666666666669 |
| Ng 18-11 3 µM_x000d_ | 190.66666666666669 |
| Ng 20-8 1 µM_x000d_ | 45.66666666666666 |
| Ng 20-9 1 µM_x000d_ | 148.33333333333331 |
| Ng 20-9 3 µM_x000d_ | 1858.0 |
| Ng 21-9 1 µM_x000d_ | 1162.6666666666667 |
| Ng 13-13 3 µM_x000d_ | 0.0 |
| Ng 14-8 1 µM_x000d_ | 0.0 |
| Ng 14-9 1 µM_x000d_ | 0.0 |
| Ng 14-9 3 µM_x000d_ | 958.6666666666667 |
| Ng 14-9 10 µM_x000d_ | 2913.3333333333335 |
| Ng 14-9 30 µM_x000d_ | 3145.0 |
| Ng 15-10 1µM_x000d_ | 0.0 |
| Ng 16-9 1 µM_x000d_ | 0.0 |
| Ng 17-8 1 µM_x000d_ | 0.0 |
| Ng 17-8 3 µM_x000d_ | 202.33333333333331 |
| Ng 18-9 1µM_x000d_ | 0.0 |
| Ng 19-11 1 µM_x000d_ | 4084.333333333333 |
| Ng 19-11 3 µM_x000d_ | 9349.0 |
| Lg 17-15 HF 10 µM_x000d_ | 15949.0 |
| Lg 15-17 1 µM_x000d_ | 1018.3333333333333 |
| Lg 17-15 1 µM_x000d_ | 4235.0 |
| Lg 17-15 3 µM_x000d_ | 18488.666666666668 |
| Lg 17-15 10 µM_x000d_ | 52093.0 |
| Lg 17-17 1 µM_x000d_ | 4295.666666666667 |
| Lg 17-16 1 µM_x000d_ | 5099.666666666667 |
| Lg 16-17 3 µM_x000d_ | 8210.333333333334 |
| Lg 18-8 1 µM_x000d_ | 3295.0 |
| Lg 19-8 1 µM_x000d_ | 4409.333333333333 |
| Lg 19-8 3 µM_x000d_ | 18135.0 |
| Lg 19-9 1 µM_x000d_ | 7701.0 |
| Lg 20-10_x000d_ | 2296.6666666666665 |
| Lg 22-12 1 µM_x000d_ | 6.666666666666657 |
| Lg 20-9 1 µM_x000d_ | 1860.3333333333333 |
| Lg 23-11 1 µM_x000d_ | 5460.333333333333 |
| Lg 23-11 3 µM_x000d_ | 9066.333333333334 |
| Lg 23-12_x000d_ | 9886.333333333334 |
| Lg 26-8_x000d_ | 1616.3333333333333 |
| Lg 26-9_x000d_ | 544.3333333333334 |
| Lg 22-13_x000d_ | 6395.333333333333 |
| Lg 26-10_x000d_ | 0.0 |
| Lg 18-7-F3 HF 1uM | 0.0 |
| Lg 19-8-F5 HF 3uM | 332.0 |
| Lg 23-11-F2 HF 3uM | 0.0 |
| Blank 1 | 0.0 |
| Blank 2 | 0.0 |
| Blank 3 | 0.0 |
| Blank 4 | 0.0 |
| Blank 5 | 0.0 |IgG1
MFI
### Chart: Un-G2
| Category | UG 000-215 IgG1 |
|---|---|
| Ng 7-13 1µM_x000d_ | 44.33333333333334 |
| Ng 7-13 3µM_x000d_ | 418.0 |
| Ng 9-9 1 µM_x000d_ | 9.333333333333343 |
| Ng 9-9 3µM_x000d_ | 56.66666666666666 |
| Ng 9-9 10µM_x000d_ | 53.33333333333334 |
| Ng 10-13 1 µM_x000d_ | 0.0 |
| Ng 10-13 3 µM_x000d_ | 0.0 |
| Ng 10-13 10 µM_x000d_ | 6.333333333333343 |
| Ng 10-13 30 µM_x000d_ | 30.333333333333343 |
| Ng 10-10 1 µM_x000d_ | 0.0 |
| Ng 10-10 3 µM_x000d_ | 0.0 |
| Ng 10-11 1 µM_x000d_ | 5.0 |
| Ng 10-11 3 µM_x000d_ | 21.666666666666657 |
| Ng 10-12 1 µM_x000d_ | 0.0 |
| Ng 11-10 1 µM_x000d_ | 9.333333333333343 |
| Ng 11-10 3 µM_x000d_ | 10.333333333333343 |
| Ng 11-11 1 µM_x000d_ | 0.0 |
| Ng 11-11 3µM_x000d_ | 5.666666666666657 |
| Ng 11-11 10µM_x000d_ | 5.333333333333343 |
| Ng 12-11 1 µM_x000d_ | 8.0 |
| Ng 12-11 3 µM_x000d_ | 32.66666666666666 |
| Ng 13-15 1 µM_x000d_ | 0.0 |
| Ng 13-15 3 µM_x000d_ | 5.0 |
| Ng 13-14_x000d_ | 1744.0 |
| Ng 14-10 1 µM_x000d_ | 4297.333333333333 |
| Ng 14-10 3 µM_x000d_ | 27504.333333333332 |
| Ng 14-11 1 µM_x000d_ | 1091.6666666666667 |
| Ng 14-12 1 µM_x000d_ | 43705.666666666664 |
| Ng 14-7 1 µM_x000d_ | 64.0 |
| Ng 15-14/15 1 µM_x000d_ | 9318.0 |
| Ng 15-12 1µM_x000d_ | 3.666666666666657 |
| Ng 17-7 1 µM_x000d_ | 0.0 |
| Ng 17-7 3 µM_x000d_ | 177.0 |
| Ng 17-7 10 µM_x000d_ | 723.6666666666666 |
| Ng 19-9 1 µM_x000d_ | 100.0 |
| Ng 19-9 3 µM_x000d_ | 1838.3333333333333 |
| Ng 19-9 10 µM_x000d_ | 3335.3333333333335 |
| Ng 20-7 1 µM_x000d_ | 0.0 |
| Ng 20-7 3 µM_x000d_ | 708.6666666666666 |
| Ng 20-7 10 µM_x000d_ | 5006.0 |
| Ng 21-8 1 µM_x000d_ | 83.66666666666666 |
| Ng 21-8 3 µM_x000d_ | 423.66666666666663 |
| Ng 21-8 10 µM_x000d_ | 3800.3333333333335 |
| Ng 15-13 3µM_x000d_ | 2369.0 |
| Ng 16-12 1 µM_x000d_ | 0.0 |
| Ng 17-9 1 µM_x000d_ | 1612.6666666666667 |
| Ng 17-9 3 µM_x000d_ | 5287.666666666667 |
| Ng 18-11 1 µM_x000d_ | 2628.0 |
| Ng 18-11 3 µM_x000d_ | 2672.6666666666665 |
| Ng 20-8 1 µM_x000d_ | 3375.6666666666665 |
| Ng 20-9 1 µM_x000d_ | 5781.0 |
| Ng 20-9 3 µM_x000d_ | 22988.0 |
| Ng 21-9 1 µM_x000d_ | 12189.333333333334 |
| Ng 13-13 3 µM_x000d_ | 0.0 |
| Ng 14-8 1 µM_x000d_ | 0.0 |
| Ng 14-9 1 µM_x000d_ | 0.0 |
| Ng 14-9 3 µM_x000d_ | 29.0 |
| Ng 14-9 10 µM_x000d_ | 1205.3333333333333 |
| Ng 14-9 30 µM_x000d_ | 2931.3333333333335 |
| Ng 15-10 1µM_x000d_ | 1037.3333333333333 |
| Ng 16-9 1 µM_x000d_ | 139.66666666666669 |
| Ng 17-8 1 µM_x000d_ | 196.33333333333331 |
| Ng 17-8 3 µM_x000d_ | 1209.6666666666667 |
| Ng 18-9 1µM_x000d_ | 130.66666666666669 |
| Ng 19-11 1 µM_x000d_ | 26517.0 |
| Ng 19-11 3 µM_x000d_ | 51674.0 |
| Lg 17-15 HF 10 µM_x000d_ | 6577.333333333333 |
| Lg 15-17 1 µM_x000d_ | 47.0 |
| Lg 17-15 1 µM_x000d_ | 3320.3333333333335 |
| Lg 17-15 3 µM_x000d_ | 12559.666666666666 |
| Lg 17-15 10 µM_x000d_ | 41546.0 |
| Lg 17-17 1 µM_x000d_ | 2746.3333333333335 |
| Lg 17-16 1 µM_x000d_ | 6671.0 |
| Lg 16-17 3 µM_x000d_ | 16995.666666666668 |
| Lg 18-8 1 µM_x000d_ | 3619.0 |
| Lg 19-8 1 µM_x000d_ | 4638.333333333333 |
| Lg 19-8 3 µM_x000d_ | 17894.0 |
| Lg 19-9 1 µM_x000d_ | 7217.666666666667 |
| Lg 20-10_x000d_ | 2713.3333333333335 |
| Lg 22-12 1 µM_x000d_ | 930.0 |
| Lg 20-9 1 µM_x000d_ | 5744.333333333333 |
| Lg 23-11 1 µM_x000d_ | 9815.666666666666 |
| Lg 23-11 3 µM_x000d_ | 10164.333333333334 |
| Lg 23-12_x000d_ | 13460.666666666666 |
| Lg 26-8_x000d_ | 6533.0 |
| Lg 26-9_x000d_ | 2210.3333333333335 |
| Lg 22-13_x000d_ | 6139.0 |
| Lg 26-10_x000d_ | 75.0 |
| Lg 18-7-F3 HF 1uM | 145.0 |
| Lg 19-8-F5 HF 3uM | 4003.0 |
| Lg 23-11-F2 HF 3uM | 147.0 |
| Blank 1 | 0.0 |
| Blank 2 | 0.0 |
| Blank 3 | 0.0 |
| Blank 4 | 0.0 |
| Blank 5 | 0.0 |MFI
### Chart: Un-G3
| Category | UG 000-223 IgG1 |
|---|---|
| Ng 7-13 1µM_x000d_ | 0.0 |
| Ng 7-13 3µM_x000d_ | 0.0 |
| Ng 9-9 1 µM_x000d_ | 0.0 |
| Ng 9-9 3µM_x000d_ | 0.0 |
| Ng 9-9 10µM_x000d_ | 0.0 |
| Ng 10-13 1 µM_x000d_ | 80.0 |
| Ng 10-13 3 µM_x000d_ | 0.0 |
| Ng 10-13 10 µM_x000d_ | 0.0 |
| Ng 10-13 30 µM_x000d_ | 0.0 |
| Ng 10-10 1 µM_x000d_ | 0.0 |
| Ng 10-10 3 µM_x000d_ | 0.0 |
| Ng 10-11 1 µM_x000d_ | 0.0 |
| Ng 10-11 3 µM_x000d_ | 0.0 |
| Ng 10-12 1 µM_x000d_ | 0.0 |
| Ng 11-10 1 µM_x000d_ | 0.0 |
| Ng 11-10 3 µM_x000d_ | 0.0 |
| Ng 11-11 1 µM_x000d_ | 0.0 |
| Ng 11-11 3µM_x000d_ | 0.0 |
| Ng 11-11 10µM_x000d_ | 0.0 |
| Ng 12-11 1 µM_x000d_ | 0.0 |
| Ng 12-11 3 µM_x000d_ | 0.0 |
| Ng 13-15 1 µM_x000d_ | 0.0 |
| Ng 13-15 3 µM_x000d_ | 0.0 |
| Ng 13-14_x000d_ | 0.0 |
| Ng 14-10 1 µM_x000d_ | 0.0 |
| Ng 14-10 3 µM_x000d_ | 0.0 |
| Ng 14-11 1 µM_x000d_ | 0.0 |
| Ng 14-12 1 µM_x000d_ | 0.0 |
| Ng 14-7 1 µM_x000d_ | 0.0 |
| Ng 15-14/15 1 µM_x000d_ | 0.0 |
| Ng 15-12 1µM_x000d_ | 0.0 |
| Ng 17-7 1 µM_x000d_ | 0.0 |
| Ng 17-7 3 µM_x000d_ | 0.0 |
| Ng 17-7 10 µM_x000d_ | 0.0 |
| Ng 19-9 1 µM_x000d_ | 0.0 |
| Ng 19-9 3 µM_x000d_ | 0.0 |
| Ng 19-9 10 µM_x000d_ | 0.0 |
| Ng 20-7 1 µM_x000d_ | 0.0 |
| Ng 20-7 3 µM_x000d_ | 0.0 |
| Ng 20-7 10 µM_x000d_ | 0.0 |
| Ng 21-8 1 µM_x000d_ | 213.0000000000001 |
| Ng 21-8 3 µM_x000d_ | 0.0 |
| Ng 21-8 10 µM_x000d_ | 0.0 |
| Ng 15-13 3µM_x000d_ | 0.0 |
| Ng 16-12 1 µM_x000d_ | 0.0 |
| Ng 17-9 1 µM_x000d_ | 0.0 |
| Ng 17-9 3 µM_x000d_ | 0.0 |
| Ng 18-11 1 µM_x000d_ | 0.0 |
| Ng 18-11 3 µM_x000d_ | 0.0 |
| Ng 20-8 1 µM_x000d_ | 0.0 |
| Ng 20-9 1 µM_x000d_ | 0.0 |
| Ng 20-9 3 µM_x000d_ | 0.0 |
| Ng 21-9 1 µM_x000d_ | 0.0 |
| Ng 13-13 3 µM_x000d_ | 0.0 |
| Ng 14-8 1 µM_x000d_ | 0.0 |
| Ng 14-9 1 µM_x000d_ | 0.0 |
| Ng 14-9 3 µM_x000d_ | 0.0 |
| Ng 14-9 10 µM_x000d_ | 0.0 |
| Ng 14-9 30 µM_x000d_ | 0.0 |
| Ng 15-10 1µM_x000d_ | 0.0 |
| Ng 16-9 1 µM_x000d_ | 0.0 |
| Ng 17-8 1 µM_x000d_ | 0.0 |
| Ng 17-8 3 µM_x000d_ | 0.0 |
| Ng 18-9 1µM_x000d_ | 0.0 |
| Ng 19-11 1 µM_x000d_ | 0.0 |
| Ng 19-11 3 µM_x000d_ | 1653.666666666667 |
| Lg 17-15 HF 10 µM_x000d_ | 0.0 |
| Lg 15-17 1 µM_x000d_ | 0.0 |
| Lg 17-15 1 µM_x000d_ | 0.0 |
| Lg 17-15 3 µM_x000d_ | 0.0 |
| Lg 17-15 10 µM_x000d_ | 0.0 |
| Lg 17-17 1 µM_x000d_ | 101.66666666666674 |
| Lg 17-16 1 µM_x000d_ | 0.0 |
| Lg 16-17 3 µM_x000d_ | 0.0 |
| Lg 18-8 1 µM_x000d_ | 0.0 |
| Lg 19-8 1 µM_x000d_ | 327.33333333333337 |
| Lg 19-8 3 µM_x000d_ | 0.0 |
| Lg 19-9 1 µM_x000d_ | 0.0 |
| Lg 20-10_x000d_ | 0.0 |
| Lg 22-12 1 µM_x000d_ | 0.0 |
| Lg 20-9 1 µM_x000d_ | 0.0 |
| Lg 23-11 1 µM_x000d_ | 0.0 |
| Lg 23-11 3 µM_x000d_ | 0.0 |
| Lg 23-12_x000d_ | 0.0 |
| Lg 26-8_x000d_ | 175.66666666666663 |
| Lg 26-9_x000d_ | 0.0 |
| Lg 22-13_x000d_ | 0.0 |
| Lg 26-10_x000d_ | 0.0 |
| Lg 18-7-F3 HF 1uM | 0.0 |
| Lg 19-8-F5 HF 3uM | 0.0 |
| Lg 23-11-F2 HF 3uM | 0.0 |
| Blank 1 | 230.33333333333337 |
| Blank 2 | 0.0 |
| Blank 3 | 0.0 |
| Blank 4 | 0.0 |
| Blank 5 | 366.83333333333337 |MFI
### Chart: Un-G4
| Category | UG 000-224 IgG1 |
|---|---|
| Ng 7-13 1µM_x000d_ | 0.0 |
| Ng 7-13 3µM_x000d_ | 0.0 |
| Ng 9-9 1 µM_x000d_ | 0.0 |
| Ng 9-9 3µM_x000d_ | 562.3333333333334 |
| Ng 9-9 10µM_x000d_ | 0.0 |
| Ng 10-13 1 µM_x000d_ | 19.666666666666657 |
| Ng 10-13 3 µM_x000d_ | 0.0 |
| Ng 10-13 10 µM_x000d_ | 0.0 |
| Ng 10-13 30 µM_x000d_ | 0.0 |
| Ng 10-10 1 µM_x000d_ | 144.0 |
| Ng 10-10 3 µM_x000d_ | 0.0 |
| Ng 10-11 1 µM_x000d_ | 0.0 |
| Ng 10-11 3 µM_x000d_ | 0.0 |
| Ng 10-12 1 µM_x000d_ | 129.33333333333331 |
| Ng 11-10 1 µM_x000d_ | 0.0 |
| Ng 11-10 3 µM_x000d_ | 0.0 |
| Ng 11-11 1 µM_x000d_ | 0.0 |
| Ng 11-11 3µM_x000d_ | 0.0 |
| Ng 11-11 10µM_x000d_ | 0.0 |
| Ng 12-11 1 µM_x000d_ | 0.0 |
| Ng 12-11 3 µM_x000d_ | 0.0 |
| Ng 13-15 1 µM_x000d_ | 0.0 |
| Ng 13-15 3 µM_x000d_ | 0.0 |
| Ng 13-14_x000d_ | 225.0 |
| Ng 14-10 1 µM_x000d_ | 62.666666666666686 |
| Ng 14-10 3 µM_x000d_ | 499.66666666666663 |
| Ng 14-11 1 µM_x000d_ | 53.33333333333334 |
| Ng 14-12 1 µM_x000d_ | 1225.0 |
| Ng 14-7 1 µM_x000d_ | 0.0 |
| Ng 15-14/15 1 µM_x000d_ | 267.6666666666667 |
| Ng 15-12 1µM_x000d_ | 0.0 |
| Ng 17-7 1 µM_x000d_ | 72.66666666666669 |
| Ng 17-7 3 µM_x000d_ | 260.6666666666667 |
| Ng 17-7 10 µM_x000d_ | 0.0 |
| Ng 19-9 1 µM_x000d_ | 43.33333333333334 |
| Ng 19-9 3 µM_x000d_ | 0.0 |
| Ng 19-9 10 µM_x000d_ | 51.66666666666666 |
| Ng 20-7 1 µM_x000d_ | 23.0 |
| Ng 20-7 3 µM_x000d_ | 58.0 |
| Ng 20-7 10 µM_x000d_ | 67.0 |
| Ng 21-8 1 µM_x000d_ | 51.66666666666666 |
| Ng 21-8 3 µM_x000d_ | 4.333333333333343 |
| Ng 21-8 10 µM_x000d_ | 70.33333333333331 |
| Ng 15-13 3µM_x000d_ | 0.0 |
| Ng 16-12 1 µM_x000d_ | 0.0 |
| Ng 17-9 1 µM_x000d_ | 0.0 |
| Ng 17-9 3 µM_x000d_ | 0.0 |
| Ng 18-11 1 µM_x000d_ | 0.0 |
| Ng 18-11 3 µM_x000d_ | 0.0 |
| Ng 20-8 1 µM_x000d_ | 0.0 |
| Ng 20-9 1 µM_x000d_ | 17.333333333333343 |
| Ng 20-9 3 µM_x000d_ | 0.0 |
| Ng 21-9 1 µM_x000d_ | 156.66666666666669 |
| Ng 13-13 3 µM_x000d_ | 0.0 |
| Ng 14-8 1 µM_x000d_ | 0.0 |
| Ng 14-9 1 µM_x000d_ | 0.0 |
| Ng 14-9 3 µM_x000d_ | 0.0 |
| Ng 14-9 10 µM_x000d_ | 0.0 |
| Ng 14-9 30 µM_x000d_ | 0.0 |
| Ng 15-10 1µM_x000d_ | 51.0 |
| Ng 16-9 1 µM_x000d_ | 54.0 |
| Ng 17-8 1 µM_x000d_ | 152.33333333333331 |
| Ng 17-8 3 µM_x000d_ | 0.0 |
| Ng 18-9 1µM_x000d_ | 0.0 |
| Ng 19-11 1 µM_x000d_ | 216.66666666666669 |
| Ng 19-11 3 µM_x000d_ | 486.0 |
| Lg 17-15 HF 10 µM_x000d_ | 2720.0 |
| Lg 15-17 1 µM_x000d_ | 527.0 |
| Lg 17-15 1 µM_x000d_ | 663.0 |
| Lg 17-15 3 µM_x000d_ | 641.3333333333334 |
| Lg 17-15 10 µM_x000d_ | 3711.3333333333335 |
| Lg 17-17 1 µM_x000d_ | 167.66666666666669 |
| Lg 17-16 1 µM_x000d_ | 102.33333333333331 |
| Lg 16-17 3 µM_x000d_ | 87.66666666666669 |
| Lg 18-8 1 µM_x000d_ | 859.6666666666667 |
| Lg 19-8 1 µM_x000d_ | 109.0 |
| Lg 19-8 3 µM_x000d_ | 699.0 |
| Lg 19-9 1 µM_x000d_ | 457.33333333333337 |
| Lg 20-10_x000d_ | 101.66666666666669 |
| Lg 22-12 1 µM_x000d_ | 5.0 |
| Lg 20-9 1 µM_x000d_ | 537.0 |
| Lg 23-11 1 µM_x000d_ | 126.33333333333331 |
| Lg 23-11 3 µM_x000d_ | 622.6666666666666 |
| Lg 23-12_x000d_ | 0.0 |
| Lg 26-8_x000d_ | 165.33333333333331 |
| Lg 26-9_x000d_ | 443.33333333333337 |
| Lg 22-13_x000d_ | 207.33333333333331 |
| Lg 26-10_x000d_ | 74.66666666666669 |
| Lg 18-7-F3 HF 1uM | 107.0 |
| Lg 19-8-F5 HF 3uM | 238.0 |
| Lg 23-11-F2 HF 3uM | 183.33333333333331 |
| Blank 1 | 0.0 |
| Blank 2 | 0.0 |
| Blank 3 | 225.0 |
| Blank 4 | 69.33333333333331 |
| Blank 5 | 0.0 |MFI
### Chart: Un-G5
| Category | UG 000-225 IgG1 |
|---|---|
| Ng 7-13 1µM_x000d_ | 151.0 |
| Ng 7-13 3µM_x000d_ | 336.6666666666667 |
| Ng 9-9 1 µM_x000d_ | 65.33333333333334 |
| Ng 9-9 3µM_x000d_ | 135.66666666666669 |
| Ng 9-9 10µM_x000d_ | 133.33333333333331 |
| Ng 10-13 1 µM_x000d_ | 0.0 |
| Ng 10-13 3 µM_x000d_ | 9.666666666666657 |
| Ng 10-13 10 µM_x000d_ | 44.66666666666666 |
| Ng 10-13 30 µM_x000d_ | 6.0 |
| Ng 10-10 1 µM_x000d_ | 37.33333333333334 |
| Ng 10-10 3 µM_x000d_ | 74.33333333333334 |
| Ng 10-11 1 µM_x000d_ | 72.33333333333334 |
| Ng 10-11 3 µM_x000d_ | 65.66666666666666 |
| Ng 10-12 1 µM_x000d_ | 48.33333333333334 |
| Ng 11-10 1 µM_x000d_ | 8.333333333333343 |
| Ng 11-10 3 µM_x000d_ | 57.33333333333334 |
| Ng 11-11 1 µM_x000d_ | 20.0 |
| Ng 11-11 3µM_x000d_ | 71.66666666666666 |
| Ng 11-11 10µM_x000d_ | 0.3333333333333428 |
| Ng 12-11 1 µM_x000d_ | 56.66666666666666 |
| Ng 12-11 3 µM_x000d_ | 57.33333333333334 |
| Ng 13-15 1 µM_x000d_ | 0.0 |
| Ng 13-15 3 µM_x000d_ | 97.33333333333334 |
| Ng 13-14_x000d_ | 1941.0 |
| Ng 14-10 1 µM_x000d_ | 14243.0 |
| Ng 14-10 3 µM_x000d_ | 32101.666666666668 |
| Ng 14-11 1 µM_x000d_ | 2650.3333333333335 |
| Ng 14-12 1 µM_x000d_ | 52033.0 |
| Ng 14-7 1 µM_x000d_ | 81.66666666666666 |
| Ng 15-14/15 1 µM_x000d_ | 20927.0 |
| Ng 15-12 1µM_x000d_ | 0.0 |
| Ng 17-7 1 µM_x000d_ | 0.0 |
| Ng 17-7 3 µM_x000d_ | 79.0 |
| Ng 17-7 10 µM_x000d_ | 222.66666666666669 |
| Ng 19-9 1 µM_x000d_ | 114.66666666666669 |
| Ng 19-9 3 µM_x000d_ | 779.3333333333334 |
| Ng 19-9 10 µM_x000d_ | 2101.0 |
| Ng 20-7 1 µM_x000d_ | 54.66666666666666 |
| Ng 20-7 3 µM_x000d_ | 141.0 |
| Ng 20-7 10 µM_x000d_ | 1629.6666666666667 |
| Ng 21-8 1 µM_x000d_ | 125.66666666666669 |
| Ng 21-8 3 µM_x000d_ | 84.33333333333334 |
| Ng 21-8 10 µM_x000d_ | 1312.6666666666667 |
| Ng 15-13 3µM_x000d_ | 1490.3333333333333 |
| Ng 16-12 1 µM_x000d_ | 11.666666666666657 |
| Ng 17-9 1 µM_x000d_ | 151.66666666666669 |
| Ng 17-9 3 µM_x000d_ | 2018.0 |
| Ng 18-11 1 µM_x000d_ | 487.0 |
| Ng 18-11 3 µM_x000d_ | 4472.0 |
| Ng 20-8 1 µM_x000d_ | 1333.6666666666667 |
| Ng 20-9 1 µM_x000d_ | 2371.6666666666665 |
| Ng 20-9 3 µM_x000d_ | 13002.333333333334 |
| Ng 21-9 1 µM_x000d_ | 3042.3333333333335 |
| Ng 13-13 3 µM_x000d_ | 211.0 |
| Ng 14-8 1 µM_x000d_ | 55.33333333333334 |
| Ng 14-9 1 µM_x000d_ | 0.3333333333333428 |
| Ng 14-9 3 µM_x000d_ | 93.33333333333334 |
| Ng 14-9 10 µM_x000d_ | 350.3333333333333 |
| Ng 14-9 30 µM_x000d_ | 299.6666666666667 |
| Ng 15-10 1µM_x000d_ | 276.3333333333333 |
| Ng 16-9 1 µM_x000d_ | 66.33333333333334 |
| Ng 17-8 1 µM_x000d_ | 80.33333333333334 |
| Ng 17-8 3 µM_x000d_ | 400.0 |
| Ng 18-9 1µM_x000d_ | 97.33333333333334 |
| Ng 19-11 1 µM_x000d_ | 23973.333333333332 |
| Ng 19-11 3 µM_x000d_ | 22759.666666666668 |
| Lg 17-15 HF 10 µM_x000d_ | 6481.0 |
| Lg 15-17 1 µM_x000d_ | 82.66666666666666 |
| Lg 17-15 1 µM_x000d_ | 2031.6666666666665 |
| Lg 17-15 3 µM_x000d_ | 211.33333333333331 |
| Lg 17-15 10 µM_x000d_ | 28418.666666666668 |
| Lg 17-17 1 µM_x000d_ | 3116.6666666666665 |
| Lg 17-16 1 µM_x000d_ | 4383.333333333333 |
| Lg 16-17 3 µM_x000d_ | 4868.333333333333 |
| Lg 18-8 1 µM_x000d_ | 3953.333333333333 |
| Lg 19-8 1 µM_x000d_ | 3689.6666666666665 |
| Lg 19-8 3 µM_x000d_ | 291.0 |
| Lg 19-9 1 µM_x000d_ | 102.33333333333334 |
| Lg 20-10_x000d_ | 2914.0 |
| Lg 22-12 1 µM_x000d_ | 520.0 |
| Lg 20-9 1 µM_x000d_ | 3144.3333333333335 |
| Lg 23-11 1 µM_x000d_ | 6042.333333333333 |
| Lg 23-11 3 µM_x000d_ | 12739.333333333334 |
| Lg 23-12_x000d_ | 13197.333333333334 |
| Lg 26-8_x000d_ | 1225.0 |
| Lg 26-9_x000d_ | 1933.0 |
| Lg 22-13_x000d_ | 6375.0 |
| Lg 26-10_x000d_ | 2167.0 |
| Lg 18-7-F3 HF 1uM | 73.0 |
| Lg 19-8-F5 HF 3uM | 874.6666666666667 |
| Lg 23-11-F2 HF 3uM | 46.0 |
| Blank 1 | 0.0 |
| Blank 2 | 0.0 |
| Blank 3 | 0.0 |
| Blank 4 | 9.666666666666657 |
| Blank 5 | 0.0 |MFI
### Chart: Un-G6
| Category | UG 000-226 IgG1 |
|---|---|
| Ng 7-13 1µM_x000d_ | 96.0 |
| Ng 7-13 3µM_x000d_ | 28.0 |
| Ng 9-9 1 µM_x000d_ | 0.0 |
| Ng 9-9 3µM_x000d_ | 595.3333333333334 |
| Ng 9-9 10µM_x000d_ | 1619.0 |
| Ng 10-13 1 µM_x000d_ | 0.0 |
| Ng 10-13 3 µM_x000d_ | 0.0 |
| Ng 10-13 10 µM_x000d_ | 0.0 |
| Ng 10-13 30 µM_x000d_ | 1060.3333333333333 |
| Ng 10-10 1 µM_x000d_ | 0.0 |
| Ng 10-10 3 µM_x000d_ | 14.333333333333343 |
| Ng 10-11 1 µM_x000d_ | 0.0 |
| Ng 10-11 3 µM_x000d_ | 14.666666666666657 |
| Ng 10-12 1 µM_x000d_ | 66.0 |
| Ng 11-10 1 µM_x000d_ | 0.0 |
| Ng 11-10 3 µM_x000d_ | 0.0 |
| Ng 11-11 1 µM_x000d_ | 0.0 |
| Ng 11-11 3µM_x000d_ | 0.0 |
| Ng 11-11 10µM_x000d_ | 0.0 |
| Ng 12-11 1 µM_x000d_ | 0.0 |
| Ng 12-11 3 µM_x000d_ | 0.0 |
| Ng 13-15 1 µM_x000d_ | 0.0 |
| Ng 13-15 3 µM_x000d_ | 4.333333333333343 |
| Ng 13-14_x000d_ | 68.33333333333334 |
| Ng 14-10 1 µM_x000d_ | 93.66666666666666 |
| Ng 14-10 3 µM_x000d_ | 751.0 |
| Ng 14-11 1 µM_x000d_ | 0.0 |
| Ng 14-12 1 µM_x000d_ | 1346.6666666666667 |
| Ng 14-7 1 µM_x000d_ | 0.0 |
| Ng 15-14/15 1 µM_x000d_ | 142.0 |
| Ng 15-12 1µM_x000d_ | 0.0 |
| Ng 17-7 1 µM_x000d_ | 0.0 |
| Ng 17-7 3 µM_x000d_ | 0.0 |
| Ng 17-7 10 µM_x000d_ | 0.0 |
| Ng 19-9 1 µM_x000d_ | 0.0 |
| Ng 19-9 3 µM_x000d_ | 0.0 |
| Ng 19-9 10 µM_x000d_ | 0.0 |
| Ng 20-7 1 µM_x000d_ | 0.0 |
| Ng 20-7 3 µM_x000d_ | 0.0 |
| Ng 20-7 10 µM_x000d_ | 19.0 |
| Ng 21-8 1 µM_x000d_ | 0.0 |
| Ng 21-8 3 µM_x000d_ | 0.0 |
| Ng 21-8 10 µM_x000d_ | 0.0 |
| Ng 15-13 3µM_x000d_ | 39.33333333333334 |
| Ng 16-12 1 µM_x000d_ | 0.0 |
| Ng 17-9 1 µM_x000d_ | 0.0 |
| Ng 17-9 3 µM_x000d_ | 0.0 |
| Ng 18-11 1 µM_x000d_ | 0.3333333333333428 |
| Ng 18-11 3 µM_x000d_ | 0.0 |
| Ng 20-8 1 µM_x000d_ | 23.666666666666657 |
| Ng 20-9 1 µM_x000d_ | 3.666666666666657 |
| Ng 20-9 3 µM_x000d_ | 440.66666666666663 |
| Ng 21-9 1 µM_x000d_ | 0.0 |
| Ng 13-13 3 µM_x000d_ | 0.0 |
| Ng 14-8 1 µM_x000d_ | 0.0 |
| Ng 14-9 1 µM_x000d_ | 0.0 |
| Ng 14-9 3 µM_x000d_ | 0.0 |
| Ng 14-9 10 µM_x000d_ | 13.0 |
| Ng 14-9 30 µM_x000d_ | 11.0 |
| Ng 15-10 1µM_x000d_ | 0.0 |
| Ng 16-9 1 µM_x000d_ | 0.0 |
| Ng 17-8 1 µM_x000d_ | 0.0 |
| Ng 17-8 3 µM_x000d_ | 0.0 |
| Ng 18-9 1µM_x000d_ | 0.0 |
| Ng 19-11 1 µM_x000d_ | 392.66666666666663 |
| Ng 19-11 3 µM_x000d_ | 781.3333333333334 |
| Lg 17-15 HF 10 µM_x000d_ | 171.0 |
| Lg 15-17 1 µM_x000d_ | 0.0 |
| Lg 17-15 1 µM_x000d_ | 0.0 |
| Lg 17-15 3 µM_x000d_ | 88.33333333333334 |
| Lg 17-15 10 µM_x000d_ | 1481.6666666666667 |
| Lg 17-17 1 µM_x000d_ | 0.0 |
| Lg 17-16 1 µM_x000d_ | 1325.6666666666667 |
| Lg 16-17 3 µM_x000d_ | 2739.0 |
| Lg 18-8 1 µM_x000d_ | 0.0 |
| Lg 19-8 1 µM_x000d_ | 15.333333333333343 |
| Lg 19-8 3 µM_x000d_ | 2708.3333333333335 |
| Lg 19-9 1 µM_x000d_ | 32.0 |
| Lg 20-10_x000d_ | 0.0 |
| Lg 22-12 1 µM_x000d_ | 0.0 |
| Lg 20-9 1 µM_x000d_ | 0.0 |
| Lg 23-11 1 µM_x000d_ | 0.0 |
| Lg 23-11 3 µM_x000d_ | 5.666666666666657 |
| Lg 23-12_x000d_ | 417.0 |
| Lg 26-8_x000d_ | 2.666666666666657 |
| Lg 26-9_x000d_ | 0.0 |
| Lg 22-13_x000d_ | 1926.6666666666665 |
| Lg 26-10_x000d_ | 0.0 |
| Lg 18-7-F3 HF 1uM | 0.0 |
| Lg 19-8-F5 HF 3uM | 45.0 |
| Lg 23-11-F2 HF 3uM | 0.0 |
| Blank 1 | 0.0 |
| Blank 2 | 0.0 |
| Blank 3 | 0.0 |
| Blank 4 | 0.0 |
| Blank 5 | 0.0 |MFI
### Chart: Un-NL1
| Category | NLD-57 IgG1 |
|---|---|
| Ng 7-13 1µM_x000d_ | 0.0 |
| Ng 7-13 3µM_x000d_ | 0.0 |
| Ng 9-9 1 µM_x000d_ | 0.0 |
| Ng 9-9 3µM_x000d_ | 0.0 |
| Ng 9-9 10µM_x000d_ | 0.0 |
| Ng 10-13 1 µM_x000d_ | 0.0 |
| Ng 10-13 3 µM_x000d_ | 0.0 |
| Ng 10-13 10 µM_x000d_ | 0.0 |
| Ng 10-13 30 µM_x000d_ | 0.0 |
| Ng 10-10 1 µM_x000d_ | 0.0 |
| Ng 10-10 3 µM_x000d_ | 0.0 |
| Ng 10-11 1 µM_x000d_ | 0.0 |
| Ng 10-11 3 µM_x000d_ | 0.0 |
| Ng 10-12 1 µM_x000d_ | 0.0 |
| Ng 11-10 1 µM_x000d_ | 0.0 |
| Ng 11-10 3 µM_x000d_ | 0.0 |
| Ng 11-11 1 µM_x000d_ | 0.0 |
| Ng 11-11 3µM_x000d_ | 0.0 |
| Ng 11-11 10µM_x000d_ | 0.0 |
| Ng 12-11 1 µM_x000d_ | 0.0 |
| Ng 12-11 3 µM_x000d_ | 0.0 |
| Ng 13-15 1 µM_x000d_ | 0.0 |
| Ng 13-15 3 µM_x000d_ | 0.0 |
| Ng 13-14_x000d_ | 0.0 |
| Ng 14-10 1 µM_x000d_ | 0.0 |
| Ng 14-10 3 µM_x000d_ | 0.0 |
| Ng 14-11 1 µM_x000d_ | 0.0 |
| Ng 14-12 1 µM_x000d_ | 0.0 |
| Ng 14-7 1 µM_x000d_ | 0.0 |
| Ng 15-14/15 1 µM_x000d_ | 0.0 |
| Ng 15-12 1µM_x000d_ | 0.0 |
| Ng 17-7 1 µM_x000d_ | 0.0 |
| Ng 17-7 3 µM_x000d_ | 0.0 |
| Ng 17-7 10 µM_x000d_ | 0.0 |
| Ng 19-9 1 µM_x000d_ | 0.0 |
| Ng 19-9 3 µM_x000d_ | 0.0 |
| Ng 19-9 10 µM_x000d_ | 0.0 |
| Ng 20-7 1 µM_x000d_ | 0.0 |
| Ng 20-7 3 µM_x000d_ | 0.0 |
| Ng 20-7 10 µM_x000d_ | 0.0 |
| Ng 21-8 1 µM_x000d_ | 0.0 |
| Ng 21-8 3 µM_x000d_ | 0.0 |
| Ng 21-8 10 µM_x000d_ | 0.0 |
| Ng 15-13 3µM_x000d_ | 0.0 |
| Ng 16-12 1 µM_x000d_ | 0.0 |
| Ng 17-9 1 µM_x000d_ | 0.0 |
| Ng 17-9 3 µM_x000d_ | 0.0 |
| Ng 18-11 1 µM_x000d_ | 0.0 |
| Ng 18-11 3 µM_x000d_ | 0.0 |
| Ng 20-8 1 µM_x000d_ | 0.0 |
| Ng 20-9 1 µM_x000d_ | 0.0 |
| Ng 20-9 3 µM_x000d_ | 0.0 |
| Ng 21-9 1 µM_x000d_ | 0.0 |
| Ng 13-13 3 µM_x000d_ | 0.0 |
| Ng 14-8 1 µM_x000d_ | 0.0 |
| Ng 14-9 1 µM_x000d_ | 30.0 |
| Ng 14-9 3 µM_x000d_ | 0.0 |
| Ng 14-9 10 µM_x000d_ | 0.0 |
| Ng 14-9 30 µM_x000d_ | 0.0 |
| Ng 15-10 1µM_x000d_ | 0.0 |
| Ng 16-9 1 µM_x000d_ | 0.0 |
| Ng 17-8 1 µM_x000d_ | 0.0 |
| Ng 17-8 3 µM_x000d_ | 0.0 |
| Ng 18-9 1µM_x000d_ | 0.0 |
| Ng 19-11 1 µM_x000d_ | 0.0 |
| Ng 19-11 3 µM_x000d_ | 0.0 |
| Lg 17-15 HF 10 µM_x000d_ | 0.0 |
| Lg 15-17 1 µM_x000d_ | 0.0 |
| Lg 17-15 1 µM_x000d_ | 0.0 |
| Lg 17-15 3 µM_x000d_ | 103.33333333333331 |
| Lg 17-15 10 µM_x000d_ | 0.0 |
| Lg 17-17 1 µM_x000d_ | 0.0 |
| Lg 17-16 1 µM_x000d_ | 0.0 |
| Lg 16-17 3 µM_x000d_ | 0.0 |
| Lg 18-8 1 µM_x000d_ | 75.66666666666663 |
| Lg 19-8 1 µM_x000d_ | 77.83333333333331 |
| Lg 19-8 3 µM_x000d_ | 102.66666666666663 |
| Lg 19-9 1 µM_x000d_ | 65.0 |
| Lg 20-10_x000d_ | 0.0 |
| Lg 22-12 1 µM_x000d_ | 157.0 |
| Lg 20-9 1 µM_x000d_ | 54.333333333333314 |
| Lg 23-11 1 µM_x000d_ | 0.0 |
| Lg 23-11 3 µM_x000d_ | 70.66666666666663 |
| Lg 23-12_x000d_ | 0.0 |
| Lg 26-8_x000d_ | 0.0 |
| Lg 26-9_x000d_ | 101.0 |
| Lg 22-13_x000d_ | 55.66666666666663 |
| Lg 26-10_x000d_ | 0.0 |
| Lg 18-7-F3 HF 1uM | 0.0 |
| Lg 19-8-F5 HF 3uM | 14.666666666666629 |
| Lg 23-11-F2 HF 3uM | 0.0 |
| Blank 1 | 0.0 |
| Blank 2 | 46.333333333333314 |
| Blank 3 | 103.0 |
| Blank 4 | 0.0 |
| Blank 5 | 0.0 |MFI
### Chart: Un-NL2
| Category | NLD-59 IgG1 |
|---|---|
| Ng 7-13 1µM_x000d_ | 12.333333333333343 |
| Ng 7-13 3µM_x000d_ | 2.666666666666657 |
| Ng 9-9 1 µM_x000d_ | 0.0 |
| Ng 9-9 3µM_x000d_ | 0.0 |
| Ng 9-9 10µM_x000d_ | 120.66666666666669 |
| Ng 10-13 1 µM_x000d_ | 0.0 |
| Ng 10-13 3 µM_x000d_ | 0.0 |
| Ng 10-13 10 µM_x000d_ | 0.0 |
| Ng 10-13 30 µM_x000d_ | 89.0 |
| Ng 10-10 1 µM_x000d_ | 0.0 |
| Ng 10-10 3 µM_x000d_ | 4.0 |
| Ng 10-11 1 µM_x000d_ | 0.0 |
| Ng 10-11 3 µM_x000d_ | 0.0 |
| Ng 10-12 1 µM_x000d_ | 7.666666666666657 |
| Ng 11-10 1 µM_x000d_ | 0.0 |
| Ng 11-10 3 µM_x000d_ | 0.0 |
| Ng 11-11 1 µM_x000d_ | 0.0 |
| Ng 11-11 3µM_x000d_ | 0.0 |
| Ng 11-11 10µM_x000d_ | 0.0 |
| Ng 12-11 1 µM_x000d_ | 0.0 |
| Ng 12-11 3 µM_x000d_ | 0.0 |
| Ng 13-15 1 µM_x000d_ | 0.0 |
| Ng 13-15 3 µM_x000d_ | 3.0 |
| Ng 13-14_x000d_ | 283.6666666666667 |
| Ng 14-10 1 µM_x000d_ | 0.0 |
| Ng 14-10 3 µM_x000d_ | 0.0 |
| Ng 14-11 1 µM_x000d_ | 0.0 |
| Ng 14-12 1 µM_x000d_ | 0.0 |
| Ng 14-7 1 µM_x000d_ | 0.0 |
| Ng 15-14/15 1 µM_x000d_ | 0.0 |
| Ng 15-12 1µM_x000d_ | 0.0 |
| Ng 17-7 1 µM_x000d_ | 0.0 |
| Ng 17-7 3 µM_x000d_ | 0.0 |
| Ng 17-7 10 µM_x000d_ | 68.0 |
| Ng 19-9 1 µM_x000d_ | 0.0 |
| Ng 19-9 3 µM_x000d_ | 0.0 |
| Ng 19-9 10 µM_x000d_ | 0.0 |
| Ng 20-7 1 µM_x000d_ | 0.0 |
| Ng 20-7 3 µM_x000d_ | 0.0 |
| Ng 20-7 10 µM_x000d_ | 35.0 |
| Ng 21-8 1 µM_x000d_ | 0.0 |
| Ng 21-8 3 µM_x000d_ | 0.0 |
| Ng 21-8 10 µM_x000d_ | 0.0 |
| Ng 15-13 3µM_x000d_ | 91.66666666666666 |
| Ng 16-12 1 µM_x000d_ | 0.0 |
| Ng 17-9 1 µM_x000d_ | 0.0 |
| Ng 17-9 3 µM_x000d_ | 0.0 |
| Ng 18-11 1 µM_x000d_ | 0.0 |
| Ng 18-11 3 µM_x000d_ | 41.66666666666666 |
| Ng 20-8 1 µM_x000d_ | 0.0 |
| Ng 20-9 1 µM_x000d_ | 0.0 |
| Ng 20-9 3 µM_x000d_ | 0.3333333333333428 |
| Ng 21-9 1 µM_x000d_ | 0.0 |
| Ng 13-13 3 µM_x000d_ | 0.0 |
| Ng 14-8 1 µM_x000d_ | 0.0 |
| Ng 14-9 1 µM_x000d_ | 0.0 |
| Ng 14-9 3 µM_x000d_ | 0.0 |
| Ng 14-9 10 µM_x000d_ | 73.0 |
| Ng 14-9 30 µM_x000d_ | 3.333333333333343 |
| Ng 15-10 1µM_x000d_ | 0.0 |
| Ng 16-9 1 µM_x000d_ | 0.0 |
| Ng 17-8 1 µM_x000d_ | 0.0 |
| Ng 17-8 3 µM_x000d_ | 0.0 |
| Ng 18-9 1µM_x000d_ | 0.0 |
| Ng 19-11 1 µM_x000d_ | 0.0 |
| Ng 19-11 3 µM_x000d_ | 0.0 |
| Lg 17-15 HF 10 µM_x000d_ | 0.0 |
| Lg 15-17 1 µM_x000d_ | 0.0 |
| Lg 17-15 1 µM_x000d_ | 0.0 |
| Lg 17-15 3 µM_x000d_ | 0.0 |
| Lg 17-15 10 µM_x000d_ | 0.0 |
| Lg 17-17 1 µM_x000d_ | 0.0 |
| Lg 17-16 1 µM_x000d_ | 0.0 |
| Lg 16-17 3 µM_x000d_ | 0.0 |
| Lg 18-8 1 µM_x000d_ | 0.0 |
| Lg 19-8 1 µM_x000d_ | 0.0 |
| Lg 19-8 3 µM_x000d_ | 0.0 |
| Lg 19-9 1 µM_x000d_ | 0.0 |
| Lg 20-10_x000d_ | 0.0 |
| Lg 22-12 1 µM_x000d_ | 0.0 |
| Lg 20-9 1 µM_x000d_ | 0.0 |
| Lg 23-11 1 µM_x000d_ | 0.0 |
| Lg 23-11 3 µM_x000d_ | 0.0 |
| Lg 23-12_x000d_ | 0.0 |
| Lg 26-8_x000d_ | 0.0 |
| Lg 26-9_x000d_ | 0.0 |
| Lg 22-13_x000d_ | 322.6666666666667 |
| Lg 26-10_x000d_ | 0.0 |
| Lg 18-7-F3 HF 1uM | 0.0 |
| Lg 19-8-F5 HF 3uM | 0.0 |
| Lg 23-11-F2 HF 3uM | 0.0 |
| Blank 1 | 0.0 |
| Blank 2 | 0.0 |
| Blank 3 | 0.0 |
| Blank 4 | 0.0 |
| Blank 5 | 0.0 |MFI
### Chart: Un-NL3
| Category | NLD-60 IgG1 |
|---|---|
| Ng 7-13 1µM_x000d_ | 0.0 |
| Ng 7-13 3µM_x000d_ | 47.33333333333337 |
| Ng 9-9 1 µM_x000d_ | 0.0 |
| Ng 9-9 3µM_x000d_ | 0.0 |
| Ng 9-9 10µM_x000d_ | 222.00000000000006 |
| Ng 10-13 1 µM_x000d_ | 0.0 |
| Ng 10-13 3 µM_x000d_ | 0.0 |
| Ng 10-13 10 µM_x000d_ | 0.0 |
| Ng 10-13 30 µM_x000d_ | 0.0 |
| Ng 10-10 1 µM_x000d_ | 0.0 |
| Ng 10-10 3 µM_x000d_ | 0.0 |
| Ng 10-11 1 µM_x000d_ | 0.0 |
| Ng 10-11 3 µM_x000d_ | 0.0 |
| Ng 10-12 1 µM_x000d_ | 0.0 |
| Ng 11-10 1 µM_x000d_ | 0.0 |
| Ng 11-10 3 µM_x000d_ | 0.0 |
| Ng 11-11 1 µM_x000d_ | 0.0 |
| Ng 11-11 3µM_x000d_ | 0.0 |
| Ng 11-11 10µM_x000d_ | 0.0 |
| Ng 12-11 1 µM_x000d_ | 0.0 |
| Ng 12-11 3 µM_x000d_ | 0.0 |
| Ng 13-15 1 µM_x000d_ | 0.0 |
| Ng 13-15 3 µM_x000d_ | 0.0 |
| Ng 13-14_x000d_ | 0.0 |
| Ng 14-10 1 µM_x000d_ | 0.0 |
| Ng 14-10 3 µM_x000d_ | 0.0 |
| Ng 14-11 1 µM_x000d_ | 0.0 |
| Ng 14-12 1 µM_x000d_ | 0.0 |
| Ng 14-7 1 µM_x000d_ | 0.0 |
| Ng 15-14/15 1 µM_x000d_ | 0.0 |
| Ng 15-12 1µM_x000d_ | 0.0 |
| Ng 17-7 1 µM_x000d_ | 118.66666666666669 |
| Ng 17-7 3 µM_x000d_ | 0.0 |
| Ng 17-7 10 µM_x000d_ | 0.0 |
| Ng 19-9 1 µM_x000d_ | 1.0 |
| Ng 19-9 3 µM_x000d_ | 73.33333333333337 |
| Ng 19-9 10 µM_x000d_ | 0.0 |
| Ng 20-7 1 µM_x000d_ | 0.0 |
| Ng 20-7 3 µM_x000d_ | 0.0 |
| Ng 20-7 10 µM_x000d_ | 0.0 |
| Ng 21-8 1 µM_x000d_ | 0.0 |
| Ng 21-8 3 µM_x000d_ | 0.0 |
| Ng 21-8 10 µM_x000d_ | 0.0 |
| Ng 15-13 3µM_x000d_ | 0.0 |
| Ng 16-12 1 µM_x000d_ | 0.0 |
| Ng 17-9 1 µM_x000d_ | 0.0 |
| Ng 17-9 3 µM_x000d_ | 0.0 |
| Ng 18-11 1 µM_x000d_ | 34.33333333333337 |
| Ng 18-11 3 µM_x000d_ | 0.0 |
| Ng 20-8 1 µM_x000d_ | 0.0 |
| Ng 20-9 1 µM_x000d_ | 135.33333333333331 |
| Ng 20-9 3 µM_x000d_ | 84.33333333333337 |
| Ng 21-9 1 µM_x000d_ | 0.0 |
| Ng 13-13 3 µM_x000d_ | 0.0 |
| Ng 14-8 1 µM_x000d_ | 0.0 |
| Ng 14-9 1 µM_x000d_ | 0.0 |
| Ng 14-9 3 µM_x000d_ | 78.33333333333337 |
| Ng 14-9 10 µM_x000d_ | 0.0 |
| Ng 14-9 30 µM_x000d_ | 0.0 |
| Ng 15-10 1µM_x000d_ | 0.0 |
| Ng 16-9 1 µM_x000d_ | 0.0 |
| Ng 17-8 1 µM_x000d_ | 32.0 |
| Ng 17-8 3 µM_x000d_ | 307.00000000000006 |
| Ng 18-9 1µM_x000d_ | 0.0 |
| Ng 19-11 1 µM_x000d_ | 0.0 |
| Ng 19-11 3 µM_x000d_ | 64.0 |
| Lg 17-15 HF 10 µM_x000d_ | 0.0 |
| Lg 15-17 1 µM_x000d_ | 0.0 |
| Lg 17-15 1 µM_x000d_ | 0.0 |
| Lg 17-15 3 µM_x000d_ | 0.0 |
| Lg 17-15 10 µM_x000d_ | 0.0 |
| Lg 17-17 1 µM_x000d_ | 0.0 |
| Lg 17-16 1 µM_x000d_ | 0.0 |
| Lg 16-17 3 µM_x000d_ | 0.0 |
| Lg 18-8 1 µM_x000d_ | 0.0 |
| Lg 19-8 1 µM_x000d_ | 0.0 |
| Lg 19-8 3 µM_x000d_ | 0.0 |
| Lg 19-9 1 µM_x000d_ | 0.0 |
| Lg 20-10_x000d_ | 0.0 |
| Lg 22-12 1 µM_x000d_ | 0.0 |
| Lg 20-9 1 µM_x000d_ | 0.0 |
| Lg 23-11 1 µM_x000d_ | 0.0 |
| Lg 23-11 3 µM_x000d_ | 0.0 |
| Lg 23-12_x000d_ | 0.0 |
| Lg 26-8_x000d_ | 0.0 |
| Lg 26-9_x000d_ | 0.0 |
| Lg 22-13_x000d_ | 0.0 |
| Lg 26-10_x000d_ | 0.0 |
| Lg 18-7-F3 HF 1uM | 0.0 |
| Lg 19-8-F5 HF 3uM | 0.0 |
| Lg 23-11-F2 HF 3uM | 0.0 |
| Blank 1 | 0.0 |
| Blank 2 | 0.0 |
| Blank 3 | 0.0 |
| Blank 4 | 0.0 |
| Blank 5 | 0.0 |MFI
### Chart: Un-NL4
| Category | NLD-61 IgG1 |
|---|---|
| Ng 7-13 1µM_x000d_ | 0.0 |
| Ng 7-13 3µM_x000d_ | 16.0 |
| Ng 9-9 1 µM_x000d_ | 0.0 |
| Ng 9-9 3µM_x000d_ | 412.33333333333337 |
| Ng 9-9 10µM_x000d_ | 0.0 |
| Ng 10-13 1 µM_x000d_ | 0.0 |
| Ng 10-13 3 µM_x000d_ | 0.0 |
| Ng 10-13 10 µM_x000d_ | 0.0 |
| Ng 10-13 30 µM_x000d_ | 0.0 |
| Ng 10-10 1 µM_x000d_ | 0.0 |
| Ng 10-10 3 µM_x000d_ | 0.0 |
| Ng 10-11 1 µM_x000d_ | 0.0 |
| Ng 10-11 3 µM_x000d_ | 1.0 |
| Ng 10-12 1 µM_x000d_ | 0.0 |
| Ng 11-10 1 µM_x000d_ | 0.0 |
| Ng 11-10 3 µM_x000d_ | 0.0 |
| Ng 11-11 1 µM_x000d_ | 0.0 |
| Ng 11-11 3µM_x000d_ | 0.0 |
| Ng 11-11 10µM_x000d_ | 0.0 |
| Ng 12-11 1 µM_x000d_ | 0.0 |
| Ng 12-11 3 µM_x000d_ | 0.0 |
| Ng 13-15 1 µM_x000d_ | 0.0 |
| Ng 13-15 3 µM_x000d_ | 0.0 |
| Ng 13-14_x000d_ | 205.66666666666669 |
| Ng 14-10 1 µM_x000d_ | 0.0 |
| Ng 14-10 3 µM_x000d_ | 0.0 |
| Ng 14-11 1 µM_x000d_ | 0.0 |
| Ng 14-12 1 µM_x000d_ | 0.0 |
| Ng 14-7 1 µM_x000d_ | 0.0 |
| Ng 15-14/15 1 µM_x000d_ | 0.0 |
| Ng 15-12 1µM_x000d_ | 0.0 |
| Ng 17-7 1 µM_x000d_ | 0.0 |
| Ng 17-7 3 µM_x000d_ | 0.0 |
| Ng 17-7 10 µM_x000d_ | 0.0 |
| Ng 19-9 1 µM_x000d_ | 0.0 |
| Ng 19-9 3 µM_x000d_ | 0.0 |
| Ng 19-9 10 µM_x000d_ | 0.0 |
| Ng 20-7 1 µM_x000d_ | 0.0 |
| Ng 20-7 3 µM_x000d_ | 0.0 |
| Ng 20-7 10 µM_x000d_ | 0.0 |
| Ng 21-8 1 µM_x000d_ | 0.0 |
| Ng 21-8 3 µM_x000d_ | 0.0 |
| Ng 21-8 10 µM_x000d_ | 0.0 |
| Ng 15-13 3µM_x000d_ | 2.333333333333343 |
| Ng 16-12 1 µM_x000d_ | 0.0 |
| Ng 17-9 1 µM_x000d_ | 0.0 |
| Ng 17-9 3 µM_x000d_ | 0.0 |
| Ng 18-11 1 µM_x000d_ | 0.0 |
| Ng 18-11 3 µM_x000d_ | 0.0 |
| Ng 20-8 1 µM_x000d_ | 0.0 |
| Ng 20-9 1 µM_x000d_ | 0.0 |
| Ng 20-9 3 µM_x000d_ | 0.0 |
| Ng 21-9 1 µM_x000d_ | 0.0 |
| Ng 13-13 3 µM_x000d_ | 0.0 |
| Ng 14-8 1 µM_x000d_ | 0.0 |
| Ng 14-9 1 µM_x000d_ | 0.0 |
| Ng 14-9 3 µM_x000d_ | 0.0 |
| Ng 14-9 10 µM_x000d_ | 11.333333333333343 |
| Ng 14-9 30 µM_x000d_ | 0.0 |
| Ng 15-10 1µM_x000d_ | 0.0 |
| Ng 16-9 1 µM_x000d_ | 0.0 |
| Ng 17-8 1 µM_x000d_ | 0.0 |
| Ng 17-8 3 µM_x000d_ | 0.0 |
| Ng 18-9 1µM_x000d_ | 0.0 |
| Ng 19-11 1 µM_x000d_ | 0.0 |
| Ng 19-11 3 µM_x000d_ | 0.0 |
| Lg 17-15 HF 10 µM_x000d_ | 0.0 |
| Lg 15-17 1 µM_x000d_ | 0.0 |
| Lg 17-15 1 µM_x000d_ | 0.0 |
| Lg 17-15 3 µM_x000d_ | 0.0 |
| Lg 17-15 10 µM_x000d_ | 5.666666666666657 |
| Lg 17-17 1 µM_x000d_ | 0.0 |
| Lg 17-16 1 µM_x000d_ | 0.0 |
| Lg 16-17 3 µM_x000d_ | 158.66666666666669 |
| Lg 18-8 1 µM_x000d_ | 0.0 |
| Lg 19-8 1 µM_x000d_ | 0.0 |
| Lg 19-8 3 µM_x000d_ | 0.0 |
| Lg 19-9 1 µM_x000d_ | 0.0 |
| Lg 20-10_x000d_ | 0.0 |
| Lg 22-12 1 µM_x000d_ | 0.0 |
| Lg 20-9 1 µM_x000d_ | 0.0 |
| Lg 23-11 1 µM_x000d_ | 0.0 |
| Lg 23-11 3 µM_x000d_ | 0.0 |
| Lg 23-12_x000d_ | 0.0 |
| Lg 26-8_x000d_ | 0.0 |
| Lg 26-9_x000d_ | 0.0 |
| Lg 22-13_x000d_ | 0.0 |
| Lg 26-10_x000d_ | 0.0 |
| Lg 18-7-F3 HF 1uM | 0.0 |
| Lg 19-8-F5 HF 3uM | 0.0 |
| Lg 23-11-F2 HF 3uM | 0.0 |
| Blank 1 | 0.0 |
| Blank 2 | 0.0 |
| Blank 3 | 0.0 |
| Blank 4 | 0.0 |
| Blank 5 | 0.0 |MFI
### Chart: Un-NL5
| Category | NLD-62 IgG1 |
|---|---|
| Ng1_1uM
 | 0.0 |
| Ng1_3uM
 | 20.666666666666657 |
| Ng2_1uM | 0.0 |
| Ng2_3uM | 203.33333333333331 |
| Ng2_10uM | 0.3333333333333428 |
| Ng3_1uM | 0.0 |
| Ng3_3uM | 0.0 |
| Ng3_10uM | 8.666666666666657 |
| Ng3_30uM | 0.0 |
| Ng4_1uM | 0.0 |
| Ng4_3uM | 17.0 |
| Ng5_1uM | 0.0 |
| Ng5_3uM | 41.0 |
| Ng6_1uM | 0.0 |
| Ng7_1uM | 0.0 |
| Ng7_3uM | 0.0 |
| Ng8_1uM | 0.0 |
| Ng8_3uM | 0.0 |
| Ng8_10uM | 5.0 |
| Ng9_1uM | 0.0 |
| Ng9_3uM | 47.0 |
| Ng10_3uM | 0.0 |
| Ng10_1uM | 31.0 |
| Ng11 | 14.333333333333343 |
| Ng12_1uM | 0.0 |
| Ng12_3uM | 6.666666666666657 |
| Ng13_1uM | 0.0 |
| Ng14_1uM | 0.0 |
| Ng15_1uM | 0.0 |
| Ng16_1uM | 0.0 |
| Ng17_1uM | 0.0 |
| Ng18_1uM | 0.0 |
| Ng18_3uM | 0.6666666666666572 |
| Ng18_10uM | 84.66666666666666 |
| Ng19_1uM | 0.0 |
| Ng19_3uM | 21.0 |
| Ng19_10uM | 36.0 |
| Ng20_1uM | 0.0 |
| Ng20_3uM | 0.0 |
| Ng20_10uM | 52.33333333333334 |
| Ng21_1uM | 0.0 |
| Ng21 _3uM | 0.0 |
| Ng21_10uM | 0.0 |
| Ng22_1uM | 70.66666666666666 |
| Ng23_1uM | 0.0 |
| Ng24_1uM | 0.0 |
| Ng24_3uM | 12.333333333333343 |
| Ng25_1uM | 23.0 |
| Ng25_3uM | 60.33333333333334 |
| Ng26_1uM | 0.0 |
| Ng27_1uM | 0.0 |
| Ng27_3uM | 29.0 |
| Ng28_1uM | 0.0 |
| Ng29_1uM | 1.6666666666666572 |
| Ng30_1uM | 0.0 |
| Ng31_1uM | 0.0 |
| Ng31_3uM | 35.66666666666666 |
| Ng31_10uM | 67.66666666666666 |
| Ng31_30uM | 46.66666666666666 |
| Ng32_1uM | 0.0 |
| Ng33_1uM | 0.0 |
| Ng34_1uM | 0.0 |
| Ng34_3uM | 37.33333333333334 |
| Ng35_1uM | 0.0 |
| Ng36_1uM | 37.66666666666666 |
| Ng36_3uM | 0.0 |
| Lg2_HF_10uM | 7.0 |
| Lg1_1uM | 0.0 |
| Lg2_1uM | 0.0 |
| Lg2_3uM | 0.0 |
| Lg2_10uM | 0.0 |
| Lg3_1uM | 0.0 |
| Lg4_1uM | 0.0 |
| Lg5_3uM | 274.0 |
| Lg6_1uM | 0.0 |
| Lg7_1uM | 0.0 |
| Lg7_3uM | 0.0 |
| Lg8_1uM | 0.0 |
| Lg9 | 0.0 |
| Lg10_1uM | 0.0 |
| Lg11_1uM | 0.0 |
| Lg12_1uM | 0.0 |
| Lg12_3uM | 0.0 |
| Lg13 | 0.0 |
| Lg14 | 0.0 |
| Lg15 | 0.0 |
| Lg16 | 0.0 |
| Lg17 | 0.0 |
| Lg18_HF_1uM | 0.0 |
| Lg7_HF_3uM | 0.0 |
| Lg12_HF_3uM | 0.0 |
| Blank 1 | 5.0 |
| Blank 2 | 0.0 |
| Blank 3 | 0.0 |
| Blank 4 | 0.0 |
| Blank 5 | 0.0 |MFI

## Slide 35
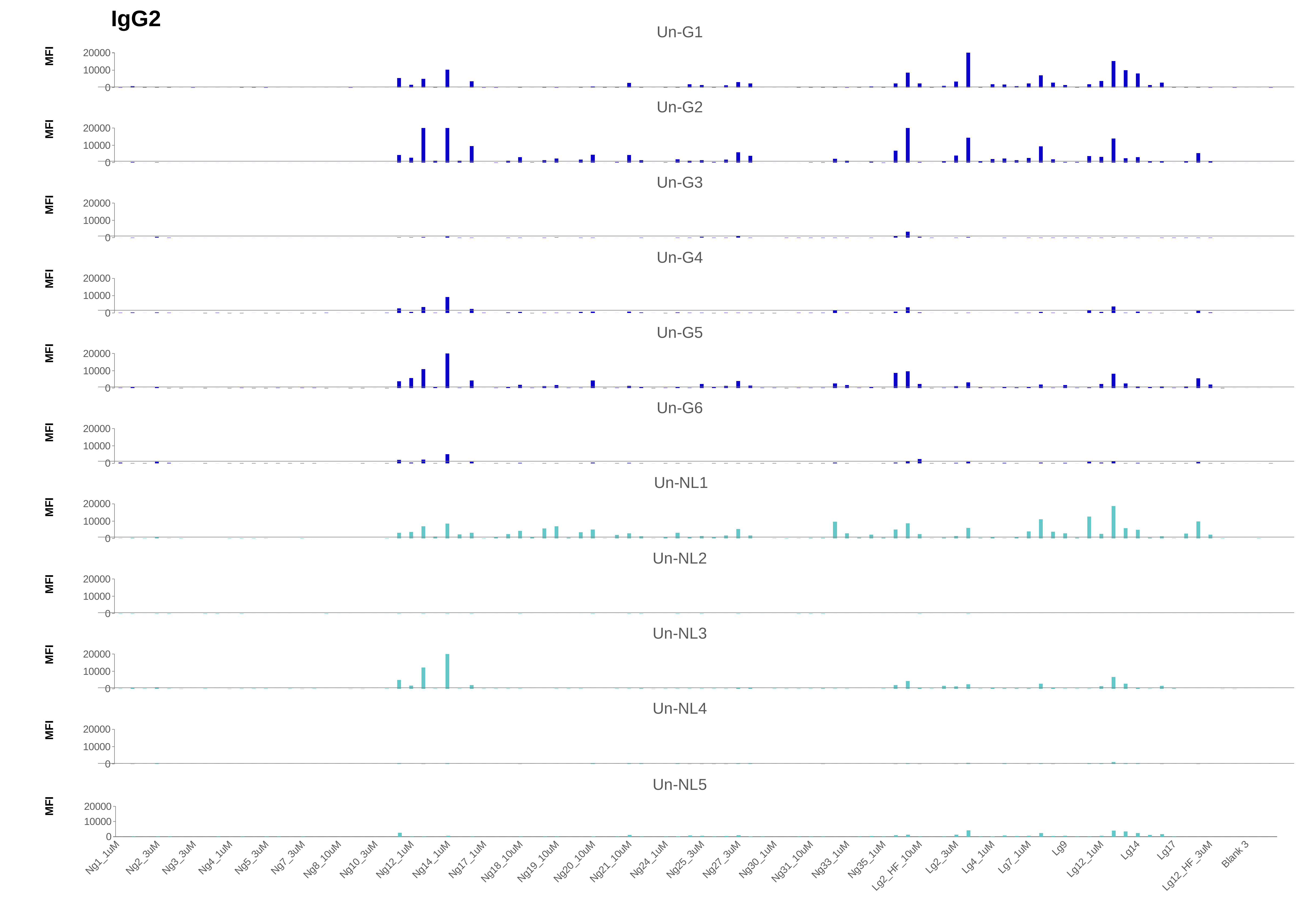

IgG2
### Chart: Un-G1
| Category | UG 000-214 IgG2 |
|---|---|
| Ng 7-13 1µM_x000d_ | 17.333333333333343 |
| Ng 7-13 3µM_x000d_ | 722.0 |
| Ng 9-9 1 µM_x000d_ | 165.33333333333331 |
| Ng 9-9 3µM_x000d_ | 278.3333333333333 |
| Ng 9-9 10µM_x000d_ | 74.66666666666666 |
| Ng 10-13 1 µM_x000d_ | 0.0 |
| Ng 10-13 3 µM_x000d_ | 19.0 |
| Ng 10-13 10 µM_x000d_ | 0.0 |
| Ng 10-13 30 µM_x000d_ | 0.0 |
| Ng 10-10 1 µM_x000d_ | 0.0 |
| Ng 10-10 3 µM_x000d_ | 68.0 |
| Ng 10-11 1 µM_x000d_ | 29.333333333333343 |
| Ng 10-11 3 µM_x000d_ | 18.666666666666657 |
| Ng 10-12 1 µM_x000d_ | 0.0 |
| Ng 11-10 1 µM_x000d_ | 0.0 |
| Ng 11-10 3 µM_x000d_ | 0.0 |
| Ng 11-11 1 µM_x000d_ | 0.0 |
| Ng 11-11 3µM_x000d_ | 0.0 |
| Ng 11-11 10µM_x000d_ | 0.0 |
| Ng 12-11 1 µM_x000d_ | 23.333333333333343 |
| Ng 12-11 3 µM_x000d_ | 0.0 |
| Ng 13-15 1 µM_x000d_ | 0.0 |
| Ng 13-15 3 µM_x000d_ | 0.0 |
| Ng 13-14_x000d_ | 5379.333333333333 |
| Ng 14-10 1 µM_x000d_ | 1527.6666666666667 |
| Ng 14-10 3 µM_x000d_ | 4916.0 |
| Ng 14-11 1 µM_x000d_ | 300.0 |
| Ng 14-12 1 µM_x000d_ | 10203.0 |
| Ng 14-7 1 µM_x000d_ | 0.0 |
| Ng 15-14/15 1 µM_x000d_ | 3492.0 |
| Ng 15-12 1µM_x000d_ | 8.333333333333343 |
| Ng 17-7 1 µM_x000d_ | 26.666666666666657 |
| Ng 17-7 3 µM_x000d_ | 0.0 |
| Ng 17-7 10 µM_x000d_ | 46.0 |
| Ng 19-9 1 µM_x000d_ | 0.0 |
| Ng 19-9 3 µM_x000d_ | 99.0 |
| Ng 19-9 10 µM_x000d_ | 6.333333333333343 |
| Ng 20-7 1 µM_x000d_ | 0.0 |
| Ng 20-7 3 µM_x000d_ | 65.66666666666666 |
| Ng 20-7 10 µM_x000d_ | 488.66666666666663 |
| Ng 21-8 1 µM_x000d_ | 75.0 |
| Ng 21-8 3 µM_x000d_ | 213.33333333333331 |
| Ng 21-8 10 µM_x000d_ | 2665.0 |
| Ng 15-13 3µM_x000d_ | 282.3333333333333 |
| Ng 16-12 1 µM_x000d_ | 0.0 |
| Ng 17-9 1 µM_x000d_ | 30.333333333333343 |
| Ng 17-9 3 µM_x000d_ | 356.0 |
| Ng 18-11 1 µM_x000d_ | 1775.0 |
| Ng 18-11 3 µM_x000d_ | 1381.6666666666667 |
| Ng 20-8 1 µM_x000d_ | 312.0 |
| Ng 20-9 1 µM_x000d_ | 1147.0 |
| Ng 20-9 3 µM_x000d_ | 3121.3333333333335 |
| Ng 21-9 1 µM_x000d_ | 2319.6666666666665 |
| Ng 13-13 3 µM_x000d_ | 0.0 |
| Ng 14-8 1 µM_x000d_ | 0.0 |
| Ng 14-9 1 µM_x000d_ | 0.0 |
| Ng 14-9 3 µM_x000d_ | 33.33333333333334 |
| Ng 14-9 10 µM_x000d_ | 94.33333333333334 |
| Ng 14-9 30 µM_x000d_ | 68.66666666666666 |
| Ng 15-10 1µM_x000d_ | 30.0 |
| Ng 16-9 1 µM_x000d_ | 6.0 |
| Ng 17-8 1 µM_x000d_ | 63.0 |
| Ng 17-8 3 µM_x000d_ | 522.6666666666666 |
| Ng 18-9 1µM_x000d_ | 93.0 |
| Ng 19-11 1 µM_x000d_ | 2211.0 |
| Ng 19-11 3 µM_x000d_ | 8558.666666666666 |
| Lg 17-15 HF 10 µM_x000d_ | 2264.6666666666665 |
| Lg 15-17 1 µM_x000d_ | 53.66666666666666 |
| Lg 17-15 1 µM_x000d_ | 906.0 |
| Lg 17-15 3 µM_x000d_ | 3313.6666666666665 |
| Lg 17-15 10 µM_x000d_ | 22075.0 |
| Lg 17-17 1 µM_x000d_ | 405.0 |
| Lg 17-16 1 µM_x000d_ | 1822.0 |
| Lg 16-17 3 µM_x000d_ | 1689.0 |
| Lg 18-8 1 µM_x000d_ | 644.6666666666666 |
| Lg 19-8 1 µM_x000d_ | 2275.6666666666665 |
| Lg 19-8 3 µM_x000d_ | 6926.666666666667 |
| Lg 19-9 1 µM_x000d_ | 2681.6666666666665 |
| Lg 20-10_x000d_ | 1272.6666666666667 |
| Lg 22-12 1 µM_x000d_ | 383.0 |
| Lg 20-9 1 µM_x000d_ | 1818.3333333333333 |
| Lg 23-11 1 µM_x000d_ | 3758.3333333333335 |
| Lg 23-11 3 µM_x000d_ | 15243.333333333334 |
| Lg 23-12_x000d_ | 9896.333333333334 |
| Lg 26-8_x000d_ | 8007.666666666667 |
| Lg 26-9_x000d_ | 1365.0 |
| Lg 22-13_x000d_ | 2747.0 |
| Lg 26-10_x000d_ | 92.0 |
| Lg 18-7-F3 HF 1uM | 59.0 |
| Lg 19-8-F5 HF 3uM | 261.0 |
| Lg 23-11-F2 HF 3uM | 10.333333333333343 |
| Blank 1 | 0.0 |
| Blank 2 | 21.333333333333343 |
| Blank 3 | 0.0 |
| Blank 4 | 0.0 |
| Blank 5 | 2.333333333333343 |MFI
### Chart: Un-G2
| Category | UG 000-215 IgG2 |
|---|---|
| Ng 7-13 1µM_x000d_ | 0.0 |
| Ng 7-13 3µM_x000d_ | 297.0 |
| Ng 9-9 1 µM_x000d_ | 0.0 |
| Ng 9-9 3µM_x000d_ | 164.5 |
| Ng 9-9 10µM_x000d_ | 0.0 |
| Ng 10-13 1 µM_x000d_ | 0.0 |
| Ng 10-13 3 µM_x000d_ | 0.0 |
| Ng 10-13 10 µM_x000d_ | 0.0 |
| Ng 10-13 30 µM_x000d_ | 0.0 |
| Ng 10-10 1 µM_x000d_ | 0.0 |
| Ng 10-10 3 µM_x000d_ | 0.0 |
| Ng 10-11 1 µM_x000d_ | 0.0 |
| Ng 10-11 3 µM_x000d_ | 0.0 |
| Ng 10-12 1 µM_x000d_ | 0.0 |
| Ng 11-10 1 µM_x000d_ | 0.0 |
| Ng 11-10 3 µM_x000d_ | 0.0 |
| Ng 11-11 1 µM_x000d_ | 0.0 |
| Ng 11-11 3µM_x000d_ | 0.0 |
| Ng 11-11 10µM_x000d_ | 0.0 |
| Ng 12-11 1 µM_x000d_ | 0.0 |
| Ng 12-11 3 µM_x000d_ | 0.0 |
| Ng 13-15 1 µM_x000d_ | 0.0 |
| Ng 13-15 3 µM_x000d_ | 0.0 |
| Ng 13-14_x000d_ | 4306.0 |
| Ng 14-10 1 µM_x000d_ | 2812.6666666666665 |
| Ng 14-10 3 µM_x000d_ | 20520.333333333332 |
| Ng 14-11 1 µM_x000d_ | 1079.0 |
| Ng 14-12 1 µM_x000d_ | 54848.333333333336 |
| Ng 14-7 1 µM_x000d_ | 1074.3333333333333 |
| Ng 15-14/15 1 µM_x000d_ | 9556.333333333334 |
| Ng 15-12 1µM_x000d_ | 0.0 |
| Ng 17-7 1 µM_x000d_ | 23.0 |
| Ng 17-7 3 µM_x000d_ | 1140.6666666666667 |
| Ng 17-7 10 µM_x000d_ | 3158.3333333333335 |
| Ng 19-9 1 µM_x000d_ | 100.33333333333334 |
| Ng 19-9 3 µM_x000d_ | 1474.6666666666667 |
| Ng 19-9 10 µM_x000d_ | 2380.6666666666665 |
| Ng 20-7 1 µM_x000d_ | 0.0 |
| Ng 20-7 3 µM_x000d_ | 1642.3333333333333 |
| Ng 20-7 10 µM_x000d_ | 4542.666666666667 |
| Ng 21-8 1 µM_x000d_ | 0.0 |
| Ng 21-8 3 µM_x000d_ | 346.3333333333333 |
| Ng 21-8 10 µM_x000d_ | 4291.333333333333 |
| Ng 15-13 3µM_x000d_ | 1363.3333333333333 |
| Ng 16-12 1 µM_x000d_ | 0.0 |
| Ng 17-9 1 µM_x000d_ | 87.66666666666666 |
| Ng 17-9 3 µM_x000d_ | 1852.0 |
| Ng 18-11 1 µM_x000d_ | 1137.0 |
| Ng 18-11 3 µM_x000d_ | 1449.0 |
| Ng 20-8 1 µM_x000d_ | 296.0 |
| Ng 20-9 1 µM_x000d_ | 1730.0 |
| Ng 20-9 3 µM_x000d_ | 6012.333333333333 |
| Ng 21-9 1 µM_x000d_ | 3870.6666666666665 |
| Ng 13-13 3 µM_x000d_ | 0.0 |
| Ng 14-8 1 µM_x000d_ | 0.0 |
| Ng 14-9 1 µM_x000d_ | 0.0 |
| Ng 14-9 3 µM_x000d_ | 0.0 |
| Ng 14-9 10 µM_x000d_ | 130.66666666666669 |
| Ng 14-9 30 µM_x000d_ | 164.0 |
| Ng 15-10 1µM_x000d_ | 2176.6666666666665 |
| Ng 16-9 1 µM_x000d_ | 1103.0 |
| Ng 17-8 1 µM_x000d_ | 0.0 |
| Ng 17-8 3 µM_x000d_ | 539.6666666666666 |
| Ng 18-9 1µM_x000d_ | 46.66666666666666 |
| Ng 19-11 1 µM_x000d_ | 6826.333333333333 |
| Ng 19-11 3 µM_x000d_ | 27155.333333333332 |
| Lg 17-15 HF 10 µM_x000d_ | 242.66666666666669 |
| Lg 15-17 1 µM_x000d_ | 0.0 |
| Lg 17-15 1 µM_x000d_ | 630.0 |
| Lg 17-15 3 µM_x000d_ | 4117.333333333333 |
| Lg 17-15 10 µM_x000d_ | 14425.666666666666 |
| Lg 17-17 1 µM_x000d_ | 809.6666666666666 |
| Lg 17-16 1 µM_x000d_ | 2052.0 |
| Lg 16-17 3 µM_x000d_ | 2303.0 |
| Lg 18-8 1 µM_x000d_ | 1426.3333333333333 |
| Lg 19-8 1 µM_x000d_ | 2734.3333333333335 |
| Lg 19-8 3 µM_x000d_ | 9306.333333333334 |
| Lg 19-9 1 µM_x000d_ | 1894.6666666666667 |
| Lg 20-10_x000d_ | 393.0 |
| Lg 22-12 1 µM_x000d_ | 259.3333333333333 |
| Lg 20-9 1 µM_x000d_ | 3819.0 |
| Lg 23-11 1 µM_x000d_ | 3311.6666666666665 |
| Lg 23-11 3 µM_x000d_ | 13819.666666666666 |
| Lg 23-12_x000d_ | 2479.6666666666665 |
| Lg 26-8_x000d_ | 3087.0 |
| Lg 26-9_x000d_ | 818.3333333333334 |
| Lg 22-13_x000d_ | 622.3333333333334 |
| Lg 26-10_x000d_ | 0.0 |
| Lg 18-7-F3 HF 1uM | 597.6666666666666 |
| Lg 19-8-F5 HF 3uM | 5512.0 |
| Lg 23-11-F2 HF 3uM | 883.6666666666667 |
| Blank 1 | 0.0 |
| Blank 2 | 0.0 |
| Blank 3 | 0.0 |
| Blank 4 | 0.0 |
| Blank 5 | 0.0 |MFI
### Chart: Un-G3
| Category | UG 000-223 IgG2 |
|---|---|
| Ng 7-13 1µM_x000d_ | 0.0 |
| Ng 7-13 3µM_x000d_ | 46.33333333333334 |
| Ng 9-9 1 µM_x000d_ | 0.0 |
| Ng 9-9 3µM_x000d_ | 563.3333333333334 |
| Ng 9-9 10µM_x000d_ | 3.333333333333343 |
| Ng 10-13 1 µM_x000d_ | 0.0 |
| Ng 10-13 3 µM_x000d_ | 0.0 |
| Ng 10-13 10 µM_x000d_ | 0.0 |
| Ng 10-13 30 µM_x000d_ | 0.0 |
| Ng 10-10 1 µM_x000d_ | 0.0 |
| Ng 10-10 3 µM_x000d_ | 0.0 |
| Ng 10-11 1 µM_x000d_ | 0.0 |
| Ng 10-11 3 µM_x000d_ | 0.0 |
| Ng 10-12 1 µM_x000d_ | 0.0 |
| Ng 11-10 1 µM_x000d_ | 0.0 |
| Ng 11-10 3 µM_x000d_ | 0.0 |
| Ng 11-11 1 µM_x000d_ | 0.0 |
| Ng 11-11 3µM_x000d_ | 0.0 |
| Ng 11-11 10µM_x000d_ | 0.0 |
| Ng 12-11 1 µM_x000d_ | 0.0 |
| Ng 12-11 3 µM_x000d_ | 0.0 |
| Ng 13-15 1 µM_x000d_ | 0.0 |
| Ng 13-15 3 µM_x000d_ | 0.0 |
| Ng 13-14_x000d_ | 162.66666666666669 |
| Ng 14-10 1 µM_x000d_ | 230.66666666666669 |
| Ng 14-10 3 µM_x000d_ | 383.33333333333337 |
| Ng 14-11 1 µM_x000d_ | 0.0 |
| Ng 14-12 1 µM_x000d_ | 749.3333333333334 |
| Ng 14-7 1 µM_x000d_ | 4.0 |
| Ng 15-14/15 1 µM_x000d_ | 33.0 |
| Ng 15-12 1µM_x000d_ | 0.0 |
| Ng 17-7 1 µM_x000d_ | 0.0 |
| Ng 17-7 3 µM_x000d_ | 18.666666666666657 |
| Ng 17-7 10 µM_x000d_ | 90.0 |
| Ng 19-9 1 µM_x000d_ | 0.0 |
| Ng 19-9 3 µM_x000d_ | 93.66666666666666 |
| Ng 19-9 10 µM_x000d_ | 166.33333333333331 |
| Ng 20-7 1 µM_x000d_ | 0.0 |
| Ng 20-7 3 µM_x000d_ | 7.0 |
| Ng 20-7 10 µM_x000d_ | 46.66666666666666 |
| Ng 21-8 1 µM_x000d_ | 0.0 |
| Ng 21-8 3 µM_x000d_ | 0.0 |
| Ng 21-8 10 µM_x000d_ | 0.0 |
| Ng 15-13 3µM_x000d_ | 65.0 |
| Ng 16-12 1 µM_x000d_ | 0.0 |
| Ng 17-9 1 µM_x000d_ | 0.0 |
| Ng 17-9 3 µM_x000d_ | 40.66666666666666 |
| Ng 18-11 1 µM_x000d_ | 95.0 |
| Ng 18-11 3 µM_x000d_ | 549.0 |
| Ng 20-8 1 µM_x000d_ | 4.333333333333343 |
| Ng 20-9 1 µM_x000d_ | 42.66666666666666 |
| Ng 20-9 3 µM_x000d_ | 766.0 |
| Ng 21-9 1 µM_x000d_ | 18.666666666666657 |
| Ng 13-13 3 µM_x000d_ | 0.0 |
| Ng 14-8 1 µM_x000d_ | 0.0 |
| Ng 14-9 1 µM_x000d_ | 0.3333333333333428 |
| Ng 14-9 3 µM_x000d_ | 26.333333333333343 |
| Ng 14-9 10 µM_x000d_ | 43.33333333333334 |
| Ng 14-9 30 µM_x000d_ | 10.333333333333343 |
| Ng 15-10 1µM_x000d_ | 29.666666666666657 |
| Ng 16-9 1 µM_x000d_ | 8.666666666666657 |
| Ng 17-8 1 µM_x000d_ | 0.0 |
| Ng 17-8 3 µM_x000d_ | 16.333333333333343 |
| Ng 18-9 1µM_x000d_ | 0.0 |
| Ng 19-11 1 µM_x000d_ | 1046.3333333333333 |
| Ng 19-11 3 µM_x000d_ | 3531.6666666666665 |
| Lg 17-15 HF 10 µM_x000d_ | 566.3333333333334 |
| Lg 15-17 1 µM_x000d_ | 6.0 |
| Lg 17-15 1 µM_x000d_ | 0.0 |
| Lg 17-15 3 µM_x000d_ | 9.666666666666657 |
| Lg 17-15 10 µM_x000d_ | 403.33333333333337 |
| Lg 17-17 1 µM_x000d_ | 0.0 |
| Lg 17-16 1 µM_x000d_ | 0.0 |
| Lg 16-17 3 µM_x000d_ | 5.666666666666657 |
| Lg 18-8 1 µM_x000d_ | 0.0 |
| Lg 19-8 1 µM_x000d_ | 28.666666666666657 |
| Lg 19-8 3 µM_x000d_ | 45.33333333333334 |
| Lg 19-9 1 µM_x000d_ | 5.666666666666657 |
| Lg 20-10_x000d_ | 21.666666666666657 |
| Lg 22-12 1 µM_x000d_ | 3.333333333333343 |
| Lg 20-9 1 µM_x000d_ | 104.66666666666666 |
| Lg 23-11 1 µM_x000d_ | 35.0 |
| Lg 23-11 3 µM_x000d_ | 263.3333333333333 |
| Lg 23-12_x000d_ | 96.33333333333334 |
| Lg 26-8_x000d_ | 11.0 |
| Lg 26-9_x000d_ | 0.0 |
| Lg 22-13_x000d_ | 20.333333333333343 |
| Lg 26-10_x000d_ | 16.0 |
| Lg 18-7-F3 HF 1uM | 5.0 |
| Lg 19-8-F5 HF 3uM | 13.666666666666657 |
| Lg 23-11-F2 HF 3uM | 10.0 |
| Blank 1 | 0.0 |
| Blank 2 | 0.0 |
| Blank 3 | 0.0 |
| Blank 4 | 0.0 |
| Blank 5 | 0.0 |MFI
### Chart: Un-G4
| Category | UG 000-224 IgG2 |
|---|---|
| Ng 7-13 1µM_x000d_ | 123.66666666666669 |
| Ng 7-13 3µM_x000d_ | 240.33333333333331 |
| Ng 9-9 1 µM_x000d_ | 0.0 |
| Ng 9-9 3µM_x000d_ | 336.6666666666667 |
| Ng 9-9 10µM_x000d_ | 74.0 |
| Ng 10-13 1 µM_x000d_ | 0.0 |
| Ng 10-13 3 µM_x000d_ | 0.0 |
| Ng 10-13 10 µM_x000d_ | 22.666666666666657 |
| Ng 10-13 30 µM_x000d_ | 52.0 |
| Ng 10-10 1 µM_x000d_ | 33.66666666666666 |
| Ng 10-10 3 µM_x000d_ | 30.666666666666657 |
| Ng 10-11 1 µM_x000d_ | 0.0 |
| Ng 10-11 3 µM_x000d_ | 31.0 |
| Ng 10-12 1 µM_x000d_ | 29.666666666666657 |
| Ng 11-10 1 µM_x000d_ | 0.0 |
| Ng 11-10 3 µM_x000d_ | 14.333333333333343 |
| Ng 11-11 1 µM_x000d_ | 14.0 |
| Ng 11-11 3µM_x000d_ | 62.66666666666666 |
| Ng 11-11 10µM_x000d_ | 0.0 |
| Ng 12-11 1 µM_x000d_ | 0.0 |
| Ng 12-11 3 µM_x000d_ | 17.333333333333343 |
| Ng 13-15 1 µM_x000d_ | 0.0 |
| Ng 13-15 3 µM_x000d_ | 41.33333333333334 |
| Ng 13-14_x000d_ | 2602.6666666666665 |
| Ng 14-10 1 µM_x000d_ | 658.3333333333334 |
| Ng 14-10 3 µM_x000d_ | 3382.3333333333335 |
| Ng 14-11 1 µM_x000d_ | 183.33333333333331 |
| Ng 14-12 1 µM_x000d_ | 9226.666666666666 |
| Ng 14-7 1 µM_x000d_ | 151.0 |
| Ng 15-14/15 1 µM_x000d_ | 2293.3333333333335 |
| Ng 15-12 1µM_x000d_ | 114.33333333333331 |
| Ng 17-7 1 µM_x000d_ | 0.0 |
| Ng 17-7 3 µM_x000d_ | 230.33333333333331 |
| Ng 17-7 10 µM_x000d_ | 633.6666666666666 |
| Ng 19-9 1 µM_x000d_ | 23.333333333333343 |
| Ng 19-9 3 µM_x000d_ | 90.66666666666666 |
| Ng 19-9 10 µM_x000d_ | 153.66666666666669 |
| Ng 20-7 1 µM_x000d_ | 54.66666666666666 |
| Ng 20-7 3 µM_x000d_ | 650.0 |
| Ng 20-7 10 µM_x000d_ | 671.0 |
| Ng 21-8 1 µM_x000d_ | 0.0 |
| Ng 21-8 3 µM_x000d_ | 0.0 |
| Ng 21-8 10 µM_x000d_ | 701.6666666666666 |
| Ng 15-13 3µM_x000d_ | 319.0 |
| Ng 16-12 1 µM_x000d_ | 0.0 |
| Ng 17-9 1 µM_x000d_ | 9.333333333333343 |
| Ng 17-9 3 µM_x000d_ | 218.33333333333331 |
| Ng 18-11 1 µM_x000d_ | 118.33333333333331 |
| Ng 18-11 3 µM_x000d_ | 115.0 |
| Ng 20-8 1 µM_x000d_ | 16.666666666666657 |
| Ng 20-9 1 µM_x000d_ | 69.0 |
| Ng 20-9 3 µM_x000d_ | 113.0 |
| Ng 21-9 1 µM_x000d_ | 100.66666666666666 |
| Ng 13-13 3 µM_x000d_ | 20.333333333333343 |
| Ng 14-8 1 µM_x000d_ | 13.0 |
| Ng 14-9 1 µM_x000d_ | 0.0 |
| Ng 14-9 3 µM_x000d_ | 77.0 |
| Ng 14-9 10 µM_x000d_ | 51.66666666666666 |
| Ng 14-9 30 µM_x000d_ | 49.66666666666666 |
| Ng 15-10 1µM_x000d_ | 1295.3333333333333 |
| Ng 16-9 1 µM_x000d_ | 48.33333333333334 |
| Ng 17-8 1 µM_x000d_ | 0.0 |
| Ng 17-8 3 µM_x000d_ | 10.666666666666657 |
| Ng 18-9 1µM_x000d_ | 24.0 |
| Ng 19-11 1 µM_x000d_ | 807.0 |
| Ng 19-11 3 µM_x000d_ | 3190.3333333333335 |
| Lg 17-15 HF 10 µM_x000d_ | 257.0 |
| Lg 15-17 1 µM_x000d_ | 0.0 |
| Lg 17-15 1 µM_x000d_ | 0.0 |
| Lg 17-15 3 µM_x000d_ | 15.666666666666657 |
| Lg 17-15 10 µM_x000d_ | 97.66666666666666 |
| Lg 17-17 1 µM_x000d_ | 0.0 |
| Lg 17-16 1 µM_x000d_ | 0.0 |
| Lg 16-17 3 µM_x000d_ | 0.0 |
| Lg 18-8 1 µM_x000d_ | 46.0 |
| Lg 19-8 1 µM_x000d_ | 96.0 |
| Lg 19-8 3 µM_x000d_ | 661.0 |
| Lg 19-9 1 µM_x000d_ | 98.0 |
| Lg 20-10_x000d_ | 36.66666666666666 |
| Lg 22-12 1 µM_x000d_ | 0.0 |
| Lg 20-9 1 µM_x000d_ | 1314.3333333333333 |
| Lg 23-11 1 µM_x000d_ | 613.6666666666666 |
| Lg 23-11 3 µM_x000d_ | 3748.0 |
| Lg 23-12_x000d_ | 147.33333333333331 |
| Lg 26-8_x000d_ | 801.6666666666666 |
| Lg 26-9_x000d_ | 125.0 |
| Lg 22-13_x000d_ | 12.333333333333343 |
| Lg 26-10_x000d_ | 0.0 |
| Lg 18-7-F3 HF 1uM | 15.333333333333343 |
| Lg 19-8-F5 HF 3uM | 1275.0 |
| Lg 23-11-F2 HF 3uM | 256.3333333333333 |
| Blank 1 | 0.0 |
| Blank 2 | 0.0 |
| Blank 3 | 0.0 |
| Blank 4 | 0.0 |
| Blank 5 | 0.0 |MFI
### Chart: Un-G5
| Category | UG 000-225 IgG2 |
|---|---|
| Ng 7-13 1µM_x000d_ | 107.66666666666669 |
| Ng 7-13 3µM_x000d_ | 626.3333333333334 |
| Ng 9-9 1 µM_x000d_ | 0.0 |
| Ng 9-9 3µM_x000d_ | 835.3333333333334 |
| Ng 9-9 10µM_x000d_ | 90.0 |
| Ng 10-13 1 µM_x000d_ | 10.0 |
| Ng 10-13 3 µM_x000d_ | 0.0 |
| Ng 10-13 10 µM_x000d_ | 50.66666666666666 |
| Ng 10-13 30 µM_x000d_ | 0.0 |
| Ng 10-10 1 µM_x000d_ | 43.33333333333334 |
| Ng 10-10 3 µM_x000d_ | 130.33333333333331 |
| Ng 10-11 1 µM_x000d_ | 13.0 |
| Ng 10-11 3 µM_x000d_ | 60.66666666666666 |
| Ng 10-12 1 µM_x000d_ | 139.66666666666669 |
| Ng 11-10 1 µM_x000d_ | 84.0 |
| Ng 11-10 3 µM_x000d_ | 120.33333333333331 |
| Ng 11-11 1 µM_x000d_ | 178.66666666666669 |
| Ng 11-11 3µM_x000d_ | 45.66666666666666 |
| Ng 11-11 10µM_x000d_ | 0.0 |
| Ng 12-11 1 µM_x000d_ | 17.666666666666657 |
| Ng 12-11 3 µM_x000d_ | 78.66666666666666 |
| Ng 13-15 1 µM_x000d_ | 0.0 |
| Ng 13-15 3 µM_x000d_ | 72.33333333333334 |
| Ng 13-14_x000d_ | 3906.3333333333335 |
| Ng 14-10 1 µM_x000d_ | 5774.666666666667 |
| Ng 14-10 3 µM_x000d_ | 10964.666666666666 |
| Ng 14-11 1 µM_x000d_ | 457.0 |
| Ng 14-12 1 µM_x000d_ | 29323.666666666668 |
| Ng 14-7 1 µM_x000d_ | 199.66666666666669 |
| Ng 15-14/15 1 µM_x000d_ | 4400.333333333333 |
| Ng 15-12 1µM_x000d_ | 0.0 |
| Ng 17-7 1 µM_x000d_ | 156.33333333333331 |
| Ng 17-7 3 µM_x000d_ | 818.3333333333334 |
| Ng 17-7 10 µM_x000d_ | 1946.3333333333335 |
| Ng 19-9 1 µM_x000d_ | 166.33333333333331 |
| Ng 19-9 3 µM_x000d_ | 1147.3333333333333 |
| Ng 19-9 10 µM_x000d_ | 1675.0 |
| Ng 20-7 1 µM_x000d_ | 123.33333333333331 |
| Ng 20-7 3 µM_x000d_ | 189.0 |
| Ng 20-7 10 µM_x000d_ | 4322.0 |
| Ng 21-8 1 µM_x000d_ | 39.66666666666666 |
| Ng 21-8 3 µM_x000d_ | 256.6666666666667 |
| Ng 21-8 10 µM_x000d_ | 1224.6666666666667 |
| Ng 15-13 3µM_x000d_ | 661.0 |
| Ng 16-12 1 µM_x000d_ | 51.0 |
| Ng 17-9 1 µM_x000d_ | 130.66666666666669 |
| Ng 17-9 3 µM_x000d_ | 553.3333333333334 |
| Ng 18-11 1 µM_x000d_ | 189.0 |
| Ng 18-11 3 µM_x000d_ | 2383.0 |
| Ng 20-8 1 µM_x000d_ | 470.33333333333337 |
| Ng 20-9 1 µM_x000d_ | 1312.6666666666667 |
| Ng 20-9 3 µM_x000d_ | 4097.333333333333 |
| Ng 21-9 1 µM_x000d_ | 1383.3333333333333 |
| Ng 13-13 3 µM_x000d_ | 162.33333333333331 |
| Ng 14-8 1 µM_x000d_ | 109.33333333333331 |
| Ng 14-9 1 µM_x000d_ | 32.33333333333334 |
| Ng 14-9 3 µM_x000d_ | 138.33333333333331 |
| Ng 14-9 10 µM_x000d_ | 206.66666666666669 |
| Ng 14-9 30 µM_x000d_ | 186.0 |
| Ng 15-10 1µM_x000d_ | 2621.3333333333335 |
| Ng 16-9 1 µM_x000d_ | 1730.0 |
| Ng 17-8 1 µM_x000d_ | 131.0 |
| Ng 17-8 3 µM_x000d_ | 478.33333333333337 |
| Ng 18-9 1µM_x000d_ | 74.66666666666666 |
| Ng 19-11 1 µM_x000d_ | 8689.333333333334 |
| Ng 19-11 3 µM_x000d_ | 9658.333333333334 |
| Lg 17-15 HF 10 µM_x000d_ | 2400.0 |
| Lg 15-17 1 µM_x000d_ | 75.0 |
| Lg 17-15 1 µM_x000d_ | 251.0 |
| Lg 17-15 3 µM_x000d_ | 1109.3333333333333 |
| Lg 17-15 10 µM_x000d_ | 3311.3333333333335 |
| Lg 17-17 1 µM_x000d_ | 279.6666666666667 |
| Lg 17-16 1 µM_x000d_ | 107.33333333333331 |
| Lg 16-17 3 µM_x000d_ | 874.0 |
| Lg 18-8 1 µM_x000d_ | 307.3333333333333 |
| Lg 19-8 1 µM_x000d_ | 628.6666666666666 |
| Lg 19-8 3 µM_x000d_ | 2013.3333333333335 |
| Lg 19-9 1 µM_x000d_ | 225.66666666666669 |
| Lg 20-10_x000d_ | 1673.6666666666667 |
| Lg 22-12 1 µM_x000d_ | 143.0 |
| Lg 20-9 1 µM_x000d_ | 335.6666666666667 |
| Lg 23-11 1 µM_x000d_ | 2432.3333333333335 |
| Lg 23-11 3 µM_x000d_ | 8336.666666666666 |
| Lg 23-12_x000d_ | 2602.3333333333335 |
| Lg 26-8_x000d_ | 901.0 |
| Lg 26-9_x000d_ | 419.33333333333337 |
| Lg 22-13_x000d_ | 962.0 |
| Lg 26-10_x000d_ | 110.0 |
| Lg 18-7-F3 HF 1uM | 891.3333333333333 |
| Lg 19-8-F5 HF 3uM | 5630.666666666667 |
| Lg 23-11-F2 HF 3uM | 2029.3333333333335 |
| Blank 1 | 18.0 |
| Blank 2 | 0.0 |
| Blank 3 | 0.0 |
| Blank 4 | 0.0 |
| Blank 5 | 0.0 |MFI
### Chart: Un-G6
| Category | UG 000-226 IgG2 |
|---|---|
| Ng 7-13 1µM_x000d_ | 366.33333333333337 |
| Ng 7-13 3µM_x000d_ | 149.0 |
| Ng 9-9 1 µM_x000d_ | 11.666666666666657 |
| Ng 9-9 3µM_x000d_ | 871.3333333333334 |
| Ng 9-9 10µM_x000d_ | 164.0 |
| Ng 10-13 1 µM_x000d_ | 0.0 |
| Ng 10-13 3 µM_x000d_ | 0.0 |
| Ng 10-13 10 µM_x000d_ | 37.33333333333334 |
| Ng 10-13 30 µM_x000d_ | 0.0 |
| Ng 10-10 1 µM_x000d_ | 65.0 |
| Ng 10-10 3 µM_x000d_ | 36.0 |
| Ng 10-11 1 µM_x000d_ | 50.66666666666666 |
| Ng 10-11 3 µM_x000d_ | 97.33333333333334 |
| Ng 10-12 1 µM_x000d_ | 0.6666666666666572 |
| Ng 11-10 1 µM_x000d_ | 8.0 |
| Ng 11-10 3 µM_x000d_ | 39.66666666666666 |
| Ng 11-11 1 µM_x000d_ | 32.33333333333334 |
| Ng 11-11 3µM_x000d_ | 0.0 |
| Ng 11-11 10µM_x000d_ | 0.0 |
| Ng 12-11 1 µM_x000d_ | 0.0 |
| Ng 12-11 3 µM_x000d_ | 35.66666666666666 |
| Ng 13-15 1 µM_x000d_ | 0.0 |
| Ng 13-15 3 µM_x000d_ | 55.33333333333334 |
| Ng 13-14_x000d_ | 2015.0 |
| Ng 14-10 1 µM_x000d_ | 348.3333333333333 |
| Ng 14-10 3 µM_x000d_ | 2133.6666666666665 |
| Ng 14-11 1 µM_x000d_ | 36.0 |
| Ng 14-12 1 µM_x000d_ | 5149.0 |
| Ng 14-7 1 µM_x000d_ | 27.333333333333343 |
| Ng 15-14/15 1 µM_x000d_ | 923.3333333333333 |
| Ng 15-12 1µM_x000d_ | 0.0 |
| Ng 17-7 1 µM_x000d_ | 59.33333333333334 |
| Ng 17-7 3 µM_x000d_ | 56.33333333333334 |
| Ng 17-7 10 µM_x000d_ | 163.66666666666669 |
| Ng 19-9 1 µM_x000d_ | 0.0 |
| Ng 19-9 3 µM_x000d_ | 52.33333333333334 |
| Ng 19-9 10 µM_x000d_ | 82.33333333333334 |
| Ng 20-7 1 µM_x000d_ | 0.0 |
| Ng 20-7 3 µM_x000d_ | 127.66666666666669 |
| Ng 20-7 10 µM_x000d_ | 327.3333333333333 |
| Ng 21-8 1 µM_x000d_ | 0.0 |
| Ng 21-8 3 µM_x000d_ | 48.66666666666666 |
| Ng 21-8 10 µM_x000d_ | 221.0 |
| Ng 15-13 3µM_x000d_ | 146.0 |
| Ng 16-12 1 µM_x000d_ | 0.0 |
| Ng 17-9 1 µM_x000d_ | 46.33333333333334 |
| Ng 17-9 3 µM_x000d_ | 36.0 |
| Ng 18-11 1 µM_x000d_ | 23.0 |
| Ng 18-11 3 µM_x000d_ | 0.0 |
| Ng 20-8 1 µM_x000d_ | 122.0 |
| Ng 20-9 1 µM_x000d_ | 50.33333333333334 |
| Ng 20-9 3 µM_x000d_ | 42.0 |
| Ng 21-9 1 µM_x000d_ | 112.33333333333331 |
| Ng 13-13 3 µM_x000d_ | 5.666666666666657 |
| Ng 14-8 1 µM_x000d_ | 31.666666666666657 |
| Ng 14-9 1 µM_x000d_ | 0.0 |
| Ng 14-9 3 µM_x000d_ | 21.0 |
| Ng 14-9 10 µM_x000d_ | 13.0 |
| Ng 14-9 30 µM_x000d_ | 23.333333333333343 |
| Ng 15-10 1µM_x000d_ | 407.66666666666663 |
| Ng 16-9 1 µM_x000d_ | 123.66666666666669 |
| Ng 17-8 1 µM_x000d_ | 0.0 |
| Ng 17-8 3 µM_x000d_ | 0.0 |
| Ng 18-9 1µM_x000d_ | 3.333333333333343 |
| Ng 19-11 1 µM_x000d_ | 458.0 |
| Ng 19-11 3 µM_x000d_ | 1197.3333333333333 |
| Lg 17-15 HF 10 µM_x000d_ | 2439.6666666666665 |
| Lg 15-17 1 µM_x000d_ | 2.333333333333343 |
| Lg 17-15 1 µM_x000d_ | 35.0 |
| Lg 17-15 3 µM_x000d_ | 221.33333333333331 |
| Lg 17-15 10 µM_x000d_ | 873.6666666666666 |
| Lg 17-17 1 µM_x000d_ | 24.666666666666657 |
| Lg 17-16 1 µM_x000d_ | 20.666666666666657 |
| Lg 16-17 3 µM_x000d_ | 225.66666666666669 |
| Lg 18-8 1 µM_x000d_ | 63.0 |
| Lg 19-8 1 µM_x000d_ | 0.0 |
| Lg 19-8 3 µM_x000d_ | 461.33333333333337 |
| Lg 19-9 1 µM_x000d_ | 36.66666666666666 |
| Lg 20-10_x000d_ | 182.33333333333331 |
| Lg 22-12 1 µM_x000d_ | 0.0 |
| Lg 20-9 1 µM_x000d_ | 803.6666666666666 |
| Lg 23-11 1 µM_x000d_ | 354.6666666666667 |
| Lg 23-11 3 µM_x000d_ | 969.0 |
| Lg 23-12_x000d_ | 114.66666666666669 |
| Lg 26-8_x000d_ | 206.66666666666669 |
| Lg 26-9_x000d_ | 51.66666666666666 |
| Lg 22-13_x000d_ | 21.0 |
| Lg 26-10_x000d_ | 106.66666666666669 |
| Lg 18-7-F3 HF 1uM | 12.666666666666657 |
| Lg 19-8-F5 HF 3uM | 729.0 |
| Lg 23-11-F2 HF 3uM | 80.33333333333334 |
| Blank 1 | 103.33333333333334 |
| Blank 2 | 0.0 |
| Blank 3 | 0.0 |
| Blank 4 | 0.0 |
| Blank 5 | 124.0 |MFI
### Chart: Un-NL1
| Category | NLD-57 IgG2 |
|---|---|
| Ng 7-13 1µM_x000d_ | 92.0 |
| Ng 7-13 3µM_x000d_ | 327.0 |
| Ng 9-9 1 µM_x000d_ | 51.333333333333314 |
| Ng 9-9 3µM_x000d_ | 683.0 |
| Ng 9-9 10µM_x000d_ | 96.33333333333331 |
| Ng 10-13 1 µM_x000d_ | 15.666666666666657 |
| Ng 10-13 3 µM_x000d_ | 0.0 |
| Ng 10-13 10 µM_x000d_ | 0.0 |
| Ng 10-13 30 µM_x000d_ | 0.0 |
| Ng 10-10 1 µM_x000d_ | 8.0 |
| Ng 10-10 3 µM_x000d_ | 23.333333333333314 |
| Ng 10-11 1 µM_x000d_ | 6.666666666666657 |
| Ng 10-11 3 µM_x000d_ | 78.0 |
| Ng 10-12 1 µM_x000d_ | 0.0 |
| Ng 11-10 1 µM_x000d_ | 0.0 |
| Ng 11-10 3 µM_x000d_ | 18.666666666666657 |
| Ng 11-11 1 µM_x000d_ | 0.0 |
| Ng 11-11 3µM_x000d_ | 0.0 |
| Ng 11-11 10µM_x000d_ | 0.0 |
| Ng 12-11 1 µM_x000d_ | 0.0 |
| Ng 12-11 3 µM_x000d_ | 0.0 |
| Ng 13-15 1 µM_x000d_ | 0.0 |
| Ng 13-15 3 µM_x000d_ | 10.333333333333314 |
| Ng 13-14_x000d_ | 3223.0 |
| Ng 14-10 1 µM_x000d_ | 3668.6666666666665 |
| Ng 14-10 3 µM_x000d_ | 6945.333333333334 |
| Ng 14-11 1 µM_x000d_ | 1127.3333333333335 |
| Ng 14-12 1 µM_x000d_ | 8607.333333333332 |
| Ng 14-7 1 µM_x000d_ | 2325.0 |
| Ng 15-14/15 1 µM_x000d_ | 3187.333333333333 |
| Ng 15-12 1µM_x000d_ | 33.333333333333314 |
| Ng 17-7 1 µM_x000d_ | 731.0 |
| Ng 17-7 3 µM_x000d_ | 2456.6666666666665 |
| Ng 17-7 10 µM_x000d_ | 4307.333333333334 |
| Ng 19-9 1 µM_x000d_ | 901.9999999999999 |
| Ng 19-9 3 µM_x000d_ | 5695.333333333334 |
| Ng 19-9 10 µM_x000d_ | 7061.333333333334 |
| Ng 20-7 1 µM_x000d_ | 480.66666666666663 |
| Ng 20-7 3 µM_x000d_ | 3524.333333333333 |
| Ng 20-7 10 µM_x000d_ | 5117.0 |
| Ng 21-8 1 µM_x000d_ | 159.99999999999997 |
| Ng 21-8 3 µM_x000d_ | 1936.3333333333333 |
| Ng 21-8 10 µM_x000d_ | 3014.6666666666665 |
| Ng 15-13 3µM_x000d_ | 1167.3333333333335 |
| Ng 16-12 1 µM_x000d_ | 85.66666666666666 |
| Ng 17-9 1 µM_x000d_ | 804.6666666666666 |
| Ng 17-9 3 µM_x000d_ | 3252.333333333333 |
| Ng 18-11 1 µM_x000d_ | 824.3333333333333 |
| Ng 18-11 3 µM_x000d_ | 1349.3333333333335 |
| Ng 20-8 1 µM_x000d_ | 731.6666666666666 |
| Ng 20-9 1 µM_x000d_ | 1718.3333333333335 |
| Ng 20-9 3 µM_x000d_ | 5493.0 |
| Ng 21-9 1 µM_x000d_ | 1696.6666666666667 |
| Ng 13-13 3 µM_x000d_ | 0.0 |
| Ng 14-8 1 µM_x000d_ | 123.33333333333334 |
| Ng 14-9 1 µM_x000d_ | 25.0 |
| Ng 14-9 3 µM_x000d_ | 102.0 |
| Ng 14-9 10 µM_x000d_ | 331.0 |
| Ng 14-9 30 µM_x000d_ | 285.33333333333337 |
| Ng 15-10 1µM_x000d_ | 9683.333333333332 |
| Ng 16-9 1 µM_x000d_ | 3018.6666666666665 |
| Ng 17-8 1 µM_x000d_ | 523.3333333333333 |
| Ng 17-8 3 µM_x000d_ | 2196.333333333333 |
| Ng 18-9 1µM_x000d_ | 423.66666666666663 |
| Ng 19-11 1 µM_x000d_ | 5136.333333333334 |
| Ng 19-11 3 µM_x000d_ | 8677.666666666666 |
| Lg 17-15 HF 10 µM_x000d_ | 2476.0 |
| Lg 15-17 1 µM_x000d_ | 115.66666666666666 |
| Lg 17-15 1 µM_x000d_ | 514.0 |
| Lg 17-15 3 µM_x000d_ | 1398.0 |
| Lg 17-15 10 µM_x000d_ | 6092.333333333334 |
| Lg 17-17 1 µM_x000d_ | 337.66666666666663 |
| Lg 17-16 1 µM_x000d_ | 601.3333333333333 |
| Lg 16-17 3 µM_x000d_ | 90.33333333333331 |
| Lg 18-8 1 µM_x000d_ | 653.0 |
| Lg 19-8 1 µM_x000d_ | 3992.6666666666665 |
| Lg 19-8 3 µM_x000d_ | 11063.666666666666 |
| Lg 19-9 1 µM_x000d_ | 3942.333333333333 |
| Lg 20-10_x000d_ | 2967.333333333333 |
| Lg 22-12 1 µM_x000d_ | 418.66666666666663 |
| Lg 20-9 1 µM_x000d_ | 12560.666666666666 |
| Lg 23-11 1 µM_x000d_ | 2679.0 |
| Lg 23-11 3 µM_x000d_ | 18636.666666666668 |
| Lg 23-12_x000d_ | 5894.666666666667 |
| Lg 26-8_x000d_ | 5039.333333333334 |
| Lg 26-9_x000d_ | 369.33333333333326 |
| Lg 22-13_x000d_ | 1245.0 |
| Lg 26-10_x000d_ | 85.33333333333331 |
| Lg 18-7-F3 HF 1uM | 2764.0 |
| Lg 19-8-F5 HF 3uM | 9829.666666666666 |
| Lg 23-11-F2 HF 3uM | 2199.6666666666665 |
| Blank 1 | 2.666666666666657 |
| Blank 2 | 0.0 |
| Blank 3 | 0.0 |
| Blank 4 | 30.666666666666657 |
| Blank 5 | 0.0 |MFI
### Chart: Un-NL2
| Category | NLD-59 IgG2 |
|---|---|
| Ng 7-13 1µM_x000d_ | 19.666666666666657 |
| Ng 7-13 3µM_x000d_ | 18.333333333333343 |
| Ng 9-9 1 µM_x000d_ | 0.0 |
| Ng 9-9 3µM_x000d_ | 51.66666666666666 |
| Ng 9-9 10µM_x000d_ | 41.33333333333334 |
| Ng 10-13 1 µM_x000d_ | 0.0 |
| Ng 10-13 3 µM_x000d_ | 0.0 |
| Ng 10-13 10 µM_x000d_ | 12.333333333333343 |
| Ng 10-13 30 µM_x000d_ | 7.333333333333343 |
| Ng 10-10 1 µM_x000d_ | 0.0 |
| Ng 10-10 3 µM_x000d_ | 36.66666666666666 |
| Ng 10-11 1 µM_x000d_ | 0.0 |
| Ng 10-11 3 µM_x000d_ | 0.0 |
| Ng 10-12 1 µM_x000d_ | 0.0 |
| Ng 11-10 1 µM_x000d_ | 0.0 |
| Ng 11-10 3 µM_x000d_ | 0.0 |
| Ng 11-11 1 µM_x000d_ | 0.0 |
| Ng 11-11 3µM_x000d_ | 3.333333333333343 |
| Ng 11-11 10µM_x000d_ | 0.0 |
| Ng 12-11 1 µM_x000d_ | 0.0 |
| Ng 12-11 3 µM_x000d_ | 0.0 |
| Ng 13-15 1 µM_x000d_ | 0.0 |
| Ng 13-15 3 µM_x000d_ | 0.0 |
| Ng 13-14_x000d_ | 3.0 |
| Ng 14-10 1 µM_x000d_ | 0.0 |
| Ng 14-10 3 µM_x000d_ | 56.0 |
| Ng 14-11 1 µM_x000d_ | 0.0 |
| Ng 14-12 1 µM_x000d_ | 73.66666666666666 |
| Ng 14-7 1 µM_x000d_ | 0.0 |
| Ng 15-14/15 1 µM_x000d_ | 0.3333333333333428 |
| Ng 15-12 1µM_x000d_ | 0.0 |
| Ng 17-7 1 µM_x000d_ | 0.0 |
| Ng 17-7 3 µM_x000d_ | 0.0 |
| Ng 17-7 10 µM_x000d_ | 31.0 |
| Ng 19-9 1 µM_x000d_ | 0.0 |
| Ng 19-9 3 µM_x000d_ | 0.0 |
| Ng 19-9 10 µM_x000d_ | 0.0 |
| Ng 20-7 1 µM_x000d_ | 0.0 |
| Ng 20-7 3 µM_x000d_ | 0.0 |
| Ng 20-7 10 µM_x000d_ | 14.0 |
| Ng 21-8 1 µM_x000d_ | 0.0 |
| Ng 21-8 3 µM_x000d_ | 0.0 |
| Ng 21-8 10 µM_x000d_ | 9.0 |
| Ng 15-13 3µM_x000d_ | 97.0 |
| Ng 16-12 1 µM_x000d_ | 0.0 |
| Ng 17-9 1 µM_x000d_ | 0.0 |
| Ng 17-9 3 µM_x000d_ | 1.6666666666666572 |
| Ng 18-11 1 µM_x000d_ | 0.0 |
| Ng 18-11 3 µM_x000d_ | 7.333333333333343 |
| Ng 20-8 1 µM_x000d_ | 0.0 |
| Ng 20-9 1 µM_x000d_ | 0.0 |
| Ng 20-9 3 µM_x000d_ | 20.666666666666657 |
| Ng 21-9 1 µM_x000d_ | 0.0 |
| Ng 13-13 3 µM_x000d_ | 0.0 |
| Ng 14-8 1 µM_x000d_ | 0.0 |
| Ng 14-9 1 µM_x000d_ | 0.0 |
| Ng 14-9 3 µM_x000d_ | 1.3333333333333428 |
| Ng 14-9 10 µM_x000d_ | 58.33333333333334 |
| Ng 14-9 30 µM_x000d_ | 1.6666666666666572 |
| Ng 15-10 1µM_x000d_ | 0.0 |
| Ng 16-9 1 µM_x000d_ | 0.0 |
| Ng 17-8 1 µM_x000d_ | 0.0 |
| Ng 17-8 3 µM_x000d_ | 0.0 |
| Ng 18-9 1µM_x000d_ | 0.0 |
| Ng 19-11 1 µM_x000d_ | 0.0 |
| Ng 19-11 3 µM_x000d_ | 0.0 |
| Lg 17-15 HF 10 µM_x000d_ | 4.666666666666657 |
| Lg 15-17 1 µM_x000d_ | 0.0 |
| Lg 17-15 1 µM_x000d_ | 0.0 |
| Lg 17-15 3 µM_x000d_ | 0.0 |
| Lg 17-15 10 µM_x000d_ | 11.666666666666657 |
| Lg 17-17 1 µM_x000d_ | 0.0 |
| Lg 17-16 1 µM_x000d_ | 0.0 |
| Lg 16-17 3 µM_x000d_ | 0.0 |
| Lg 18-8 1 µM_x000d_ | 0.0 |
| Lg 19-8 1 µM_x000d_ | 0.0 |
| Lg 19-8 3 µM_x000d_ | 0.0 |
| Lg 19-9 1 µM_x000d_ | 0.0 |
| Lg 20-10_x000d_ | 0.0 |
| Lg 22-12 1 µM_x000d_ | 0.0 |
| Lg 20-9 1 µM_x000d_ | 0.0 |
| Lg 23-11 1 µM_x000d_ | 0.0 |
| Lg 23-11 3 µM_x000d_ | 0.0 |
| Lg 23-12_x000d_ | 0.0 |
| Lg 26-8_x000d_ | 0.0 |
| Lg 26-9_x000d_ | 0.0 |
| Lg 22-13_x000d_ | 0.0 |
| Lg 26-10_x000d_ | 0.0 |
| Lg 18-7-F3 HF 1uM | 0.0 |
| Lg 19-8-F5 HF 3uM | 0.0 |
| Lg 23-11-F2 HF 3uM | 0.0 |
| Blank 1 | 0.0 |
| Blank 2 | 0.0 |
| Blank 3 | 0.0 |
| Blank 4 | 0.0 |
| Blank 5 | 0.0 |MFI
### Chart: Un-NL3
| Category | NLD-60 IgG2 |
|---|---|
| Ng 7-13 1µM_x000d_ | 76.33333333333334 |
| Ng 7-13 3µM_x000d_ | 486.0 |
| Ng 9-9 1 µM_x000d_ | 47.33333333333334 |
| Ng 9-9 3µM_x000d_ | 804.3333333333334 |
| Ng 9-9 10µM_x000d_ | 90.33333333333334 |
| Ng 10-13 1 µM_x000d_ | 2.0 |
| Ng 10-13 3 µM_x000d_ | 0.0 |
| Ng 10-13 10 µM_x000d_ | 14.666666666666657 |
| Ng 10-13 30 µM_x000d_ | 0.0 |
| Ng 10-10 1 µM_x000d_ | 3.333333333333343 |
| Ng 10-10 3 µM_x000d_ | 25.0 |
| Ng 10-11 1 µM_x000d_ | 20.0 |
| Ng 10-11 3 µM_x000d_ | 79.66666666666666 |
| Ng 10-12 1 µM_x000d_ | 0.0 |
| Ng 11-10 1 µM_x000d_ | 45.66666666666666 |
| Ng 11-10 3 µM_x000d_ | 1.3333333333333428 |
| Ng 11-11 1 µM_x000d_ | 10.0 |
| Ng 11-11 3µM_x000d_ | 0.0 |
| Ng 11-11 10µM_x000d_ | 0.0 |
| Ng 12-11 1 µM_x000d_ | 0.6666666666666572 |
| Ng 12-11 3 µM_x000d_ | 6.666666666666657 |
| Ng 13-15 1 µM_x000d_ | 0.0 |
| Ng 13-15 3 µM_x000d_ | 18.666666666666657 |
| Ng 13-14_x000d_ | 5110.333333333333 |
| Ng 14-10 1 µM_x000d_ | 1784.3333333333333 |
| Ng 14-10 3 µM_x000d_ | 12274.0 |
| Ng 14-11 1 µM_x000d_ | 101.0 |
| Ng 14-12 1 µM_x000d_ | 22341.666666666668 |
| Ng 14-7 1 µM_x000d_ | 34.66666666666666 |
| Ng 15-14/15 1 µM_x000d_ | 2083.6666666666665 |
| Ng 15-12 1µM_x000d_ | 14.0 |
| Ng 17-7 1 µM_x000d_ | 26.0 |
| Ng 17-7 3 µM_x000d_ | 84.33333333333334 |
| Ng 17-7 10 µM_x000d_ | 133.33333333333331 |
| Ng 19-9 1 µM_x000d_ | 0.0 |
| Ng 19-9 3 µM_x000d_ | 0.0 |
| Ng 19-9 10 µM_x000d_ | 58.66666666666666 |
| Ng 20-7 1 µM_x000d_ | 84.33333333333334 |
| Ng 20-7 3 µM_x000d_ | 73.0 |
| Ng 20-7 10 µM_x000d_ | 0.0 |
| Ng 21-8 1 µM_x000d_ | 0.0 |
| Ng 21-8 3 µM_x000d_ | 149.0 |
| Ng 21-8 10 µM_x000d_ | 9.666666666666657 |
| Ng 15-13 3µM_x000d_ | 201.66666666666669 |
| Ng 16-12 1 µM_x000d_ | 3.0 |
| Ng 17-9 1 µM_x000d_ | 25.333333333333343 |
| Ng 17-9 3 µM_x000d_ | 121.0 |
| Ng 18-11 1 µM_x000d_ | 105.33333333333334 |
| Ng 18-11 3 µM_x000d_ | 71.33333333333334 |
| Ng 20-8 1 µM_x000d_ | 72.0 |
| Ng 20-9 1 µM_x000d_ | 114.66666666666669 |
| Ng 20-9 3 µM_x000d_ | 410.66666666666663 |
| Ng 21-9 1 µM_x000d_ | 446.0 |
| Ng 13-13 3 µM_x000d_ | 0.0 |
| Ng 14-8 1 µM_x000d_ | 61.33333333333334 |
| Ng 14-9 1 µM_x000d_ | 37.66666666666666 |
| Ng 14-9 3 µM_x000d_ | 101.66666666666666 |
| Ng 14-9 10 µM_x000d_ | 53.66666666666666 |
| Ng 14-9 30 µM_x000d_ | 226.66666666666669 |
| Ng 15-10 1µM_x000d_ | 16.0 |
| Ng 16-9 1 µM_x000d_ | 58.66666666666666 |
| Ng 17-8 1 µM_x000d_ | 0.0 |
| Ng 17-8 3 µM_x000d_ | 0.0 |
| Ng 18-9 1µM_x000d_ | 46.0 |
| Ng 19-11 1 µM_x000d_ | 2157.3333333333335 |
| Ng 19-11 3 µM_x000d_ | 4494.333333333333 |
| Lg 17-15 HF 10 µM_x000d_ | 496.0 |
| Lg 15-17 1 µM_x000d_ | 96.0 |
| Lg 17-15 1 µM_x000d_ | 1580.3333333333333 |
| Lg 17-15 3 µM_x000d_ | 1304.6666666666667 |
| Lg 17-15 10 µM_x000d_ | 2650.0 |
| Lg 17-17 1 µM_x000d_ | 85.0 |
| Lg 17-16 1 µM_x000d_ | 367.0 |
| Lg 16-17 3 µM_x000d_ | 214.66666666666669 |
| Lg 18-8 1 µM_x000d_ | 172.33333333333331 |
| Lg 19-8 1 µM_x000d_ | 311.3333333333333 |
| Lg 19-8 3 µM_x000d_ | 2843.3333333333335 |
| Lg 19-9 1 µM_x000d_ | 351.3333333333333 |
| Lg 20-10_x000d_ | 115.66666666666669 |
| Lg 22-12 1 µM_x000d_ | 110.66666666666669 |
| Lg 20-9 1 µM_x000d_ | 105.0 |
| Lg 23-11 1 µM_x000d_ | 1499.6666666666667 |
| Lg 23-11 3 µM_x000d_ | 6827.0 |
| Lg 23-12_x000d_ | 2861.0 |
| Lg 26-8_x000d_ | 342.0 |
| Lg 26-9_x000d_ | 119.66666666666669 |
| Lg 22-13_x000d_ | 1608.3333333333333 |
| Lg 26-10_x000d_ | 189.33333333333331 |
| Lg 18-7-F3 HF 1uM | 0.0 |
| Lg 19-8-F5 HF 3uM | 0.0 |
| Lg 23-11-F2 HF 3uM | 0.0 |
| Blank 1 | 6.666666666666657 |
| Blank 2 | 5.333333333333343 |
| Blank 3 | 0.0 |
| Blank 4 | 0.0 |
| Blank 5 | 0.0 |MFI
### Chart: Un-NL4
| Category | NLD-61 IgG2 |
|---|---|
| Ng 7-13 1µM_x000d_ | 0.0 |
| Ng 7-13 3µM_x000d_ | 7.0 |
| Ng 9-9 1 µM_x000d_ | 0.0 |
| Ng 9-9 3µM_x000d_ | 223.33333333333331 |
| Ng 9-9 10µM_x000d_ | 0.0 |
| Ng 10-13 1 µM_x000d_ | 0.0 |
| Ng 10-13 3 µM_x000d_ | 0.0 |
| Ng 10-13 10 µM_x000d_ | 0.0 |
| Ng 10-13 30 µM_x000d_ | 0.0 |
| Ng 10-10 1 µM_x000d_ | 0.0 |
| Ng 10-10 3 µM_x000d_ | 0.0 |
| Ng 10-11 1 µM_x000d_ | 0.0 |
| Ng 10-11 3 µM_x000d_ | 0.0 |
| Ng 10-12 1 µM_x000d_ | 0.0 |
| Ng 11-10 1 µM_x000d_ | 0.0 |
| Ng 11-10 3 µM_x000d_ | 0.0 |
| Ng 11-11 1 µM_x000d_ | 0.0 |
| Ng 11-11 3µM_x000d_ | 0.0 |
| Ng 11-11 10µM_x000d_ | 0.0 |
| Ng 12-11 1 µM_x000d_ | 0.0 |
| Ng 12-11 3 µM_x000d_ | 0.0 |
| Ng 13-15 1 µM_x000d_ | 0.0 |
| Ng 13-15 3 µM_x000d_ | 0.0 |
| Ng 13-14_x000d_ | 383.66666666666663 |
| Ng 14-10 1 µM_x000d_ | 0.0 |
| Ng 14-10 3 µM_x000d_ | 72.33333333333334 |
| Ng 14-11 1 µM_x000d_ | 0.0 |
| Ng 14-12 1 µM_x000d_ | 215.33333333333331 |
| Ng 14-7 1 µM_x000d_ | 0.0 |
| Ng 15-14/15 1 µM_x000d_ | 0.0 |
| Ng 15-12 1µM_x000d_ | 0.0 |
| Ng 17-7 1 µM_x000d_ | 0.0 |
| Ng 17-7 3 µM_x000d_ | 0.0 |
| Ng 17-7 10 µM_x000d_ | 41.0 |
| Ng 19-9 1 µM_x000d_ | 0.0 |
| Ng 19-9 3 µM_x000d_ | 0.0 |
| Ng 19-9 10 µM_x000d_ | 0.0 |
| Ng 20-7 1 µM_x000d_ | 0.0 |
| Ng 20-7 3 µM_x000d_ | 0.0 |
| Ng 20-7 10 µM_x000d_ | 82.33333333333334 |
| Ng 21-8 1 µM_x000d_ | 0.0 |
| Ng 21-8 3 µM_x000d_ | 0.0 |
| Ng 21-8 10 µM_x000d_ | 209.33333333333331 |
| Ng 15-13 3µM_x000d_ | 86.0 |
| Ng 16-12 1 µM_x000d_ | 0.0 |
| Ng 17-9 1 µM_x000d_ | 0.0 |
| Ng 17-9 3 µM_x000d_ | 199.0 |
| Ng 18-11 1 µM_x000d_ | 27.0 |
| Ng 18-11 3 µM_x000d_ | 24.333333333333343 |
| Ng 20-8 1 µM_x000d_ | 29.0 |
| Ng 20-9 1 µM_x000d_ | 4.333333333333343 |
| Ng 20-9 3 µM_x000d_ | 82.33333333333334 |
| Ng 21-9 1 µM_x000d_ | 150.0 |
| Ng 13-13 3 µM_x000d_ | 0.0 |
| Ng 14-8 1 µM_x000d_ | 0.0 |
| Ng 14-9 1 µM_x000d_ | 0.0 |
| Ng 14-9 3 µM_x000d_ | 0.0 |
| Ng 14-9 10 µM_x000d_ | 0.0 |
| Ng 14-9 30 µM_x000d_ | 0.3333333333333428 |
| Ng 15-10 1µM_x000d_ | 0.0 |
| Ng 16-9 1 µM_x000d_ | 0.0 |
| Ng 17-8 1 µM_x000d_ | 0.0 |
| Ng 17-8 3 µM_x000d_ | 0.0 |
| Ng 18-9 1µM_x000d_ | 0.0 |
| Ng 19-11 1 µM_x000d_ | 22.666666666666657 |
| Ng 19-11 3 µM_x000d_ | 328.6666666666667 |
| Lg 17-15 HF 10 µM_x000d_ | 2.666666666666657 |
| Lg 15-17 1 µM_x000d_ | 0.0 |
| Lg 17-15 1 µM_x000d_ | 0.0 |
| Lg 17-15 3 µM_x000d_ | 57.0 |
| Lg 17-15 10 µM_x000d_ | 686.0 |
| Lg 17-17 1 µM_x000d_ | 0.0 |
| Lg 17-16 1 µM_x000d_ | 0.0 |
| Lg 16-17 3 µM_x000d_ | 188.0 |
| Lg 18-8 1 µM_x000d_ | 0.0 |
| Lg 19-8 1 µM_x000d_ | 35.0 |
| Lg 19-8 3 µM_x000d_ | 345.6666666666667 |
| Lg 19-9 1 µM_x000d_ | 8.666666666666657 |
| Lg 20-10_x000d_ | 0.0 |
| Lg 22-12 1 µM_x000d_ | 0.0 |
| Lg 20-9 1 µM_x000d_ | 94.66666666666666 |
| Lg 23-11 1 µM_x000d_ | 171.66666666666669 |
| Lg 23-11 3 µM_x000d_ | 1038.6666666666667 |
| Lg 23-12_x000d_ | 326.6666666666667 |
| Lg 26-8_x000d_ | 301.0 |
| Lg 26-9_x000d_ | 0.0 |
| Lg 22-13_x000d_ | 48.66666666666666 |
| Lg 26-10_x000d_ | 0.0 |
| Lg 18-7-F3 HF 1uM | 0.0 |
| Lg 19-8-F5 HF 3uM | 48.66666666666666 |
| Lg 23-11-F2 HF 3uM | 0.0 |
| Blank 1 | 0.0 |
| Blank 2 | 0.0 |
| Blank 3 | 0.0 |
| Blank 4 | 0.0 |
| Blank 5 | 0.0 |MFI
### Chart: Un-NL5
| Category | NLD-62 IgG2 |
|---|---|
| Ng1_1uM
 | 0.0 |
| Ng1_3uM
 | 94.33333333333334 |
| Ng2_1uM | 0.0 |
| Ng2_3uM | 40.33333333333334 |
| Ng2_10uM | 11.666666666666657 |
| Ng3_1uM | 0.0 |
| Ng3_3uM | 0.0 |
| Ng3_10uM | 0.0 |
| Ng3_30uM | 3.333333333333343 |
| Ng4_1uM | 0.0 |
| Ng4_3uM | 9.0 |
| Ng5_1uM | 0.0 |
| Ng5_3uM | 7.333333333333343 |
| Ng6_1uM | 1.0 |
| Ng7_1uM | 0.0 |
| Ng7_3uM | 4.333333333333343 |
| Ng8_1uM | 0.0 |
| Ng8_3uM | 0.0 |
| Ng8_10uM | 0.0 |
| Ng9_1uM | 0.0 |
| Ng9_3uM | 0.0 |
| Ng10_3uM | 0.0 |
| Ng10_1uM | 0.0 |
| Ng11 | 2532.0 |
| Ng12_1uM | 4.666666666666657 |
| Ng12_3uM | 61.66666666666666 |
| Ng13_1uM | 0.0 |
| Ng14_1uM | 560.3333333333334 |
| Ng15_1uM | 0.0 |
| Ng16_1uM | 73.0 |
| Ng17_1uM | 0.0 |
| Ng18_1uM | 0.0 |
| Ng18_3uM | 0.0 |
| Ng18_10uM | 56.66666666666666 |
| Ng19_1uM | 0.0 |
| Ng19_3uM | 41.66666666666666 |
| Ng19_10uM | 32.66666666666666 |
| Ng20_1uM | 0.0 |
| Ng20_3uM | 0.0 |
| Ng20_10uM | 198.33333333333331 |
| Ng21_1uM | 0.0 |
| Ng21 _3uM | 95.0 |
| Ng21_10uM | 1152.0 |
| Ng22_1uM | 120.66666666666669 |
| Ng23_1uM | 0.0 |
| Ng24_1uM | 55.66666666666666 |
| Ng24_3uM | 183.0 |
| Ng25_1uM | 707.3333333333334 |
| Ng25_3uM | 613.3333333333334 |
| Ng26_1uM | 107.66666666666669 |
| Ng27_1uM | 469.0 |
| Ng27_3uM | 1044.0 |
| Ng28_1uM | 312.6666666666667 |
| Ng29_1uM | 14.0 |
| Ng30_1uM | 0.0 |
| Ng31_1uM | 0.0 |
| Ng31_3uM | 0.6666666666666572 |
| Ng31_10uM | 58.33333333333334 |
| Ng31_30uM | 10.0 |
| Ng32_1uM | 0.0 |
| Ng33_1uM | 0.0 |
| Ng34_1uM | 84.33333333333334 |
| Ng34_3uM | 377.66666666666663 |
| Ng35_1uM | 24.0 |
| Ng36_1uM | 898.0 |
| Ng36_3uM | 1252.0 |
| Lg2_HF_10uM | 32.0 |
| Lg1_1uM | 0.0 |
| Lg2_1uM | 88.66666666666666 |
| Lg2_3uM | 1351.3333333333333 |
| Lg2_10uM | 4239.0 |
| Lg3_1uM | 46.0 |
| Lg4_1uM | 209.66666666666669 |
| Lg5_3uM | 804.0 |
| Lg6_1uM | 447.33333333333337 |
| Lg7_1uM | 589.6666666666666 |
| Lg7_3uM | 2447.3333333333335 |
| Lg8_1uM | 398.0 |
| Lg9 | 558.6666666666666 |
| Lg10_1uM | 111.0 |
| Lg11_1uM | 58.0 |
| Lg12_1uM | 610.0 |
| Lg12_3uM | 4036.333333333333 |
| Lg13 | 3406.0 |
| Lg14 | 2416.6666666666665 |
| Lg15 | 1182.0 |
| Lg16 | 1755.6666666666667 |
| Lg17 | 35.33333333333334 |
| Lg18_HF_1uM | 0.0 |
| Lg7_HF_3uM | 0.0 |
| Lg12_HF_3uM | 0.0 |
| Blank 1 | 0.0 |
| Blank 2 | 0.0 |
| Blank 3 | 0.0 |
| Blank 4 | 0.0 |
| Blank 5 | 0.0 |MFI

## Slide 36
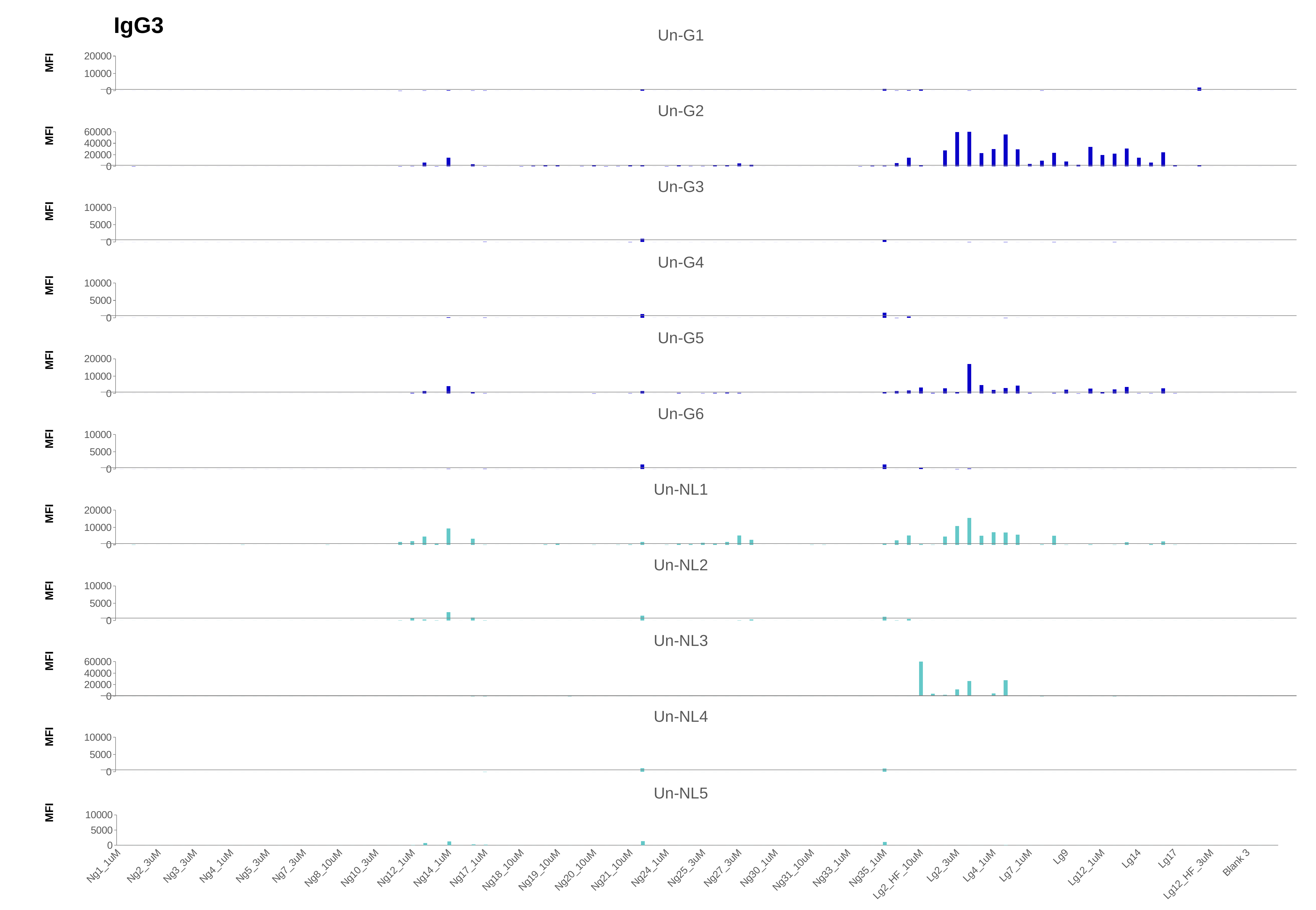

IgG3
### Chart: Un-G1
| Category | UG 000-214 IgG3 |
|---|---|
| Ng 7-13 1µM_x000d_ | 0.0 |
| Ng 7-13 3µM_x000d_ | 0.0 |
| Ng 9-9 1 µM_x000d_ | 0.0 |
| Ng 9-9 3µM_x000d_ | 0.0 |
| Ng 9-9 10µM_x000d_ | 0.0 |
| Ng 10-13 1 µM_x000d_ | 0.0 |
| Ng 10-13 3 µM_x000d_ | 0.0 |
| Ng 10-13 10 µM_x000d_ | 0.0 |
| Ng 10-13 30 µM_x000d_ | 0.0 |
| Ng 10-10 1 µM_x000d_ | 0.0 |
| Ng 10-10 3 µM_x000d_ | 0.0 |
| Ng 10-11 1 µM_x000d_ | 0.0 |
| Ng 10-11 3 µM_x000d_ | 0.0 |
| Ng 10-12 1 µM_x000d_ | 0.0 |
| Ng 11-10 1 µM_x000d_ | 0.0 |
| Ng 11-10 3 µM_x000d_ | 0.0 |
| Ng 11-11 1 µM_x000d_ | 0.0 |
| Ng 11-11 3µM_x000d_ | 0.0 |
| Ng 11-11 10µM_x000d_ | 0.0 |
| Ng 12-11 1 µM_x000d_ | 0.0 |
| Ng 12-11 3 µM_x000d_ | 0.0 |
| Ng 13-15 1 µM_x000d_ | 0.0 |
| Ng 13-15 3 µM_x000d_ | 0.0 |
| Ng 13-14_x000d_ | 5.333333333333343 |
| Ng 14-10 1 µM_x000d_ | 0.0 |
| Ng 14-10 3 µM_x000d_ | 106.0 |
| Ng 14-11 1 µM_x000d_ | 0.0 |
| Ng 14-12 1 µM_x000d_ | 253.66666666666669 |
| Ng 14-7 1 µM_x000d_ | 0.0 |
| Ng 15-14/15 1 µM_x000d_ | 64.33333333333334 |
| Ng 15-12 1µM_x000d_ | 44.0 |
| Ng 17-7 1 µM_x000d_ | 0.0 |
| Ng 17-7 3 µM_x000d_ | 0.0 |
| Ng 17-7 10 µM_x000d_ | 0.0 |
| Ng 19-9 1 µM_x000d_ | 0.0 |
| Ng 19-9 3 µM_x000d_ | 0.0 |
| Ng 19-9 10 µM_x000d_ | 0.0 |
| Ng 20-7 1 µM_x000d_ | 0.0 |
| Ng 20-7 3 µM_x000d_ | 0.0 |
| Ng 20-7 10 µM_x000d_ | 0.0 |
| Ng 21-8 1 µM_x000d_ | 0.0 |
| Ng 21-8 3 µM_x000d_ | 0.0 |
| Ng 21-8 10 µM_x000d_ | 0.0 |
| Ng 15-13 3µM_x000d_ | 907.0 |
| Ng 16-12 1 µM_x000d_ | 0.0 |
| Ng 17-9 1 µM_x000d_ | 0.0 |
| Ng 17-9 3 µM_x000d_ | 0.0 |
| Ng 18-11 1 µM_x000d_ | 0.0 |
| Ng 18-11 3 µM_x000d_ | 0.0 |
| Ng 20-8 1 µM_x000d_ | 0.0 |
| Ng 20-9 1 µM_x000d_ | 0.0 |
| Ng 20-9 3 µM_x000d_ | 0.0 |
| Ng 21-9 1 µM_x000d_ | 0.0 |
| Ng 13-13 3 µM_x000d_ | 0.0 |
| Ng 14-8 1 µM_x000d_ | 0.0 |
| Ng 14-9 1 µM_x000d_ | 0.0 |
| Ng 14-9 3 µM_x000d_ | 0.0 |
| Ng 14-9 10 µM_x000d_ | 0.0 |
| Ng 14-9 30 µM_x000d_ | 0.0 |
| Ng 15-10 1µM_x000d_ | 0.0 |
| Ng 16-9 1 µM_x000d_ | 0.0 |
| Ng 17-8 1 µM_x000d_ | 0.0 |
| Ng 17-8 3 µM_x000d_ | 0.0 |
| Ng 18-9 1µM_x000d_ | 1047.6666666666667 |
| Ng 19-11 1 µM_x000d_ | 43.0 |
| Ng 19-11 3 µM_x000d_ | 301.3333333333333 |
| Lg 17-15 HF 10 µM_x000d_ | 520.3333333333334 |
| Lg 15-17 1 µM_x000d_ | 0.0 |
| Lg 17-15 1 µM_x000d_ | 0.0 |
| Lg 17-15 3 µM_x000d_ | 0.0 |
| Lg 17-15 10 µM_x000d_ | 164.33333333333331 |
| Lg 17-17 1 µM_x000d_ | 0.0 |
| Lg 17-16 1 µM_x000d_ | 0.0 |
| Lg 16-17 3 µM_x000d_ | 0.0 |
| Lg 18-8 1 µM_x000d_ | 0.0 |
| Lg 19-8 1 µM_x000d_ | 0.0 |
| Lg 19-8 3 µM_x000d_ | 51.33333333333334 |
| Lg 19-9 1 µM_x000d_ | 0.0 |
| Lg 20-10_x000d_ | 0.0 |
| Lg 22-12 1 µM_x000d_ | 0.0 |
| Lg 20-9 1 µM_x000d_ | 0.0 |
| Lg 23-11 1 µM_x000d_ | 0.0 |
| Lg 23-11 3 µM_x000d_ | 0.0 |
| Lg 23-12_x000d_ | 0.0 |
| Lg 26-8_x000d_ | 0.0 |
| Lg 26-9_x000d_ | 0.0 |
| Lg 22-13_x000d_ | 0.0 |
| Lg 26-10_x000d_ | 0.0 |
| Lg 18-7-F3 HF 1uM | 0.0 |
| Lg 19-8-F5 HF 3uM | 1752.6666666666667 |
| Lg 23-11-F2 HF 3uM | 0.0 |
| Blank 1 | 0.0 |
| Blank 2 | 0.0 |
| Blank 3 | 0.0 |
| Blank 4 | 0.0 |
| Blank 5 | 0.0 |MFI
### Chart: Un-G2
| Category | UG 000-215 IgG3 |
|---|---|
| Ng 7-13 1µM_x000d_ | 0.0 |
| Ng 7-13 3µM_x000d_ | 146.0 |
| Ng 9-9 1 µM_x000d_ | 0.0 |
| Ng 9-9 3µM_x000d_ | 0.0 |
| Ng 9-9 10µM_x000d_ | 0.0 |
| Ng 10-13 1 µM_x000d_ | 0.0 |
| Ng 10-13 3 µM_x000d_ | 0.0 |
| Ng 10-13 10 µM_x000d_ | 0.0 |
| Ng 10-13 30 µM_x000d_ | 0.0 |
| Ng 10-10 1 µM_x000d_ | 0.0 |
| Ng 10-10 3 µM_x000d_ | 0.0 |
| Ng 10-11 1 µM_x000d_ | 0.0 |
| Ng 10-11 3 µM_x000d_ | 0.0 |
| Ng 10-12 1 µM_x000d_ | 0.0 |
| Ng 11-10 1 µM_x000d_ | 0.0 |
| Ng 11-10 3 µM_x000d_ | 0.0 |
| Ng 11-11 1 µM_x000d_ | 0.0 |
| Ng 11-11 3µM_x000d_ | 0.0 |
| Ng 11-11 10µM_x000d_ | 0.0 |
| Ng 12-11 1 µM_x000d_ | 0.0 |
| Ng 12-11 3 µM_x000d_ | 0.0 |
| Ng 13-15 1 µM_x000d_ | 0.0 |
| Ng 13-15 3 µM_x000d_ | 0.0 |
| Ng 13-14_x000d_ | 162.33333333333331 |
| Ng 14-10 1 µM_x000d_ | 549.6666666666666 |
| Ng 14-10 3 µM_x000d_ | 6331.333333333333 |
| Ng 14-11 1 µM_x000d_ | 12.333333333333343 |
| Ng 14-12 1 µM_x000d_ | 14913.0 |
| Ng 14-7 1 µM_x000d_ | 0.0 |
| Ng 15-14/15 1 µM_x000d_ | 3701.6666666666665 |
| Ng 15-12 1µM_x000d_ | 90.0 |
| Ng 17-7 1 µM_x000d_ | 0.0 |
| Ng 17-7 3 µM_x000d_ | 0.0 |
| Ng 17-7 10 µM_x000d_ | 4.0 |
| Ng 19-9 1 µM_x000d_ | 796.6666666666666 |
| Ng 19-9 3 µM_x000d_ | 1721.0 |
| Ng 19-9 10 µM_x000d_ | 2055.3333333333335 |
| Ng 20-7 1 µM_x000d_ | 0.0 |
| Ng 20-7 3 µM_x000d_ | 473.66666666666663 |
| Ng 20-7 10 µM_x000d_ | 1976.0 |
| Ng 21-8 1 µM_x000d_ | 7.0 |
| Ng 21-8 3 µM_x000d_ | 264.6666666666667 |
| Ng 21-8 10 µM_x000d_ | 2325.0 |
| Ng 15-13 3µM_x000d_ | 1516.3333333333333 |
| Ng 16-12 1 µM_x000d_ | 0.0 |
| Ng 17-9 1 µM_x000d_ | 205.0 |
| Ng 17-9 3 µM_x000d_ | 1400.3333333333333 |
| Ng 18-11 1 µM_x000d_ | 502.33333333333337 |
| Ng 18-11 3 µM_x000d_ | 367.0 |
| Ng 20-8 1 µM_x000d_ | 1708.6666666666667 |
| Ng 20-9 1 µM_x000d_ | 2203.3333333333335 |
| Ng 20-9 3 µM_x000d_ | 5354.333333333333 |
| Ng 21-9 1 µM_x000d_ | 2865.0 |
| Ng 13-13 3 µM_x000d_ | 0.0 |
| Ng 14-8 1 µM_x000d_ | 0.0 |
| Ng 14-9 1 µM_x000d_ | 0.0 |
| Ng 14-9 3 µM_x000d_ | 0.0 |
| Ng 14-9 10 µM_x000d_ | 0.0 |
| Ng 14-9 30 µM_x000d_ | 0.0 |
| Ng 15-10 1µM_x000d_ | 0.0 |
| Ng 16-9 1 µM_x000d_ | 0.0 |
| Ng 17-8 1 µM_x000d_ | 66.33333333333334 |
| Ng 17-8 3 µM_x000d_ | 719.6666666666666 |
| Ng 18-9 1µM_x000d_ | 1003.6666666666667 |
| Ng 19-11 1 µM_x000d_ | 5625.0 |
| Ng 19-11 3 µM_x000d_ | 14841.666666666666 |
| Lg 17-15 HF 10 µM_x000d_ | 1476.6666666666667 |
| Lg 15-17 1 µM_x000d_ | 0.0 |
| Lg 17-15 1 µM_x000d_ | 27629.666666666668 |
| Lg 17-15 3 µM_x000d_ | 59285.666666666664 |
| Lg 17-15 10 µM_x000d_ | 64908.0 |
| Lg 17-17 1 µM_x000d_ | 22775.666666666668 |
| Lg 17-16 1 µM_x000d_ | 29735.333333333332 |
| Lg 16-17 3 µM_x000d_ | 55312.333333333336 |
| Lg 18-8 1 µM_x000d_ | 29449.333333333332 |
| Lg 19-8 1 µM_x000d_ | 4077.0 |
| Lg 19-8 3 µM_x000d_ | 9740.666666666666 |
| Lg 19-9 1 µM_x000d_ | 23422.0 |
| Lg 20-10_x000d_ | 8467.333333333334 |
| Lg 22-12 1 µM_x000d_ | 2704.0 |
| Lg 20-9 1 µM_x000d_ | 33646.0 |
| Lg 23-11 1 µM_x000d_ | 19825.0 |
| Lg 23-11 3 µM_x000d_ | 21802.666666666668 |
| Lg 23-12_x000d_ | 30857.333333333332 |
| Lg 26-8_x000d_ | 14996.333333333334 |
| Lg 26-9_x000d_ | 6749.666666666667 |
| Lg 22-13_x000d_ | 24525.333333333332 |
| Lg 26-10_x000d_ | 1355.6666666666667 |
| Lg 18-7-F3 HF 1uM | 0.0 |
| Lg 19-8-F5 HF 3uM | 1273.0 |
| Lg 23-11-F2 HF 3uM | 0.0 |
| Blank 1 | 0.0 |
| Blank 2 | 0.0 |
| Blank 3 | 0.0 |
| Blank 4 | 0.0 |
| Blank 5 | 0.0 |MFI
### Chart: Un-G3
| Category | UG 000-223 IgG3 |
|---|---|
| Ng 7-13 1µM_x000d_ | 0.0 |
| Ng 7-13 3µM_x000d_ | 0.0 |
| Ng 9-9 1 µM_x000d_ | 0.0 |
| Ng 9-9 3µM_x000d_ | 0.0 |
| Ng 9-9 10µM_x000d_ | 0.0 |
| Ng 10-13 1 µM_x000d_ | 0.0 |
| Ng 10-13 3 µM_x000d_ | 0.0 |
| Ng 10-13 10 µM_x000d_ | 0.0 |
| Ng 10-13 30 µM_x000d_ | 0.0 |
| Ng 10-10 1 µM_x000d_ | 0.0 |
| Ng 10-10 3 µM_x000d_ | 0.0 |
| Ng 10-11 1 µM_x000d_ | 0.0 |
| Ng 10-11 3 µM_x000d_ | 0.0 |
| Ng 10-12 1 µM_x000d_ | 0.0 |
| Ng 11-10 1 µM_x000d_ | 0.0 |
| Ng 11-10 3 µM_x000d_ | 0.0 |
| Ng 11-11 1 µM_x000d_ | 0.0 |
| Ng 11-11 3µM_x000d_ | 0.0 |
| Ng 11-11 10µM_x000d_ | 0.0 |
| Ng 12-11 1 µM_x000d_ | 0.0 |
| Ng 12-11 3 µM_x000d_ | 0.0 |
| Ng 13-15 1 µM_x000d_ | 0.0 |
| Ng 13-15 3 µM_x000d_ | 0.0 |
| Ng 13-14_x000d_ | 0.0 |
| Ng 14-10 1 µM_x000d_ | 0.0 |
| Ng 14-10 3 µM_x000d_ | 0.0 |
| Ng 14-11 1 µM_x000d_ | 0.0 |
| Ng 14-12 1 µM_x000d_ | 0.0 |
| Ng 14-7 1 µM_x000d_ | 0.0 |
| Ng 15-14/15 1 µM_x000d_ | 0.0 |
| Ng 15-12 1µM_x000d_ | 81.66666666666666 |
| Ng 17-7 1 µM_x000d_ | 0.0 |
| Ng 17-7 3 µM_x000d_ | 0.0 |
| Ng 17-7 10 µM_x000d_ | 0.0 |
| Ng 19-9 1 µM_x000d_ | 0.0 |
| Ng 19-9 3 µM_x000d_ | 0.0 |
| Ng 19-9 10 µM_x000d_ | 0.0 |
| Ng 20-7 1 µM_x000d_ | 0.0 |
| Ng 20-7 3 µM_x000d_ | 0.0 |
| Ng 20-7 10 µM_x000d_ | 0.0 |
| Ng 21-8 1 µM_x000d_ | 0.0 |
| Ng 21-8 3 µM_x000d_ | 0.0 |
| Ng 21-8 10 µM_x000d_ | 3.666666666666657 |
| Ng 15-13 3µM_x000d_ | 935.6666666666667 |
| Ng 16-12 1 µM_x000d_ | 0.0 |
| Ng 17-9 1 µM_x000d_ | 0.0 |
| Ng 17-9 3 µM_x000d_ | 0.0 |
| Ng 18-11 1 µM_x000d_ | 0.0 |
| Ng 18-11 3 µM_x000d_ | 0.0 |
| Ng 20-8 1 µM_x000d_ | 0.0 |
| Ng 20-9 1 µM_x000d_ | 0.0 |
| Ng 20-9 3 µM_x000d_ | 0.0 |
| Ng 21-9 1 µM_x000d_ | 0.0 |
| Ng 13-13 3 µM_x000d_ | 0.0 |
| Ng 14-8 1 µM_x000d_ | 0.0 |
| Ng 14-9 1 µM_x000d_ | 0.0 |
| Ng 14-9 3 µM_x000d_ | 0.0 |
| Ng 14-9 10 µM_x000d_ | 0.0 |
| Ng 14-9 30 µM_x000d_ | 0.0 |
| Ng 15-10 1µM_x000d_ | 0.0 |
| Ng 16-9 1 µM_x000d_ | 0.0 |
| Ng 17-8 1 µM_x000d_ | 0.0 |
| Ng 17-8 3 µM_x000d_ | 0.0 |
| Ng 18-9 1µM_x000d_ | 687.3333333333334 |
| Ng 19-11 1 µM_x000d_ | 0.0 |
| Ng 19-11 3 µM_x000d_ | 0.0 |
| Lg 17-15 HF 10 µM_x000d_ | 0.0 |
| Lg 15-17 1 µM_x000d_ | 0.0 |
| Lg 17-15 1 µM_x000d_ | 0.0 |
| Lg 17-15 3 µM_x000d_ | 0.0 |
| Lg 17-15 10 µM_x000d_ | 6.666666666666657 |
| Lg 17-17 1 µM_x000d_ | 0.0 |
| Lg 17-16 1 µM_x000d_ | 0.0 |
| Lg 16-17 3 µM_x000d_ | 9.333333333333343 |
| Lg 18-8 1 µM_x000d_ | 0.0 |
| Lg 19-8 1 µM_x000d_ | 0.0 |
| Lg 19-8 3 µM_x000d_ | 0.0 |
| Lg 19-9 1 µM_x000d_ | 1.0 |
| Lg 20-10_x000d_ | 0.0 |
| Lg 22-12 1 µM_x000d_ | 0.0 |
| Lg 20-9 1 µM_x000d_ | 0.0 |
| Lg 23-11 1 µM_x000d_ | 0.0 |
| Lg 23-11 3 µM_x000d_ | 2.333333333333343 |
| Lg 23-12_x000d_ | 0.0 |
| Lg 26-8_x000d_ | 0.0 |
| Lg 26-9_x000d_ | 0.0 |
| Lg 22-13_x000d_ | 0.0 |
| Lg 26-10_x000d_ | 0.0 |
| Lg 18-7-F3 HF 1uM | 0.0 |
| Lg 19-8-F5 HF 3uM | 0.0 |
| Lg 23-11-F2 HF 3uM | 0.0 |
| Blank 1 | 0.0 |
| Blank 2 | 0.0 |
| Blank 3 | 0.0 |
| Blank 4 | 0.0 |
| Blank 5 | 0.0 |MFI
### Chart: Un-G4
| Category | UG 000-224 IgG3 |
|---|---|
| Ng 7-13 1µM_x000d_ | 0.0 |
| Ng 7-13 3µM_x000d_ | 0.0 |
| Ng 9-9 1 µM_x000d_ | 0.0 |
| Ng 9-9 3µM_x000d_ | 0.0 |
| Ng 9-9 10µM_x000d_ | 0.0 |
| Ng 10-13 1 µM_x000d_ | 0.0 |
| Ng 10-13 3 µM_x000d_ | 0.0 |
| Ng 10-13 10 µM_x000d_ | 0.0 |
| Ng 10-13 30 µM_x000d_ | 0.0 |
| Ng 10-10 1 µM_x000d_ | 0.0 |
| Ng 10-10 3 µM_x000d_ | 0.0 |
| Ng 10-11 1 µM_x000d_ | 0.0 |
| Ng 10-11 3 µM_x000d_ | 0.0 |
| Ng 10-12 1 µM_x000d_ | 0.0 |
| Ng 11-10 1 µM_x000d_ | 0.0 |
| Ng 11-10 3 µM_x000d_ | 0.0 |
| Ng 11-11 1 µM_x000d_ | 0.0 |
| Ng 11-11 3µM_x000d_ | 0.0 |
| Ng 11-11 10µM_x000d_ | 0.0 |
| Ng 12-11 1 µM_x000d_ | 0.0 |
| Ng 12-11 3 µM_x000d_ | 0.0 |
| Ng 13-15 1 µM_x000d_ | 0.0 |
| Ng 13-15 3 µM_x000d_ | 0.0 |
| Ng 13-14_x000d_ | 0.0 |
| Ng 14-10 1 µM_x000d_ | 0.0 |
| Ng 14-10 3 µM_x000d_ | 0.0 |
| Ng 14-11 1 µM_x000d_ | 0.0 |
| Ng 14-12 1 µM_x000d_ | 129.33333333333331 |
| Ng 14-7 1 µM_x000d_ | 0.0 |
| Ng 15-14/15 1 µM_x000d_ | 0.0 |
| Ng 15-12 1µM_x000d_ | 30.0 |
| Ng 17-7 1 µM_x000d_ | 0.0 |
| Ng 17-7 3 µM_x000d_ | 0.0 |
| Ng 17-7 10 µM_x000d_ | 0.0 |
| Ng 19-9 1 µM_x000d_ | 0.0 |
| Ng 19-9 3 µM_x000d_ | 0.0 |
| Ng 19-9 10 µM_x000d_ | 0.0 |
| Ng 20-7 1 µM_x000d_ | 0.0 |
| Ng 20-7 3 µM_x000d_ | 0.0 |
| Ng 20-7 10 µM_x000d_ | 0.0 |
| Ng 21-8 1 µM_x000d_ | 0.0 |
| Ng 21-8 3 µM_x000d_ | 0.0 |
| Ng 21-8 10 µM_x000d_ | 0.0 |
| Ng 15-13 3µM_x000d_ | 1099.3333333333333 |
| Ng 16-12 1 µM_x000d_ | 0.0 |
| Ng 17-9 1 µM_x000d_ | 0.0 |
| Ng 17-9 3 µM_x000d_ | 0.0 |
| Ng 18-11 1 µM_x000d_ | 0.0 |
| Ng 18-11 3 µM_x000d_ | 0.0 |
| Ng 20-8 1 µM_x000d_ | 0.0 |
| Ng 20-9 1 µM_x000d_ | 0.0 |
| Ng 20-9 3 µM_x000d_ | 0.0 |
| Ng 21-9 1 µM_x000d_ | 0.0 |
| Ng 13-13 3 µM_x000d_ | 0.0 |
| Ng 14-8 1 µM_x000d_ | 0.0 |
| Ng 14-9 1 µM_x000d_ | 0.0 |
| Ng 14-9 3 µM_x000d_ | 0.0 |
| Ng 14-9 10 µM_x000d_ | 0.0 |
| Ng 14-9 30 µM_x000d_ | 0.0 |
| Ng 15-10 1µM_x000d_ | 0.0 |
| Ng 16-9 1 µM_x000d_ | 0.0 |
| Ng 17-8 1 µM_x000d_ | 0.0 |
| Ng 17-8 3 µM_x000d_ | 0.0 |
| Ng 18-9 1µM_x000d_ | 1410.0 |
| Ng 19-11 1 µM_x000d_ | 3.333333333333343 |
| Ng 19-11 3 µM_x000d_ | 374.0 |
| Lg 17-15 HF 10 µM_x000d_ | 0.0 |
| Lg 15-17 1 µM_x000d_ | 0.0 |
| Lg 17-15 1 µM_x000d_ | 0.0 |
| Lg 17-15 3 µM_x000d_ | 0.0 |
| Lg 17-15 10 µM_x000d_ | 0.0 |
| Lg 17-17 1 µM_x000d_ | 0.0 |
| Lg 17-16 1 µM_x000d_ | 0.0 |
| Lg 16-17 3 µM_x000d_ | 1.6666666666666572 |
| Lg 18-8 1 µM_x000d_ | 0.0 |
| Lg 19-8 1 µM_x000d_ | 0.0 |
| Lg 19-8 3 µM_x000d_ | 0.0 |
| Lg 19-9 1 µM_x000d_ | 0.0 |
| Lg 20-10_x000d_ | 0.0 |
| Lg 22-12 1 µM_x000d_ | 0.0 |
| Lg 20-9 1 µM_x000d_ | 0.0 |
| Lg 23-11 1 µM_x000d_ | 0.0 |
| Lg 23-11 3 µM_x000d_ | 0.0 |
| Lg 23-12_x000d_ | 0.0 |
| Lg 26-8_x000d_ | 0.0 |
| Lg 26-9_x000d_ | 0.0 |
| Lg 22-13_x000d_ | 0.0 |
| Lg 26-10_x000d_ | 0.0 |
| Lg 18-7-F3 HF 1uM | 0.0 |
| Lg 19-8-F5 HF 3uM | 0.0 |
| Lg 23-11-F2 HF 3uM | 0.0 |
| Blank 1 | 0.0 |
| Blank 2 | 0.0 |
| Blank 3 | 0.0 |
| Blank 4 | 0.0 |
| Blank 5 | 0.0 |MFI
### Chart: Un-G5
| Category | UG 000-225 IgG3 |
|---|---|
| Ng 7-13 1µM_x000d_ | 0.0 |
| Ng 7-13 3µM_x000d_ | 0.0 |
| Ng 9-9 1 µM_x000d_ | 0.0 |
| Ng 9-9 3µM_x000d_ | 0.0 |
| Ng 9-9 10µM_x000d_ | 0.0 |
| Ng 10-13 1 µM_x000d_ | 0.0 |
| Ng 10-13 3 µM_x000d_ | 0.0 |
| Ng 10-13 10 µM_x000d_ | 0.0 |
| Ng 10-13 30 µM_x000d_ | 0.0 |
| Ng 10-10 1 µM_x000d_ | 0.0 |
| Ng 10-10 3 µM_x000d_ | 0.0 |
| Ng 10-11 1 µM_x000d_ | 0.0 |
| Ng 10-11 3 µM_x000d_ | 0.0 |
| Ng 10-12 1 µM_x000d_ | 0.0 |
| Ng 11-10 1 µM_x000d_ | 0.0 |
| Ng 11-10 3 µM_x000d_ | 0.0 |
| Ng 11-11 1 µM_x000d_ | 0.0 |
| Ng 11-11 3µM_x000d_ | 0.0 |
| Ng 11-11 10µM_x000d_ | 0.0 |
| Ng 12-11 1 µM_x000d_ | 0.0 |
| Ng 12-11 3 µM_x000d_ | 0.0 |
| Ng 13-15 1 µM_x000d_ | 0.0 |
| Ng 13-15 3 µM_x000d_ | 0.0 |
| Ng 13-14_x000d_ | 0.0 |
| Ng 14-10 1 µM_x000d_ | 218.33333333333331 |
| Ng 14-10 3 µM_x000d_ | 1420.3333333333333 |
| Ng 14-11 1 µM_x000d_ | 0.0 |
| Ng 14-12 1 µM_x000d_ | 4225.0 |
| Ng 14-7 1 µM_x000d_ | 0.0 |
| Ng 15-14/15 1 µM_x000d_ | 807.0 |
| Ng 15-12 1µM_x000d_ | 97.66666666666666 |
| Ng 17-7 1 µM_x000d_ | 0.0 |
| Ng 17-7 3 µM_x000d_ | 0.0 |
| Ng 17-7 10 µM_x000d_ | 0.0 |
| Ng 19-9 1 µM_x000d_ | 0.0 |
| Ng 19-9 3 µM_x000d_ | 0.0 |
| Ng 19-9 10 µM_x000d_ | 0.0 |
| Ng 20-7 1 µM_x000d_ | 0.0 |
| Ng 20-7 3 µM_x000d_ | 0.0 |
| Ng 20-7 10 µM_x000d_ | 21.0 |
| Ng 21-8 1 µM_x000d_ | 0.0 |
| Ng 21-8 3 µM_x000d_ | 0.0 |
| Ng 21-8 10 µM_x000d_ | 158.0 |
| Ng 15-13 3µM_x000d_ | 1322.6666666666667 |
| Ng 16-12 1 µM_x000d_ | 0.0 |
| Ng 17-9 1 µM_x000d_ | 0.0 |
| Ng 17-9 3 µM_x000d_ | 272.3333333333333 |
| Ng 18-11 1 µM_x000d_ | 0.0 |
| Ng 18-11 3 µM_x000d_ | 73.33333333333334 |
| Ng 20-8 1 µM_x000d_ | 277.0 |
| Ng 20-9 1 µM_x000d_ | 470.0 |
| Ng 20-9 3 µM_x000d_ | 262.6666666666667 |
| Ng 21-9 1 µM_x000d_ | 0.0 |
| Ng 13-13 3 µM_x000d_ | 0.0 |
| Ng 14-8 1 µM_x000d_ | 0.0 |
| Ng 14-9 1 µM_x000d_ | 0.0 |
| Ng 14-9 3 µM_x000d_ | 0.0 |
| Ng 14-9 10 µM_x000d_ | 0.0 |
| Ng 14-9 30 µM_x000d_ | 0.0 |
| Ng 15-10 1µM_x000d_ | 0.0 |
| Ng 16-9 1 µM_x000d_ | 0.0 |
| Ng 17-8 1 µM_x000d_ | 0.0 |
| Ng 17-8 3 µM_x000d_ | 0.0 |
| Ng 18-9 1µM_x000d_ | 778.3333333333334 |
| Ng 19-11 1 µM_x000d_ | 1385.6666666666667 |
| Ng 19-11 3 µM_x000d_ | 1727.3333333333333 |
| Lg 17-15 HF 10 µM_x000d_ | 3361.3333333333335 |
| Lg 15-17 1 µM_x000d_ | 260.0 |
| Lg 17-15 1 µM_x000d_ | 2948.0 |
| Lg 17-15 3 µM_x000d_ | 676.3333333333334 |
| Lg 17-15 10 µM_x000d_ | 16944.666666666668 |
| Lg 17-17 1 µM_x000d_ | 4833.0 |
| Lg 17-16 1 µM_x000d_ | 1942.6666666666665 |
| Lg 16-17 3 µM_x000d_ | 3158.0 |
| Lg 18-8 1 µM_x000d_ | 4486.333333333333 |
| Lg 19-8 1 µM_x000d_ | 225.0 |
| Lg 19-8 3 µM_x000d_ | 0.0 |
| Lg 19-9 1 µM_x000d_ | 331.6666666666667 |
| Lg 20-10_x000d_ | 2088.3333333333335 |
| Lg 22-12 1 µM_x000d_ | 0.6666666666666572 |
| Lg 20-9 1 µM_x000d_ | 2861.6666666666665 |
| Lg 23-11 1 µM_x000d_ | 928.0 |
| Lg 23-11 3 µM_x000d_ | 2374.0 |
| Lg 23-12_x000d_ | 3664.6666666666665 |
| Lg 26-8_x000d_ | 148.66666666666669 |
| Lg 26-9_x000d_ | 129.0 |
| Lg 22-13_x000d_ | 2944.6666666666665 |
| Lg 26-10_x000d_ | 162.0 |
| Lg 18-7-F3 HF 1uM | 0.0 |
| Lg 19-8-F5 HF 3uM | 0.0 |
| Lg 23-11-F2 HF 3uM | 0.0 |
| Blank 1 | 0.0 |
| Blank 2 | 0.0 |
| Blank 3 | 0.0 |
| Blank 4 | 0.0 |
| Blank 5 | 0.0 |MFI
### Chart: Un-G6
| Category | UG 000-226 IgG3 |
|---|---|
| Ng 7-13 1µM_x000d_ | 0.0 |
| Ng 7-13 3µM_x000d_ | 0.0 |
| Ng 9-9 1 µM_x000d_ | 0.0 |
| Ng 9-9 3µM_x000d_ | 0.0 |
| Ng 9-9 10µM_x000d_ | 0.0 |
| Ng 10-13 1 µM_x000d_ | 0.0 |
| Ng 10-13 3 µM_x000d_ | 0.0 |
| Ng 10-13 10 µM_x000d_ | 0.0 |
| Ng 10-13 30 µM_x000d_ | 0.0 |
| Ng 10-10 1 µM_x000d_ | 0.0 |
| Ng 10-10 3 µM_x000d_ | 0.0 |
| Ng 10-11 1 µM_x000d_ | 0.0 |
| Ng 10-11 3 µM_x000d_ | 0.0 |
| Ng 10-12 1 µM_x000d_ | 0.0 |
| Ng 11-10 1 µM_x000d_ | 0.0 |
| Ng 11-10 3 µM_x000d_ | 0.0 |
| Ng 11-11 1 µM_x000d_ | 0.0 |
| Ng 11-11 3µM_x000d_ | 0.0 |
| Ng 11-11 10µM_x000d_ | 0.0 |
| Ng 12-11 1 µM_x000d_ | 0.0 |
| Ng 12-11 3 µM_x000d_ | 0.0 |
| Ng 13-15 1 µM_x000d_ | 0.0 |
| Ng 13-15 3 µM_x000d_ | 0.0 |
| Ng 13-14_x000d_ | 0.0 |
| Ng 14-10 1 µM_x000d_ | 0.0 |
| Ng 14-10 3 µM_x000d_ | 0.0 |
| Ng 14-11 1 µM_x000d_ | 0.0 |
| Ng 14-12 1 µM_x000d_ | 112.33333333333331 |
| Ng 14-7 1 µM_x000d_ | 0.0 |
| Ng 15-14/15 1 µM_x000d_ | 0.0 |
| Ng 15-12 1µM_x000d_ | 78.66666666666666 |
| Ng 17-7 1 µM_x000d_ | 0.0 |
| Ng 17-7 3 µM_x000d_ | 0.0 |
| Ng 17-7 10 µM_x000d_ | 0.0 |
| Ng 19-9 1 µM_x000d_ | 0.0 |
| Ng 19-9 3 µM_x000d_ | 0.0 |
| Ng 19-9 10 µM_x000d_ | 0.0 |
| Ng 20-7 1 µM_x000d_ | 0.0 |
| Ng 20-7 3 µM_x000d_ | 0.0 |
| Ng 20-7 10 µM_x000d_ | 0.0 |
| Ng 21-8 1 µM_x000d_ | 0.0 |
| Ng 21-8 3 µM_x000d_ | 0.0 |
| Ng 21-8 10 µM_x000d_ | 0.0 |
| Ng 15-13 3µM_x000d_ | 1347.0 |
| Ng 16-12 1 µM_x000d_ | 0.0 |
| Ng 17-9 1 µM_x000d_ | 0.0 |
| Ng 17-9 3 µM_x000d_ | 0.0 |
| Ng 18-11 1 µM_x000d_ | 0.0 |
| Ng 18-11 3 µM_x000d_ | 0.0 |
| Ng 20-8 1 µM_x000d_ | 0.0 |
| Ng 20-9 1 µM_x000d_ | 0.0 |
| Ng 20-9 3 µM_x000d_ | 0.0 |
| Ng 21-9 1 µM_x000d_ | 0.0 |
| Ng 13-13 3 µM_x000d_ | 0.0 |
| Ng 14-8 1 µM_x000d_ | 0.0 |
| Ng 14-9 1 µM_x000d_ | 0.0 |
| Ng 14-9 3 µM_x000d_ | 0.0 |
| Ng 14-9 10 µM_x000d_ | 0.0 |
| Ng 14-9 30 µM_x000d_ | 0.0 |
| Ng 15-10 1µM_x000d_ | 0.0 |
| Ng 16-9 1 µM_x000d_ | 0.0 |
| Ng 17-8 1 µM_x000d_ | 0.0 |
| Ng 17-8 3 µM_x000d_ | 0.0 |
| Ng 18-9 1µM_x000d_ | 1345.3333333333333 |
| Ng 19-11 1 µM_x000d_ | 0.0 |
| Ng 19-11 3 µM_x000d_ | 0.0 |
| Lg 17-15 HF 10 µM_x000d_ | 293.0 |
| Lg 15-17 1 µM_x000d_ | 0.0 |
| Lg 17-15 1 µM_x000d_ | 0.0 |
| Lg 17-15 3 µM_x000d_ | 2.0 |
| Lg 17-15 10 µM_x000d_ | 144.0 |
| Lg 17-17 1 µM_x000d_ | 0.0 |
| Lg 17-16 1 µM_x000d_ | 0.0 |
| Lg 16-17 3 µM_x000d_ | 0.0 |
| Lg 18-8 1 µM_x000d_ | 0.0 |
| Lg 19-8 1 µM_x000d_ | 0.0 |
| Lg 19-8 3 µM_x000d_ | 0.0 |
| Lg 19-9 1 µM_x000d_ | 0.0 |
| Lg 20-10_x000d_ | 0.0 |
| Lg 22-12 1 µM_x000d_ | 0.0 |
| Lg 20-9 1 µM_x000d_ | 0.0 |
| Lg 23-11 1 µM_x000d_ | 0.0 |
| Lg 23-11 3 µM_x000d_ | 0.0 |
| Lg 23-12_x000d_ | 0.0 |
| Lg 26-8_x000d_ | 0.0 |
| Lg 26-9_x000d_ | 0.0 |
| Lg 22-13_x000d_ | 0.0 |
| Lg 26-10_x000d_ | 0.0 |
| Lg 18-7-F3 HF 1uM | 0.0 |
| Lg 19-8-F5 HF 3uM | 0.0 |
| Lg 23-11-F2 HF 3uM | 0.0 |
| Blank 1 | 0.0 |
| Blank 2 | 0.0 |
| Blank 3 | 0.0 |
| Blank 4 | 0.0 |
| Blank 5 | 0.0 |MFI
### Chart: Un-NL1
| Category | NLD-57 IgG3 |
|---|---|
| Ng 7-13 1µM_x000d_ | 0.0 |
| Ng 7-13 3µM_x000d_ | 27.0 |
| Ng 9-9 1 µM_x000d_ | 0.0 |
| Ng 9-9 3µM_x000d_ | 0.0 |
| Ng 9-9 10µM_x000d_ | 0.0 |
| Ng 10-13 1 µM_x000d_ | 0.0 |
| Ng 10-13 3 µM_x000d_ | 0.0 |
| Ng 10-13 10 µM_x000d_ | 0.0 |
| Ng 10-13 30 µM_x000d_ | 0.0 |
| Ng 10-10 1 µM_x000d_ | 0.0 |
| Ng 10-10 3 µM_x000d_ | 3.666666666666657 |
| Ng 10-11 1 µM_x000d_ | 0.0 |
| Ng 10-11 3 µM_x000d_ | 0.0 |
| Ng 10-12 1 µM_x000d_ | 0.0 |
| Ng 11-10 1 µM_x000d_ | 0.0 |
| Ng 11-10 3 µM_x000d_ | 0.0 |
| Ng 11-11 1 µM_x000d_ | 0.0 |
| Ng 11-11 3µM_x000d_ | 0.3333333333333428 |
| Ng 11-11 10µM_x000d_ | 0.0 |
| Ng 12-11 1 µM_x000d_ | 0.0 |
| Ng 12-11 3 µM_x000d_ | 0.0 |
| Ng 13-15 1 µM_x000d_ | 0.0 |
| Ng 13-15 3 µM_x000d_ | 0.0 |
| Ng 13-14_x000d_ | 1563.6666666666667 |
| Ng 14-10 1 µM_x000d_ | 2051.0 |
| Ng 14-10 3 µM_x000d_ | 4688.666666666667 |
| Ng 14-11 1 µM_x000d_ | 757.3333333333334 |
| Ng 14-12 1 µM_x000d_ | 9379.666666666666 |
| Ng 14-7 1 µM_x000d_ | 0.0 |
| Ng 15-14/15 1 µM_x000d_ | 3522.6666666666665 |
| Ng 15-12 1µM_x000d_ | 107.0 |
| Ng 17-7 1 µM_x000d_ | 0.0 |
| Ng 17-7 3 µM_x000d_ | 0.0 |
| Ng 17-7 10 µM_x000d_ | 0.0 |
| Ng 19-9 1 µM_x000d_ | 0.0 |
| Ng 19-9 3 µM_x000d_ | 192.33333333333331 |
| Ng 19-9 10 µM_x000d_ | 515.0 |
| Ng 20-7 1 µM_x000d_ | 0.0 |
| Ng 20-7 3 µM_x000d_ | 0.0 |
| Ng 20-7 10 µM_x000d_ | 46.0 |
| Ng 21-8 1 µM_x000d_ | 0.0 |
| Ng 21-8 3 µM_x000d_ | 42.0 |
| Ng 21-8 10 µM_x000d_ | 215.0 |
| Ng 15-13 3µM_x000d_ | 1710.0 |
| Ng 16-12 1 µM_x000d_ | 0.0 |
| Ng 17-9 1 µM_x000d_ | 86.33333333333334 |
| Ng 17-9 3 µM_x000d_ | 856.0 |
| Ng 18-11 1 µM_x000d_ | 417.0 |
| Ng 18-11 3 µM_x000d_ | 1163.3333333333333 |
| Ng 20-8 1 µM_x000d_ | 626.6666666666666 |
| Ng 20-9 1 µM_x000d_ | 1641.6666666666667 |
| Ng 20-9 3 µM_x000d_ | 5442.0 |
| Ng 21-9 1 µM_x000d_ | 2807.6666666666665 |
| Ng 13-13 3 µM_x000d_ | 0.0 |
| Ng 14-8 1 µM_x000d_ | 0.0 |
| Ng 14-9 1 µM_x000d_ | 0.0 |
| Ng 14-9 3 µM_x000d_ | 0.0 |
| Ng 14-9 10 µM_x000d_ | 32.0 |
| Ng 14-9 30 µM_x000d_ | 27.666666666666657 |
| Ng 15-10 1µM_x000d_ | 0.0 |
| Ng 16-9 1 µM_x000d_ | 0.0 |
| Ng 17-8 1 µM_x000d_ | 0.0 |
| Ng 17-8 3 µM_x000d_ | 0.0 |
| Ng 18-9 1µM_x000d_ | 835.6666666666666 |
| Ng 19-11 1 µM_x000d_ | 2541.0 |
| Ng 19-11 3 µM_x000d_ | 5436.666666666667 |
| Lg 17-15 HF 10 µM_x000d_ | 331.0 |
| Lg 15-17 1 µM_x000d_ | 78.33333333333334 |
| Lg 17-15 1 µM_x000d_ | 4710.0 |
| Lg 17-15 3 µM_x000d_ | 10777.0 |
| Lg 17-15 10 µM_x000d_ | 15495.0 |
| Lg 17-17 1 µM_x000d_ | 5199.666666666667 |
| Lg 17-16 1 µM_x000d_ | 7299.333333333333 |
| Lg 16-17 3 µM_x000d_ | 7129.666666666667 |
| Lg 18-8 1 µM_x000d_ | 5905.666666666667 |
| Lg 19-8 1 µM_x000d_ | 0.0 |
| Lg 19-8 3 µM_x000d_ | 183.33333333333331 |
| Lg 19-9 1 µM_x000d_ | 5233.666666666667 |
| Lg 20-10_x000d_ | 44.33333333333334 |
| Lg 22-12 1 µM_x000d_ | 0.0 |
| Lg 20-9 1 µM_x000d_ | 195.66666666666669 |
| Lg 23-11 1 µM_x000d_ | 0.0 |
| Lg 23-11 3 µM_x000d_ | 127.33333333333331 |
| Lg 23-12_x000d_ | 1475.6666666666667 |
| Lg 26-8_x000d_ | 0.0 |
| Lg 26-9_x000d_ | 402.66666666666663 |
| Lg 22-13_x000d_ | 1967.0 |
| Lg 26-10_x000d_ | 7.666666666666657 |
| Lg 18-7-F3 HF 1uM | 0.0 |
| Lg 19-8-F5 HF 3uM | 0.0 |
| Lg 23-11-F2 HF 3uM | 0.0 |
| Blank 1 | 0.0 |
| Blank 2 | 0.0 |
| Blank 3 | 0.0 |
| Blank 4 | 0.0 |
| Blank 5 | 0.0 |MFI
### Chart: Un-NL2
| Category | NLD-59 IgG3 |
|---|---|
| Ng 7-13 1µM_x000d_ | 0.0 |
| Ng 7-13 3µM_x000d_ | 0.0 |
| Ng 9-9 1 µM_x000d_ | 0.0 |
| Ng 9-9 3µM_x000d_ | 0.0 |
| Ng 9-9 10µM_x000d_ | 0.0 |
| Ng 10-13 1 µM_x000d_ | 0.0 |
| Ng 10-13 3 µM_x000d_ | 0.0 |
| Ng 10-13 10 µM_x000d_ | 0.0 |
| Ng 10-13 30 µM_x000d_ | 0.0 |
| Ng 10-10 1 µM_x000d_ | 0.0 |
| Ng 10-10 3 µM_x000d_ | 0.0 |
| Ng 10-11 1 µM_x000d_ | 0.0 |
| Ng 10-11 3 µM_x000d_ | 0.0 |
| Ng 10-12 1 µM_x000d_ | 0.0 |
| Ng 11-10 1 µM_x000d_ | 0.0 |
| Ng 11-10 3 µM_x000d_ | 0.0 |
| Ng 11-11 1 µM_x000d_ | 0.0 |
| Ng 11-11 3µM_x000d_ | 0.0 |
| Ng 11-11 10µM_x000d_ | 0.0 |
| Ng 12-11 1 µM_x000d_ | 0.0 |
| Ng 12-11 3 µM_x000d_ | 0.0 |
| Ng 13-15 1 µM_x000d_ | 0.0 |
| Ng 13-15 3 µM_x000d_ | 0.0 |
| Ng 13-14_x000d_ | 91.66666666666666 |
| Ng 14-10 1 µM_x000d_ | 768.3333333333334 |
| Ng 14-10 3 µM_x000d_ | 265.6666666666667 |
| Ng 14-11 1 µM_x000d_ | 39.66666666666666 |
| Ng 14-12 1 µM_x000d_ | 2422.3333333333335 |
| Ng 14-7 1 µM_x000d_ | 0.0 |
| Ng 15-14/15 1 µM_x000d_ | 809.3333333333334 |
| Ng 15-12 1µM_x000d_ | 89.33333333333334 |
| Ng 17-7 1 µM_x000d_ | 0.0 |
| Ng 17-7 3 µM_x000d_ | 0.0 |
| Ng 17-7 10 µM_x000d_ | 0.0 |
| Ng 19-9 1 µM_x000d_ | 0.0 |
| Ng 19-9 3 µM_x000d_ | 0.0 |
| Ng 19-9 10 µM_x000d_ | 0.0 |
| Ng 20-7 1 µM_x000d_ | 0.0 |
| Ng 20-7 3 µM_x000d_ | 0.0 |
| Ng 20-7 10 µM_x000d_ | 0.0 |
| Ng 21-8 1 µM_x000d_ | 0.0 |
| Ng 21-8 3 µM_x000d_ | 0.0 |
| Ng 21-8 10 µM_x000d_ | 0.0 |
| Ng 15-13 3µM_x000d_ | 1388.6666666666667 |
| Ng 16-12 1 µM_x000d_ | 0.0 |
| Ng 17-9 1 µM_x000d_ | 0.0 |
| Ng 17-9 3 µM_x000d_ | 0.0 |
| Ng 18-11 1 µM_x000d_ | 0.0 |
| Ng 18-11 3 µM_x000d_ | 0.0 |
| Ng 20-8 1 µM_x000d_ | 0.0 |
| Ng 20-9 1 µM_x000d_ | 0.0 |
| Ng 20-9 3 µM_x000d_ | 41.66666666666666 |
| Ng 21-9 1 µM_x000d_ | 282.3333333333333 |
| Ng 13-13 3 µM_x000d_ | 0.0 |
| Ng 14-8 1 µM_x000d_ | 0.0 |
| Ng 14-9 1 µM_x000d_ | 0.0 |
| Ng 14-9 3 µM_x000d_ | 0.0 |
| Ng 14-9 10 µM_x000d_ | 0.0 |
| Ng 14-9 30 µM_x000d_ | 0.0 |
| Ng 15-10 1µM_x000d_ | 0.0 |
| Ng 16-9 1 µM_x000d_ | 0.0 |
| Ng 17-8 1 µM_x000d_ | 0.0 |
| Ng 17-8 3 µM_x000d_ | 0.0 |
| Ng 18-9 1µM_x000d_ | 1072.0 |
| Ng 19-11 1 µM_x000d_ | 24.666666666666657 |
| Ng 19-11 3 µM_x000d_ | 455.0 |
| Lg 17-15 HF 10 µM_x000d_ | 0.0 |
| Lg 15-17 1 µM_x000d_ | 0.0 |
| Lg 17-15 1 µM_x000d_ | 0.0 |
| Lg 17-15 3 µM_x000d_ | 0.0 |
| Lg 17-15 10 µM_x000d_ | 0.0 |
| Lg 17-17 1 µM_x000d_ | 0.0 |
| Lg 17-16 1 µM_x000d_ | 0.0 |
| Lg 16-17 3 µM_x000d_ | 0.0 |
| Lg 18-8 1 µM_x000d_ | 0.0 |
| Lg 19-8 1 µM_x000d_ | 0.0 |
| Lg 19-8 3 µM_x000d_ | 0.0 |
| Lg 19-9 1 µM_x000d_ | 0.0 |
| Lg 20-10_x000d_ | 0.0 |
| Lg 22-12 1 µM_x000d_ | 0.0 |
| Lg 20-9 1 µM_x000d_ | 0.0 |
| Lg 23-11 1 µM_x000d_ | 0.0 |
| Lg 23-11 3 µM_x000d_ | 0.0 |
| Lg 23-12_x000d_ | 0.0 |
| Lg 26-8_x000d_ | 0.0 |
| Lg 26-9_x000d_ | 0.0 |
| Lg 22-13_x000d_ | 0.0 |
| Lg 26-10_x000d_ | 0.0 |
| Lg 18-7-F3 HF 1uM | 0.0 |
| Lg 19-8-F5 HF 3uM | 0.0 |
| Lg 23-11-F2 HF 3uM | 0.0 |
| Blank 1 | 0.0 |
| Blank 2 | 0.0 |
| Blank 3 | 0.0 |
| Blank 4 | 0.0 |
| Blank 5 | 0.0 |MFI
### Chart: Un-NL3
| Category | NLD-60 IgG3 |
|---|---|
| Ng 7-13 1µM_x000d_ | 0.0 |
| Ng 7-13 3µM_x000d_ | 0.0 |
| Ng 9-9 1 µM_x000d_ | 0.0 |
| Ng 9-9 3µM_x000d_ | 0.0 |
| Ng 9-9 10µM_x000d_ | 0.0 |
| Ng 10-13 1 µM_x000d_ | 0.0 |
| Ng 10-13 3 µM_x000d_ | 0.0 |
| Ng 10-13 10 µM_x000d_ | 0.0 |
| Ng 10-13 30 µM_x000d_ | 0.0 |
| Ng 10-10 1 µM_x000d_ | 0.0 |
| Ng 10-10 3 µM_x000d_ | 0.0 |
| Ng 10-11 1 µM_x000d_ | 0.0 |
| Ng 10-11 3 µM_x000d_ | 0.0 |
| Ng 10-12 1 µM_x000d_ | 0.0 |
| Ng 11-10 1 µM_x000d_ | 0.0 |
| Ng 11-10 3 µM_x000d_ | 0.0 |
| Ng 11-11 1 µM_x000d_ | 0.0 |
| Ng 11-11 3µM_x000d_ | 0.0 |
| Ng 11-11 10µM_x000d_ | 0.0 |
| Ng 12-11 1 µM_x000d_ | 0.0 |
| Ng 12-11 3 µM_x000d_ | 0.0 |
| Ng 13-15 1 µM_x000d_ | 0.0 |
| Ng 13-15 3 µM_x000d_ | 0.0 |
| Ng 13-14_x000d_ | 0.0 |
| Ng 14-10 1 µM_x000d_ | 0.0 |
| Ng 14-10 3 µM_x000d_ | 0.0 |
| Ng 14-11 1 µM_x000d_ | 0.0 |
| Ng 14-12 1 µM_x000d_ | 0.0 |
| Ng 14-7 1 µM_x000d_ | 0.0 |
| Ng 15-14/15 1 µM_x000d_ | 27.333333333333343 |
| Ng 15-12 1µM_x000d_ | 139.66666666666669 |
| Ng 17-7 1 µM_x000d_ | 0.0 |
| Ng 17-7 3 µM_x000d_ | 0.0 |
| Ng 17-7 10 µM_x000d_ | 0.0 |
| Ng 19-9 1 µM_x000d_ | 0.0 |
| Ng 19-9 3 µM_x000d_ | 0.0 |
| Ng 19-9 10 µM_x000d_ | 0.0 |
| Ng 20-7 1 µM_x000d_ | 19.0 |
| Ng 20-7 3 µM_x000d_ | 0.0 |
| Ng 20-7 10 µM_x000d_ | 0.0 |
| Ng 21-8 1 µM_x000d_ | 0.0 |
| Ng 21-8 3 µM_x000d_ | 0.0 |
| Ng 21-8 10 µM_x000d_ | 0.0 |
| Ng 15-13 3µM_x000d_ | 891.3333333333333 |
| Ng 16-12 1 µM_x000d_ | 0.0 |
| Ng 17-9 1 µM_x000d_ | 0.0 |
| Ng 17-9 3 µM_x000d_ | 0.0 |
| Ng 18-11 1 µM_x000d_ | 0.0 |
| Ng 18-11 3 µM_x000d_ | 0.0 |
| Ng 20-8 1 µM_x000d_ | 0.0 |
| Ng 20-9 1 µM_x000d_ | 0.0 |
| Ng 20-9 3 µM_x000d_ | 0.0 |
| Ng 21-9 1 µM_x000d_ | 0.0 |
| Ng 13-13 3 µM_x000d_ | 0.0 |
| Ng 14-8 1 µM_x000d_ | 0.0 |
| Ng 14-9 1 µM_x000d_ | 0.0 |
| Ng 14-9 3 µM_x000d_ | 0.0 |
| Ng 14-9 10 µM_x000d_ | 0.0 |
| Ng 14-9 30 µM_x000d_ | 0.0 |
| Ng 15-10 1µM_x000d_ | 0.0 |
| Ng 16-9 1 µM_x000d_ | 382.0 |
| Ng 17-8 1 µM_x000d_ | 0.0 |
| Ng 17-8 3 µM_x000d_ | 0.0 |
| Ng 18-9 1µM_x000d_ | 948.3333333333333 |
| Ng 19-11 1 µM_x000d_ | 0.0 |
| Ng 19-11 3 µM_x000d_ | 0.0 |
| Lg 17-15 HF 10 µM_x000d_ | 64730.333333333336 |
| Lg 15-17 1 µM_x000d_ | 4011.0 |
| Lg 17-15 1 µM_x000d_ | 2213.6666666666665 |
| Lg 17-15 3 µM_x000d_ | 11518.333333333334 |
| Lg 17-15 10 µM_x000d_ | 26260.666666666668 |
| Lg 17-17 1 µM_x000d_ | 1064.3333333333333 |
| Lg 17-16 1 µM_x000d_ | 4527.333333333333 |
| Lg 16-17 3 µM_x000d_ | 27817.666666666668 |
| Lg 18-8 1 µM_x000d_ | 1491.0 |
| Lg 19-8 1 µM_x000d_ | 0.0 |
| Lg 19-8 3 µM_x000d_ | 7.666666666666657 |
| Lg 19-9 1 µM_x000d_ | 761.0 |
| Lg 20-10_x000d_ | 0.0 |
| Lg 22-12 1 µM_x000d_ | 0.0 |
| Lg 20-9 1 µM_x000d_ | 0.0 |
| Lg 23-11 1 µM_x000d_ | 0.0 |
| Lg 23-11 3 µM_x000d_ | 31.333333333333343 |
| Lg 23-12_x000d_ | 0.0 |
| Lg 26-8_x000d_ | 0.0 |
| Lg 26-9_x000d_ | 0.0 |
| Lg 22-13_x000d_ | 0.0 |
| Lg 26-10_x000d_ | 0.0 |
| Lg 18-7-F3 HF 1uM | 0.0 |
| Lg 19-8-F5 HF 3uM | 291.6666666666667 |
| Lg 23-11-F2 HF 3uM | 0.0 |
| Blank 1 | 0.0 |
| Blank 2 | 0.0 |
| Blank 3 | 0.0 |
| Blank 4 | 0.0 |
| Blank 5 | 0.0 |MFI
### Chart: Un-NL4
| Category | NLD-61 IgG3 |
|---|---|
| Ng 7-13 1µM_x000d_ | 0.0 |
| Ng 7-13 3µM_x000d_ | 0.0 |
| Ng 9-9 1 µM_x000d_ | 0.0 |
| Ng 9-9 3µM_x000d_ | 0.0 |
| Ng 9-9 10µM_x000d_ | 0.0 |
| Ng 10-13 1 µM_x000d_ | 0.0 |
| Ng 10-13 3 µM_x000d_ | 0.0 |
| Ng 10-13 10 µM_x000d_ | 0.0 |
| Ng 10-13 30 µM_x000d_ | 0.0 |
| Ng 10-10 1 µM_x000d_ | 0.0 |
| Ng 10-10 3 µM_x000d_ | 0.0 |
| Ng 10-11 1 µM_x000d_ | 0.0 |
| Ng 10-11 3 µM_x000d_ | 0.0 |
| Ng 10-12 1 µM_x000d_ | 0.0 |
| Ng 11-10 1 µM_x000d_ | 0.0 |
| Ng 11-10 3 µM_x000d_ | 0.0 |
| Ng 11-11 1 µM_x000d_ | 0.0 |
| Ng 11-11 3µM_x000d_ | 0.0 |
| Ng 11-11 10µM_x000d_ | 0.0 |
| Ng 12-11 1 µM_x000d_ | 0.0 |
| Ng 12-11 3 µM_x000d_ | 0.0 |
| Ng 13-15 1 µM_x000d_ | 0.0 |
| Ng 13-15 3 µM_x000d_ | 0.0 |
| Ng 13-14_x000d_ | 0.0 |
| Ng 14-10 1 µM_x000d_ | 0.0 |
| Ng 14-10 3 µM_x000d_ | 0.0 |
| Ng 14-11 1 µM_x000d_ | 0.0 |
| Ng 14-12 1 µM_x000d_ | 0.0 |
| Ng 14-7 1 µM_x000d_ | 0.0 |
| Ng 15-14/15 1 µM_x000d_ | 0.0 |
| Ng 15-12 1µM_x000d_ | 40.0 |
| Ng 17-7 1 µM_x000d_ | 0.0 |
| Ng 17-7 3 µM_x000d_ | 0.0 |
| Ng 17-7 10 µM_x000d_ | 0.0 |
| Ng 19-9 1 µM_x000d_ | 0.0 |
| Ng 19-9 3 µM_x000d_ | 0.0 |
| Ng 19-9 10 µM_x000d_ | 0.0 |
| Ng 20-7 1 µM_x000d_ | 0.0 |
| Ng 20-7 3 µM_x000d_ | 0.0 |
| Ng 20-7 10 µM_x000d_ | 0.0 |
| Ng 21-8 1 µM_x000d_ | 0.0 |
| Ng 21-8 3 µM_x000d_ | 0.0 |
| Ng 21-8 10 µM_x000d_ | 0.0 |
| Ng 15-13 3µM_x000d_ | 1040.6666666666667 |
| Ng 16-12 1 µM_x000d_ | 0.0 |
| Ng 17-9 1 µM_x000d_ | 0.0 |
| Ng 17-9 3 µM_x000d_ | 0.0 |
| Ng 18-11 1 µM_x000d_ | 0.0 |
| Ng 18-11 3 µM_x000d_ | 0.0 |
| Ng 20-8 1 µM_x000d_ | 0.0 |
| Ng 20-9 1 µM_x000d_ | 0.0 |
| Ng 20-9 3 µM_x000d_ | 0.0 |
| Ng 21-9 1 µM_x000d_ | 0.0 |
| Ng 13-13 3 µM_x000d_ | 0.0 |
| Ng 14-8 1 µM_x000d_ | 0.0 |
| Ng 14-9 1 µM_x000d_ | 0.0 |
| Ng 14-9 3 µM_x000d_ | 0.0 |
| Ng 14-9 10 µM_x000d_ | 0.0 |
| Ng 14-9 30 µM_x000d_ | 0.0 |
| Ng 15-10 1µM_x000d_ | 0.0 |
| Ng 16-9 1 µM_x000d_ | 0.0 |
| Ng 17-8 1 µM_x000d_ | 0.0 |
| Ng 17-8 3 µM_x000d_ | 0.0 |
| Ng 18-9 1µM_x000d_ | 947.0 |
| Ng 19-11 1 µM_x000d_ | 0.0 |
| Ng 19-11 3 µM_x000d_ | 0.0 |
| Lg 17-15 HF 10 µM_x000d_ | 0.0 |
| Lg 15-17 1 µM_x000d_ | 0.0 |
| Lg 17-15 1 µM_x000d_ | 0.0 |
| Lg 17-15 3 µM_x000d_ | 0.0 |
| Lg 17-15 10 µM_x000d_ | 0.0 |
| Lg 17-17 1 µM_x000d_ | 0.0 |
| Lg 17-16 1 µM_x000d_ | 0.0 |
| Lg 16-17 3 µM_x000d_ | 0.0 |
| Lg 18-8 1 µM_x000d_ | 0.0 |
| Lg 19-8 1 µM_x000d_ | 0.0 |
| Lg 19-8 3 µM_x000d_ | 0.0 |
| Lg 19-9 1 µM_x000d_ | 0.0 |
| Lg 20-10_x000d_ | 0.0 |
| Lg 22-12 1 µM_x000d_ | 0.0 |
| Lg 20-9 1 µM_x000d_ | 0.0 |
| Lg 23-11 1 µM_x000d_ | 0.0 |
| Lg 23-11 3 µM_x000d_ | 0.0 |
| Lg 23-12_x000d_ | 0.0 |
| Lg 26-8_x000d_ | 0.0 |
| Lg 26-9_x000d_ | 0.0 |
| Lg 22-13_x000d_ | 0.0 |
| Lg 26-10_x000d_ | 0.0 |
| Lg 18-7-F3 HF 1uM | 0.0 |
| Lg 19-8-F5 HF 3uM | 0.0 |
| Lg 23-11-F2 HF 3uM | 0.0 |
| Blank 1 | 0.0 |
| Blank 2 | 0.0 |
| Blank 3 | 0.0 |
| Blank 4 | 0.0 |
| Blank 5 | 0.0 |MFI
### Chart: Un-NL5
| Category | NLD-62 IgG3 |
|---|---|
| Ng1_1uM
 | 0.0 |
| Ng1_3uM
 | 0.0 |
| Ng2_1uM | 0.0 |
| Ng2_3uM | 0.0 |
| Ng2_10uM | 0.0 |
| Ng3_1uM | 0.0 |
| Ng3_3uM | 0.0 |
| Ng3_10uM | 0.0 |
| Ng3_30uM | 0.0 |
| Ng4_1uM | 0.0 |
| Ng4_3uM | 0.0 |
| Ng5_1uM | 0.0 |
| Ng5_3uM | 0.0 |
| Ng6_1uM | 0.0 |
| Ng7_1uM | 0.0 |
| Ng7_3uM | 0.0 |
| Ng8_1uM | 0.0 |
| Ng8_3uM | 0.0 |
| Ng8_10uM | 0.0 |
| Ng9_1uM | 0.0 |
| Ng9_3uM | 0.0 |
| Ng10_3uM | 0.0 |
| Ng10_1uM | 0.0 |
| Ng11 | 0.0 |
| Ng12_1uM | 95.66666666666666 |
| Ng12_3uM | 725.0 |
| Ng13_1uM | 0.0 |
| Ng14_1uM | 1273.6666666666667 |
| Ng15_1uM | 0.0 |
| Ng16_1uM | 211.33333333333331 |
| Ng17_1uM | 130.33333333333331 |
| Ng18_1uM | 0.0 |
| Ng18_3uM | 0.0 |
| Ng18_10uM | 0.0 |
| Ng19_1uM | 0.0 |
| Ng19_3uM | 0.0 |
| Ng19_10uM | 0.0 |
| Ng20_1uM | 0.0 |
| Ng20_3uM | 0.0 |
| Ng20_10uM | 0.0 |
| Ng21_1uM | 0.0 |
| Ng21 _3uM | 0.0 |
| Ng21_10uM | 0.0 |
| Ng22_1uM | 1304.3333333333333 |
| Ng23_1uM | 0.0 |
| Ng24_1uM | 0.0 |
| Ng24_3uM | 0.0 |
| Ng25_1uM | 0.0 |
| Ng25_3uM | 0.0 |
| Ng26_1uM | 0.0 |
| Ng27_1uM | 0.0 |
| Ng27_3uM | 0.0 |
| Ng28_1uM | 0.0 |
| Ng29_1uM | 0.0 |
| Ng30_1uM | 0.0 |
| Ng31_1uM | 0.0 |
| Ng31_3uM | 0.0 |
| Ng31_10uM | 0.0 |
| Ng31_30uM | 0.0 |
| Ng32_1uM | 0.0 |
| Ng33_1uM | 0.0 |
| Ng34_1uM | 0.0 |
| Ng34_3uM | 0.0 |
| Ng35_1uM | 1086.0 |
| Ng36_1uM | 0.0 |
| Ng36_3uM | 0.0 |
| Lg2_HF_10uM | 0.0 |
| Lg1_1uM | 0.0 |
| Lg2_1uM | 0.0 |
| Lg2_3uM | 0.0 |
| Lg2_10uM | 0.0 |
| Lg3_1uM | 0.0 |
| Lg4_1uM | 0.0 |
| Lg5_3uM | 63.66666666666666 |
| Lg6_1uM | 0.0 |
| Lg7_1uM | 0.0 |
| Lg7_3uM | 0.0 |
| Lg8_1uM | 0.0 |
| Lg9 | 0.0 |
| Lg10_1uM | 0.0 |
| Lg11_1uM | 0.0 |
| Lg12_1uM | 0.0 |
| Lg12_3uM | 0.0 |
| Lg13 | 0.0 |
| Lg14 | 0.0 |
| Lg15 | 0.0 |
| Lg16 | 0.0 |
| Lg17 | 0.0 |
| Lg18_HF_1uM | 0.0 |
| Lg7_HF_3uM | 0.0 |
| Lg12_HF_3uM | 0.0 |
| Blank 1 | 0.0 |
| Blank 2 | 0.0 |
| Blank 3 | 0.0 |
| Blank 4 | 0.0 |
| Blank 5 | 0.0 |MFI

## Slide 37
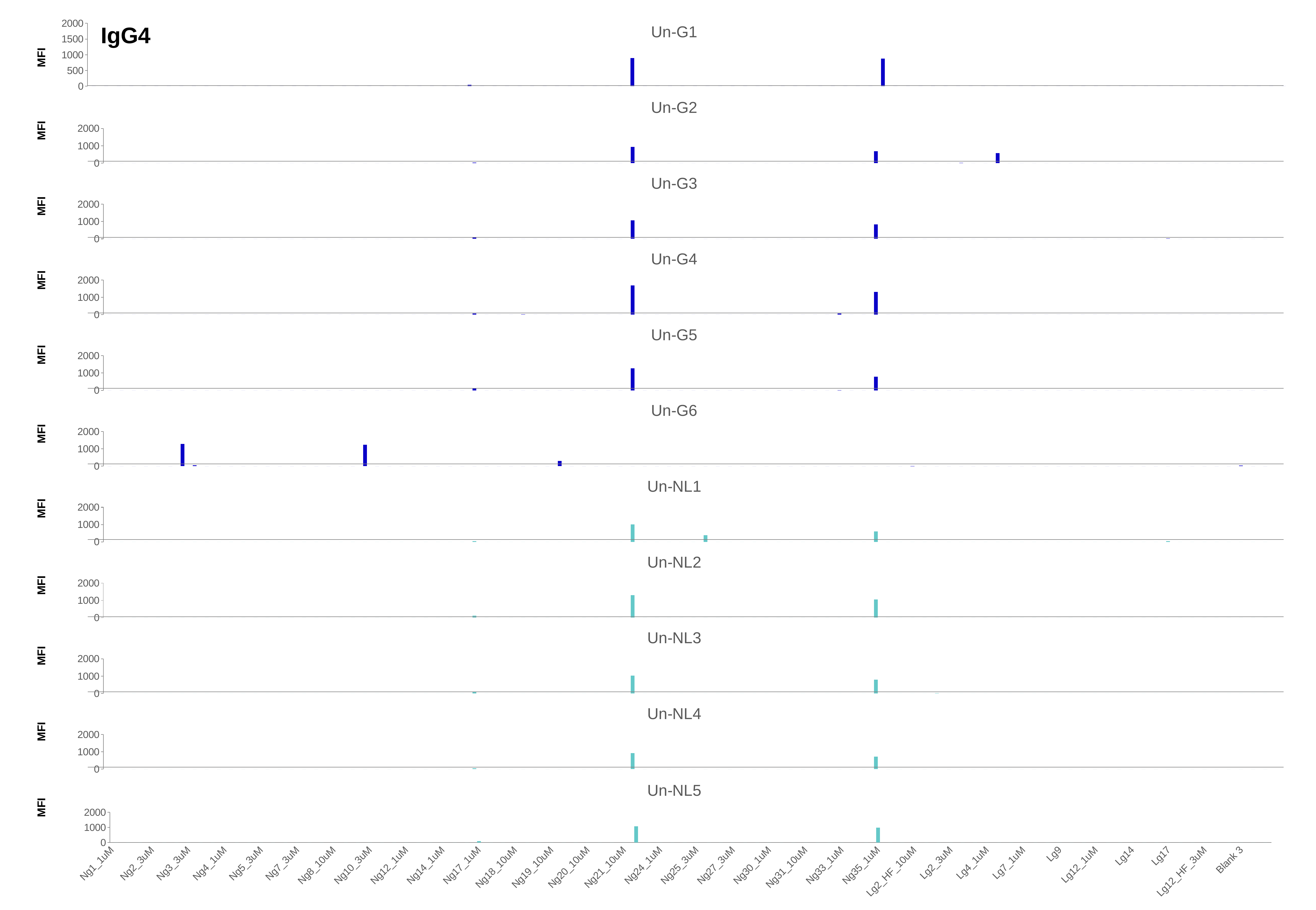

### Chart: Un-G1
| Category | UG 000-214 IgG4 |
|---|---|
| Ng 7-13 1µM_x000d_ | 0.0 |
| Ng 7-13 3µM_x000d_ | 0.0 |
| Ng 9-9 1 µM_x000d_ | 0.0 |
| Ng 9-9 3µM_x000d_ | 0.0 |
| Ng 9-9 10µM_x000d_ | 0.0 |
| Ng 10-13 1 µM_x000d_ | 0.0 |
| Ng 10-13 3 µM_x000d_ | 0.0 |
| Ng 10-13 10 µM_x000d_ | 0.0 |
| Ng 10-13 30 µM_x000d_ | 0.0 |
| Ng 10-10 1 µM_x000d_ | 0.0 |
| Ng 10-10 3 µM_x000d_ | 0.0 |
| Ng 10-11 1 µM_x000d_ | 0.0 |
| Ng 10-11 3 µM_x000d_ | 0.0 |
| Ng 10-12 1 µM_x000d_ | 0.0 |
| Ng 11-10 1 µM_x000d_ | 0.0 |
| Ng 11-10 3 µM_x000d_ | 0.0 |
| Ng 11-11 1 µM_x000d_ | 0.0 |
| Ng 11-11 3µM_x000d_ | 0.0 |
| Ng 11-11 10µM_x000d_ | 0.0 |
| Ng 12-11 1 µM_x000d_ | 0.0 |
| Ng 12-11 3 µM_x000d_ | 0.0 |
| Ng 13-15 1 µM_x000d_ | 0.0 |
| Ng 13-15 3 µM_x000d_ | 0.0 |
| Ng 13-14_x000d_ | 0.0 |
| Ng 14-10 1 µM_x000d_ | 0.0 |
| Ng 14-10 3 µM_x000d_ | 0.0 |
| Ng 14-11 1 µM_x000d_ | 0.0 |
| Ng 14-12 1 µM_x000d_ | 0.0 |
| Ng 14-7 1 µM_x000d_ | 0.0 |
| Ng 15-14/15 1 µM_x000d_ | 0.0 |
| Ng 15-12 1µM_x000d_ | 44.0 |
| Ng 17-7 1 µM_x000d_ | 0.0 |
| Ng 17-7 3 µM_x000d_ | 0.0 |
| Ng 17-7 10 µM_x000d_ | 0.0 |
| Ng 19-9 1 µM_x000d_ | 0.0 |
| Ng 19-9 3 µM_x000d_ | 0.0 |
| Ng 19-9 10 µM_x000d_ | 0.0 |
| Ng 20-7 1 µM_x000d_ | 0.0 |
| Ng 20-7 3 µM_x000d_ | 0.0 |
| Ng 20-7 10 µM_x000d_ | 0.0 |
| Ng 21-8 1 µM_x000d_ | 0.0 |
| Ng 21-8 3 µM_x000d_ | 0.0 |
| Ng 21-8 10 µM_x000d_ | 0.0 |
| Ng 15-13 3µM_x000d_ | 893.6666666666667 |
| Ng 16-12 1 µM_x000d_ | 0.0 |
| Ng 17-9 1 µM_x000d_ | 0.0 |
| Ng 17-9 3 µM_x000d_ | 0.0 |
| Ng 18-11 1 µM_x000d_ | 0.0 |
| Ng 18-11 3 µM_x000d_ | 0.0 |
| Ng 20-8 1 µM_x000d_ | 0.0 |
| Ng 20-9 1 µM_x000d_ | 0.0 |
| Ng 20-9 3 µM_x000d_ | 0.0 |
| Ng 21-9 1 µM_x000d_ | 0.0 |
| Ng 13-13 3 µM_x000d_ | 0.0 |
| Ng 14-8 1 µM_x000d_ | 0.0 |
| Ng 14-9 1 µM_x000d_ | 0.0 |
| Ng 14-9 3 µM_x000d_ | 0.0 |
| Ng 14-9 10 µM_x000d_ | 0.0 |
| Ng 14-9 30 µM_x000d_ | 0.0 |
| Ng 15-10 1µM_x000d_ | 0.0 |
| Ng 16-9 1 µM_x000d_ | 0.0 |
| Ng 17-8 1 µM_x000d_ | 0.0 |
| Ng 17-8 3 µM_x000d_ | 0.0 |
| Ng 18-9 1µM_x000d_ | 873.3333333333334 |
| Ng 19-11 1 µM_x000d_ | 0.0 |
| Ng 19-11 3 µM_x000d_ | 0.0 |
| Lg 17-15 HF 10 µM_x000d_ | 0.0 |
| Lg 15-17 1 µM_x000d_ | 0.0 |
| Lg 17-15 1 µM_x000d_ | 0.0 |
| Lg 17-15 3 µM_x000d_ | 0.0 |
| Lg 17-15 10 µM_x000d_ | 0.0 |
| Lg 17-17 1 µM_x000d_ | 0.0 |
| Lg 17-16 1 µM_x000d_ | 0.0 |
| Lg 16-17 3 µM_x000d_ | 0.0 |
| Lg 18-8 1 µM_x000d_ | 0.0 |
| Lg 19-8 1 µM_x000d_ | 0.0 |
| Lg 19-8 3 µM_x000d_ | 0.0 |
| Lg 19-9 1 µM_x000d_ | 0.0 |
| Lg 20-10_x000d_ | 0.0 |
| Lg 22-12 1 µM_x000d_ | 0.0 |
| Lg 20-9 1 µM_x000d_ | 0.0 |
| Lg 23-11 1 µM_x000d_ | 0.0 |
| Lg 23-11 3 µM_x000d_ | 0.0 |
| Lg 23-12_x000d_ | 0.0 |
| Lg 26-8_x000d_ | 0.0 |
| Lg 26-9_x000d_ | 0.0 |
| Lg 22-13_x000d_ | 0.0 |
| Lg 26-10_x000d_ | 0.0 |
| Lg 18-7-F3 HF 1uM | 0.0 |
| Lg 19-8-F5 HF 3uM | 0.0 |
| Lg 23-11-F2 HF 3uM | 0.0 |
| Blank 1 | 0.0 |
| Blank 2 | 0.0 |
| Blank 3 | 0.0 |
| Blank 4 | 0.0 |
| Blank 5 | 0.0 |IgG4
MFI
### Chart: Un-G2
| Category | UG 000-215 IgG4 |
|---|---|
| Ng 7-13 1µM_x000d_ | 0.0 |
| Ng 7-13 3µM_x000d_ | 0.0 |
| Ng 9-9 1 µM_x000d_ | 0.0 |
| Ng 9-9 3µM_x000d_ | 0.0 |
| Ng 9-9 10µM_x000d_ | 0.0 |
| Ng 10-13 1 µM_x000d_ | 0.0 |
| Ng 10-13 3 µM_x000d_ | 0.0 |
| Ng 10-13 10 µM_x000d_ | 0.0 |
| Ng 10-13 30 µM_x000d_ | 0.0 |
| Ng 10-10 1 µM_x000d_ | 0.0 |
| Ng 10-10 3 µM_x000d_ | 0.0 |
| Ng 10-11 1 µM_x000d_ | 0.0 |
| Ng 10-11 3 µM_x000d_ | 0.0 |
| Ng 10-12 1 µM_x000d_ | 0.0 |
| Ng 11-10 1 µM_x000d_ | 0.0 |
| Ng 11-10 3 µM_x000d_ | 0.0 |
| Ng 11-11 1 µM_x000d_ | 0.0 |
| Ng 11-11 3µM_x000d_ | 0.0 |
| Ng 11-11 10µM_x000d_ | 0.0 |
| Ng 12-11 1 µM_x000d_ | 0.0 |
| Ng 12-11 3 µM_x000d_ | 0.0 |
| Ng 13-15 1 µM_x000d_ | 0.0 |
| Ng 13-15 3 µM_x000d_ | 0.0 |
| Ng 13-14_x000d_ | 0.0 |
| Ng 14-10 1 µM_x000d_ | 0.0 |
| Ng 14-10 3 µM_x000d_ | 0.0 |
| Ng 14-11 1 µM_x000d_ | 0.0 |
| Ng 14-12 1 µM_x000d_ | 0.0 |
| Ng 14-7 1 µM_x000d_ | 0.0 |
| Ng 15-14/15 1 µM_x000d_ | 0.0 |
| Ng 15-12 1µM_x000d_ | 30.666666666666657 |
| Ng 17-7 1 µM_x000d_ | 0.0 |
| Ng 17-7 3 µM_x000d_ | 0.0 |
| Ng 17-7 10 µM_x000d_ | 0.0 |
| Ng 19-9 1 µM_x000d_ | 0.0 |
| Ng 19-9 3 µM_x000d_ | 0.0 |
| Ng 19-9 10 µM_x000d_ | 0.0 |
| Ng 20-7 1 µM_x000d_ | 0.0 |
| Ng 20-7 3 µM_x000d_ | 0.0 |
| Ng 20-7 10 µM_x000d_ | 0.0 |
| Ng 21-8 1 µM_x000d_ | 0.0 |
| Ng 21-8 3 µM_x000d_ | 0.0 |
| Ng 21-8 10 µM_x000d_ | 0.0 |
| Ng 15-13 3µM_x000d_ | 938.3333333333333 |
| Ng 16-12 1 µM_x000d_ | 0.0 |
| Ng 17-9 1 µM_x000d_ | 0.0 |
| Ng 17-9 3 µM_x000d_ | 0.0 |
| Ng 18-11 1 µM_x000d_ | 0.0 |
| Ng 18-11 3 µM_x000d_ | 0.0 |
| Ng 20-8 1 µM_x000d_ | 0.0 |
| Ng 20-9 1 µM_x000d_ | 0.0 |
| Ng 20-9 3 µM_x000d_ | 0.0 |
| Ng 21-9 1 µM_x000d_ | 0.0 |
| Ng 13-13 3 µM_x000d_ | 0.0 |
| Ng 14-8 1 µM_x000d_ | 0.0 |
| Ng 14-9 1 µM_x000d_ | 0.0 |
| Ng 14-9 3 µM_x000d_ | 0.0 |
| Ng 14-9 10 µM_x000d_ | 0.0 |
| Ng 14-9 30 µM_x000d_ | 0.0 |
| Ng 15-10 1µM_x000d_ | 0.0 |
| Ng 16-9 1 µM_x000d_ | 0.0 |
| Ng 17-8 1 µM_x000d_ | 0.0 |
| Ng 17-8 3 µM_x000d_ | 0.0 |
| Ng 18-9 1µM_x000d_ | 681.6666666666666 |
| Ng 19-11 1 µM_x000d_ | 0.0 |
| Ng 19-11 3 µM_x000d_ | 0.0 |
| Lg 17-15 HF 10 µM_x000d_ | 0.0 |
| Lg 15-17 1 µM_x000d_ | 0.0 |
| Lg 17-15 1 µM_x000d_ | 0.0 |
| Lg 17-15 3 µM_x000d_ | 0.0 |
| Lg 17-15 10 µM_x000d_ | 17.333333333333343 |
| Lg 17-17 1 µM_x000d_ | 0.0 |
| Lg 17-16 1 µM_x000d_ | 0.0 |
| Lg 16-17 3 µM_x000d_ | 569.6666666666666 |
| Lg 18-8 1 µM_x000d_ | 0.0 |
| Lg 19-8 1 µM_x000d_ | 0.0 |
| Lg 19-8 3 µM_x000d_ | 0.0 |
| Lg 19-9 1 µM_x000d_ | 0.0 |
| Lg 20-10_x000d_ | 0.0 |
| Lg 22-12 1 µM_x000d_ | 0.0 |
| Lg 20-9 1 µM_x000d_ | 0.0 |
| Lg 23-11 1 µM_x000d_ | 0.0 |
| Lg 23-11 3 µM_x000d_ | 0.0 |
| Lg 23-12_x000d_ | 0.0 |
| Lg 26-8_x000d_ | 0.0 |
| Lg 26-9_x000d_ | 0.0 |
| Lg 22-13_x000d_ | 0.0 |
| Lg 26-10_x000d_ | 0.0 |
| Lg 18-7-F3 HF 1uM | 0.0 |
| Lg 19-8-F5 HF 3uM | 0.0 |
| Lg 23-11-F2 HF 3uM | 0.0 |
| Blank 1 | 0.0 |
| Blank 2 | 0.0 |
| Blank 3 | 0.0 |
| Blank 4 | 0.0 |
| Blank 5 | 0.0 |MFI
### Chart: Un-G3
| Category | UG 000-223 IgG4 |
|---|---|
| Ng 7-13 1µM_x000d_ | 0.0 |
| Ng 7-13 3µM_x000d_ | 0.0 |
| Ng 9-9 1 µM_x000d_ | 0.0 |
| Ng 9-9 3µM_x000d_ | 0.0 |
| Ng 9-9 10µM_x000d_ | 0.0 |
| Ng 10-13 1 µM_x000d_ | 0.0 |
| Ng 10-13 3 µM_x000d_ | 0.0 |
| Ng 10-13 10 µM_x000d_ | 0.0 |
| Ng 10-13 30 µM_x000d_ | 0.0 |
| Ng 10-10 1 µM_x000d_ | 0.0 |
| Ng 10-10 3 µM_x000d_ | 0.0 |
| Ng 10-11 1 µM_x000d_ | 0.0 |
| Ng 10-11 3 µM_x000d_ | 0.0 |
| Ng 10-12 1 µM_x000d_ | 0.0 |
| Ng 11-10 1 µM_x000d_ | 0.0 |
| Ng 11-10 3 µM_x000d_ | 0.0 |
| Ng 11-11 1 µM_x000d_ | 0.0 |
| Ng 11-11 3µM_x000d_ | 0.0 |
| Ng 11-11 10µM_x000d_ | 0.0 |
| Ng 12-11 1 µM_x000d_ | 0.0 |
| Ng 12-11 3 µM_x000d_ | 0.0 |
| Ng 13-15 1 µM_x000d_ | 0.0 |
| Ng 13-15 3 µM_x000d_ | 0.0 |
| Ng 13-14_x000d_ | 0.0 |
| Ng 14-10 1 µM_x000d_ | 0.0 |
| Ng 14-10 3 µM_x000d_ | 0.0 |
| Ng 14-11 1 µM_x000d_ | 0.0 |
| Ng 14-12 1 µM_x000d_ | 0.0 |
| Ng 14-7 1 µM_x000d_ | 0.0 |
| Ng 15-14/15 1 µM_x000d_ | 0.0 |
| Ng 15-12 1µM_x000d_ | 62.33333333333334 |
| Ng 17-7 1 µM_x000d_ | 0.0 |
| Ng 17-7 3 µM_x000d_ | 0.0 |
| Ng 17-7 10 µM_x000d_ | 0.0 |
| Ng 19-9 1 µM_x000d_ | 0.0 |
| Ng 19-9 3 µM_x000d_ | 0.0 |
| Ng 19-9 10 µM_x000d_ | 0.0 |
| Ng 20-7 1 µM_x000d_ | 0.0 |
| Ng 20-7 3 µM_x000d_ | 0.0 |
| Ng 20-7 10 µM_x000d_ | 0.0 |
| Ng 21-8 1 µM_x000d_ | 0.0 |
| Ng 21-8 3 µM_x000d_ | 0.0 |
| Ng 21-8 10 µM_x000d_ | 0.0 |
| Ng 15-13 3µM_x000d_ | 1059.0 |
| Ng 16-12 1 µM_x000d_ | 0.0 |
| Ng 17-9 1 µM_x000d_ | 0.0 |
| Ng 17-9 3 µM_x000d_ | 0.0 |
| Ng 18-11 1 µM_x000d_ | 0.0 |
| Ng 18-11 3 µM_x000d_ | 0.0 |
| Ng 20-8 1 µM_x000d_ | 0.0 |
| Ng 20-9 1 µM_x000d_ | 0.0 |
| Ng 20-9 3 µM_x000d_ | 0.0 |
| Ng 21-9 1 µM_x000d_ | 0.0 |
| Ng 13-13 3 µM_x000d_ | 0.0 |
| Ng 14-8 1 µM_x000d_ | 0.0 |
| Ng 14-9 1 µM_x000d_ | 0.0 |
| Ng 14-9 3 µM_x000d_ | 0.0 |
| Ng 14-9 10 µM_x000d_ | 0.0 |
| Ng 14-9 30 µM_x000d_ | 0.0 |
| Ng 15-10 1µM_x000d_ | 0.0 |
| Ng 16-9 1 µM_x000d_ | 0.0 |
| Ng 17-8 1 µM_x000d_ | 0.0 |
| Ng 17-8 3 µM_x000d_ | 0.0 |
| Ng 18-9 1µM_x000d_ | 837.6666666666666 |
| Ng 19-11 1 µM_x000d_ | 0.0 |
| Ng 19-11 3 µM_x000d_ | 0.0 |
| Lg 17-15 HF 10 µM_x000d_ | 0.0 |
| Lg 15-17 1 µM_x000d_ | 0.0 |
| Lg 17-15 1 µM_x000d_ | 0.0 |
| Lg 17-15 3 µM_x000d_ | 0.0 |
| Lg 17-15 10 µM_x000d_ | 0.0 |
| Lg 17-17 1 µM_x000d_ | 0.0 |
| Lg 17-16 1 µM_x000d_ | 0.0 |
| Lg 16-17 3 µM_x000d_ | 0.0 |
| Lg 18-8 1 µM_x000d_ | 0.0 |
| Lg 19-8 1 µM_x000d_ | 0.0 |
| Lg 19-8 3 µM_x000d_ | 0.0 |
| Lg 19-9 1 µM_x000d_ | 0.0 |
| Lg 20-10_x000d_ | 0.0 |
| Lg 22-12 1 µM_x000d_ | 0.0 |
| Lg 20-9 1 µM_x000d_ | 0.0 |
| Lg 23-11 1 µM_x000d_ | 0.0 |
| Lg 23-11 3 µM_x000d_ | 0.0 |
| Lg 23-12_x000d_ | 0.0 |
| Lg 26-8_x000d_ | 0.0 |
| Lg 26-9_x000d_ | 0.0 |
| Lg 22-13_x000d_ | 0.0 |
| Lg 26-10_x000d_ | 26.666666666666657 |
| Lg 18-7-F3 HF 1uM | 0.0 |
| Lg 19-8-F5 HF 3uM | 0.0 |
| Lg 23-11-F2 HF 3uM | 0.0 |
| Blank 1 | 0.0 |
| Blank 2 | 0.0 |
| Blank 3 | 0.0 |
| Blank 4 | 0.0 |
| Blank 5 | 0.0 |MFI
### Chart: Un-G4
| Category | UG 000-224 IgG4 |
|---|---|
| Ng 7-13 1µM_x000d_ | 0.0 |
| Ng 7-13 3µM_x000d_ | 0.0 |
| Ng 9-9 1 µM_x000d_ | 0.0 |
| Ng 9-9 3µM_x000d_ | 0.0 |
| Ng 9-9 10µM_x000d_ | 0.0 |
| Ng 10-13 1 µM_x000d_ | 0.0 |
| Ng 10-13 3 µM_x000d_ | 0.0 |
| Ng 10-13 10 µM_x000d_ | 0.0 |
| Ng 10-13 30 µM_x000d_ | 0.0 |
| Ng 10-10 1 µM_x000d_ | 0.0 |
| Ng 10-10 3 µM_x000d_ | 0.0 |
| Ng 10-11 1 µM_x000d_ | 0.0 |
| Ng 10-11 3 µM_x000d_ | 0.0 |
| Ng 10-12 1 µM_x000d_ | 0.0 |
| Ng 11-10 1 µM_x000d_ | 0.0 |
| Ng 11-10 3 µM_x000d_ | 0.0 |
| Ng 11-11 1 µM_x000d_ | 0.0 |
| Ng 11-11 3µM_x000d_ | 0.0 |
| Ng 11-11 10µM_x000d_ | 0.0 |
| Ng 12-11 1 µM_x000d_ | 0.0 |
| Ng 12-11 3 µM_x000d_ | 0.0 |
| Ng 13-15 1 µM_x000d_ | 0.0 |
| Ng 13-15 3 µM_x000d_ | 0.0 |
| Ng 13-14_x000d_ | 0.0 |
| Ng 14-10 1 µM_x000d_ | 0.0 |
| Ng 14-10 3 µM_x000d_ | 0.0 |
| Ng 14-11 1 µM_x000d_ | 0.0 |
| Ng 14-12 1 µM_x000d_ | 0.0 |
| Ng 14-7 1 µM_x000d_ | 0.0 |
| Ng 15-14/15 1 µM_x000d_ | 0.0 |
| Ng 15-12 1µM_x000d_ | 62.66666666666666 |
| Ng 17-7 1 µM_x000d_ | 0.0 |
| Ng 17-7 3 µM_x000d_ | 0.0 |
| Ng 17-7 10 µM_x000d_ | 0.0 |
| Ng 19-9 1 µM_x000d_ | 14.0 |
| Ng 19-9 3 µM_x000d_ | 0.0 |
| Ng 19-9 10 µM_x000d_ | 0.0 |
| Ng 20-7 1 µM_x000d_ | 0.0 |
| Ng 20-7 3 µM_x000d_ | 0.0 |
| Ng 20-7 10 µM_x000d_ | 0.0 |
| Ng 21-8 1 µM_x000d_ | 0.0 |
| Ng 21-8 3 µM_x000d_ | 0.0 |
| Ng 21-8 10 µM_x000d_ | 0.0 |
| Ng 15-13 3µM_x000d_ | 1682.0 |
| Ng 16-12 1 µM_x000d_ | 0.0 |
| Ng 17-9 1 µM_x000d_ | 0.0 |
| Ng 17-9 3 µM_x000d_ | 0.0 |
| Ng 18-11 1 µM_x000d_ | 0.0 |
| Ng 18-11 3 µM_x000d_ | 0.0 |
| Ng 20-8 1 µM_x000d_ | 0.0 |
| Ng 20-9 1 µM_x000d_ | 0.0 |
| Ng 20-9 3 µM_x000d_ | 0.0 |
| Ng 21-9 1 µM_x000d_ | 0.0 |
| Ng 13-13 3 µM_x000d_ | 0.0 |
| Ng 14-8 1 µM_x000d_ | 0.0 |
| Ng 14-9 1 µM_x000d_ | 0.0 |
| Ng 14-9 3 µM_x000d_ | 0.0 |
| Ng 14-9 10 µM_x000d_ | 0.0 |
| Ng 14-9 30 µM_x000d_ | 0.0 |
| Ng 15-10 1µM_x000d_ | 0.0 |
| Ng 16-9 1 µM_x000d_ | 60.0 |
| Ng 17-8 1 µM_x000d_ | 0.0 |
| Ng 17-8 3 µM_x000d_ | 0.0 |
| Ng 18-9 1µM_x000d_ | 1315.6666666666667 |
| Ng 19-11 1 µM_x000d_ | 0.0 |
| Ng 19-11 3 µM_x000d_ | 0.0 |
| Lg 17-15 HF 10 µM_x000d_ | 0.0 |
| Lg 15-17 1 µM_x000d_ | 0.0 |
| Lg 17-15 1 µM_x000d_ | 0.0 |
| Lg 17-15 3 µM_x000d_ | 0.0 |
| Lg 17-15 10 µM_x000d_ | 0.0 |
| Lg 17-17 1 µM_x000d_ | 0.0 |
| Lg 17-16 1 µM_x000d_ | 0.0 |
| Lg 16-17 3 µM_x000d_ | 0.0 |
| Lg 18-8 1 µM_x000d_ | 0.0 |
| Lg 19-8 1 µM_x000d_ | 0.0 |
| Lg 19-8 3 µM_x000d_ | 0.0 |
| Lg 19-9 1 µM_x000d_ | 0.0 |
| Lg 20-10_x000d_ | 0.0 |
| Lg 22-12 1 µM_x000d_ | 0.0 |
| Lg 20-9 1 µM_x000d_ | 0.0 |
| Lg 23-11 1 µM_x000d_ | 0.0 |
| Lg 23-11 3 µM_x000d_ | 0.0 |
| Lg 23-12_x000d_ | 0.0 |
| Lg 26-8_x000d_ | 0.0 |
| Lg 26-9_x000d_ | 0.0 |
| Lg 22-13_x000d_ | 0.0 |
| Lg 26-10_x000d_ | 0.0 |
| Lg 18-7-F3 HF 1uM | 0.0 |
| Lg 19-8-F5 HF 3uM | 0.0 |
| Lg 23-11-F2 HF 3uM | 0.0 |
| Blank 1 | 0.0 |
| Blank 2 | 0.0 |
| Blank 3 | 0.0 |
| Blank 4 | 0.0 |
| Blank 5 | 0.0 |MFI
### Chart: Un-G5
| Category | UG 000-225 IgG4 |
|---|---|
| Ng 7-13 1µM_x000d_ | 0.0 |
| Ng 7-13 3µM_x000d_ | 0.0 |
| Ng 9-9 1 µM_x000d_ | 0.0 |
| Ng 9-9 3µM_x000d_ | 0.0 |
| Ng 9-9 10µM_x000d_ | 0.0 |
| Ng 10-13 1 µM_x000d_ | 0.0 |
| Ng 10-13 3 µM_x000d_ | 0.0 |
| Ng 10-13 10 µM_x000d_ | 0.0 |
| Ng 10-13 30 µM_x000d_ | 0.0 |
| Ng 10-10 1 µM_x000d_ | 0.0 |
| Ng 10-10 3 µM_x000d_ | 0.0 |
| Ng 10-11 1 µM_x000d_ | 0.0 |
| Ng 10-11 3 µM_x000d_ | 0.0 |
| Ng 10-12 1 µM_x000d_ | 0.0 |
| Ng 11-10 1 µM_x000d_ | 0.0 |
| Ng 11-10 3 µM_x000d_ | 0.0 |
| Ng 11-11 1 µM_x000d_ | 0.0 |
| Ng 11-11 3µM_x000d_ | 0.0 |
| Ng 11-11 10µM_x000d_ | 0.0 |
| Ng 12-11 1 µM_x000d_ | 0.0 |
| Ng 12-11 3 µM_x000d_ | 0.0 |
| Ng 13-15 1 µM_x000d_ | 0.0 |
| Ng 13-15 3 µM_x000d_ | 0.0 |
| Ng 13-14_x000d_ | 0.0 |
| Ng 14-10 1 µM_x000d_ | 0.0 |
| Ng 14-10 3 µM_x000d_ | 0.0 |
| Ng 14-11 1 µM_x000d_ | 0.0 |
| Ng 14-12 1 µM_x000d_ | 0.0 |
| Ng 14-7 1 µM_x000d_ | 0.0 |
| Ng 15-14/15 1 µM_x000d_ | 0.0 |
| Ng 15-12 1µM_x000d_ | 100.0 |
| Ng 17-7 1 µM_x000d_ | 0.0 |
| Ng 17-7 3 µM_x000d_ | 0.0 |
| Ng 17-7 10 µM_x000d_ | 0.0 |
| Ng 19-9 1 µM_x000d_ | 0.0 |
| Ng 19-9 3 µM_x000d_ | 0.0 |
| Ng 19-9 10 µM_x000d_ | 0.0 |
| Ng 20-7 1 µM_x000d_ | 0.0 |
| Ng 20-7 3 µM_x000d_ | 0.0 |
| Ng 20-7 10 µM_x000d_ | 0.0 |
| Ng 21-8 1 µM_x000d_ | 0.0 |
| Ng 21-8 3 µM_x000d_ | 0.0 |
| Ng 21-8 10 µM_x000d_ | 0.0 |
| Ng 15-13 3µM_x000d_ | 1269.6666666666667 |
| Ng 16-12 1 µM_x000d_ | 0.0 |
| Ng 17-9 1 µM_x000d_ | 0.0 |
| Ng 17-9 3 µM_x000d_ | 0.0 |
| Ng 18-11 1 µM_x000d_ | 0.0 |
| Ng 18-11 3 µM_x000d_ | 0.0 |
| Ng 20-8 1 µM_x000d_ | 0.0 |
| Ng 20-9 1 µM_x000d_ | 0.0 |
| Ng 20-9 3 µM_x000d_ | 0.0 |
| Ng 21-9 1 µM_x000d_ | 0.0 |
| Ng 13-13 3 µM_x000d_ | 0.0 |
| Ng 14-8 1 µM_x000d_ | 0.0 |
| Ng 14-9 1 µM_x000d_ | 0.0 |
| Ng 14-9 3 µM_x000d_ | 0.0 |
| Ng 14-9 10 µM_x000d_ | 0.0 |
| Ng 14-9 30 µM_x000d_ | 0.0 |
| Ng 15-10 1µM_x000d_ | 0.0 |
| Ng 16-9 1 µM_x000d_ | 2.333333333333343 |
| Ng 17-8 1 µM_x000d_ | 0.0 |
| Ng 17-8 3 µM_x000d_ | 0.0 |
| Ng 18-9 1µM_x000d_ | 790.6666666666666 |
| Ng 19-11 1 µM_x000d_ | 0.0 |
| Ng 19-11 3 µM_x000d_ | 0.0 |
| Lg 17-15 HF 10 µM_x000d_ | 0.0 |
| Lg 15-17 1 µM_x000d_ | 0.0 |
| Lg 17-15 1 µM_x000d_ | 0.0 |
| Lg 17-15 3 µM_x000d_ | 0.0 |
| Lg 17-15 10 µM_x000d_ | 0.0 |
| Lg 17-17 1 µM_x000d_ | 0.0 |
| Lg 17-16 1 µM_x000d_ | 0.0 |
| Lg 16-17 3 µM_x000d_ | 0.0 |
| Lg 18-8 1 µM_x000d_ | 0.0 |
| Lg 19-8 1 µM_x000d_ | 0.0 |
| Lg 19-8 3 µM_x000d_ | 0.0 |
| Lg 19-9 1 µM_x000d_ | 0.0 |
| Lg 20-10_x000d_ | 0.0 |
| Lg 22-12 1 µM_x000d_ | 0.0 |
| Lg 20-9 1 µM_x000d_ | 0.0 |
| Lg 23-11 1 µM_x000d_ | 0.0 |
| Lg 23-11 3 µM_x000d_ | 0.0 |
| Lg 23-12_x000d_ | 0.0 |
| Lg 26-8_x000d_ | 0.0 |
| Lg 26-9_x000d_ | 0.0 |
| Lg 22-13_x000d_ | 0.0 |
| Lg 26-10_x000d_ | 0.0 |
| Lg 18-7-F3 HF 1uM | 0.0 |
| Lg 19-8-F5 HF 3uM | 0.0 |
| Lg 23-11-F2 HF 3uM | 0.0 |
| Blank 1 | 0.0 |
| Blank 2 | 0.0 |
| Blank 3 | 0.0 |
| Blank 4 | 0.0 |
| Blank 5 | 0.0 |MFI
### Chart: Un-G6
| Category | UG 000-226 IgG4 |
|---|---|
| Ng 7-13 1µM_x000d_ | 0.0 |
| Ng 7-13 3µM_x000d_ | 0.0 |
| Ng 9-9 1 µM_x000d_ | 0.0 |
| Ng 9-9 3µM_x000d_ | 0.0 |
| Ng 9-9 10µM_x000d_ | 0.0 |
| Ng 10-13 1 µM_x000d_ | 0.0 |
| Ng 10-13 3 µM_x000d_ | 1274.3333333333333 |
| Ng 10-13 10 µM_x000d_ | 49.66666666666666 |
| Ng 10-13 30 µM_x000d_ | 0.0 |
| Ng 10-10 1 µM_x000d_ | 0.0 |
| Ng 10-10 3 µM_x000d_ | 0.0 |
| Ng 10-11 1 µM_x000d_ | 0.0 |
| Ng 10-11 3 µM_x000d_ | 0.0 |
| Ng 10-12 1 µM_x000d_ | 0.0 |
| Ng 11-10 1 µM_x000d_ | 0.0 |
| Ng 11-10 3 µM_x000d_ | 0.0 |
| Ng 11-11 1 µM_x000d_ | 0.0 |
| Ng 11-11 3µM_x000d_ | 0.0 |
| Ng 11-11 10µM_x000d_ | 0.0 |
| Ng 12-11 1 µM_x000d_ | 0.0 |
| Ng 12-11 3 µM_x000d_ | 0.0 |
| Ng 13-15 1 µM_x000d_ | 1226.6666666666667 |
| Ng 13-15 3 µM_x000d_ | 0.0 |
| Ng 13-14_x000d_ | 0.0 |
| Ng 14-10 1 µM_x000d_ | 0.0 |
| Ng 14-10 3 µM_x000d_ | 0.0 |
| Ng 14-11 1 µM_x000d_ | 0.0 |
| Ng 14-12 1 µM_x000d_ | 0.0 |
| Ng 14-7 1 µM_x000d_ | 0.0 |
| Ng 15-14/15 1 µM_x000d_ | 0.0 |
| Ng 15-12 1µM_x000d_ | 0.0 |
| Ng 17-7 1 µM_x000d_ | 0.0 |
| Ng 17-7 3 µM_x000d_ | 0.0 |
| Ng 17-7 10 µM_x000d_ | 0.0 |
| Ng 19-9 1 µM_x000d_ | 0.0 |
| Ng 19-9 3 µM_x000d_ | 0.0 |
| Ng 19-9 10 µM_x000d_ | 0.0 |
| Ng 20-7 1 µM_x000d_ | 292.0 |
| Ng 20-7 3 µM_x000d_ | 0.0 |
| Ng 20-7 10 µM_x000d_ | 0.0 |
| Ng 21-8 1 µM_x000d_ | 0.0 |
| Ng 21-8 3 µM_x000d_ | 0.0 |
| Ng 21-8 10 µM_x000d_ | 0.0 |
| Ng 15-13 3µM_x000d_ | 0.0 |
| Ng 16-12 1 µM_x000d_ | 0.0 |
| Ng 17-9 1 µM_x000d_ | 0.0 |
| Ng 17-9 3 µM_x000d_ | 0.0 |
| Ng 18-11 1 µM_x000d_ | 0.0 |
| Ng 18-11 3 µM_x000d_ | 0.0 |
| Ng 20-8 1 µM_x000d_ | 0.0 |
| Ng 20-9 1 µM_x000d_ | 0.0 |
| Ng 20-9 3 µM_x000d_ | 0.0 |
| Ng 21-9 1 µM_x000d_ | 0.0 |
| Ng 13-13 3 µM_x000d_ | 0.0 |
| Ng 14-8 1 µM_x000d_ | 0.0 |
| Ng 14-9 1 µM_x000d_ | 0.0 |
| Ng 14-9 3 µM_x000d_ | 0.0 |
| Ng 14-9 10 µM_x000d_ | 0.0 |
| Ng 14-9 30 µM_x000d_ | 0.0 |
| Ng 15-10 1µM_x000d_ | 0.0 |
| Ng 16-9 1 µM_x000d_ | 0.0 |
| Ng 17-8 1 µM_x000d_ | 0.0 |
| Ng 17-8 3 µM_x000d_ | 0.0 |
| Ng 18-9 1µM_x000d_ | 0.0 |
| Ng 19-11 1 µM_x000d_ | 0.0 |
| Ng 19-11 3 µM_x000d_ | 0.0 |
| Lg 17-15 HF 10 µM_x000d_ | 1.0 |
| Lg 15-17 1 µM_x000d_ | 0.0 |
| Lg 17-15 1 µM_x000d_ | 0.0 |
| Lg 17-15 3 µM_x000d_ | 0.0 |
| Lg 17-15 10 µM_x000d_ | 0.0 |
| Lg 17-17 1 µM_x000d_ | 0.0 |
| Lg 17-16 1 µM_x000d_ | 0.0 |
| Lg 16-17 3 µM_x000d_ | 0.0 |
| Lg 18-8 1 µM_x000d_ | 0.0 |
| Lg 19-8 1 µM_x000d_ | 0.0 |
| Lg 19-8 3 µM_x000d_ | 0.0 |
| Lg 19-9 1 µM_x000d_ | 0.0 |
| Lg 20-10_x000d_ | 0.0 |
| Lg 22-12 1 µM_x000d_ | 0.0 |
| Lg 20-9 1 µM_x000d_ | 0.0 |
| Lg 23-11 1 µM_x000d_ | 0.0 |
| Lg 23-11 3 µM_x000d_ | 0.0 |
| Lg 23-12_x000d_ | 0.0 |
| Lg 26-8_x000d_ | 0.0 |
| Lg 26-9_x000d_ | 0.0 |
| Lg 22-13_x000d_ | 0.0 |
| Lg 26-10_x000d_ | 0.0 |
| Lg 18-7-F3 HF 1uM | 0.0 |
| Lg 19-8-F5 HF 3uM | 0.0 |
| Lg 23-11-F2 HF 3uM | 0.0 |
| Blank 1 | 0.0 |
| Blank 2 | 0.0 |
| Blank 3 | 37.33333333333334 |
| Blank 4 | 0.0 |
| Blank 5 | 0.0 |MFI
### Chart: Un-NL1
| Category | NLD-57 IgG4 |
|---|---|
| Ng 7-13 1µM_x000d_ | 0.0 |
| Ng 7-13 3µM_x000d_ | 0.0 |
| Ng 9-9 1 µM_x000d_ | 0.0 |
| Ng 9-9 3µM_x000d_ | 0.0 |
| Ng 9-9 10µM_x000d_ | 0.0 |
| Ng 10-13 1 µM_x000d_ | 0.0 |
| Ng 10-13 3 µM_x000d_ | 0.0 |
| Ng 10-13 10 µM_x000d_ | 0.0 |
| Ng 10-13 30 µM_x000d_ | 0.0 |
| Ng 10-10 1 µM_x000d_ | 0.0 |
| Ng 10-10 3 µM_x000d_ | 0.0 |
| Ng 10-11 1 µM_x000d_ | 0.0 |
| Ng 10-11 3 µM_x000d_ | 0.0 |
| Ng 10-12 1 µM_x000d_ | 0.0 |
| Ng 11-10 1 µM_x000d_ | 0.0 |
| Ng 11-10 3 µM_x000d_ | 0.0 |
| Ng 11-11 1 µM_x000d_ | 0.0 |
| Ng 11-11 3µM_x000d_ | 0.0 |
| Ng 11-11 10µM_x000d_ | 0.0 |
| Ng 12-11 1 µM_x000d_ | 0.0 |
| Ng 12-11 3 µM_x000d_ | 0.0 |
| Ng 13-15 1 µM_x000d_ | 0.0 |
| Ng 13-15 3 µM_x000d_ | 0.0 |
| Ng 13-14_x000d_ | 0.0 |
| Ng 14-10 1 µM_x000d_ | 0.0 |
| Ng 14-10 3 µM_x000d_ | 0.0 |
| Ng 14-11 1 µM_x000d_ | 0.0 |
| Ng 14-12 1 µM_x000d_ | 0.0 |
| Ng 14-7 1 µM_x000d_ | 0.0 |
| Ng 15-14/15 1 µM_x000d_ | 0.0 |
| Ng 15-12 1µM_x000d_ | 43.0 |
| Ng 17-7 1 µM_x000d_ | 0.0 |
| Ng 17-7 3 µM_x000d_ | 0.0 |
| Ng 17-7 10 µM_x000d_ | 0.0 |
| Ng 19-9 1 µM_x000d_ | 0.0 |
| Ng 19-9 3 µM_x000d_ | 0.0 |
| Ng 19-9 10 µM_x000d_ | 0.0 |
| Ng 20-7 1 µM_x000d_ | 0.0 |
| Ng 20-7 3 µM_x000d_ | 0.0 |
| Ng 20-7 10 µM_x000d_ | 0.0 |
| Ng 21-8 1 µM_x000d_ | 0.0 |
| Ng 21-8 3 µM_x000d_ | 0.0 |
| Ng 21-8 10 µM_x000d_ | 0.0 |
| Ng 15-13 3µM_x000d_ | 1014.6666666666667 |
| Ng 16-12 1 µM_x000d_ | 0.0 |
| Ng 17-9 1 µM_x000d_ | 0.0 |
| Ng 17-9 3 µM_x000d_ | 0.0 |
| Ng 18-11 1 µM_x000d_ | 0.0 |
| Ng 18-11 3 µM_x000d_ | 0.0 |
| Ng 20-8 1 µM_x000d_ | 384.66666666666663 |
| Ng 20-9 1 µM_x000d_ | 0.0 |
| Ng 20-9 3 µM_x000d_ | 0.0 |
| Ng 21-9 1 µM_x000d_ | 0.0 |
| Ng 13-13 3 µM_x000d_ | 0.0 |
| Ng 14-8 1 µM_x000d_ | 0.0 |
| Ng 14-9 1 µM_x000d_ | 0.0 |
| Ng 14-9 3 µM_x000d_ | 0.0 |
| Ng 14-9 10 µM_x000d_ | 0.0 |
| Ng 14-9 30 µM_x000d_ | 0.0 |
| Ng 15-10 1µM_x000d_ | 0.0 |
| Ng 16-9 1 µM_x000d_ | 0.0 |
| Ng 17-8 1 µM_x000d_ | 0.0 |
| Ng 17-8 3 µM_x000d_ | 0.0 |
| Ng 18-9 1µM_x000d_ | 602.0 |
| Ng 19-11 1 µM_x000d_ | 0.0 |
| Ng 19-11 3 µM_x000d_ | 0.0 |
| Lg 17-15 HF 10 µM_x000d_ | 0.0 |
| Lg 15-17 1 µM_x000d_ | 0.0 |
| Lg 17-15 1 µM_x000d_ | 0.0 |
| Lg 17-15 3 µM_x000d_ | 0.0 |
| Lg 17-15 10 µM_x000d_ | 0.0 |
| Lg 17-17 1 µM_x000d_ | 0.0 |
| Lg 17-16 1 µM_x000d_ | 0.0 |
| Lg 16-17 3 µM_x000d_ | 0.0 |
| Lg 18-8 1 µM_x000d_ | 0.0 |
| Lg 19-8 1 µM_x000d_ | 0.0 |
| Lg 19-8 3 µM_x000d_ | 0.0 |
| Lg 19-9 1 µM_x000d_ | 0.0 |
| Lg 20-10_x000d_ | 0.0 |
| Lg 22-12 1 µM_x000d_ | 0.0 |
| Lg 20-9 1 µM_x000d_ | 0.0 |
| Lg 23-11 1 µM_x000d_ | 0.0 |
| Lg 23-11 3 µM_x000d_ | 0.0 |
| Lg 23-12_x000d_ | 0.0 |
| Lg 26-8_x000d_ | 0.0 |
| Lg 26-9_x000d_ | 0.0 |
| Lg 22-13_x000d_ | 0.0 |
| Lg 26-10_x000d_ | 41.33333333333334 |
| Lg 18-7-F3 HF 1uM | 0.0 |
| Lg 19-8-F5 HF 3uM | 0.0 |
| Lg 23-11-F2 HF 3uM | 0.0 |
| Blank 1 | 0.0 |
| Blank 2 | 0.0 |
| Blank 3 | 0.0 |
| Blank 4 | 0.0 |
| Blank 5 | 0.0 |MFI
### Chart: Un-NL2
| Category | NLD-59 IgG4 |
|---|---|
| Ng 7-13 1µM_x000d_ | 0.0 |
| Ng 7-13 3µM_x000d_ | 0.0 |
| Ng 9-9 1 µM_x000d_ | 0.0 |
| Ng 9-9 3µM_x000d_ | 0.0 |
| Ng 9-9 10µM_x000d_ | 0.0 |
| Ng 10-13 1 µM_x000d_ | 0.0 |
| Ng 10-13 3 µM_x000d_ | 0.0 |
| Ng 10-13 10 µM_x000d_ | 0.0 |
| Ng 10-13 30 µM_x000d_ | 0.0 |
| Ng 10-10 1 µM_x000d_ | 0.0 |
| Ng 10-10 3 µM_x000d_ | 0.0 |
| Ng 10-11 1 µM_x000d_ | 0.0 |
| Ng 10-11 3 µM_x000d_ | 0.0 |
| Ng 10-12 1 µM_x000d_ | 0.0 |
| Ng 11-10 1 µM_x000d_ | 0.0 |
| Ng 11-10 3 µM_x000d_ | 0.0 |
| Ng 11-11 1 µM_x000d_ | 0.0 |
| Ng 11-11 3µM_x000d_ | 0.0 |
| Ng 11-11 10µM_x000d_ | 0.0 |
| Ng 12-11 1 µM_x000d_ | 0.0 |
| Ng 12-11 3 µM_x000d_ | 0.0 |
| Ng 13-15 1 µM_x000d_ | 0.0 |
| Ng 13-15 3 µM_x000d_ | 0.0 |
| Ng 13-14_x000d_ | 0.0 |
| Ng 14-10 1 µM_x000d_ | 0.0 |
| Ng 14-10 3 µM_x000d_ | 0.0 |
| Ng 14-11 1 µM_x000d_ | 0.0 |
| Ng 14-12 1 µM_x000d_ | 0.0 |
| Ng 14-7 1 µM_x000d_ | 0.0 |
| Ng 15-14/15 1 µM_x000d_ | 0.0 |
| Ng 15-12 1µM_x000d_ | 111.66666666666669 |
| Ng 17-7 1 µM_x000d_ | 0.0 |
| Ng 17-7 3 µM_x000d_ | 0.0 |
| Ng 17-7 10 µM_x000d_ | 0.0 |
| Ng 19-9 1 µM_x000d_ | 0.0 |
| Ng 19-9 3 µM_x000d_ | 0.0 |
| Ng 19-9 10 µM_x000d_ | 0.0 |
| Ng 20-7 1 µM_x000d_ | 0.0 |
| Ng 20-7 3 µM_x000d_ | 0.0 |
| Ng 20-7 10 µM_x000d_ | 0.0 |
| Ng 21-8 1 µM_x000d_ | 0.0 |
| Ng 21-8 3 µM_x000d_ | 0.0 |
| Ng 21-8 10 µM_x000d_ | 0.0 |
| Ng 15-13 3µM_x000d_ | 1301.0 |
| Ng 16-12 1 µM_x000d_ | 0.0 |
| Ng 17-9 1 µM_x000d_ | 0.0 |
| Ng 17-9 3 µM_x000d_ | 0.0 |
| Ng 18-11 1 µM_x000d_ | 0.0 |
| Ng 18-11 3 µM_x000d_ | 0.0 |
| Ng 20-8 1 µM_x000d_ | 0.0 |
| Ng 20-9 1 µM_x000d_ | 0.0 |
| Ng 20-9 3 µM_x000d_ | 0.0 |
| Ng 21-9 1 µM_x000d_ | 0.0 |
| Ng 13-13 3 µM_x000d_ | 0.0 |
| Ng 14-8 1 µM_x000d_ | 0.0 |
| Ng 14-9 1 µM_x000d_ | 0.0 |
| Ng 14-9 3 µM_x000d_ | 0.0 |
| Ng 14-9 10 µM_x000d_ | 0.0 |
| Ng 14-9 30 µM_x000d_ | 0.0 |
| Ng 15-10 1µM_x000d_ | 0.0 |
| Ng 16-9 1 µM_x000d_ | 0.0 |
| Ng 17-8 1 µM_x000d_ | 0.0 |
| Ng 17-8 3 µM_x000d_ | 0.0 |
| Ng 18-9 1µM_x000d_ | 1051.6666666666667 |
| Ng 19-11 1 µM_x000d_ | 0.0 |
| Ng 19-11 3 µM_x000d_ | 0.0 |
| Lg 17-15 HF 10 µM_x000d_ | 0.0 |
| Lg 15-17 1 µM_x000d_ | 0.0 |
| Lg 17-15 1 µM_x000d_ | 0.0 |
| Lg 17-15 3 µM_x000d_ | 0.0 |
| Lg 17-15 10 µM_x000d_ | 0.0 |
| Lg 17-17 1 µM_x000d_ | 0.0 |
| Lg 17-16 1 µM_x000d_ | 0.0 |
| Lg 16-17 3 µM_x000d_ | 0.0 |
| Lg 18-8 1 µM_x000d_ | 0.0 |
| Lg 19-8 1 µM_x000d_ | 0.0 |
| Lg 19-8 3 µM_x000d_ | 0.0 |
| Lg 19-9 1 µM_x000d_ | 0.0 |
| Lg 20-10_x000d_ | 0.0 |
| Lg 22-12 1 µM_x000d_ | 0.0 |
| Lg 20-9 1 µM_x000d_ | 0.0 |
| Lg 23-11 1 µM_x000d_ | 0.0 |
| Lg 23-11 3 µM_x000d_ | 0.0 |
| Lg 23-12_x000d_ | 0.0 |
| Lg 26-8_x000d_ | 0.0 |
| Lg 26-9_x000d_ | 0.0 |
| Lg 22-13_x000d_ | 0.0 |
| Lg 26-10_x000d_ | 0.0 |
| Lg 18-7-F3 HF 1uM | 0.0 |
| Lg 19-8-F5 HF 3uM | 0.0 |
| Lg 23-11-F2 HF 3uM | 0.0 |
| Blank 1 | 0.0 |
| Blank 2 | 0.0 |
| Blank 3 | 0.0 |
| Blank 4 | 0.0 |
| Blank 5 | 0.0 |MFI
### Chart: Un-NL3
| Category | NLD-60 IgG4 |
|---|---|
| Ng 7-13 1µM_x000d_ | 0.0 |
| Ng 7-13 3µM_x000d_ | 0.0 |
| Ng 9-9 1 µM_x000d_ | 0.0 |
| Ng 9-9 3µM_x000d_ | 0.0 |
| Ng 9-9 10µM_x000d_ | 0.0 |
| Ng 10-13 1 µM_x000d_ | 0.0 |
| Ng 10-13 3 µM_x000d_ | 0.0 |
| Ng 10-13 10 µM_x000d_ | 0.0 |
| Ng 10-13 30 µM_x000d_ | 0.0 |
| Ng 10-10 1 µM_x000d_ | 0.0 |
| Ng 10-10 3 µM_x000d_ | 0.0 |
| Ng 10-11 1 µM_x000d_ | 0.0 |
| Ng 10-11 3 µM_x000d_ | 0.0 |
| Ng 10-12 1 µM_x000d_ | 0.0 |
| Ng 11-10 1 µM_x000d_ | 0.0 |
| Ng 11-10 3 µM_x000d_ | 0.0 |
| Ng 11-11 1 µM_x000d_ | 0.0 |
| Ng 11-11 3µM_x000d_ | 0.0 |
| Ng 11-11 10µM_x000d_ | 0.0 |
| Ng 12-11 1 µM_x000d_ | 0.0 |
| Ng 12-11 3 µM_x000d_ | 0.0 |
| Ng 13-15 1 µM_x000d_ | 0.0 |
| Ng 13-15 3 µM_x000d_ | 0.0 |
| Ng 13-14_x000d_ | 0.0 |
| Ng 14-10 1 µM_x000d_ | 0.0 |
| Ng 14-10 3 µM_x000d_ | 0.0 |
| Ng 14-11 1 µM_x000d_ | 0.0 |
| Ng 14-12 1 µM_x000d_ | 0.0 |
| Ng 14-7 1 µM_x000d_ | 0.0 |
| Ng 15-14/15 1 µM_x000d_ | 0.0 |
| Ng 15-12 1µM_x000d_ | 69.33333333333334 |
| Ng 17-7 1 µM_x000d_ | 0.0 |
| Ng 17-7 3 µM_x000d_ | 0.0 |
| Ng 17-7 10 µM_x000d_ | 0.0 |
| Ng 19-9 1 µM_x000d_ | 0.0 |
| Ng 19-9 3 µM_x000d_ | 0.0 |
| Ng 19-9 10 µM_x000d_ | 0.0 |
| Ng 20-7 1 µM_x000d_ | 0.0 |
| Ng 20-7 3 µM_x000d_ | 0.0 |
| Ng 20-7 10 µM_x000d_ | 0.0 |
| Ng 21-8 1 µM_x000d_ | 0.0 |
| Ng 21-8 3 µM_x000d_ | 0.0 |
| Ng 21-8 10 µM_x000d_ | 0.0 |
| Ng 15-13 3µM_x000d_ | 1028.3333333333333 |
| Ng 16-12 1 µM_x000d_ | 0.0 |
| Ng 17-9 1 µM_x000d_ | 0.0 |
| Ng 17-9 3 µM_x000d_ | 0.0 |
| Ng 18-11 1 µM_x000d_ | 0.0 |
| Ng 18-11 3 µM_x000d_ | 0.0 |
| Ng 20-8 1 µM_x000d_ | 0.0 |
| Ng 20-9 1 µM_x000d_ | 0.0 |
| Ng 20-9 3 µM_x000d_ | 0.0 |
| Ng 21-9 1 µM_x000d_ | 0.0 |
| Ng 13-13 3 µM_x000d_ | 0.0 |
| Ng 14-8 1 µM_x000d_ | 0.0 |
| Ng 14-9 1 µM_x000d_ | 0.0 |
| Ng 14-9 3 µM_x000d_ | 0.0 |
| Ng 14-9 10 µM_x000d_ | 0.0 |
| Ng 14-9 30 µM_x000d_ | 0.0 |
| Ng 15-10 1µM_x000d_ | 0.0 |
| Ng 16-9 1 µM_x000d_ | 0.0 |
| Ng 17-8 1 µM_x000d_ | 0.0 |
| Ng 17-8 3 µM_x000d_ | 0.0 |
| Ng 18-9 1µM_x000d_ | 792.3333333333334 |
| Ng 19-11 1 µM_x000d_ | 0.0 |
| Ng 19-11 3 µM_x000d_ | 0.0 |
| Lg 17-15 HF 10 µM_x000d_ | 0.0 |
| Lg 15-17 1 µM_x000d_ | 0.0 |
| Lg 17-15 1 µM_x000d_ | 8.666666666666657 |
| Lg 17-15 3 µM_x000d_ | 0.0 |
| Lg 17-15 10 µM_x000d_ | 0.0 |
| Lg 17-17 1 µM_x000d_ | 0.0 |
| Lg 17-16 1 µM_x000d_ | 0.0 |
| Lg 16-17 3 µM_x000d_ | 0.0 |
| Lg 18-8 1 µM_x000d_ | 0.0 |
| Lg 19-8 1 µM_x000d_ | 0.0 |
| Lg 19-8 3 µM_x000d_ | 0.0 |
| Lg 19-9 1 µM_x000d_ | 0.0 |
| Lg 20-10_x000d_ | 0.0 |
| Lg 22-12 1 µM_x000d_ | 0.0 |
| Lg 20-9 1 µM_x000d_ | 0.0 |
| Lg 23-11 1 µM_x000d_ | 0.0 |
| Lg 23-11 3 µM_x000d_ | 0.0 |
| Lg 23-12_x000d_ | 0.0 |
| Lg 26-8_x000d_ | 0.0 |
| Lg 26-9_x000d_ | 0.0 |
| Lg 22-13_x000d_ | 0.0 |
| Lg 26-10_x000d_ | 0.0 |
| Lg 18-7-F3 HF 1uM | 0.0 |
| Lg 19-8-F5 HF 3uM | 0.0 |
| Lg 23-11-F2 HF 3uM | 0.0 |
| Blank 1 | 0.0 |
| Blank 2 | 0.0 |
| Blank 3 | 0.0 |
| Blank 4 | 0.0 |
| Blank 5 | 0.0 |MFI
### Chart: Un-NL4
| Category | NLD-61 IgG4 |
|---|---|
| Ng 7-13 1µM_x000d_ | 0.0 |
| Ng 7-13 3µM_x000d_ | 0.0 |
| Ng 9-9 1 µM_x000d_ | 0.0 |
| Ng 9-9 3µM_x000d_ | 0.0 |
| Ng 9-9 10µM_x000d_ | 0.0 |
| Ng 10-13 1 µM_x000d_ | 0.0 |
| Ng 10-13 3 µM_x000d_ | 0.0 |
| Ng 10-13 10 µM_x000d_ | 0.0 |
| Ng 10-13 30 µM_x000d_ | 0.0 |
| Ng 10-10 1 µM_x000d_ | 0.0 |
| Ng 10-10 3 µM_x000d_ | 0.0 |
| Ng 10-11 1 µM_x000d_ | 0.0 |
| Ng 10-11 3 µM_x000d_ | 0.0 |
| Ng 10-12 1 µM_x000d_ | 0.0 |
| Ng 11-10 1 µM_x000d_ | 0.0 |
| Ng 11-10 3 µM_x000d_ | 0.0 |
| Ng 11-11 1 µM_x000d_ | 0.0 |
| Ng 11-11 3µM_x000d_ | 0.0 |
| Ng 11-11 10µM_x000d_ | 0.0 |
| Ng 12-11 1 µM_x000d_ | 0.0 |
| Ng 12-11 3 µM_x000d_ | 0.0 |
| Ng 13-15 1 µM_x000d_ | 0.0 |
| Ng 13-15 3 µM_x000d_ | 0.0 |
| Ng 13-14_x000d_ | 0.0 |
| Ng 14-10 1 µM_x000d_ | 0.0 |
| Ng 14-10 3 µM_x000d_ | 0.0 |
| Ng 14-11 1 µM_x000d_ | 0.0 |
| Ng 14-12 1 µM_x000d_ | 0.0 |
| Ng 14-7 1 µM_x000d_ | 0.0 |
| Ng 15-14/15 1 µM_x000d_ | 0.0 |
| Ng 15-12 1µM_x000d_ | 50.0 |
| Ng 17-7 1 µM_x000d_ | 0.0 |
| Ng 17-7 3 µM_x000d_ | 0.0 |
| Ng 17-7 10 µM_x000d_ | 0.0 |
| Ng 19-9 1 µM_x000d_ | 0.0 |
| Ng 19-9 3 µM_x000d_ | 0.0 |
| Ng 19-9 10 µM_x000d_ | 0.0 |
| Ng 20-7 1 µM_x000d_ | 0.0 |
| Ng 20-7 3 µM_x000d_ | 0.0 |
| Ng 20-7 10 µM_x000d_ | 0.0 |
| Ng 21-8 1 µM_x000d_ | 0.0 |
| Ng 21-8 3 µM_x000d_ | 0.0 |
| Ng 21-8 10 µM_x000d_ | 0.0 |
| Ng 15-13 3µM_x000d_ | 931.3333333333333 |
| Ng 16-12 1 µM_x000d_ | 0.0 |
| Ng 17-9 1 µM_x000d_ | 0.0 |
| Ng 17-9 3 µM_x000d_ | 0.0 |
| Ng 18-11 1 µM_x000d_ | 0.0 |
| Ng 18-11 3 µM_x000d_ | 0.0 |
| Ng 20-8 1 µM_x000d_ | 0.0 |
| Ng 20-9 1 µM_x000d_ | 0.0 |
| Ng 20-9 3 µM_x000d_ | 0.0 |
| Ng 21-9 1 µM_x000d_ | 0.0 |
| Ng 13-13 3 µM_x000d_ | 0.0 |
| Ng 14-8 1 µM_x000d_ | 0.0 |
| Ng 14-9 1 µM_x000d_ | 0.0 |
| Ng 14-9 3 µM_x000d_ | 0.0 |
| Ng 14-9 10 µM_x000d_ | 0.0 |
| Ng 14-9 30 µM_x000d_ | 0.0 |
| Ng 15-10 1µM_x000d_ | 0.0 |
| Ng 16-9 1 µM_x000d_ | 0.0 |
| Ng 17-8 1 µM_x000d_ | 0.0 |
| Ng 17-8 3 µM_x000d_ | 0.0 |
| Ng 18-9 1µM_x000d_ | 724.0 |
| Ng 19-11 1 µM_x000d_ | 0.0 |
| Ng 19-11 3 µM_x000d_ | 0.0 |
| Lg 17-15 HF 10 µM_x000d_ | 0.0 |
| Lg 15-17 1 µM_x000d_ | 0.0 |
| Lg 17-15 1 µM_x000d_ | 0.0 |
| Lg 17-15 3 µM_x000d_ | 0.0 |
| Lg 17-15 10 µM_x000d_ | 0.0 |
| Lg 17-17 1 µM_x000d_ | 0.0 |
| Lg 17-16 1 µM_x000d_ | 0.0 |
| Lg 16-17 3 µM_x000d_ | 0.0 |
| Lg 18-8 1 µM_x000d_ | 0.0 |
| Lg 19-8 1 µM_x000d_ | 0.0 |
| Lg 19-8 3 µM_x000d_ | 0.0 |
| Lg 19-9 1 µM_x000d_ | 0.0 |
| Lg 20-10_x000d_ | 0.0 |
| Lg 22-12 1 µM_x000d_ | 0.0 |
| Lg 20-9 1 µM_x000d_ | 0.0 |
| Lg 23-11 1 µM_x000d_ | 0.0 |
| Lg 23-11 3 µM_x000d_ | 0.0 |
| Lg 23-12_x000d_ | 0.0 |
| Lg 26-8_x000d_ | 0.0 |
| Lg 26-9_x000d_ | 0.0 |
| Lg 22-13_x000d_ | 0.0 |
| Lg 26-10_x000d_ | 0.0 |
| Lg 18-7-F3 HF 1uM | 0.0 |
| Lg 19-8-F5 HF 3uM | 0.0 |
| Lg 23-11-F2 HF 3uM | 0.0 |
| Blank 1 | 0.0 |
| Blank 2 | 0.0 |
| Blank 3 | 0.0 |
| Blank 4 | 0.0 |
| Blank 5 | 0.0 |MFI
### Chart: Un-NL5
| Category | NLD-62 IgG4 |
|---|---|
| Ng1_1uM
 | 0.0 |
| Ng1_3uM
 | 0.0 |
| Ng2_1uM | 0.0 |
| Ng2_3uM | 0.0 |
| Ng2_10uM | 0.0 |
| Ng3_1uM | 0.0 |
| Ng3_3uM | 0.0 |
| Ng3_10uM | 0.0 |
| Ng3_30uM | 0.0 |
| Ng4_1uM | 0.0 |
| Ng4_3uM | 0.0 |
| Ng5_1uM | 0.0 |
| Ng5_3uM | 0.0 |
| Ng6_1uM | 0.0 |
| Ng7_1uM | 0.0 |
| Ng7_3uM | 0.0 |
| Ng8_1uM | 0.0 |
| Ng8_3uM | 0.0 |
| Ng8_10uM | 0.0 |
| Ng9_1uM | 0.0 |
| Ng9_3uM | 0.0 |
| Ng10_3uM | 0.0 |
| Ng10_1uM | 0.0 |
| Ng11 | 0.0 |
| Ng12_1uM | 0.0 |
| Ng12_3uM | 0.0 |
| Ng13_1uM | 0.0 |
| Ng14_1uM | 0.0 |
| Ng15_1uM | 0.0 |
| Ng16_1uM | 0.0 |
| Ng17_1uM | 93.66666666666666 |
| Ng18_1uM | 0.0 |
| Ng18_3uM | 0.0 |
| Ng18_10uM | 0.0 |
| Ng19_1uM | 0.0 |
| Ng19_3uM | 0.0 |
| Ng19_10uM | 0.0 |
| Ng20_1uM | 0.0 |
| Ng20_3uM | 0.0 |
| Ng20_10uM | 0.0 |
| Ng21_1uM | 0.0 |
| Ng21 _3uM | 0.0 |
| Ng21_10uM | 0.0 |
| Ng22_1uM | 1068.6666666666667 |
| Ng23_1uM | 0.0 |
| Ng24_1uM | 0.0 |
| Ng24_3uM | 0.0 |
| Ng25_1uM | 0.0 |
| Ng25_3uM | 0.0 |
| Ng26_1uM | 0.0 |
| Ng27_1uM | 0.0 |
| Ng27_3uM | 0.0 |
| Ng28_1uM | 0.0 |
| Ng29_1uM | 0.0 |
| Ng30_1uM | 0.0 |
| Ng31_1uM | 0.0 |
| Ng31_3uM | 0.0 |
| Ng31_10uM | 0.0 |
| Ng31_30uM | 0.0 |
| Ng32_1uM | 0.0 |
| Ng33_1uM | 0.0 |
| Ng34_1uM | 0.0 |
| Ng34_3uM | 0.0 |
| Ng35_1uM | 986.3333333333333 |
| Ng36_1uM | 0.0 |
| Ng36_3uM | 0.0 |
| Lg2_HF_10uM | 0.0 |
| Lg1_1uM | 0.0 |
| Lg2_1uM | 0.0 |
| Lg2_3uM | 0.0 |
| Lg2_10uM | 0.0 |
| Lg3_1uM | 0.0 |
| Lg4_1uM | 0.0 |
| Lg5_3uM | 0.0 |
| Lg6_1uM | 0.0 |
| Lg7_1uM | 0.0 |
| Lg7_3uM | 0.0 |
| Lg8_1uM | 0.0 |
| Lg9 | 0.0 |
| Lg10_1uM | 0.0 |
| Lg11_1uM | 0.0 |
| Lg12_1uM | 0.0 |
| Lg12_3uM | 0.0 |
| Lg13 | 0.0 |
| Lg14 | 0.0 |
| Lg15 | 0.0 |
| Lg16 | 0.0 |
| Lg17 | 0.0 |
| Lg18_HF_1uM | 0.0 |
| Lg7_HF_3uM | 0.0 |
| Lg12_HF_3uM | 0.0 |
| Blank 1 | 0.0 |
| Blank 2 | 0.0 |
| Blank 3 | 0.0 |
| Blank 4 | 0.0 |
| Blank 5 | 0.0 |MFI
